# Abiraterone acetate plus prednisolone for metastatic patients starting hormone therapy: 5‐year follow‐up results from the STAMPEDE randomised trial (NCT00268476)

**DOI:** 10.1002/ijc.34018

**Published:** 2022-05-16

**Authors:** Nicholas D. James, Noel W. Clarke, Adrian Cook, Adnan Ali, Alex P. Hoyle, Gerhardt Attard, Christopher D. Brawley, Simon Chowdhury, William R. Cross, David P. Dearnaley, Johann S. de Bono, Carlos Diaz‐Montana, Duncan Gilbert, Silke Gillessen, Clare Gilson, Rob J. Jones, Ruth E. Langley, Zafar I. Malik, David J. Matheson, Robin Millman, Chris C. Parker, Cheryl Pugh, Hannah Rush, J. Martin Russell, Dominik R. Berthold, Michelle L. Buckner, Malcolm D. Mason, Alastair W. S. Ritchie, Alison J. Birtle, Susannah J. Brock, Prantik Das, Dan Ford, Joanna Gale, Warren Grant, Emma K. Gray, Peter Hoskin, Mohammad M. Khan, Caroline Manetta, Neil J. McPhail, Joe M. O'Sullivan, Omi Parikh, Carla Perna, Carmel J. Pezaro, Andrew S. Protheroe, Angus J. Robinson, Sarah M. Rudman, Denise J. Sheehan, Narayanan N. Srihari, Isabel Syndikus, Jacob S. Tanguay, Carys W. Thomas, Salil Vengalil, John Wagstaff, James P. Wylie, Mahesh K. B. Parmar, Matthew R. Sydes, for the STAMPEDE Trials Collaborative Group

**Affiliations:** ^1^ Institute of Cancer Research London UK; ^2^ The Departments of Surgery & Urology The Christie & Salford Royal Hospitals Manchester UK; ^3^ MRC Clinical Trials Unit at UCL, Institute of Clinical Trials & Methodology, UCL London UK; ^4^ The Christie NHS Foundation Trust Manchester UK; ^5^ Salford Royal HS Foundation Trust Manchester UK; ^6^ UCL Cancer Institute, University College London London UK; ^7^ Guy's, King's, & St. Thomas' Hospitals, and Sarah Cannon Research Institute London UK; ^8^ St James University Hospital Leeds UK; ^9^ The Institute of Cancer Research and Royal Marsden NHS Foundation Trust London UK; ^10^ Istituto Oncologico della Svizzera Italiana Bellinzona Switzerland; ^11^ Royal Marsden Hospital London UK; ^12^ Beatson West of Scotland Cancer Centre, University of Glasgow Glasgow UK; ^13^ Radiotherapy Unit The Clatterbridge Cancer Centre NHS Foundation Trust Liverpool Liverpool L7 8YA UK; ^14^ School of Allied Health and Midwifery, Faculty of Education, Health and Wellbeing University of Wolverhampton Wolverhampton WS1 3BD UK; ^15^ (PPI) c/o MRC CTU at UCL London UK; ^16^ Uro‐Oncology Unit Royal Marsden Hospital and Institute of Cancer Research Sutton UK; ^17^ Guys and St Thomas' NHS Foundation Trust London UK; ^18^ Institute of Cancer Sciences, University of Glasgow Glasgow UK; ^19^ Beatson West of Scotland Cancer Centre Glasgow UK; ^20^ Centre Hospitalier Universitaire Vaudois (CHUV) Lausanne Switzerland; ^21^ Cardiff University School of Medicine Cardiff UK; ^22^ Gloucestershire Hospitals NHS Foundation Trust Gloucester UK; ^23^ Rosemere Cancer Centre, Lancashire Teaching Hospitals & University of Manchester, University of Central Lancashire Lancashire UK; ^24^ University Hospital Dorset Bournemouth UK; ^25^ Department of Oncology University Hospitals of Derby and Burton NHS Foundation Trust Derby UK; ^26^ City Hospital, Cancer Centre at Queen Elizabeth Hospital Birmingham UK; ^27^ Portsmouth Hospitals University Trust Portsmouth UK; ^28^ Gloucestershire Oncology Centre, Cheltenham General Hospital Cheltenham UK; ^29^ Musgrove Park Hospital Taunton UK; ^30^ Mount Vernon Cancer Centre Northwood UK; ^31^ Department of Oncology Castle Hill Hospital Hull UK; ^32^ Scarborough General Hospital Scarborough UK; ^33^ Brighton and Sussex University Hospitals NHS Trust Brighton UK; ^34^ Department of Clinical Oncology Raigmore Hospital Inverness UK; ^35^ Patrick G Johnston Centre for Cancer Research, Queen's University Belfast Belfast UK; ^36^ Rosemere Cancer Centre, Lancashire Teaching Hospitals NHS Trust Preston UK; ^37^ Royal Surrey NHS Foundation Trust Guildford UK; ^38^ Sheffield Teaching Hospitals NHS Foundation Trust Sheffield UK; ^39^ Oxford University Hospitals NHS Foundation Trust Oxfordshire UK; ^40^ Sussex Cancer Centre Brighton UK; ^41^ Royal Devon & Exeter NHS Foundation Trust Exeter UK; ^42^ Shrewsbury & Telford Hospitals NHS Trust Shrewsbury UK; ^43^ Velindre Cancer Centre Cardiff UK; ^44^ Kent Oncology Centre Maidstone Kent UK; ^45^ University Hospital North Midlands NHS Trust Staffordshire UK; ^46^ Swansea University and the South West UK Cancer Centre Swansea UK

**Keywords:** abiraterone, clinical trial, hormone therapy, phase III, prostate cancer, randomised controlled trial, survival

## Abstract

Abiraterone acetate plus prednisolone (AAP) previously demonstrated improved survival in STAMPEDE, a multiarm, multistage platform trial in men starting long‐term hormone therapy for prostate cancer. This long‐term analysis in metastatic patients was planned for 3 years after the first results. Standard‐of‐care (SOC) was androgen deprivation therapy. The comparison randomised patients 1:1 to SOC‐alone with or without daily abiraterone acetate 1000 mg + prednisolone 5 mg (SOC + AAP), continued until disease progression. The primary outcome measure was overall survival. Metastatic disease risk group was classified retrospectively using baseline CT and bone scans by central radiological review and pathology reports. Analyses used Cox proportional hazards and flexible parametric models, accounting for baseline stratification factors. One thousand and three patients were contemporaneously randomised (November 2011 to January 2014): median age 67 years; 94% newly‐diagnosed; metastatic disease risk group: 48% high, 44% low, 8% unassessable; median PSA 97 ng/mL. At 6.1 years median follow‐up, 329 SOC‐alone deaths (118 low‐risk, 178 high‐risk) and 244 SOC + AAP deaths (75 low‐risk, 145 high‐risk) were reported. Adjusted HR = 0.60 (95% CI: 0.50‐0.71; *P* = 0.31 × 10^−9^) favoured SOC + AAP, with 5‐years survival improved from 41% SOC‐alone to 60% SOC + AAP. This was similar in low‐risk (HR = 0.55; 95% CI: 0.41‐0.76) and high‐risk (HR = 0.54; 95% CI: 0.43‐0.69) patients. Median and current maximum time on SOC + AAP was 2.4 and 8.1 years. Toxicity at 4 years postrandomisation was similar, with 16% patients in each group reporting grade 3 or higher toxicity. A sustained and substantial improvement in overall survival of all metastatic prostate cancer patients was achieved with SOC + abiraterone acetate + prednisolone, irrespective of metastatic disease risk group.

## Graphical abstract

What's new?

Initial results from the STAMPEDE trial for advanced prostate cancer showed that adding abiraterone acetate and prednisone (AAP) to androgen deprivation therapy improved progression‐free survival. Here, the authors present long‐term results of metastatic patients in the STAMPEDE trial. Median follow‐up increased from 52 to 73 months. They found that AAP improved overall survival of all metastatic prostate cancer patients, whether their disease was high‐risk or low‐risk.

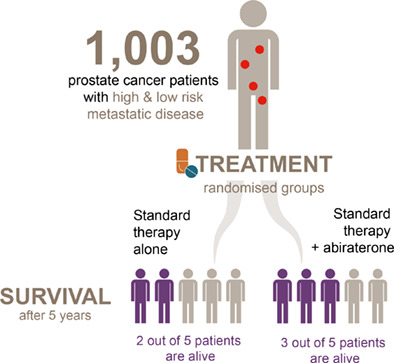

**Abbreviations**
AA: abiraterone acetate
AAP: abiraterone acetate + prednisone/prednisolone
ADT: androgen deprivation therapy
BP: blood pressure
CI: confidence interval
CRUK: Cancer Research UK
CT: computerised tomography (as in CT scan)
CTCAE: Common Terminology Criteria for Adverse Events
CTU: clinical trials unit
FFS: failure‐free survival
GnRH: gonadotrophin‐releasing hormone
HR: hazard ratio
IQR: interquartile range
mg: milligrams
MRC: Medical Research Council
MRI: magnetic resonance imaging
NHS: National Health Service
NIHR: National Institute of Health Research
NSAID: nonsteroidal antiinflammatory drug
PSA: prostate‐specific antigen
QOL: quality of life
SAKK: Swiss Group for Clinical Cancer Research
SOC: standard‐of‐care
SOC + AAP: standard‐of‐care plus abiraterone acetate + prednisone/prednisolone
UCL: University College London
UK: United Kingdom
WHO: World Health Organisation

## 1 INTRODUCTION

Intensifying Androgen Deprivation Therapy (ADT) with abiraterone, enzalutamide or apalutamide is effective for metastatic prostate cancer.^1^, ^2^, ^3^, ^4^, ^5^, ^6^, ^7^ The LATITUDE trial defined metastatic disease risk groups and recruited only patients from a predefined ‘high‐risk’ group. That trial has reported a sustained improvement in survival after a median of 52 months. The primary analysis of the STAMPEDE ‘abiraterone comparison’ was presented in 2017 and reported clinically meaningful and statistically significant improvements in overall and progression‐free survival for adding abiraterone acetate with prednisolone to life‐long ADT compared to life‐long ADT alone.^6^ A long‐term analysis was planned for 3 years after the primary analysis. In 2019, the STAMPEDE Trial Steering Committee, which includes members independent of the Trial Management Group, agreed that future analyses should present results separately for metastatic (M1) and nonmetastatic (M0) patients.

We present here the long‐term results of metastatic patients in the STAMPEDE ‘abiraterone comparison’ with an increase in median follow‐up to 73 months and an increase >50% in the number of deaths. This analysis also incorporates the separation of cases by metastatic disease risk group, classified retrospectively, using the system adopted in the LATITUDE trial.^4^, ^5^ The extended follow‐up from our previous article^8^ with additional events is of particular importance in clarifying treatment effects for patients with low‐risk disease since they were excluded from the LATITUDE trial.^4^, ^5^

## 2 METHODS

The patients, design, treatment and analytic approach have been described in detail previously^6^ and are summarised here.

### 2.1 Study participants

For this comparison in STAMPEDE, eligible patients had metastatic prostate cancer that was newly‐diagnosed or relapsing after previous local therapy and were initiating long‐term androgen deprivation therapy (ADT) which had started no longer than 12 weeks prior to randomisation. There were no age restrictions, but patients were required to have no clinically significant cardiovascular history. For this analysis, patients had metastatic disease confirmed by scintigraphic bone scan and cross‐sectional soft tissue imaging performed within 12 weeks of starting ADT.

### 2.2 Randomisation and masking

Patients were randomised centrally using a computerised algorithm, developed and maintained by the MRC Clinical Trials Unit at UCL. Minimisation with a random element of 20% was used, stratifying for hospital, age at randomisation (<70 vs ≥70 years), nodal involvement (negative vs positive vs indeterminate), WHO performance status (0 vs 1 or 2), planned SOC therapy, and regular aspirin or NSAID use (yes or no). Allocation was 1:1 to standard‐of‐care (SOC‐alone) only group or SOC‐alone with abiraterone acetate and prednisolone/prednisone group (SOC + AAP). There was no blinding to treatment allocation for practical reasons and the key efficacy outcome measures were objective.

### 2.3 Procedures

All patients received lifelong ADT using gonadotrophin‐releasing hormone (GnRH) agonists, antagonists, or orchidectomy. Patients allocated to the SOC + AAP group were also planned to receive abiraterone (1000 mg daily) with prednisolone (5 mg daily). Treatment continued until progression that usually included PSA but also required radiologic or clinical progression, or initiation of second‐line therapy. Dose modifications were described in the protocol.

Patients were followed‐up 6‐weekly until 6 months after randomisation, 12‐weekly to 2 years, 6‐monthly to 5 years and then annually. PSA was measured at every follow‐up visit; further tests were at the clinician's discretion. Nadir PSA was the lowest PSA reported within 24 weeks after randomisation. Regular safety monitoring was required as per the abiraterone product characteristics recommendations. Toxicities and symptoms were reported at regular follow‐up visits, if associated with a change in treatment or when an adverse event was categorised as ‘serious’. These were graded with Common Terminology Criteria (CTCAE) v3·0 until Feb‐2015, v4.0 subsequently. Limited data were collected on long‐term toxicity.

Metastatic disease risk group at randomisation was evaluated through whole body scintigraphy and CT or MRI staging scans. Bone scans were centralised and reviewed by two co‐authors (AH and AA) with 10% independent review by a consultant uro‐radiologist. Visceral metastases and Gleason score were recorded prior to randomisation. Gleason score was reported locally by a clinically qualified pathologist. The metastatic disease risk group was classified according to the definition used in the LATITUDE trial,^1^, ^2^ with high‐risk disease defined as at least two of: ≥3 bone metastases; visceral metastases; Gleason score ≥8.

### 2.4 Outcome measures and statistical analysis

The primary outcome for this comparison was overall survival, with secondary outcomes of failure‐free survival, progression‐free survival, metastatic progression‐free survival, skeletal‐related events, disease‐specific survival, toxicity and therapy for progression.

All analyses were by intention‐to‐treat. For time‐to‐event outcomes, the stratified log‐rank test was used to test for differences between groups. Estimates of effect were obtained from stratified Cox regression models, with Kaplan‐Meier plots presented in KMunicate format.^9^ The Grambsch‐Therneau test was used to check the proportional hazards assumption, with restricted mean survival times from a flexible parametric model taking precedence in the presence of nonproportional hazards (shown where needed). Statistical significance was two‐sided, taken as a *P*‐value of .05, with no formal adjustment for interim analyses since this was preconsidered in the design. Differences in categorical variables were analysed using the *χ* ^2^ test. The prevalence of adverse effects at 2 and 4 years after randomisation are presented for the solicited categories. Further drug treatment at any time after primary treatment failure is also presented.

A sensitivity analysis was undertaken to exclude patients who did not meet the strictest interpretation of all the protocol eligibility criteria, which primarily related to baseline blood pressure. The eligibility criteria excluded patients with uncontrolled hypertension. Patients reported as being fit for the trial, with no signs of uncontrolled hypertension or other severe cardiovascular history, but whose single baseline blood pressure (BP) reading was out‐of‐range were conservatively excluded in sensitivity analysis.

## 3 RESULTS

**FIGURE 1:**
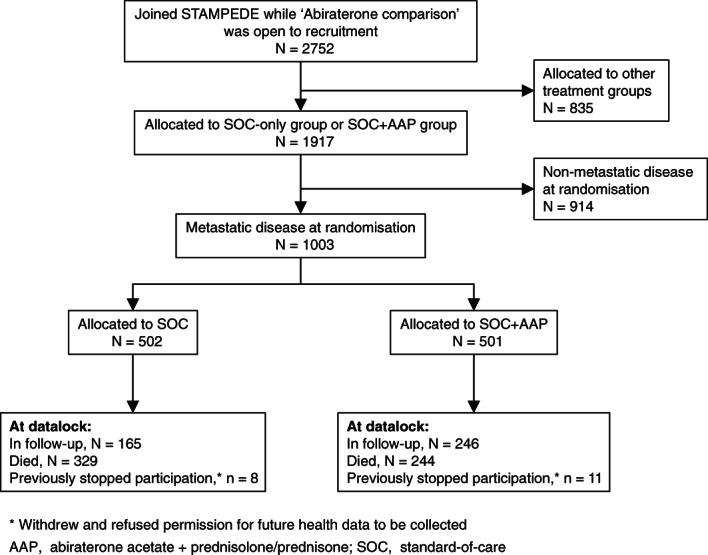
CONSORT diagram

Between 15 November 2011 and 17 January 2014, 1917 patients were randomised to the arms of STAMPEDE constituting the ‘abiraterone comparison’. Of these, all 1003 patients with metastatic disease were analysed here: 502 (50%) allocated to standard treatment (SOC‐alone group) and 501 (50%) to standard treatment plus abiraterone and prednisolone (SOC + AAP group) (Figure 1). Median age at randomisation was 67 years (IQR 62‐71), 941 (94%) had newly diagnosed disease (Table 1). Metastatic disease risk group was retrospectively classified as low‐risk in 436 (43%) patients, high in 473 (47%) and was unclassified in a further 94 (9%). Bone metastases were detected in 882 (88%) patients and distant lymph node metastases in 293 (29%). All baseline disease characteristics were balanced between randomised groups.

**TABLE 1:** Baseline characteristics

	SOC‐alone	SOC ± AAP	All
	n = 502	n = 501	n = 1003
Age (years)			
Median (IQR)	67 (62‐72)	67 (62‐71)	67 (62‐71)
Range	39‐84	42‐85	39‐85
Eligibility category			
Newly diagnosed	475 (95%)	466 (93%)	941 (94%)
Relapsing	27 (5%)	35 (7%)	62 (6%)
PSA (ng/mL)			
Median (IQR)	97.2 (26.0‐358)	96.3 (29‐371)	96.9 (27.3‐363)
Range	0.6‐10 530	0.1‐21 460	0.1–21 460
Metastatic disease risk group			
Low‐risk	220 (44%)	208 (42%)	428 (43%)
High‐risk	232 (46%)	241 (48%)	473 (47%)
Unclassified	50 (10%)	52 (10%)	102^a^ (10%)
Site of metastases^b^			
Bone	448 (89%)	434 (87%)	882 (88%)
Liver	8 (2%)	7 (1%)	15 (2%)
Lung	21 (4%)	21 (4%)	42 (4%)
Distant lymph nodes	150 (30%)	143 (29%)	293 (29%)
Other	26 (5%)	23 (5%)	49 (5%)

Abbreviations: AAP, abiraterone acetate + prednisolone/prednisone; IQR, interquartile range; SOC, standard‐of‐care.

^a^ Includes 14 patients at Swiss sites for whom imaging could not be obtained.

^b^ Patients can be in multiple categories.

The database was locked for this analysis on 3 April 2020. Median follow‐up was 73 months (6.1 years). Median time on abiraterone in the SOC + AAP group was 29 months (IQR 12‐71) and 126 (25%) participants were still on their trial supplies of abiraterone at the data freeze.

**FIGURE 2:**
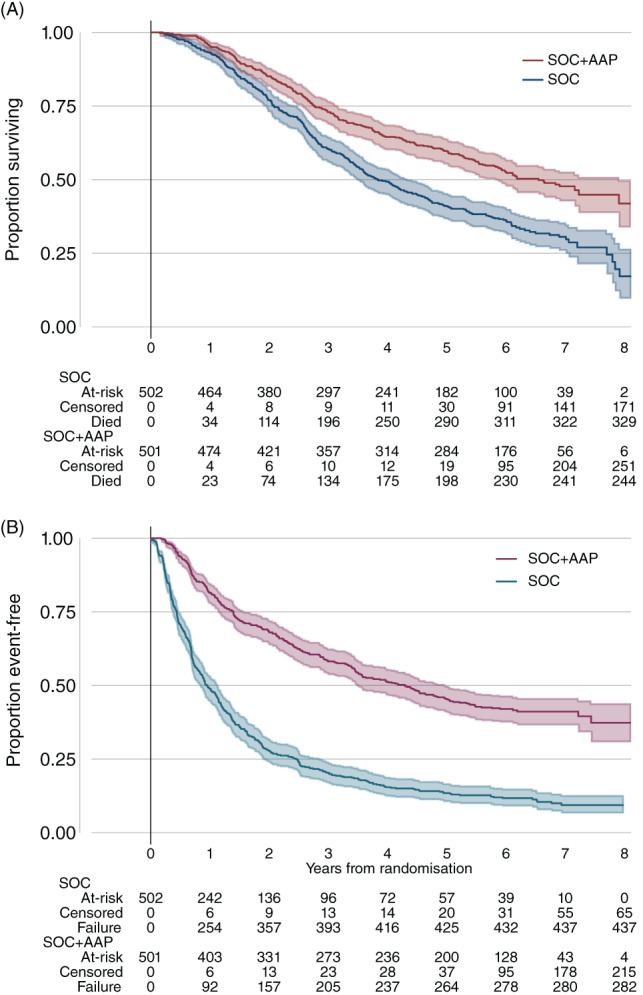
Overall survival by allocated treatment: 2A ‐ Overall survival by allocated treatment; 2B ‐ Failure‐free survival by allocated treatment [Color figure can be viewed at wileyonlinelibrary.com]

Deaths were reported in 573/1003 (57%) participants including 329 (66%) in the SOC‐alone group and 244 (49%) in the SOC + AAP group: HR = 0.60 for SOC + AAP (95% CI: 0.50‐0.71, *P* < .0001) (Table 2, Figure 2A). There was no evidence of nonproportional hazards in the treatment effect (*P* = .78). Median survival was 46 months (IQR 25, 92) in the SOC‐alone group and 79 months (IQR 33, not reached) in the SOC + AAP group; 5‐year survival was 41% (95% CI: 37%‐45%) for SOC‐alone and 60% (95% CI: 50%‐71%) for SOC + AAP. The sensitivity analyses excluding 157 patients (16%) did not change the primary outcome measure results HR = 0.62 (95% CI: 0.52‐0.75; *P* = 0.14 × 10^−6^).

**TABLE 2:** Primary and secondary outcome measure

	SOC‐alone	SOC ± AAP	
	n = 502	n = 501	
Overall survival			
Events	329	244	
% alive at 5 years (95% CI)	41% (37‐45)	60% (55‐64)	
HR = vs SOC‐alone (95% CI), *P*	(Reference)	0.60 (0.50‐0.71)	<.0001
RMST (months), *P* (proportional hazards)	54 (51‐57)	66 (63‐69)	.78
Failure‐free survival			
Events	437	282	
% event‐free at 5 years (95% CI)	13% (11‐17)	45% (41‐50)	
HR = vs SOC‐alone (95% CI), *P*	(Reference)	0.34 (0.29‐0.40)	<.0001
RMST (months), *P* (proportional hazards)	24 (21‐27)	55 (51‐59)	.0001
Progression‐free survival			
Events	323	241	
% event‐free at 5 years (95% CI)	37% (33‐42)	54% (50‐59)	
HR = vs SOC‐alone (95% CI), *P*	(Reference)	0.58	<.0001
RMST (months), *P* (proportional hazards)	47 (43‐51)	62 (59‐66)	.038
Metastatic PFS			
Events	309	230	
% event‐free at 5 years (95% CI)	40% (36‐45)	56% (52‐61)	
HR = vs SOC‐alone (95% CI), *P*	(Reference)	0.60 (0.50‐0.71)	<.0001
RMST (months), *P* (proportional hazards)	50 (46‐53)	64 (60‐67)	.13
Skeletal‐related events			
Events	100	76	
% event‐free at 5 years (95% CI)	76% (71‐80)	82% (78‐86)	
HR = vs SOC‐alone (95% CI), *P*	(Reference)	0.56 (0.41‐0.76)	.0008
RMST (months), *P* (proportional hazards)	78 (74‐81)	84 (82‐87)	.33
Disease‐specific survival			
Events	255	156	
% event‐free at 5 years (95% CI)	50% (45‐55)	72% (67‐76)	
HR = vs SOC‐alone (95% CI), *P*	(Reference)	0.49 (0.39‐0.60)	<.0001
RMST (months), *P* (proportional hazards)	60 (57‐64)	75 (72‐78)	.97

Abbreviations: 95% CI, 95% confidence interval; AAP, abiraterone acetate + prednisolone/prednisone; *P*, *P*‐value; Reference, reference arm; RMST, restricted mean survival time; SOC, standard‐of‐care.

Failure‐free survival (FFS) events were reported for 437 (87%) in the SOC‐alone group and 282 (56%) in the SOC + AAP group, HR = 0.34 for SOC + AAP (95% CI: 0.29‐0.40, *P* < .0001) (Table 2, Figure 2B). A statistically significant benefit of treatment with SOC + AAP, compared to SOC‐alone, was observed in all other secondary outcomes (Table 2, Figures S1 and S2): progression‐free survival (HR = 0.58, 95% CI: 0.49‐0.69, *P* < .0001), metastatic progression‐free survival (HR = 0.60, 95% CI: 0.50‐0.71, *P* < .0001), skeletal‐related events (HR = 0.56, 95% CI: 0.41‐0.76, *P* = .0008) and disease‐specific survival (HR = 0.49, 95% CI: 0.39‐0.60, *P* < .0001).

**FIGURE 3:**
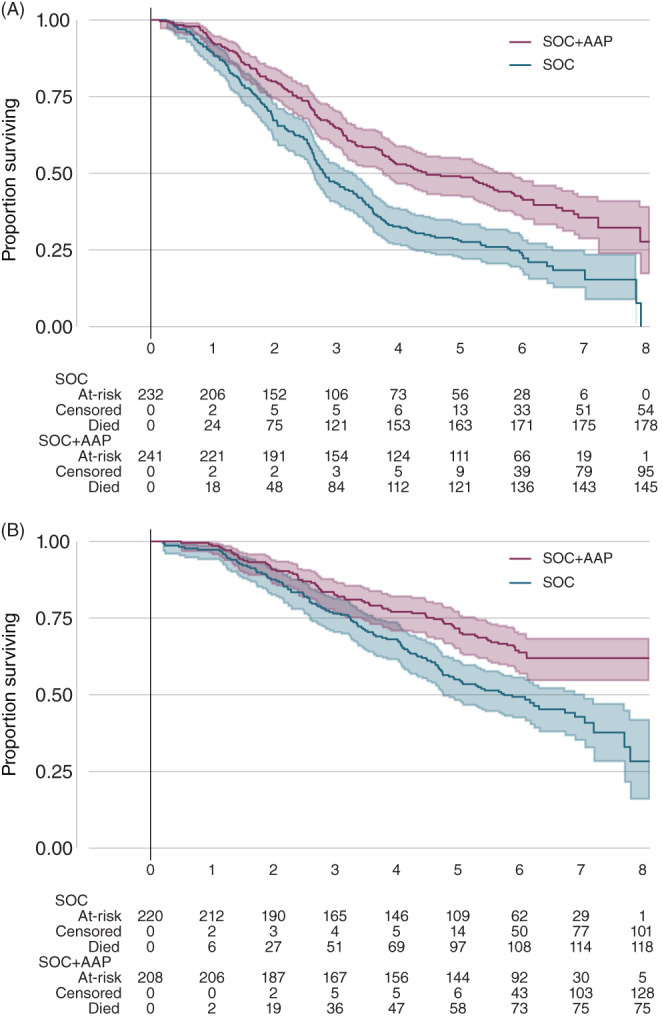
Overall survival by allocated treatment and metastatic disease risk group: 3A ‐ high‐risk metastatic disease risk group; 3B ‐ low‐risk metastatic disease risk group [Color figure can be viewed at wileyonlinelibrary.com]

Focusing on the 91% (909/1003) of patients for whom metastatic disease risk group could be calculated, the relative effect of SOC + AAP on overall survival was similar in both low‐risk and high‐risk metastatic disease risk groups (low‐risk HR = 0.54, 95% CI: 0.40‐0.74; high‐risk HR = 0.54, 95% CI: 0.43‐0.69, respectively) (Table 3, Figure 3) [Correction added on 6 June 2022, after first online publication: The HR and the 95% CI values have been changed from 0.55 to 0.54 and 0.41‐0.76 to 0.40‐0.74, respectively.]. The effect of SOC + AAP was also observed to be similar in both low‐risk and high‐risk metastatic disease risk groups for the secondary outcomes of failure‐free survival, progression‐free survival, metastatic progression‐free survival, skeletal‐related events and disease‐specific survival.

**TABLE 3:** Primary and Secondary outcomes, by metastatic disease risk group using LATITUDE criteria

	Metastatic disease risk group					
	Low‐risk		High‐risk		Unclassified^a^	
	SOC‐alone	SOC ± AAP	SOC‐alone	SOC ± AAP	SOC‐alone	SOC ± AAP
	n = 220	n = 208	n = 232	n = 241	n = 45	n = 43
Overall survival						
Events	118	75	178	145	29	22
% alive at 5 years, (95% CI)	55% (48‐61)	72% (65‐77)	28% (22‐34)	49% (43‐55)	40% (26‐54)	59% (42‐72)
HR = vs SOC‐alone (95% CI), *P*	0.54 (0.40‐0.74)	<.0001	0.54 (0.43‐0.69)	<.0001	0.63 (0.33‐1.23)	.180
Failure‐free survival						
Events	178	92	215	165	40	22
% event‐free at 5 years, (95% CI)	21% (16‐26)	61% (54‐67)	6% (3‐9)	31% (25‐37)	18% (8‐30)	44% (29‐59)
HR = vs SOC‐alone (95% CI), *P*	0.32 (0.25‐0.42)	<.0001	0.28 (0.22‐0.36)	<.0001	0.33 (0.17‐0.64)	0002
Progression‐free survival						
Events	118	73	169	146	32	19
% event‐free at 5 years, (95% CI)	50% (43‐57)	70% (63‐76)	25% (19‐31)	39% (33‐46)	36% (22‐49)	59% (42‐73)
HR = vs SOC‐alone (95% CI), *P*	0.55 (0.40‐0.75)	<.0001	0.56 (0.46‐0.72)	<.0001	0.38 (0.19‐0.75)	.007
Metastatic PFS						
Events	113	66	164	142	30	19
% event‐free at 5 years, (95% CI)	52% (45‐59)	73% (66‐79)	28% (22‐34)	41% (34‐47)	39% (25‐53)	59% (42‐73)
HR = vs SOC‐alone (95% CI), *P*	0.52 (0.37‐0.72)	<.0001	0.59 (0.47‐0.75)	<.0001	0.44 (0.22‐0.88)	.009
Skeletal‐related events						
Events	35	24	55	44	9	8
% event‐free at 5 years, (95% CI)	84% (78‐89)	88% (82‐92)	65% (56‐73)	78% (71‐83)	77% (57‐88)	75% (56‐87)
HR = vs SOC‐alone (95% CI), *P*	0.47 (0.27‐0.83)	.010	0.51 (0.33‐0.79)	.008	0.83 (0.29‐2.39)	.82
Disease‐specific survival						
Events	90	39	138	100	25	15
% event‐free at 5 years, (95% CI)	64% (57‐70)	86% (80‐90)	37% (30‐44)	60% (53‐66)	46% (31‐60)	72% (54‐84)
HR = vs SOC‐alone (95% CI), *P*	0.36 (0.24‐0.54)	<.0001	0.49 (0.37‐0.65)	<.0001	0.48 (0.22‐1.04)	.050

^a^ Scans were unavailable for patients at sites in Switzerland, 14 further patients therefore do not appear in this table.

Abbreviations: 95% CI, 95% confidence interval; AAP, abiraterone acetate + prednisolone/prednisone; *P*, *P*‐value; Ref, reference arm; RMST, restricted mean survival time; SOC, standard‐of‐care.

Further treatment was reported for most patients within 1 year of first disease progression (Table S1, Figure S3). Patients allocated to the SOC‐alone group were more likely to receive abiraterone or enzalutamide within 1 year (abiraterone, 19% vs 2%, Chi‐square *P* < .0001; enzalutamide, 16% vs 8%, Chi‐square *P* = .002). Reported use of docetaxel within 1 year after first progression was higher among patients allocated to SOC + AAP (32% SOC‐alone and 40% SOC + AAP, Chi‐square *P* = .048).

Adverse event data was reported at 2 years after randomisation for 136 patients in the SOC‐alone group whose disease had not already progressed and 291 patients in the SOC + AAP group who were still on treatment. Of these, data was received from 133 (98%) SOC‐alone and 286 (98%) SOC + AAP patients, respectively. The worst reported grade of toxicity was similar between randomised groups (*P* = .29, Table S2) with grade 3 toxicity for 12 (9%) in the SOC‐alone group patients and 20 (7%) in the SOC + AAP group, and no grade 4 or 5 toxicity. Four years after randomisation, the worst grade of toxicity reported was again similar between randomised groups (*P* = .56).

## 4 DISCUSSION

This updated follow‐up of the STAMPEDE ‘abiraterone comparison’ demonstrated that the effects reported previously^6^ were robust. With 57% participants now deceased and a median follow‐up of more than 6 years, this represented a considerable increase in information over the previous report (Table S3): these results are unlikely to change meaningfully with any further follow‐up.

There was no evidence of difference in effect size when the patients were separated by metastatic disease risk group using the system defined by researchers for the LATITUDE trial.^4^, ^5^ This is important as, in many regions, both the licenced indication and reimbursement for the drug are restricted to the high‐risk group defined by the eligibility criteria for the LATITUDE trial. We have shown previously that the ‘low‐risk’ metastatic disease risk group constitutes >40% of patients presenting with metastatic prostate cancer.^6^, ^10^ The overall effect of abiraterone may be underestimated as the effect size was larger in both the low‐risk and high‐risk groups, indicating potential confounding effect of risk on the association between treatment and survival. We also carried out additional analyses using the definition of metastatic disease risk group employed in the CHAARTED trial.^11^ Our previous analysis showed that the two systems for defining metastatic disease risk group largely coincided but that 18% (164/901) of patients were low‐risk on one system and high‐risk on the other (or vice versa). There was no evidence that the classifier used affected the overall conclusion with respect to the impact of disease burden on treatment effects.^8^ The classification was done with scans taken before randomisation but collected afterwards. The minority of patients for whom suitable imaging was unavailable are presented separately. This group will have had either missing scans or been staged using techniques such as PSMA‐PET or whole‐body MRI and thus are not directly classifiable using the separate systems for LATITUDE and CHAARTED. Our results strongly support the option for use of abiraterone for all patients starting long‐term hormone therapy for metastatic prostate cancer, irrespective of metastatic disease risk group.

Table 4 shows the findings of STAMPEDE alongside the results from LATITUDE. Combining the aggregate results using standard meta‐analysis methods further clarifies the survival advantage for SOC + AAP over SOC; this extended to the wider population of patients with metastatic prostate cancer, not just those in the high‐risk metastatic disease risk group defined for LATITUDE. Our long‐term results are strikingly similar to those observed with both apalutamide^1^, ^2^ and enzalutamide^3^ in trials with similar eligibility criteria to the metastatic population into STAMPEDE. While those agents are androgen receptor antagonists, all three drugs work by targeting the androgen receptor axis. This suggests that a choice of any of these agents may be clinically reasonable and should be driven by secondary considerations such as side‐effect profiles or cost rather than by primary efficacy or disease risk/metastatic burden. No additional toxicity data has been collected since the primary report in 2017, so we have not updated those aspects here. No further treatment for relapse had been reported for 29 of the 329 patients on the control arm who had died. Planned future access to national healthcare system data may facilitate reporting of additional long‐term adverse events, such as late effects on cardiovascular effects, skeletal events and the need for additional systemic therapies.

**TABLE 4:** Combined analyses from STAMPEDE and LATITUDE

Trial & population	Published	Pts	Control	Research	Hazard ratio (95% CI)
LATITUDE					
M1, High‐risk	*First results: 2017* ^*4*^	*1199*	*232*/*602*	*169*/*597*	*0*.*62* (*0*.*51*‐*0*.*76*)
	Updated results: 2019^5^	1199	305/602	230/597	0.66 (0.56‐0.78)
STAMPEDE ‘abiraterone comparison’					
All: M0 and M1, any risk	*First results: 2017* ^*6*^	*1917*	*262*/*957*	*184*/*960*	*0*.*63* (*0*.*52*‐*0*.*76*)
Subset: M1, any risk	*First results: 2017* ^*6*^	*1002*	*218*/*502*	*150*/*501*	*0*.*61* (*0*.*49*‐*0*.*75*)
	Updated results (here)	1002	329/502	244/ 501	0.60 (0.50‐0.71)
Subset: M1, high‐risk	*First results: 2019* ^8^, ^a^	*473*	*136*/*232*	*94*/*241*	*0*.*54* (*0*.*41*‐*0*.*70*)
	Updated results (here)	473	178/232	145/241	0.54 (0.43‐0.69)
Combined					
Metastatic, any risk			634/1104	182/1097	0.63 (0.50‐0.71)
Metastatic, high‐risk			483/843	182/829	0.62 (0.54‐0.71)

*Note*: Risks defined using the metastatic disease risk group system used for LATITUDE.

^a^ Same data freeze as 2017 paper.

Most patients in STAMPEDE had de novo metastatic disease, a higher proportion than most other trials in this setting and few patients had visceral metastatic disease at entry. Therefore, these trial data could not be used to explore whether there is a differential treatment effect by these characteristics.

The widely‐accessible, alternative standard‐of‐care for men with hormone‐sensitive metastatic prostate cancer is docetaxel.^11^, ^12^ Controversy exists as to whether metastatic disease risk group predicts the effectiveness of the agent. Previous data from STAMPEDE supports the use of docetaxel as an alternative to androgen receptor targeting in all newly‐diagnosed groups irrespective of metastatic disease risk group.^10^, ^13^ Direct comparison of patient‐related QoL outcomes previously supported the use of abiraterone over docetaxel,^14^ however, costs of abiraterone are currently higher than docetaxel and hence reimbursement varies in different countries. As the abiraterone patent will be expired in most territories in the coming years, these costs can be expected to fall.

We have previously reported the effects of prostate radiotherapy for hormone‐sensitive metastatic prostate cancer from another comparison in STAMPEDE.^15^ Patients in that comparison did not receive upfront abiraterone hence we do not currently know the effect of the interaction between these two possible upfront therapies. The forthcoming data from the PEACE‐1 trial, recently presented at ESMO, reports a failure free and overall survival advantage from the triplet compared to the ADT‐abiraterone doublet. Further data are awaited from the ENZAMET and ARASENS trials on the same question. No trial has addressed the reverse question (should docetaxel be added to abiraterone). There will be quality of life plus relative fitness for docetaxel vs abiraterone issues that will likely limit uptake of the triplet therapy. Long‐term follow‐up of the complementary cohort of nonmetastatic patients from this ‘abiraterone comparison’ in STAMPEDE were analysed alongside first results from the trial ‘enzalutamide + abiraterone comparison’ and show compelling evidence of improved metastases‐free survival and overall survival with abiraterone‐based therapy. Those nonmetastatic patients did receive radiotherapy in the majority of cases, unless there was a clinical contraindication.^16^

In conclusion, this extended analysis further reinforces the body of data on the substantial benefits of upfront targeting of the androgen receptor pathway using abiraterone acetate in all men with hormone‐sensitive metastatic prostate cancer, irrespective of metastatic disease risk group.

## AUTHOR CONTRIBUTION

Nicholas D James was the Chief Investigator. MP developed the MAMS concept. Nicholas D James was the Comparison Chief Investigator. Mahesh KB Parmar, Nicholas D James, Matthew R Sydes, Ruth E Langley, Noel W Clarke, Malcolm D Mason and David P Dearnaley designed the trial. David P Dearnaley, Nicholas D James, Malcolm D Mason, Mahesh KB Parmar, Matthew R Sydes and Noel W Clarke were Grant holders (UK). Nicholas D James, Noel W Clarke, Adrian Cook, Gerhardt Attard, Christopher D Brawley, Simon Chowdhury, William R Cross, David P Dearnaley, Johann S de Bono, Duncan Gilbert, Silke Gillessen, Clare Gilson, Rob J Jones, Ruth E Langley, Zafar I Malik, David J Matheson, Robin Millman, Chris C Parker, Cheryl Pugh, Hannah Rush, J Martin Russell, Michelle L Buckner, Malcolm D Mason, Alastair WS Ritchie, Mahesh KB Parmar and Matthew R Sydes were members of the Trial Management Group. CA, Cheryl Pugh and MB were part of trial operations. All authors collated data. Adrian Cook, NJ, Noel W Clarke, Adnan Ali, Mahesh KB Parmar, and Matthew R Sydes wrote the Statistical Analysis Plan. Adrian Cook, CB and Matthew R Sydes performed the analyses. All authors interpreted the data. Nicholas D James, Adrian Cook, Noel W Clarke and Matthew R Sydes wrote critical sections of the article. All authors reviewed, edited and approved the final article. The work reported in the article has been performed by the authors, unless clearly specified in the text.

## CONFLICT OF INTEREST

Gerhard Attard received personal fees from Sanofi Aventis, Astellas, Medivation, Novartis, Millennium Pharmaceuticals, Abbott Laboratories, Essa Pharmaceuticals, Bayer Healthcare Pharmaceuticals, Takeda, Janssen, Veridex, Roche/Ventana, Pfizer, the Institute of Cancer Research (ICR); grants from Astra Zeneca, Arno therapeutics, Innocrin Pharma, Janssen; and Royalty income from Institute of Cancer Research abiraterone, share of income through ICR's Rewards to discoverers scheme.

Alison J. Birtle served on Advisory Boards for Astellas, Bayer, Janssen, Roche, MSD, Merck, Pfizer, BMS, Astra Zeneca and has speaker fees and travel support from Bayer, Janssen, Sanofi, Astellas. Simon Chowdhury received speaker fees and/or article writing and/or educational events from Astra Zeneca, Novartis/AAA, Clovis Oncology, Janssen, Bayer, Pfizer, Beigene & Astellas; is an advisory board member of Astellas, Janssen, Novartis/AAA, Bayer, Astellas, Athenex, Beigene, Clovis Oncology. He received consulting fees from Telix, Remedy Bio, Huma; research support from Clovis Oncology; and meeting/travel expenses from Janssen, Beigene; he is the founder of Curve. life and earns stock of Curve. life, Huma, Remedy Bio. Noel W Clarke received honoraria from Astellas & Janssen; took a consulting/advisory role for Astellas, Janssen, Ferring, Bayer & Sanofi; was paid speakers fees from Janssen & Astellas; received funding for the institution from Astra Zeneca; received meeting and travel expenses from Janssen, Astellas, Sanofi, Astra Zeneca, Ferring & Ipsen. Prof D. Dearnaley is an advisory board member for Janssen Pharma; holds European patent EP1933709B1 (pending in Canada and India) for a Location and Stabilisation Device; and his previous employer. The Institute of Cancer Research, receives loyalty income from abiraterone from which Prof, Dearnaley receives a share of this income through the ICR's Rewards to Discoverer's Scheme. Johann S. de Bono received personal fees from Amgen, Astellas, Astra Zeneca, Bayer, Bioxcel Therapeutics, Boehringer Ingelheim, Cellcentric, Daiichi, Eisai, Gentech Roche, Genmab, GlaxoSmithKline, Harpoon, Janssen, Menarini Silicon Biosystems, Merck Serono, Merck Sharpe & Dome, Orion Pharma, Pfizer, Qiagen, Sanofi Aventis, Sierra Oncology, Taiho, Terumo, Vertex Pharmaceuticals; grants received from Astellas, Bayer, Cellcentric, Daiichi, Genmab, GlaxoSmithKline, Janssen, Merck Serono, Merck Sharpe & Dome, Orion Pharma, Pfizer, Sanofi Aventis, Sierra Oncology, Taiho, Vertex Pharmaceuticals. Other payments received from Amgen, Astellas, Astra Zeneca, Bayer, Bioxcel Therapeutics, Boehringer Ingelheim, Cellcentric, Daiichi, Eisai, Gentech Roche, Genmab, GlaxoSmithKline, Harpoon, Janssen, Menarini Silicon Biosystems, Merck Serono, Merck Sharpe & Dome, Orion Pharma, Pfizer, Qiagen, Sanofi Aventis, Sierra Oncology, Taiho, Terumo, Vertex Pharmaceuticals; in addition, Prof. De Bono has a patent DNA damage repair inhibitors for treatment of cancer (patent no. WO 2005 053662) licenced to Astra Zeneca, and a patent 17‐substituted steroids useful in cancer treatment; patent no. S 5604213) licenced to Janssen. Silke Gillessen is on the advisory board of Menarini Silicon Biosystems, Aranda, Orion, Amgen, Tolero Pharmaceuticals, Astellas, Janssen, Merck Sharpe & Dome, Bayer, Roche, Pfizer, Telix Pharma, Bristol‐Mysers Squibb, AAA International SA, Novartis, Modra Pharmaceuticals and the steering committee of AMGEN; a speaker: Orikata, SAKK, Beijing Family Hospital, ESMO, Swiss Academy of Multidisciplinary Oncology (SAMO); and on the speakers bureau of Janssen; she received travel/meeting expenses from ProteoMedix and for consultancy for S. Grassi Consulting; other payments received from DESO, RSI, Oncoforum. Clare Gilson received research funding to the institution from Janssen, Clovis Oncology, Sanofi, Astellas, Medical Research Council & Cancer Research UK. Dan Ford received speaker fees and/or article writing and/or educational events from BMS, IPSEN, EUSA, Pfizer, ESAI; they received travel expenses from Janssen & IPSEN. Nicholas D James received research funding to the institution from Astellas, Astra Zeneca & Janssen; receipt of honoraria/fees on the advisory board for Astra Zeneca, Clovis Oncology, Janssen, Merck, Novartis & Sanofi; received fees as a speaker for Bayer & Novartis. Ruth E. Langley received an institutional grant from the MRC. Malcolm D. Mason is an advisory board member for Endocyte & Clovis Oncology. Neil J. McPhail received consulting fees from GlaxoSmithKline, Eisai & IPSEN; received meeting attendance expenses from IPSEN and received conference fees from Bayer. Carmel J. Pezaro received honoraria for lectures from AAA, Astra Zeneca, Janssen, they received meeting/travel support from Bayer & IPSEN. Joe M. O'Sullivan received speaker fees from AAA, Astellas, Bayer, Janssen, Novartis, Sanofi and participated as an advisory board member and/or member of the data safety monitoring board for AAA, Astellas, Bayer, Janssen, Novartis & Sanofi. Sarah Rudman has received speakers fees from Janssen. Mahesh K. B. Parmar received research funding to the Unit he directs from Acoria Pvt Ltd, Akagera, Amgen, Aspirin Foundation, Astellas, AstraZeneca, Baxter, Bayer, BMS US, Bri‐Bio, Cepheid, Cipla, Clovis Inc, CSL Behring, Eli‐Lilly, Emergent Biosolutions, Gilead Sciences, GlaxoSmithKline, Grifols, Janssen Products LP, Janssen‐Cilag, Johnson & Johnson, Micronoma, Modus Theraputics, Mylan, Novartis, Pfizer, Sanofi, Serum Institute of India, Shionogi, Synteny Biotechnology, Takeda, Tibotec, Transgene, ViiV Healthcare, Virco and Xenothera. Narayanan N. Srihari received travel/meeting payments from Janssen. Rob J. Jones received research funding to the institution from Bayer, Astellas & Pfizer; received honoraria on the advisory board for Janssen, Astellas, Bayer, Pfizer; received speaker fees from Janssen, Astellas, Bayer & Pfizer. Matthew R. Sydes received research funding to the institution from Astellas, Clovis Oncology, Janssen, Novartis, Pfizer, Sanofi‐Aventis; received speaker fees from Lilly Oncology & Janssen; independent member of data monitoring committees. The other authors do not declare relevant competing interests for this work.

## ETHICS STATEMENT

Appropriate ethical review was in place for each participating country. All participants gave written, informed consent. The trial identification for STAMPEDE is NCT00268476 (clinicaltrials.gov) and ISRCTN78818544 (www.isrctn.com).

## Supporting information


**Appendix S1**Supporting Information.

**Appendix S2**Supporting Information.

## Data Availability

The data that support the findings of our study are available from the corresponding author upon request, and following approval by the MRC CTU at UCL.

## References

[1] Chi KN , Agarwal N , Bjartell A , et al. Apalutamide for metastatic, castration‐sensitive prostate cancer. N Engl J Med. 2019;381:13‐24. 31150574 10.1056/NEJMoa1903307

[2] Chi KN , Chowdhury S , Bjartell A , et al. Apalutamide in patients with metastatic castration‐sensitive prostate cancer: final survival analysis of the randomized, double‐blind, phase III TITAN study. J Clin Oncol. 2021;39:2303. 10.1200/JCO.20.03488 33914595

[3] Davis ID , Martin AJ , Stockler MR , et al. Enzalutamide with standard first‐line therapy in metastatic prostate cancer. N Engl J Med. 2019;381:121‐131. 31157964 10.1056/NEJMoa1903835

[4] Fizazi K , Tran N , Fein L , et al. Abiraterone plus prednisone in metastatic, castration‐sensitive prostate cancer. N Engl J Med. 2017;377:352‐360. 28578607 10.1056/NEJMoa1704174

[5] Fizazi K , Tran N , Fein L , et al. Abiraterone acetate plus prednisone in patients with newly diagnosed high‐risk metastatic castration‐sensitive prostate cancer (LATITUDE): final overall survival analysis of a randomised, double‐blind, phase 3 trial. Lancet Oncol. 2019;20:686‐700. 30987939 10.1016/S1470-2045(19)30082-8

[6] James ND , de Bono JS , Spears MR , et al. Abiraterone for prostate cancer not previously treated with hormone therapy. N Engl J Med. 2017;377:338‐351. 28578639 10.1056/NEJMoa1702900 PMC5533216

[7] Armstrong AJ , Szmulewitz RZ , Petrylak DP , et al. ARCHES: a randomized, phase III study of androgen deprivation therapy with enzalutamide or placebo in men with metastatic hormone‐sensitive prostate cancer. J Clin Oncol. 2019;37:2974‐2986. 31329516 10.1200/JCO.19.00799 PMC6839905

[8] Hoyle AP , Ali A , James ND , et al. Abiraterone in “high‐” and “low‐risk” metastatic hormone‐sensitive prostate cancer. Eur Urol. 2019;76:719‐728. 31447077 10.1016/j.eururo.2019.08.006

[9] Morris TP , Jarvis CI , Cragg W , Phillips PPJ , Choodari‐Oskooei B , Sydes MR . Proposals on Kaplan‐Meier plots in medical research and a survey of stakeholder views: KMunicate. BMJ Open. 2019;9:e030215. 10.1136/bmjopen-2019-030215 PMC6773317 31575572

[10] Clarke NW , Ali A , Ingleby FC , et al. Addition of docetaxel to hormonal therapy in low‐ and high‐burden metastatic hormone sensitive prostate cancer: long‐term survival results from the STAMPEDE trial. Ann Oncol. 2019;30:1992‐2003. 31560068 10.1093/annonc/mdz396 PMC6938598

[11] Sweeney CJ , Chen YH , Carducci M , et al. Chemohormonal therapy in metastatic hormone‐sensitive prostate cancer. N Engl J Med. 2015;373:737‐746. 26244877 10.1056/NEJMoa1503747 PMC4562797

[12] James ND , Sydes MR , Clarke NW , et al. Addition of docetaxel, zoledronic acid, or both to first‐line long‐term hormone therapy in prostate cancer (STAMPEDE): survival results from an adaptive, multiarm, multistage, platform randomised controlled trial. Lancet. 2016;387:1163‐1177. 26719232 10.1016/S0140-6736(15)01037-5 PMC4800035

[13] Sydes MR , Spears MR , Mason MD , et al. Adding abiraterone or docetaxel to long‐term hormone therapy for prostate cancer: directly randomised data from the STAMPEDE multi‐arm, multi‐stage platform protocol. Ann Oncol. 2018;29:1235‐1248. 29529169 10.1093/annonc/mdy072 PMC5961425

[14] Rush HL , Murphy L , Morgans AK , et al. Quality of life for men with prostate cancer contemporaneously randomly allocated to receive either docetaxel or abiraterone in the STAMPEDE trial. J Clin Oncol. 2021;40:825‐836. 34757812 10.1200/JCO.21.00728 PMC7612717

[15] Parker CC , James ND , Brawley CD , et al. Systemic therapy for advanced or metastatic prostate cancer: evaluation of drug efficacy i. radiotherapy to the primary tumour for newly diagnosed, metastatic prostate cancer (STAMPEDE): a randomised controlled phase 3 trial. Lancet. 2018;392:2353‐2366. 30355464 10.1016/S0140-6736(18)32486-3 PMC6269599

[16] Attard G , Murphy L , Clarke NW , et al. Systemic therapy in advancing or metastatic prostate cancer: evaluation of drug efficacy i. Abiraterone acetate and prednisolone with or without enzalutamide for high‐risk non‐metastatic prostate cancer: a meta‐analysis of primary results from two randomised controlled phase 3 trials of the STAMPEDE platform protocol. Lancet. 2022;399:447‐460. 34953525 10.1016/S0140-6736(21)02437-5 PMC8811484

