# Supplementary material for: Abiraterone acetate plus prednisolone for metastatic patients starting hormone therapy: 5‐year follow‐up results from the STAMPEDE randomised trial (NCT00268476)
Source: Int J Cancer. 2022 May 16;151(3):422–34. doi: 10.1002/ijc.34018 (PMC9321995; doi:10.1002/ijc.34018)
Supplement: Supplementary file 1 — Appendix S1Supporting Information. [file IJC-151-422-s002.pdf]

Supplementary Materials for:

Abiraterone acetate plus prednisolone for metastatic patients starting hormone therapy: 5-year follow-up results from the STAMPEDE randomised trial (NCT00268476)

Authors: Nicholas D James, Noel W Clarke, Adrian Cook, Adnan Ali, Alex P Hoyle, Gert Attard, Chris D Brawley, Simon Chowdhury, William R Cross, David P Dearnaley, Johann S de Bono, Carlos Diaz Montana, Duncan Gilbert, Silke Gillessen, Clare Gilson, Rob J Jones, Ruth E Langley, Zafar I Malik, David J Matheson, Robin Millman, Chris C Parker, Cheryl Pugh, Hannah Rush, J Martin Russell, Dominic R Berthold, Michelle L Buckner, Malcolm D Mason, Alastair WS Ritchie, Alison J Birtle, Susannah J Brock, Prantik Das, Dan Ford, Joanna Gale, Warren Grant, Emma K Gray, Peter Hoskin, Mohammad M Khan, Caroline Manetta, Neil J McPhail, Joe M O'Sullivan, Omi Parikh, Carla Perna, Carmel J Pezaro, Andrew S Protheroe, Angus J Robinson, Sarah M Rudman, Denise J Sheehan, Narayanan N Srihari, Isabel Syndikus, Jacob Tanguay, Carys W Thomas, Salil Vengalil, John Wagstaff, James P Wylie, Mahesh KB Parmar, Matthew R Sydes

CONTENTS

CONTENTS 1

SUPPLEMENTARY TABLES 2

SUPPLEMENTARY FIGURES 5

STAMPEDE OVERSIGHT COMMITTEES, STAFF AND COLLABORATORS 8

PROTOCOL 9

SUPPLEMENTARY TABLES

Table S1: Post-progression treatment

Patients with reported progression*	SOC-alone n=437	(100%)	SOC+AAP n=282	(100%)	p
Any second-line treatment	405	(93%)	217	(77%)	<0.0001
Anti-androgen	316	(72%)	84	(30%)	<0.0001
Abiraterone	131	(30%)	10	(4%)	<0.0001
Enzalutamide	157	(36%)	46	(16%)	<0.0001
Docetaxel	199	(46%)	137	(49%)	0.424
Zoledronic acid	81	(19%)	51	(18%)	0.88
Dexamethasone	116	(27%)	66	(23%)	0.344
Prednisolone	93	(21%)	43	(15%)	0.044

* Biochemical progression, local progression, metastatic progression or skeletal-related event

Note: SOC = Standard-of-care, AAP = Abiraterone acetate + prednisolone

Table S2: Worst grade toxicity reported two years after randomisation*, and four years after randomisation*

	Two years					Four years				
	SOC-alone		SOC+AAP			SOC-alone		SOC+AAP		
**Grade**	**N=136**	**(100%)**	**N=291**	**(100%)**	**p**	**N=72**	**(100%)**	**N=197**	**(100%)**	**p**
0	36	(27%)	59	(21%)	0.29	5	(10%)	18	(12%)	0.56
1	63	(47%)	143	(50%)		25	(50%)	57	(38%)	
2	22	(17%)	64	(22%)		12	(24%)	51	(34%)	
3	12	(9%)	20	(7%)		8	(16%)	23	(15%)	
4	0	(0%)	0	(0%)		0	(0%)	1	(1%)	
5	0	(0%)	0	(0%)		0	(0%)	0	(0%)	
Missing	*3*	*n/a*	*5*	*n/a*		*22*	*n/a*	*47*	*n/a*	

* Toxicity data is collected until first progression in the SOC-alone group and until the end of abiraterone treatment for SOC+AAP patients. This determines the number of patients included in the denominator at two and four years, with patients counted as missing if data is expected but not reported within a 12-week window.

Note: SOC = Standard-of-care, AAP = Abiraterone acetate + prednisolone

Table S3: Overall survival and Failure-free survival, at time of primary analysis and at long-term follow-up

	Primary analysis (2017)		Long-term follow-up analysis (2020)	
	SOC-alone n=502	SOC+AAP n=501	SOC-alone n=502	SOC+AAP n=501
**Overall survival**				
Events	218	150	329	244
Proportion event-free at 5 yrs (95%CI)	0.43 (0.36,0.50)	0.58 (0.47,0.67)	0.41 (0.37,0.45)	0.60 (0.55,0.64)
SOC+AAP vs. SOC-alone, HR=(95%CI) p	0.61 (0.49,0.75)	<0.0001	0.60 (0.50,0.71)	<0.0001
p (proportional hazards)		0.33		0.78
RMST (months)	42 (40,44)	48 (46,50)	54 (51,57)	66 (63,69)
**Failure-free survival**				
Events	393	210	437	282
Proportion event-free at 5 yrs (95%CI)	0.18 (0.15,0.22)	0.51 (0.44,0.57)	0.13 (0.11,0.17)	0.45 (0.41,0.50)
SOC+AAP vs. SOC-alone, HR=(95%CI) p	0.31 (0.26,0.37)	<0.0001	0.34 (0.29,0.40)	<0.0001
p (proportional hazards)		0.0199		0.0001
RMST (months)	20 (18,23)	40 (38,43)	24 (21,27)	55 (51,59)

Note: SOC = Standard-of-care, AAP = Abiraterone acetate + prednisolone,

RMST = restricted mean “survival” (event-free) time, CI = confidence interval,

yr = years, HR = hazard ratio

SUPPLEMENTARY FIGURES

Figure S1: Time from randomisation to event for two secondary outcome measures

(A) Progression-free survival

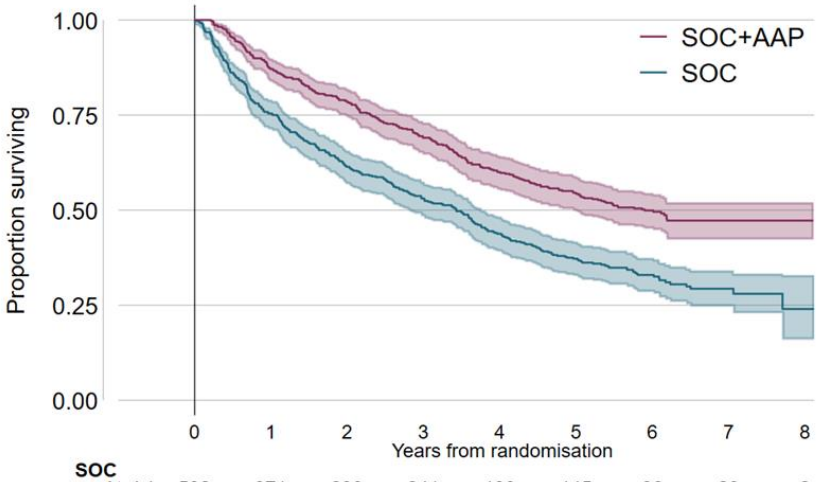

	0	1	2	3	4	5	6	7	8
**SOC**									
At-risk	502	371	293	241	190	145	86	29	2
Censored	0	9	19	32	42	59	102	152	177
Progressed	0	122	190	229	270	298	314	321	323
**SOC+AAP**									
At-risk	501	431	381	318	269	232	146	47	4
Censored	0	6	14	32	40	52	120	213	256
Progressed	0	64	106	151	192	217	235	241	241

(B) Metastatic progression-free survival

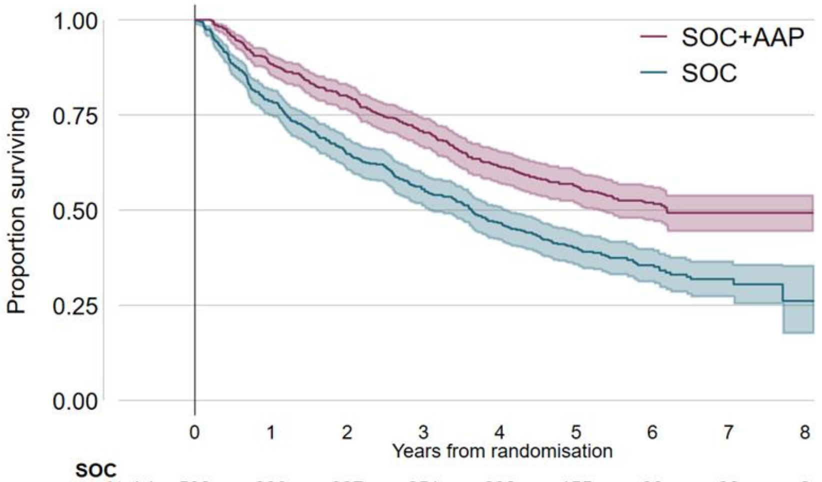

	0	1	2	3	4	5	6	7	8
**SOC**									
At-risk	502	386	307	251	202	155	92	32	2
Censored	0	10	22	35	45	64	110	163	191
Progressed	0	106	173	216	255	283	300	307	309
**SOC+AAP**									
At-risk	501	437	386	321	272	237	151	48	5
Censored	0	6	16	36	45	57	126	223	266
Progressed	0	58	99	144	184	207	224	230	230

Figure S2: Time from randomisation to event for two secondary outcome measures

(A) Skeletal-related events

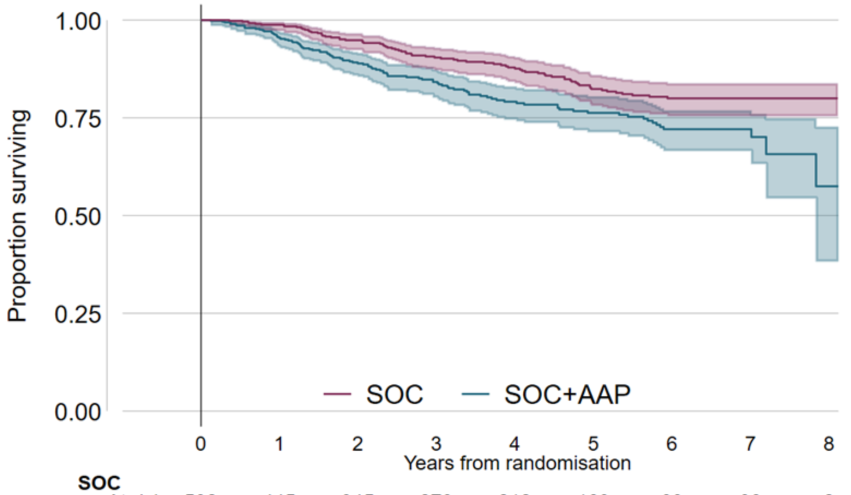

	0	1	2	3	4	5	6	7	8
**SOC**									
At-risk	502	445	345	270	212	163	89	38	2
Censored	0	35	107	165	208	250	316	367	400
Event	0	22	50	67	82	89	97	97	100
**SOC+AAP**									
At-risk	501	473	408	339	297	259	163	53	6
Censored	0	22	69	120	152	173	262	372	419
Event	0	6	24	42	52	69	76	76	76

(B) Disease-specific survival

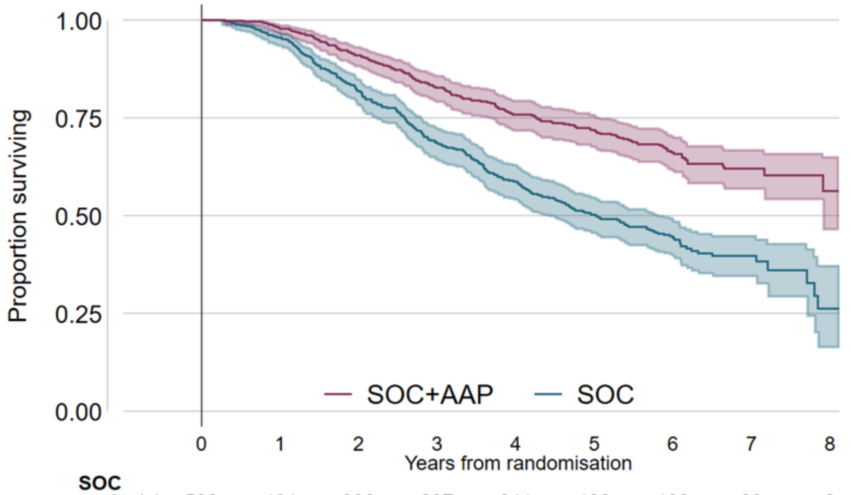

	0	1	2	3	4	5	6	7	8
**SOC**									
At-risk	502	464	380	297	241	182	100	39	2
Censored	0	16	35	58	73	98	160	213	245
Progressed	0	22	87	147	188	222	242	250	255
**SOC+AAP**									
At-risk	501	474	421	357	314	284	176	56	6
Censored	0	16	37	64	78	92	179	291	339
Progressed	0	11	43	80	109	125	146	154	156

Figure S3: Time to from first progression event to starting of reported further treatment

(A) Abiraterone

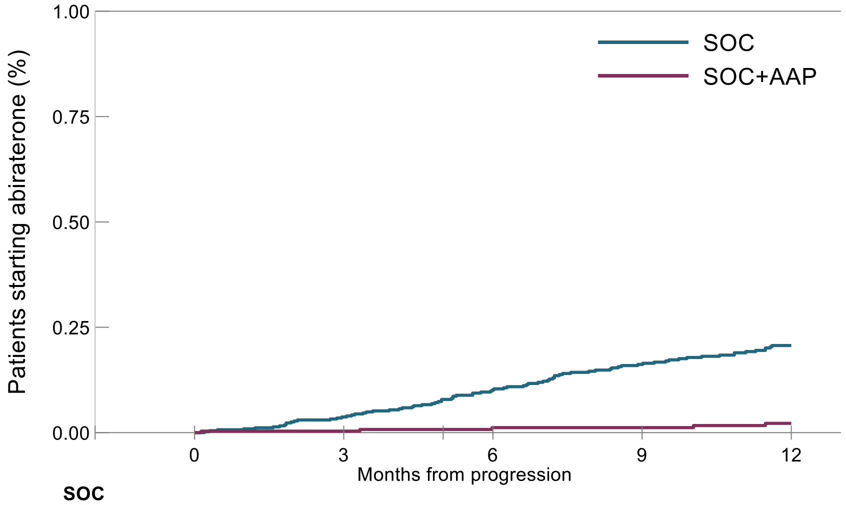

	0	3	6	9	12
**SOC**					
Progressed	437	405	356	309	274
Censored	0	16	39	63	82
Started	0	16	42	65	81
**SOC+AAP**					
Progressed	282	259	230	212	185
Censored	0	22	49	67	92
Started	0	1	3	3	5

(B) Docetaxel (outside of treatment allocation)

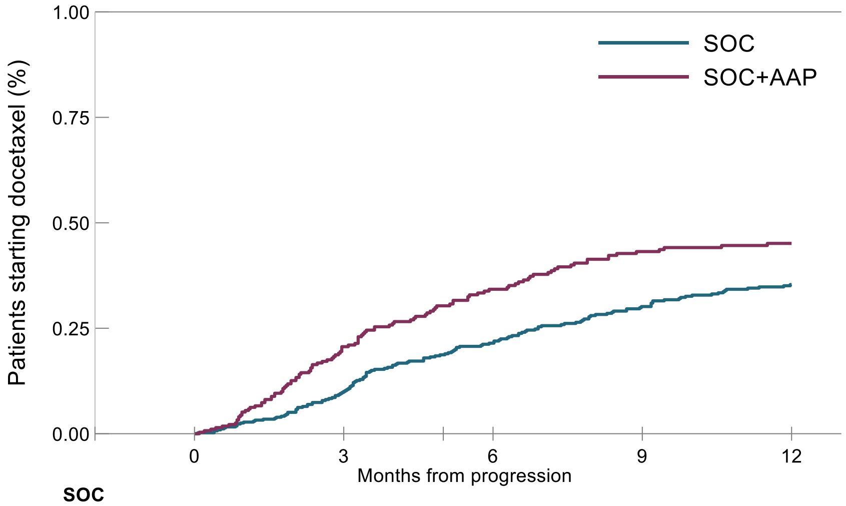

	0	3	6	9	12
**SOC**					
Progressed	437	380	310	261	230
Censored	0	14	37	53	65
Started	0	43	90	123	142
**SOC+AAP**					
Progressed	282	208	153	127	112
Censored	0	19	41	47	58
Started	0	55	88	108	112

STAMPEDE OVERSIGHT COMMITTEES, STAFF AND COLLABORATORS

See separate **supplementary document** for pdf list of STAMPEDE oversight committees, staff and collaborators.

Any subsequent updates may also be found through:
http://www.stampedetrial.org/media-section/presentation-repository/trial-recognition/

PROTOCOL

Pertinent versions of the protocol are provided as separate **supplementary documents**.

A full list of previous protocol versions can be found via the trial website:
http://www.stampedetrial.org/
