# Supplementary material for: Abiraterone acetate plus prednisolone for metastatic patients starting hormone therapy: 5‐year follow‐up results from the STAMPEDE randomised trial (NCT00268476)
Source: Int J Cancer. 2022 May 16;151(3):422–34. doi: 10.1002/ijc.34018 (PMC9321995; doi:10.1002/ijc.34018)
Supplement: Supplementary file 2 — Appendix S2Supporting Information. [file IJC-151-422-s001.zip › IJC_34018_STAMPEDE_Protocol_v1.1.pdf]

STAMPEDE

Systemic Therapy in Advancing or Metastatic Prostate Cancer: Evaluation of Drug Efficacy

A 5-stage multi-arm randomised controlled trial

Developed on behalf of the NCRI Prostate Clinical Studies Group

MRC PR08
ISRCTN number: ISRCTN78818544
EUDRACT number: 2004-000193-31

Part of the National Cancer Research Network Portfolio

PROTOCOL VERSION 1.1

04 May 2005

Authorised by:

Name	**Professor Mahesh KB Parmar**	Signature	
Role	**Head, CTU Cancer Division**	Date	**04 May 2005**
Name	**Professor Nicholas D James**	Signature	
Role	**Chief Investigator**	Date	**04 May 2005**

GENERAL INFORMATION

This document describes a trial coordinated by the Medical Research Council (MRC) Clinical Trials Unit (CTU) and provides information about procedures for entering patients into it. The protocol should not be used as an aide-memoire or guide for the treatment of other patients; every care was taken in its drafting, but corrections or amendments may be necessary. These will be circulated to the known investigators in the trial, but centres entering patients for the first time are advised to contact the Cancer Division, MRC CTU, London to confirm they have the most up to date version. Clinical problems relating to this study should be referred to the Chief Investigator. There is also an abridged version of this protocol that is intended primarily to provide only the essential clinical details to healthcare professionals who are working directly with patients.

Sponsor

Medical Research Council, 20 Park Crescent, London, W1B 4AL

Funding

Clinical Trials Advisory Awards Committee (on behalf of Cancer Research UK, Medical Research Council, and other charities) together with educational grants from Novartis and Aventis.

Compliance

This trial will adhere to the principles outlined in the International Conference on Harmonization (ICH) Good Clinical Practice (GCP) guidelines. It will be conducted in compliance with the protocol, MRC GCP, Data Protection Act (DPA number: G0027154) and other regulatory requirements, as appropriate.

Authorisation

The following persons are authorised to sign the final protocol and protocol amendments for the sponsor: Professor N James (Chief Investigator) and Professor M Parmar (Head, Cancer Division, MRC Clinical Trials Unit).

RANDOMISATIONS

To randomise call MRC CTU, Monday to Friday 0900-1700

Tel: 020 7670 4777

TRIAL ADMINISTRATION

Chief Investigator
Prof Nicholas James
CRUK Institute for Cancer Studies
University of Birmingham
Edgbaston, Birmingham
B15 2TT
Tel: 0121 414 4097/7584
Fax: 0121 414 3263
Email: n.d.James@bham.ac.uk
d.meredith@bham.ac.uk (secretary)

Co-Investigators
John Anderson, Urologist , Sheffield
Mr Noel Clarke, Urologist, Manchester
Prof David Dearnaley, Oncologist, Royal Marsden
Prof Malcolm Mason, Oncologist, Cardiff
Rick Popert, Oncologist, London
Andrew Stanley, Pharmacist, Birmingham

Patient Reprentitives
John Dwyer, Stockport, Patient
Jim Stansfeld, Hampshire, Patient

Trial Physician
---To be appointed---
Research Fellow
CRUK Institute for Cancer Studies
University of Birmingham
Edgbaston, Birmingham
B15 2TT
Tel: ---
Email: ---

Health Economics
Prof Mark Sculpher, York

Quality of Life
Richard Stephens, MRC CTU, London

Molecular Genetics
Prof John Masters, Pathologist, London

For full details of all trial contacts please see Appendix L

Coordinating Centre
MRC Clinical Trials Unit
Cancer Division
222 Euston Road
London
NW1 2DA

Tel: 020 7670 4700
Fax: 020 7670 4818
Email: stampede@ctu.mrc.ac.uk

MRC Clinical Trials Unit Staff

Trial Manager:	Jacqui Nuttall	020 7670 4831	stampede@ctu.mrc.ac.uk
Data Manager:	Shama Hassan	020 7670 4844	stampede@ctu.mrc.ac.uk
Senior Trial Statistician:	Matthew Sydes	020 7670 4798	ms@ctu.mrc.ac.uk
Head of Cancer Group:	Prof Max Parmar	020 7670 4729	mp@ctu.mrc.ac.uk

For general queries, supply of trial materials and collection of data please contact the STAMPEDE trial manager

Clinical queries during office hours should be directed to the Chief Investigator, Professor Nick James or the Trial Physician (see above). Out of hours, please call Queen Elizabeth Hospital switchboard on (0121) 472 1311 and ask to bleep Professor James.

CONTENTS

1 Summary 6
1.1 LAY SUMMARY 6
1.2 ABSTRACT AND SUMMARY OF TRIAL DESIGN 7
1.3 TRIAL DOCUMENTATION 8

2 Background 9
2.1 INTRODUCTION AND RATIONALE 9
2.2 BISPHOSPHONATES 9
2.3 CHEMOTHERAPY 10
2.4 CYCLOOXYGENASE-2 INHIBITORS 11
2.5 TREATMENT COMBINATIONS 12

3 Selection of Institutions and Investigators 12

4 Selection of Patients 14
4.1 PATIENT INCLUSION CRITERIA 14
4.2 PATIENT EXCLUSION CRITERIA 15
4.3 SCREENING PROCEDURES 15

5 Randomisation and Enrolment 16
5.1 CO-ENROLMENT GUIDELINES 17

6 Treatment of Patients 17
6.1 TRIAL TREATMENT 17
6.2 ADMINISTRATION AND DOSE MODIFICATIONS 19
6.3 TRIAL PRODUCTS 20
6.4 MEASURES OF COMPLIANCE/ADHERENCE 20
6.5 TREATMENT DATA COLLECTION 20
6.6 NON-TRIAL TREATMENT 20

7 Assessments and Procedures 21
7.1 FLOW CHART/SCHEDULE FOR FOLLOW-UP 21
7.2 EFFICACY PARAMETERS 21
7.3 FOLLOW-UP22
7.4 TRIAL CLOSURE 22

8 Withdrawal of Patients 24
8.1 WITHDRAWAL FROM TRIAL INTERVENTIONS 24
8.2 WITHDRAWAL FROM THE TRIAL COMPLETELY 24
8.3 PATIENT TRANSFERS 25

9 Statistical considerations 25
9.1 METHOD OF RANDOMISATION 25
9.2 OUTCOME MEASURES 25
9.3 SAMPLE SIZE 26
9.4 INTERIM MONITORING AND ANALYSES 29
9.5 OUTLINE ANALYSIS PLAN 29

10 Data Verification and Site Monitoring 30
10.1 VERIFICATION OF DATA AT MRC CTU 30
10.2 CLINICAL SITE MONITORING 30

11 Safety Reporting 31
11.1 ADVERSE EVENTS AND GRADING OF ADVERSE EVENTS 31
11.2 INSTITUTION RESPONSIBILITIES 32
11.3 MRC CTU RESPONSIBILITIES 33
11.4 SEVERITY/GRADING OF ADVERSE EVENTS 34
11.5 RELATIONSHIP TO TRIAL TREATMENT 34
11.6 FOLLOW-UP AFTER ADVERSE EVENTS 34

12 Ethical considerations and approval 35
12.1 ETHICAL CONSIDERATIONS 35
12.2 ETHICAL APPROVAL 35

13 Regulatory Approval 36

14 Indemnity 36

15 Finance 36

16 Trial Committees 37
16.1 TRIAL MANAGEMENT GROUP (TMG) 37
16.2 TRIAL STEERING COMMITTEE (TSC) 37
16.3 INDEPENDENT DATA MONITORING COMMITTEE (IDMC) 37

17 Ancillary studies 39
17.1 QUALITY OF LIFE 39
17.2 HEALTH ECONOMICS 39
17.3 MOLECULAR GENETICS 40

18 Publication 40

19 Protocol Amendments 41

20 References 43

21 Appendices 46
APPENDIX A – ADDITIONAL DEFINITIONS 47
APPENDIX B - PATIENT INFORMATION SHEETS & CONSENT FORM 49
APPENDIX C - GP LETTER 82
APPENDIX D - MOLECULAR GENETICS SUB-STUDY 84
APPENDIX E - DRUG SUPPLY INFORMATION 85
APPENDIX F - ADMINISTRATION OF DOCETAXEL AND ZOLEDRONIC ACID 86
APPENDIX G - DRUG SAFETY INFORMATION FOR DRUGS USED IN THE TRIAL 93
APPENDIX H - HEALTH CARE AT HOME STUDY 103
APPENDIX I - EVALUATION OF BASELINE LESIONS AND NEW LESIONS 104
APPENDIX J - COMMON TOXICITY CRITERIA 106
APPENDIX K - DEFINITION OF BIOCHEMICAL FAILURE 110
APPENDIX L - TRIAL CONTACTS 113
APPENDIX M - PARTICIPATING SITE ACCREDITATION FORM 116
APPENDIX N - QUALITY OF LIFE AND HEALTH ECONOMICS 119
APPENDIX O - CASE REPORT FORMS 122
APPENDIX P - ASSESSING AND NOTIFYING CTU OF ADVERSE EVENTS 123

LIST OF FIGURES

Figure 1: Arms of the STAMPEDE trial
Figure 2: Summary of timing of trial documentation
Figure 3: Detailed schedule for completion of forms
Figure 4: Progress of STAMPEDE through the trial stages
Figure 5: Diagram of relationships between trial committees

LIST OF TABLES

Table 1: Outcome Measures
Table 2: Hazard Ratio assumptions under null and alternative hypothesis
Table 3: Guidelines for stopping accrual to the ith research arm
Table 4: Terms and definitions for adverse events
Table 5: Adverse events; some inclusions and exclusions

ABBREVIATIONS AND GLOSSARY

**ACE**	Angiotensin-Converting Enzyme
**AS**	Androgen suppression
bid	Twice a day *(bis in die)*
**BP**	Blood pressure
**BSA**	Body surface area
**CERES**	Consumers for Ethics in Research
**CF**	Consent Form
**CI**	Chief Investigator
**CI**	Confidence interval
**COSTART**	Coding Symbols for a Thesaurus of Adverse Reaction Terms
**Cox-2**	Cyclooxygenase-2
**CRF**	Case Report Form
**CRUK**	Cancer Research UK
**CT**	Computerised tomography
**CTA**	Clinical Trials Authorisation
**CTAAC**	Clinical Trials Advisory and Awards Committee
**CTC**	Common Toxicity Criteria
**CTU**	Clinical Trials Unit
**CXR**	Chest X-ray
**DDX**	Doctors and Dentists Exemption
**DNA**	Deoxyribonucleic Acid
**DPA**	Data Protection Act
**ERC**	Endpoint Review Committee
**ICH**	International Conference on Harmonization
**ECG**	Electro cardiogram
**FBC**	Full Blood Count
**FFS**	Failure Free Survival
**GCP**	Good Clinical Practice
**GP**	General Practitioner
**GRO**	General Register Office
**HE**	Health Economics
**hr**	Hour
**HR**	Hazard Ratio
**HRPC**	Hormone Refractory Prostate Cancer
**IDMC**	Independent Data Monitoring Committee
**IM**	Intramuscular
**ISRCTN**	International Standard Randomised Controlled Trial Number
**IU**	International Units
**IV**	Intravenous
**LD**	Longest diameter
**LFT's**	Liver Function Tests
**LHRH**	Luteinising Hormone Releasing Hormone
**LREC**	Local Research Ethics Committee
**MHRA**	Medicine and Healthcare Products Regulatory Agency
**min**	Minutes
**MRC**	Medical Research Council
**MREC**	Multi-Centre Research Ethics Committee
**MRI**	Magnetic resonance imaging
**NCI**	National Cancer Institute (USA)
**NCRN**	National Cancer Research Network
**NHS**	National Health Service
**NSAID**	Non-Steroidal Anti-inflammatory Drugs
**ONS**	Office for National Statistics
**OS**	Overall Survival

**PI**	Principal Investigator
**PIS**	Patient Information Sheet
po	*per orum* (orally)
**PSA**	Prostate Specific Antigen
**QALY**	Quality-adjusted Life Years
qds	*quater die sumendus* (4 times each day)
**QL**	Quality of Life
**R&D**	Research and Development
**RECIST**	Response Evaluation Criteria In Solid Tumours
**SAE**	Serious Adverse Event
sc	*Sub-cutaneous* (under skin)
**SNP**	Single Nucleotide Polymorphism
**SSA**	Site Specific Assessment
**STAMPEDE**	Systemic Therapy in Advancing and Metastatic Prostate Cancer: Evaluation of Drug Efficacy
**SUSAR**	Suspected Unexpected Serious Adverse Reactions
**TMG**	Trial Management Group
**TURP**	Trans-Urethral Resection of Prostate
**TSC**	Trial Steering Committee
**ULN**	Upper Limit of Normal
**U+E**	Urea and Electrolytes
**WHO**	World Health Organisation

1 SUMMARY

1.1 Lay summary

Prostate cancers depend upon the male hormone testosterone for their growth. Lowering testosterone levels (either by removing all or part of both testes, or by giving anti-hormone injections) slows the growth of prostate cancers. This type of treatment is called hormone treatment and is often used when prostate cancers have spread outside the prostate gland. Although hormone treatment is usually successful at stopping the cancer growing for a period of time, the cancer will begin to grow again in most men.

A number of newer treatments have recently become available and have shown initial promise against prostate cancer. Newer treatments are usually used in prostate cancer when hormone treatment is no longer effective and the cancer has started to grow again. The aim of this trial, which is called STAMPEDE, is to assess three of these newer treatments, given earlier in the course of the disease in combination with hormone treatment.

The three new treatments to be assessed are: -

1. Zoledronic acid: Prostate cancer cells can spread to bones and weaken them. Zoledronic acid is a drug that reduces bone destruction and hardens bones. This may make them more resistant to attack by cancer cells.

2. Docetaxel: A drug that stops cells replicating that is currently being used to treat lung, breast and ovarian cancer.

3. Celecoxib: An aspirin-like drug that is used to treat arthritis. It slows down the growth of cancer cells in the laboratory. We wish to see if it has the same effect on cancer cells in patients.

STAMPEDE will look at the effect of combining one or two of the new treatments described above with hormone treatment. A computer program will be used to allocate which treatment the patient receives, using a chance process. Two in every seven patients will receive hormone treatment only; the other five in seven patients will be split equally between the 5 newer combined treatment options. The trial will look at the effects of the combined treatments on quality of life and find out whether the new treatment combinations increase the time when the cancer is not growing and result in patients living longer. The study will also look at which treatment provides the greater value for money for the health service. Approximately 3,300 patients will participate in the trial and it will take between 6-10 years to complete.

1.2 Abstract and summary of trial design

STAMPEDE is a multi-centre, randomised controlled trial for patients with locally advanced or metastatic prostate cancer who are about to commence androgen suppression (AS) therapy. Patients can have either newly diagnosed disease, or have been previously treated with radical radiotherapy or surgery but now have a rising prostate specific antigen (PSA) (further details on eligibility see section 4). The trial will assess the effects of adding three different agents, both as single agents and in combinations, to AS therapy. The investigational agents are (i) a bisphosphonate, zoledronic acid, (ii) a cytotoxic chemotherapeutic agent, docetaxel and (iii) a cyclooxygenase (Cox-2) inhibitor, celecoxib. The trial has six arms; the control arm of the trial is AS only, achieved through the use of luteinising hormone releasing hormone (LHRH) analogues or bilateral orchidectomy according to local practice. The other trial arms are summarised in Figure 1.

Figure 1 - Arms of the STAMPEDE Trial

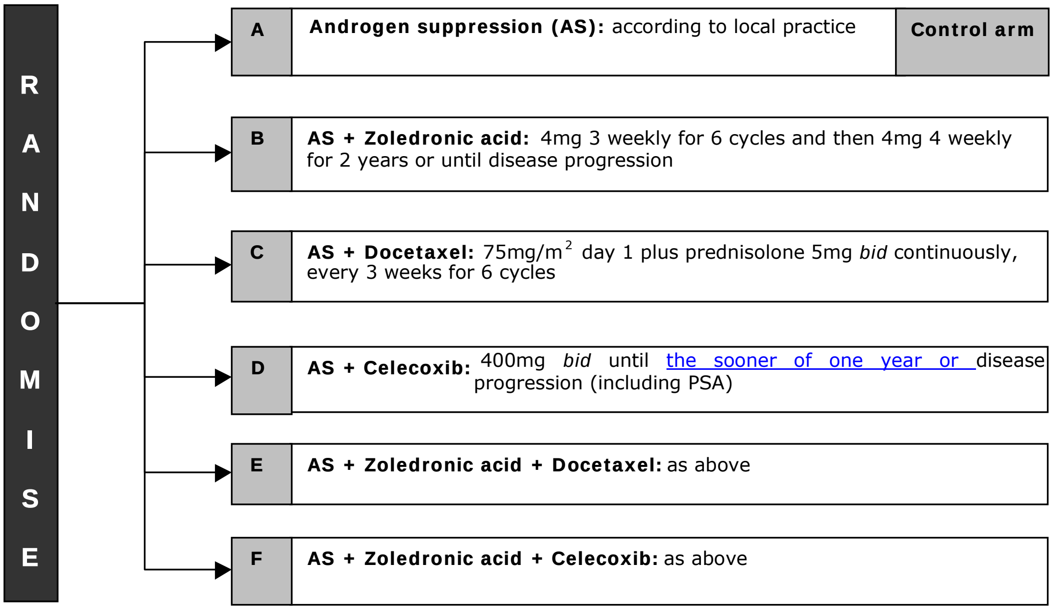

AS = Androgen suppression as in Arm A

The trial will be conducted in five stages: a Pilot Phase, Efficacy Stages I to III and Efficacy Stage IV. The primary outcome measure of the Pilot Phase is the safety of the investigational arms and 210 patients will be recruited. Investigational arms will only proceed to recruitment in the next stage if they have been shown to be both safe and feasible, although patient data from all patients and all stages will be included in the final analyses. In Efficacy Stages I-III the primary outcome measure is failure free survival (FFS). Further patients will be recruited until 115, 225 and 355 FFS events have been observed in the control arm. Some evidence of efficacy will be

required for an research arm to proceed to further recruitment in each stage and guidelines are in place. In Efficacy Stage IV, patients will be recruited until 440 deaths have been observed in the control arm. Overall, approximately 3,300 patients will be recruited over 5-7 years although the exact number of patients and duration will depend on the observed accrual rate, observed event rate and the number of patients accruing at each stage.

Patients will be assessed 6 weekly for the first 24 weeks after randomisation and then every 12 weeks up to 2 years. Assessments will then be 3-monthly for the next 2 years and then 6-monthly, thereafter. Patients will also be asked to complete questionnaires aimed at assessing the effects of the investigational treatments on their quality of life (QL) and on their use of health care resources (HE study).

In addition, there is a molecular genetics substudy. Patients willing to participate will be asked to donate 8ml of blood which will be stored for either DNA and protein analysis in order to try to identify markers that are associated with response to therapy, side-effects or susceptibility to prostate cancer. There are separate patient information sheets for the QL and HE study, and the molecular genetics study (For further details of ancillary studies see section 16).

1.3 Trial Documentation

Figure 2 presents a summary of the timing of the required trial documentation for participating centres and their randomised patients.

Figure 2 - Summary of timing of trial documentation

Trial documentation	Timing
Centre accreditation form	Before centre participation
PIS and CF on local paper	Before centre accreditation
R&D approval	Before centre accreditation
GP letter	Before centre participation
SSA Favourable Opinion	Before centre participation – Main REC
Eligibility log	To be appended for each considered patient
Randomisation form	At randomisation
Baseline form	At randomisation
Cardiovascular assessment form	At randomisation
Treatment forms	Every 6wks until 18wks, then with FU forms
FOLLOW-UP FORMS	Every 12wks until 2yrs then 3 months until 5yrs, then 6 monthly
Toxicity form	Every 12wks until 2yrs then 3 months until 5yrs, then 6 monthly
Quality of life forms	At 6, 12, 18, 24 and 52wks, then annually
Progression forms	*As needed*
Serious adverse event form	*As needed*
Death form	*As needed*

2 BACKGROUND

2.1 Introduction and Rationale

Prostate cancer is a major health problem world-wide and accounts for nearly one fifth of all newly diagnosed male cancers. In the UK, approximately 25,000 men are diagnosed with prostate cancer each year and in 2001 almost 10,000 men died from the disease (1).

The initial (first line) treatment for locally advanced or metastatic prostate cancer is androgen suppression (AS) achieved either surgically with bilateral orchidectomy, or medically with LHRH analogues (2). AS produces responses in up to 85% of patients (3) but it is not curative and disease recurs in virtually all patients, with a median time to progression of 18-24 months. Such disease is referred to as hormone refractory prostate cancer (HRPC).

There are several treatments, which are used 'second-line' in patients with HRPC, but no evidence as to which is associated with the best response or whether any of them might have a role in first-line treatment; these include further hormonal manipulations (4;5), bisphosphonates (6), cytotoxic chemotherapy (3) and novel agents (7). The traditional approach to the testing and introduction of new treatments for prostate cancer is in hormone refractory disease. An alternative approach is to investigate new drugs and new approaches to treatment as first-line therapy in patients starting hormone therapy. At this point patients would be fitter and better able to tolerate treatment than when they have HRPC, and there is also the possibility of having a larger and more durable effect.

STAMPEDE (as known as MRC PR08) is an innovative, 6-arm, multi-centre, randomised controlled trial. It assesses the effects of a bisphosphonate (zoledronic acid), a cytotoxic chemotherapeutic agent (docetaxel) and a cyclooxygenase (Cox-2) inhibitor (celecoxib), as single agents or combinations, in patients commencing AS therapy for advancing or metastatic prostate cancer. The trial is divided into five stages such that, for each investigational arm, safety and activity data are generated in the first four stages; an investigational arm will only proceed to the fifth and final stage of recruitment, where it will be assessed for its effect on overall survival, if it has been shown to be safe and active. It is important to note, however, that patient data from all arms and all stages will be included in the final analyses of the primary outcome measure, even if the investigational arm did not proceed to the final stage.

2.2 Bisphosphonates

The bisphosphonates are a class of drug that act by reducing osteoclast formation, inhibiting osteoclast activity and inducing osteoclast apoptosis. They are effective at controlling hypercalcaemia and preventing skeletal complications associated with malignant disease (8).

Zoledronic acid is a new, highly potent, third generation bisphosphonate; studies comparing the efficacy of zoledronic acid to other bisphosphonates suggest that zoledronic acid has a 40-850 fold higher potency than clodronate in preclinical models of bone resorption (9). It has also been shown to be more effective than pamidronate (90mg) in controlling malignant hypercalcaemia (10). In addition, zoledronic acid has also demonstrated direct anti-cancer activity, including inhibition of proliferation of breast cancer and prostate cancer cells *in vitro* (11).

In randomised controlled trials of 1,648 patients, 4mg zoledronic acid was more effective than pamidronate in reducing the risk of skeletal complications in patients with bone metastases from breast cancer (12). Also, in metastatic prostate cancer, zoledronic acid has been shown to reduce the rate of skeletal related events compared to placebo in a trial involving 429 men (13). In April 2002, zoledronic acid received approval from the Committee for Propriety Medicinal Products for the prevention of skeletal related events (for example, fractures) in patients with any advanced malignancies involving bone.

The MRC PR05 prostate cancer trial showed that a first generation bisphosphonate (clodronate) commenced at the time of hormone therapy initiation, delayed time to progression in patients with bony metastatic disease and there was some evidence that it may also improve survival (14). There is, therefore, a good rationale for investigating a more potent bisphosphonate in patients with prostate cancer who are about to commence AS therapy.

2.3 Chemotherapy

Over recent years there has been increasing evidence of the clinical efficacy of chemotherapy in prostate cancer. One of the most active agents is docetaxel, a semi-synthetic taxane that binds microtubules and inhibits mitosis (15). In phase I and II trials, docetaxel exhibited significant activity in prostate cancer in dosing regimens in the range of 40 to 75 mg/m^2, administered every three weeks. Evidence of this activity includes PSA decline, objective response in bi-dimensionally measurable lesions and improvements in pain control. The safety profile was assessed as being acceptable throughout these studies with a good risk/benefit ratio in this clinical setting (16-19). In addition, the concurrent administration of prednisolone has been shown to materially decrease the toxicity associated with docetaxel (20).

Recent evidence also indicates that docetaxel compares favourably with mitozantrone which had previously been considered the most active chemotherapy agent against prostate cancer. A phase II trial reported PSA response rates of 38-46% with docetaxel with prednisolone compared to 28% for mitozantrone plus prednisolone (21).

More recently two metastatic phase III studies in patients with androgen-independent prostate cancer (AIPC) using a docetaxel-containing regimen have been completed: the SWOG 9916 study (22) and the TAX 327 study (23). Both studies show that the use of a docetaxel-based regimen improved survival for patients with metastatic AIPC and had significantly greater PSA response rates compared to the mitoxantrone plus prednisolone arm.

In the TAX 327 trial (23), 1,006 patients with metastatic AIPC were randomized to receive either mitoxantrone 12 mg/m² with prednisone 10 mg daily (Arm C) or docetaxel 75 mg/m² q3weekly x 10 cycles and prednisone (Arm A) or docetaxel 30 mg/m²/wk x 5 of 6 weeks x 5 cycles with prednisone (Arm B). Median overall survival was 16.5 months for patients treated with mitoxantrone versus 18.9 months for the 3 weekly docetaxel regimen (hazard ratio 0.76 (0.62-0.94)). There was also improvements for 3 weekly docetaxel in pain (22% Vs 35%, p = 0.01) and PSA response (32% Vs 45%, p = 0.0005)

2.4 Cyclooxygenase-2 inhibitors

Cyclooxygenase-2 (Cox-2) is an isoenzyme induced by a variety of mitogens, cytokines and growth factors that are associated with inflammation, ovulation and carcinogenesis (24-26). There is a growing body of evidence that inhibition of Cox-2 may play an important role in the prevention of cancer and the delay of progression in established cancer. A number of case-control studies have shown a reduction in risk of prostate cancer associated with the use of non-steroidal anti-inflammatory drugs (NSAID), which include inhibition of Cox-2 amongst their mode of action (27). Pathological studies show Cox-2 is upregulated in prostate carcinoma (28) and one study suggested that NSAID use may delay progression from subclinical to clinical prostate cancer (29).

Celecoxib, a Cox-2 inhibitor, is better tolerated than other NSAIDs and there is evidence that it is active as a chemoprevention agent (30). It also has important antineoplastic properties such as the ability to inhibit angiogenic factors and induce apoptosis in human cancer cells including prostate cancer (31;32).

Evidence has suggested that an anti-cancer effect is only seen at higher doses of celecoxib than required for an anti-inflammatory effect (33). Therefore, the dose of 800mg/day for STAMPEDE patients has been chosen. Although there is some high profile evidence of a small absolute increase in CVS toxicity risk associated with higher doses of celecoxib (34), most current cancer trials are using a dose of 800mg/day as it is believed that a higher dose will result in a greater increase in cancer effect.

There is also some evidence of a schedule effect on CVS toxicity. It has been observed that CVS toxicity becomes evident after 1 year of taking celecoxib (34). Therefore a maximum duration of

1 year has been set for celecoxib use in this trial. Any potential risks of course have to be weighed against any potential benefits of celecoxib in the delay of progression in established prostate cancer.

Given case-control data suggesting effects on prostate cancer, pathological expression of Cox-2 in prostate cancer and *in vitro* data suggesting that inhibition of Cox-2 inhibits growth and invasiveness, further investigation in prostate cancer is warranted.

2.5 Treatment Combinations

Bisphosphonate and Chemotherapy

Zoledronic acid and docetaxel have different mechanisms of action. In addition to its skeletal protection activity, zoledronic acid has shown direct activity against prostate cancer cells, both *in vitro* and *in vivo* (11;35;36). There is also *in vitro* and *in vivo* evidence to suggest synergy between zoledronic acid and chemotherapy in breast cancer cells and anti-angiogenic effects in patients (37;38).

Toxicities of the two agents are complementary and administration in combination is expected to be feasible and safe. These aspects will be evaluated in the initial Pilot Phase of the trial. Since both agents show considerable promise as single agents and there is *in vitro* evidence of synergy, we believe there is a strong rationale for evaluating these two agents in combination.

Bisphosphonate and Cyclooxygenase-2 Inhibitors

An alternative approach to combination therapy is to target the principal site of relapse and a key mode of progression and this is the rationale for combining zoledronic acid with a Cox-2 inhibitor. Bisphosphonates have already been shown to delay bone disease progression in hormone refractory disease (14). Cox-2 appears to play a crucial role in the molecular phenotype of advanced prostate cancer as outlined above, and this effect is likely to be apparent in both soft tissue and in bone. Toxicities of the two agents are likely to be complementary and there is no strong *a priori* reason to anticipate unacceptable toxicity. The Pilot Phase of the trial will evaluate tolerability and safety of the combination. Targeting both bone progression and the underlying molecular changes leading to progression can be expected to have synergistic benefits in terms of delaying development of hormone refractory disease.

3 SELECTION OF INSTITUTIONS AND INVESTIGATORS

Centres who wish to participate in the trial should be registered with the Medical Research Council Clinical Trials Unit (MRC CTU). Before any patients are randomised the MRC CTU must

receive a completed and signed Investigator Statement. The STAMPEDE investigator statement is signed by the Principal Investigator for that institution on behalf of all staff at that site who will be working on the STAMPEDE trial (**Appendix M**). A favourable Site Specific Assessment (SSA) and R&D approval for the site is also required before recruitment can begin.

In addition and in compliance with ICH GCP all institutions participating in the trial will complete a delegation log and forward this to the MRC CTU. Each person working on the STAMPEDE trial must complete a section of this log and indicate their responsibilities. The MRC CTU must be notified of any changes to trial personnel and/or their responsibilities. An up-to-date copy of this log must be stored in the trial master file at the institution and also at the MRC CTU.

Prior to entering patients into the trial the MRC CTU must receive full contact details for all site personnel. This must be updated whenever there are changes to trial staff or their contact details. The Clinical Trial Authorisation (CTA) for the STAMPEDE trial requires that the Medicines and Healthcare Products Regulatory Agency (MHRA) be supplied with the names and addresses of all participating investigators/institutions. Trial staff at the MRC CTU will perform this task; hence it is vital to receive full contact details for all investigators prior to their entering patients..

Finally before a patient is entered into the trial written informed consent must be obtained. Approved patient information sheets and informed consent forms are supplied in **Appendix B**. In addition, a baseline questionnaire for the QL and HE studies, must also be completed before the patient is informed which treatment has been allocated.

Only a limited number of centres will participate in the Pilot Phase of the trial; this is to ensure that safety and feasibility data are collected expediently. The other stages of the trial will be open to any centre that wishes to participate and has fulfilled the requirements described above.

4 SELECTION OF PATIENTS

4.1 Patient inclusion criteria

Patients must fulfil **one** of the criteria in section 4.1.1 or **one** of the criteria in section 4.1.2. Additionally, **all** patients must fulfil the criteria in section 4.1.3.

4.1.1 High Risk Newly Diagnosed Patients

…with one of:-

(i) Stage T3/4 N0 M0 histologically confirmed prostate adenocarcinoma with PSA≥40ng/ml or Gleason sum score 8-10

(ii) Stage T_{any} N+ M0 or T_{any} N_{any} M+ histologically confirmed prostate adenocarcinoma

(iii) Multiple sclerotic bone metastases with a PSA≥100ng/ml without histological confirmation

OR

4.1.2 Patients with histologically confirmed prostate adenocarcinoma previously treated$^{\Psi}$ with radical surgery or radiotherapy who are now relapsing

…with one of:-

(i) PSA ≥4ng/ml and rising with doubling time less than 6 months

(ii) PSA ≥20ng/ml

$^{\Psi}$**Note:** Prior hormone therapy for localised disease must have been completed at least 12 months previously and have been no longer than 12 months in duration. It can have been given as adjuvant or neoadjuvant therapy.

AND

4.1.3 For all patients:-

(i) Intention to treat with long-term androgen suppression

(ii) Fit for all protocol treatment$^{\varphi}$ and follow-up, WHO performance status 0-2 (see **Appendix A**)

(iii) Have completed the appropriate investigations prior to randomisation

(iv) Adequate haematological function: neutrophil count $\geq 1.5 \times 10^9$/l and platelets $\geq 100 \times 10^9$/l

(v) Adequate renal function: Serum creatinine $\leq$1.5 ULN

(vi) Adequate liver function: ALT or AST $\leq$1.5 ULN, bilirubin $\leq$ULN

(vii) Normal testosterone level prior to hormone treatment

(viii) Written informed consent

(ix) Willing and expected to comply with follow-up schedule

$^{\varphi}$ Note: Medical contraindications to the trial medications are given in **Appendix G**, in particular please note celecoxib is contraindicated in patients with active peptic ulceration, gastrointestinal bleeding, inflammatory bowel disease or severe congestive heart failure.

4.2 Patient exclusion criteria

(i) Prior systemic therapy for locally advanced or metastatic prostate cancer except as listed in 4.1.2.
(ii) Metastatic brain disease or leptomeningeal disease.
(iii) Any other previous or current malignant disease which, in the judgement of the responsible physician, is likely to interfere with STAMPEDE treatment or assessment.
(iv) Symptomatic peripheral neuropathy ≥ grade 2 (NCI CTC).
(v) Any surgery (e.g. TURP) performed within the past 4 weeks
(vi) Renal insufficiency with estimated creatinine clearance <30ml/min
(vii) Patients who have been on a cox-2-inhibitor for at least 6 months prior to trial entry
(viii) Patients with confirmed cardiovascular history including:
   a. Severe/unstable angina
   b. Myocardial infarction
   c. Severe cardiac failure (NYHA II-IV*)
   d. Cerebrovascular disease (eg stroke or transient ischaemic episode)

*NYHA classifications can be found in **Appendix A**

4.3 Screening procedures

4.3.1 Investigations Prior to Randomisation

All patients require the following examinations to have been performed within 5 weeks (35 days) prior to randomisation:-

- CT or MRI of pelvis and abdomen
- Bone Scan
- Chest X-ray and ECG

…and the following blood tests within 2 weeks (14 days) prior to randomisation:-

- PSA
- Testosterone (if available, also, see note below)
- Urea and Electrolytes
- Liver Function Tests
- Serum creatinine
- Serum corrected calcium
- Phosphates
- Magnesium
- Albumin
- Total Cholesterol
- HDL Cholesterol
- Systolic blood pressure

- Diastolic blood pressure

It is preferable that patients are not started on hormones prior to randomisation. However, if this has occurred, then AS therapy should have not have started more than 4 weeks (28 days) before randomisation and the baseline PSA measurement must be taken before the treatment was initiated and within the 4 week period prior to randomisation. Testosterone measurement will not be required in patients who have already commenced hormone manipulation.

For patients who are hypercalcaemic prior to randomisation and require treatment it is recommended that they are treated with a bisphosphonate and that the treatment should be discontinued when they are stabilised. For patients who are currently on a cox-2-inhibitor and who meet the inclusion criteria, please ensure that treatment is discontinued before randomisation.

4.3.2 Concomitant Medications

All concomitant medications should be recorded including regular consumption of NSAID and/or aspirin and use of other bisphosphonates (see **Section 4.3.1**). All concomitant medications should be continued throughout the trial unless the responsible physician decides otherwise.

4.3.3 Additional Details for Patients Participating in the Sub-studies

Baseline QL and HE forms must be completed before randomisation. Full details of these studies are given in Section 17.

An additional 8ml blood must be taken if the patient has given their consent to participate in the molecular genetics study. Full details of this study and instructions relating to the handling of the blood sample are given in Section 17 and **Appendix D**.

5 RANDOMISATION AND ENROLMENT

To enter a patient the randomisation form should be completed and the MRC CTU contacted by phone: -

RANDOMISATIONS

To randomise call MRC CTU, Monday to Friday 0900-1700

Tel: 020 7670 4777

A trial number and treatment will be allocated and given over the phone or by return fax. In addition, a letter confirming these details will be sent. The trial number will be the primary way in which the patient will be identified and should be used in all correspondence.

5.1 Co-enrolment guidelines

Ideally, patients should not be participating in any other clinical trial of prostate cancer treatment when they enter STAMPEDE and should not enter any other trials until the patient has had a failure-free survival (FFS) event reported. After this point, the patient may be entered into further, second-line treatment studies. The primary endpoint of STAMPEDE is survival. Therefore, follow-up to STAMPEDE must continue and must not be affected by co-enrolment to other studies. It is preferable that the MRC CTU should be notified in writing, with details of the trial: trial name, sponsor, randomisation arms, study endpoints and a declaration that STAMPEDE follow-up will not be impeded, before a patient is co-enrolled.

6 TREATMENT OF PATIENTS

6.1 Trial treatment

Patients will be randomised to the control arm (Arm A) or one of the five investigational arms. All patients will receive AS to achieve castration levels of testosterone. The method of AS is a local choice but must be specified for each patient prior to randomisation. The recommended methods of AS are given in section 6.1.1. **All trial treatments should commence as soon as practically possible after randomisation.** Patients having a bilateral orchidectomy should commence any additional treatment with 4 weeks of the operation unless there is a strong clinical reason not to do so.

6.1.1 Arm A (Control arm) - Androgen Suppression Only

The recommended methods of AS are bilateral orchidectomy or LHRH analogues (see below). Please note that anti-androgens alone are not permissible as hormone therapy for patients participating in STAMPEDE, but their use is recommended in the short-term to prevent tumour "flare" which may occur after commencing LHRH analogues. At the time of randomisation centres will be asked to specify the method of AS for each patient. Other methods of AS should be discussed with the Chief Investigator or the Trial Physician.

- <u>Bilateral orchidectomy</u> Operations should be performed by appropriately trained surgeons. A total or subcapsular orchidectomy may be performed.
- <u>LHRH agonists</u> LHRH analogues used according to local practice. The prophylactic use of anti-androgens to prevent tumour "flare" is recommended.

6.1.2 Arm B - Androgen Suppression + Zoledronic Acid

- Androgen suppression as described in section 6.1.1.
- Zoledronic Acid 4mg 15min IV infusion every 3 weeks, for 6 treatments followed by zoledronic acid 4mg 15min IV infusion every 4 weeks up to a maximum of 2 years from the start of the treatment or until disease (including PSA) progression (see Section 7.2). Patients should also receive an oral supplement of 500mg calcium and 400IU vitamin D daily. These doses are available as a combination tablet.

6.1.3 Arm C - Androgen Suppression + Docetaxel

- Androgen suppression as described in section 6.1.1.
- Docetaxel 75mg/m^2 Day 1 as 1hr IV infusion, plus prednisolone 5mg *bid* daily for 21 days. The cycle should be repeated every 3 weeks for a maximum of 6 cycles. The recommended administration schedule, anti-emetic regimen and dose modifications for docetaxel are given in **Appendix F**.

6.1.4 Arm D - Androgen Suppression + Celecoxib

- Androgen suppression as described in section 6.1.1.
- Celecoxib 400mg *bid* until the sooner of 1 year or disease (including PSA) progression (see Section 7.2).

6.1.5 Arm E - Androgen Suppression + Zoledronic Acid + Docetaxel

- Androgen suppression as described in section 6.1.1.
- Zoledronic Acid 4mg 15min IV infusion every 3 weeks, for 6 treatments followed by zoledronic acid 4mg 15min IV infusion every 4 weeks up to a maximum of 2 years from the start of the treatment or until disease (including PSA) progression (see Section 7.2). Patients should also receive an oral supplement of 500mg calcium and 400IU vitamin D daily. These doses are available as a combination tablet.
- Docetaxel 75mg/m^2 Day 1 as 1hr IV infusion, plus prednisolone 5mg *bid* daily for 21 days. The cycle should be repeated every 3 weeks for a maximum of 6 cycles. The recommended administration schedule, anti-emetic regimen and dose modifications for docetaxel are given in **Appendix F**.
- Co-administration of docetaxel and zoledronic acid: Docetaxel 75mg/m^2 Day 1 as 1hr IV infusion, plus prednisolone 5mg *bid* daily followed by zoledronic Acid 4mg 15min IV infusion. There is evidence to suggest that the co-administration of docetaxel and zoledronic acid is sequence dependent (39). Consequently docetaxel should be administered before zoledronic acid

6.1.6 Arm F - Androgen Suppression + Zoledronic Acid + Celecoxib

- Androgen suppression as described in section 6.1.1.
- Zoledronic Acid 4mg 15min IV infusion every 3 weeks, for 6 treatments followed by zoledronic acid 4mg 15min IV infusion every 4 weeks up to a maximum of 2 years from the start of the treatment or until disease (including PSA) progression (see Section 7.2). Patients should also receive an oral supplement of 500mg calcium and 400IU vitamin D daily. These doses are available as a combination tablet.
- Celecoxib 400mg *bid* until the sooner of 1 year or disease (including PSA) progression (see Section 7.2).

6.2 Administration and Dose Modifications

6.2.1 Zoledronic Acid

Zoledronic acid will be administered by IV infusion in accordance with the instructions in the summary of product characteristics at a dose of 4mg every 3 weeks for the first 6 cycles, and thereafter every 4 weeks. There may be the opportunity for the 4-weekly infusions to be administered in the patient's home (**Appendix H**).

Serum Creatinine Measurements**:** Serum creatinine should be measured at baseline and within 48 hours prior to every administration of zoledronic acid.

Serum electrolytes and FBC**:** Serum electrolytes including calcium, phosphate and magnesium should also be measured prior to each infusion. FBC should be measured at least 3 monthly.

Zoledronic acid should be discontinued if there is any evidence of hypersensitivity to the drug. Dose reductions are not anticipated. For full details of zoledronic acid administration see **Appendix F.** Contraindications, special precautions, interactions and side effects are listed in **Appendix G.**

6.2.2 Docetaxel

The use of docetaxel should be confined to units specialised in the administration of cytotoxic chemotherapy and it should only be administered under the supervision of a physician qualified in the use of anticancer chemotherapy.

Docetaxel (Taxotere) will be administered by IV infusion in accordance with the instructions in the summary of product characteristics at a dose of 75mg/m^2 (up to a maximum dose of 160mg) on day 1 of the study treatment period and then every 3 weeks, thereafter, for a maximum of 6 doses. Patients with a body surface area (BSA) greater than 2.13m^2 should be dosed as though they have a BSA of 2.13m^2. No ideal weight should be used for BSA

calculations. Prednisolone 5mg *bid* will be given until completion of chemotherapy. Additional dexamethasone should be given pre- and post-docetaxel infusion to suppress allergic reactions. For full details of premedication schedule, recommended anti-emetic regimen and dose modifications for docetaxel see **Appendix F**. Contraindications, special precautions, interactions and side effects are listed in **Appendix G.**

Docetaxel in combination with prednisone or prednisolone is indicated for the treatment of patients with hormone refractory metastatic prostate cancer (22)

6.2.3 Celecoxib

Celecoxib should be administered in accordance with the instructions in the summary of product characteristics at a dose of 400mg *bid* orally. Rarely this drug is poorly tolerated and in this instance should be discontinued, particular care should be taken with patients with a history of gastrointestinal disease and patients with significant risk factors for cardiovascular events. (see **Appendix G**). Patients with confirmed cardiovascular history should not be in STAMPEDE (see exclusion criteria, section 4.2). Contraindications, special precautions, interactions and side effects are listed in **Appendix G.**. Dose reductions are not anticipated.

6.3 Trial products

Details of the procedures for obtaining the drugs within the trial, dispensing and disposal of unused drug are given in **Appendix E**.

Arrangements for free or discounted drugs are given in the Finance section (section 15).

6.4 Measures of compliance/adherence

Date of treatment, dose, delays and reasons for delays or dose modifications of all study infusions (zoledronic acid and docetaxel) will be recorded. The estimated number of celecoxib tablets taken in a given time period will also be recorded as well as any dose reductions.

6.5 Treatment data collection

Data will be recorded on case report forms (CRFs); the top copy/original should be sent to the MRC CTU for data entry and a copy kept at the local centre. The data to be recorded on these can be viewed in the CRF appendix (**Appendix O**). The type of data to be recorded is detailed in the Assessments and Procedures section (Section 7).

6.6 Non-trial treatment

6.6.1 Medications permitted

Any additional treatment that the responsible physician feels is appropriate is permitted.

6.6.2 Data on concomitant medication

All concomitant medication will be recorded on the baseline form prior to randomisation and on any subsequent Serious Adverse Event forms. This should include aspirin that may be taken on a regular basis for cardiovascular disease and the use of any Non-Steroidal Anti-inflammatory Drugs (NSAID).

7 ASSESSMENTS AND PROCEDURES

7.1 Flow chart/Schedule for follow-up

A detailed follow up schedule is given in **Figure 3 (see page 21)**.

7.1.1 PSA Measurements

All patients should have PSA measured pre-treatment (within two weeks of starting treatment) and at weeks 6, 12, 18 and 24 and every 12 weeks, thereafter, up to 2 years post randomisation. Following this, PSA should be measured every 3 months for the next two years and 6 monthly, thereafter. For patients who do not have a scheduled hospital visit, it would be acceptable for arrangements to be made for blood samples to be drawn either in a GP's surgery or in the patient's home.

7.1.2 Radiological Investigations

All patients should have baseline radiological examinations as detailed in section 4.3.1. In addition all patients should have scans or X-rays repeated at 24 weeks if they were abnormal at baseline, and whenever clinically appropriate.

7.2 Efficacy parameters

The primary outcome measure in Efficacy Stage I to III is failure-free survival. This will be defined as time from randomisation to the first of four possible events:

1. Biochemical failure (see **Appendix K**)
2. New Lesions (following modified RECIST criteria see **Appendix I**)
3. Increase in baseline lesions (following modified RECIST criteria see **Appendix I**)
4. Death from any cause.

<u>Biochemical Failure</u>: Patients who have a rising PSA and fulfil the criteria in **Appendix K**.

<u>Clinical Failure</u>: Patients with new lesions or lesions that have increased in size from baseline and fulfil the criteria in **Appendix I**

7.3 Follow-up

Every effort should be made to follow-up all patients who have been randomised. Patients should, if possible, remain under the care of an oncologist or urologist for the duration of the trial. If care of a patient is returned to the GP, it is the responsibility of the consultant who obtained the patient's consent to participate in the trial to ensure that the data collection forms are completed. If the patient moves from the local area, arrangements should be made for trial follow-up to be undertaken by their new local centre. Details of other participating centres can be obtained from the MRC CTU. The consent of patients should be obtained for their names to be flagged for survival information through national registries (e.g. ONS in England/Wales and GRO in Scotland). If the clinician moves, appropriate arrangements should be made to arrange for trial follow-up to continue at the centre.

7.4 Trial closure

For the purpose of complying with UK Clinical Regulations introduced on May 2004, the trial will be considered '*closed*' when the last patient has completed protocol treatment. However, further observational follow-up of all patients enrolled in the trial will continue until all randomised patients have died. This will initially be via the hospital, but in the longer term may employ national registers.

Figure 3 - Detailed schedule for completion of forms.

Timing of Assessment		Baseline		Treatment		Outcomes		Freq
		Randomisⁿ	Pre-Treatment	Zoledronic acid	Docetaxel	Follow-up $^{\Psi}$	QL + HE	
Yr 0	Wk 0	all	all				all	
	Wk 6			BEF †	CE †	all	all	6 weekly
	Wk 12			BEF †	CE †	all	all	
	Wk 18			BEF †	CE †	all	all	
	Wk 24			BEF †		all	all	
	Wk 36			BEF ‡		all	all	12 weekly
	Wk 48			BEF ‡		all	all	
	Wk 60			BEF ‡		all	all	
	Wk 72			BEF ‡		all	all	
	Wk 84			BEF ‡		all	all	
	Wk 96			BEF ‡		all	all	
Yr 2	Month 24			BEF ‡		all	all	3 monthly
	Month 27					all	all	
	Month 30					all	all	
	Month 33					all	all	
Yr 3	Month 36					all	all	
	Month 39					all	all	
	Month 42					all	all	
	Month 45					all	all	
Yr 4	Month 48					all	all	6 monthly
	Month 54					all	all	
Yr 5	Month 60					all	all	
	6 monthly…					all	all	

Key: all=arms A-F, A=AS, B=AS + Zoledronic acid, C=AS + Docetaxel, D=AS + Celecoxib, E=AS + Zoledronic acid + Docetaxel, F= Zoledronic acid + Celecoxib

$^{\Psi}$ Including treatment with hormone therapy and celecoxib and toxicity assessments

† Form records data for two cycles

‡ Form records data for three cycles

Progression, **Death** and **Withdrawal** forms to be completed as required

Note: an individualised form with a follow-up schedule will be provided for each randomised patient. For patients who are receiving LHRH analogues it is assumed that any additional treatment will commence within two weeks of randomisation. For patients who are due to have an orchidectomy it is recognised that surgery will have to be scheduled and the scheduling of any additional treatments may be affected by post-operative recovery. All patients who had abnormal radiological investigations at baseline should have them repeated 24 weeks after randomisation.

8 WITHDRAWAL OF PATIENTS

Patients should be given every encouragement to adhere to protocol treatment and follow-up, in order to reduce biases. However, a patient has the right to withdraw consent for participation in any aspect of this trial at any time. They may refuse to take certain treatments, attend scheduled follow-up visits, or move from the area (see section 8.3). Clear distinction must be made as to whether the patient is withdrawing from trial treatments/procedures whilst allowing further follow-up, or whether the patient refuses **any** further trial treatments/procedures **and** follow-up participation. In all instances the MRC-CTU must be informed immediately.

8.1 Withdrawal from trial interventions

A patient may withdraw, or be withdrawn, from trial treatment for the following reasons:

1. Progression whilst on therapy
2. Unacceptable toxicity
3. Intercurrent illness which prevents further treatment
4. Withdrawal of consent for treatment.
5. Any alteration in the patient's condition which justifies the discontinuation of treatment in the clinician's opinion.

The reason should be recorded on the treatment and/or follow-up forms as well as the withdrawal form.

Unless a patient states otherwise, it should be assumed that consent is given to continue to record trial data.

8.2 Withdrawal from the trial completely

If a patient explicitly withdraws consent to have **any** data recorded their decision must be respected and recorded on a withdrawal form. All communication surrounding the withdrawal should be noted in the patient's records and no further STAMPEDE CRFs should be completed for that patient.

Patients can change their minds about withdrawal at any time and re-consent to participate in the trial. Follow-up data should be collected **only** from the point of when consent was re-instated.

8.3 Patient transfers

For patients moving from the area, every effort should be made for the patient to be followed-up at another participating trial centre and for this trial centre to take over responsibility for the patient. A copy of the patient STAMPEDE CRFs will need to be provided to the new site. The patient will have to sign a new consent form at the new site, and until this occurs, the patient remains the responsibility of the original centre.

9 STATISTICAL CONSIDERATIONS

9.1 Method of Randomisation

Patients will be randomised centrally using a computerised algorithm developed and maintained by the MRC CTU. Randomisation will be performed using the method of minimisation over a number of clinically important stratification factors with an additional random element. To decrease determinability, the factors are not listed here. The trial has 1 control arm and 5 research arms. As the control arm is the comparator arm for all the research arms, it is intended to recruit twice as many patients to the control arm as to each research arm. Therefore, the randomisation ratio will be 2:1:1:1:1:1.

9.2 Outcome Measures

The overall primary outcome measure for the trial is overall survival (all cause mortality). The design of the trial is such that it is important to have additional intermediate outcome measures to assess each research arm as the trial progresses. These are listed in Table 1. The reasons for different emphases in each recruitment stage is explained in section 9.3.

Table 1: Trial Outcome Measures

Trials stage	Primary outcome measures	Secondary outcome measures
**Pilot phase**	Safety*	Feasibility
**Efficacy Stage I-III**	Failure-free survival (FFS)†	Overall survival (OS)<br>Toxicity<br>Skeletal related events
**Efficacy Stage IV**	Overall survival	Quality of life<br>Cost effectiveness<br>Failure-free survival†<br>Toxicity<br>Skeletal related events

*Based on toxicity

†Including biochemical failure (see **Appendix K**)

9.3 Sample Size

9.3.1 Overall sample size

The overall design for this study is a six arm, multi-centre randomised controlled trial (see Figure 4, page 26). There are fives stages to the study, a Pilot Phase, Efficacy Stages I-IV. Full details of the methodology underlying the trial design are given by Royston *et al*. (40). The sample size calculations were performed using the -stage2- program (version 1.2.0, March 2002) (41) and -stagen- program (version 1.1.1, 18 May 2004), both implemented in Stata 8 (Stata Corp, TX). The trial is designed under the assumptions in Table 2, and additionally, we assume a slightly higher proportion of non-metastatic than metastatic patients such that the median FFS is two years and median OS four years.

Table 2: Hazard ratio assumptions under null and alternative hypotheses

Size of HR	Pilot	Stage I-III	Stage IV
Under null hypothesis (H_0)	n/a	HR(FFS) = 1.0	HR(OS) = 1.0
Under alternative hypothesis (H_1)	n/a	HR(FFS) = 0.75	HR(OS) = 0.75

The HR of 0.75 for any research arm relative to control would translate into an absolute improvement in FFS of 10%, from approximately 50% to 60% at two years and OS of 10%, from approximately 50% to 60% at four years. A beneficial difference of this size would be clinically worthwhile and, indeed, experience tells us it may be unrealistic to expect a larger difference. Therefore, we have adequately powered the trial to detect a HR of 0.75 for overall survival. This design gives 95% power at Efficacy Stages I-III and 90% power at Efficacy Stage IV. Further details of the sample size calculations are given in a separate Statistical Design document which is available on request.

Assuming an accrual rate of 500 patients/year then between 2800 and 3600 patients are planned to be entered into the trial over a period of 5½ and 7 years. The exact number of patients to be entered depends on the observed accrual and event rates. The primary analysis on overall survival requires 445 deaths to be observed on the control arm.

9.3.2 Pilot Phase

It is anticipated that 210 patients will be recruited to the Pilot Phase from a limited number of centres over a one year period. Approximately 60 patients will be randomised to the control arm and 30 patients to each of the five research arms each of which will be assessed for safety and feasibility. If recruitment proves infeasible or any of the research arms prove unsafe or not feasible to administer (e.g. poorly tolerated or unexpected toxicity) recruitment to these arms will be discontinued. There are already considerable safety data on the use of docetaxel and zoledronic acid in patients with malignancies including prostate cancer, and on the use of Cox-2

inhibitors (including celecoxib), although mainly from patients with musculoskeletal disorders. There are fewer data on the combination arms; but it is thought very unlikely that any of the research arms will be discontinued during the Pilot Phase. Safety data will continue to be assessed throughout the trial. When 210 patients have been on the trial for a minimum of 18 weeks, the independent Data Monitoring Committee (IDMC) will then review the data from the Pilot phase. Recruitment will continue to the trial during this period as equipoise will remain.

9.3.3 Efficacy Stages I-III

In the sample size calculations we assume that all research arms successfully pass through the Pilot Phase to Efficacy Stage I and that patients will be recruited at a rate of approximately 500 per year. This is faster than in the Pilot Phase because the trial will recruit from additional centres, both in the UK and internationally. The analysis of Efficacy Stages I, II and III are planned with 115, 225 and 355 failure-free survival events have been observed in the control arm, respectively.

The Efficacy Stage analyses will comprise pairwise comparisons of FFS between the control arm and each of the 5 research arms (i=1 to 5). Let HR_i(true) represent the hazard ratio (HR) of the ith research arm to the control arm, and HR_i(observed) the observed value. Discontinuation of accrual of further patients will be considered for the ith research regimen at each of Efficacy Stages I-III according to the guidelines in Table 3.

Table 3: Guidelines for stopping accrual to the ith research arm

Efficacy Stage	Number of control arm events	Consider discontinuation if HR_i(observed) is...
I	115	>1.00
II	225	>0.92
III	355	>0.89

9.3.4 Efficacy Stage IV

The analysis of Efficacy Stage IV will be performed when 445 deaths have been observed in the control arm. This would give 90% power to detect the targeted hazard ratio of 0.75 at one-sided significance level of 0.025. The actual length of this stage and the number of patients to be recruited depends on the number of arms passing through to further recruitment from Efficacy Stages I-III and the observed accrual and event rates.

Figure 4: Progress of STAMPEDE through the trial stages

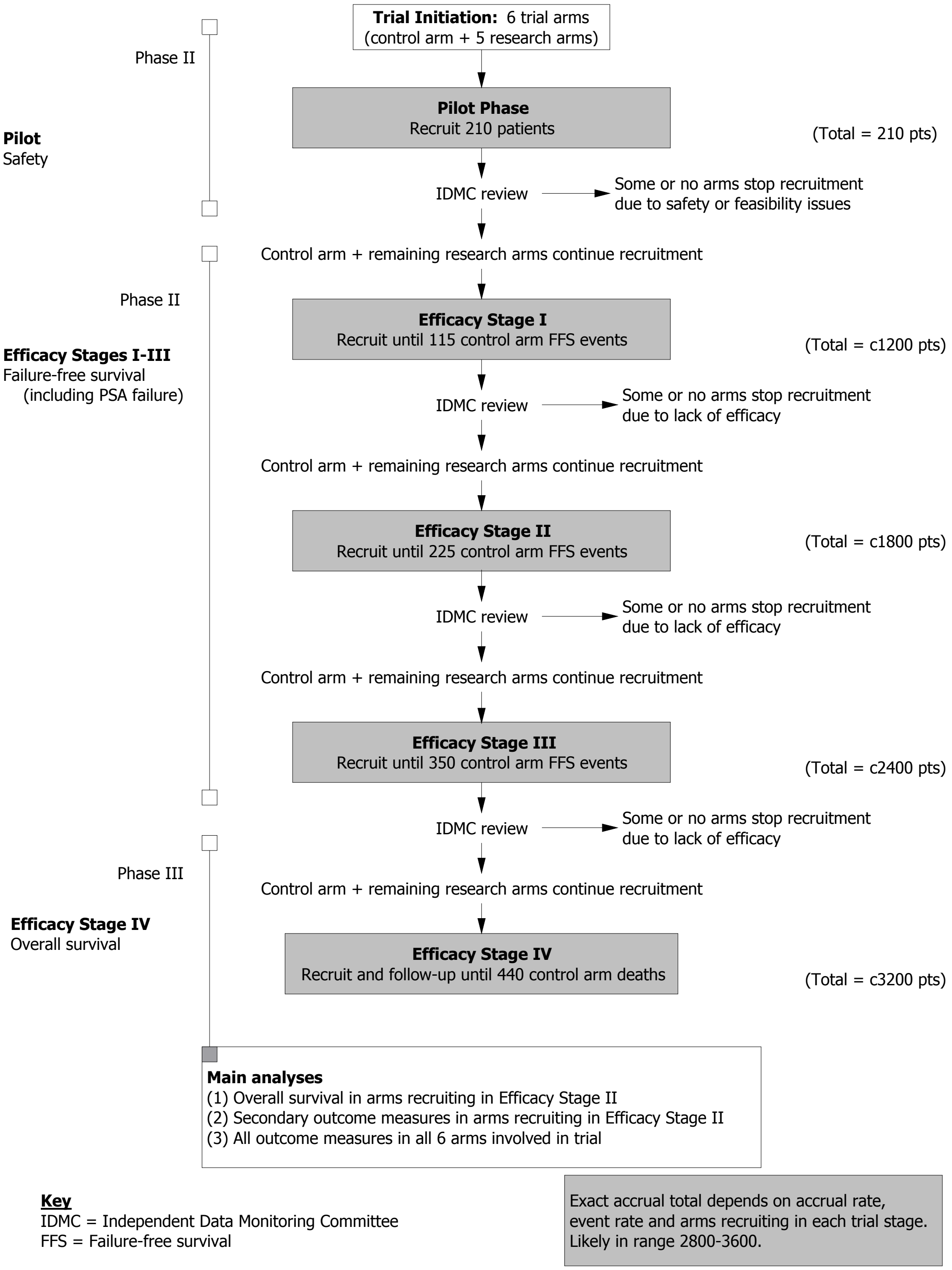

9.3.5 Factorial design

We note here that we have not employed a factorial design in this trial because we anticipate the possibility of synergy between hormone therapy, zoledronic acid and docetaxel and between hormone therapy, zoledronic acid and celecoxib. It would not be possible to assess any such interactions reliably in a factorial trial (see the Statistical Design document for further details).

9.4 Interim Monitoring and Analyses

Formal interim analyses of the accumulating data will be performed at regular intervals (approximately annually) for review by an Independent Data Monitoring Committee (IDMC) (see also section 16). These analyses will be performed by the trial team at the MRC CTU. The IDMC will be asked to give advice on whether the accumulating data from the trial with the guidelines for discontinuation of accrual for Efficacy Stages I-III, together with results from any other relevant trials, justifies continuing recruitment of further patients or further follow-up. A decision to discontinue recruitment, in all patients or in selected subgroups will be made only if the result is likely to convince a broad range of clinicians including those entering patients into the trial and the general clinical community. If a decision is made to continue, the IDMC will advise on the frequency of future reviews of the data on the basis of accrual and event rates. The IDMC will make recommendations to the Trial Steering Committee (TSC, see section 16) as to whether the trial should continue in its present form. While the trial is ongoing the accumulating data will remain confidential.

9.5 Outline Analysis Plan

Analyses will be performed on an intention-to-treat basis. The standard unadjusted log-rank approach will be applied to analyses of FFS and OS. The impact of potential confounders including the stratification factors used at randomisation will be considered in a Cox proportional hazard model. The χ^2 test or Mann-Whitney test will be implemented for categorical data comparisons, including toxicity, as appropriate. The primary outcome measures in Table 1 (see page 25) will be considered for all arms of the trial at each phase, but the main emphasis will be placed on the comparison of the research arms that have continued to recruit throughout the trial.

9.5.1 Pilot Phase

The Pilot Phase randomises patients between all the study arms so that the results from these patients can be included in the main trial. Feasibility is considered in terms of the acceptability of the trial randomisation and reported toxicities and adherence to trial medication. Centres participating in the Pilot Phase will be required to keep an anonymised log of all patients

assessed for trial eligibility. We will then summarise the number of patients who do not participate in the study, and also the number of eligible patients who choose to not participate in the study (reasons for non-participation will be collected where the patients are willing). On the patients who are randomised, we shall describe the incidence of expected and unexpected severe toxicities and adverse events/reactions (see Section 11) to decide whether to continue with research arms beyond the Pilot Phase. As indicated above, we do not anticipate that recruitment to the research arms will be discontinued after the Pilot Phase, as there is considerable experience with zoledronic acid and docetaxel when combined with AS, while Cox-2 inhibitors generally have a good toxicity profile. Although there are limited data on the combinations, we do not expect severe toxicity.

9.5.2 Efficacy Stages

The approach to analysis of these stages is summarised within the sample size calculations (see Section 9.3.3). Each research arm will be compared in a pairwise fashion against the control arm.

10 DATA VERIFICATION AND SITE MONITORING

10.1 Verification of data at MRC CTU

Data stored at the MRC CTU will be checked for missing or unusual values (range checks) and checked for consistency over time. If any such problems are identified, a photocopy of the problematic CRF(s) will be returned to the local site by post or fax for correction. The incorrect data should be crossed through with a single line in black biro. The correct data should be written next to the amendment in black biro and initialled and dated by the investigator, in accordance with GCP. The amended version should be returned to MRC CTU and the site’s copy should also be amended. MRC CTU will send reminders for any overdue data.

10.2 Clinical site monitoring

10.2.1 Direct Access to Data

Participating investigators should agree to allow trial-related monitoring, including audits, ethics committee review and regulatory inspections by providing direct access to source data/documents as required. Patients’ consent for this must be obtained.

10.2.2 Quality Assurance and Quality Control of Data

A sample of 10% of patients will be selected for source data verification throughout the trial. This may include random sampling, but may also include patients who might exhibit a reason for the data to be checked in this way.

11 SAFETY REPORTING

11.1 Adverse events and grading of adverse events

Adverse events will be graded using the Common Toxicity Criteria (CTC) version 3.0. The complete CTCv3 can be found at http://ctep.cancer.gov/reporting/ctc.html, the most common adverse events for the drugs being used in STAMPEDE are given in **Appendix J.** The CTC grades need to be recorded on the CTC toxicity part of the treatment form (see **Appendix O**). All patients experiencing a SAE or an adverse event should be followed up as outlined in the protocol. For patients who decide to withdraw from the trial treatment after an adverse event the procedure outlined in Section 8 should be followed.

Previously reported (and, therefore, expected) reactions from zoledronic acid, docetaxel and celecoxib are listed in **Appendix G**.

The Medicines for Human Use (Clinical Trials) Regulations 2004 provides the definitions given in table 4.

Table 4: Terms and definitions for adverse events

Term	Definition
**Adverse Event (AE)**	Any untoward medical occurrence in a subject to whom a medicinal product has been administered including occurrences which are not necessarily caused by or related to that product.
**Adverse Reaction (AR)**	Any untoward and unintended response in a subject to an investigational medicinal product, which is related to any dose administered to that subject.
**Unexpected Adverse Reaction (UAR)**	An adverse reaction the nature and severity of which is not consistent with the information about the medicinal product in question set out in:<br>• The SPC for that product (for products with a marketing authorisation)<br>• The Investigator's Brochure (IB) relating to the trial in question (for any other investigational product)
**Serious Adverse Event (SAE)**<br>**Serious Adverse Reaction (SAR)**<br>**Suspected Unexpected Serious Adverse Reaction (SUSAR)**	Respectively, any adverse event, adverse reaction or unexpected adverse reaction that:<br>• results in death<br>• is life-threatening*<br>• requires hospitalisation or prolongation of existing hospitalisation**<br>• results in persistent or significant disability or incapacity<br>• consists of a congenital anomaly or birth defect<br>• other important medical event(s)***

* The term 'life-threatening' in the definition of 'serious' refers to an event in which the patient was at risk of death at the time of the event; it does not refer to an event which hypothetically might have caused death if it were more severe.

** Hospitalisation is defined as an inpatient admission, regardless of length of stay, even if the hospitalisation is a precautionary measure for continued observation. Hospitalisations for a pre-existing condition, including elective procedures that have not worsened, do not constitute an SAE.

*** Other events that may not result in death, are not life threatening, or do not require hospitalisation may be considered a serious adverse experience when, based upon appropriate medical judgement, the event may jeopardise the patient and may require medical or surgical intervention to prevent one of the outcomes listed above (excluding new cancers or result of overdose).

11.2 Institution Responsibilities

Please see **Appendix P** for flow chart of adverse event reporting.

All SAEs must be reported immediately by the investigator to the MRC CTU on an SAE form unless the SAE is specified in the protocol (see below and **Appendix G**) as not requiring immediate reporting. All other adverse events should be reported on the regular progress/follow-up reports.

Please note:

a. Trial events/outcome measures such as disease progression or death as a result of disease progression are not considered to be SAEs and should be reported on the follow-up form.
b. Certain conditions defined as SAEs may be excluded from expedited reporting on an SAE form, i.e.:
    i. Elective hospitalisation and surgery for treatment of disease or its complications.
    ii. Elective hospitalisation to simplify treatment or procedures.

Procedures

1. The SAE form should be completed by the responsible investigator (consultant named on the signature list and delegation of responsibilities log who is responsible for the patient's care). The investigator should assess the SAE for the likelihood that that it is a response to an investigational medicine. In the absence of the responsible investigator the form should be completed and signed by a member of the site trial team and faxed to the MRC CTU immediately. The responsible investigator should check the SAE form, make changes as

appropriate, sign and then re-fax to the MRC CTU as soon as possible. The initial report shall be followed by detailed, written reports.

2. Send the SAE form by fax (within 24 hours or next working day) to the MRC CTU

Fax Number: + 44 (0)20 7670 4818

3. Staff at the institution must **notify** their local ethics committee (LREC) of the event (as per standard local procedure).

4. Follow-up of SAEs: In the case of an SAE the subject must be followed-up until clinical recovery is complete and laboratory results have returned to normal, or until the event has stabilised. Follow-up may continue after completion of protocol treatment if necessary. Follow-up information is noted on another SAE form by ticking the box marked 'follow-up' and faxing to the MRC CTU as information becomes available. Extra, annotated information and/or copies of test results may be provided separately. The patient **must** be identified by trial number, date of birth and initials only. The patient's name **should not** be used on any correspondence.

11.3 MRC CTU Responsibilities

The MRC CTU is undertaking the duties of trial sponsor and is responsible for the reporting of SUSARs and other SARs to the regulatory authorities (MHRA, competent authorities of other European member states in which the trial is taking place and, if required, the research ethics committees) as follows:

- SUSARs which are fatal or life-threatening must be reported not later than 7 days after the MRC CTU is first aware of the reaction. Any additional relevant information must be reported within a further 8 days.
- SUSARs that are not fatal or life-threatening must be reported within 15 days of the MRC CTU first becoming aware of the reaction.
- A list of all SARs (expected and unexpected) must be reported annually.

Medically qualified staff at the MRC CTU and the Chief Investigator (or a delegate) will evaluate all SAEs received for seriousness, expectedness and causality. Investigator reports of suspected SARs will be reviewed immediately and those that are SUSARs identified and reported to regulatory authorities. The causality assessment given by the local Investigator at the hospital cannot be overruled and in the case of disagreement, both opinions will be provided with the report.

The MRC CTU will inform the MREC in the UK of all SUSARs on an annual basis. The MRC CTU will send the reports directly to international central ethics committees unless there is a local co-ordinating centre, which will assume this responsibility. The MRC CTU will also send an annual safety report containing a list of all SARs to MREC (and all international centralised ethics committees). A copy of the report will be sent to the Principal Investigator at all institutions participating in the trial for their information.

Table 5: Adverse events – some inclusions and exclusions

Adverse events include	Adverse events do not include
a) an exacerbation of a pre-existing illness<br>b) an increase in frequency or intensity of a pre-existing episodic event/condition<br>c) a condition (even though it may have been present prior to the start of the trial) detected after trial drug administration<br>d) continuous persistent disease or symptoms present at baseline that worsens following the administration of the study/trial treatment	a) medical or surgical procedures- the condition which leads to the procedure is the adverse event<br>b) pre-existing disease or conditions present before treatment that do not worsen<br>c) situations where an untoward medical occurrence has occurred e.g. cosmetic elective surgery<br>d) overdose of medication without signs or symptoms<br>e) the disease being treated or associated symptoms/signs unless more severe than expected for the patient's condition

11.4 Severity/grading of adverse events

Adverse events will be graded using the NCI Common Toxicity Criteria (CTC) Version 3.0, an abridged version is given in **Appendix J**.

11.5 Relationship to trial treatment

The relationship of an adverse event to study medication should be graded as definite, probable, possible, (unlikely) or not related. For SAEs this causality assessment should be noted on the SAE form.

11.6 Follow-up after adverse events

It is up to the discretion of the treating clinician as to whether trial participants should continue with protocol treatment following an adverse or serious adverse event subject to the dose modification schedules in **Appendix F** Treatment delays and dose modifications should be detailed on the requisite treatment form.

12 ETHICAL CONSIDERATIONS AND APPROVAL

12.1 Ethical considerations

This is a randomised trial therefore neither the patients nor their physicians will be able to choose the patients' treatment. Treatment will be allocated randomly using a computer-based algorithm. This is to ensure that the groups of patients receiving each of the different treatments are similar.

Five sevenths of the patients will receive one or two of the newer treatments in combination with hormone treatment; hormone treatment alone is the standard treatment for these forms of prostate cancer. These newer combined treatment options are being assessed in a detailed and systematic fashion in this trial. There is some evidence to suggest that the newer treatment options *may* have advantages over standard treatment (hormone therapy) alone with regards clinical outcome, but this is not confirmed and toxicity may be increased. This trial will follow a large group of men who have been randomly allocated to either the standard treatment (hormone therapy alone) or the newer combined treatment options in order to measure the benefits of the new treatments. The patients will also be followed-up for toxicity and safety issues, so that any benefits can be weighed against any negative aspects.

Patients participating in the trial will have some additional hospital visits and some extra blood samples taken compared to patients who are not participating in the trial. Sometimes the blood samples can be taken when the patient is attending hospital for treatment, anyway. On some of the trial arms, the patient may have to make additional visits to the hospital for the blood sample to be taken, although in some cases it may be possible for the blood sample to be taken in the GP's surgery or in the patients home. The additional visits and blood samples are to ensure that follow-up of patients is comparable in all the treatment groups. The blood samples will also be used for genetic and serum marker studies, where this information will be considered with clinical data. Blood samples will be link-anonamysed. There will be no feedback to individual patients.

12.2 Ethical approval

The protocol has a Favourable Opinion from a Main Research Ethics Committee (MREC), but each site must also gain a favourable Site Specific Assessment (SSA) before patients can be entered into the trial. The patient's informed consent to participate in the trial should be obtained after a full explanation has been given of the treatment options, including the conventional and generally accepted methods of treatment. Suggested patient information sheets and patient consent forms are given in **Appendix B**.

The right of the patient to refuse to participate in the trial without giving reasons must be respected. After the patient has entered the trial, the clinician must remain free to give alternative treatment to that specified in the protocol, at any stage, if he feels it to be in the best interest of the patient. However, the reason for doing so should be recorded and the patient will remain within the trial for the purpose of follow-up and data analysis according to the treatment option to which he has been allocated. Similarly, the patient must remain free to withdraw at any time from the protocol treatment without giving reasons and without prejudicing his further treatment.

A statement of MRC policy on ethical considerations in clinical trials of cancer therapy, including the question of informed consent, is available from the MRC Head Office web site (http://www.mrc.ac.uk).

13 REGULATORY APPROVAL

This trial has been approved by the MHRA and will be conducted under a CTA in the UK.

14 INDEMNITY

The MRC and NHS are both publicly funded bodies and are not allowed to purchase advance insurance to cover indemnity because they are backed by the resources of the Treasury.

The MRC will give sympathetic consideration to claims for non-negligent harm suffered by a person as a result of trial or other work supported by MRC. This does not extend to liability for non-negligent harm arising from conventional treatment where this is one arm of a trial. The MRC acts as its own insurer and does not provide cover for non-negligent harm in advance for participants in MRC-funded studies.

Where studies are carried out in a hospital, the hospital continues to have a duty of care to a patient being treated within the hospital, whether or not the patient is participating in an MRC-supported study. MRC does not accept liability for any breach in the hospital's duty of care, or any negligence on the part of employees of hospitals. This applies whether the hospital is a NHS Trust or not.

15 FINANCE

STAMPEDE is funded by the Clinical Trials Advisory Awards Committee (CTAAC) (on behalf of Cancer Research UK, Medical Research Council, and other charities). The trial has National

Cancer Research Network (NCRN) approval and, therefore, local NCRN funds may be available at each centre to support entry of patients into this trial.

Zoledronic acid is manufactured by Novartis. Novartis have agreed to provide an educational grant to support the conduct of this study. Novartis have also agreed to supply the study drug, zoledronic acid free of charge for patients participating in the study.

Docetaxel is manufactured by Aventis Pharma. They have agreed to supply the study drug, docetaxel at a discounted rate for patients that are participating in the trial and to provide an educational grant to support the conduct of the study. The Department of Health has agreed to provide a central subvention as follow: £1,787 per patient randomised to arms C and E of the trial and prescribed docetaxel. This amount is payable in respect of a hospital trust randomising more than 3 patients. For more details contact the STAMPEDE Trial Manager.

Celecoxib is manufactured by Pfizer. They have agreed to supply free drug and provide funds to distribute drug to participating sites.

16 TRIAL COMMITTEES

16.1 Trial Management Group (TMG)

A Trial Management Group (TMG) has been formed comprising the Chief Investigator, other co-investigators and members of the MRC CTU. The membership of the TMG may be expanded if other groups of trialists wish to participate. The TMG will be responsible for the day-to-day running and management of the trial and will meet by teleconference at least 3 monthly and in person as needed. The TMG members are detailed in **Appendix L**.

16.2 Trial Steering Committee (TSC)

A Trial Steering Committee (TSC) will be formed to provide overall supervision for the trial and provide advice through its independent chair. The ultimate decision for the continuation of the trial lies with the TSC. The TSC will meet twice a year.

16.3 Independent Data Monitoring Committee (IDMC)

An Independent Data Monitoring Committee (IDMC) will be formed. The IDMC will be the only group who sees the confidential, accumulating data to the trial. Reports to the IDMC will be produced by the MRC CTU. The IDMC will meet within 6 months of the trial opening with the frequency of meetings dictated by the IDMC. The IDMC will consider data in accordance with the analysis plan (see section 9.5) and will be advisory to the TSC. The IDMC can recommend

premature closure or reporting of the trial, or that recruitment to any research arm be discontinued.

Further details of IDMC functioning, and the procedures for interim analysis and monitoring are provided in the IDMC charter (available on resquest).

Figure 5 - Diagram of relationships between trial committees

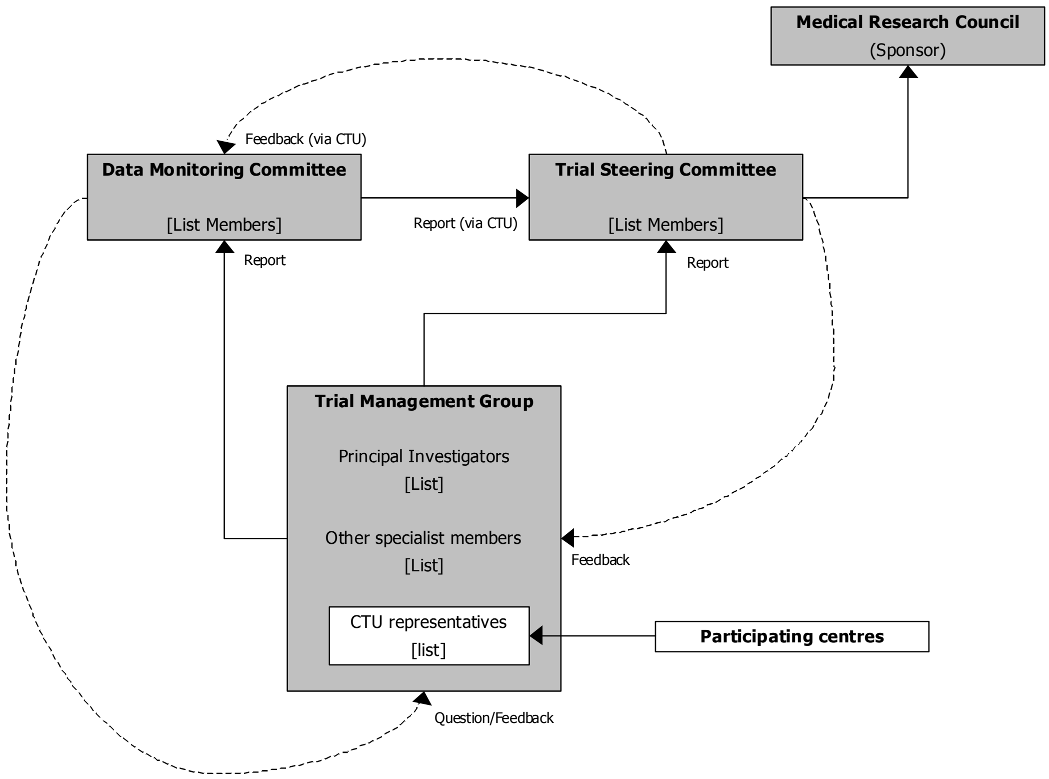

17 ANCILLARY STUDIES

17.1 Quality of LIfe

A quality of life (QL) study is being performed to assess the impact of each treatment arm on the quality of patient's lives and all patients should be asked if they are willing to participate. The EORTC QLQ-C30 with the prostate-specific module QLQ PR25 will be used. Key items for assessment are pain reduction for patients with metastatic disease and urinary symptoms for patients with locally advanced disease. In addition specific hypotheses will be generated for each of the research arms. The EuroQol (EQ-5D) (42) will be used in the study as a generic measure of health-related quality of life which can be linked to public preferences. These data will be used to calculate quality-adjusted life-years as part of the economic evaluation (see Section 17.2).

A patient information sheet about the QL study is given in **Appendix B** and the QL questionnaires are given in **Appendix N**. The first questionnaires should be administered within three days prior to, or on the day of randomisation, but in either case before the patient is informed which trial treatment he has been assigned. The subsequent questionnaires will be administered during the treatment and during the follow-up period. Questionnaires should be self-administered, although it is recommended that a key person (e.g. research nurse) at each centre be responsible for the data collection to optimise compliance and completeness of the data.

The QL and the HE questionnaires should be completed without conferring with friends or relatives and all questions should be answered even if the patient feels them to be irrelevant.

The responsible person should check each questionnaire for its completeness, ensuring that the correct date of completion and patient identifiers are present. The research nurse should approach patients at appropriate clinical visits to complete a questionnaire. If no clinical visit is scheduled for the patient (with a window of 4 weeks around the expected date) the nurse should organise the completion of the questionnaire, by post or by a visit to the patient at home (or in a hospice).

17.2 Health Economics

A health economics (HE) sub-study will be performed. Core resource use information will be collected, using CRFs on days in hospital (by speciality) and outpatient visits. Data being collected on concomitant medication will also be used in the economic analysis. Information on patients' use of primary care and community-based services will be collected as additional questions in the QL questionnaire. Costs will be calculated on the basis of representative UK unit

costs at the point of analysis. Health outcomes will be assessed in terms of quality-adjusted life years (QALYs). Quality adjustments will be based on patients' responses to the EQ-5D health status measure which will be administered at baseline and each point of follow-up as part of the QL questionnaire (**Appendix N**). A cost-effectiveness analysis will compare all regimens in Efficacy Stage II.

17.3 Molecular genetics

A molecular genetics sub-study is being performed. With the patient's consent, an additional 8ml blood sample will be collected and stored for either DNA and protein analysis in order to try to identify genes that control response to therapy and its side effects, and susceptibility to prostate cancer.

Blood samples should be sent directly to the central laboratory in the packs provided. Patient information sheets and consent forms **Appendix B**, while details of specimen collection, posting and contact details are given in **Appendix D**.

18 PUBLICATION

The results from different centres will be analysed together and published as soon as possible. Individual clinicians must not publish data concerning their patients that are directly relevant to questions posed by the study until the TMG has published its report. The TMG together with the STAMPEDE collaborators will form the basis of the writing committee and decide on the nature of publications. The main publication will be in the name of the STAMPEDE Trial Collaborators and the writing committee will be listed at the end of the manuscript. All publications will acknowledge the participating centres and clinicians, and these will be detailed in an appendix to the main report.

19 PROTOCOL AMENDMENTS

Amendments made to sections in Protocol version 1.0 May 2004

1. Administrative changes such as typos, word change etc.
2. Name additions/changes to:
   - TMG members
   - TSC members
   - IDMC members
3. 'General Information' Section – additional information re. Abridged version of protocol
4. Section 1.2 – Figure 1, Celecoxib duration amended
5. Section 1.3 – Figure 2, addition of cardiovascular assessment form, name and timings amended
6. Section 2.3 – Docetaxel information updated
7. Section 2.4 – Additional text re. dose and duration justification for Celecoxib use.
8. Section 3 – Title change and content updated
9. Section 4.2 – New exclusion criteria added
10. Section 4.3.1 – New investigations added and additional text re. testosterone measurements and additional text re. prior celecoxib treatment
11. Section 6.1.4 – Celecoxib duration amended
12. Section 6.1.5 – Additional text re. Co-administration of docetaxel and bisphosphonates
13. Section 6.1.6 – Celecoxib duration amended
14. Section 6.2.2 – additional docetaxel information
15. Section 6.2.3 – addition of CV event history
16. Section 11 – Safety reporting updated
17. Section 12.1 – Additional text re. the collection of blood for genetic and serum marker studies
18. Section 15 – Additional information re. Central Subvention for docetaxel arms

Amendments made to Appendices V1.0 May 2004.

1. Appendix A – Addition of NYHA classifications
2. Appendix B General PIS - Docetaxel information updated, additional information explaining cardiovascular risk related to celecoxib and celecoxib duration amended
3. Appendix B PIS C – Docetaxel information updated
4. Appendix B PIS D – Additional information explaining cardiovascular risk related to celecoxib and celecoxib duration amended
5. Appendix B PIS E – Docetaxel information updated

6. Appendix B PIS F – Additional information explaining cardiovascular risk related to celecoxib and celecoxib duration amended
7. Appendix C GP letter – product name changed from Celebrex to Onsenal
8. Appendix E – Drug supply/ordering procedures amended
9. Appendix F 'Administration of Zoledronic acid' – updated dose modification section
10. Appendix F – Additional section added 'Co-administration of docetaxel + zoledronic acid'
11. Appendix G Celecoxib – updated from revised celecoxib SPC
12. Appendix G: Table G.2 - Comparative table of undesirable effects of docetaxel, Zoledronic Acid and Celecoxib – updated information from revised celecoxib SPC
13. Appendix J: Common Toxicity Criteria – additional clarification and change in table structure
14. Appendix M: Accreditation documents – addition of new documents
15. Appendix P – Assessing and notifying CTU of adverse events flow diagram added

20 REFERENCES

1. ww.cancerresearchuk.org/aboutcancer/statistics/statstables/prostatecancer . 2005.

2. Goktas S, Crawford ED. Optimal hormonal therapy for advanced prostatic carcinoma. Semin.Oncol 1999;26(2):162-73.

3. Catalona WJ. Management of cancer of the prostate. N Engl J Med 1994;331(15):996-1004.

4. Scher HI, Kelly WK. Flutamide withdrawal syndrome: its impact on clinical trials in hormone-refractory prostate cancer. J Clin Oncol 1993;11(8):1566-72.

5. Vogelzang NJ. One hundred thirteen men with hormone-refractory prostate cancer died today. J Clin Oncol 1996;14(6):1753-5.

6. Posadas EM, Dahut WL, Gulley J. The emerging role of bisphosphonates in prostate cancer. Am.J Ther. 2004;11(1):60-73.

7. Morris MJ, Scher HI. Optimizing targeted therapy and developing novel outcome measures for patients with advanced prostate cancer at Memorial Sloan-Kettering Cancer Center. Crit Rev.Oncol Hematol. 2003;46 Suppl:S21-S31.

8. Riccardi A, Grasso D, Danova M. Bisphosphonates in oncology: physiopathologic bases and clinical activity. Tumori 2003;89(3):223-36.

9. Green JR, Muller K, Jaeggi KA. Preclinical pharmacology of CGP 42'446, a new, potent, heterocyclic bisphosphonate compound. J.Bone Miner.Res. 1994;9(5):745-51.

10. Major P, Lortholary A, Hon J, Abdi E, Mills G, Menssen HD et al. Zoledronic acid is superior to pamidronate in the treatment of hypercalcemia of malignancy: a pooled analysis of two randomized, controlled clinical trials. J Clin Oncol 2001;19(2):558-67.

11. Santini D, Vesasiani Gentilucci A, and Vincenzi A. The antineoplastic role of bisphosphonates: from basic research to clinical evidence. Annals of Oncology 14, 1468-1476. 2003.

12. Rosen LS, Gordon D, Kaminski M, Howell A, Belch A, Mackey J et al. Long-term efficacy and safety of zoledronic acid compared with pamidronate disodium in the treatment of skeletal complications in patients with advanced multiple myeloma or breast carcinoma: a randomized, double-blind, multicenter, comparative trial. Cancer 2003;98(8):1735-44.

13. Saad F, Gleason DM, Murray R, Tchekmedyian S, Venner P, Lacombe L et al. A randomized, placebo-controlled trial of zoledronic acid in patients with hormone-refractory metastatic prostate carcinoma. J Natl.Cancer Inst. 2002;94(19):1458-68.

14. Dearnaley DP, Sydes MR, Mason MD, Stott M, Powell CS, Robinson AC et al. A double-blind, placebo-controlled, randomized trial of oral sodium clodronate for metastatic prostate cancer (MRC PR05 Trial). J Natl.Cancer Inst. 2003;95(17):1300-11.

15. Khan MA, Carducci MA, Partin AW. The evolving role of docetaxel in the management of androgen independent prostate cancer. J Urol. 2003;170(5):1709-16.

16. Petrylak DP, Macarthur RB, O'Connor J, Shelton G, Judge T, Balog J et al. Phase I trial of docetaxel with estramustine in androgen-independent prostate cancer. J Clin Oncol 1999;17(3):958-67.

17. Picus J, Schultz M. Docetaxel (Taxotere) as monotherapy in the treatment of hormone-refractory prostate cancer: preliminary results. Semin.Oncol 1999;26(5 Suppl 17):14-8.

18. Beer TM, El Geneidi M, Eilers KM. Docetaxel (taxotere) in the treatment of prostate cancer. Expert.Rev.Anticancer Ther. 2003;3(3):261-8.

19. Kreis W, Budman DR, Fetten J, Gonzales AL, Barile B, Vinciguerra V. Phase I trial of the combination of daily estramustine phosphate and intermittent docetaxel in patients with metastatic hormone refractory prostate carcinoma. Ann.Oncol 1999;10(1):33-8.

20. Piccart MJ, Klijn J, Paridaens R, Nooij M, Mauriac L, Coleman R et al. Corticosteroids significantly delay the onset of docetaxel-induced fluid retention: final results of a randomized study of the European Organization for Research and Treatment of Cancer Investigational Drug Branch for Breast Cancer. J Clin Oncol 1997;15(9):3149-55.

21. Oudard, S., Beuzeboc, P., Dourthe, L. M., and et al. Preliminary results of a phase II randomized trial of docetaxel (D), estramustine (E) and prednisolone (P) - two schedules - versus mitoxantrone (M) and prednisone in patients with Hormone Refractory Prostate Cancer (HRPC). Proc Am Soc Clin Oncol 21, 117a-(Abstract No. 706). 2002.

22. Petrylak DP, Tangen CM, Hussain MH, Lara PN, Jr., Jones JA, Taplin ME et al. Docetaxel and estramustine compared with mitoxantrone and prednisone for advanced refractory prostate cancer. N Engl J Med 2004;351(15):1513-20.

23. Tannock IF, de Wit R, Berry WR, Horti J, Pluzanska A, Chi KN et al. Docetaxel plus prednisone or mitoxantrone plus prednisone for advanced prostate cancer. N Engl J Med 2004;351(15):1502-12.

24. Hawkey CJ. COX-2 inhibitors. Lancet 1999;353(9149):307-14.

25. Taketo MM. Cyclooxygenase-2 inhibitors in tumorigenesis (Part II). J Natl.Cancer Inst. 1998;90(21):1609-20.

26. Taketo MM. Cyclooxygenase-2 inhibitors in tumorigenesis (part I). J Natl.Cancer Inst. 1998;90(20):1529-36.

27. Nelson JE, Harris RE. Inverse association of prostate cancer and non-steroidal anti-inflammatory drugs (NSAIDs): results of a case-control study. Oncol Rep. 2000;7(1):169-70.

28. Eberhart CE, Coffey RJ, Radhika A, Giardiello FM, Ferrenbach S, DuBois RN. Up-regulation of cyclooxygenase 2 gene expression in human colorectal adenomas and adenocarcinomas. Gastroenterology 1994;107(4):1183-8.

29. Roberts RO, Jacobson DJ, Girman CJ, Rhodes T, Lieber MM, Jacobsen SJ. A population-based study of daily nonsteroidal anti-inflammatory drug use and prostate cancer. Mayo Clin Proc. 2002;77(3):219-25.

30. Basler JW, Piazza GA. Nonsteroidal anti-inflammatory drugs and cyclooxygenase-2 selective inhibitors for prostate cancer chemoprevention. J Urol. 2004;171(2 Pt 2):S59-S62.

31. Masferrer JL, Leahy KM, Koki AT, Zweifel BS, Settle SL, Woerner BM et al. Antiangiogenic and antitumor activities of cyclooxygenase-2 inhibitors. Cancer Res. 2000;60(5):1306-11.

32. Hsu AL, Ching TT, Wang DS, Song X, Rangnekar VM, Chen CS. The cyclooxygenase-2 inhibitor celecoxib induces apoptosis by blocking Akt activation in human prostate cancer cells independently of Bcl-2. J Biol.Chem. 2000;275(15):11397-403.

33. Steinbach G, Lynch PM, Phillips RK, Wallace MH, Hawk E, Gordon GB et al. The effect of celecoxib, a cyclooxygenase-2 inhibitor, in familial adenomatous polyposis. N.Engl.J.Med. 2000;342(26):1946-52.

34. Solomon SD, McMurray JJ, Pfeffer MA, Wittes J, Fowler R, Finn P et al. Cardiovascular risk associated with celecoxib in a clinical trial for colorectal adenoma prevention. N Engl J Med 2005;352(11):1071-80.

35. Boissier S, Ferreras M, Peyruchaud O, Magnetto S, Ebetino FH, Colombel M et al. Bisphosphonates inhibit breast and prostate carcinoma cell invasion, an early event in the formation of bone metastases. Cancer Res. 2000;60(11):2949-54.

36. Corey E, Brown LG, Quinn JE, Poot M, Roudier MP, Higano CS et al. Zoledronic acid exhibits inhibitory effects on osteoblastic and osteolytic metastases of prostate cancer. Clin Cancer Res. 2003;9(1):295-306.

37. Witters LM, Crispino J, Fraterrigo T, Green J, Lipton A. Effect of the combination of docetaxel, zoledronic acid, and a COX-2 inhibitor on the growth of human breast cancer cell lines. Am.J Clin Oncol 2003;26(4):S92-S97.

38. Hiraga T, Ueda A, Tamura D, Hata K, Ikeda F, Williams PJ et al. Effects of oral UFT combined with or without zoledronic acid on bone metastasis in the 4T1/luc mouse breast cancer. Int.J Cancer 2003;106(6):973-9.

39. Neville-Webbe HL, Rostami-Hodjegan A, Evans CA, Coleman RE, Holen I. Sequence- and schedule-dependent enhancement of zoledronic acid induced apoptosis by doxorubicin in breast and prostate cancer cells. Int.J Cancer 2005;113(3):364-71.

40. Royston P, Parmar MK, Qian W. Novel designs for multi-arm clinical trials with survival outcomes with an application in ovarian cancer. Stat.Med 2003;22(14):2239-56.

41. Royston, P. stage2- program. MRC Clinical Trials Unit. 2001.

42. kind, P. The EuroQoL instrument: an index of health-related quality of life. In Spilker B, Quality of Life and Pharmaeconomics in Clinical Trials, Philadelphia: Lippincott-Raven. 1996.

43. Therasse P, Arbuck SG, Eisenhauer EA, Wanders J, Kaplan RS, Rubinstein L et al. New guidelines to evaluate the response to treatment in solid tumors. European Organization for Research and Treatment of Cancer, National Cancer Institute of the United States, National Cancer Institute of Canada. J Natl.Cancer Inst. 2000;92(3):205-16.

44. Dolan, P, Gudex, C, kind, P, and et al. A Social Tariff for EuroQol: Results from a UK General Population Survey. Centre for Health Economics Discussion Paper 138. Centre for Health Economics, University of York: CHE. 1995.

21 APPENDICES

APPENDIX A – ADDITIONAL DEFINITIONS 47
APPENDIX B - PATIENT INFORMATION SHEETS & CONSENT FORM 49
APPENDIX C - GP LETTER 82
APPENDIX D - MOLECULAR GENETICS SUB-STUDY 84
APPENDIX E - DRUG SUPPLY INFORMATION 85
APPENDIX F - ADMINISTRATION OF DOCETAXEL AND ZOLEDRONIC ACID 86
APPENDIX G - DRUG SAFETY INFORMATION FOR DRUGS USED IN THE TRIAL 93
APPENDIX H - HEALTH CARE AT HOME STUDY 103
APPENDIX I - EVALUATION OF BASELINE LESIONS AND NEW LESIONS 104
APPENDIX J - COMMON TOXICITY CRITERIA 106
APPENDIX K - DEFINITION OF BIOCHEMICAL FAILURE 110
APPENDIX L - TRIAL CONTACTS...113
APPENDIX M - PARTICIPATING SITE ACCREDITATION FORM 116
APPENDIX N - QUALITY OF LIFE AND HEALTH ECONOMICS 119
APPENDIX O - CASE REPORT FORMS 122
APPENDIX P - ASSESSING AND NOTIFYING CTU OF ADVERSE EVENTS 123

Appendix A – Additional Definitions

A1: WHO Performance Status

Grade	Performance status
0	Able to carry out all normal activity without restriction
1	Restricted in physically strenuous activity but ambulatory and able to carry out light work
2	Ambulatory and capable of all self-care but unable to carry out any work; up and about more than 50% of waking hours
3	Capable of only limited self-care; confined to bed or chair more than 50% of waking hours
4	Completely disabled; cannot carry on any self-care; totally confined to bed or chair

A2: TNM Classification*

Primary Tumour

Tx Primary tumour cannot be assessed
T0 No evidence of primary tumour
T1 Clinically upapparent tumour not palpable or visible by imaging
- T1a: Tumour incidental, found in 5% or less resected tissue
- T1b: Tumour incidental, found in more than 5% resected tissue
- T1c: Tumour identified by needle biopsy (because of high PSA)

T2 Tumour confined within the prostate gland
- T2a: Tumour involves one lobe
- T2b: Tumour involves both lobes

T3 Tumour extends through the prostatic capsule
- T3a: Extracapsular extensions (unilateral or bilateral)
- T3b: Tumour invades seminal vesicles

T4 Tumour is fixed or invades adjacent structures other than seminal vesicles: bladder neck, external sphincter, rectum, levator muscles and/or pelvic wall

Regional Lymph Nodes

Nx Regional lymph nodes have not been assessed
N0 No regional lymph node metastasis
N1 Regional lymph node metastasis

Distant metastasis

Mx Distant metastasis has not been assessed
M0 No distant metastasis
M1 Distant metastasis
- M1a: Non-regional lymph nodes
- M1b: Bone
- M1c: Other sites

*TNM staging of Prostate Cancer. (1997 TNM Classification of malignant tumours. Fifth edition. Wiley-Liss)

NYHA classification of heart failure

Class I: asymptomatic
No limitation in physical activity despite presence of heart disease. This can be suspected only if there is a history of heart disease which is confirmed by investigations - for example, echocardiography

Class II: mild
Slight limitation in physical activity. More strenuous activity causes shortness of breath - for example, walking on steep inclines and several flights of steps. Patients in this group can continue to have an almost normal lifestyle and employment

Class III: moderate
More marked limitation of activity which interferes with work. Walking on the flat produces symptoms

Class IV: severe
Unable to carry out any physical activity without symptoms. Patients are breathless at rest and mostly housebound

Appendix B - Patient Information Sheets & Consent Form

Guidance for Administrators

As STAMPEDE is a relatively complex trial with six different treatment arms, each of which requires a certain amount of specific patient information, the patient information sheets have been organised into two sections.

1. General Patient Information Sheet.

This is intended for use prior to randomisation. It describes the reason for the research, the design of the trial and a brief outline of the treatments that the patient may receive.

2. Treatment Arm-Specific Patient Information Sheets

These should usually be given to the patient after randomisation and before treatment starts. Some patients may wish to receive all of these before making a decision about randomisation; this is acceptable, but is not mandatory. They include more detailed information about possible unwanted effects and safety instructions. These sheets also cover important general issues such as withdrawing from the research, indemnity and confidentiality.

There is also a separate information sheet entitled **Additional Research** that covers quality of life, health economics and the molecular genetics sub-study that should be given to all patients prior to randomisation.

These forms follow:

- STAMPEDE patient information sheet – general
- STAMPEDE additional research
- STAMPEDE patient consent form
- STAMPEDE patient information sheet – treatment group A
- STAMPEDE patient information sheet – treatment group B
- STAMPEDE patient information sheet – treatment group C
- STAMPEDE patient information sheet – treatment group D
- STAMPEDE patient information sheet – treatment group E
- STAMPEDE patient information sheet – treatment group F

STAMPEDE: PATIENT INFORMATION SHEET-GENERAL

(to be printed on local hospital headed paper)

Version 1.1 (May 2005)

A LARGE PRINT VERSION IS AVAILABLE ON REQUEST

STAMPEDE: Systemic Therapy in Advancing or Metastatic Prostate Cancer - Evaluation of Drug Efficacy.

General Information

Your doctor has explained to you that you have prostate cancer and has invited you to participate in this clinical trial. Before you decide, it is important for you to understand why the research is being done and what it will involve. Participation is entirely voluntary. If you decide not to take part, your decision will be accepted without question, and your subsequent treatment will not be affected in any way.

Why is this research being done?

Prostate cancer often depends upon the male hormone (testosterone) to grow, and one of the main ways of treating prostate cancer is to lower the level of male hormones in the body. This is called "hormone treatment". It is usually successful at first: further growth of the cancer is prevented and it may cause the cancer to shrink. However, after a period of time the cancer can and often does begin to grow again. This trial aims to investigate whether combining newer drugs with hormone treatment can lengthen the time before the cancer starts to grow again.

There are three different, newer drugs that have shown promising activity in prostate cancer that we wish to study. These are given in Table 1 below.

Table 1: The newer drugs in this study

Zoledronic acid (Zometa®) - Prostate cancer cells can spread to bones and weaken them. Zoledronic acid reduces bone destruction and hardens them; this may make them more resistant to attack by cancer cells.

Docetaxel (Taxotere®) - This is a drug that stops cells dividing and multiplying. It is currently used to treat lung, breast and ovarian cancer. Docetaxel, in combination with prednisone (a steroid), is approved by regulatory authorities for the treatment of hormone refractory prostate cancer (cancer that is no longer responsive to hormone therapy).

Celecoxib (Onsenal®) - This is an aspirin-like drug that is used to treat arthritis. It slows down the growth of cancer cells in laboratory tests, and is being tested for use in preventing bowel cancers in patients. We want to see if it has the same effect on prostate cancer.

This trial is called STAMPEDE and will look at the effect of combining one or two of the newer drug treatments described above with hormone treatment. In addition to studying the effect of the treatment on the growth of your cancer, we will also be asking how it has affected your quality of life, and will be aiming to find out more about how prostate cancer develops and grows. This knowledge will help us to better understand the disease and help treat patients like you in the future. Details of these additional studies are covered in the information sheet entitled "Additional Research" that you will have been given.

How is the research done?

The best way of determining whether one treatment is more effective than another is by carrying out a randomised controlled trial. This means that a computer will allocate which treatment you receive randomly and you will have a fair and equal chance of receiving any of the new treatments being studied. Neither you nor your doctor can choose the treatment and you should therefore be willing to accept whichever treatment you are allocated. We are aiming to include approximately 3300 men like you in this study (Figure 1). They will be treated at hospitals all over the UK and it is expected that it will take between 6 and 10 years to complete the study.

Figure 1: Entering the study

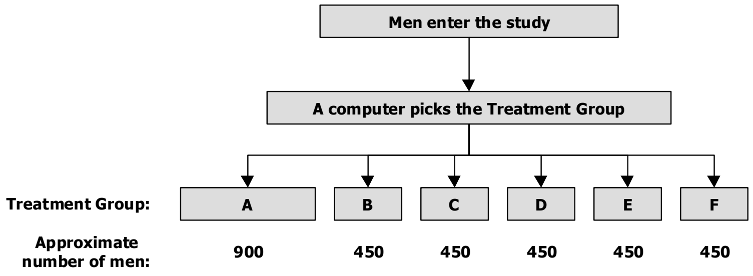

NB: A computer is used to allocate each of the treatments groups - A, B, C, D, E or F; these treatments are summarised in table 2. Overall twice as many men (2 out of 7) will be allocated to treatment group 'A' when compared to each of the other five treatment groups (5 out of 7: 1 per treatment group).

Which treatment might I receive if I take part?

All men taking part in the trial will receive hormone treatment which is standard treatment for your type of prostate cancer. The men who receive hormone treatment on its own, we have called Treatment Group A. The other men will receive hormone treatment and one or two of the newer drugs, and we have called these Treatment Groups B, C, D, E and F. Details of the

treatments are given in Table 2 (below). Overall, two out of every seven men in the trial can expect to receive hormone treatment only. The other five out of seven men will be allocated equally between the five newer research treatments.

There are separate information sheets for each treatment group that give more details about hormone therapy and the newer drugs. If you choose to enter the trial you will be given the sheet for the treatment group you are allocated, but you are welcome to read all of these at any time.

Table 2: The Different Treatment Groups in the STAMPEDE Trial.

A. Hormone Treatment Only

If you are in Treatment Group A, you will receive hormone treatment only. This is the standard treatment for patients with your type of prostate cancer. Your study doctor will discuss the different types of hormone treatment and together you can decide on the best form of hormone treatment for you. The options within the trial are:

1. "Anti-hormone" injections: these are given once a month or once every three months.
2. An operation where all or the functioning parts of both testicles are removed. This is called a bilateral orchidectomy.

B. Hormone Treatment and Zoledronic Acid

If you are in Treatment Group B, you will receive hormone treatment as described in Treatment Group A and zoledronic acid. Zoledronic acid will be given as an injection into the vein once every three weeks, for 6 injections and then once every four weeks for up to two years.

C. Hormone Treatment and Docetaxel

If you are in Treatment Group C, you will receive hormone treatment as described in Treatment Group A and docetaxel. Docetaxel will be given as an injection into a vein once every three weeks, for a total of 6 injections. You will also be given steroid tablets to take while you are on docetaxel.

D. Hormone Treatment and Celecoxib

If you are in Treatment Group D, you will receive hormone treatment as described in Treatment Group A and celecoxib. Celecoxib will be given as tablets. You will need to take these twice a day for a maximum of 1 year, but may stop them if your cancer shows signs of getting worse.

E. Hormone Treatment and Zoledronic Acid and Docetaxel

If you are in Treatment Group E, you will receive hormone treatment as described in Treatment Group A and both zoledronic acid and docetaxel. Zoledronic acid and docetaxel will be given as separate injections into a vein once every three weeks for the first 6 injections. Then only zoledronic acid will continue to be given, once every four weeks for up to two years. You will also be given steroid tablets to take while you are on docetaxel.

F. Hormone Treatment and Zoledronic Acid and Celecoxib

If you are in Treatment Group F, you will receive hormone treatment as described in Treatment Group A and both zoledronic acid and celecoxib. Zoledronic acid will be given as an injection into the vein once every three weeks for the first 6 injections and then every four weeks for up to two years. Celecoxib will be given as tablets. You will need to take these twice a day for a maximum of 1 year, but may stop them if your cancer shows signs of getting worse.

What are the side-effects ?

The standard therapy, hormone treatment alone, lowers the level of the male hormone testosterone in the body. It may cause impotence, decreased sex drive (loss of libido), hot flushes, and occasionally may cause a small amount of swelling of breast tissue. Table 3 outlines the most common side-effects that might occur with the newer treatments, however most patients will not experience all of these.

**Table 3: Possible side-effects from the newer treatments**
**Zoledronic acid -** flu-like symptoms, e.g. raised temperature and body aches.
**Docetaxel -** hair loss, allergic reactions, fluid retention, sickness, anaemia. Also it may lower the level of other blood cells making you more susceptible to bruising, bleeding and infections.
**Celecoxib -** stomach discomfort, sinusitis (painful sinuses), insomnia and a small increased risk of a cardiovascular event (see below)
Recently, information from three long-term studies of celecoxib has become available. In the first study, which was aimed at preventing rather treating cancer, a small increased risk of heart attacks, strokes, and/or deaths resulting from heart or blood vessel disease was reported among people taking celecoxib. Approximately 1 in 100 patients in this study on placebo treatment had one of these serious events. In contrast, between 2 and 3 in 100 patients taking celecoxib (between 400 and 800 mg daily) had one of these serious events. Another clinical cancer prevention study found no increased risks in patients taking celecoxib 400 mg daily. The third study, an Alzheimer's disease prevention study, did not find increased risks with celecoxib..

Do I have to take part?

Your participation in the STAMPEDE trial is entirely voluntary. If you decide not to take part, your decision will be accepted without question, and your subsequent treatment will not be affected in any way. The standard treatment for your type of prostate cancer is hormone treatment. All men taking part in the trial will receive hormone treatment. If you agree to take part you are free to withdraw from the trial treatment without giving your reasons. To ensure full co-operation between the hospital and the surgery we would like to inform your family doctor about the treatment and the details of the trial. If you do not want your GP to be informed about your treatment or involvement in the study please discuss this with your study doctor.

What will happen to me if I take part?

Whether you receive your treatment in hospital or at home may depend on the Treatment Group you are in. If you are to receive docetaxel, it will be given to you in a specialised ward or unit <insert where this would be>. If you are to receive zoledronic acid you will also need to make extra visits to your hospital for treatment though it may be possible for some of the treatments to be administered at home. Celecoxib tablets can be taken at home. If you agree

to take part in the trial there will be some additional blood tests to measure the progress of your cancer (approximately 6 in the first 2 years). If you are to receive zoledronic acid and / or docetaxel (Treatment Groups B, C, E or F) there will be some more blood tests to monitor the safety of your treatment. Your study doctor will see you at least every 3 months for 3 years, then at least every 6 months as part of this trial.

What are the possible advantages of taking part in the trial?

There are a number of newer treatments that have shown early promise against prostate cancer as described above. Although the study treatments have shown promise in treating prostate cancer we don't know whether these treatments in combination with hormone treatment will be better than hormone treatment alone. Therefore your participation may not be of direct benefit to you, however it will help answer this question and help us to improve treatment for men like you in the future.

What are the possible disadvantages?

In this trial you may be asked to take additional treatment(s) as well as the hormone treatment. We do not expect there to be any disadvantage to you in terms of how your cancer responds but the additional treatments will probably have some additional side-effects (Table 3). There will also be some extra hospital visits and blood tests, as described above.

What will happen to the results of the research study?

The results will be of interest to a variety of health professionals including consultants, nurses and other healthcare professionals. Our findings will be disseminated through conference presentations and journal articles and through links already existing between the MRC and hospital sites. Your study doctor will be informed of the results and he/she can discuss the results with you and a copy of the results will be freely available to you on request. No patients will be identified in such publications.

Will my taking part be kept confidential?

If you do choose to participate we should like to tell your GP, but only with your permission. Relevant medical information about you will be sent regularly to MRC Clinical Trials Unit who are running the trial. It will be treated confidentially at all times and kept in a secure area, in a manner compliant with the Data Protection Act. Scientific and medical employees of the MRC Clinical Trials Unit and other staff involved in the trial may need to examine your medical records, but your confidentiality will be maintained. We would also like to register your name with the Office of National Statistics (ONS) if you were born or live in England or Wales, or with the General Register Office (GRO) if you were born or live in Scotland; this would allow us to check on your health status after the trial has closed, even if you are no longer seeing your hospital doctor. Again full confidentiality would be maintained.

Further Information

Whichever treatment(s) you receive, the aim is to control your cancer and help you feel well for as long as possible. If your cancer starts to grow despite the treatment or any new information becomes available or you would be better helped by an alternative treatment, your study doctor will discuss this with you. You can withdraw from the trial at anytime and it will not affect your subsequent care in any way. Similarly, your care will not be affected if you do not choose to participate.

More information about clinical trials can be found on the Cancer Research UK's patient website, Cancer Help UK (www.cancerhelp.org.uk). CancerBACUP also provide information on all aspects of cancer care including clinical trials (freephone 0808 800 1234 or www.cancerbacup.org). Consumers for Ethics in Research (CERES) publish a leaflet entitled 'Medical Research and You'. This leaflet gives more information about medical research and looks at some questions potential recruits may want to ask. You may obtain copies from CERES, PO Box 1365, London N16 0BW.

If you have private medical insurance, you may wish to check with your company before agreeing to take part in the trial to ensure that your participation in the trial will not affect your insurance cover.

Please ask any questions you may have; take time to discuss the trial with your family, friends and family doctor. If you agree to take part in the trial, you will be asked to sign a consent form.

For more information, please contact …

<Please insert Principal Investors Names and Address>

STAMPEDE ADDITIONAL RESEARCH

(to be printed on local hospital headed paper)

Version 1.1 (May 2005)

- **QUALITY OF LIFE + HEALTH ECONOMICS STUDY - PATIENT INFORMATION SHEET**

About your questionnaires

As well as looking at the effect different treatments have on your cancer we think it is equally important to find out how you feel when you are having your treatment and after your treatment has finished. Therefore, we are asking you to complete some questionnaires that are designed to find out more about your physical and emotional feelings while you are on the trial.

These questionnaires ask you how you have been feeling during the past week and are designed to assess your day-to-day well-being, as well as any side-effects you may be experiencing. We will also ask if you have required any additional medications, or visits to your hospital or GP. Your questionnaires will be sent directly to the MRC Clinical Trials Unit where they will be treated in confidence and analysed together with questionnaires from patients in other hospitals. Your study doctor should not see the questionnaires once you have filled them in. This is to ensure that you are not influenced by what you think your study doctor may think about your responses. However, it is important that you report any concerns to your study doctor or research nurse.

We ask about a wide range of symptoms as the questionnaires are designed for use in many different areas of research, and may also be used to evaluate the impact of the newer treatments on the cost of health care in the UK.

Completing the questionnaires

If possible, you should complete the questionnaires on your own. Please make sure the correct date is written at the top of the questionnaire before you start. Try to answer all the questions but do not spend too much time thinking about each answer, as your first response is likely to be more accurate. If a question is not applicable to you, please write "not applicable" or "N/A" instead, but please do not leave any answers blank.

When you attend hospital for the first time, you will be asked to complete a questionnaire. We would like you to complete further questionnaires when you come to the hospital for an assessment at 6, 12 18 and 24 weeks after you enter the trial. Then every 3 months for the next 3 years followed by every 6 months thereafter. If you are not given a questionnaire to complete, please remind your doctor. You can, of course, decline to complete a questionnaire at any time without affecting your relationship with your doctor or your participation in the trial.

- **MOLECULAR GENETICS SUB-STUDY**

We would like to ask you to donate a small sample of your blood (8ml) which will be stored for future use at University College London. It is particularly useful for researchers to have access to blood samples from patients who have your type of cancer. These samples will be used to research the causes of prostate cancer and the way in which prostate cancers respond to different treatments.

The donation of a sample is entirely voluntary and you are free to withhold permission without it affecting your participation in the STAMPEDE TRIAL or your relationship with your doctor in any way.

The research may involve the analysis of components of your blood sample such as DNA or proteins. We would like to see whether certain genes are associated either with certain side effects or with beneficial results from the drugs we are using in this study. This research may help to predict which patients will benefit most from future treatments. This part of the research is unlikely to benefit you, but may help to improve treatments and be of benefit to cancer sufferers in the future.

All such work is anonymous as far as possible. Your blood sample will be coded by a number and your name will not be used. It is not intended to link the results of any genetic analysis back to you or your relatives. Neither you nor your relatives will be identified or contacted in connection with this research. Your personal information will be stored confidentially and will only be accessible to staff who have a duty of confidentiality to you as the donor of the blood sample, in accordance with the Data Protection Act.

The Medical Research Council (MRC) will specify the arrangements for access to and control of the use of the blood samples. Access to the blood samples by the commercial sector or other academic researchers is not excluded, but no one commercial company will be given exclusive rights of access. This blood sample should be considered as a gift and donors would not be entitled to share in (the unlikely event of) any financial profits from this research.

All research projects involving the stored blood samples will be approved by an independent ethics committee. If we find anything important during our research on prostate cancer, it will be published in a well-recognised scientific journal so it will be available to the whole medical and scientific community. Your personal details will not be identified in any way in any publication.

If you have any further questions please contact: Professor John Masters

3rd Floor research Laboratories
67 Riding House Street
London, W1W 7EJ
Tel: 020 7679 9543
Fax: 020 7679 9366
E-mail: j.masters@ucl.ac.uk

STAMPEDE: Patient consent form
Version 1.1 (May 2005) (to be printed on local hospital headed paper)

STAMPEDE: Systemic Therapy in Advancing or Metastatic Prostate cancer - Evaluation of Drug Efficacy.

Please initial the appropriate boxes:

	Agree	Disagree
1. I have read the patient information sheet on this clinical trial and have been given a copy to keep. I have had the opportunity to ask questions about the project and understand why the research is being done and have been told about possible side effects.	☐	
2. I agree to participate in the Quality of Life/Health Economics study and to complete these questionnaires. *(This is optional)*	☐	☐
3. I have read the patient information sheet relating to the Molecular Genetics Study and agree to donating 8ml blood which will be stored and may be used for research in the future. I understand this is a gift. *(This is optional)*	☐	☐
4. I understand that sections of any of my medical notes may be looked at by responsible individuals from the Medical Research Council (MRC) Clinical Trials Unit (CTU) or from regulatory authorities where it is relevant to my taking part in research. I give permission for these individuals to have access to my records, but understand that my confidentiality will be maintained.	☐	
5. I understand that I may not benefit directly by participating in this study but that the research may help men like me in the future.	☐	
6. I understand that information about me will be held by the MRC CTU and that the unit is registered under the Data Protection Act to hold such information, and that my GP will be informed.	☐	
7. I agree that my data can be stored and used for this clinical trial.	☐	
8. I understand that my participation in all aspects of this trial is voluntary and that I am free to withdraw from the trial at any time, without giving any reason and without my medical care or legal rights being affected.	☐	
9. I agree that I can be flagged with the Office for National Statistics for the purposes of long-term follow-up. *(This is optional)*	☐	
10. I agree to take part in the above trial.	☐	
11. I agree that my GP can be notified of my participation in the STAMPEDE trial.	☐	☐

Name of patient (BLOCK CAPITALS)	Date (dd/mm/yyyy)	Signature
Name of researcher (BLOCK CAPITALS)	Date (dd/mm/yyyy)	Signature

Please sign 3 copies: 1 copy to be kept by the patient, 1 copy to be kept with hospital notes, 1 copy kept in the local investigator's file

STAMPEDE PATIENT INFORMATION SHEET – TREATMENT GROUP A

(to be printed on local hospital headed paper)
Version 1.1 (May 2005)

Information for patients receiving Hormone Treatment

You are going to receive hormone treatment alone. Details of the treatment are given below.

Hormone Treatment

Prostate cancers often depend upon the male hormone testosterone to grow. Reducing the amount of testosterone in the body usually prevents further growth of the cancer and may cause it to shrink. This is called hormone treatment and can be achieved either by the use of anti-hormone injections or an operation to remove part or all of both testicles, which produce the male hormone. Your doctor will discuss these different options with you and together you can decide which is the best form of hormone treatment for you. All forms of hormone treatment can cause the following side-effects: impotence, loss of libido (sexual drive), hot flushes, occasional swelling of breast tissue and absent-mindedness. In addition, if you receive the treatment over a long period of time you may notice an increase in weight, a reduction in your muscle tissues and your bones may be weakened.

Anti-hormone Injections: These injections (known as LHRH analogues) stop the production of the male hormone testosterone by the testicles. Depending on the type of injection, they are given once every month or once every three months into the skin of the abdomen or into the arm. Occasionally they temporarily aggravate the cancer before a benefit occurs and for this reason additional anti-hormone tablets are given for the first few weeks of the treatment. Other unwanted effects that have been reported are allergic reactions, irritation at the injection site and headaches. The injections usually have to continue indefinitely.

OR

Bilateral Subcapsular Orchidectomy: This is an operation where the functioning part of the testicles is removed. This is normally done by taking out the centre of the testicles, leaving the testicles themselves behind but reduced in size. Sometimes, instead of this operation the testicles are removed completely. Your surgeon will discuss the surgical options with you. These operations are usually straightforward but there will be some pain or discomfort in the scrotum afterwards. There may also be some swelling and bruising in the scrotum that takes a couple of weeks to subside, and as with any surgical operation an infection can occur in the wound.

Treatment Summary Table

What is my treatment?	How will I be given the treatment?	When, and for how long for?
Androgen Suppression	Orchidectomy or Hormone Treatment	According to Local Practice. (Centres to complete)

Will my doctor be paid if I participate?

There will be no payment to your doctor if you choose to participate in the trial. However, the trial is part of the National Cancer Research Network (NRCN) portfolio of trials. This means your doctor will be entitled to research support for the trial (for example, help from a research nurse to run the trial).

Will I be paid for participating?

There will be no payments to patients who agree to participate in this trial and additional travel expenses will not be reimbursed.

What if I change my mind about participating in the study?

If you change your mind about taking part in the study, you can withdraw from the trial at any time. This will not affect your relationship with the doctors and nurses, or your subsequent care, in any way. Even though you would not be taking part in the trial in this case, we would still like to monitor your progress. If you agree we would like to continue to collect some information so that the long-term effects of your treatment can be assessed.

Who is organising this trial?

The Medical Research Council (MRC) has overall responsibility for this trial. The study is funded by the Clinical Trials Advisory and Awards Committee which allocates money from MRC and Cancer Research UK. Some financial support has also been obtained from Novartis and the newer drugs have been supplied by the Pharmaceutical companies Novartis, Aventis and Pfiser free of charge or at a reduced cost. The study has received the favourable opinion of an Independent Research Ethics Committees. The trial is run from the MRC Clinical Trials Unit (CTU), London.

What if things go wrong?

The MRC is the sponsor of this trial and as such would give sympathetic consideration to claims for compensation for any non-negligent harm that you may suffer by participating in this trial. The MRC and NHS are both publicly funded bodies and are not allowed to purchase advance insurance to cover indemnity because they are backed by the resources of the Treasury. Like other publicly funded bodies, any liability arising from the MRC's activities is

underwritten by the UK Government. However, this does not extend to harm arising from receiving the “standard treatment”.

The hospital(s) you are treated in continue to have a duty of care to you, whether or not you agree to participate in this trial. Therefore, the MRC does not accept liability for negligence on the part of employees of hospital. This applies whether the hospital is an NHS Trust or not, and the MRC cannot be held liable for any breach in the hospital’s duty of care. If you wish to complain about any aspect of the way you have been approached or treated during the course of the study, the normal National Health Service complaint mechanisms will be available to you.

What if new information becomes available?

Sometimes during the course of a research project, new information becomes available about the treatment/drug that is being studied. Throughout the study, medical information from this study and any other studies will be looked at by an independent committee. If any new information becomes available that may affect your participation in this study or would affect your future care, your study doctor will tell you about it and discuss with you whether you want to continue in the study. If you decide to withdraw, your study doctor will make arrangements for your care to continue. If you decide to continue in the study you would be asked to sign an updated consent form.

Please report any unwanted effects to your cancer doctor or nurse.

If you become unwell between hospital visits, please seek advice immediately, either from your hospital team or from your GP.

Your contact numbers are:

STAMPEDE PATIENT INFORMATION SHEET – TREATMENT GROUP B

(to be printed on local hospital headed paper)

Version 1.1 (May 2005)

Information for patients receiving Hormone Treatment and Zoledronic Acid

You are going to receive hormone treatment and zoledronic acid. Details of the treatment are given below.

Hormone Treatment

Prostate cancers often depend upon the male hormone testosterone to grow. Reducing the amount of testosterone in the body usually prevents further growth of the cancer and may cause it to shrink. This is called hormone treatment and can be achieved either by the use of anti-hormone injections or an operation to remove part or all of both testicles, which produce the male hormone. Your doctor will discuss these different options with you and together you can decide which is the best form of hormone treatment for you. All forms of hormone treatment can cause the following side-effects: impotence, loss of libido (sexual drive), hot flushes, occasional swelling of breast tissue and absent-mindedness. In addition, if you receive the treatment over a long period of time you may notice an increase in weight, a reduction in your muscle tissues and your bones may be weakened.

Anti-hormone Injections: These injections (known as LHRH analogues) stop the production of the male hormone testosterone by the testicles. Depending on the type of injection, they are given once every month or once every three months into the skin of the abdomen or into the arm. Occasionally they temporarily aggravate the cancer before a benefit occurs and for this reason additional anti-hormone tablets are given for the first few weeks of the treatment. Other unwanted effects that have been reported are allergic reactions, irritation at the injection site and headaches. The injections usually have to continue indefinitely.

OR

Bilateral Subcapsular Orchidectomy: This is an operation where the functioning part of the testicles is removed. This is normally done by taking out the centre of the testicles, leaving the testicles themselves behind but reduced in size. Sometimes, instead of this operation the testicles are removed completely. Your surgeon will discuss the surgical options with you. These operations are usually straightforward but there will be some pain or discomfort in the scrotum afterwards. There may also be some swelling and bruising in the scrotum that takes a couple of weeks to subside, and as with any surgical operation an infection can occur in the wound.

Zoledronic Acid

Zoledronic acid is a drug that prevents weakening of bones. It will be given as an injection into a vein once every 3 weeks for the first 6 injections and then once every 4 weeks until 2 years. The drug will be stopped earlier if there is a reason to suggest that your cancer is not responding to the treatment. The injection will take approximately 15 minutes. Unwanted effects are unusual although it may cause flu-like symptoms such as fever, bone pain and muscle cramps. It may also lower the level of some of the salts in your blood such as calcium and phosphate; blood tests will be needed to monitor these levels.

Treatment Summary Table

What is my treatment?	How will I be given the treatment?	When, and for how long for?
Androgen Suppression	Orchidectomy or Hormone Treatment	According to Local Practice. (Centres to complete)
Zoledronic Acid	15 Minute Intravenous (IV) Infusion	Once every 3 weeks for 18 weeks then<br>Once every 4 weeks for up to 2 years.

(A blood test will be performed before every cycle of Zoledronic Acid).

Will my doctor be paid if I participate?

There will be no payment to your doctor if you choose to participate in the trial. However, the trial is part of the National Cancer Research Network (NRCN) portfolio of trials. This means your doctor will be entitled to research support for the trial (for example, help from a research nurse to run the trial).

Will I be paid for participating?

There will be no payments to patients who agree to participate in this trial and additional travel expenses will not be reimbursed.

What if I change my mind about participating in the study?

If you change your mind about taking part in the study, you can withdraw from the trial at any time. This will not affect your relationship with the doctors and nurses, or your subsequent care, in any way. Even though you would not be taking part in the trial in this case, we would still like to monitor your progress. If you agree we would like to continue to collect some information so that the long-term effects of your treatment can be assessed.

Who is organising this trial?

The Medical Research Council (MRC) has overall responsibility for this trial. The study is funded by the Clinical Trials Advisory and Awards Committee which allocates money from MRC and Cancer Research UK. Some financial support has also been obtained from Novartis and the newer drugs have been supplied by the Pharmaceutical companies Novartis, Aventis and Pfiser free of charge or at a reduced cost. The study has received the favourable opinion of an Independent Research Ethics Committees. The trial is run from the MRC Clinical Trials Unit (CTU), London.

What if things go wrong?

The MRC is the sponsor of this trial and as such would give sympathetic consideration to claims for compensation for any non-negligent harm that you may suffer by participating in this trial. The MRC and NHS are both publicly funded bodies and are not allowed to purchase advance insurance to cover indemnity because they are backed by the resources of the Treasury. Like other publicly funded bodies, any liability arising from the MRC's activities is underwritten by the UK Government. However, this does not extend to harm arising from receiving the "standard treatment".

The hospital(s) you are treated in continue to have a duty of care to you, whether or not you agree to participate in this trial. Therefore, the MRC does not accept liability for negligence on the part of employees of hospital. This applies whether the hospital is an NHS Trust or not, and the MRC cannot be held liable for any breach in the hospital's duty of care. If you wish to complain about any aspect of the way you have been approached or treated during the course of the study, the normal National Health Service complaint mechanisms will be available to you.

What if new information becomes available?

Sometimes during the course of a research project, new information becomes available about the treatment/drug that is being studied. Throughout the study, medical information from this study and any other studies will be looked at by an independent committee. If any new information becomes available that may affect your participation in this study or would affect your future care, your study doctor will tell you about it and discuss with you whether you want to continue in the study. If you decide to withdraw, your study doctor will make arrangements for your care to continue. If you decide to continue in the study you would be asked to sign an updated consent form.

Please report any unwanted effects to your cancer doctor or nurse.

If you become unwell between hospital visits, please seek advice immediately, either from your hospital team or from your GP.

Your contact numbers are:

STAMPEDE PATIENT INFORMATION SHEET – TREATMENT GROUP C

(to be printed on local hospital headed paper)

Version 1.1 (May 2005)

Information for patients receiving Hormone Treatment and Docetaxel

You are going to receive hormone treatment and docetaxel. Details of the treatment are given below.

Hormone Treatment

Prostate cancers often depend upon the male hormone testosterone to grow. Reducing the amount of testosterone in the body usually prevents further growth of the cancer and may cause it to shrink. This is called hormone treatment and can be achieved either by the use of anti-hormone injections or an operation to remove part or all of both testicles, which produce the male hormone. Your doctor will discuss these different options with you and together you can decide which is the best form of hormone treatment for you. All forms of hormone treatment can cause the following side-effects: impotence, loss of libido (sexual drive), hot flushes, occasional swelling of breast tissue and absent-mindedness. In addition, if you receive the treatment over a long period of time you may notice an increase in weight, a reduction in your muscle tissues and your bones may be weakened.

Anti-hormone Injections: These injections (known as LHRH analogues) stop the production of the male hormone testosterone by the testicles. Depending on the type of injection, they are given once every month or once every three months into the skin of the abdomen or into the arm. Occasionally they temporarily aggravate the cancer before a benefit occurs and for this reason additional anti-hormone tablets are given for the first few weeks of the treatment. Other unwanted effects that have been reported are allergic reactions, irritation at the injection site and headaches. The injections usually have to continue indefinitely.

OR

Bilateral Subcapsular Orchidectomy: This is an operation where the functioning part of the testicles is removed. This is normally done by taking out the centre of the testicles, leaving the testicles themselves behind but reduced in size. Sometimes, instead of this operation the testicles are removed completely. Your surgeon will discuss the surgical options with you. These operations are usually straightforward but there will be some pain or discomfort in the scrotum afterwards. There may also be some swelling and bruising in the scrotum that takes a couple of weeks to subside, and as with any surgical operation an infection can occur in the wound.

Docetaxel

Docetaxel will be given as an injection into a vein once every three weeks, for a total of six injections. In addition, you will be given prednisolone (steroid tablets) to be taken twice a day while you are receiving the docetaxel. The docetaxel injection will take approximately one hour. Unwanted effects may include nausea and vomiting (although you will be given additional medication to prevent this), hair loss, fluid retention such as lower leg swelling, allergic reactions and numbness of the hands and feet. Docetaxel may also effect some cells in your blood, causing anaemia or making you more susceptible to bruising, bleeding and infections. It will be necessary to check your blood count before each injection to be sure it is safe. If you have a temperature or become unwell while you are on docetaxel you should contact your hospital cancer team immediately. The 24 hour contact details for your hospital are given below.

The prednisolone tablets may cause indigestion, weight gain, swelling of the lower legs, increased appetite, mood changes and difficulty in sleeping.

Docetaxel, in combination with prednisone (a steroid), is approved by regulatory authorities for the treatment of hormone refractory prostate cancer (cancer that is no longer responsive to hormone therapy).

Treatment Summary Table

What is my treatment?	How will I be given the treatment?	When, and for how long for?
Androgen Suppression	Orchidectomy or Hormone Treatment	According to Local Practice. Centres to complete)
Docetaxel	1 hour Intravenous (IV) Infusion	Once every three weeks for 18 weeks.

(Blood Tests will be taken before each session of Docetaxol treatment)

Will my doctor be paid if I participate?

There will be no payment to your doctor if you choose to participate in the trial. However, the trial is part of the National Cancer Research Network (NRCN) portfolio of trials. This means your doctor will be entitled to research support for the trial (for example, help from a research nurse to run the trial).

Will I be paid for participating?

There will be no payments to patients who agree to participate in this trial and additional travel expenses will not be reimbursed.

What if I change my mind about participating in the study?

If you change your mind about taking part in the study, you can withdraw from the trial at any time. This will not affect your relationship with the doctors and nurses, or your subsequent care, in any way. Even though you would not be taking part in the trial in this case, we would still like to monitor your progress. If you agree we would like to continue to collect some information so that the long-term effects of your treatment can be assessed.

Who is organising this trial?

The Medical Research Council (MRC) has overall responsibility for this trial. The study is funded by the Clinical Trials Advisory and Awards Committee which allocates money from MRC and Cancer Research UK. Some financial support has also been obtained from Novartis and the newer drugs have been supplied by the Pharmaceutical companies Novartis, Aventis and Pfiser free of charge or at a reduced cost. The study has received the favourable opinion of an Independent Research Ethics Committees. The trial is run from the MRC Clinical Trials Unit (CTU), London.

What if things go wrong?

The MRC is the sponsor of this trial and as such would give sympathetic consideration to claims for compensation for any non-negligent harm that you may suffer by participating in this trial. The MRC and NHS are both publicly funded bodies and are not allowed to purchase advance insurance to cover indemnity because they are backed by the resources of the Treasury. Like other publicly funded bodies, any liability arising from the MRC's activities is underwritten by the UK Government. However, this does not extend to harm arising from receiving the "standard treatment".

The hospital(s) you are treated in continue to have a duty of care to you, whether or not you agree to participate in this trial. Therefore, the MRC does not accept liability for negligence on the part of employees of hospital. This applies whether the hospital is an NHS Trust or not, and the MRC cannot be held liable for any breach in the hospital's duty of care. If you wish to complain about any aspect of the way you have been approached or treated during the course of the study, the normal National Health Service complaint mechanisms will be available to you.

What if new information becomes available?

Sometimes during the course of a research project, new information becomes available about the treatment/drug that is being studied. Throughout the study, medical information from this study and any other studies will be looked at by an independent committee. If any new information becomes available that may affect your participation in this study or would affect

your future care, your study doctor will tell you about it and discuss with you whether you want to continue in the study. If you decide to withdraw, your study doctor will make arrangements for your care to continue. If you decide to continue in the study you would be asked to sign an updated consent form.

Please report any unwanted effects to your cancer doctor or nurse.

If you become unwell between hospital visits, please seek advice immediately, either from your hospital team or from your GP.

Your contact numbers are:

STAMPEDE PATIENT INFORMATION SHEET – TREATMENT GROUP D

(to be printed on local hospital headed paper)

Version 1.1 (May 2005)

Information for patients receiving Hormone Treatment and Celecoxib

You are going to receive hormone treatment and celecoxib. Details of the treatment are given below.

Hormone Treatment

Prostate cancers often depend upon the male hormone testosterone to grow. Reducing the amount of testosterone in the body usually prevents further growth of the cancer and may cause it to shrink. This is called hormone treatment and can be achieved either by the use of anti-hormone injections or an operation to remove part or all of both testicles, which produce the male hormone. Your doctor will discuss these different options with you and together you can decide which is the best form of hormone treatment for you. All forms of hormone treatment can cause the following side-effects: impotence, loss of libido (sexual drive), hot flushes, occasional swelling of breast tissue and absent-mindedness. In addition, if you receive the treatment over a long period of time you may notice an increase in weight, a reduction in your muscle tissues and your bones may be weakened.

Anti-hormone Injections: These injections (known as LHRH analogues) stop the production of the male hormone testosterone by the testicles. Depending on the type of injection, they are given once every month or once every three months into the skin of the abdomen or into the arm. Occasionally they temporarily aggravate the cancer before a benefit occurs and for this reason additional anti-hormone tablets are given for the first few weeks of the treatment. Other unwanted effects that have been reported are allergic reactions, irritation at the injection site and headaches. The injections usually have to continue indefinitely.

OR

Bilateral Subcapsular Orchidectomy: This is an operation where the functioning part of the testicles is removed. This is normally done by taking out the centre of the testicles, leaving the testicles themselves behind but reduced in size. Sometimes, instead of this operation the testicles are removed completely. Your surgeon will discuss the surgical options with you. These operations are usually straightforward but there will be some pain or discomfort in the scrotum afterwards. There may also be some swelling and bruising in the scrotum that takes a couple of weeks to subside, and as with any surgical operation an infection can occur in the wound.

Celecoxib

The celecoxib tablets are taken twice a day and will continue for a maximum of 1 year. They will only be stopped if your cancer shows signs of not responding to the treatment or if you and your doctor decide this is best. Unwanted effects may include stomach discomfort, indigestion, abdominal pain and flatulence, fluid retention, difficulty sleeping, sinusitis or a sore throat, a rash and a small increased risk of a cardiovascular event (see below).

Recently, information from three long-term studies of celecoxib has become available. In the first study, which was aimed at preventing rather than treating cancer, a small increased risk of heart attacks, strokes, and/or deaths resulting from heart or blood vessel disease was reported among people taking celecoxib. Approximately 1 in 100 patients in this study on placebo treatment had one of these serious events. In contrast, between 2 and 3 in 100 patients taking celecoxib (between 400 and 800 mg daily) had one of these serious events. Another clinical cancer prevention study found no increased risks in patients taking celecoxib 400 mg daily. The third study, an Alzheimer's disease prevention study, did not find increased risks with celecoxib.

Treatment Summary Table

What is my treatment?	How will I be given the treatment?	When, and for how long for?
Androgen Suppression	Orchidectomy or Hormone Treatment	According to Local Practice. (Centres to complete)
Celecoxib	Tablet	Two times a day for 1 year disease progression (which ever is earlier).

Will my doctor be paid if I participate?

There will be no payment to your doctor if you choose to participate in the trial. However, the trial is part of the National Cancer Research Network (NRCN) portfolio of trials. This means your doctor will be entitled to research support for the trial (for example, help from a research nurse to run the trial).

Will I be paid for participating?

There will be no payments to patients who agree to participate in this trial and additional travel expenses will not be reimbursed.

What if I change my mind about participating in the study?

If you change your mind about taking part in the study, you can withdraw from the trial at any time. This will not affect your relationship with the doctors and nurses, or your subsequent care, in any way. Even though you would not be taking part in the trial in this

case, we would still like to monitor your progress. If you agree we would like to continue to collect some information so that the long-term effects of your treatment can be assessed.

Who is organising this trial?

The Medical Research Council (MRC) has overall responsibility for this trial. The study is funded by the Clinical Trials Advisory and Awards Committee which allocates money from MRC and Cancer Research UK. Some financial support has also been obtained from Novartis and the newer drugs have been supplied by the Pharmaceutical companies Novartis, Aventis and Pfiser free of charge or at a reduced cost. The study has received the favourable opinion of an Independent Research Ethics Committees. The trial is run from the MRC Clinical Trials Unit (CTU), London.

What if things go wrong?

The MRC is the sponsor of this trial and as such would give sympathetic consideration to claims for compensation for any non-negligent harm that you may suffer by participating in this trial. The MRC and NHS are both publicly funded bodies and are not allowed to purchase advance insurance to cover indemnity because they are backed by the resources of the Treasury. Like other publicly funded bodies, any liability arising from the MRC's activities is underwritten by the UK Government. However, this does not extend to harm arising from receiving the "standard treatment".

The hospital(s) you are treated in continue to have a duty of care to you, whether or not you agree to participate in this trial. Therefore, the MRC does not accept liability for negligence on the part of employees of hospital. This applies whether the hospital is an NHS Trust or not, and the MRC cannot be held liable for any breach in the hospital's duty of care. If you wish to complain about any aspect of the way you have been approached or treated during the course of the study, the normal National Health Service complaint mechanisms will be available to you.

What if new information becomes available?

Sometimes during the course of a research project, new information becomes available about the treatment/drug that is being studied. Throughout the study, medical information from this study and any other studies will be looked at by an independent committee. If any new information becomes available that may affect your participation in this study or would affect your future care, your study doctor will tell you about it and discuss with you whether you want to continue in the study. If you decide to withdraw, your study doctor will make arrangements for your care to continue. If you decide to continue in the study you would be asked to sign an updated consent form.

Please report any unwanted effects to your cancer doctor or nurse.

If you become unwell between hospital visits, please seek advice immediately, either from your hospital team or from your GP.

Your contact numbers are:

STAMPEDE PATIENT INFORMATION SHEET – TREATMENT GROUP E

(to be printed on local hospital headed paper)

Version 1.1 (May 2005)

Information for patients receiving Hormone Treatment and Zoledronic Acid and Docetaxel

You are going to receive hormone treatment and zoledronic acid and docetaxel. Details of the treatment are given below.

Hormone Treatment

Prostate cancers often depend upon the male hormone testosterone to grow. Reducing the amount of testosterone in the body usually prevents further growth of the cancer and may cause it to shrink. This is called hormone treatment and can be achieved either by the use of anti-hormone injections or an operation to remove part or all of both testicles, which produce the male hormone. Your doctor will discuss these different options with you and together you can decide which is the best form of hormone treatment for you. All forms of hormone treatment can cause the following side-effects: impotence, loss of libido (sexual drive), hot flushes, occasional swelling of breast tissue and absent-mindedness. In addition, if you receive the treatment over a long period of time you may notice an increase in weight, a reduction in your muscle tissues and your bones may be weakened.

Anti-hormone Injections: These injections (known as LHRH analogues) stop the production of the male hormone testosterone by the testicles. Depending on the type of injection, they are given once every month or once every three months into the skin of the abdomen or into the arm. Occasionally they temporarily aggravate the cancer before a benefit occurs and for this reason additional anti-hormone tablets are given for the first few weeks of the treatment. Other unwanted effects that have been reported are allergic reactions, irritation at the injection site and headaches. The injections usually have to continue indefinitely.

OR

Bilateral Subcapsular Orchidectomy: This is an operation where the functioning part of the testicles is removed. This is normally done by taking out the centre of the testicles, leaving the testicles themselves behind but reduced in size. Sometimes, instead of this operation the testicles are removed completely. Your surgeon will discuss the surgical options with you. These operations are usually straightforward but there will be some pain or discomfort in the scrotum afterwards. There may also be some swelling and bruising in the scrotum that takes a couple of weeks to subside, and as with any surgical operation an infection can occur in the wound.

Zoledronic Acid

Zoledronic acid is a drug that prevents weakening of bones. It will be given as an injection into a vein once every 3 weeks for the first 6 injections and then once every 4 weeks until 2 years. The drug will be stopped earlier if there is a reason to suggest that your cancer is not responding to the treatment. The injection will take approximately 15 minutes. Unwanted effects are unusual although it may cause flu-like symptoms such as fever, bone pain and muscle cramps. It may also lower the level of some of the salts in your blood such as calcium and phosphate; blood tests will be needed to monitor these levels.

Docetaxel

Docetaxel will be given as an injection into a vein once every three weeks, for a total of six injections, on the same day that you receive your zoledronic acid injection. In addition, you will be given prednisolone (steroid tablets) to be taken twice a day while you are receiving the docetaxel. Docetaxel will be given as an injection into a vein once every three weeks, for a total of six injections. In addition, you will be given prednisolone (steroid tablets) to be taken twice a day while you are receiving the docetaxel. The docetaxel injection will take approximately one hour. Unwanted effects may include nausea and vomiting (although you will be given additional medication to prevent this), hair loss, fluid retention such as lower leg swelling, allergic reactions and numbness of the hands and feet. Docetaxel may also effect some cells in your blood, causing anaemia or making you more susceptible to bruising, bleeding and infections. It will be necessary to check your blood count before each injection to be sure it is safe. If you have a temperature or become unwell while you are on docetaxel you should contact your hospital cancer team immediately. The 24 hour contact details for your hospital are given below.

<u>Treatment Summary Table</u>

What is my treatment?	How will I be given the treatment?	When, and for how long for?
Androgen Suppression	Orchidectomy or Hormone Treatment	According to Local Practice (Centres to complete)
Zoledronic Acid	15 Minute Intravenous (IV) Infusion	Once every 3 weeks for 18 weeks then<br>Once every 4 weeks for up to 2 years.
Docetaxel	1 hour Intravenous (IV) Infusion	Once every three weeks for 18 weeks.

(Blood Tests will be taken before each treatment)

The prednisolone tablets may cause indigestion, weight gain, swelling of the lower legs, increased appetite, mood changes and difficulty in sleeping.

Docetaxel, in combination with prednisone (a steroid), is approved by regulatory authorities for hormone refractory prostate cancer (cancer that is no longer responsive to hormone therapy).

To receive both the zoledronic acid and the docetaxel you will probably be in the hospital for about half a day.

Will my doctor be paid if I participate?

There will be no payment to your doctor if you choose to participate in the trial. However, the trial is part of the National Cancer Research Network (NRCN) portfolio of trials. This means your doctor will be entitled to research support for the trial (for example, help from a research nurse to run the trial).

Will I be paid for participating?

There will be no payments to patients who agree to participate in this trial and additional travel expenses will not be reimbursed.

What if I change my mind about participating in the study?

If you change your mind about taking part in the study, you can withdraw from the trial at any time. This will not affect your relationship with the doctors and nurses, or your subsequent care, in any way. Even though you would not be taking part in the trial in this case, we would still like to monitor your progress. If you agree we would like to continue to collect some information so that the long-term effects of your treatment can be assessed.

Who is organising this trial?

The Medical Research Council (MRC) has overall responsibility for this trial. The study is funded by the Clinical Trials Advisory and Awards Committee which allocates money from MRC and Cancer Research UK. Some financial support has also been obtained from Novartis and the newer drugs have been supplied by the Pharmaceutical companies Novartis, Aventis and Pfiser free of charge or at a reduced cost. The study has received the favourable opinion of an Independent Research Ethics Committees. The trial is run from the MRC Clinical Trials Unit (CTU), London.

What if things go wrong?

The MRC is the sponsor of this trial and as such would give sympathetic consideration to claims for compensation for any non-negligent harm that you may suffer by participating in this trial. The MRC and NHS are both publicly funded bodies and are not allowed to purchase

advance insurance to cover indemnity because they are backed by the resources of the Treasury. Like other publicly funded bodies, any liability arising from the MRC's activities is underwritten by the UK Government. However, this does not extend to harm arising from receiving the "standard treatment".

The hospital(s) you are treated in continue to have a duty of care to you, whether or not you agree to participate in this trial. Therefore, the MRC does not accept liability for negligence on the part of employees of hospital. This applies whether the hospital is an NHS Trust or not, and the MRC cannot be held liable for any breach in the hospital's duty of care. If you wish to complain about any aspect of the way you have been approached or treated during the course of the study, the normal National Health Service complaint mechanisms will be available to you.

What if new information becomes available?

Sometimes during the course of a research project, new information becomes available about the treatment/drug that is being studied. Throughout the study, medical information from this study and any other studies will be looked at by an independent committee. If any new information becomes available that may affect your participation in this study or would affect your future care, your study doctor will tell you about it and discuss with you whether you want to continue in the study. If you decide to withdraw, your study doctor will make arrangements for your care to continue. If you decide to continue in the study you would be asked to sign an updated consent form.

Please report any unwanted effects to your cancer doctor or nurse.

If you become unwell between hospital visits, please seek advice immediately, either from your hospital team or from your GP.

Your contact numbers are:

STAMPEDE PATIENT INFORMATION SHEET – TREATMENT GROUP F

(to be printed on local hospital headed paper)

Version 1.1 (May 2005)

Information for patients receiving Hormone Treatment and zoledronic acid and celecoxib

You are going to receive hormone treatment and zoledronic acid and celecoxib. Details of the treatment are given below.

Hormone Treatment

Prostate cancers often depend upon the male hormone testosterone to grow. Reducing the amount of testosterone in the body usually prevents further growth of the cancer and may cause it to shrink. This is called hormone treatment and can be achieved either by the use of anti-hormone injections or an operation to remove part or all of both testicles, which produce the male hormone. Your doctor will discuss these different options with you and together you can decide which is the best form of hormone treatment for you. All forms of hormone treatment can cause the following side-effects: impotence, loss of libido (sexual drive), hot flushes, occasional swelling of breast tissue and absent-mindedness. In addition, if you receive the treatment over a long period of time you may notice an increase in weight, a reduction in your muscle tissues and your bones may be weakened.

Anti-hormone Injections: These injections (known as LHRH analogues) stop the production of the male hormone testosterone by the testicles. Depending on the type of injection, they are given once every month or once every three months into the skin of the abdomen or into the arm. Occasionally they temporarily aggravate the cancer before a benefit occurs and for this reason additional anti-hormone tablets are given for the first few weeks of the treatment. Other unwanted effects that have been reported are allergic reactions, irritation at the injection site and headaches. The injections usually have to continue indefinitely.

OR

Bilateral Subcapsular Orchidectomy: This is an operation where the functioning part of the testicles is removed. This is normally done by taking out the centre of the testicles, leaving the testicles themselves behind but reduced in size. Sometimes, instead of this operation the testicles are removed completely. Your surgeon will discuss the surgical options with you. These operations are usually straightforward but there will be some pain or discomfort in the scrotum afterwards. There may also be some swelling and bruising in the

scrotum that takes a couple of weeks to subside, and as with any surgical operation an infection can occur in the wound.

Zoledronic Acid

Zoledronic acid is a drug that prevents weakening of bones. It will be given as an injection into a vein once every 3 weeks for the first 6 injections and then once every 4 weeks until 2 years. The drug will be stopped earlier if there is a reason to suggest that your cancer is not responding to the treatment. The injection will take approximately 15 minutes. Unwanted effects are unusual although it may cause flu-like symptoms such as fever, bone pain and muscle cramps. It may also lower the level of some of the salts in your blood such as calcium and phosphate; blood tests will be needed to monitor these levels.

Celecoxib

The celecoxib tablets are taken twice a day and will continue for a maximum of 1 year. They will only be stopped if your cancer shows signs of not responding to the treatment or if you and your doctor decide this is best. Unwanted effects may include stomach discomfort, indigestion, abdominal pain and flatulence, fluid retention, difficulty sleeping, sinusitis or a sore throat, a rash and a small increased risk of a cardiovascular event (see below)..

Recently, information from three long-term studies of celecoxib has become available. In the first study, which was aimed at preventing rather than treating cancer, a small increased risk of heart attacks, strokes, and/or deaths resulting from heart or blood vessel disease was reported among people taking celecoxib. Approximately 1 in 100 patients in this study on placebo treatment had one of these serious events. In contrast, between 2 and 3 in 100 patients taking celecoxib (between 400 and 800 mg daily) had one of these serious events. Another clinical cancer prevention study found no increased risks in patients taking celecoxib 400 mg daily. The third study, an Alzheimer's disease prevention study, did not find increased risks with celecoxib.

Treatment Summary Table

What is my treatment?	How will I be given the treatment?	When, and for how long for?
Androgen Suppression	Orchidectomy or Hormone Treatment	According to Local Practice (Centres to complete)
Zoledronic Acid	15 Minute Intravenous (IV) Infusion	Once every 3 weeks for 18 weeks then<br>Once every 4 weeks for up to 2 years.

Celecoxib	Tablet	Two times a day for 1 year or until disease progression (which ever is sooner).

(Blood Tests will be taken before each session of Zoledronic Acid treatment)

Will my doctor be paid if I participate?

There will be no payment to your doctor if you choose to participate in the trial. However, the trial is part of the National Cancer Research Network (NRCN) portfolio of trials. This means your doctor will be entitled to research support for the trial (for example, help from a research nurse to run the trial).

Will I be paid for participating?

There will be no payments to patients who agree to participate in this trial and additional travel expenses will not be reimbursed.

What if I change my mind about participating in the study?

If you change your mind about taking part in the study, you can withdraw from the trial at any time. This will not affect your relationship with the doctors and nurses, or your subsequent care, in any way. Even though you would not be taking part in the trial in this case, we would still like to monitor your progress. If you agree we would like to continue to collect some information so that the long-term effects of your treatment can be assessed.

Who is organising this trial?

The Medical Research Council (MRC) has overall responsibility for this trial. The study is funded by the Clinical Trials Advisory and Awards Committee which allocates money from MRC and Cancer Research UK. Some financial support has also been obtained from Novartis and the newer drugs have been supplied by the Pharmaceutical companies Novartis, Aventis and Pfiser free of charge or at a reduced cost. The study has received the favourable opinion of an Independent Research Ethics Committees. The trial is run from the MRC Clinical Trials Unit (CTU), London.

What if things go wrong?

The MRC is the sponsor of this trial and as such would give sympathetic consideration to claims for compensation for any non-negligent harm that you may suffer by participating in this trial. The MRC and NHS are both publicly funded bodies and are not allowed to purchase advance insurance to cover indemnity because they are backed by the resources of the Treasury. Like other publicly funded bodies, any liability arising from the MRC's activities is underwritten by the UK Government. However, this does not extend to harm arising from receiving the "standard treatment".

The hospital(s) you are treated in continue to have a duty of care to you, whether or not you agree to participate in this trial. Therefore, the MRC does not accept liability for negligence on the part of employees of hospital. This applies whether the hospital is an NHS Trust or not, and the MRC cannot be held liable for any breach in the hospital's duty of care. If you wish to complain about any aspect of the way you have been approached or treated during the course of the study, the normal National Health Service complaint mechanisms will be available to you.

What if new information becomes available?

Sometimes during the course of a research project, new information becomes available about the treatment/drug that is being studied. Throughout the study, medical information from this study and any other studies will be looked at by an independent committee. If any new information becomes available that may affect your participation in this study or would affect your future care, your study doctor will tell you about it and discuss with you whether you want to continue in the study. If you decide to withdraw, your study doctor will make arrangements for your care to continue. If you decide to continue in the study you would be asked to sign an updated consent form.

Please report any unwanted effects to your cancer doctor or nurse.

If you become unwell between hospital visits, please seek advice immediately, either from your hospital team or from your GP.

Your contact numbers are:

Appendix C - GP Letter

Version 1.1 (May 2005)

Dear Dr __________

STAMPEDE: SYSTEMATIC THERAPY FOR ADVANCING OR METASTATIC PROSTATE CANCER

Your patient has agreed to participate in a clinical trial named STAMPEDE. This is an international study for patients with locally advanced or metastatic prostate cancer who are about to commence androgen suppression therapy. It is funded by the Clinical Trials Advisory and Awards Committee (CTAAC), sponsored by the Medical Research Council (MRC) and coordinated by the MRC Clinical Trials Unit. It will evaluate the effects of three different types of drugs that have shown promise against prostate cancer: a bisphosphonate (zoledronic acid), a chemotherapeutic agent (docetaxel) and a cyclooxygenase-2 inhibitor (celecoxib). Patients are randomised either to the control arm of the trial, androgen suppression only, or to one of five investigational arms that consist of androgen suppression plus one or two of the drugs described above.

Enclosed with this letter is a copy of the information that has been supplied to the patient about this trial. This includes a general information sheet that provides an overview of the trial and details of the general issues relating to the trial, and a separate sheet giving details about the specific treatment your patient will receive.

Your patient has been randomised to the marked arm:

- ☐ **A:** Androgen suppression
- ☐ **B:** Androgen suppression + zoledronic acid (Zometa)
- ☐ **C:** Androgen suppression + docetaxel (Taxotere)
- ☐ **D:** Androgen suppression + celecoxib (Onsenal)
- ☐ **E:** Androgen suppression + zoledronic acid (Zometa) + docetaxel (Taxotere)
- ☐ **F:** Androgen suppression + zoledronic acid (Zometa) + celecoxib (Onsenal)

Androgen suppression will be given by ☐ Bilateral orchiectomy
☐ LHRH analogues

Patients receiving docetaxel may develop myelosuppression and therefore they are at risk of neutropenic sepsis and thrombocytopaenia. Should your patient develop a fever and/or other signs of sepsis during their treatment they have been advised to contact the hospital urgently as prompt treatment with intravenous antibiotics may be life saving.

Patient's Prostate Specific Antigen (PSA) levels will be measured regularly especially in the first 6 months of the trial, and the patient's consultant may wish to discuss your surgery's possible involvement in taking blood samples.

The preferred hospital for admissions for patients with suspected neutropenic sepsis or other emergencies is:…………………………………………………………………………………….
Please contact: (telephone no):……………………………………………………………………….

For non-urgent queries relating to your patients treatment please contact the consultant, as detailed on the accompanying correspondence.

Yours sincerely,

____

Hospital Consultant

Appendix D - Molecular Genetics Sub-study

Instructions to research nurses and/or clinicians for collecting blood samples for patients who have consented to take part in the molecular genetics study.

1. Collect 8ml of blood in 4ml EDTA tubes (lilac tops) at the time of randomisation.
2. Label tube with trial number, date and time.
3. Wrap in tissue to absorb fluid in case of leakage and seal in the plastic bag supplied in the container.
4. Place in the container (addressed blue plastic safebox) together with a completed blood sample request form.
5. Fix the lid securely as requested and send by first class mail the same day.

Professor John Masters
3rd Floor research Laboratories
67 Riding House Street
London
W1W 7EJ
Tel: 020 7679 9543
Fax: 020 7679 9366
E-mail: j.masters@ucl.ac.uk

Appendix E - Drug Supply information

The pharmacist is responsible for many aspects of managing the study drug supply. This includes, where indicated in the pharmacy information sheet, ordering study directly from the drug distribution centres as needed to maintain a reasonable inventory; maintaining drug accountability records; managing the drug supply; assuring that the study drug is not accessible to unauthorized persons and that it remains stored under the correct conditions.

Study drugs can be ordered once a favourable opinion of Site Specific Assessment (SSA – formerly LREC) and Research and Development (R&D) approval have been issued for the trial to take place at that site. Copies of completed Site Accreditation Forms, SSA and R&D approval documents should be forwarded to the MRC-CTU when available. On receipt of these documents the MRC-CTU will notify the drug distributing centres of the new centre status and approve the centre for trial drug delivery. A 'starter pack' of celecoxib and of zoledronic acid will then be dispatched from the distributing centre to a named Pharmacist at the site. For finance purposes docetaxel will be ordered directly from the Pharmacy.

The starter pack of drugs will comprise of –
Docetaxel: Twelve vials of 80mg + 4 vials of 20mg
Zoledronic acid: 40 vials of 4mg
Celecoxib: 15 bottles (containing 140 capsules each) of 200mg capsules

When a new patient is randomised, the MRC-CTU will notify the relevant pharmacy of the randomisation and the treatment regimen to which the patient has been allocated and the patients treatment schedule. However, the patient will be treated with trial drugs held in stock at that site and it is the responsibility of the pharmacy to docetaxel using the docetaxel order form. Drug re-ordering for docetaxel will be left to the Lead Pharmacist at each centre. It is recommend that sufficient drugs for the treatment of newly randomised patients are ordered in a timely manner. For zoledronic acid and celecoxib, the CTU will order the drugs using a central drug monitoring and ordering database designed and maintained at CTU. Notification that drugs have been ordered will be sent to the Pharmacy. If a site find they are with out study drug for whatever reason, they can alert the CTU by sending an urgent Pharmacy request fax.

Returned or out of date stock should be destroyed in accordance with local policies and procedures. All drug destruction should be documented on the 'Destruction logs' for auditing and monitoring purposes.

Appendix F - Administration of Docetaxel and Zoledronic Acid

Administration of Docetaxel

The use of docetaxel should be confined to units specialised in the administration of cytotoxic chemotherapy and it should only be administered under the supervision of a physician qualified in the use of anticancer chemotherapy.

Pre-medication Regimen

Dexamethasone 8mg *bid* for 2 days, with the first dose taken the night before the chemotherapy.

Suggested Anti-emetic Regimen

30 min prior to the administration of docetaxel:-

- Granisetron 3mg IV stat
- Dexamethasone 8mg IV stat

Followed by:-

- Granisetron 1mg orally the morning after the docetaxel
- Domperidone 20mg orally qds as required.

Dose Modifications in the Event of Toxicity

General guidance: If possible, toxicities should be managed symptomatically. If toxicity occurs, the appropriate treatment will be used to ameliorate signs and symptoms including antiemetics for nausea and vomiting, antidiarrheals for diarrhoea, and antipyretics and/or antihistamines for drug fever.

No more than 2 dose reductions will be adopted per patient. If more than 2 dose reductions are indicated the patient should be withdrawn from trial treatment.

Docetaxel dose reductions:

Doses should be adjusted according to the following:

- Standard dose: 75 mg/m²
- First level dose reduction: 60 mg/m²
- Second level dose reduction: 45 mg/m²

Doses which have been reduced for toxicity must not be re-escalated even if the toxicity has resolved.

Docetaxel dose delay:

A treatment delay >4 days should be reported in the CRF. Treatment may be delayed no more than 2 weeks to allow recovery from acute toxicity. In case of treatment delay greater than 2 weeks, patients should be withdrawn from trial treatment.

Management of Myelosuppression

(a) Neutropaenia and its complications

Adverse event	Action to be taken
- Grade 4 neutropenia* for 7 days or more.<br>- Grade 3-4 neutropenia with oral fever $\geq$38.5°C<br>- Infection* (i.e. documented infection with grade 3-4 neutropenia)	If the patient develops one of these adverse events, the next infusion should be given with a one-level dose reduction.

*according to NCI-CTC version 3

Neutrophil count on day of infusion	Action to be taken
$\geq 1.5 \times 10^9$/L	Treat on time
$< 1.5 \times 10^9$/L	Delay maximum 2 weeks<br>Blood counts have to be performed until ANC ≥ 1.5 x 10^9/l.<br>Then treat with a one-level dose reduction.<br>If no recovery (ANC still < 1.5 x 10^9/l) after 2 week delay: the patient will be withdrawn from trial treatment.

(b) Thrombocytopaenia

In case of grade $\geq$3 platelets (NCI-CTC), delay maximum 2 weeks until platelets recover to $\geq$100 x 10^9/l, then treat with a one-level dose reduction.

Allergy and hypersensitivity reactions

Hypersensitivity reactions that occur despite pre-medication are very likely to occur *within a few minutes of start of the first or of the second infusion of docetaxel*. Therefore, during the 1st and the 2nd infusions, careful evaluation of the general sense of well being and of blood pressure and heart rate monitoring will be performed *for at least the first 10 minutes*, so that immediate intervention can occur in response to symptoms of an untoward reaction.

Facilities and equipment for resuscitation must be immediately available. If a reaction occurs, the specific treatment that is medically indicated can be given (e.g. adrenaline in case of anaphylactic shock, aminophylline in case of bronchospasm, etc.). In addition, it is recommended to take the measures listed below:

Reaction	Action to be taken
**Mild symptoms**<br>localized cutaneous reaction<br>localized pruritus<br>flushing<br>rash	Consider decreasing the rate of infusion until recovery of symptoms, stay at bedside<br>At subsequent cycles consider whether additional premedication is required see below.
**Moderate symptoms**<br>generalized pruritus<br>more severe flushing or rash<br>mild dyspnoea<br>hypotension with systolic BP≤80mmHg	Stop study drug infusion<br>Give IV antihistamine and IV corticosteroids†<br>A clinical decision should be made as to whether it is appropriate to resume the infusion after recovery of symptoms. At subsequent cycles, antihistamines† and steroids† will be given IV, one hour before infusion, in addition to the oral dexamethasone premedication Detailed below..
**Severe symptoms**<br>bronchospasm<br>generalized urticaria<br>hypotension with systolic BP≤80mmHg<br>angioedema	Stop study drug infusion<br>Give IV antihistamine and steroids†<br>add adrenaline‡ or bronchodilators and/or IV plasma expanders if indicated.

†Antihistamines: Chlorpheniramine IV 10-20 mg
Promethazine IM 25-50mg, max-100mg

Corticosteroids: dexamethasone or equivalent IV † 5-10 mg of dexamethasone

‡Adrenaline: administer standard dose - 500μg *sc*.

If a moderate/severe hypersensitivity reaction occurs careful consideration should be given as to whether it is clinically appropriate to administer another dose and the appropriate pre-medication required (IV steroids and antihistamines, and in addition dexamethasone 20 mg orally 24, 18, 13, 7 and 1 hour before study drug infusion).

Nausea/Vomiting

A prophylactic anti-emetic treatment should be given to patients from the first cycle. If despite the appropriate medication, grade ≥3 nausea/vomiting still occur, reduce the dose of study drug by one dose level. If despite dose reduction, nausea/vomiting still occur at grade ≥3, the patient should be withdrawn from trial treatment.

Diarrhoea

No prophylactic treatment for diarrhoea is recommended from cycle one. However, following the first episode of diarrhoea, the patient should receive symptomatic treatment with loperamide: 4mg following the first episode and then 2mg following each new episode until recovery of diarrhoea (no more than 16mg daily).

If despite the use of loperamide, grade ≥3 diarrhoea still occurs, reduce the dose of study drug by one dose level. If despite dose reduction, diarrhoea still occurs at grade ≥3, the patient should be withdrawn from trial treatment.

Stomatitis

Grade ≤2: No change, study chemotherapy should be withheld until resolution to grade ≤1.

Grade 3: If grade 3 stomatitis occurs, study drug should be withheld until resolution to grade ≤1. Treatment may then be resumed, but the dose of study drug should be reduced by one dose level for all subsequent doses.

Grade 4: In case of grade 4 stomatitis, the patient should be withdrawn from trial treatment.

Peripheral Neuropathy

If symptoms or signs experienced by the patient, dose modification should be performed as follows:-

Grade ≤1: no change

Grade 2: treat with a one-level dose reduction (no further dose reduction is planned)

Grade 3: patient should be withdrawn from trial treatment.

Skin Toxicity

Grade ≤2: No change

Grade 3: delay until grade ≤ 1, maximum two weeks then reduce dose of study drug by one dose level; if no recovery to ≤ grade 1 within two weeks delay, patient should be withdrawn from trial treatment.

Liver Toxicity

In case of increase of ALT and/or AST>1.5xULN or bilirubin>ULN, delay study drug treatment for up to 2 weeks until ALT and/or AST returned to ≤1.5xULN and bilirubin ≤ULN. Then treat at one level dose reduction.

Docetaxel-induced fluid retention

In case of fluid retention (peripheral oedema and/or effusions) during the treatment with docetaxel. The patient’s body weight should be recorded and followed as frequently as possible to document any weight gain, which could be related to oedema. Treatment should commence when signs and/or symptoms of fluid retention are observed, including weight gain from baseline ≥1 grade not otherwise explained. Based on the hypothesis of capillary damage due to docetaxel, the following treatment is recommended in case fluid retention occurs: frusemide 20mg po once daily.

If the symptoms cannot be controlled adequately i.e. worsening of the fluid retention or spread to another area, the dose of frusemide should be increased to 40mg. The addition of metolazone po at the recommended dose together with potassium and/or magnesium supplements may be useful.

The clinical tolerance of the patient, the overall tumour response and the medical judgment of the investigator will determine if it is in the patient's best interest to continue or to discontinue the study drug. It is recommended, however, that patients with fluid retention of grade ≥3 severity should be withdrawn from the trial.

Docetaxel-induced hyperlacrimation

The excessive lacrimation (epiphora) seen in some patients receiving docetaxel appears to be related to cumulative dose (median~300 mg/m^2) and resolves rapidly after treatment discontinuation. It seems to be the result of a chemical conjunctivitis and/or chemical inflammation (with oedema) of the lacrimal duct epithelium (producing a reversible lacrimal duct stenosis). If epiphora persists patients should be referred to an Opthalmologist.

In patients experiencing clinically significant hyperlacrimation, the following approach is recommended:

- No dose reduction planned
- Frequent instillation of artificial tears
- Prescribe a steroid ophthalmic solution (e.g. prednisolone acetate): 2 drops each eye *bid* for 3 days starting the day before docetaxel administration, in patients **without** history of herpetic eye disease.

ADMINISTRATION OF ZOLEDRONIC ACID

Zoledronic acid will be provided in plastic vials containing 4 mg zoledronic acid in 5 mL concentrate solution for infusion. Prior to administration, the 5ml of the concentrate solution must be diluted with 100 mL calcium-free infusion solution (0.9% sodium chloride solution or 5% glucose solution). The appropriate volume of the reconstituted zoledronic acid solution is 105 ml.

Zoledronic acid 4 mg/5 mL concentrate solution must not be mixed with calcium-containing solutions such as Ringer's solution.

If not used immediately after dilution with infusion media, for microbiological integrity, the final solution must be placed in a refrigerator with a temperature between 2-8 ^{0}C. The refrigerated solution should then be equilibrated to room temperature prior to administration The total time between dilution, storage in a refrigerator and end of administration of the infusion must not exceed 24 hours. Reconstituted zoledronic acid solutions must be administered in no less than a 15-minute intravenous infusion in a line separate from all other drugs.

Patients must be evaluated prior to and following the administration of the zoledronic acid infusion to ensure that they are adequately hydrated.

Since no data are available on the compatibility of zoledronic acid with other intravenously administered substances, zoledronic acid must not be mixed with other medications or substances and should always be given through a separate infusion line.

Stability studies using diluted zoledronic acid 4 mg/5 mL concentrate solutions in glass bottles and infusion bags made from polyvinylchloride (PVC), polypropylene (PP) and polyethylene (PE) prefilled with 0.9% sodium chloride solution or 5% dextrose solution and with infusion lines made from PVC and PE showed no incompatibility.

Dose Modifications

Dose reductions are not anticipated. Renal function should be closely monitored throughout the zoledronic treatment.

Zoledronic acid should be discontinued if GFR increases to $\geq$1.5 ULN. Treatment can be restarted if renal function recovers.

Serum creatinine should be evaluated prior to each zoledronic acid infusion.

- If the patient's baseline serum creatinine value was $\leq$ 126μmol/L at the time of study entry (baseline), an increase of 40μmol/L or more will require a delay of the zoledronic acid infusion until the patient's serum creatinine value returns to no higher than 10% above the baseline value.
- If the patient's baseline serum creatinine value was $\geq$ 126μmol/L, then any increase in the serum creatinine of 80μmol/L or more will require that the zoledronic acid infusion be delayed until the patient's serum creatinine value returns to no higher than 10% the baseline value.
- Any doubling of the baseline serum creatinine value will require that the study drug be delayed until the patient's serum creatinine return to no higher than 10% above the baseline value.
- Should the zoledronic acid infusions need to be delayed the patient's serum creatinine values will continue to be followed at the regularly scheduled study visits until full recovery (i.e., return to no higher than 10% above the baseline value). If the zoledronic acid infusions are delayed, other trial-related evaluations should proceed according to the protocol.

CO-ADMINISTRATION OF DOCETAXEL AND ZOLEDRONIC ACID

For patients on arm E:

In order to induce apoptosis in a synergystic fashion and for synergy to occur it has been found that cells must be re-treated with the chemotherapy agent followed directly by the bisphosphonate agent (29). Consequently docetaxel should be administered before zoledronic acid

Appendix G - Drug Safety Information for Drugs used in the Trial

ZOLEDRONIC ACID

- **Contraindications**

Zoledronic acid is contraindicated in patients with known clinically significant hypersensitivity to zoledronic acid, other bisphosphonates or any of the excipients in the formulation of Zometa.

- **Special Warnings and precautions for use**

Patients must be assessed prior to administration of zoledronic acid to ensure that they are adequately hydrated. Metabolic parameters, such as serum levels of calcium, phosphate and magnesium, should be carefully monitored after initiating zoledronic acid therapy. If hypocalcaemia, hypophosphataemia, or hypomagnesaemia occurs, short-term supplemental therapy may be necessary.

As with other bisphosphonates, zoledronic acid has been associated with reports of renal dysfunction. Factors that may increase the potential for deterioration in renal function include dehydration and pre-existing renal impairment. While the risk is reduced with a dose of zoledronic acid 4mg administered over 15 minutes, deterioration in renal function may still occur. Increases in serum creatinine also occur in some patients with chronic administration of zoledronic although less frequently.

As with other bisphosphonates renal monitoring is recommended, for instance, measurement of serum creatinine prior to each dose of zoledronic acid. In all patients the dose should be withheld if renal function has deteriorated. In clinical studies, zoledronic acid treatment was resumed only when the creatinine level returned to within 10% of the baseline value.

Overhydration should be avoided in patients at risk of cardiac failure.

- **Interaction with other medicinal products and other forms of interaction.**

In clinical studies, zoledronic acid has been administered concomitantly with commonly used anticancer agents, diuretics, antibiotics and analgesics without clinically apparent interactions occurring. Zoledronic acid shows no appreciable binding to plasma proteins and does not inhibit human P450 enzymes, but no formal clinical interaction studies have been performed. Caution is advised when bisphosphonates are administered with aminoglycosides, since both agents may have an additive effect, resulting in a lower serum calcium level for longer periods. Caution is also indicated when zoledronic acid is used with

other potentially nephrotoxic drugs. Attention should also be paid to the possibility of hypomagnesaemia developing during treatment.

- **Undesirable Effects**

Frequencies of adverse reactions for zoledronic acid 4mg are mainly based on data collection from chronic treatment. Adverse reactions to zoledronic acid are similar to those reported for other bisphosphonates and can be expected to occur in approximately one third of patients. Intravenous administration has been most commonly associated with a flu-like syndrome in about 9% of patients, including bone pain (9.1%), fever (7.2%), fatigue (4.1%) and rigors (2.9%). Arthralgia and myalgia have been reported in approximately 3%. No information is available on the reversibility of these adverse effects.

Frequently, the reduction in renal calcium excretion is accompanied by a fall in serum phosphate levels (in approximately 20% of patients), which is asymptomatic not requiring treatment. The serum calcium may fall to asymptomatic hypocalcaemic levels in approximately 3% of patients.

Gastrointestinal reactions, such as nausea (5.8%) and vomiting (2.6%) have been reported following intravenous infusion of zoledronic acid. Occasionally local reactions at the infusion site such as redness or swelling and/or pain were also observed in less than 1% of the patients.

Anorexia was reported in 1.5% of patients treated with zoledronic acid 4mg. Few cases of rash or pruritus have been observed (below 1%). As with other bisphosphonates, cases of conjunctivitis in approximately 1% have been reported. There have been some reports of impaired renal function (2.3%), although the aetiology appears to be multifactorial in many cases. Based on pooled analysis of placebo controlled studies, severe anaemia (Hb<8.0g/dl) was reported in 5.2% of patients receiving zoledronic acid 4mg versus 4.2% on placebo.

Other adverse drug reactions are listed in Table G.2.

- **Overdose**

There is no experience of acute intoxication with zoledronic acid. Patients who have received doses higher than those recommended should be carefully monitored. In the event of clinically significant hypocalcaemia, reversal may be achieved with an infusion of calcium gluconate.

DOCETAXEL

- **Contraindications**

Docetaxel is contraindicated in those patients with a known hypersensitivity to docetaxel or to any of the excipients and should not be used in patients with absolute neutrophil counts <1.5 x 10^9/l or if there is severe liver impairment.

- **Special Warnings and Precautions**

A premedication consisting of an oral corticosteroid is required (see **Appendix F**).

Neutropenia is the most frequent adverse reaction of docetaxel. Neutrophil nadirs occur at a median of seven days. Frequent monitoring of complete blood counts should be conducted on all patients receiving docetaxel.

In the case of severe neutropenia (<500 cells/mm^3 for seven days or more) during a course of docetaxel therapy, a reduction in dose for subsequent courses is recommended (see **Appendix F**).

- **Hypersensitivity reactions**

Patients should be observed closely for hypersensitivity reactions especially during the first and second infusions. Hypersensitivity reactions may occur within a few minutes following the initiation of the infusion of docetaxel, thus facilities for the treatment of hypotension and bronchospasm should be available. If hypersensitivity reactions occur, minor symptoms such as flushing or localised cutaneous reactions do not require interruption of therapy. However, severe reactions, such as severe hypotension, bronchospasm or generalised rash/erythema require immediate discontinuation of docetaxel and appropriate therapy, (see **Appendix F**). Patients who have developed severe hypersensitivity reactions should not be re-challenged with docetaxel.

- **Cutaneous reactions**

Localised skin erythema of the extremities (palms of the hands and soles of the feet) with oedema followed by desquamation has been observed. Severe symptoms such as eruptions followed by desquamation which lead to interruption or discontinuation of docetaxel treatment have been reported

- **Interaction with other medicinal products and other forms of interaction**

In vitro studies have shown that the metabolism of docetaxel may be modified by the concomitant administration of compounds which induce, inhibit or are metabolised by (and thus may inhibit the enzyme competitively) cytochrome P450-3A such as cyclosporine, terfenadine, ketoconazole, erythromycin and troleandomycin. As a result, caution should be

exercised when treating patients with these drugs as concomitant therapy since there is a potential for a significant interaction.

Undesirable effects

- **Blood disorders**

The most commonly reported adverse reaction was neutropenia, which was reversible and not cumulative. The median time to nadir was 7 days and the median duration of severe neutropenia (<500 cells/mm^3) was 7 days.

Bone marrow suppression and other hematological adverse reactions have also been reported.

- **Skin and subcutaneous tissue disorders**

Reversible cutaneous reactions have been observed and were generally considered as mild to moderate. Reactions were characterised by a rash including localised eruptions mainly on the feet and hands, but also on the arms, face or thorax, and frequently associated with pruritus. Eruptions generally occurred within one week after the docetaxel infusion. Less frequently, severe symptoms such as eruptions followed by desquamation which rarely lead to interruption or discontinuation of docetaxel treatment were seen. Severe nail disorders are characterised by hypo- or hyperpigmentation and sometimes pain and onycholysis. Very rare cases of bullous eruption such as erythema multiforme or Stevens-Johnson syndrome have been reported with docetaxel and other concomitant factors may have contributed to the development of these effects.

- **Fluid retention**

Events such as peripheral oedema and less frequently pleural effusion, pericardial effusion, ascites and weight gain have been reported. The peripheral oedema usually starts at the lower extremities and may become generalised with a weight gain of 3 kg or more. Fluid retention is cumulative in incidence and severity. Fluid retention has not been accompanied by acute episodes of oliguria or hypotension. Dehydration and pulmonary oedema have rarely been reported.

- **Gastrointestinal disorders**

Nausea, Stomatitis, Vomiting, Diarrhoea and Constipation may occur.

- **Nervous system disorders**

The development of severe peripheral neurotoxicity requires a reduction of dose (see **Appendix F**). Mild to moderate neuro-sensory signs are characterised by paraesthesia,

dysaesthesia or pain including burning. Neuro-motor events are mainly characterised by weakness.

- **General disorders and administration site conditions**

Infusion site reactions were generally mild and consisted of hyperpigmentation, inflammation, redness or dryness of the skin, phlebitis or extravasation and swelling of the vein.
Other undesirable effects are described in table G.2.

- **Overdose**

There were a few reports of overdose. There is no known antidote for docetaxel overdose. In case of overdose, the patient should be kept in a specialised unit and vital functions closely monitored. The primary anticipated complications of overdose would consist of bone marrow suppression, peripheral neurotoxicity and mucositis. Patients should receive therapeutic G-CSF as soon as possible after discovery of overdose. Other appropriate symptomatic measures should be taken, as needed.

CELECOXIB

- **Contraindications**

Celecoxib is contraindicated if the patient has a known hypersensitivity to the active substance or to any of the excipients or sulphonamides. It is also contraindicated in patients who have experienced asthma, acute rhinitis, nasal polyps, angioneurotic oedema, urticaria or other allergic-type reactions after taking acetylsalicylic acid or NSAIDs.

It is also contraindicated if the patient has active peptic ulceration, gastrointestinal bleeding, inflammatory bowel disease, renal insufficiency, severe congestive heart failure (NYHA II-IV), established ischaemic heart disease and/or cerebrovascular disease and severe hepatic disease.

- **Special Warnings and Precautions for use**

Upper gastrointestinal complications [perforations, ulcers or bleeds (PUBs)], some of them resulting in fatal outcome, have occurred in patients treated with celecoxib. Caution is advised with treatment of patients most at risk of developing a gastrointestinal complication with NSAIDs: the elderly, patients using any other NSAID or acetylsalicylic acid concomitantly or patients with a prior history of gastrointestinal disease, such as ulceration and GI bleeding. There is further increase in the risk of gastrointestinal adverse effects, when celecoxib is taken concomitantly with acetylsalicylic acid (even at low doses).

As with other medicinal products known to inhibit prostaglandin synthesis fluid retention and oedema have been observed in patients taking celecoxib. Therefore, celecoxib should be used with caution in patients with history of cardiac failure, left ventricular dysfunction or hypertension, and in patients with pre-existing oedema from any other reason, since prostaglandin inhibition may result in deterioration of renal function and fluid retention. Caution is also required in patients taking diuretic treatment or otherwise at risk of hypovolaemia.

A small Increase in the number of serious cardiovascular events, mainly myocardial infarction, has been found in a long-term placebo-controlled study in subjects with sporadic adenomatous polyps treated with celecoxib at doses of 200 mg BID and 400 mg BID compared to placebo. Patients with significant risk factors for cardiovascular events (e.g. hypertension, hyperlipidaemia, diabetes mellitus, smoking) or peripheral arterial disease should only be treated with celecoxib after careful consideration
In the event of elderly patients with mild to moderate cardiac dysfunction requiring therapy, special care and follow up is warranted.

Experience with celecoxib in patients with mild or moderate renal or hepatic impairment is limited, therefore such patients should be treated with caution. If during treatment, patients deteriorate in any of the organ system functions described above, appropriate measures should be taken and discontinuation of celecoxib therapy should be considered.
In patients on concurrent therapy with warfarin, serious bleeding events have occurred. Caution should be exercised when combining celecoxib with warfarin (See Interactions).

Onsenal 200 mg capsules contain lactose (49.8 mg). Patients with rare hereditary problems of galactose intolerance, the Lapp lactase deficiency or glucose-galactose malabsorption should not take this medicine.

- **Interaction with other medicinal products and other forms of interaction.**

Pharmacodynamic interactions

Anticoagulant activity should be monitored in patients taking warfarin or other anticoagulants, particularly in the first few days after initiating or changing the dose of celecoxib, since these patients have an increased risk of bleeding complications. Therefore, patients receiving oral anticoagulants should be closely monitored for their prothrombin time INR. Bleeding events in association with increases in prothrombin time have been reported, in arthritis patients (mainly elderly) receiving celecoxib concurrently with warfarin, some of them fatal

NSAIDs may reduce the effect of diuretics and antihypertensive drugs. As for NSAIDs, the risk of acute renal insufficiency may be increased when ACE inhibitors are combined with celecoxib. Therefore, the combination should be administered with caution, especially in the elderly. Patients should be adequately hydrated and consideration should be given to monitoring of renal function after initiation of concomitant therapy, and periodically thereafter.

Coadministration of NSAIDs and cyclosporin or tacrolimus have been suggested to increase the nephrotoxic effect of cyclosporin and tacrolimus. Renal function should be monitored when celecoxib and any of these drugs are combined.

Celecoxib can be used with low dose acetylsalicylic acid but is not a substitute for acetylsalicylic acid for cardiovascular prophylaxis. As with other NSAIDs, an increased risk of gastrointestinal ulceration or other gastrointestinal complications compared to use of celecoxib alone was shown for concomitant administration of low-dose acetylsalicylic acid.

Pharmacokinetic interactions

- *Effects of celecoxib on other drugs*

Celecoxib is a weak inhibitor of CYP2D6. During celecoxib treatment, the plasma concentrations of the CYP2D6 substrate dextromethorphan were increased by 136%. The plasma concentrations of drugs that are substrates of this enzyme may be increased when celecoxib is used concomitantly. Examples of drugs which are metabolised by CYP2D6 are antidepressants (tricyclics and SSRIs), neuroleptics, anti-arrhythmic drugs, etc. The dose of individually dose-titrated CYP2D6 substrates may need to be reduced when treatment with celecoxib is initiated or increased if treatment with celecoxib is terminated.

In vitro studies have shown some potential for celecoxib to inhibit CYP2C19 catalysed metabolism. The clinical significance of this *in vitro* finding is unknown. Examples of drugs which are metabolised by CYP2C19 are diazepam, citalopram and imipramine.

In healthy subjects, co-administration of celecoxib 200mg twice daily with 450mg twice daily of lithium resulted in a mean increase in Cmax of 16% and in AUC of 18% of lithium. Therefore, patients on lithium treatment should be closely monitored when celecoxib is introduced or withdrawn.

- *Effects of other drugs on celecoxib*

Since celecoxib is predominantly metabolised by CYP2C9 it should be used at half the recommended dose in patients receiving fluconazole. Concomitant use of 200mg single dose of celecoxib and 200mg once daily of fluconazole, a potent CYP2C9 inhibitor, resulted in a mean increase in celecoxib Cmax of 60% and in AUC of 130%. Concomitant use of inducers of CYP2C9 such as rifampicin, carbamazepine and barbiturates may reduce plasma concentrations of celecoxib.

Ketoconazole or antacids have not been observed to affect the pharmacokinetics of celecoxib.

- **Undesirable Effects**

The common reactions, those occurring in less than 1 in 10 patients are listed below. Other reactions are summarised in Table G.2.

<u>Common</u>
Body as a whole: peripheral oedema/ fluid retention.
Cardiac: myocardial infarction
Gastrointestinal: abdominal pain, diarrhoea, dyspepsia, flatulence.
Nervous system: dizziness.
Psychiatric: insomnia.
Respiratory: pharyngitis, rhinitis, sinusitis, upper respiratory tract infection.

Skin: rash.

- **Overdose**

There is no clinical experience of overdose. Single doses up to 1,200 mg and multiple doses up to 1,200 mg twice daily have been administered to healthy subjects for nine days without clinically significant adverse effects. In the event of suspected overdose, appropriate supportive medical care should be provided e.g. by eliminating the gastric contents, clinical supervision and, if necessary, the institution of symptomatic treatment. Dialysis is unlikely to be an efficient method of drug removal due to high protein binding.

Table G.2: Comparative table of Undesirable effects of Docetaxel, Zoledronic Acid and Celecoxib

Adverse reactions listed using the following convention:

- very common (>1/10)
- common (>1/100, <1/10)
- uncommon (>1/1,000, <1/100)
- rare (>1/10,000, <1/1,000)
- very rare (<1/10,000).

System	Symptom	Docetaxel	Celecoxib	Zoledronic acid
Allergic Reactions	Anaphylactic shock	-	Very rare	-
	Angioedema	-	Very rare	Rare
	Hypersensitivity reactions	Common	-	Uncommon
	Myositis	-	Very rare	-
	Severe allegic reactions	-	Very rare	-
	Vasculitis	-	Very rare	-
Auditory	Decreased hearing	-	Very rare	-
	Tinnitus	-	Uncommon	-
Blood & Lymph	Anaemia	Very common	Uncommon	Common
	Febrile neutropaenia	Common	-	-
	Leukopaenia	-	Rare	Uncommon
	Neutropaenia	Very common	-	-
	Pancytopaenia	-	Very rare	Rare
	Thrombocytopaenia	Very common	Rare	Uncommon
Cardiac Disorders	Bradycardia	-	-	Rare
	Cardiac dysrhythmia	Common	Uncommon	-
	Congestive heart failure	-	Very rare	
	Heart failure	-	Uncommon	-
	Hypertension	-	Uncommon	Uncommon
	Hypotension	Common	-	-
	Ischaemic stroke	-	Uncommon	-
	Myocardial infarction	-	Common	-
Gastrointestinal Disorders	Acute pancreatitis	-	Very rare	-
	Abdominal pain	-	Common	Uncommon
	Constipation	Common	Uncommon	Uncommon
	Diarrhoea	Very common	Common	Uncommon
	Dry mouth	-	-	Uncommon
	Duodenal/gastric/ oesophageal ulceration	-	Rare	-
	Dyspepsia	-	Common	Uncommon
	Dysphagia	-	Rare	-
	Eructation	-	Uncommon	-
	Flatulence	-	Common	-
	Gastritis	-	Uncommon	-
	GI haemorrhage	-	Very rare	-
	Intestinal perforation	-	Rare	-
	Melaena	-	Rare	-
	Nausea	Very common	-	Common
	Oesophagitis	-	Rare	-
	Stomatitis	Very common	Uncommon	Uncommon
	Vomiting	Very common	Uncommon	Common
General Disorders	Asthenia	Very common	-	Uncommon
	Chest pain	-	-	Uncommon
	Fever	-	-	Common
	Flu-like symptoms	-	-	Common
	Injection site reactions	-	-	Uncommon
	Lethargy	Common	Uncommon	-
	Oral candidiasis	Common	-	-
	Pain	Very common	-	-
	Pain in limb	Common	Uncommon	-
	Radiation recall phenomena	Rare	-	-
	Weight increase	-	-	Uncommon
Hepatic Disorders	Abnormal hepatic function	-	Uncommon	-
	G3/4 Bilirubin increase (<2%)	Common	-	-
	Hepatitis	-	Very rare	-
	Increased SGOT	-	Uncommon	-
	Increased SGPT	-	Uncommon	-

System	Symptom	Docetaxel	Celecoxib	Zoledronic acid
	Jaundice	-	Very rare	-
Laboratory Abnormalities	Hyperkalaemia	-	-	Rare
	Hypernatraemia	-	-	Rare
	Hypocalcaemia	-	-	Common
	Hypokalaemia	-	-	Uncommon
	Hypomagnesaemia	-	-	Uncommon
	Hypophosphataemia	-	-	Very Common
Metabolism and Nutrition Disorders	Abnormal renal function tests (increased creatinine, BUN, hyerkalaemia)	-	Uncommon	Common
	Anorexia	Very common	-	Common
	Taste alteration	-	Rare	Uncommon
Musculoskeletal Disorders	Arthralgia	-	-	Common
	Bone pain	-	-	Common
	Generalised pain	-	-	Common
	Muscle cramps	-	-	Uncommon
	Myalgia	Common	Uncommon	Common
Nervous System Disorders	Ataxia	-	Rare	-
	Aggravated epilepsy	-	Very rare	-
	Dizziness	-	Common	Uncommon
	Headache	-	-	Common
	Hyperaesthesia	-	-	Uncommon
	Hypertonia	-	Uncommon	-
	Hypoaesthesia	-	-	Uncommon
	Paraesthesia	-	Uncommon	Uncommon
	Tremor	-	-	Uncommon
Occular	Blurred vision	- Common	Uncommon	Uncommon
	Conjunctivitis	-	-	Common
Peripheral Oedema/Fluid Retention	Fluid retention	Very common	Common	Uncommon
Psychiatric Disorders	Anxiety	-	Uncommon	Uncommon
	Confusion	-	Very rare	Rare
	Depression	-	Uncommon	-
	Hallucinations	-	Very rare	-
	Insomnia	-	Common	Uncommon
Renal	Abnormal renal function tests (increased creatinine, BUN, hyerkalaemia)	-	Uncommon	Common
	Acute renal failure	-	Very rare	Uncommon
	Haematuria	-	-	Uncommon
	Interstitial nephritis	-	Very rare	-
	Proteinuria	-	-	Uncommon
	Renal impairment	-	-	Common
	Urinary tract infection	-	Uncommon	-
Respiratory Disorders	Bronchospasm	-	Very rare	-
	Coughing	-	Uncommon	Uncommon
	Dyspnoea	-	Uncommon	Uncommon
	Pharyngitis	-	Common	-
	Rhinitis	-	Common	-
	Sinusitis	-	Common	-
	Upper respiratory tract infection	-	Common	-
Skin/Subcutaneous Tissue Disorders	Alopecia	Very common	Rare	-
	Cutaneous reaction	Very common		-
	Epidermal necrolysis	-	Very rare	-
	Erythema multiforme	-	Very rare	-
	Increased sweating	-	-	Uncommon
	Nail changes	Common	-	-
	Photosensitivity	-	Rare	-
	Pruritus	-	-	Uncommon
	Rash	-	Common	Uncommon
	Stevens-Johnson syndrome	-	Very rare	-
	Urticaria	-	Uncommon	-

Appendix H - Health Care at Home Study

The possibility of Zoledronic acid infusions being administered at home if patients prefer, by trained nurses, is currently being discussed with Novartis.

Appendix I - Evaluation of Baseline Lesions and New Lesions

These guidelines are based on the RECIST criteria (43) but have been modified to include progression based on PSA measurements.

Measurable Disease

Measurable disease is defined as at least one lesion that can be accurately measured in at least one dimension (longest dimension to be recorded). Each lesion must $\geq$20mm when measured by conventional techniques, including palpation, CT, and MRI, or $\geq$10mm when measured by spiral CT.

Baseline documentation of "Target" and Non-Target lesions

All measurable lesions up to a maximum of 5 lesions per organ and 10 lesions in total representative of all involved organs should be identified as target lesions and will be recorded and measured at baseline. Target lesions should be selected on the basis of their size (lesions with the longest dimension) and their suitability for accurate repetitive measurements by one consistent method of assessment (either by imaging techniques or clinically). A sum of the longest dimension (LD) for all target lesions will be calculated and reported as the baseline sum LD. The baseline sum LD will be used as reference to further characterise

All other lesions (or sites of disease) should be identified as non-target lesions and should also be recorded at baseline. Measurements are not required.

All baseline evaluations of disease status should be performed as close as possible to the start of the treatment and not more than 4 weeks before the beginning of the treatment.

Definition of Progression

(for patients with measurable disease at randomisation)

Progression is defined as ANY of the following:-

- At least a 20% increase in the sum of LD target lesions taking as reference the smallest sum LD recorded since study entry
- The appearance of one or more new lesions
- Death due to disease without prior objective documentation of progression
- Global deterioration in health status attributable to the disease requiring a change in therapy without objective evidence of progression

- Unequivocal progression of non-target lesions, (other than pleural effusions without cytological proof of neoplastic origin) in the opinion of the treating physician (in this case an explanation must be provided).
- Progression will be most usually based on PSA measurements (see **Appendix K**) but tumour measurements should take precedence over PSA response. If measurable disease is shrinking during treatment, but the PSA is rising the patient should continue to receive protocol treatment.

Definition of Progression
(for patients with non-measurable disease at randomisation)

Progression (for patients with non-measurable disease at randomisation) is defined as increasing clinical or radiological evidence of disease since study entry. Progression can also be based on PSA measurements (**Appendix K**).

Appendix J - Common Toxicity Criteria

The following table shows the known side effects for the drugs being used in STAMPEDE and how they relate to the CTCv3 Toxicity heading. The CTC v3 toxicity grade heading/adverse event column states first the broad classification of AEs based on anatomy and/or pathophysiology (category)_and then the adverse event term the symptom falls within. The complete CTCv3 can be found at the following web site - http://ctep.cancer.gov/reporting/ctc.html

System	Symptom	CTC v3 Toxicity Grade Heading/Adverse Event
Allergic Reactions	Anaphylactic shock	Allergy/Immunology, Allergic reaction/hypersensitivity
	Angioedema	Dermatology, Urticaria
	Hypersensitivity reactions	Allergy/Immunology, Allergic reaction/hypersensitivity
	Myositis	Musculoskeletal, Myositis
	Severe allegic reactions	Allergy/Immunology, Allergic reaction/hypersensitivity
	Vasculitis	Allergy/Immunology, Allergic reaction/hypersensitivity
Auditory	Decreased hearing	Auditory/Ear, Hearing
	Tinnitus	Auditory/Ear, Tinnitus
Blood & Lymph	Anaemia	Blood/Bone Marrow, Haemoglobin
	Febrile neutropaenia	Infection, Febrile neutropaenia
	Leukopaenia	Blood/Bone Marrow, Leukocytes
	Bone Marrow depletion	Blood/Bone Marrow, Bone marrow cellularity
	Infection	Infection, Other *(Specify)*
	Neutropaenia	Blood/Bone Marrow, Neutrophils
	Other Haematology	Blood/Bone Marrow, Other *(Specify)*
	Pancytopaenia	Blood/Bone Marrow, See Haemoglobin, Leukocytes, Platelets
	Thrombocytopaenia	Blood/Bone Marrow, Platelets
Cardiac Disorders	Bradycardia	Cardiac Arrhythmia, Supraventricular and nodal arrhythmia
	Cardiac dysrhythmia/Palpitations	Cardiac Arrhythmia, Cardiac arrhythmia
	Congestive heart failure	Cardiac General, Cardiac ischemia/infarction
	Heart failure	Cardiac General, Cardiac ischemia/infarction
	Hypertension	Cardiac General, Hypertension
	Hypotension	Cardiac General, Hypotension
	Myocardial infarction	Cardiac General, Cardiac ischemia/infarction
	Pericardial effusion	Cardiac General, Pericardial effusion
	Venous thromboembolitic events	Vascular, Thrombosis/thrombus/embolism
Gastrointestinal Disorders	Ascites	Gastrointestinal, Ascites
	Acute pancreatitis	Hepatobiliary/Pancreas, Pancreatitis

System	Symptom	CTC v3 Toxicity Grade Heading/Adverse Event
	Abdominal pain	Pain, Abdomen
	Colitis	Gastrointestinal, Colitis
	Constipation	Gastrointestinal, Constipation
	Dehydration	Gastrointestinal, Dehydration
	Diarrhoea	Gastrointestinal, Diarrhoea
	Dry mouth	Gastrointestinal, Dry mouth
	Ulceration (Duadenal/gastric/ Oesophageal)	Gastrointestinal, Ulcer *(Select site)*
	Dyspepsia	Gastrointestinal, Dyspepsia
	Dysphagia	Gastrointestinal, Dysphagia
	Eructation	Gastrointestinal, Dyspepsia
	Flatulence	Gastrointestinal, Flatulence
	Gastritis	Gastrointestinal, Gastritis
	GI haemorrhage	Haemorrhage/Bleeding, GI
	Intestinal perforation	Gastrointestinal, Perforation GI *(Select site)*
	Melaena	Haemorrhage/Bleeding, GI
	Nausea	Gastrointestinal, Nausea
	Neutropaenic enterocolitis	Infection, GI
	Obstruction	Gastrointestinal, Obstruction GI *(Select site)*
	Oesophagitis	Gastrointestinal, Oesophagitis
	Stomatitis	Gastrointestinal, Mucositis/Stomatitis
	Vomiting	Gastrointestinal, Vomiting
General Disorders	Asthenia	Constitutional Symptoms, Asthenia
	Back pain	Pain, Back
	Chest pain	Pain, Chest
	Chills	Constitutional Symptoms, Rigors/chills
	Fever	Constitutional Symptoms, Fever
	Flu-like symptoms (including fatigue, rigors, malaise and flushing)	Syndromes, Flu-like syndrome
	Injection/administration site reactions (including pain, irritation, swelling and induration)	Dermatology/Skin, Injection site reaction/extravasation changes
	Lethargy	Constitutional Symptoms, Fatigue
	Oral candidiasis	Infection, Oral cavity
	Pain	Pain, *(Select site)*
	Pain in limb	Pain, Extremity – limb
	Radiation recall phenomena	Dermatology/Skin, Rash (chemo/radiation)

System	Symptom	CTC v3 Toxicity Grade Heading/Adverse Event
	Weight increase (see fluid retention)	Constitutional Symptoms, Weight gain
Hepatic Disorders	Abnormal hepatic function	Hepatobiliary/Pancreas, Other *(Specify)*
	Bilirubin increase	Metabolic/Laboratory, Bilirubin
	Hepatitis	Infection, Viral hepatitis/Infection *(Select)*
	Increased SGOT	Metabolic/Laboratory, AST, SGOT (serum glutamic oxaloacetic transaminase)
	Increased SGPT	Metabolic/Laboratory, ALT, SGPT (serum glutamic pyruvic transaminase)
	Jaundice	Metabolic/Laboratory, Bilirubin
Laboratory Abnormalities	Hyperkalaemia	Metabolic/Laboratory, Hyperkalaemia
	Hypernatraemia	Metabolic/Laboratory, Hypernatraemia
	Hypocalcaemia	Metabolic/Laboratory, Hypocalcaemia
	Hypokalaemia	Metabolic/Laboratory, Hypokalaemia
	Hypomagnesaemia	Metabolic/Laboratory, Hypomagnesaemia
	Hypophosphataemia	Metabolic/Laboratory, Hypophosphataemia
Metabolism and Nutrition Disorders	Abnormal renal function tests (increased creatinine, Blood Urea Nitrogen, hyerkalaemia)	Renal/Genitourinary, Other *(Specify)*<br>See also:<br>Metabolic/Laboratory, Creatinine/hyperkalaemia/hyperuricaemia
	Anorexia	Gastrointestinal, Anorexia
	Taste alteration	Gastrointestinal, Taste alteration
Musculoskeletal Disorders	Arthralgia	Pain, Joint
	Bone pain	Pain, Bone
	Generalised pain	Pain, *(Select site)*
	Muscle cramps	Pain, Muscle
	Myalgia	Pain, Muscle
Nervous System Disorders	Ataxia	Neurology, Ataxia
	Aggravated epilepsy	Neurology, Seizure
	Burning pain	Neurology, Neuropathy sensory
	Convulsion	Neurology, Seizure
	Dizziness	Neurology, Dizziness
	Dysaethesia	Neurology, Neuropathy sensory
	Headache	Pain, Headache
	Hyperaesthesia	Neurology, Neuropathy sensory
	Hypertonia	Neurology, Pyramidal tract dysfunction
	Hypoaesthesia	Neurology, Neuropathy sensory
	Loss of Conciousness (transient)	Neurology, Syncope
	Paraesthesia	Neurology, Neuropathy sensory

System	Symptom	CTC v3 Toxicity Grade Heading/Adverse Event
	Tremor	Neurology, Tremor
	Weakness	Neurology, Neuropathy motor
Occular	Blurred vision	Ocular/Visual, Blurred vision
	Conjunctivitis	Infection, Eye
	Lacrimal duct obstruction	Ocular/Visual, Other (*Specify)*
	Visual disturbance	Ocular/Visual, Blurred vision, Diplopia, Flashing lights
Peripheral Oedema/Fluid Retention	Fluid retention	Lymphatics, Oedema, limb
Psychiatric Disorders	Anxiety	Neurology, Mood alteration
	Confusion	Neurology, Confusion
	Depression	Neurology, Mood alteration
	Hallucinations	Neurology, Psychosis
	Insomnia/sleep disturbace	Constitutional Symptoms, Insomnia
Renal	Abnormal renal function tests (increased creatinine, Blood Urea Nitrogen, hyerkalaemia)	Renal/Genitourinary, Other *(Specify)*<br>See also:<br>Metabolic/Laboratory, Creatinine/hyperkalaemia/hyperuricaemia
	Acute renal failure	Renal/Genitourinary, Renal failure
	Haematuria	Haemorrhage/Bleeding, GU
	Interstitial nephritis	Infection, Kidney
	Proteinuria	Metabolic/Laboratory, Proteinuria
	Renal impairment	Renal/Genitourinary, Other *(Specify)*
	Urinary tract infection	Infection, Urinary tract
Respiratory Disorders	Bronchospasm	Pulmonary/Upper Respiratory, Bronchospasm
	Chest tightness	Pain, Chest/thorax
	Coughing	Pulmonary/Upper Respiratory, Cough
	Dyspnoea	Pulmonary/Upper Respiratory, Dyspnoea
	Pharyngitis	Infection, Pharynx
	Pleural effusion	Pulmonary/Upper Respiratory, Pleural effusion
	Pulmonary oedema	Cardiac General, Left ventricular diastolic dysfunction, Left ventricular systolic dysfunction
	Rhinitis	Infection, Nose
	Sinusitis	Infection, Sinus
	Upper respiratory tract infection	Infection, Upper airway
Skin/Subcutaneous Tissue Disorders	Alopecia	Dermatology/Skin, Alopecia
	Cutaneous reaction	Dermatology/Skin, Rash
	Desquamation	Dermatology/Skin, Rash
	Epidermal necrolysis	Dermatology/Skin, Erythema multiforme etc.
	Erythema multiforme	Dermatology/Skin, Erythema multiforme etc.

System	Symptom	CTC v3 Toxicity Grade Heading/Adverse Event
	Flushing	Dermatology/Skin, Flushing
	Increased sweating	Constitutional Symptoms, Sweating
	Nail changes	Dermatology/Skin, Nail changes
	Photosensitivity	Dermatology/Skin, Photosensitivity
	Pruritus	Dermatology/Skin, Pruritis
	Rash	Dermatology/Skin, Rash
	Stevens-Johnson syndrome	Dermatology/Skin, Erythema multiforme etc.
	Urticaria	Dermatology/Skin, Urticaria

Appendix K - Definition of Biochemical Failure

Biochemical failure: The initial response to hormonal therapy for prostate cancer can be variable. A few patients exhibit only a small fall in PSA, with little evidence of clinical response. At the other extreme a rapid fall to the normal range or even undetectable levels of PSA occurs. In a group of patients the response lies between these two extremes. The rate of fall of PSA and the level of the PSA nadir are recognised to have prognostic significance. Comparison of PSA responses between the treatment groups could be used as secondary data for confirming the response rate of the trial treatments. However, in defining PSA relapse, the extent of the primary response has to be taken into account. Three groups of patients will be defined:-

A. If the PSA nadir is more than 50% of the last pre-treatment PSA, the patient should be defined as a treatment failure (at time zero).

B. For patients whose PSA falls by more than 50% of the last pre-treatment PSA, but remains above 4ng/ml, PSA relapse will be deemed to have occurred when PSA is confirmed as increasing by 50% above the nadir level.

C. For patients whose PSA falls below 4ng/ml, PSA relapse will be defined by either 50% increase from their nadir or the PSA increasing above 4, whichever is the greater. For example, a nadir PSA of 3.6 would require a PSA of 5.4 to define relapse, while PSA nadir of 2.5 will be considered to have relapsed at a PSA of 4.

Timing of PSA tests: All patients will commence hormone therapy shortly after randomisation. The final pre-randomisation PSA must be measured within 4 weeks prior to

randomisation. Once on trial, PSA tests will be performed at 6, 12, 18 and 24 weeks from randomisation then at each follow-up visit (3 monthly until 3 years, then 6 monthly).

Nadir PSA: The PSA nadir will be the lowest reported PSA level between randomisation and the 24 weeks PSA assessment (i.e. assessments at 6, 12, 18 and 24 weeks). The critical value that would constitute subsequent biochemical progression will be calculated from this nadir value. Once the 6 month PSA level has been recorded and sent promptly to the MRC CTU, a letter will be sent to the responsible clinician confirming the PSA level which would be taken as progression.

Confirming failure-free survival (including biochemical failure): In applying the definitions of Failure-free survival (including biochemical failure) above, the increase in PSA must be confirmed (i.e. at least two readings are required). The following approach will be applied:

1. Patient reaches nadir PSA and continues with 3-monthly PSA assessments. [Any PSA measurement taking place within 6 weeks of a change in hormone therapy or a manipulation (e.g. cystoscopy) that could produce a spurious increase in PSA is to be ignored].
2. At a subsequent assessment (say, assessment X), the PSA value is noted to have risen above the critical value, but patient is symptom free.
3. Patient should be recalled for a confirmatory PSA test between one week and 3 months later. If this value is at the same level or higher, the patient will be considered to have reached the biochemical failure endpoint.

Confirmatory step 3 will not be required if the patient has demonstrable local progression, new metastases or death from prostate cancer during the intervening period.

Time to failure-free survival (including biochemical failure): Patients in Group A (treatment failure) will be recorded as events at time zero. The time to FFS for patients in Groups B and C will be from randomisation to the first PSA above the critical value (i.e. assessment X).

Non-protocol treatment: No new non-protocol treatment for prostate cancer should be administered until trial progression has been confirmed.

PSA Monitoring Group: Consideration will be given to the formation of such a group, whose remit would be to monitor the accurate recording of PSA data, and independently assess failure outcomes, blinded of the treatment allocation. This would be, by definition, an independent group involving clinicians not recruiting patients to STAMPEDE.

Appendix L - Trial Contacts

Trial Management Group

Mr Noel Clarke
Consultant Urologist
Department of Urology
Hope Hospital
Salford Royal Hospitals Trust
Stott Lane
Salford
M6 8HD
T: 0161 787 5568
F: 0161 787 5814
E: nclarke@hope.srht.nwest.nhs.uk
jhumphries@hope.srht.nwest.nhs.uk

Prof David Dearnaley
Consultant Oncologist
Academic Unit of Radiotherapy & Oncology
Royal Marsden NHS Trust
Downs Road
Sutton
Surrey
SM2 5PT
T: 020 8642 6011
F: 020 8643 8809
E: davidd@icr.ac.uk

Mr John Anderson
Dept of Urology
Royal Hallamshire Hospital
Glossop Rd
Sheffield
S10 2JF
T: 0114 271 3482
F: 0114 271 3425
E: johnanderson@clara.co.uk

Professor Nicholas James (Chair)
Cancer Research UK Institute for Cancer Studies
University of Birmingham
Edgbaston, Birmingham
B15 2TT
T: 0121 414 4097/3787
F: 0121 414 3263
E: Jamesnd@trials.bham.ac.uk
HayleyLS@cancer.bham.ac.uk

Mr Rick Popert
Department of Urology
1st Floor
Thomas Guy house
Guys Hospital
St Thomas Street, SE1 9RT
T: 0207 188 7188 ext 86794
F:
E: rick.popert@gstt.sthames.nhs.uk

Mr Andrew Stanley
Pharmacist

Professor Malcolm Mason
Section of Clinical Oncology
Velindre Hospital
Velindre Road
Whitchurch
Cardiff
CF14 2TL
T: 029 2031 6964
F: 029 2052 9625
E: masonmd@cf.ac.uk

Research Fellow
To be confirmed
CRUK Institute for Cancer Studies
University of Birmingham
Edgbaston, Birmingham
B15 2TT
Tel: ---
Email: ---

Patient Representitives
John Dwyer, Stockport
Jim Stansfeld, Hampshire

Molecular Genetics Advisor
Professor John Masters
University College London
67 Riding House Street
London
W1W 7EY
T: 020 7679 9543
F: 020 7679 9366
E: j.masters@ucl.ac.uk

Health Economics Advisor
Professor Mark Sculpher
The University of York
Centre for Heath Economics
Heslington
York
YO10 5DD
Tel: 01904 321440
Email: mjs23@york.ac.uk

Quality of Life Advisor
Mr. Richard Stephens
MRC Clinical Trials Unit
Cancer Division

Other members
MRC Clinical Trials Unit staff

Representatives from the three drug companies involved

222 Euston Road
London
NW1 2DA
Tel: 020 7670 4700
Fax: 020 7670 4818
Email: stampede@ctu.mrc.ac.uk

(the drug company representatives only attend open sessions of Trial Management Group meetings)

MRC CTU STAFF

Senior Trial Statistician
Professor Max Parmar
Head, Cancer Division
MRC Clinical Trials Unit
222 Euston Road
London
NW1 2DA
Tel: 020 7670 4731
Fax: 020 7670 4818
Email: mp@ctu.mrc.ac.uk

Senior Trial Statistician
Matthew Sydes
Cancer Division
MRC Clinical Trials Unit
222 Euston Road
London
NW1 2DA
Tel: 020 7670 4798
Fax: 020 7670 4818
Email: ms@ctu.mrc.ac.uk

Trial Manager
Jacqui Nuttall
Cancer Division
MRC Clinical Trials Unit
222 Euston Road
London
NW1 2DA
Tel: 020 7670 4831
Fax: 020 7670 4818
Email: stampede@ctu.mrc.ac.uk

Data Manager
Shama Hassan
Cancer Division
MRC Clinical Trials Unit
222 Euston Road
London
NW1 2DA
Tel: 020 7670 4844
Fax: 020 7670 4818
Email: stampede@ctu.mrc.ac.uk

Trial Steering Committee

Professor David Kirk
Consultant Urologist
Department of Urology
Gartnavel General Hospital
1035 Great Western Rd
Glasgow
G12 0YN

Jim Paul
Senior Statistician
CRC Trials Unit
Beatson Oncology Centre
E Block, Western Infirmary
Glasgow
G11 6NT

Dr Jonathan Ledermann MD FRCP
Cancer Research UK and UCL Cancer Trials Centre
Stephenson House
158-160 North Gower Street
London
NW1 2ND

Independent Data Monitoring Committee

Professor Reginald Hall
Director / Urologist
Northern Cancer Network
Freeman Hospital
Newcastle-upon-Tyne
NE7 7DN

Christopher Williams
Cochrane Cancer Network
Institute of Health Sciences
3rd Floor, Old Road
Headington, Oxford
OX3 7LF

Doug Altman
Director of CSM and
Cancer Research UK Medical Statistics Group
Centre for Statistics in Medicine
Old Road Campus
Old Road
Oxford
OX3 7LF

Appendix M - Participating Site Accreditation Form

Investigator statement

Institution: ____________________________________

I, the undersigned declare that:

1. The above named institution for which I am the named Principal Investigator regularly undertakes the treatment for Locally advanced and metastatic prostate cancer. Supporting evidence of this will be made available to the Trial Management Group if requested.
2. The institution for which I am the named Principal Investigator will participate in the STAMPEDE trial and expects to recruit approximately ______ patients per year.
3. I have read and am familiar with the current protocol.
4. I am thoroughly familiar with the appropriate use of the investigational products, as described in the protocol (and in current investigators brochure).
5. The institution has an adequate number of qualified staff and adequate facilities for the foreseen duration of the trial to conduct the trial properly and safely.
6. I will ensure that all colleagues and supporting staff assisting with the trial are adequately informed about the protocol, the investigational products and their trial related duties.
7. I agree to conduct the study in accordance with the current protocol and will only depart from the protocol when necessary to protect the safety, rights or welfare of patients.
8. To ensure Zoledronic Acid and Doxetaxel is administered in a dedicated oncology facility with full-time consultant oncologist supervision.
9. To ensure a formal protocol is in place to deal with neutropenic sepsis and thrombocytopaenia.
10. To ensure Pharmacists have read the protocol and are aware of the necessary procedures stated within.

I agree to comply with the obligations below:

a) The trial will be conducted in compliance with GCP and applicable regulatory requirements.
b) The institution will permit monitoring and auditing by the MRC CTU or individuals or organisations appointed or agreed by them (e.g. MRC Head Office, NCRN) and inspection by the appropriate regulatory authorities. Direct access will be made available to all relevant data, documents, clinical case records and reports at the trial sites and any related sites.
c) The institution will maintain a Trial Master file, which will contain essential documents for the conduct of the trial.
d) To submit all trial data in a timely manner and as described in the protocol. Individual institutions may be suspended if data returns are poor or if trial conduct is violated in other ways.
e) To report all Serious Adverse Events (SAEs) immediately to the MRC Clinical trials unit, except for those that the protocol or summary of products characteristics identifies as not requiring immediate reporting (these should be reported on the CRFs for the trial). The initial SAE report shall be promptly followed by detailed written reports.
f) That no trial data will be disclosed, presented or published without the approval of the Trial Steering Committee.
g) To retain all trial related documents for 5 years after the completion of the trial.

□ **I have no potential conflict of interest,** e.g. a professional interest, a proprietary interest or any other conflict of interest.
□ **YES, I have a potential conflict of interest** (If you have a potential conflict of interest, we will send you an appropriate form).

Name of Principal Investigator: ____
(Print name in Capitals)

Signature: ________________________________ **Date:** ________________________________

STAMPEDE Signature list and delegation of responsibilities

Institution: ____

This form must be completed by all personnel managing patients and those responsible for completing CRFs (e.g oncologists, surgeons, pathologists and research nurses/data managers). Only staff who are included on this form will be authorised to sign CRFs.

Name	Job title	Sample signature	Sample short signature (initials)	Responsibilities (please tick all applicable boxes)											
				A	B	C	D	E	F	G	H	I	J	K	L

Please notify the MRC CTU of any changes to trial personnel by updating this form.

Responsibilities key:

A Medical care of patients
B Adverse event reporting
C Ethics/regulatory approval
D Registration/Randomisation
E Informed consent
F CRF completion.
G Trial master file maintenance
H Drug accountability
I Pathology specimen processing
J Quality of Life Administration
K Pharmacy
L Laboratory

Formatted

Full contact details for all trial personnel

**Hospital:**		**Principle Investigator:**	
**Address:**		**Main contact person** *(e.g. data queries, general correspondence):*	

- Complete this form for all trial personnel
- Notify the MRC CTU of any contact or trial personnel changes
- Use additional sheets if necessary

	Research nurse/ Data manager	Research nurse/ Data manager
**Name (title, first name/initial, surname)**		
**Department**		
**Phone**		
**Fax**		
**E-mail**		
**Address** (if different from above)		

	Oncologist / Urologist	Oncologist / Urologist
**Name (title, first name/initial, surname)**		
**Department**		
**Phone**		
**Fax**		
**E-mail**		
**Address** (if different from above		

	Lead Pharmacist	Back-up Pharmacist
**Name (title, first name/initial, surname)**		
**Department**		
**Phone**		
**Fax**		
**E-mail**		
**Address** (if different from above)		

Appendix N - Quality of life and health economics

1. Overview

The economic evaluation will take the form of a cost-effectiveness analysis in which the differential cost of the alternative treatments will be related to their differential benefits in terms of quality-adjusted life years (QALYs). Incremental analysis will be undertaken and cost-effectiveness acceptability curves will be used to show the probability of one option being more cost-effective than the others.

2. Estimating costs

A cost analysis will be undertaken from the perspective of the National Health Service. Resource use measurement during the trial will be divided into that relating to the hospital and NHS non-hospital. These are dealt with in turn below.

2.1 Hospital resource use

Within the trial, hospital resource use data will be collected on all patients entering the trial. Specifically, this will include in-patient nights in hospital, distinguishing intensive care from stay on a general ward. These will be collected using case record forms completed at clinical review at the follow-up points shown in Figure 1 of the main document. Some visits to, and stays in, hospital may relate to non-study hospitals. To ensure that data on this form of resource use are captured, a questionnaire will be administered to patients as part of the quality of life assessments.

These resources will be valued in monetary terms using unit costs representative of UK practice at the time of analysis. For drugs, this will be based on British National Formulary prices. For hospital procedure and hotel costs, unit costs will, if available, be based on NHS Reference Costs. Otherwise, they will be estimated from a sample of UK centres randomising patients into the trial.

2.2 NHS non-hospital resource use

Data on patients' use of community-based NHS (and complementary health) services will be collected from patients as part of the quality of life assessments. The resources will include visits to and from a GP or district nurse. Costing of community-based resources will be based on published unit costs. Other services will be costed using data available at the point of analysis.

3. Measuring effects

The clinical trial is estimating a range of clinical and health-related quality of life effects in trial patients. The purpose of the economic evaluation will be to set these in context of the resource costs incurred in achieving them. A cost-effectiveness analysis will relate

differential cost to an aggregated measure of effect in the form of a QALY. QALYs will be based on observed mortality and patients' responses to the EQ-5D questionnaire. The latter asks patients to categorise their health, with 3 levels of response (no problems, moderate problems, severe problems) on 5 dimensions (mobility, self care, usual activities, pain/discomfort, depression anxiety) (44). Each of the 245 possible health states has been 'valued' on a zero to one 'utility' based on the preferences of 3,395 members of the UK public (ref. 40).

4. Analysis

All resource use data will be valued in monetary terms as described above such that each patient has a cost over the period of follow-up. A full stochastic analysis will be undertaken to allow for sample variation in resource use and effect data. Methods are developing quickly in this area and, by the time of the analysis, 'best practice' may have altered markedly from today. If such an analysis were to be undertaken now, the general methods would be as follows.

A QALY profile will be estimated for each patient based on their survival duration weighted by their responses to the EQ-5D Health Related QL questionnaire, which generates a single index value for health at each point of follow-up6. The profiles will assume a straight-line relationship between the index value at time t and the value at time t+1. The number of QALYs they experience during the period of follow-up in the trial will be the area under the QALY profile.

In the primary analysis, only data collected in the trial will be used in the analysis; in other words, the estimate of QALYs for each group is likely to reflect the fact that some patients are still alive after the follow-up (i.e. the survival curve is truncated and survival analysis techniques will be used to estimate QALYs).

As a secondary analysis, extrapolation techniques will be used to estimate the final portion of the survival curve so as to provide a full estimate of differential life expectancy. A number of extrapolation techniques will be used to provide a range of estimates of differential QALYs over a lifetime time horizon.

Cost-effectiveness acceptability curves will be used to facilitate a measure of uncertainty around cost-effectiveness estimates. These curves show the probability of one form of management being more cost-effective than the others assuming alternative levels of the maximum amount decision-makers are willing to pay for an extra QALY.

Sensitivity analysis will be used to consider the importance of sources of uncertainty other than sample variation (e.g. unit costs, discount rates, method of extrapolation). Multiple

regression techniques will be employed to provide as precise a measure of cost-effectiveness as possible and to undertake sub-group analysis using baseline patient characteristics which will be defined in advance in the analysis plan.

Appendix O - Case report forms

The case report forms will be inserted here.

Appendix P - Assessing and notifying CTU of adverse events

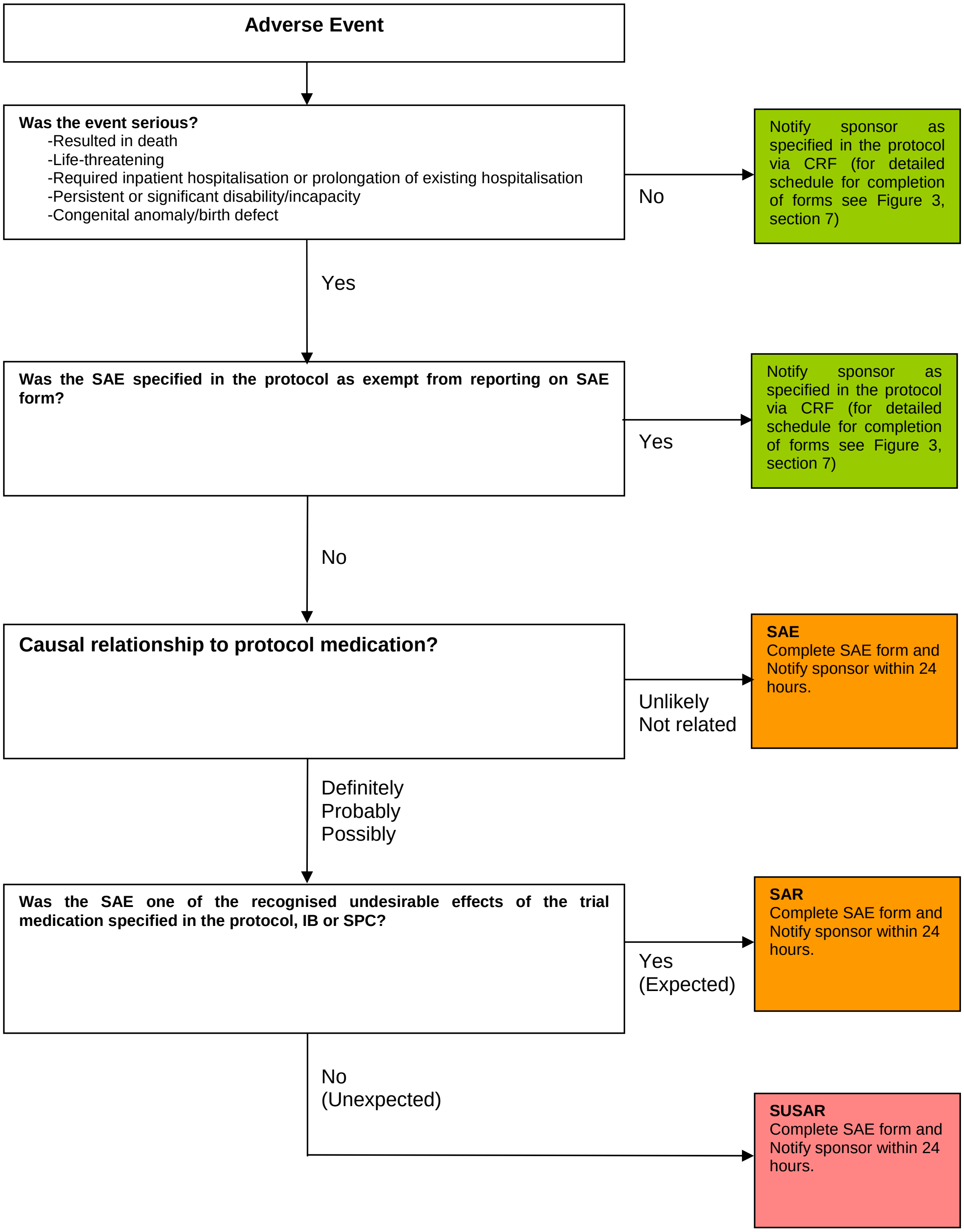

CRF: Case report form
IB: Investigator's brochure
SAE: Serious adverse event
SAR: Serious adverse reaction
SPC: Summary of product characteristics
SUSAR: Suspected unexpected serious adverse reaction
