# Supplementary material for: Abiraterone acetate plus prednisolone for metastatic patients starting hormone therapy: 5‐year follow‐up results from the STAMPEDE randomised trial (NCT00268476)
Source: Int J Cancer. 2022 May 16;151(3):422–34. doi: 10.1002/ijc.34018 (PMC9321995; doi:10.1002/ijc.34018)
Supplement: Supplementary file 2 — Appendix S2Supporting Information. [file IJC-151-422-s001.zip › IJC_34018_STAMPEDE_Protocol_v12.0.pdf]

# STAMPEDE

# Systemic Therapy in Advancing or Metastatic Prostate Cancer: Evaluation of Drug Efficacy

## A multi-arm multi-stage randomised controlled trial

**Version:** 12.0
**Date:** 13-Jan-2014

| | |
|---|---|
| MRC CTU ID: | PR08 |
| ISRCTN #: | ISRCTN78818544 |
| NCT #: | NCT00268476 |
| EUDRACT #: | 2004-000193-31 |
| CTA #: | 00316/0026/001-0001 |
| MREC #: | 04/MRE07/35 |


**Authorised by:**
Name: PROFESSOR NICHOLAS D JAMES
Role: CHIEF INVESTIGATOR

Name: MATTHEW SYDES
Role: TRIAL STATISTICIAN

# GENERAL INFORMATION

This document was constructed using the MRC CTU Protocol Template Version 4.0. It describes the STAMPEDE trial, coordinated by the Medical Research Council (MRC) Clinical Trials Unit (CTU) at University College London (UCL), and provides information about procedures for entering patients into it. The protocol should not be used as an aide-memoire or guide for the treatment of other patients. Every care has been taken in drafting this protocol, but corrections or amendments may be necessary. These will be circulated to the registered investigators in the trial, but sites entering patients for the first time are advised to contact the Cancer And Other Non-Infectious Diseases Group, MRC CTU at UCL, London, UK, to confirm they have the most up-to-date version.

## COMPLIANCE

The trial will be conducted in compliance with the approved protocol, the Declaration of Helsinki 1996, the principles of Good Clinical Practice (GCP), Commission Directive 2005/28/EC with the implementation in national legislation in the UK by Statutory Instrument 2004/1031 and subsequent amendments, the UK Data Protection Act (DPA number: Z5886415), and the National Health Service (NHS) Research Governance Framework for Health and Social Care (RGF). International sites will comply with the principles of GCP as laid down by the ICH topic E6 (Note for Guidance on GCP), Commission Directive 2005/28/EC (the European Directive 2001/20/EC [where applicable]) and applicable national regulations.

## SPONSOR

Medical Research Council, 2nd Floor, David Phillips Building, Polaris House, North Star Avenue, Swindon, SN2 1FL, UK

## FUNDING

Clinical Trials Advisory Awards Committee (on behalf of Cancer Research UK, Medical Research Council, and other charities) together with educational grants from Novartis, Sanofi-Aventis, Pfizer ,Janssen Pharma NV, Astellas.

## AUTHORISATIONS AND APPROVALS

The following persons are authorised to sign the final protocol and protocol amendments for the sponsor: Professor Nicholas James (Chief Investigator) and Matthew Sydes (Trial Statistician).

## TRIAL REGISTRATION

This trial has been registered with the ClinicalTrials.gov Clinical Trials Register, where it is identified as NCT00268476.

**RANDOMISATIONS**
To randomise, call MRC CTU at UCL, Monday to Friday 0900-1700
excluding public holidays or dates when notice has been given by the CTU.
Tel: +44 (0) 20 7670 4777

**SAE REPORTING**
Fax to 020 7670 4818 within 24 hours of becoming aware of the event

# TRIAL ADMINISTRATION

## COORDINATING SITE

MRC Clinical Trials Unit at UCL
Aviation House
Kingsway
London
WC2B 6NH
UK

Switchboard: 020 7670 4700
Fax: 020 7670 4818
Email: mrcctu.stampede@ucl.ac.uk

## MRC CTU STAFF

| | | | |
|---|---|---|---|
| Trial Manager: | Francesca Schiavone | Tel: | 0207 670 4632 |
| Trial Manager: | Alanna Brown | Tel: | 0207 670 4882 |
| Data Manager: | Dominic Hague | Tel: | 0207 670 4947 |
| Data Manager: | Katie Ward | Tel: | 0207 670 4794 |
| Data Manager: | Peter Vaughan | Tel: | 0207 670 4655 |
| Clinical Project Manager | Claire Amos | Tel: | 0207 670 4771 |
| Statistician: | Melissa Spears | Tel: | 0207 670 4764 |
| Senior Trial Statistician: | Matt Sydes | Tel: | 0207 670 4798 |
| Director of MRC CTU: | Max Parmar | Tel: | 0207 670 4729 |
| Trial Surgeon: | Alastair Ritchie | Tel: | Please contact STAMPEDE team |

## CHIEF INVESTIGATOR

Professor Nicholas James
Warwick Medical School
University of Warwick
Coventry
CV4 7AL
UK

Tel: 024 7657 4365 (Secretary)
Fax:
Email: n.d.james@warwick.ac.uk

## CO-INVESTIGATORS

| | | |
|---|---|---|
| Prof Noel Clarke<br>Urologist | Manchester, UK | Vice-Chair |
| Prof Johann De Bono<br>Oncologist | London, UK | |
| Prof Malcolm Mason<br>Oncologist | Cardiff, UK | Vice-Chair |
| Dr Chris Parker<br>Oncologist | London, UK | Lead: M1/RT comparison |
| Dr Gerhardt Attard<br>Oncologist | London, UK | Lead: Enzalutamide + Abiraterone comparison |
| Dr Daniel Aebersold<br>Oncologist | Bern, Switzerland | |
| Dr Martin Russell | Glasgow, UK | |
| Prof George Thalmann<br>Urologist | Bern, Switzerland | |

**Patient Representative**
Robin Millman
Patient

David Matheson
Patient

**Health Economics**
Prof Mark Sculpher — York, UK

**Molecular Genetics**
Prof John Masters, Pathologist — London, UK

For full details of all trial committees, please see **Appendix M**.

# CONTENTS

GENERAL INFORMATION ........ I

CONTENTS ........ 4

ABBREVIATIONS ........ 8

1 SUMMARY ........ 11
1.1 LAY SUMMARY ........ 11
1.2 ABSTRACT AND SUMMARY OF TRIAL DESIGN ........ 12
1.3 TRIAL DOCUMENTATION ........ 18

2 BACKGROUND ........ 19
2.1 INTRODUCTION AND SETTING ........ 19
2.1.1 Long-term Androgen Deprivation Therapy ........ 19
2.1.2 Role of Radiotherapy for Patients With M0 Disease ........ 19
2.2 RATIONALE ........ 19
2.3 DESIGN ........ 20
2.4 RESEARCH TREATMENT AND BISPHOSPHONATES ........ 20
2.5 RESEARCH TREATMENT: CHEMOTHERAPY ........ 21
2.6 RESEARCH TREATMENT: CYCLOOXYGENASE-2 INHIBITORS ........ 21
2.7 RESEARCH TREATMENT: STEROID SYNTHESIS INHIBITORS ........ 22
2.8 RESEARCH TREATMENT: RADIOTHERAPY TO THE PROSTATE FOR PATIENTS WITH NEWLY-DIAGNOSED METASTATIC DISEASE ........ 22
2.9 RESEARCH TREATMENT: COMBINATIONS OF ORIGINAL RESEARCH ARMS ........ 23
2.9.1 Bisphosphonate And Chemotherapy ........ 23
2.9.2 Bisphosphonate And Cyclooxygenase-2 Inhibitors ........ 24
2.10 COMBINATION OF STEROID SYNTHESIS INHIBITORS AND ANDROGEN RECEPTOR SIGNALLING INHIBITOR ........ 25
2.10.1 Supplementing Abiraterone And Prednisolone With Enzalutamide ........ 25
2.10.2 Supplementing Enzalutamide With Abiraterone And Prednisolone ........ 25
2.10.3 Summary Of Rationale For This Combination ........ 25

3 SELECTION OF INSTITUTIONS AND INVESTIGATORS ........ 27
3.1 RADIOTHERAPY ACCREDITATION ........ 27

4 SELECTION OF PATIENTS ........ 28
4.1 PATIENT INCLUSION CRITERIA ........ 28
4.1.1 High-Risk Newly-Diagnosed Non-Metastatic Node-Negative Disease ........ 28
4.1.2 Newly-Diagnosed Metastatic Or Node-Positive Disease ........ 28
4.1.3 Previously Treated With Radical Surgery And/Or Radiotherapy, Now Relapsing ........ 28
4.1.4 For All Patients ........ 28
4.2 PATIENT EXCLUSION CRITERIA ........ 29
4.3 SELECTION CRITERIA FOR COMPARISON OF RESEARCH (M1) RT FOR METASTATIC DISEASE ........ 30
4.4 SCREENING PROCEDURES ........ 30
4.4.1 Investigations Prior To Randomisation ........ 30
4.4.2 Androgen Deprivation Therapy Prior To Randomisation ........ 31
4.4.3 Hypercalcaemia At Randomisation ........ 31

4.4.4 NSAIDs And Cox-2 Inhibitors At Randomisation ..... 31
4.4.5 Starting Trial Treatment ..... 31
4.4.6 Concomitant Medications ..... 32
**4.5 ADDITIONAL DETAILS FOR PATIENTS JOINING SUB-STUDIES ..... 32**

**5 RANDOMISATION AND ENROLMENT ..... 33**
**5.1 CO-ENROLMENT GUIDELINES ..... 33**

**6 TREATMENT OF PATIENTS ..... 34**
**6.1 TRIAL TREATMENT ..... 34**
**6.2 ARM A: ADT ALONE OR ADT + STANDARD-OF-CARE (M0) RT (CONTROL ARM) ..... 34**
6.2.1 Hormone Therapy ..... 34
6.2.2 Standard-of-care (M0) RT ..... 34
**6.3 ARM B: ADT + ZOLEDRONIC ACID ..... 35**
**6.4 ARM C: ADT + DOCETAXEL ..... 35**
**6.5 ARM D: ADT + CELECOXIB ..... 35**
**6.6 ARM E: ADT + DOCETAXEL + ZOLEDRONIC ACID ..... 36**
**6.7 ARM F: ADT + ZOLEDRONIC ACID + CELECOXIB ..... 36**
**6.8 ARM G: ADT + ABIRATERONE ..... 36**
**6.9 ARM H: ADT + PROSTATE RADIOTHERAPY IN M1 PATIENTS ..... 37**
**6.10 ARM J: ADT + ABIRATERONE + PREDNISOLONE + ENZALUTAMIDE ADMINISTRATION ..... 38**
**6.11 ADMINISTRATION AND DOSE MODIFICATIONS ..... 38**
6.11.1 Zoledronic Acid ..... 38
6.11.2 Docetaxel ..... 39
6.11.3 Celecoxib ..... 39
6.11.4 Abiraterone or Enzalutamide + Abiraterone ..... 40
6.11.5 Research (M1) Prostate Radiotherapy ..... 42
**6.12 TRIAL PRODUCTS ..... 44**
**6.13 MEASURES OF COMPLIANCE/ADHERENCE ..... 44**
**6.14 TREATMENT DATA COLLECTION ..... 44**
**6.15 ADMINISTRATION OF STANDARD RADIOTHERAPY TO NON-METASTATIC PATIENTS ..... 44**
6.15.1 Treatment Details ..... 44
**6.16 NON-TRIAL TREATMENT ..... 46**
6.16.1 Medications Permitted ..... 46
6.16.2 Data On Concomitant Medication ..... 46

**7 ASSESSMENTS AND PROCEDURES ..... 47**
**7.1 SCHEDULE FOR ASSESSMENTS ..... 47**
7.1.1 PSA Measurements ..... 47
7.1.2 Assessment Of Treatment Failure (Definition Of Progression) ..... 47
7.1.3 Additional Safety Assessment ..... 47
7.1.4 Data Collection and Non-Administration Of Standard Radiotherapy ..... 48
7.1.5 Data Collection Palliative Radiotherapy ..... 48
7.1.6 Data Collection Research (M1) Radiotherapy ..... 48
7.1.7 Follow-Up Schedules ..... 48
**7.2 FOLLOW-UP ..... 48**
**7.3 TRIAL CLOSURE ..... 52**

**8 STOPPING OF TREATMENT OR FOLLOW UP ..... 53**
**8.1 STOPPING RESEARCH INTERVENTIONS ..... 53**

**8.2 PATIENT TRANSFERS ..... 53**
**8.3 WITHDRAWAL FROM THE TRIAL COMPLETELY ..... 53**

**9 STATISTICAL CONSIDERATIONS ..... 55**
**9.1 METHOD OF RANDOMISATION ..... 55**
9.1.1 To Version 7 ..... 55
9.1.2 Version 8 ..... 55
9.1.3 Version 10 and 11 ..... 55
9.1.4 Version 12 ..... 55
**9.2 OUTCOME MEASURES ..... 56**
**9.3 SAMPLE SIZE: PRINCIPLES AND ASSUMPTIONS ..... 57**
**9.4 SAMPLE SIZE ISSUES AND TRIAL STAGES: ORIGINAL RESEARCH ARMS (B-F) ..... 58**
9.4.1 Pilot Phase: Original Research Arms (B-F) ..... 58
9.4.2 Activity Stages I-III: Original Research Arms (B-F) ..... 58
9.4.3 Efficacy Stage IV: Original Research Arms (B-F) ..... 59
9.4.4 Sample Size For Original Research Arms (B-F) ..... 59
**9.5 SAMPLE SIZE ISSUES AND TRIAL STAGES: ADDITIONAL RESEARCH ARM G ..... 59**
9.5.1 Pilot Phase: Additional Research Arm G ..... 59
9.5.2 Activity Stages I-III: Additional Research Arm G ..... 59
9.5.3 Efficacy Stage IV: Additional Research Arm G ..... 60
9.5.4 Sample Size For Additional Research Arm G ..... 60
**9.6 SAMPLE SIZE ISSUES AND TRIAL STAGES: ADDITIONAL RESEARCH ARM H ..... 61**
9.6.1 Pilot Phase: Additional Research Arm H ..... 61
9.6.2 Activity Stages I-III: Additional Research Arm H ..... 61
9.6.3 Efficacy Stage IV: Additional Research Arm H ..... 61
9.6.4 Sample Size For Additional Research Arm H ..... 61
**9.7 SAMPLE SIZE ISSUES AND TRIAL STAGES: ADDITIONAL RESEARCH ARM J ..... 62**
9.7.1 Pilot Phase: Additional Research Arm J ..... 62
9.7.2 Activity Stages I-II: Additional Research Arm J ..... 62
9.7.3 Efficacy Stage III: Additional Research Arm J ..... 62
9.7.4 Sample Size For Additional Research Arm J ..... 63
9.7.5 Further Sample Size Issues For Additional Research Arm J ..... 63
**9.8 FURTHER NOTES ON TRIAL DESIGN ..... 64**
9.8.1 Overall Sample Size ..... 64
9.8.2 Factorial Design ..... 64
**9.9 INTERIM MONITORING AND ANALYSES ..... 65**
**9.10 OUTLINE ANALYSIS PLAN ..... 65**
9.10.1 Pilot / Safety Phases ..... 65
9.10.2 Activity And Efficacy Stages ..... 66

**10 MONITORING AND QUALITY ASSURANCE ..... 67**
**10.1 MONITORING AT MRC CTU ..... 67**
**10.2 DIRECT ACCESS TO DATA ..... 67**
**10.3 VISITS TO INVESTIGATOR SITES ..... 67**
**10.4 CONFIDENTIALITY ..... 67**

**11 SAFETY REPORTING ..... 68**
**11.1 DEFINITIONS ..... 68**
11.1.1 Trial-Specific Exemptions ..... 69
**11.2 INSTITUTION/INVESTIGATOR RESPONSIBILITIES ..... 69**

11.2.1 Investigator Assessment ........ 69
11.2.2 Notification Procedure ........ 70
**11.3 MRC CTU RESPONSIBILITIES ........ 71**

**12 ETHICAL CONSIDERATIONS AND APPROVAL ........ 72**
**12.1 ETHICAL CONSIDERATIONS ........ 72**
**12.2 ETHICAL APPROVAL ........ 72**

**13 REGULATORY APPROVAL ........ 74**

**14 INDEMNITY ........ 75**

**15 FINANCE ........ 76**

**16 TRIAL COMMITTEES ........ 77**
**16.1 TRIAL MANAGEMENT GROUP (TMG) ........ 77**
**16.2 TRIAL STEERING COMMITTEE (TSC) ........ 77**
**16.3 INDEPENDENT DATA MONITORING COMMITTEE (IDMC) ........ 77**

**17 ANCILLARY STUDIES ........ 79**
**17.1 QUALITY OF LIFE ........ 79**
**17.2 HEALTH ECONOMICS ........ 79**
**17.3 TRANSLATIONAL SUB-STUDIES ........ 79**
17.3.1 DNA Analysis ........ 79
17.3.2 Tissue Microarray ........ 80

**18 PUBLICATION ........ 81**

**19 PROTOCOL AMENDMENTS ........ 82**
**19.1 PROTOCOL ........ 82**
19.1.1 Amendments Made To Sections In Protocol Version 1.0 (May 2004) ........ 82
19.1.2 Amendments Made To Sections In Protocol Version 1.1 (May 2005) ........ 82
19.1.3 Amendments Made To Sections In Protocol Version 2.0 (Jun 2005) ........ 82
19.1.4 Amendments Made To Section In Protocol Version 3.0 (Jul 2006) ........ 83
19.1.5 Amendments Made To Protocol Version 4.0 (Dec 2007) ........ 84
19.1.6 Amendments Made To Protocol Version 5.0 (Aug 2008) ........ 84
19.1.7 Amendments Made To Protocol Version 6.0 (Jul 2009) ........ 85
19.1.8 Amendments Made To Protocol Version 7.0 (Jul 2011) ........ 86
19.1.9 Amendments Made To Protocol Version 7.1 (Jul 2011) ........ 86
19.1.10 Amendments Made To Protocol Version 8.0 (Sep 2011) ........ 87
19.1.11 Amendments Made To Protocol Version 9.0 (Oct-2012) ........ 88
19.1.12 Amendments Made To Protocol Version 10.0 (Apr-2013) ........ 89
19.1.13 Amendments Made To Protocol Version 11.0 (Sep-2013) ........ 89

**20 REFERENCES ........ 90**

# ABBREVIATIONS

| Abbreviation | Expansion |
|---|---|
| ACE | Angiotensin-Converting Enzyme |
| ACTH | Adrenocorticotropic hormone |
| ADT | Androgen deprivation therapy |
| AR | Androgen receptor |
| AS | Activity Stage |
| bid | Twice a day (bis in die) |
| BP | Blood pressure |
| BSA | Body surface area |
| CERES | Consumers for Ethics in Research |
| CF | Consent Form |
| CI | Chief Investigator |
| CI | Confidence interval |
| COSTART | Coding Symbols for a Thesaurus of Adverse Reaction Terms |
| Cox 2 | Cyclooxygenase 2 |
| CRF | Case Report Form |
| CRUK | Cancer Research UK |
| CRPC | Castrate Refractory Prostate Cancer |
| CT | Computerised tomography |
| CTA | Clinical Trials Authorisation |
| CTAAC | Clinical Trials Advisory and Awards Committee |
| CTC | Common Toxicity Criteria |
| CTU | Clinical Trials Unit |
| CTV | Clinical Tumour Volume |
| CXR | Chest X-ray |
| DDX | Doctors and Dentists Exemption |
| DHT | Dihydrotestosterone |
| DNA | Deoxyribonucleic Acid |
| DPA | Data Protection Act |
| ERC | Endpoint Review Committee |
| ES | Efficacy Stage |
| ICH | International Conference on Harmonization |

| Abbreviation | Expansion |
|---|---|
| ECG | Electro cardiogram |
| FBC | Full Blood Count |
| FFS | Failure-Free Survival |
| GCP | Good Clinical Practice |
| GP | General Practitioner |
| GRO | General Register Office |
| HE | Health Economics |
| HES | Hospital Episode Statistics |
| hr | Hour |
| HR | Hazard Ratio |
| HRPC | Hormone Refractory Prostate Cancer |
| HT | Hormone Therapy |
| IDMC | Independent Data Monitoring Committee |
| IM | Intramuscular |
| IMRT | Intensity Modulated Radiation Therapy |
| ISRCTN | International Standard Randomised Controlled Trial Number |
| IU | International Units |
| IV | Intravenous |
| LD | Longest diameter |
| LFTs | Liver Function Tests |
| LHRH | Luteinising Hormone Releasing Hormone |
| LREC | Local Research Ethics Committee |
| m | Month |
| MHRA | Medicine and Healthcare Products Regulatory Agency |
| min | Minutes |
| MRC | Medical Research Council |
| MREC | Multi-Centre Research Ethics Committee |
| MRI | Magnetic resonance imaging |
| M0 | Non-metastatic |
| M1 | Metastatic |
| NCI | National Cancer Institute (USA) |
| NCRN | National Cancer Research Network |
| NHS | National Health Service |

| Abbreviation | Expansion |
|---|---|
| NSAID | Non-Steroidal Anti-inflammatory Drugs |
| ONS | Office for National Statistics |
| OS | Overall Survival |
| PI | Principal Investigator |
| PIS | Patient Information Sheet |
| po | per orum (orally) |
| PSA | Prostate Specific Antigen |
| pts | Patients |
| PTV | Planned Tumour Volume |
| QALY | Quality-adjusted Life Years |
| qds | quater die sumendus (4 times each day) |
| QL | Quality of Life |
| R&D | Research and Development |
| RECIST | Response Evaluation Criteria In Solid Tumours |
| SAE | Serious Adverse Event |
| SAR | Serious Adverse Reaction |
| sc | Sub-cutaneous (under skin) |
| SNP | Single Nucleotide Polymorphism |
| SSA | Site Specific Assessment |
| STAMPEDE | Systemic Therapy in Advancing and Metastatic Prostate Cancer: Evaluation of Drug Efficacy |
| SUSAR | Suspected Unexpected Serious Adverse Reactions |
| SWOG | South West Oncology Group |
| TMG | Trial Management Group |
| TMT | Trial Management Team |
| TURP | Trans-Urethral Resection of Prostate |
| TSC | Trial Steering Committee |
| UCL | University College London |
| ULN | Upper Limit of Normal |
| U+E | Urea and Electrolytes |
| WHO | World Health Organisation |

# 1 SUMMARY

## 1.1 LAY SUMMARY

Prostate cancers depend upon the male hormone testosterone for their growth. Lowering testosterone levels (either by removing all or part of both testes, or by giving anti-hormone treatment) slows the growth of prostate cancers. This type of treatment is called hormone treatment and is often used when prostate cancers have spread outside the prostate gland. Although hormone treatment is usually successful at stopping the cancer growing for a period of time, the cancer will begin to grow again in most men.

There are increasing numbers of treatments available for advanced prostate cancer. These treatments are usually used in prostate cancer when hormone treatment is no longer effective and the cancer has started to grow again. The aim of this trial, which is called STAMPEDE, is to assess some of these treatments, given earlier in the course of the disease in combination with hormone treatment.

The treatments that have been, or are being, assessed during the trial are:

**1. Zoledronic acid:** Prostate cancer cells can spread to bones and weaken them. Zoledronic acid is a drug that reduces bone destruction and hardens bones. This may make them more resistant to attack by cancer cells.

**2. Docetaxel:** A drug that stops cells replicating that is currently being used to treat a range of cancers including lung, breast and ovarian cancer as well as prostate cancer. Docetaxel prolongs survival in men with relapsed metastatic prostate cancer.

**3. Celecoxib:** An aspirin-like drug that is used to treat arthritis. It slows down the growth of cancer cells in the laboratory. We wished to see if it had the same effect on cancer cells in patients. Recruitment to new patients for the evaluation of this drug is finished as a planned interim analysis failed to demonstrate sufficient activity.

**4. Abiraterone** (included from protocol version 8.0)**:** An inhibitor of steroid hormone synthesis that blocks prostate cancer cells from generating their own male hormones. This is thought to be a major way in which prostate cancer cells resume growth following anti-hormonal therapies. The agent prolongs survival when given to men following failure of docetaxel chemotherapy.

**5. Prostate radiotherapy** (included from protocol version 9.0)**:** treatment with high-energy x-rays targeted to the prostate gland. This treatment is now mandatory for patients with cancer that is confined to the prostate gland as large trials have shown it improves survival times. We are not certain whether we should give radiotherapy to the prostate if the cancer has already spread.

**6. Enzalutamide** (included from protocol version 12.0)**:** This is a blocker of androgen receptors. These stimulate the cancer when hormone therapies have failed. Enzalutamide may be mutually complementary to abiraterone in terms of blocking mechanisms of resistance. The agent prolongs survival when given to men following failure of docetaxel chemotherapy.

STAMPEDE will look at the effect of combining one or two of the treatments described above with hormone treatment. A computer program will be used to allocate which treatment the patient receives, using a chance process. The trial will look at the effects of the combined treatments on quality of life and find out whether the new treatment combinations increase the time when the cancer is not growing and ultimately results in patients living longer. The study will also look at which treatment provides the greater value for money for the health service. More than 7,000 patients will join the trial with answers becoming available over 7 to 12 years.

## 1.2 ABSTRACT AND SUMMARY OF TRIAL DESIGN

STAMPEDE is a multi-centre, randomised controlled trial for patients with locally advanced or metastatic prostate cancer who are about to commence Androgen Deprivation Therapy (ADT). Patients can have either newly diagnosed disease, or have been previously treated with radical radiotherapy or surgery but now have signed of progression such as a rising prostate specific antigen (PSA) (further details on eligibility see **Section 4**). The trial will assess the effects of adding different agents, both as single agents and in combinations, to androgen deprivation therapy. The investigational agents are (i) a bisphosphonate, zoledronic acid, (ii) a cytotoxic chemotherapeutic agent, docetaxel and (iii) a cyclooxygenase (Cox-2) inhibitor, celecoxib (iv) a novel androgen deprivation therapy drug called abiraterone, a steroid synthesis inhibitor and a androgen receptor signalling inhibitor (v) enzalutamide. Recruitment to the celecoxib arms (D and F) is now closed. An additional arm containing abiraterone was added in protocol version 8.0. A further comparison arm involving prostate radiotherapy for patients with metastatic disease was added in protocol version 9.0. The trial has multiple arms; the control arm of the trial is androgen deprivation therapy (ADT) only, achieved through the use of luteinising hormone releasing hormone (LHRH) analogues or LHRH antagonists, or bilateral orchidectomy according to local practice. The other trial arms are summarised in **Figures 1 to 7**.

**Figure 1: Recruiting arms of the STAMPEDE trial to Apr-2011**

Patients eligible for STAMPEDE

↓

**STARTING LONG-TERM HORMONES**

**RANDOMISATION**

| | |
|---|---|
| **A** | ADT |
| **B** | ADT + zoledronic acid |
| **C** | ADT + docetaxel |
| **D** | ADT + celecoxib |
| **E** | ADT + ZA + docetaxel |
| **F** | ADT + ZA + celecoxib |

**Figure 2: Recruiting arms of the STAMPEDE trial from Apr-2011 to Nov-2011 (v7.0)**

Patients eligible for STAMPEDE

↓

**STARTING LONG-TERM HORMONES**

**RANDOMISATION**

| | |
|---|---|
| **A** | ADT |
| **B** | ADT + zoledronic acid |
| **C** | ADT + docetaxel |
| **E** | ADT + ZA + docetaxel |

Accrual stopped to celecoxib-containing Arms, D and F, after their Activity Stage II analysis

**Figure 3: Recruiting arms of the STAMPEDE trial from Nov-2011 to Jan-2013 (v9.0)**

Patients eligible for STAMPEDE

↓

**STARTING LONG-TERM HORMONES[1]**

**RANDOMISATION**

| | |
|---|---|
| **A** | ADT |
| **B** | ADT + zoledronic acid |
| **C** | ADT + docetaxel |
| **E** | ADT + ZA + docetaxel |
| **G** | ADT + abiraterone |

[1] All suitable pts with newly diagnosed locally advanced disease should also have RT to the prostate

Accrual was initiated to the abiraterone arm, Arm G, in Nov-2011.

**Figure 4: Recruiting arms of the STAMPEDE trial from protocol version 9.0 (Jan-2013 to Mar-2013)**

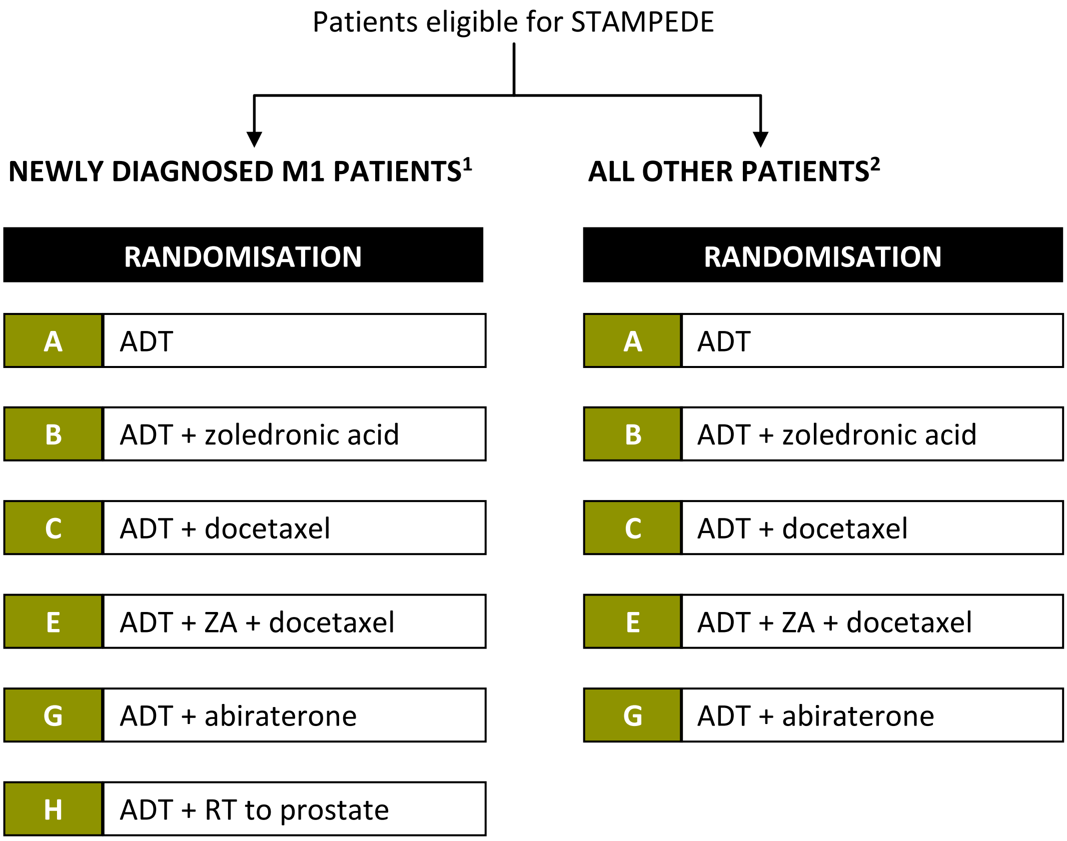


[1] Except pts with a contra-indication to RT
[2] All suitable pts with newly diagnosed locally advanced disease should also have RT to the prostate

Accrual was initiated to the radiotherapy-to-the-prostate for metastatic disease arm, Arm H, in Jan-2013.

**Figure 5: Arms of the STAMPEDE Trial from protocol version 10.0 (after original research arms completed accrual in March 2013)**

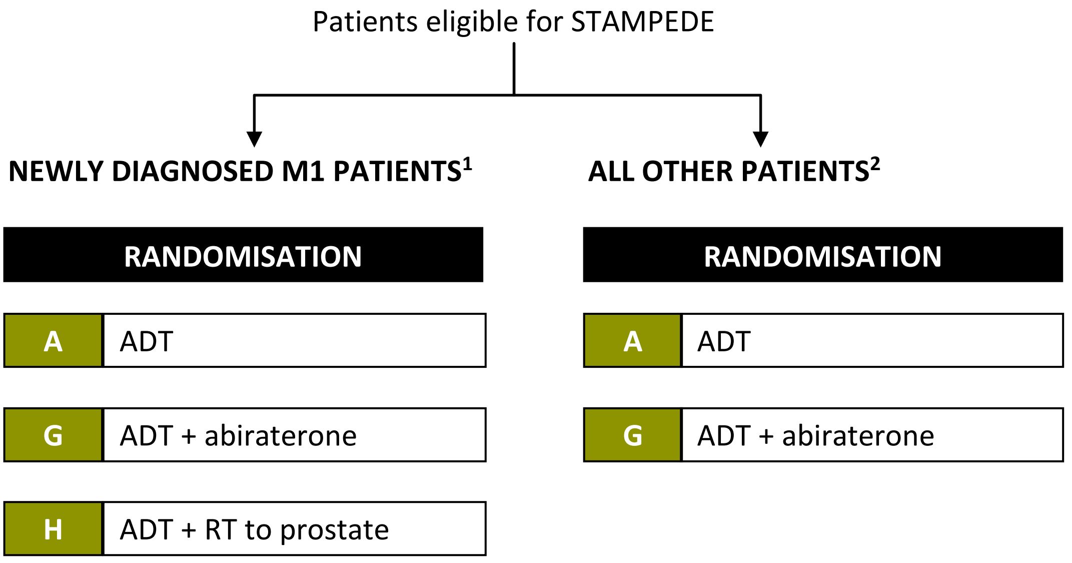


[1] Except pts with a contra-indication to RT
[2] All suitable pts with newly diagnosed locally advanced disease should also have RT to the prostate

**Figure 6:** Arms of the STAMPEDE Trial from protocol version 12.0 (end of recruitment to Arm G and introduction of enzalutamide + abiraterone comparison)

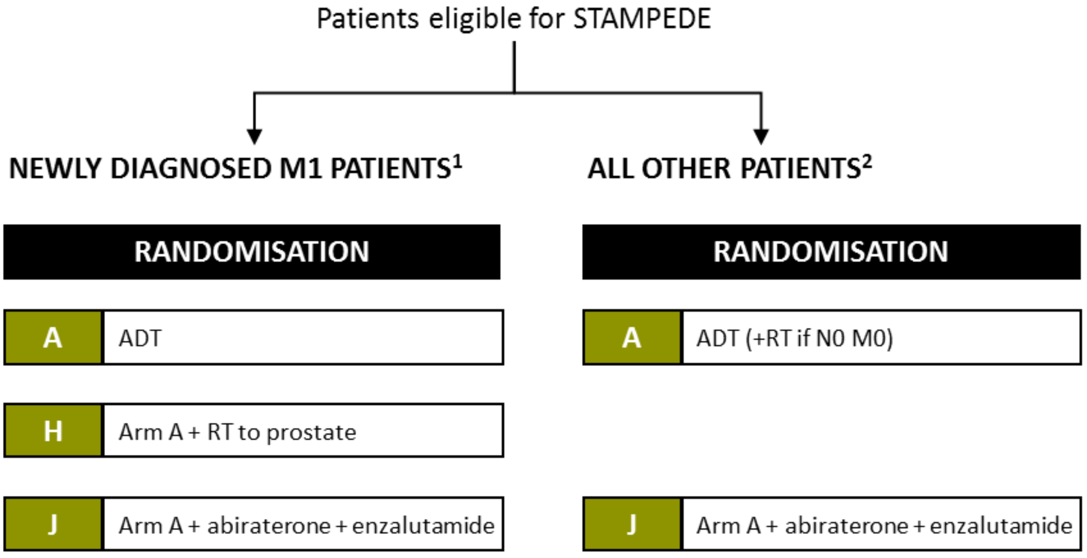


[1] Except pts with a contra-indication to RT
[2] All suitable pts with newly diagnosed locally advanced disease should also have RT to the prostate1

## Figure 7: Arms of the STAMPEDE trial open to recruitment over time

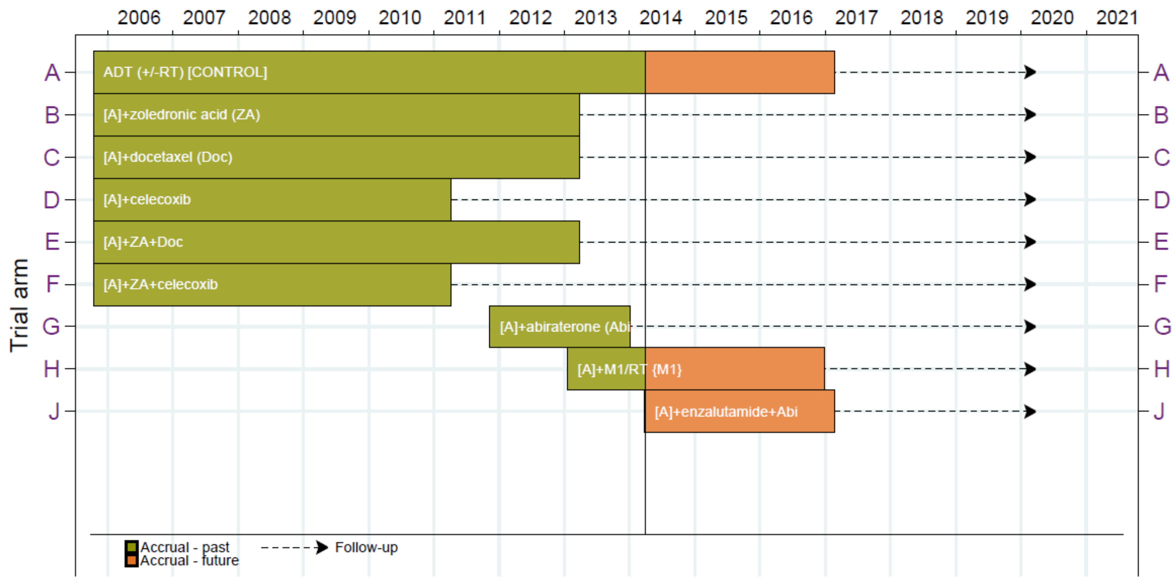


For each comparison of research arm against control, the trial will be conducted in a number of stages: a Pilot/Safety Phase, Activity Stages and a final Efficacy Stage. The primary outcome measure of the Pilot/Safety Phase is the safety, with 30 patients recruited to each research arm. Research arms will only continue to recruitment in the next stage if they have been shown to be both safe and feasible, although patient data from all patients and all stages will be included in the final analyses. In the Activity Stages until the primary outcome measure is failure-free survival (FFS). Further patients will be recruited until a certain number of FFS events have been observed in the control arm (see **Section 9** for further detail). Some evidence of activity will be required for a research arm to proceed to further recruitment in each stage and guidelines are in place. In the Efficacy Stage, patients will be recruited when a certain number of primary outcome measure events are foreseeable in the control arm. This is when around 403 deaths have been reported in the control arm for the “original research comparisons” and around 267 in the control arm for the “abiraterone comparison”, the “M1/RT comparison” and the “enzalutamide+abiraterone comparison”. The exact number of patients and duration of the trial will depend on the observed accrual rate, observed event rate and the number of other arms open to recruitment contemporaneously.

Recruitment to Arms D (ADT + celecoxib) and F (ADT + zoledronic acid + celecoxib) was stopped in Apr-2011 after the second planned activity analysis when the IDMC and TSC considered the lack-of-benefit guidelines.(1) Refer to **Section 9.3** for further information regarding the guidelines for stopping accrual to research arms during the activity stages of the trial.

In version 8.0 of the protocol a new arm G (ADT + abiraterone) was added. Arm H (ADT+ prostate radiotherapy) was added in protocol version 9.0. The trial stages remain as at trial inception but will be staggered in time compared to the stages for the original arms A-F. Protocol version 10.0 was approved following the completion of recruitment to the remaining original trial arms (B, C and E) and was a "housekeeping" change to remove references to the completed arms from the information sheets. Protocol version 11.0 was approved following the extension of the recruitment target sample for the “abiraterone comparison” from 1,500 to 1,800 patients. Protocol version 12.0 adds a new combination therapy arm containing abiraterone with enzalutamide.

Patients will be assessed 6 weekly for the first 24 weeks after randomisation and then every 12 weeks up to 2 years, then 6-monthly until 5 years and annually, thereafter. The first 700 patients on trial completed questionnaires aimed at assessing the effects of the investigational treatments on their quality of life (QL) and on their use of health care resources (Health Economics (HE) study). From protocol version 8.0, the QL and HE study has been re-opened to all new patients.

In addition, there are translational sub-studies. Patients willing to participate will be asked at randomisation to donate a droplet of blood, which will be stored for DNA and protein analysis in order to try to identify markers that are associated with response to therapy, side-effects or susceptibility to prostate cancer.

Patients will also be asked to give permission to use some of their stored material (blood or biopsy samples) for further studies on the causes and nature of prostate cancer. In selected centres patients were asked to participate in a bone mineral density sub-study. This sub-study has now stopped recruitment. There are separate patient information sheets for the QL and HE study and the translational sub-studies (For further details of ancillary studies, see **Section 17**).

## 1.3 TRIAL DOCUMENTATION

**Table 1** presents a summary of the required trial documentation for participating centres

**Table 1: Trial documentation required for participating centres**

| TRIAL DOCUMENTATION | TIMING |
|---|---|
| R&D approval (including IRMER approval) | Before centre participation |
| Investigator Statement | Before centre participation |
| Signature list & delegation of responsibilities | Before centre participation |
| Trial personnel contact details | Before centre participation |
| PIS, GP & CF on local paper | Before centre participation |
| Signed Clinical Trial Agreement between Trust and Sponsor | Before centre participation |
| RTQA accreditation | Before centre participation |
| Clinical Trial Agreement (or Variation if applicable) | Before centre participation |

# 2 BACKGROUND

## 2.1 INTRODUCTION AND SETTING

Prostate cancer is a major health problem world-wide and accounts for nearly one fifth of all newly diagnosed male cancers. In the UK, approximately 35,000 men are diagnosed with prostate cancer each year and in 2008 almost 10,000 men died from the disease.(2)

### 2.1.1 LONG-TERM ANDROGEN DEPRIVATION THERAPY

The initial (first line) treatment for locally advanced or metastatic prostate cancer is androgen deprivation therapy (ADT) achieved either surgically with bilateral orchidectomy, or medically with LHRH agonists or antagonists or oral anti-androgens alone. (3) Oral anti-androgens were permitted in the trial but were used by very few patients and are no longer permitted for new patients within the trial from version 8.0.

ADT produces responses in up to 95% of patients but it is not curative and disease recurs in virtually all patients treated with ADT as sole therapy, with a median time to progression of 18-24 months. (3) Such disease is referred to as hormone-refractory or, increasingly, as castrate resistant prostate cancer (HRPC or CRPC); this latter term is unpopular with patient groups due to its perceived pejorative overtones related to castration and hence terminology may yet change again in the future.

### 2.1.2 ROLE OF RADIOTHERAPY FOR PATIENTS WITH M0 DISEASE

Two randomised trials, SPCG7 (4) and NCIC PR.3 / MRC PR07 (5-7) have tested the question of whether androgen deprivation therapy alone or combined with radiotherapy is the best treatment for high-risk patients with no evidence of spread outside the pelvis. Both trials demonstrate an improvement in overall and disease specific survival from the addition of radiotherapy to androgen deprivation therapy. The size of this overall survival benefit is substantial (hazard ratio 0.68 in SPCG7 and HR 0.77 in PR07). With substantial benefit demonstrated in two mature, large, well conducted randomised trials, we now recommend that radiotherapy be considered standard for patients with no nodal or metastatic spread. Patients in this category will now only be allowed to enter the trial if standard radiotherapy is planned, with the exception of those for whom radiotherapy is contra-indicated. Such patients should be discussed with the Trials Unit prior to inclusion. For patients with node positive, M0 disease there are no clear data on whether radiotherapy is or is not indicated. The NCIC PR.3 / MRC PR07 trial included patients with unknown nodal status who received whole pelvic radiotherapy. Given the large overall benefit observed in this trial, the STAMPEDE TMG recommends that pelvic nodal radiotherapy be considered for patients with node positive, non-metastatic disease at the discretion of the treating clinician.

## 2.2 RATIONALE

There are increasing numbers of treatments which are used post relapse of first-line androgen deprivation therapy in patients with CRPC, but little evidence as to which is associated with the best response or how they may be combined or sequenced or whether any of them might have a role in first-line treatment. Such treatments include further hormonal manipulations, bisphosphonates, (8), cytotoxic chemotherapy (9), new hormone therapies (10) and palliative radiotherapy. The traditional approach to the testing and introduction of new treatments for prostate cancer is to use them in

patients with castrate resistant disease. An alternative approach is to investigate new drugs and new approaches to treatment, as first-line therapy in patients starting androgen deprivation therapy. At this point, patients should be fitter and better able to tolerate treatment than when they have CRPC and there is the possibility of having a larger and longer lasting effect.

## 2.3 DESIGN

STAMPEDE (also known as MRC PR08) is an innovative, multi-arm multi-stage, multi-centre, randomised controlled trial. It initially assessed the effects of a bisphosphonate (zoledronic acid), a cytotoxic chemotherapeutic agent (docetaxel) and a cyclooxygenase (Cox-2) inhibitor (celecoxib), as single agents or combinations, in patients commencing androgen deprivation therapy for advancing or metastatic prostate cancer. Each comparison is divided into five stages such that, for each investigational arm, safety and activity data are generated in the first four stages; an investigational arm will only proceed to the fifth and final stage of recruitment, where it will be assessed for its effect on overall survival, if it has been shown to be sufficiently safe and active. It is important to note, however, that patient data from all arms and all stages will be included in the final analyses of the primary outcome measure, even if the investigational arm did not proceed to the final stage.

Planned interim analysis failed to demonstrate sufficient activity for celecoxib and this agent has now been removed from the trial recruitment; patients remaining on celecoxib treatment reverted to standard care. Protocol version 8.0 added a new drug abiraterone to the study as an additional arm (see Section 2.7). Protocol version 9.0 added a new comparison arm involving prostate radiotherapy for patients with metastatic disease (see Section 2.8). Protocol version 10.0 reflected the successful completion of recruitment to three docetaxel- and bisphosphonate-containing arms (Arms B, C and E) and removed references to these agents in the information sheets for new patients. Protocol version 11 extended the recruitment target for the abiraterone research comparison (A vs G) from 1,500 to 1,800 patients.   Current protocol 12.0 adds a new comparison involving the combination of abiraterone and enzalutamide.

### 2.4 RESEARCH TREATMENT AND BISPHOSPHONATES

**Note: recruitment stopped to the zoledronic acid and docetaxel-containing arms in Mar-2013 at the end of Activity Stage IV.**

The bisphosphonates are a class of drug that act by reducing osteoclast formation, inhibiting osteoclast activity and inducing osteoclast apoptosis. They are effective at controlling hypercalcaemia and preventing skeletal complications associated with malignant disease. (11, 12) Zoledronic acid is a highly potent, third generation bisphosphonate; studies comparing the efficacy of zoledronic acid to other bisphosphonates suggest that zoledronic acid has a 40-850 fold higher potency than clodronate in preclinical models of bone resorption. (13). It has also been shown to be more effective than pamidronate (90mg) in controlling malignant hypercalcaemia. (14) In addition, zoledronic acid has also demonstrated direct anti-cancer activity, including inhibition of proliferation of breast cancer and prostate cancer cells in vitro. (15)

In randomised controlled trials of 1,648 patients, 4mg zoledronic acid was more effective than pamidronate in reducing the risk of skeletal complications in patients with bone metastases from breast cancer. (16, 17) Also, in metastatic prostate cancer, zoledronic acid has been shown to reduce the rate of skeletal-related events compared to placebo in a trial involving 429 men. (18) In April 2002, zoledronic acid received approval from the Committee for Proprietary Medicinal Products for the prevention of skeletal-related events (for example, fractures) in patients with any advanced malignancies involving bone.

The MRC PR05 prostate cancer trial showed that a first generation bisphosphonate (clodronate) commenced at the time of androgen deprivation therapy initiation, delayed time to progression in patients with bony metastatic disease and there was some evidence that it may also improve survival. (19) There is, therefore, a good rationale for investigating a more potent bisphosphonate in patients with prostate cancer who are about to commence ADT therapy.

## 2.5 RESEARCH TREATMENT: CHEMOTHERAPY

**Note: recruitment stopped to the zoledronic acid and docetaxel-containing arms in Mar-2013 at the end of Activity Stage IV.**

There is increasing evidence of the clinical efficacy of chemotherapy in prostate cancer. (9) Two randomised phase III trials in patients with metastatic hormone refractory prostate cancer (HRPC) using a docetaxel-containing regimen have been completed: the SWOG 9916 study (20) and the TAX-327 study. (21) Both studies show that the use of a docetaxel-based regimen improved survival for patients with metastatic HRPC and had significantly greater PSA response rates compared to the mitoxantrone plus prednisolone arm.

In the TAX-327 trial, (21) 1,006 patients with metastatic HRPC were randomized to receive either mitoxantrone 12 mg/m² with prednisone 10mg daily (Arm C) or docetaxel 75mg/m² 3-weekly for 10 cycles with prednisone (Arm A) or docetaxel 30 mg/m²/wk x 5 of 6 weeks x 5 cycles with prednisone (Arm B). Median overall survival was 16.5 months for patients treated with mitoxantrone versus 18.9 months for the 3-weekly docetaxel regimen (hazard ratio 0.76 (0.62-0.94)). There was also improvements for 3-weekly docetaxel in pain (22% vs 35%, p = 0.01) and PSA response (32% vs 45%, p=0.0005).

In June 2006 in the UK docetaxel was given NICE (National Institute for Health and Clinical Excellence) approval for use in hormone (now more commonly termed castrate) refractory prostate cancer patients.

## 2.6 RESEARCH TREATMENT: CYCLOOXYGENASE-2 INHIBITORS

**Note: recruitment completed to both celecoxib-containing arms in Apr-2011 at the end of Activity Stage II of this comparison**

Cyclooxygenase-2 (Cox-2) is an isoenzyme induced by a variety of mitogens, cytokines and growth factors that are associated with a range of process including inflammation, (22) and carcinogenesis.(23, 24) There is a growing body of evidence that inhibition of Cox-2 may play an important role in the prevention of cancer and the delay of progression in established cancer. A number of case-control studies have shown a reduction in risk of prostate cancer associated with the use of non-steroidal anti-inflammatory drugs (NSAID), which include inhibition of Cox-2 amongst their mode of action. (25) Pathological studies show Cox-2 is upregulated in carcinomas (26) and one study suggested that NSAID use may delay progression from subclinical to clinical prostate cancer. (27)

Celecoxib, a Cox-2 inhibitor, is better tolerated than other NSAIDs and there is evidence that it is active as a chemoprevention agent. (28) It also has important antineoplastic properties such as the ability to inhibit angiogenic factors and induce apoptosis in human cancer cells including prostate cancer. (29)

Evidence has suggested that an anti-cancer effect is only seen at higher doses of celecoxib than **is** required for an anti-inflammatory effect. (30) Therefore, the dose of 800mg/day for STAMPEDE patients has been chosen. Although there is some high profile evidence of a small absolute increase in CVS toxicity risk associated with higher doses of celecoxib, (31) most current cancer trials are using a dose of 800mg/day as it is believed that a higher dose will result in a greater increase in cancer effect.

There is also some evidence of a schedule effect on CVS toxicity. It has been observed that CVS toxicity becomes evident after one year of taking celecoxib. (31) Therefore, a maximum duration of one year has been

set for celecoxib use in this trial. Any potential risks of course have to be weighed against any potential benefits of celecoxib in the delay of progression in established prostate cancer.

Given case-control data suggesting effects on prostate cancer, pathological expression of Cox-2 in prostate cancer and in vitro data suggesting that inhibition of Cox-2 inhibits growth and invasiveness, further investigation in prostate cancer is warranted.

## 2.7 RESEARCH TREATMENT: STEROID SYNTHESIS INHIBITORS

Recent evidence suggests that an important mechanism for escape from tumour control by androgen ablation is the intracellular conversion of steroid precursors to androgenic steroids by prostate cancer cells. A key enzyme in this process is CYP17, which therefore represents a logical target for therapy in CRPC. (10) Abiraterone acetate is a selective inhibitor of CYP17 and is highly active in patients developing resistance to standard androgen ablation therapies. (32-34) Recruitment to a phase III study comparing abiraterone acetate to placebo in CRPC patients post-docetaxel, completed accrual in 2009 and reported initial results in 2011 with an improvement in overall survival of around 4 months and a hazard ratio of 0.65. (35) The drug has now received a marketing authorisation in the USA and in the EU from September 2011. A second trial in pre-chemotherapy CRPC patients completed recruitment April 2010; preliminary results are positive and were published in 2012 (36) and the licence for abiraterone was extended to the pre-chemotherapy CRPC population in Europe in 2012. Side-effects with abiraterone acetate are modest with the main adverse effects being elevated transaminases (usually mild), hypokalaemia and hypertension due to secondary hyperaldosteronism and fluid retention (preventable by low doses of glucocorticoids). In order to prevent secondary hyperaldosteronism, it is recommended that prednisolone (or prednisone) 10mg daily be administered in the CRPC setting. Within more recent studies in earlier stage patients, lower doses (typically 5mg of prednisone/prednisolone) are being used due to concerns about long-term exposure to glucocorticoid side effects. More recent evidence even suggests that for most patients, no glucocorticoids may be needed. (37) Within the STAMPEDE trial, we suggest prednisolone/ prednisone dose of 5mg daily.

We hypothesise that the agent may be more active still when given up-front in combination with first-line androgen deprivation therapy by preventing or delaying the development of castrate refractory disease.

## 2.8 RESEARCH TREATMENT: RADIOTHERAPY TO THE PROSTATE FOR PATIENTS WITH NEWLY-DIAGNOSED METASTATIC DISEASE

Therapy directed against the primary tumour in the presence of metastatic disease has been evaluated rigorously in only one malignancy to date: renal cell carcinoma. Two cooperative groups ran randomised trials enrolling patients with previously untreated metastatic RCC whose primary tumours were amenable to surgical resection. Patients were randomized to receive the standard systemic therapy of the day, interferon-alpha, either alone or with radical nephrectomy. The combination of nephrectomy and interferon was shown to significantly improve median survival from 7 to 17 months in one trial (38) and from 8 to 11 months in the other.(39) The mechanism by which nephrectomy improves survival remains obscure. In preclinical models, the primary tumour has been found to secrete molecules that prime the microenvironment in which metastases can develop. An implication of this work is that therapy directed at the primary tumour, by abrogating this endocrine signalling, could retard the formation and the growth of distant metastases.

The results of two large-scale randomised trials of prostate radiotherapy are also provocative. The Scandinavian SPCG-7 trial and the MRC PR07 trial randomised men with locally advanced prostate cancer, who were at high risk of possessing occult metastatic disease, to either androgen deprivation therapy (ADT) alone or ADT plus prostate radiotherapy.(4, 40) The addition of radiotherapy

dramatically improved 10-year outcomes: mortality from prostate cancer was halved. Interestingly, the benefit of radiotherapy started to emerge as early as three years from the time of randomisation. This seems improbably early if the benefit of local treatment is mediated via the prevention of subsequent disease dissemination. Rather, it is more consistent with the possibility that local treatment has a beneficial impact on the rate of progression of existing micrometastatic disease.

We hypothesise that local therapy to the primary site may retard distant disease progression and prolong survival in patients with metastatic prostate cancer.

**Figure 8: Use of RT in STAMPEDE**

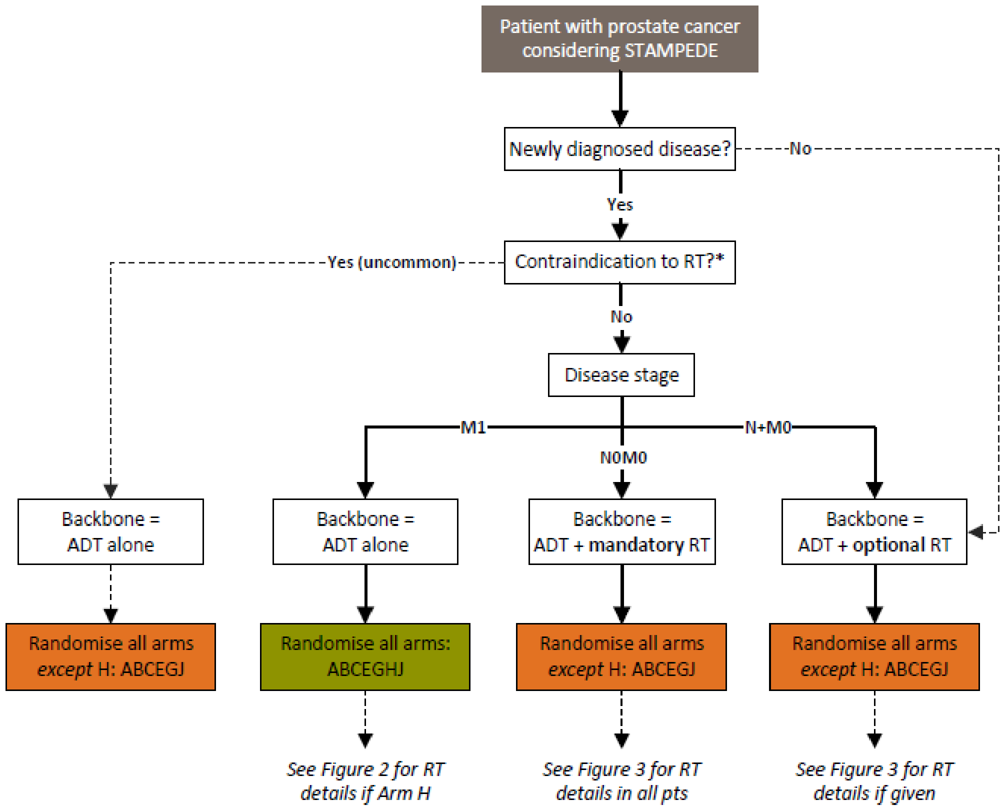


*It is expected that only around 1% of patients will have a contraindication to RT e.g. inflammatory bowel disease. These cases should be discussed with the trials unit prior to randomisation (see **Section 4.3**).

## 2.9 RESEARCH TREATMENT: COMBINATIONS OF ORIGINAL RESEARCH ARMS

### 2.9.1 Bisphosphonate And Chemotherapy

**Note: recruitment stopped to the zoledronic acid and docetaxel-containing arms in Mar-2013 at the end of Activity Stage IV.**

Zoledronic acid and docetaxel have different mechanisms of action. In addition to its skeletal protection activity, zoledronic acid has shown direct activity against prostate cancer cells, both in vitro and in vivo. (15)

There is also in vitro and in vivo evidence to suggest synergy between zoledronic acid and chemotherapy in breast cancer cells and anti-angiogenic effects in patients. (41, 42)

Toxicities of the two agents are complementary and administration in combination is expected to be feasible and safe. These aspects were evaluated in the initial Pilot Phase of the trial. Since both agents show considerable promise as single agents and there is in vitro evidence of synergy, we believe there is a strong rationale for evaluating these two agents in combination.

### 2.9.2 Bisphosphonate And Cyclooxygenase-2 Inhibitors

**Note: recruitment stopped to both celecoxib-containing arms in Apr-2011 at the end of Activity Stage II.**

An alternative approach to combination therapy is to target the principal site of relapse and a key mode of progression and this is the rationale for combining zoledronic acid with a Cox-2 inhibitor. Bisphosphonates have already been shown to delay bone disease progression in hormone refractory disease. (19) Cox-2 appears to play a crucial role in the molecular phenotype of advanced prostate cancer as outlined above, and this effect is likely to be apparent in both soft tissue and in bone. Toxicities of the two agents are likely to be complementary and there is no strong a priori reason to anticipate unacceptable toxicity. The Pilot Phase of the trial will evaluate tolerability and safety of the combination. Targeting both bone progression and the underlying molecular changes leading to progression can be expected to have synergistic benefits in terms of delaying development of hormone refractory disease.

## 2.10 COMBINATION OF STEROID SYNTHESIS INHIBITORS AND ANDROGEN RECEPTOR SIGNALLING INHIBITOR

The majority of patients with advanced prostate cancer who have disease progression on abiraterone or enzalutamide taken as single agents, have a rise in PSA, suggesting reactivation of androgen receptor (AR), or other steroid signalling pathways resulting in increased PSA transcription, is the pathway to the development of resistance.(43)

The question under investigation is: can progression be delayed (and survival extended) by using a combination of abiraterone and enzalutamide?

### 2.10.1 Supplementing Abiraterone And Prednisolone With Enzalutamide

Several studies have shown that the AR can become promiscuously activated by very low levels of androgens or other steroid metabolites and drugs that bind the AR.(44-47) It is known that very low levels of androgens can persist in patients treated with abiraterone acetate.(48) Drugs that bind the AR, may include co-administered glucocorticoids. Furthermore, AR mutations of the sort previously described in castration-resistant prostate cancer (CRPC), can be activated by cortisol and other glucocorticoids at levels much lower than those reported in patients treated with abiraterone and prednisolone at a dose of 5mg bid.(47, 49) Moreover, abiraterone binds the AR and although weak antagonism of wild-type and most previously described AR mutations are observed,(49) a similar mechanism to that described with classical anti-androgens, such as bicalutamide, could lead to change-of-function AR mutations associated with AR activation following abiraterone binding. Therefore, concomitant treatment with an androgen receptor signalling inhibitor could prevent “promiscuous” AR activation in patients treated with abiraterone. Enzalutamide is a androgen receptor signalling inhibitor and has gained recent approval for use on its own in the treatment of advanced CRPC,(50) and there is evidence of activity for hormone-naïve prostate cancer.(51)

### 2.10.2 Supplementing Enzalutamide With Abiraterone And Prednisolone

Enzalutamide in combination with ADT is effective and well tolerated in CRPC.(50) However, recent studies have suggested that intratumoral testosterone levels increase in patients treated with enzalutamide.(52) The implications of this finding are that the increase in intratumoral testosterone could be associated with up-regulation of enzymes involved in steroid biosynthesis.(53) Although enzalutamide has a high affinity for the AR, this is several-fold lower than both the natural ligands testosterone and DHT,(54) which means that enzalutamide would be out-competed at the AR ligand-binding domain if and when androgen levels rise. In vitro, a ten-fold rise in intra-cellular androgen was sufficient to prevent inhibition of AR by 30uM of enzalutamide;(49) these levels are representative of the plasma levels of enzalutamide active metabolites, which can be achieved with enzalutamide 160mg po daily.(55)

A strategy for preventing the rise in intra-cellular androgens in patients treated with enzalutamide would be inhibition of CYP17A1. Abiraterone is currently the only CYP17A1 inhibitor with proven efficacy. It therefore seems logical to use the combination of enzalutamide and abiraterone to both block a rise of intra-cellular androgens and prevent promiscuous activation of the AR.

### 2.10.3 Summary Of Rationale For This Combination

To date, investigation has focussed on patients with CRPC but there is a strong rationale for the combination of enzalutamide and abiraterone in the hormone treatment-naïve setting in which STAMPEDE is focused.

STAMPEDE already has an abiraterone plus conventional ADT arm but we will not assess the combination of conventional ADT plus enzalutamide; other trials by industry and other cooperative groups will address that question. The inclusion of an arm with ADT and enzalutamide in STAMPEDE was therefore considered to be a duplication of effort and was not supported by the Trial Management Group.

The combination of enzalutamide and abiraterone is a novel approach and offers considerable promise in delaying progression – it therefore represents an attractive addition to the comparisons under investigation in STAMPEDE, and one that is unlikely to be replicated in other planned trials of this size.

# 3 SELECTION OF INSTITUTIONS AND INVESTIGATORS

Centres who wish to participate in the STAMPEDE trial should be registered with the Medical Research Council Clinical Trials Unit at University College London (MRC CTU at UCL) for this purpose. Before any patients are randomised the MRC CTU must receive a completed and signed Investigator Statement. The STAMPEDE investigator statement is signed by the Principal Investigator for that institution (Appendix M). R&D approval for the site, along with a fully-signed model agreement, are also required before recruitment can begin.

In addition and in compliance with the principles of GCP all institutions participating in the trial will complete a delegation log and forward this to the MRC CTU. Each person working on the STAMPEDE trial must sign off a section of this log indicating their responsibilities. The MRC CTU must be notified of any changes to trial personnel and/or their responsibilities. An up-to-date copy of this log must be stored in the Investigator Site file at the institution and also at the MRC CTU.

The Clinical Trial Authorisation (CTA) for the STAMPEDE trial requires that the Medicines and Healthcare Products Regulatory Agency (MHRA) be supplied with the names and addresses of all participating investigators/institutions. Trial staff at the MRC CTU will perform this task; hence, it is vital to receive full contact details for all investigators prior to their entering patients.

Finally, before a patient is entered into the trial written informed consent must be obtained. Approved patient information sheets and informed consent forms are supplied as templates.

Only a limited number of centres participated in the initial Pilot Phase of the original trial; this was to ensure that safety and feasibility data were collected expediently. Subsequent stages of the trial are open to any centre that wishes to participate and has fulfilled the requirements described above.

## 3.1 RADIOTHERAPY ACCREDITATION

The introduction of the RT comparison in v9.0 introduced the need for RTQA accreditation in sites giving radiotherapy. The details of RTQA accreditation is in Appendix K. However, centres that have been RTQA accredited for another multi-centre prostate radiotherapy trial in the UK (e.g. RADICALS or CHHIP) will be automatically granted STAMPEDE RTQA accreditation.

# 4 SELECTION OF PATIENTS

## 4.1 PATIENT INCLUSION CRITERIA

Patients must fulfil both of the criteria in **Section 4.1.1** **or** one criterion in **Section 4.1.2** **or** at least one criterion in **Section 4.1.3**. Additionally, all patients must fulfil the criteria in **Section 4.1.4**.

### 4.1.1 High-Risk Newly-Diagnosed Non-Metastatic Node-Negative Disease

Both:

- At least two of: Stage T3/4, PSA≥40ng/ml or Gleason sum score 8-10
- Intention to treat with radical radiotherapy (unless there is a contra-indication; exemption can be sought in advance of consent, after discussion with MRC CTU)

OR

### 4.1.2 Newly-Diagnosed Metastatic Or Node-Positive Disease

At least one of:

- Stage $T_{any}$ N+ M0
- Stage $T_{any}$ $N_{any}$ M+

OR

### 4.1.3 Previously Treated With Radical Surgery And/Or Radiotherapy, Now Relapsing[1]

At least one of:

- PSA ≥4ng/ml and rising with doubling time less than 6 months
- PSA ≥20ng/ml
- N+
- M+

AND

### 4.1.4 For All Patients

I. Histologically confirmed prostate adenocarcinoma
II. Intention to treat with long-term androgen deprivation therapy
III. Fit for all protocol treatment[2] and follow-up, WHO performance status 0-2[3]
IV. Have completed the appropriate investigations prior to randomisation
V. Adequate haematological function: neutrophil count >$1.5x10^9$/l and platelets >$100x10^9$/l
VI. Estimated creatinine clearance >30ml/min
VII. Serum potassium ≥3.5mmol/L
VIII. Written informed consent
IX. Willing and expected to comply with follow-up schedule
X. Using effective contraceptive method if applicable

[1] Courses of hormone therapy for localised disease must have been completed at least 12 months previously and have been no longer than 12 months in duration. It can have been given as adjuvant or neoadjuvant therapy.
[2] Medical contraindications to the trial medications are given in **Appendix G**
[3] For WHO performance status definitions see **Appendix A**

## 4.2 PATIENT EXCLUSION CRITERIA[4]

Patients must not fulfil any of the criteria, below.

I. Prior systemic therapy for locally advanced or metastatic prostate cancer except as listed in **Section 4.1.3**
II. Metastatic brain disease or leptomeningeal disease
III. Abnormal liver functions consisting of any of the following:
   - Serum bilirubin ≥1.5 x ULN (except for patients with Gilbert's disease, for whom the upper limit of serum bilirubin is 51.3µmol/l or 3mg/dl)
   - Aspartate aminotransferase (AST) or alanine aminotransferase (ALT) ≥2.5 x ULN
IV. Any other previous or current malignant disease which, in the judgement of the responsible physician, is likely to interfere with STAMPEDE treatment or assessment
V. Patients with contra-indications to prednisolone, including active peptic ulceration or a history of gastrointestinal bleeding
VI. Patients with active inflammatory bowel disease
VII. Symptomatic peripheral neuropathy grade ≥2 (NCI CTC)[5]
VIII. Any surgery (e.g. TURP) performed within the past 4 weeks
IX. Patients with significant cardiovascular disease such that, in the investigator's opinion, the patient is unfit for any of the study treatments. This might include:
   - Severe/unstable angina
   - Myocardial infarction less than 6 months prior to randomisation
   - Arterial thrombotic events less than 6 months prior to randomisation
   - Clinically significant cardiac failure requiring treatment (NYHA II-IV)[6]
   - Cerebrovascular disease (e.g. stroke or transient ischaemic episode) less than 2 years prior to randomisation
   - Patients with uncontrolled hypertension defined as systolic BP greater or equal than 160 mmHg or diastolic BP greater or equal than 95 mmHg
X. Patients receiving treatment with drugs known to induce CYP3A4 (including phenytoin, carbamazepine, Phenobarbital)[7]
XI. Prior exposure to abiraterone
XII. Prior exposure to enzalutamide
XIII. Prior chemotherapy for prostate cancer
XIV. Prior therapy with zoledronic acid or other bisphosphonates other than treatment for hypercalcaemia or low bone density
XV. Prior exposure to policy of long-term hormone therapy before randomisation (unless as described in **Section 4.4.2**)
XVI. History of seizure including any febrile seizure, loss of consciousness, or transient ischaemic attack within 12 months of randomisation or any condition that may pre-dispose to seizure (e.g., prior stroke, brain arteriovenous malformation, head trauma with loss of consciousness requiring hospitalization)
XVII. Unexplained history of loss of consciousness within 12 months of randomisation
XVIII. Operation of heavy machinery during treatment

[4] The exclusion criteria for patients who have been on a Cox-2-inhibitor for 6+ months has been removed
[5] See **Appendix I** for common toxicity grading
[6] NYHA classifications can be found in **Appendix A**
[7] A full list is included in **Appendix G**

## 4.3 SELECTION CRITERIA FOR COMPARISON OF RESEARCH (M1) RT FOR METASTATIC DISEASE

All patients meeting criteria in **Section 4.1** and **4.2** are eligible for the trial, but not all can be allocated to the research (M1) radiotherapy arm. The selection criteria for this "RT to the prostate" comparison are:

- Newly-diagnosed prostate cancer
- Demonstrable M1 disease
- No contraindication to radiotherapy e.g. no previous pelvic radiotherapy and no history of inflammatory bowel disease
- No previous radical prostatectomy

Any patients meeting these criteria will have a chance to be allocated to Arm H.

## 4.4 SCREENING PROCEDURES

### 4.4.1 INVESTIGATIONS PRIOR TO RANDOMISATION

All patients should have the following examinations performed. The latest available scans should be used:

- CT or MRI of pelvis and abdomen
- Bone Scan (or equivalent e.g. whole body MRI)
- Chest X-ray (only if chest was not included in CT)
- ECG
- PSA Test

The following blood tests within 8 weeks (56 days) prior to randomisation:

- Testosterone (if available)
- Urea and Electrolytes
- Liver function tests
- Serum creatinine
- Serum corrected calcium
- Phosphates
- Magnesium
- Albumin
- Total cholesterol
- HDL cholesterol
- Systolic blood pressure
- Diastolic blood pressure
- Waist circumference measure

Patients who initially fail to meet the eligibility criteria can be re-screened at a later date.

Prior to randomisation:

- Check details of any prior treatments for prostate cancer
- Check any contraindications to radiotherapy

### 4.4.2 Androgen Deprivation Therapy Prior To Randomisation

It is preferable that patients are not started on hormones prior to randomisation. However, if androgen deprivation therapy has already started, the primary therapy should have not have started more than 12 weeks before randomisation, and the baseline PSA measurement must be taken before this was initiated (please report the latest PSA measurement taken before the start of androgen deprivation therapy).

Short periods of prior anti-androgens to cover tumour flare are allowed but will not be counted in the 12 week time period mentioned above; but a PSA measurement must be taken before this is initiated.

Note that long-term anti-androgen monotherapy is not permitted in the trial for newly recruited patients from version 8.0 (see **Section 6.1**); patients may change treatment to join the trial, provided that they have not had more than 12 weeks of androgen deprivation therapy prior to randomisation. Further details on hormone therapies allowed prior to randomisation are discussed in Appendix L.

Any relapsing patients treated with adjuvant or neo-adjuvant hormone therapy alongside their radical surgery or radiotherapy must have completed that period of hormone therapy at least 12 months before joining STAMPEDE and it must have been no longer than 12 months in duration .

Note that baseline testosterone measurements will not be required in patients who have already commenced hormone manipulation prior to randomisation.

### 4.4.3 Hypercalcaemia At Randomisation

For patients who are hypercalcaemic prior to randomisation and require treatment, it is recommended that they are treated with a bisphosphonate and that the treatment should be discontinued when they are stabilised.

### 4.4.4 NSAIDs And Cox-2 Inhibitors At Randomisation

**Note: recruitment completed to both celecoxib-containing arms in Apr-2011 at the end of Activity Stage II**

For patients who are currently on a Cox-2-inhibitor and who meet the inclusion criteria, please ensure that treatment is discontinued before randomisation. If the patient is allocated to an arm, which does not include celecoxib (arms A, B, C or E), it is advised that the Cox-2 be replaced with a suitable NSAID.

For patients who are taking an NSAID prior to randomisation and are allocated a celecoxib arm (Arm D or F), a clinical decision should be taken as to whether the patient should continue taking the NSAID alongside the celecoxib. This decision should take into account the risk of gastrointestinal problems, and consideration should be given to the co-administration of a proton pump inhibitor

### 4.4.5 Starting Trial Treatment

Trial treatment should be commenced as soon as possible after randomisation. Investigators should aim that this is at least within 4 weeks post randomisation and within 12 weeks of starting androgen Deprivation Therapy (see Section 6).

Radiotherapy for patients allocated to Arm H should be commenced within 4 weeks from randomisations and continued according to the predefined scheduled unless toxicity is reported. Any delays in starting research radiotherapy should be discussed with the STAMPEDE team and recorded as appropriate in the relevant CRF.

#### 4.4.6 Concomitant Medications

All concomitant medications should be recorded including any vitamin and mineral supplements the patient is taking, regular consumption of NSAID and/or aspirin and use of other bisphosphonates (see **Section 4.3.1**). Of particular interest in this are herbal preparations such as PC-SPES, Prostasol, Saw Palmetto and St John's Wort. All concomitant medications should be continued throughout the trial unless the responsible clinician decides otherwise.

### 4.5 ADDITIONAL DETAILS FOR PATIENTS JOINING SUB-STUDIES

An additional droplet of blood must be taken if the patient has given their consent to participate in the DNA analysis sub-study.

The local pathologist will also be asked to give the tumour sample remaining after primary interrogation for tissue micro array analysis to be carried out, if the patient has given consent for his remaining samples to be used for further analyses. Full details of all sub-studies and instructions relating to the handling of the blood sample are given in **Section 17** and **Appendix D**.

# 5 RANDOMISATION AND ENROLMENT

Patients will be allocated to any of the open research arms for which they are suitable. Patients with non-metastatic disease or who have had previous local therapy to the prostate or who have a contraindication to radiotherapy will not be allocated to Arm H (see **Section 4.3**).

To enter a patient the randomisation form should be completed carefully and the MRC CTU contacted by phone:

**RANDOMISATIONS**
To randomise, call MRC CTU at UCL, Monday to Friday 0900-1700
excluding public holidays or dates when notice has been given by the CTU.
Tel: +44 (0) 20 7670 4777

A trial number and treatment will be allocated and given over the phone or by return fax. In addition, a letter confirming these details will be sent. The trial number will be the primary way in which the patient will be identified and should be used in all correspondence.

## 5.1 CO-ENROLMENT GUIDELINES

Ideally, patients should not be participating in any other clinical trial of prostate cancer treatment when they enter STAMPEDE and should not enter any other trials until the patient has had a failure-free survival (FFS) event reported. After this point, the patient may be entered into further, second-line treatment studies. The primary outcome measure of STAMPEDE is overall survival. Participation in post-progression studies should be reported on the Co-enrolment CRF.

Data sharing agreements with "down-stream" trials are encouraged to improve data quality in both trials and to reduce costs to both organisations.

# 6 TREATMENT OF PATIENTS

## 6.1 TRIAL TREATMENT

Patients will be randomised to the control arm (Arm A) or one of the research arms. All patients will receive androgen deprivation therapy (ADT) to achieve castration levels of testosterone. The method of ADT is a local choice but must be specified for each patient prior to randomisation. The recommended methods of ADT are given in **Section 6.1.1**. All trial treatments should commence as soon as practically possible after randomisation. Patients having a bilateral orchidectomy should commence any additional treatment within 12 weeks of the operation unless there is a strong clinical reason not to do so. Note that from protocol version 8.0 onwards, bicalutamide monotherapy is no longer permitted as a trial therapy for new patients (but patients may switch to a permitted therapy to join the trial – see **Section 4.3.2**).

## 6.2 ARM A: ADT ALONE OR ADT + STANDARD-OF-CARE (M0) RT (CONTROL ARM)

The standard of care for this patient group is **androgen deprivation therapy** (see **Section 6.2.1**). For some patient groups, this should now be supplemented with standard radiotherapy (see **Section 6.2.2**).

### 6.2.1 HORMONE THERAPY

The permitted methods of ADT are bilateral orchidectomy, LHRH analogues and LHRH antagonists. Anti-androgens alone are not permissible as hormone therapy for patients participating in STAMPEDE, but their use is recommended in the short-term to prevent tumour “flare” which may occur after commencing LHRH analogues. Anti-androgen prophylaxis of tumour flare is not required when using LHRH antagonists. At the time of randomisation, centres will be asked to specify the method of ADT for each patient. Other methods of ADT should be discussed with the Chief Investigator or the Trial Surgeon. The planned duration of ADT should be at least 2 years.

**Bilateral orchidectomy:** Operations should be performed by appropriately trained surgeons. A total or subcapsular orchidectomy may be performed.

**LHRH agonists**: LHRH agonists/analogues used according to local practice. The prophylactic use of anti-androgens to prevent tumour “flare” is recommended.

**LHRH antagonists:** LHRH antagonists used according to local practice. The use of prophylactic use of anti-androgens to prevent tumour “flare” is not necessary.

### 6.2.2 STANDARD-OF-CARE (M0) RT

**N0M0 patients:** Investigators should give standard radiotherapy (RT) to patients with node negative, non-metastatic disease (N0M0), in accordance with the data from the PR07 and SPCG trials. If there is an intention to omit radiotherapy (e.g contraindications) in patients with N0M0 disease this must be discussed with the Trials Office before consent. See **Section 6.6** for further details of radiotherapy administration.

**N+M0 patients:** the benefit of radiotherapy in this group is at present uncertain with no firm data to either support or refute its use. However, the PR07 trial included some node positive patients as

cross sectional imaging was not a part of the baseline assessment in this trial, which did include whole pelvis radiotherapy. For patients with node positive, non-metastatic disease, radiotherapy is therefore recommended in suitable cases. Investigators will be asked to state their intention with regards to planned radiotherapy in this group at randomisation. Intention to give radiotherapy (or not) for node positive patients must be stated at randomisation to ensure that there is no bias towards particular combinations of systemic therapy with radiotherapy.

Standard radiotherapy is not a core part of the trial, therefore we intend to collect minimal data about the radiotherapy administered. It is accepted that some patients will develop progressive disease before radiotherapy can be administered and if this occurs the reasons for non-delivery of treatment must be recorded on the radiotherapy form.

## 6.3 ARM B: ADT + ZOLEDRONIC ACID

**Note: recruitment stopped to the zoledronic acid and docetaxel-containing arms in Mar-2013 at the end of Activity Stage IV**.

Androgen deprivation therapy (+/- standard-of-care M0 RT) as described in **Section 6.2.1**.

Zoledronic Acid: 4mg 15min IV infusion every 3 weeks, for 6 treatments followed by zoledronic acid 4mg 15min IV infusion every 4 weeks up to a maximum of 2 years from the start of the treatment or until disease (including PSA) progression (see **Section 7.2**). Patients should also receive an oral supplement of 500mg calcium and 400IU vitamin D daily. These doses are available as a combination tablet. See **Section 6.6** for further information.

## 6.4 ARM C: ADT + DOCETAXEL

**Note: recruitment stopped to the zoledronic acid and docetaxel-containing arms in Mar-2013 at the end of Activity Stage IV.**

Androgen deprivation therapy (+/-standard-of-care M0 RT) as described in Section 6.2.1.

Docetaxel: 75mg/m2 Day 1 as 1hr IV infusion, plus prednisolone 5mg bid daily for 21 days. The cycle should be repeated every 3 weeks for a maximum of 6 cycles. The recommended administration schedule, anti-emetic regimen and dose modifications for docetaxel are given in Appendix F. See Section 6.2.2 for further information.

## 6.5 ARM D: ADT + CELECOXIB

**Note: recruitment completed to both celecoxib-containing arms in Apr-2011 at the end of its Activity Stage II**

Androgen deprivation therapy as described in Section 6.2.1.

Celecoxib 400mg bid until the sooner of 1 year or disease (including PSA) progression (see Section 7.2). See Section 6.2.3 for further information.

## 6.6 ARM E: ADT + DOCETAXEL + ZOLEDRONIC ACID

**Note: recruitment stopped to the zoledronic acid and docetaxel-containing arms in Mar-2013 at the end of Activity Stage IV.**

Androgen deprivation therapy (+/- standard-of-care M0 RT) as described in **Section 6.2.1**.

Docetaxel: 75mg/m2 Day 1 as 1hr IV infusion, plus prednisolone 5mg bid daily for 21 days. The cycle should be repeated every 3 weeks for a maximum of 6 cycles. The recommended administration schedule, anti-emetic regimen and dose modifications for docetaxel are given in Appendix F. See Section 6.4 for further information.

Zoledronic Acid: 4mg 15min IV infusion every 3 weeks, for 6 treatments followed by zoledronic acid 4mg 15min IV infusion every 4 weeks up to a maximum of 2 years from the start of the treatment or until disease (including PSA) progression (see **Section 7.2**). Patients should also receive an oral supplement of 500mg calcium and 400IU vitamin D daily. These doses are available as a combination tablet. See **Section 6.3** for further information.

Co-administration of docetaxel and zoledronic acid: Docetaxel 75mg/m2 Day 1 as 1hr IV infusion, plus prednisolone 5mg bid daily followed by zoledronic acid 4mg 15min IV infusion. There is evidence to suggest that the co-administration of docetaxel and zoledronic acid is sequence dependent.(42) Consequently, docetaxel should be administered before zoledronic acid

## 6.7 ARM F: ADT + ZOLEDRONIC ACID + CELECOXIB

**Note: recruitment completed to both celecoxib-containing arms in Apr-2011 at the end of Activity Stage II**

Androgen deprivation therapy as described in **Section 6.2.1**.

Zoledronic Acid 4mg 15min IV infusion every 3 weeks, for 6 treatments followed by zoledronic acid 4mg 15min IV infusion every 4 weeks up to a maximum of 2 years from the start of the treatment or until disease (including PSA) progression (see Section 7.2). Patients should also receive an oral supplement of 500mg calcium and 400IU vitamin D daily (Calcichew). These doses are available as a combination tablet. See **Section 6.3** for further information.

Celecoxib 400mg bid until the sooner of 1 year or disease (including PSA) progression (see Section 7.2). See Section 6.5 for further information.

## 6.8 ARM G: ADT + ABIRATERONE

**Note: recruitment to the "abiraterone comparison" completed in January 2014. Please note that some patients will continue treatment until progression or up to a maximum of 2 years. Please see sections below for more information**

Androgen deprivation therapy (+/- standard-of-care M0 RT) as described in **Section 6.2.1.**

Abiraterone will be administered as a single 1000mg daily oral dose (4 tablets to be taken together once a day) together with prednisolone or prednisone 5mg daily to prevent secondary mineralocorticoid excess. Abiraterone absorption is increased by food. The tablets should be taken at least 2 hours after food, swallowed whole with some water. No food should be eaten for 1 hour afterwards.

Prednisolone (prednisone in Switzerland) should be taken as a single dose with food in the morning.

In patients with M1 disease, treatment with abiraterone will continue from randomisation until clinical disease progression, consistent with the COU-AA-301 trial (35) i.e., abiraterone would be given for these patients until a composite of PSA progression (as defined in Appendix J), radiological progression (appearance of new lesions or progression of existing lesions) and clinical progression (defined as new cancer-related symptoms). It is accepted that these flexible criteria for stopping treatment with abiraterone are open to the investigator's interpretation and discretion. Patients might continue treatment beyond the first failure-free survival (FFS) event (see Table 1 in **Section 9.2**); the first FFS event must be reported as per the other arms.

In patients with N0M0 disease or N+M0 disease undergoing radical radiotherapy, treatment would continue for 2 years or disease progression as defined for M1 patients, whichever is the sooner. ADT can be discontinued in this group at 2 years at the discretion of the local investigator (see **Section 6.2.1**).

For patients with N+M0 disease not planned for radical radiotherapy, treatment will continue as for patients with M1 disease until disease progression. Trial treatment must stop if other systemic treatments are initiated (such as anti-androgen therapy for biochemical failure).

If a patient allocated to Arm G develops only biochemical failure, the responsible clinician might switch from abiraterone + prednisolone 5mg od to abiraterone and dexamethasone 0.5mg od. Trial treatment must stop if other systemic treatments are initiated at any time for disease progression control (including the addition or swapping of anti-androgens, chemotherapy etc).

See **Section 6.2.4 and 6.2.6** for further information for all groups.

## 6.9 ARM H: ADT + PROSTATE RADIOTHERAPY IN M1 PATIENTS

**Androgen deprivation therapy** as described in **Section 6.2.1**.

**Radiotherapy** will commence as soon as practicable and ideally within four weeks after randomization. Treatment will be according to the guidelines in **Section 6.11.5**. Two radiotherapy dose-fractionation schedules are permitted:

- 36Gy in 6 fractions of 6Gy, administered weekly over 6 consecutive weeks
- 55Gy in 20 fractions of 2.75Gy, administered daily, five days per week, over 4 consecutive weeks

Details of the recommendations for outlining, CTV and PTV are in **Section 6.11.5**.

## 6.10 ARM J: ADT + ABIRATERONE + PREDNISOLONE + ENZALUTAMIDE ADMINISTRATION

**Androgen deprivation therapy** (+/- standard-of-care M0 RT) as described in **Section 6.2.1**.

**Abiraterone** as described in **Section 6.8**.

**Prednisolone** as described in **Section 6.8**.

**Enzalutamide** will be administered as a 160mg oral dose (four capsules), taken together at the same time every day, with or without food.

In patients with M1 disease, treatment with both abiraterone and enzalutamide will continue from randomisation until clinical disease progression, consistent with the approach taken for abiraterone (see **Section 6.8**) i.e., abiraterone and enzalutamide would be given for these patients until a composite of PSA progression (as defined in **Appendix J**), radiological progression (appearance of new lesions or progression of existing lesions) and clinical progression (defined as new cancer-related symptoms). It is accepted that these flexible criteria for stopping treatment with abiraterone and enzalutamide are open to the investigator's interpretation and discretion. Patients may continue treatment beyond the first failure-free survival (FFS) event (see Table 1 in Section 9.2); the first FFS event must be reported as per the other arms.

In patients with N0M0 disease or N+M0 disease undergoing radical radiotherapy, treatment would continue for 2 years or disease progression as defined for M1 patients, whichever is the sooner. ADT can be discontinued in this group at 2 years at the discretion of the local investigator (see **Section 6.2.1**).

For patients with N+M0 disease not planned for radical radiotherapy, treatment will continue as for patients with M1 disease until disease progression.

Trial treatment must stop if other systemic treatments are initiated at any time for disease progression or control (including the addition or swapping of anti-androgens, chemotherapy etc).

See **Section 6.2.4** and **Section 6.2.6** for further information for all groups.

## 6.11 ADMINISTRATION AND DOSE MODIFICATIONS

### 6.11.1 ZOLEDRONIC ACID

**Note: recruitment stopped to the zoledronic acid and docetaxel-containing arms in Mar-2013 at the end of Activity Stage IV.**

Zoledronic acid will be administered by IV infusion in accordance with the instructions in the summary of product characteristics at a target dose of 4mg (adjusted for renal function, see below) every 3 weeks for the first 6 cycles and every 4 weeks, thereafter.

Serum Creatinine Measurements: Serum creatinine should be measured at baseline and within 48 hours prior to every administration of zoledronic acid. It is permissible to have serum creatinine levels measured on Fridays prior to the administration of zoledronic acid on the following Monday.

Serum Electrolytes and FBC: Serum electrolytes including calcium, phosphate and magnesium should also be measured prior to each infusion. FBC should be measured at least 3 monthly. Zoledronic acid should be discontinued if there is any evidence of hypersensitivity to the drug. In patients with mild to moderate renal impairment, lower doses of zoledronic acid are recommended according to standard dose reduction schedules for administration of this drug. In rare cases, zoledronic acid treatment has been associated with the development of osteonecrosis of the jaw, particularly following dental extractions. If a patient develops osteonecrosis of the jaw then the zoledronic acid should be immediately and permanently discontinued. For full details of zoledronic acid administration and does reductions see **Appendix F**. Contraindications, special precautions, interactions and side effects are listed in **Appendix G**.

### 6.11.2 DOCETAXEL

**Note: recruitment stopped to the zoledronic acid and docetaxel-containing arms in Mar-2013 at the end of Activity Stage IV.**

The use of docetaxel should be confined to units specialised in the administration of cytotoxic chemotherapy and it should only be administered under the supervision of a physician qualified in the use of anticancer chemotherapy.

Docetaxel will be administered by IV infusion in accordance with the instructions in the summary of product characteristics at a dose of 75mg/m2 (up to a maximum dose of 160mg) on Day 1 of the study treatment period and then every 3 weeks thereafter for a maximum of 6 doses. Patients with a body surface area (BSA) greater than 2.13m$^2$ should be dosed as though they have a BSA of 2.13m$^2$. No ideal weight should be used for BSA calculations. Prednisolone or prednisone 5mg bid will be given until completion of chemotherapy. Additional dexamethasone should be given pre- and post-docetaxel infusion to suppress allergic reactions.

Please note that liver function test (LFTs) should be carried out within a week before the first cycle of docetaxel if an anti-androgen has been administered. This is due to an increased risk of neutropenia associated with docetaxel use following anti-androgen administration. Treatment should be delayed if LFTs are abnormal.

For full details of premedication schedule, recommended anti-emetic regimen and dose modifications for docetaxel (see Appendix F). Contraindications, special precautions, interactions and side effects are listed in Appendix G.

Docetaxel in combination with prednisone or prednisolone is indicated for the treatment of patients with hormone refractory metastatic prostate cancer.(20, 21)

### 6.11.3 CELECOXIB

**Note: recruitment completed to both celecoxib-containing arms in Apr-2011 at the end of Activity Stage II. No new patients should be receiving this agent now within the trial.**

Celecoxib should be administered in accordance with the instructions in the summary of product characteristics at a dose of 400mg bid orally. Rarely this drug is poorly tolerated and in this instance should be discontinued; particular care should be taken with patients with a history of gastrointestinal disease and patients with significant risk factors for cardiovascular events (see Appendix G). Patients with confirmed severe cardiovascular history should not be in STAMPEDE (see exclusion criteria, Section 4.2). Contraindications, special precautions, interactions and side effects are listed in Appendix G. Dose reductions are not anticipated.

### 6.11.4 Abiraterone or Enzalutamide + Abiraterone

Abiraterone absorption is increased by food. The tablets should be taken at least 2 hours after food, swallowed whole with some water. No food should be eaten for 1 hour afterwards. Prednisolone (prednisone in Switzerland) should be taken as a single dose with food in the morning.

Enzalutamide can be taken with or without food.

If clinical symptoms or signs suggestive of hepatotoxicity develop, serum transaminases, in particular serum alanine aminotransferase (ALT) should be measured immediately. If a rise in transaminases or bilirubin is confirmed, action should be taken as detailed in **Appendix G**.

#### 6.11.4.A Management of Specific Toxicities from Abiraterone

The safety monitoring and toxicity management plan described below takes into account AEs based on the reported clinical safety data of abiraterone.

**Hypokalemia:**
At the initial observation of **Grade 1** hypokalemia (serum potassium <3.5mM or below lower limit of normal range, but ≥3.0 mM), oral potassium supplement will be initiated. The dose of potassium supplement must be carefully titrated to maintain serum potassium at ≥3.5mM but ≤5.0mM. Any subject with low potassium while on study or a history of hypokalemia from a pre-existing or concurrent medical condition will undergo weekly or more frequent laboratory electrolyte evaluation. The investigator should consider maintaining potassium level at ≥4.0mM in these subjects.

If any subject experiences **Grade 3** hypokalemia (serum potassium levels <3.0mM–2.5mM, NCI CTCAE v4.0) or life-threatening hypokalemia with potassium levels <2.5mM (NCI CTCAE v4.0 hypokalemia grade 4), abiraterone will be discontinued and the subject will be hospitalized for intravenous potassium replacement and cardiac monitoring. After the return of serum potassium to normal, prednisolone will be discontinued but the patient can be maintained on enzalutamide.

**Hypertension:**
If **Grade 1-2:** Management per investigator with anti-hypertensive treatment.

If **Grade 3-4:** Withhold abiraterone. Adjust or add anti-hypertensive medications to mitigate the toxicity. When hypertension resolves to **Grade ≤1**, resume abiraterone at full dose with prednisolone 5mg bid. Enzalutamide can be continued.

**Fluid retention/oedema:**
If **Grade 1-2:** Increase prednisolone dose to 5mg bid.

If **Grade 3-4:** Withhold abiraterone. Consider addition of mineralocorticoid receptor antagonist eplerenone until resolution of symptoms. When fluid retention/oedema resolves to ≤Grade 1, resume abiraterone at full dose with prednisone 5mg bid. Enzalutamide can be continued. Abiraterone may be re-started when symptoms return to baseline or are equivalent to grade 1; if oedema does not resolve, abiraterone should not be re-started.

**Abnormal liver function tests:**
If **Grade 1** increases in AST, ALT or bilirubin occur (eg, increase in AST or ALT from ULN to 2.5 x ULN; increase in total bilirubin from ULN to 1.5 x ULN): the frequency of liver function test monitoring should be increased, if the investigator judges that the laboratory abnormalities are potentially related to study medication. No dose reduction is required.

If **Grade 2** increases in AST, ALT or bilirubin occur (eg, increase in AST or ALT to >2.5-5 x ULN; increase in total bilirubin from >1.5-3 x ULN): the frequency of liver function test monitoring should be increased to ≥once a week, if the investigator judges that the laboratory abnormalities are potentially related to study medication. No dose reduction is required.

If **Grade 3** or higher increases in AST, ALT, or bilirubin occur (eg, increase in AST or ALT to >5 x ULN; increase in total bilirubin to >3 x ULN), withhold abiraterone and all other concomitant medications that are potentially hepatotoxic. Frequent laboratory evaluations (at least once weekly) should be conducted until the liver function tests return to baseline value or grade 1. If study treatment resumption is considered for subjects who have experienced grade 3 increases in AST, ALT, or bilirubin, resume abiraterone with the first dose level reduction (3 tablets, 750 mg of study treatment) when grade 3 toxicities resolve to grade 1 or baseline.

If **Grade 4** increases in AST, ALT, or bilirubin occur (eg, increase in AST or ALT to >20 x ULN; increase in total bilirubin to >10 x ULN), subjects must discontinue abiraterone and enzalutamide immediately. They should be followed-up until resolution of abnormal liver function tests and then prednisone can be discontinued and the investigator can consider restarting enzalutamide.

### 6.11.4.B Management of Specific Toxicities from Prednisolone
Prednisolone or prednisone will be started at 5mg once daily, to prevent secondary mineralocorticoid excess. Prednisolone/prednisone dose increase of up to 10mg/day is permitted to manage mineralocorticoid-related toxicities (e.g., hypokalaemia, hypertension) which are refractory to standard management. Patients experiencing serious Cushing symptoms (e.g., weight gain, muscle loss) can decrease or discontinue (temporarily or permanently) steroids at the investigator's discretion. It should be noted that weight gain and muscle loss are also associated with androgen deprivation therapy.

### 6.11.4.C Management of Specific Toxicities from Enzalutamide
If any subject suffers a seizure whilst on treatment, enzalutamide should be discontinued. Abiraterone and prednisolone can be continued if the subject is not suffering from any abiraterone-specific toxicities.

**Fatigue:**
If **Grade 1-2:** No change in treatment.

If **Grade 3-4:** Withhold abiraterone and enzalutamide. Restarting of all treatments with a dose reduction of enzalutamide to 80mg/day can be considered when fatigue resolves.

### 6.11.4.D Management of Specific Toxicities from Combination of Enzalutamide + Abiraterone
To date no specific toxicities from the combination of abiraterone and enzalutamide have been described (N = 57 patients with mCRPC exposed for a median of 5.5 months).(56)

### 6.11.5 Research (M1) Prostate Radiotherapy

A treatment planning CT scan will be acquired with the patient supine, with empty rectum and comfortably full bladder.

Megavoltage equipment is required with effective photon energies ≥6MV. Minimum source-to-axis distance is 100cm. Field arrangement is at the clinician's discretion: acceptable treatment techniques (field arrangement) include a 3-field (anterior, right lateral, and left lateral), 4-field (anterior, posterior, right lateral, and left lateral), or 6-field (right and left anterior oblique, right and left posterior oblique, and right and left lateral) or equivalent coplanar technique with multi-leaf collimation for all fields to adequately protect normal structures.

The Clinical Target Volume (CTV) will consist of the prostate gland alone as visualized on the treatment-planning CT scan. The base of the seminal vesicles may also be included if they are macroscopically involved. Inclusion of pelvic lymph nodes in the CTV is not permitted. The Planning Target Volume will have a 0.8 cm margin posteriorly and 1.0 cm margin in all other directions around the CTV to account for prostate gland motion and uncertainty in daily treatment setup.

Critical normal tissues should be delineated on the treatment-planning CT scan by the treating clinician:

- Rectum – inferior limit: level of ischial tuberosities; superior limit: sigmoid flexure
- Bladder – entirety

Two radiotherapy dose-fractionation schedules are permitted. In either case, radiotherapy is prescribed such that the PTV receives at least 95% of the prescribed dose:

- 36Gy in 6 fractions of 6Gy, administered weekly over 6 consecutive weeks
- 55Gy in 20 fractions of 2.75Gy, administered daily, five days per week, over 4 consecutive weeks

Dose-volume objectives for each dose-fractionation schedule are shown in **Tables 2** and **3** below. Values have been calculated using the formula BED = D[1+d/(alpha-beta ratio)] assuming an alpha-beta ratio of 3 for rectum and bladder. These are provided for guidance only.

Portal imaging to verify accuracy of treatment delivery may be done according to the participating centre's local guidelines. Image-guidance technology (e.g., gold seed intraprostatic fiducial markers, cone-beam CT scanning) will be permitted according to clinician preference but is not required. Further illustration on the research radiotherapy arm schedule is shown in **Figure 9**.

**Table 2: Rectal dose volume objectives**

| 55Gy/20F | 36Gy/6F | Max Vol (%) |
|---|---|---|
| 52.5 Gy | 33.3 Gy | 50% |
| 43.5 Gy | 27.8 Gy | 60% |
| 26.1 Gy | 16.7 Gy | 80% |

**Table 3: Bladder dose-volume objectives**

| 55Gy/20F | 36Gy/6F | Max Vol (%) |
|---|---|---|
| 52.2 | 33.3 | 25% |
| 43.5 | 27.8 | 50% |

**Figure 9: Diagram for deciding approach to research (M1) RT to the prostate**

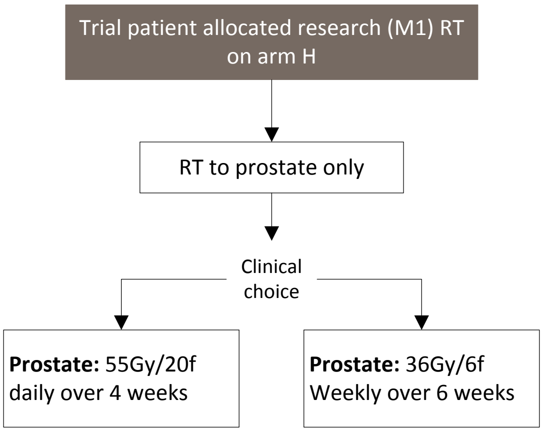


## 6.12 TRIAL PRODUCTS

Details of the procedures for obtaining the drugs within the trial, dispensing and disposal of unused drug are given in **Appendix E**.

Arrangements for free or discounted drugs are given in the Finance section (**Section 15**).

## 6.13 MEASURES OF COMPLIANCE/ADHERENCE

Date of treatment, dose, delays and reasons for delays or dose modifications of all study infusions (zoledronic acid and docetaxel) will be recorded. The estimated number of abiraterone tablets and enzalutamide capsules taken in a given time period will also be recorded as well as any dose reductions.

## 6.14 TREATMENT DATA COLLECTION

Data will be recorded on case report forms (CRFs); the top copy/original should be sent to the MRC CTU for data entry and a copy kept at the local centre. Up-to-date versions of all CRFs can be found on the trial website (http://www.stampedetrial.org/) and centres will be notified of any changes throughout the course of the trial. The type of data to be recorded is detailed in the Assessments and Procedures section (**Section 7**).

## 6.15 ADMINISTRATION OF STANDARD RADIOTHERAPY[8] TO NON-METASTATIC PATIENTS

### 6.15.1 TREATMENT DETAILS

Standard radiotherapy will be given to appropriate patients in each of the trial arms, following a period of neo-adjuvant ADT therapy, as is generally standard in UK practice. For patients receiving docetaxel, this period needs to be a minimum of 6 months after randomisation to ensure that

[8] **Note:** this text has been transferred into the protocol from the Appendices in version 8.0, and updated

chemotherapy is completed and toxicity resolved before RT begins. To ensure consistency of timing of administration of standard radiotherapy in all arms, this same 6 months period is recommended for all patients. For patients with negative nodes on axial imaging, clinicians may choose between irradiating prostate and seminal vesicles alone or including the pelvic nodes in addition. Additional staging tests such as pelvic node sampling may be considered in making this decision. Conformal or intensity modulated radiotherapy should be used in all patients. Where patients have good clinical evidence that nodes are free of tumour or patients for whom nodal radiotherapy is contra-indicated (e.g. significant bowel disease), treatment may be given to the prostate gland and seminal vesicles only. The recommended dose is 74Gy in 37 fractions to the prostate and seminal vesicles or the equivalent using hypofractionated schedules. These recommendations are summarised in **Figure 10**. Alternative dosing schedules are permitted but must be agreed with the STAMPEDE Trial Management Group.

#### 6.15.1.A Standard-of-care RT Timing in M0 patients

Radiotherapy should be given around 6 to 9 months after randomisation in all trial arms and, if receiving docetaxel, the patient must have recovered from any docetaxel toxicity before RT can begin.

#### 6.15.1.B Type Of standard-of-care RT in M0 patients

Conformal or intensity modulated radiotherapy.

#### 6.15.1.C Standard Clinical Target Volume in M0 patients

- **CTV1:** Prostate plus seminal vesicles
- **CTV2:** (Node positive patients) Regional lymph nodes to include internal iliac and the inferior part of the common iliac nodes as used in EORTC trial 22961 (57)
- **PTV1:** CTV1 plus 10-15 mm according to local practice
- **PTV2:** CTV2 plus 10-20mm according to local practice

#### 6.15.1.D Standard-of-care RT Dose in M0 patients

Prostate dose of 74Gy in 2Gy fractions or equivalent, with optional dose to the pelvic nodes of 46-50Gy in 2Gy fractions or equivalent using IMRT to deliver the treatment over 37 fractions, suggested dose is 55Gy in 37 fractions with IMRT. Higher doses may be considered if the department is experienced in using IMRT for nodal radiotherapy, particularly as data emerges from the PIVOTAL trial of nodal IMRT in high-risk node negative patients where a nodal dose of 60Gy in 37 fractions is being evaluated. Alternative schedules should be agreed with the STAMPEDE Trial Management Group.

**Figure 10: Diagram for deciding recommended approach to standard-of-care (M0) RT in non-metastatic patients**

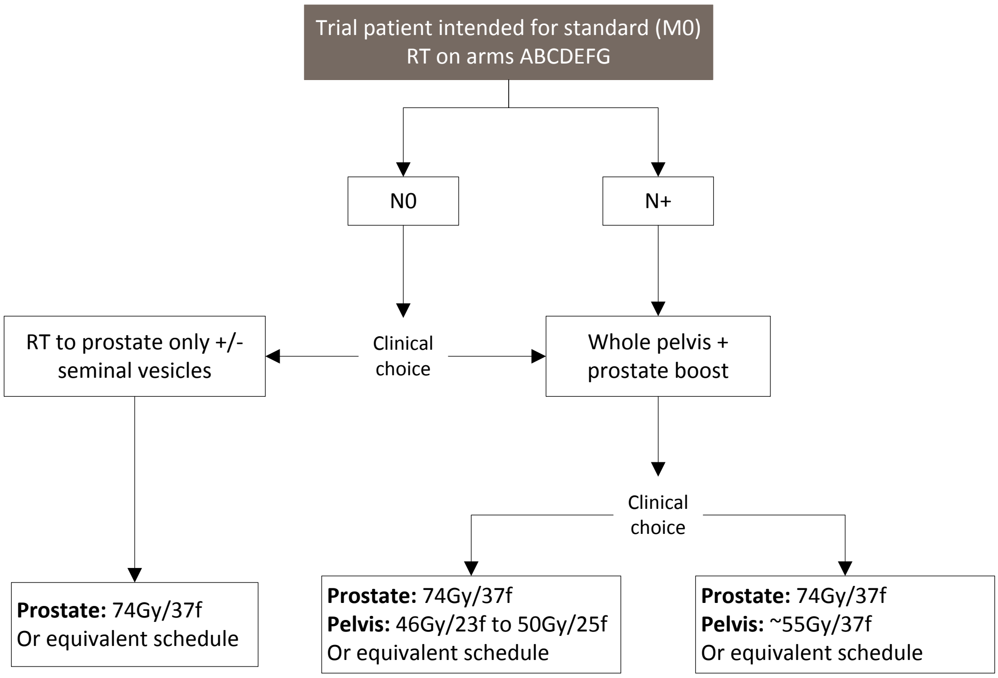


## 6.16 NON-TRIAL TREATMENT

### 6.16.1 MEDICATIONS PERMITTED

Any additional treatment that the responsible physician feels is appropriate is permitted.

### 6.16.2 DATA ON CONCOMITANT MEDICATION

All concomitant medication will be recorded on the baseline form prior to randomisation and on any subsequent Serious Adverse Event forms. This should include aspirin that may be taken on a regular basis for cardiovascular disease, the use of any Non-Steroidal Anti-inflammatory Drugs (NSAID) as well as any vitamin or mineral supplements the patient is taking.

# 7 ASSESSMENTS AND PROCEDURES

## 7.1 SCHEDULE FOR ASSESSMENTS

A detailed follow-up schedule is given in **Table 4, 5 and 6**.

### 7.1.1 PSA Measurements

All patients should have PSA measured pre-androgen deprivation therapy and at weeks 6, 12, 18 and 24 and every 12 weeks, thereafter, up to 2 years post randomisation. Following this, PSA should be measured every 6 months until 5 years and annually, thereafter. For patients who do not have a scheduled hospital visit, it would be acceptable for arrangements to be made for blood samples to be drawn in a GP's surgery.

### 7.1.2 Assessment Of Treatment Failure (Definition Of Progression)

It is not proposed to routinely assess patients for response. However, in order that objective progression can be assessed, it is necessary to have imaging taken at time of best response as judged by the treating clinician. All patients should have baseline radiological examinations as detailed in **Section 4.3.1**. In addition it is recommended that all patients should have scans or X-rays repeated at 24 weeks (and whenever clinically appropriate) if they were abnormal at baseline, particularly if they have a low PSA value on entry in to the trial making biochemical assessment of treatment failure difficult. The following events would constitute a disease progression and should be reported on a progression form:

- Biochemical failure – must be reported alongside castrate levels of testosterone if the patient has received intermittent ADT (see **Appendix J**).
- Local progression
- Lymph node progression
- Progression in distant metastases
- Development of new metastases

Please note that skeletal-related events (SREs) may be indicative of disease progression but can have other causes such as osteoporotic fracture. All SREs should be investigated further to establish whether or not the patient has progressed, in which case a progression form should be completed.

### 7.1.3 Additional Safety Assessment

Due to the risk of liver toxicity and secondary hyperaldosteronism with abiraterone, patients will require 2 weekly U+Es, LFTs and blood pressure measurement for the first 12 weeks. It is not proposed to collect the detail of these measurements unless results are abnormal; in this instance, they should be reported as AEs (on the next Follow-up CRFs) and as SAEs (see **Section 11**) if appropriate.

Medical review and PSA measurements follow the pattern in the control arm: visits at weeks 6, 12, 18 and 24 and every 12 weeks, thereafter, up to 2 years post randomisation. Following this, PSA should be measured every 6 months until 5 years and annually, thereafter. For patients who do not have a scheduled hospital visit, it would be acceptable for arrangements to be made for blood samples to be drawn either in a GP's surgery or in the patient's home.

#### 7.1.4 Data Collection and Non-Administration Of Standard Radiotherapy

There are CRFs to be completed for patients receiving primary radiotherapy whether this is standard radiotherapy for M0 patients on any arm or prostate radiotherapy for Arm H patients. All radiotherapy and acute side effects details will be recorded on the Radiotherapy Form; any late side effects will be recorded on the Follow up form.

If it is decided not to give the planned radiotherapy (for example, due to early metastatic progression or patient refusal), this should be stated on the Standard Radiotherapy form together with the reason for non-administration of the treatment.

#### 7.1.5 Data Collection Palliative Radiotherapy

For patients who receive palliative radiotherapy as part of first line treatment, a Palliative Radiotherapy CRF should be completed. Details of salvage RT for relapse and palliative treatment will be requested and completed only on the Progression Form.

#### 7.1.6 Data Collection Research (M1) Radiotherapy

There are arm specific CRFs for patients randomised to arm H. Adverse events such as hip fractures, TURPs, skeletal-related events will be collected retrospectively via the Hospital Episode Statistics (HES) database.

#### 7.1.7 Follow-Up Schedules

An individualised form with a follow-up schedule will be provided for each randomised patient. For patients who are receiving LHRH analogues, it is assumed that any additional treatment will commence within two weeks of randomisation. For patients who are due to have an orchidectomy it is recognised that surgery will have to be scheduled and the scheduling of any additional treatments may be affected by post-operative recovery. It is recommended that all patients who had abnormal radiological investigations at baseline or present with a low PSA on entry into the STAMPEDE trial should have them repeated 24 weeks after randomisation.

### 7.2 FOLLOW-UP

Every effort should be made to follow-up all patients who have been randomised. Patients should, if possible, remain under the care of an oncologist or urologist for the duration of the trial. If care of a patient is returned to the GP, it is the responsibility of the consultant who obtained the patient's consent to participate in the trial to ensure that the data collection forms are completed. If the patient moves from the local area, arrangements should be made for trial follow-up to be undertaken by their new local centre. Details of other participating centres can be obtained from the MRC CTU. The consent of patients should be obtained for their names to be flagged for survival information through national registries, for example NHS Information Centre/Office of National Statistics (ONS) in England/Wales and General Register Office in Scotland, Hospital Episode Statistics (HES). If the clinician moves, appropriate arrangements should be made to arrange for trial follow-up to continue at the centre.

**Table 4: Summary of timing of case report forms**

| CASE REPORT FORMS | TIMING OF ASSESSMENT AND CRF |
|---|---|
| **Baseline** | |
| Bone Density Risk Factor | At randomisation |
| Randomisation | At randomisation |
| Baseline | At randomisation |
| Cardiovascular Assessment | At randomisation |
| Pathology | At randomisation. When pathology sample has been taken and sent to UCL laboratory. |
| **Treatment** | |
| Pre-18 Week Bisphosphonate | Treatment administered every 3. Form holds data for 2 cycles. Form to be sent after 2nd cycle given. |
| Post-18 Week Bisphosphonate Treatment | Treatment administered every 4. Form holds data for 3 cycles. Form to be sent after 3rd cycle given. |
| Docetaxel Treatment | Treatment administered every 3 weeks Form holds 2 cycles. Form to be sent after 2nd cycle given. |
| Abiraterone and Enzalutamide Treatment | Treatments administered daily; form to be sent at each follow up visit |
| RT detail | • When standard-of-care radiotherapy is completed or if planned RT is no longer to be given<br>• Arm H when research RT completed<br>• Arm A (M1) at 3 months |
| RT Acute Toxicity | For all patients who receive primary RT. |
| **Assessments** | |
| Follow-Up | Every 6 weeks for 6 months, then every 12 weeks until 2 years. Every 6 months until 5 years and annually thereafter. (See Table 7 for more information.) |
| Palliative Radiotherapy | If applicable, when the palliative radiotherapy course is completed. |
| End of Treatment | When each treatment is completed (either at end of scheduled treatment or at early cessation of treatment). |
| Progression & Additional Treatment | At the first occurrence of each type of progression and whenever a patient that has progressed receives additional treatment. |
| Serious Adverse Event | Following any Serious Adverse Event |
| Skeletal-related Event | Whenever a patient experiences a skeletal-related event |
| Death | At Death |
| **Administration** | |
| Patient Transfer | When a patient is transferred to a different hospital for the administration of trial treatment and follow up |
| Co-enrolment | When a patient is co-enrolled in any other clinical trial. Please see Section 5.1 for more information |

**Table 5: Data required on follow-up forms**

| TIMING OF FOLLOW-UP | PSA | EVIDENCE OF PROGRESSION | ANDROGEN DEPRIVATION THERAPY | TREATMENT | UNSCHEDULED VISITS | TOXICITIES |
|---|---|---|---|---|---|---|
| Before progression | ✓ | ✓ | ✓ | ✓ | ✓ | ✓ |
| After Progression | - | ✓ | ✓ | ✓ | ✓ | ✓ |

## Table 6: Schedule for completion of treatment and outcome forms by arm.

| TIMING FROM RANDOMISATION | | | TREATMENT FORMS | | | OUTCOME FORMS | |
|---|---|---|---|---|---|---|---|
| YEARS | MONTHS | WEEKS | ZOL. ACID | ABI AND/OR ENZA | RT | FOLLOW-UP$^{\Psi}$ | QL + HE$^{¥}$ |
| **6-Weekly** | | | | | | | |
| - | - | 6 | B,E,F (†) | G, J | - | All arms | All arms |
| - | - | 12 | B,E,F (†) | G, J | M1: A,H | All arms | All arms |
| - | - | 18 | B,E,F (†) | G, J | - | All arms | All arms |
| - | - | 24 | B,E,F (‡) | G, J | - | All arms | All arms |
| **12-Weekly** | | | | | | | |
| - | - | 36 | B,E,F (‡) | G, J | - | All arms | All arms |
| - | - | 48 | B,E,F (‡) | G, J | M0: A,B,C,E,G, J | All arms | All arms |
| - | - | 60 | B,E,F (‡) | G, J | - | All arms | All arms |
| - | - | 72 | B,E,F (‡) | G, J | - | All arms | All arms |
| - | - | 84 | B,E,F (‡) | G, J | - | All arms | All arms |
| - | - | 86 | B,E,F (‡) | G, J | - | All arms | All arms |
| **6-Monthly** | | | | | | | |
| 2 | 24 | 104 | B,E,F (‡) | G, J | - | All arms | All arms |
| | 30 | 130 | - | G, J | - | All arms | All arms |
| 3 | 36 | 156 | - | G, J | - | All arms | All arms |
| | 42 | 182 | - | G, J | - | All arms | All arms |
| 4 | 48 | 208 | - | G, J | - | All arms | All arms |
| | 54 | 234 | - | G, J | - | All arms | All arms |
| 5 | 60 | 260 | - | G, J | - | All arms | All arms |
| **Annual** | | | | | | | |
| 6 | - | - | - | G, J | - | All arms | All arms |
| 7 | - | - | - | G, J | - | All arms | All arms |
| Etc | - | - | - | G, J | - | All arms | All arms |

**Key:**

A = ADT alone
B = ADT + zoledronic acid
C = ADT + docetaxel
D = ADT + celecoxib
E = ADT + zoledronic acid + docetaxel
F = ADT + zoledronic acid + celecoxib
G = ADT + abiraterone
H = ADT + M1 research RT to the prostate
J = ADT + enzalutamide + abiraterone

**Notes:**

Ψ See Table 6 for information required at follow-up
† Form records data for two cycles
‡ Form records data for three cycles
¥ 1st 700 patients and those recruited from protocol version 8.0 onwards only

**Note:** Radiotherapy, Late RT Toxicity, Palliative Radiotherapy Progression, SAE, End of Treatment, Co-enrolment and Death forms to be completed as required.

**Note:** Docetaxel forms are no longer shown on the table as all patients will have completed treatment with docetaxel

**Note**: recruitment completed to Arms D and F in April 2011; Arms B, C and E in March 2013; Arm G in January 2014

**Note**: Quality of Life Study is only for first 700 patients entered into the trial and those who were recruited after the implementation of version 8.0 of the protocol. MRC CTU will inform centres of which of their patients this applies to.

## 7.3 TRIAL CLOSURE

For the purpose of complying with UK Clinical Regulations introduced on May 2004, the trial will be considered ‘closed’ when the follow-up point for the primary analysis of the final comparison has been reached. However, further observational follow-up of all patients enrolled in the trial will continue until all randomised patients have died. This will initially be via the hospital, but in the longer term may employ national registers.

# 8 STOPPING OF TREATMENT OR FOLLOW UP

Patients should be given every encouragement to adhere to protocol treatment and follow-up schedule, in order to reduce bias. However, a patient has the right to withdraw consent for participation in any aspect of this trial at any time.

## 8.1 STOPPING RESEARCH INTERVENTIONS

A patient may stop trial treatment for the following reasons:

- Progression whilst on therapy (trial treatment must be discontinued in this instance). For patients randomised to Arm G, please refer to **Section 6.8** for criteria to stop treatment
- Unacceptable toxicity
- Intercurrent illness which prevents further treatment
- Withdrawal of consent for treatment
- Any alteration in the patient's condition which justifies the discontinuation of treatment in the clinician's opinion
- Intention to commence a new anti-cancer treatment due to evidence of relapse.

The reason should be recorded on the treatment and/or follow-up forms as well as the End of Treatment form. In the case of abiraterone, the disease event for stopping abiraterone may be after the first reportable failure-free survival event (see **Section 6.8**). Unless a patient states otherwise, it should be assumed that consent is given to continue to record trial data.

## 8.2 PATIENT TRANSFERS

For patients moving from the area, every effort should be made for the patient to be followed-up at another participating trial centre and for this trial centre to take over responsibility for the patient. To document the transfer process the main contact person at both the current and receiving hospitals should complete and sign the Patient Transfer Confirmation form. A fully completed form must be returned to the CTU prior to the patient transfer and ideally any data queries for the patient should be completed prior to transfer.

On receipt of the completed transfer form a member of the STAMPEDE team will confirm the database has been updated and request confirmation of the name of the patient's new Clinician. Photocopies of the following documents may then be sent to the new hospital to complete the transfer and copies must be also retained at the original site for monitoring purposes:

- Consent form
- Completed CRFs
- Any documentation relating to the patient's participation in STAMPEDE (patient names must be removed from any documentation).

## 8.3 WITHDRAWAL FROM THE TRIAL COMPLETELY

If a patient explicitly withdraws consent to have any data recorded their decision must be respected and the MRC CTU must be informed in writing. All communication surrounding the withdrawal

should be noted in the patient's records and no further STAMPEDE CRFs should be completed for that patient.

Early stopping of follow-up should not be undertaken lightly and the site must consider the implications for the trial and the patient in reaching such a decision.

Patients can change their minds about withdrawal at any time and re-consent to participate in the trial. Follow-up data should be collected only from the point of when consent was re-instated.

# 9 STATISTICAL CONSIDERATIONS

## 9.1 METHOD OF RANDOMISATION

Patients will be randomised centrally using a computerised algorithm developed and maintained by the MRC CTU. Randomisation will be performed using the method of minimisation over a number of clinically important stratification factors with an additional random element. To decrease determinability, the factors are not listed here but can be found in the Statistical Design Document.

Table 8 shows the allocation weighting for each arm by protocol version. The relative weighting within each pairwise comparison remains constant throughout.

### 9.1.1 To Version 7

From the outset, the trial had 1 control arm (A) and 5 research arms (B, C, D, E and F).

As the control arm is the comparator arm for all the research arms, twice as many patients were recruited to the control arm as to each of the original research arms as this is an efficient design. Therefore, the initial randomisation ratio will be A2:B1:C1:D1:E1:F1. From version 7.0, accrual to the celecoxib-containing arms was halted and the allocation ratio was A2:B1:C1:D0:E1:F0.

### 9.1.2 Version 8

From version 8.0, an additional research arm (G) was introduced. The allocation weighting for the additional Arm G is 2, meaning that as many patients are contemporaneously randomised to Arm G as the control Arm A: the randomisation ratio is 2:2 (equivalent to 1:1 control:abiraterone). This gave an overall allocation ratio of A2:B1:C1:D0:E1:F0:G2. When recruitment has been completed to the ongoing original research Arms B, C and E (which will be around 2 years before completion of accrual to arm G), the allocation ratio will be A2:B0:C0:E0:D0:F0:G2 (or A2:G2). This is more efficient for this comparison than the 2:1 allocation ratio employed for the original research arms because of the minimal co-recruitment period.

Version 9.0 introduced a RT comparison for men with newly-diagnosed metastatic disease which is irrelevant to a subset of men joining STAMPEDE. This could only be achieved by splitting the randomisation system so that newly-diagnosed patients with M1 disease and no contraindication to RT are randomised A2:B1:C1:D0:E1:F0:G2:H2 and other men are randomised A2:B1:C1:D0:E1:F0:G2:H0. Note that the allocation ratio for each pairwise comparison in unaffected, only the rate at which comparisons accrue.

### 9.1.3 Version 10 and 11

Version 10.0 followed the successful completion of recruitment to Arms B, C and E. Therefore, the allocation ratio will be A2:B0:C0:E0:D0:F0:G2 (or A2:G2) for M0 patients and A2:B0:C0:E0:D0:F0:G2:H2 for M1 radiotherapy arm patients (2A:2G:2H).The equal allocation ratio is suitable with fewer research arms open.

### 9.1.4 Version 12

Version 12.0 introduces a further allocation, Arm J: HT + abiraterone + enzalutamide. This allocation will be available to all patients. Accounting for Arm H still recruiting, this can only be achieved by keeping the randomisation system split so that newly-diagnosed patients with M1 disease and no contraindication to RT will be randomised A2:B0:C0:D0:E0:F0:G0:H2:J2 and other men will be

randomised A2:B0:C0:D0:E0:F0:G0:H0:J2. This can be simplified to equal allocation in these groups to A: H:J and A:J.

**Table 7: Allocation to each arm by protocol version**

| PROTOCOL | NEWLY-DIAGNOSED M1 PATIENTS | | | | | | | | | OTHER PATIENTS | | | | | | | | |
|---|---|---|---|---|---|---|---|---|---|---|---|---|---|---|---|---|---|---|
| VERSION | A | B | C | D | E | F | G | H | J | A | B | C | D | E | F | G | H | J |
| V1 | 2 | 1 | 1 | 1 | 1 | 1 | - | - | - | 2 | 1 | 1 | 1 | 1 | 1 | - | - | - |
| V2 | 2 | 1 | 1 | 1 | 1 | 1 | - | - | - | 2 | 1 | 1 | 1 | 1 | 1 | - | - | - |
| V3 | 2 | 1 | 1 | 1 | 1 | 1 | - | - | - | 2 | 1 | 1 | 1 | 1 | 1 | - | - | - |
| V4 | 2 | 1 | 1 | 1 | 1 | 1 | - | - | - | 2 | 1 | 1 | 1 | 1 | 1 | - | - | - |
| V5 | 2 | 1 | 1 | 1 | 1 | 1 | - | - | - | 2 | 1 | 1 | 1 | 1 | 1 | - | - | - |
| V6 | 2 | 1 | 1 | 1 | 1 | 1 | - | - | - | 2 | 1 | 1 | 1 | 1 | 1 | - | - | - |
| V7 | 2 | 1 | 1 | 0 | 1 | 0 | - | - | - | 2 | 1 | 1 | 0 | 1 | 0 | - | - | - |
| V8 | 2 | 1 | 1 | 0 | 1 | 0 | 2 | - | - | 2 | 1 | 1 | 0 | 1 | 0 | 2 | - | - |
| V9 | 2 | 1 | 1 | 0 | 1 | 0 | 2 | 2 | - | 2 | 1 | 1 | 0 | 1 | 0 | 2 | 2 | - |
| V10 | 2 | 0 | 0 | 0 | 0 | 0 | 2 | 2 | - | 2 | 0 | 0 | 0 | 0 | 0 | 2 | 2 | - |
| V11 | 2 | 0 | 0 | 0 | 0 | 0 | 2 | 2 | - | 2 | 0 | 0 | 0 | 0 | 0 | 2 | 2 | - |
| V12 | 2 | 0 | 0 | 0 | 0 | 0 | 0 | 2 | 2 | 2 | 0 | 0 | 0 | 0 | 0 | 0 | 0 | 2 |

## 9.2 OUTCOME MEASURES

The overall, definitive primary outcome measure for the trial for each comparison is overall survival (all-cause mortality). The design of the trial is such that it is important to have additional intermediate outcome measures to assess each research arm as the trial progresses. These are listed in **Table 9**. The intermediate primary outcome measure is failure-free survival. The reasons for different emphases in each recruitment stage are explained in **Section 9.3**.

**Table 8: Trial Outcome Measures by Comparison Stage**

| TRIALS STAGE | PRIMARY OUTCOME MEASURES | SECONDARY OUTCOME MEASURES |
|---|---|---|
| Pilot phase | Safety* | Feasibility |
| Activity Stage (AS) I-III | Failure-free survival (FFS)† | Overall survival (OS)<br><br>Toxicity<br>Skeletal-related events |
| Efficacy Stage (ES) IV | Overall survival | Quality of life<br><br>Cost effectiveness<br>Failure-free survival†<br>Toxicity<br>Skeletal-related events |

*Based on toxicity
†Including biochemical failure (see **Appendix J**)

## 9.3 SAMPLE SIZE: PRINCIPLES AND ASSUMPTIONS

The overall design for this study is a multi-arm multi-stage, multi-centre randomised controlled trial. There are a number of stages for each research arm: a Pilot Phase, Activity Stages and a final Efficacy Stage. Full details of the methodology underlying the trial design are given by Royston et al. (58, 59) The sample size calculations were performed using the `stage2` (version 1.2.0, March 2002) and `stagen` (version 1.1.1, 18 May 2004) programs, both implemented in Stata (Stata Corp, TX) and updated using the later `nstage` program (version 1.0.3, 13-jun-2007; version 2.1.0, 28-jun-2009). (60)

The trial was designed under the assumptions in **Table 10**, and additionally, we assume a slightly higher proportion of non-metastatic than metastatic patients joining the trial such that the median FFS is two years and median OS four years for the whole cohort.

**Table 9: Hazard ratio assumptions under null and alternative hypotheses**

| SIZE OF HR | PILOT | AS I-III | ES IV |
|---|---|---|---|
| Under null hypothesis (H0) | n/a | HR(FFS) = 1.00 | HR(OS) = 1.00 |
| Under alternative hypothesis (H1) | n/a | HR(FFS) = 0.75 | HR(OS) = 0.75 |

The HR of 0.75 for any research arm relative to control would translate into an absolute improvement in FFS of 10%, from approximately 50% to 60% at two years and OS of 10%, from approximately 50% to 60% at four years. A beneficial difference of this size would be clinically worthwhile and, indeed, experience tells us it may be unrealistic to expect a larger difference. Therefore, we have adequately powered the trial to detect a HR of 0.75 for overall survival. This design gives 95% power at Activity Stages I-III and 90% power at Efficacy Stage IV for each comparison. Further details of the sample size calculations are summarised in **Sections 9.4** and **9.5** and detailed in a separate Statistical Design Documents which are available on request.

Note that, from version 8.0, standard-of-care M0 RT was introduced to the majority of patients with N0 M0 disease. This is likely to improve the outcomes for this group. Further agents are starting to be licensed for patients with castration-refractory disease which may also improve survival rates. Improved FFS rates would delay the intermediate analyses; improved survival rates would delay the definitive analyses. The Statistical Design Document includes models where median survival is estimated at 5, 6 and 7 years rather than just 3and 4 years. The trial is powered to detect a difference in relative improvement and the analyses will be performed when a pre-planned number of events has been reported in the control arm, rather than after a certain number of patients have been recruited or a certain amount of time elapsed. **Sections 9.4** and **9.5** provide more detail, including some variations on these assumptions.

Throughout recruitment to version 12.0, at least, the proportion of metastatic men joining the trial has been fairly constant, at around 60%. From version 9.0, we introduced an allocation, Arm H, only for men with M1 disease. This means that further comparisons for the whole patient group will have proportionately fewer metastatic patients and, therefore, fewer events at any given moment in time. This will affect contemporaneously-recruiting comparisons, such as the “enzalutamide + abiraterone comparison” introduced in the current version 12.0. Median survival may therefore be higher in that comparison, at around 7 years.

## 9.4 SAMPLE SIZE ISSUES AND TRIAL STAGES: ORIGINAL RESEARCH ARMS (B-F)

### 9.4.1 PILOT PHASE: ORIGINAL RESEARCH ARMS (B-F)

It was anticipated that 210 patients would be recruited to the Pilot Phase from a limited number of centres over a one year period. Approximately 60 patients would be randomised to the control arm and 30 patients to each of the five research arms, each of which were assessed for safety and feasibility. If recruitment proved unfeasible or any of the research arms proved unsafe or not feasible to administer (e.g., poorly tolerated or unexpected toxicity) recruitment to these arms would have been discontinued. There were already considerable safety data on the use of docetaxel and zoledronic acid in patients with malignancies including prostate cancer, and on the use of Cox-2 inhibitors (including celecoxib), although mainly from patients with musculoskeletal disorders. There were fewer data on the combination arms, but it was thought very unlikely that any of the research arms would be discontinued during the Pilot Phase. When 210 patients had been on the trial for a minimum of 18 weeks, the Independent Data Monitoring Committee (IDMC) reviewed the data from the Pilot Phase and continued to the trial during this period as equipoise remained. Recruitment continued beyond this point. Safety data are assessed throughout the trial.

### 9.4.2 ACTIVITY STAGES I-III: ORIGINAL RESEARCH ARMS (B-F)

In the sample size calculations, we assumed that all research arms successfully pass through the Pilot Phase to Activity Stage I and that patients would be recruited at a rate of approximately 500 per year. This was faster than in the Pilot Phase because the trial would recruit from additional centres, both in the UK and internationally. The analysis of Activity Stages I, II and III were planned for when around 113, 216 and 334 failure-free survival events had been observed in the control arm, respectively.

The Activity Stage analyses comprise pairwise comparisons of FFS between the control arm and each of the 5 research arms (i=B, C, D, E, F). Let $HR_i$(true) represent the hazard ratio (HR) of the $i^{th}$ research arm to the control arm, and HRi(observed) the observed value. Discontinuation of accrual

of further patients was considered for the $i^{th}$ research regimen at each of Activity Stages I-III according to the guidelines in **Table 11**.

**Table 10: Guidelines for stopping accrual to the ith original research arm**

| Activity Stage | Number of control arm events | Consider discontinuation if $HR_i$(observed) is... |
|---|---|---|
| I | ~113 | >1.00 |
| II | ~216 | >0.92 |
| III | ~334 | >0.89 |

### 9.4.3 Efficacy Stage IV: Original Research Arms (B-F)

The analysis of Efficacy Stage IV for the original research arms will be performed when around 403 deaths have been observed in the control arm. This would give 90% power to detect the targeted hazard ratio of 0.75 at one-sided significance level of 0.025. The actual length of this stage, balancing continued accrual with just follow-up, depended on the number of arms passing through to further recruitment from Activity Stages I-III and the observed accrual and event rates.

### 9.4.4 Sample Size For Original Research Arms (B-F)

Assuming an accrual rate of 500 patients/year, between 2800 and 3600 patients were planned to be entered into the original research comparisons of the trial over a period of 5½ and 7 years. The exact number of patients to be entered depends on the observed accrual rate and the observed event rate, which is, in itself, dependent on the mix of patients joining the trial from the broad spectrum of eligibility. The primary analysis on overall survival requires around 403 deaths to be observed on the control arm. Accrual continued until the main analysis can be foreseen so that the overall duration of the comparisons would be as short as possible (longer accrual facilitates this) and so that few, if any, patients remain on treatment when the main results are released. The statistical team have monitored and projected the analysis timelines using the `artpep` command in Stata. Results should be due in 2015. Further information is available in the Statistical Master File.

## 9.5 SAMPLE SIZE ISSUES AND TRIAL STAGES: ADDITIONAL RESEARCH ARM G

### 9.5.1 Pilot Phase: Additional Research Arm G

A similar approach is being followed for the additional research Arm G, as detailed for the original research arms in **Section 9.4.1**. The IDMC reviewed safety data, in the context of data from the control arm, when the first 30 patients allocated to Arm G had been on trial for at least 18 weeks.

Furthermore, an additional review of safety was performed when 30 patients with newly-diagnosed non-metastatic disease allocated to Arm G had been on trial for at least 18 weeks.

Both of these milestones were successfully completed.

### 9.5.2 Activity Stages I-III: Additional Research Arm G

The same principles are applied to the new comparison as to the previous comparisons. The notable difference will be in the accrual rate to this comparison which is anticipated to be higher. There are two reasons for this. First, STAMPEDE started to recruit slowly in only a limited number of pilot sites. As more sites have been activated, including internationally, accrual has increased. At the time of

version 8.0 of the protocol, monthly accrual to the study was averaging around 60 patients/month (over 700 patients/year). Second, there is an equal allocation ratio for the abiraterone arm compared to the control arm. It is this different allocation ratio which means that the number of control arm events required to trigger the intermediate analyses is different for the assessment of abiraterone to the assessment of the original research arms. This is shown in **Table 11**.

**Table 11: Guidelines for stopping accrual to the additional research Arm G**

| ACTIVITY STAGE | NUMBER OF CONTROL ARM EVENTS | CONSIDER DISCONTINUATION IF $HR_G$(OBSERVED) IS… |
|---|---|---|
| I | ~75 | >1.00 |
| II | ~142 | >0.92 |
| III | ~221 | >0.89 |

### 9.5.3 EFFICACY STAGE IV: ADDITIONAL RESEARCH ARM G

The analysis of Efficacy Stage IV for the additional comparison will be performed when around 267 deaths have been observed in the control arm. This would give 90% power to detect the targeted hazard ratio of 0.75 at a one-sided significance level of 0.025.

### 9.5.4 SAMPLE SIZE FOR ADDITIONAL RESEARCH ARM G

Up to around 1,800 patients will join the abiraterone comparison, with half allocated to the research arm. Consideration will be given to ceasing further randomisations to Arm G if it is not showing sufficient evidence of activity at the interim analyses, just as was done for research Arms B to F.

The original plan intended for accrual to be halted either when 1,500 patients had been recruited or after 3 years, whichever was the sooner, providing the accrual rate remained above 50 patients/months.

The total number of patients joining this comparison depends not just on the same issues as the original comparisons (notably, observed accrual and event rates), but also the length of time that the original research arms co-recruit alongside the additional research arm; it was originally assumed that this would be for approximately 1 year, but it was closer to 1.5 years. The sample size calculations and projected durations are fairly robust to changes in the length of co-recruitment with the original research arms and future co-recruitment with any further research arms which the Trial Management Group may introduce. Many scenarios are detailed in the Statistical Design Document.

In Sep-2013, the target sample size for the abiraterone comparison was increased from around 1,500 patients to around 1,800 patients, with the efficacy analysis still to be triggered by 267 control arm deaths. This increase in sample size was primarily because of an increase in the proportion of non-metastatic patients joining the comparison; this related to the activation of Arm H which only recruits patients with newly-diagnosed metastatic disease and thereby reduces the numbers of metastatic patients randomised to the abiraterone comparison. Non-metastatic patients have a lower event rate than the metastatic patients and maintaining the same overall sample size would lead to a delay in time to the primary analysis. The increase in sample size was achievable because recruitment rates to the trial had been substantially higher than 50 patients/month for the preceding 6 months.

## 9.6 SAMPLE SIZE ISSUES AND TRIAL STAGES: ADDITIONAL RESEARCH ARM H

### 9.6.1 Pilot Phase: Additional Research Arm H

A similar approach will be followed for the additional research Arm H as detailed for the original research arms in **Section 9.4.1**. The IDMC will review safety data, in the context of data from the control arm, when the first 30 patients allocated to arm H have been on trial for around six months.

### 9.6.2 Activity Stages I-III: Additional Research Arm H

The same principles will be applied to the new comparison as to the previous comparisons and an equal allocation ratio of control arm patients to patients allocated to Arm H will be employed, as for Arm G. The number of control arm events required to trigger the intermediate analyses will be the same as for the abiraterone comparison (see **Table 13**).

### 9.6.3 Efficacy Stage IV: Additional Research Arm H

The analysis of Efficacy Stage IV for the additional comparison will be performed when around 267 deaths have been observed in the control arm. This would give 90% power to detect the targeted hazard ratio of 0.75 at one-sided significance level of 0.025.

### 9.6.4 Sample Size For Additional Research Arm H

Consideration will be given to ceasing further randomisations to Arm H if it is not showing sufficient evidence of activity on the intermediate primary outcome measure (FFS), just as for the other research arms. This research comparison is relevant to around 60% of patients joining STAMPEDE. At the point of the scientific approval, accrual was averaging around 80 patients per month to the trial. If accrual to the trial was slower at 70 patients per month, then accrual to this comparison could be between 18 and 42 patients per month, depending on which other trial arms are open to recruitment at the time.

We are targeting a 25% relative improvement in overall survival following local radiotherapy to the prostate in this patient group. This is the same size of effect targeted with the other research arms in STAMPEDE. This relative improvement can be further justified in the light of MRC PR07 which demonstrated an improvement of this magnitude for adding radiotherapy to ADT in locally advanced disease, with a hazard ratio for overall survival of 0.77 (95% CI 0.61 to 0.98). In that trial, fewer than half of the deaths were from prostate cancer, whereas in newly-diagnosed metastatic patients nearly all men will die of their disease. Therefore, it is relevant to note the relative benefit of radiotherapy in PR07 in terms of prostate cancer-specific survival, where the hazard ratio was 0.54 (95% CI 0.27 to 0.78). Long-term survival-based data, with a median follow-up of ~10 years, were presented orally at the American Society of Clinical Oncology 2012 which confirmed these findings.(7)

We anticipate that around 1250 patients are required over 4 years to observe 267 control arm deaths after 5.25 years. In addition to the factors listed in **Section 2.1.2**, this assumes that (i) recruitment is constantly 70 pts/m to the trial overall, (ii) the original research arms stop accrual within 6 months after activation of the RT arm, (iii) the abiraterone arm stops accrual around 24 months after activation of the RT arm, and (iv) a further new research arm with an equal allocation ratio is introduced 18 months after activation of the RT arm.

With variations on these factors, between 1000 and 1400 patients are required over 2.75 to 4.50 years to address survival within 4.50 to 6.50 years. These sample scenarios will be documented in the Trial Master File.

All patients joining the trial will be starting long-term ADT for the first time. The focus of this comparison will be on the newly-diagnosed, metastatic patients (and no contraindications to RT), which is the largest subgroup of patients in the trial and the group of patients at highest risk of death from prostate cancer. Patients with non-metastatic disease will be excluded from this particular comparison as there are already randomised data demonstrating the survival benefit from radiotherapy in patients with locally advanced disease. Radiotherapy is now mandatory in node negative patients; it is also recommended in the node-positive, non-metastatic (N+ M0) group.

For the control arm of the whole trial, we constructed sample size scenarios median failure-free survival being 18, 24 or 30 months and constructed sample size scenarios around each of these options; the event rate would depend on the patient mix. We now know that around 60% of patients have M1 disease at trial entry and we have reported that FFS at 24 months is 51% across the whole of the control arm.(1)

For the sample size calculation for this new planned comparison, we have based our estimates on the subgroup of patients with newly-diagnosed M1 disease in the control arm. Therefore, we estimate median FFS to be 1 year and estimate that median overall survival will be 3.5 years.

## 9.7 SAMPLE SIZE ISSUES AND TRIAL STAGES: ADDITIONAL RESEARCH ARM J

### 9.7.1 Pilot Phase: Additional Research Arm J

A similar approach will be followed for the additional research Arm J as detailed for the original research arms in **Section 9.4.1**. The IDMC will first review safety data for this combination when the first 50 patients allocated to Arm J have been on trial around 6 weeks (i.e. to the first follow-up visit).

The IDMC will review safety data again when 50 patients are 6 months out from randomisation. Additional safety reviews will be performed if the IDMC raises any concerns over safety and routinely reviewed at regular intervals.

Direct comparison will be available with contemporaneously randomised patients on Arm A (hormones alone). Contextual data will be provided from Arm G (hormones plus abiraterone. Indicative safety data may also be available on the combination from other studies in CRPC.

### 9.7.2 Activity Stages I-II: Additional Research Arm J

The principles of intermediate analyses will be applied to this new comparison, but some of the details will be different. Owing to the expected accrual rate (>100 pts/m) and the expected slower event rate, only two activity stages are planned before accrual is completed. These are set out in **Table 11**.

### 9.7.3 Efficacy Stage III: Additional Research Arm J

The analysis of Efficacy Stage III for the additional comparison will be performed when around 267 deaths have been observed in the control arm. This would give 90% power to detect the targeted hazard ratio of 0.75 at a one-sided significance level of 0.025.

**Table 12: Guidelines for stopping accrual to the additional research Arm J**

| Stage | Sig Level | Power | Targeted HR | Number of control arm events | Consider discontinuation if $HR_J$(observed) is… |
|---|---|---|---|---|---|
| I – Activity | 0.40 | 95% | 0.70 | ~66 | >0.957 |
| II – Activity | 0.12 | 95% | 0.70 | ~139 | >0.869 |
| III – Efficacy | 0.025 | 90% | 0.75 | ~267 | >0.845 |

### 9.7.4 Sample Size For Additional Research Arm J

Consideration will be given to ceasing further randomisations to Arm J if it is not showing sufficient evidence of activity on the intermediate primary outcome measure (FFS), just as for the other research arms.

The patient mix for this comparison is likely to represent a more favourable prognosis in average than in the original research trial's other arms, due to concurrent recruitment of M1 but not M0 patients, to Arm H.

We anticipate that up to about 1800 patients are required within 3.5 years to observe ~267 control arm deaths within 6 years. This time will be dependent on the observed overall survival. The default scenario assumes that (i) recruitment is constantly 70pts/m to the trial overall, (ii) the M1/RT arm accrues throughout and (iii) a further new research arm with an equal allocation ratio is introduced 18 months after activation of Arm J.The stopping date for Arm G is no longer an assumption.

With variations on these factors (documented in a Statistical Design Document), 1800 patients are required over 2.5 to 3.5 years to address survival within 6 years. If accrual rates to the trial are at 150pts/m (as observed during Summer 2013), accrual of 1,800 patients to the comparison could be achieved within 2 years. These sample scenarios will be documented in the Trial Master File.

### 9.7.5 Further Sample Size Issues For Additional Research Arm J

Careful consideration will be given to the emerging data from the abiraterone comparison (Arms G vs A) and whether this arm continues to recruit throughout. It is anticipated that recruitment to this Arm J comparison will be completed *before* survival data emerge from the abiraterone comparison.

Indirect comparisons to understand the contribution from each agent may be possible if this research arm is demonstrably superior to the standard-of-care. These plans will be developed and documented elsewhere, but a higher number of patients will help with the power to the indirect comparison.

**Figure 11: Schema of progress of STAMPEDE through the trial**

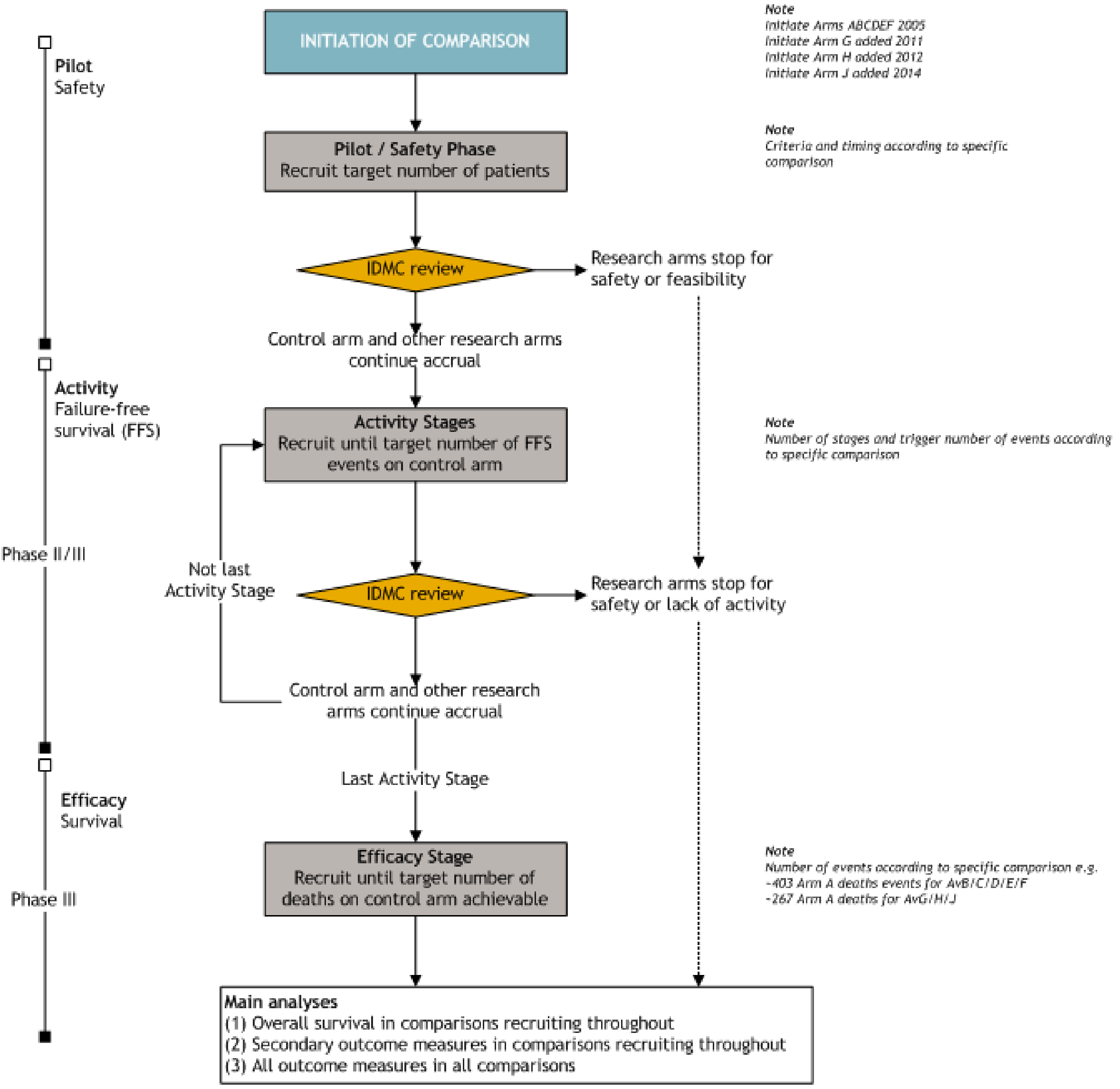


**Key**
**FFS:** Failure-free survival
**HR:** Hazard ratio
**IDMC:** Independent Data Monitoring Committee
**Pts:** Patients

**Notes**
Exact accrual depends on many factors including accrual rate, event rate and arms recruiting in each stage

## 9.8 FURTHER NOTES ON TRIAL DESIGN

### 9.8.1 OVERALL SAMPLE SIZE

Given the adaptive nature of the study, there is no formal overall sample size target, but the numbers of patients required for each comparison are detailed in **Sections 9.4** to 9.7. It is expected that more than 7,000 patients will likely be recruited overall.

### 9.8.2 FACTORIAL DESIGN

We note here that we have not employed a factorial design in this trial because we anticipate the possibility of synergy between ADT, zoledronic acid and docetaxel and between ADT, zoledronic acid

and celecoxib. It would not be possible to assess any such interactions reliably in a factorial trial (see the Statistical Design Document for further details).

## 9.9 INTERIM MONITORING AND ANALYSES

The accumulating data will be reviewed at regular intervals (approximately annually) by an Independent Data Monitoring Committee (IDMC), including pre-specified formal intermediate analyses of activity data (see also **Section 16**). These analyses will be performed by the trial team at the MRC CTU. Only patients randomised contemporaneously will be included in the comparison of each research arm against control e.g. patients allocated to the control arm prior to version 12.0 will not contribute to the "enzalutamide + abiraterone comparison" (Arm A vs Arm J).

The IDMC will be asked to give advice on whether the accumulating data from the trial with the guidelines for discontinuation of accrual for the relevant Activity Stages, together with results from any other relevant trials, justifies continuing recruitment of further patients or further follow-up. A decision to discontinue recruitment, in all patients or in selected subgroups will be made only if the result is likely to convince a broad range of clinicians including those entering patients into the trial and the general clinical community. The intermediate stopping guidelines apply to the intermediate primary outcome measure. To stop accrual early for benefit in any comparison would require convincing data in terms of the definitive primary outcome measure, overall survival. For example, this could be $p<0.001$ as proposed by Haybittle-Peto.(61, 62) The use of such a guideline for stopping for benefit has a minimal impact on the operating characteristics.

If a decision is made to continue without change, the IDMC will advise on the frequency of future reviews of the data on the basis of accrual and event rates. The IDMC will make recommendations to the Trial Steering Committee (TSC, see **Section 16**) as to whether the trial should continue in its present form. While the trial is ongoing the accumulating data will generally remain confidential, unless the TSC and IDMC agree that the data should be made public.

## 9.10 OUTLINE ANALYSIS PLAN

Analyses will be performed on an intention-to-treat basis. The standard unadjusted log-rank approach will be applied to analyses of FFS and OS. The impact of potential confounders including the stratification factors used at randomisation will be considered in a Cox proportional hazard model. Flexible parametric models will be used to calculate the absolute differences between the arms to show treatment differences over time and to estimate restricted mean "survival" times (RMST). The estimated difference in restricted means survival time will be used preferentially to compare treatment arms if the proportional hazards assumptions required for hazard ratios cannot be supported. The $\chi^2$ test or Mann-Whitney test will be implemented for categorical data comparisons, including toxicity, as appropriate. The primary outcome measures (see **Section 9.2**) will be considered for all arms of the trial at each phase, but the main emphasis will be placed on the comparison of the research arms that have continued to recruit throughout the trial.

### 9.10.1 PILOT / SAFETY PHASES

The Pilot Phase randomised patients between all the trial arms so that the results from these patients can be included in the main trial. Feasibility is considered in terms of the acceptability of the trial randomisation and reported toxicities and adherence to trial medication. Centres participating in the Pilot Phase for the original research arms were required to keep an anonymised log of all

patients assessed for trial eligibility (see protocol version 2.0) so that the number of patients who did not participate in the study and the number of eligible patients who choose to not participate in the study could be summarised (reasons for non-participation were collected where the patients was willing). The anonymised logs will not be needed for new research arms after v 8.0.

For the patients who are randomised, we shall describe the incidence of expected and unexpected severe toxicities and adverse events/reactions (see **Section 11**) to decide whether to continue with research arms beyond the Pilot Phase. As indicated above, we do not anticipate that recruitment to the research arms will be discontinued after the Pilot Phase, as there is considerable experience with zoledronic acid and docetaxel when combined with ADT, while Cox-2 inhibitors generally have a good toxicity profile. Although there are limited data on the combinations, we do not expect severe toxicity.

### 9.10.2 Activity And Efficacy Stages

The approach to analysis of these stages is summarised within the sample size calculations (see earlier subsections of Se**ction 9.4.3**). Each research arm will be compared in a pairwise fashion against the control arm.

Full details are available in the Statistical Analysis Plan.

# 10 MONITORING AND QUALITY ASSURANCE

## 10.1 MONITORING AT MRC CTU

Data provided to the MRC CTU will be checked for missing or unusual values (range checks) and consistency over time. If missing or questionable data are identified, staff at the MRC CTU will request that the data be clarified. The exact procedures for data clarification and the amendment of CRFs will be described in the trial specific SOPs and instructions will be sent to all STAMPEDE institutions as soon as they have been approved to participate in the trial. The MRC CTU will also send reminders for any overdue data.

## 10.2 DIRECT ACCESS TO DATA

Collaborating institutions should be aware that direct access to patient data by MRC CTU staff may be required for trial-related monitoring or audit. Patient consent for this will be obtained as part of the general trial consent process.

## 10.3 VISITS TO INVESTIGATOR SITES

A selection of institutions will be visited at least once during the course of the STAMPEDE trial. The MRC CTU will give the responsible investigator adequate notice of the monitoring visit to allow adequate time, space and staff for these visits. The standard operating procedures (SOP) for monitoring are available from the MRC CTU.

After the monitoring visit the monitor will complete a site visit report. This report will be circulated to the TMG for comment. Once the TMT have reviewed the report and agreed on any recommendations the monitor will finalise the report and send a copy to the Principal Investigator (PI) at the site. A copy will also be sent to the CI and TMG for the trial and another copy will be kept in the MRC CTU STAMPEDE trial master file.

## 10.4 CONFIDENTIALITY

All information collected during the course of the research will be kept strictly confidential. In addition, all procedures for handling, processing, storage and destruction of data are compliant with the Data Protection Act 1998. No individual patients will be identified when the results of the trial are published.

Patients will be asked for permission for information about their health status to be obtained from the Office of National Statistics (ONS) or via the NHS Strategic Tracing Service or similar by the Medical Research Council, if necessary. In addition, patients will be asked for permission to inform their GP of their involvement in the STAMPEDE trial.

# 11 SAFETY REPORTING

ICH GCP requires that both investigators and sponsors follow specific procedures when reporting adverse events/reactions in clinical trials. These procedures are described in this section of the protocol. Further information on the expected toxicities for the trial interventions (docetaxel, zoledronic acid, abiraterone and radiotherapy) can be found in **Appendix G**.

## 11.1 DEFINITIONS

The safety reporting definitions from ICH GCP apply in this trial protocol. These definitions are given in **Table 14**.

**Table 13: Event Terms and Definitions**

| TERM | DEFINITION |
|---|---|
| Adverse Event (**AE**) | Any untoward medical occurrence in a patient or clinical trial subject to whom a medicinal product has been administered including occurrences which are not necessarily caused by or related to that product. |
| Adverse Reaction (**AR**) | Any untoward and unintended response to an investigational medicinal product related to any dose administered. |
| Unexpected Adverse Reaction (**UAR**) | An adverse reaction, the nature or severity of which is not consistent with the information about the medicinal product in question set out in the summary of product characteristics (or Investigator brochure) for that product. |
| Serious Adverse Event (**SAE**) or Serious Adverse Reaction (**SAR**) or Suspected Unexpected Serious Adverse Reaction (**SUSAR**) | Respectively any adverse event, adverse reaction or unexpected adverse reaction that:<br>• results in death<br>• is life-threatening*<br>• requires hospitalisation or prolongation of existing hospitalisation**<br>• results in persistent or significant disability or incapacity<br>• consists of a congenital anomaly or birth defect<br>• Other important medical condition*** |

**Clarifications and Exceptions**

*The term 'life-threatening' in the definition of 'serious' refers to an event in which the patient was at risk of death at the time of the event; it does not refer to an event which hypothetically might have caused death if it were more severe.

**Hospitalisation is defined as an inpatient admission, regardless of length of stay, even if the hospitalisation is a precautionary measure for continued observation. Hospitalisations for a pre-existing condition (including elective procedures that have not worsened) do not constitute an SAE.

***Medical judgement should be exercised in deciding whether an AE/AR is serious in other situations. Important AE/ARs that are not immediately life-threatening or do not result in death or hospitalisation but may jeopardise the subject or

may require intervention to prevent one of the other outcomes listed in the definition above, should also be considered serious.

Pregnancy occurring in a STAMPEDE patient's partner during the patient's participation in the trial, must be reported to the MRC CTU within the same timelines as an SAE and classified as an 'other important medical condition' on the SAE form. The outcome of a pregnancy should be followed up carefully and any abnormal outcome to the mother or child should be reported.

#### 11.1.1 Trial-Specific Exemptions

Disease progression or death as a result of disease progression are not considered to be SAEs and should be reported on the STAMPEDE Progression Form or Death Form.

The following situations that fulfil the definition of an SAE are excluded from expedited notification on an SAE form and should be reported only on the STAMPEDE follow-up form:

- Elective hospitalisation and surgery for treatment of locally advanced or metastatic prostate cancer or its complications
- Elective hospitalisation to simplify treatment or procedures
- Elective hospitalisation for pre-existing conditions that have not been exacerbated by trial treatment

### 11.2 INSTITUTION/INVESTIGATOR RESPONSIBILITIES

All non-serious AEs/ARs, whether expected or not, should be recorded in the toxicity (symptoms) section of the Follow-up CRF and sent to the MRC CTU within one month of the form being due. SAEs/SARs should be notified to the MRC CTU as described below.

The severity (i.e. intensity) of all AEs/ARs (serious and non-serious) in this trial should be should be graded using Common Terminology Criteria for Adverse Events (CTCAE) v3.0 (ctep.cancer.gov/reporting/index.html). A flowchart is given in **Appendix I** to help explain the notification procedures. Any questions concerning this process should be directed to the MRC CTU in the first instance.

#### 11.2.1 Investigator Assessment

##### 11.2.1.A Seriousness

When an AE/AR occurs the investigator responsible for the care of the patient must first assess whether the event is serious using the definitions given in **Table 14**. If the event is serious and not exempt from expedited reporting, then an SAE form must be completed and the MRC CTU notified.

##### 11.2.1.B Causality

The Investigator must assess the causality of all serious events/reactions in relation to the trial therapy using the definitions in **Table 15**. There are 5 categories: unrelated, unlikely, possible, probable and definitely related. If the causality assessment is unrelated or unlikely to be related the event is classified as a SAE. If the causality is assessed as either possible, probable or definitely related then the event is classified as a SAR.

**Table 14: Assigning type of SAE through causality**

| RELATIONSHIP | DESCRIPTION | EVENT TYPE |
|---|---|---|
| Unrelated | There is no evidence of any causal relationship | Unrelated SAE |
| Unlikely | There is little evidence to suggest there is a causal relationship (e.g. the event did not occur within a reasonable time after administration of the trial medication). There is another reasonable explanation for the event (e.g. the patient's clinical condition, other concomitant treatment). | Unrelated SAE |
| Possible | There is some evidence to suggest a causal relationship (e.g. because the event occurs within a reasonable time after administration of the trial medication). However, the influence of other factors may have contributed to the event (e.g. the patient's clinical condition, other concomitant treatments). | SAR |
| Probable | There is evidence to suggest a causal relationship and the influence of other factors is unlikely. | SAR |
| Definitely | There is clear evidence to suggest a causal relationship and other possible contributing factors can be ruled out. | SAR |

#### 11.2.1.C Expectedness

If the event is a SAR the Investigator must assess the expectedness of the event. Please see Appendix G (Table G.2) for a list of expected toxicities associated with the drugs being used in this trial. If a SAR is assessed as being unexpected it becomes a SUSAR.

#### 11.2.1.D Notification

Investigators must notify the MRC CTU of all SAEs occurring from the time of randomisation until 30 days after the last protocol treatment administration. Similarly, SAEs occurring in patients randomised to Arm A must be reported until 2 months after last injection or progression (whichever is sooner). SARs and SUSARs must be notified to the MRC CTU indefinitely (i.e. no matter when they occur after randomisation).

### 11.2.2 NOTIFICATION PROCEDURE

The SAE form must be completed by the Investigator (consultant named on the signature list and delegation of responsibilities log who is responsible for the patient's care), with due care being paid to the grading, causality and expectedness of the event as outlined above. In the absence of the responsible investigator the form should be completed and signed by a member of the site trial team. The responsible investigator should subsequently check the SAE form, make changes as appropriate, sign and then re-fax to the MRC CTU as soon as possible. The initial report shall be followed by detailed, written reports as appropriate.

Send the SAE form by fax to the MRC CTU. Fax Number: + 44 (0) 20 7670 4818. The STAMPEDE trial team will confirm receipt of the SAE report to the main point of contact via email

Follow-up: Patients must be followed-up until clinical recovery is complete and laboratory results have returned to normal or baseline, or until the event has stabilised. Follow-up should continue after completion of protocol treatment if necessary. Follow-up information should be noted on a

further SAE form by ticking the box marked ‘follow-up’ and faxing to the MRC CTU as information becomes available. Extra, annotated information and/or copies of test results may be provided separately. The patient must be identified by trial number, date of birth and initials only. The patient’s name should not be used on any correspondence.

## 11.3 MRC CTU RESPONSIBILITIES

Medically qualified staff at the MRC CTU and/or the Chief Investigator (or a medically qualified delegate) will review all SAE reports received. The causality assessment given by the local Investigator at the hospital cannot be overruled and in the case of disagreement, both opinions will be provided in any subsequent reports.

The MRC CTU is undertaking the duties of trial sponsor and is responsible for the reporting of SUSARs and other SARs to the regulatory authorities (MHRA and competent authorities of other European member states and any other countries in which the trial is taking place) and the research ethics committees as appropriate.

The MRC CTU will also keep all investigators informed of any safety issues that arise during the course of the trial.

**SAE REPORTING**
Fax to 020 7670 4818 within 24 hours of becoming aware of the event

# 12 ETHICAL CONSIDERATIONS AND APPROVAL

## 12.1 ETHICAL CONSIDERATIONS

This is a randomised trial therefore neither the patients nor their physicians will be able to choose the patients' treatment. Treatment will be allocated randomly using a computer-based algorithm. This is to ensure that the groups of patients receiving each of the different treatments are similar.

Androgen deprivation therapy alone is the standard treatment for these forms of prostate cancer. Patients will be randomised to one or two of the newer treatments in combination with hormone treatment. The trial has employed an unequal allocation ratio for some comparison to maximise efficiency; this was explained in detail in the patient information sheet.

The newer combined treatment options are being assessed in a detailed and systematic fashion in this trial. There is some evidence to suggest that the newer treatment options may have advantages over standard treatment (androgen deprivation therapy) alone with regards clinical outcome, but this is not confirmed and toxicity may be increased. This trial will follow a large group of men who have been randomly allocated to either the standard treatment (androgen deprivation therapy alone) or the newer combined treatment options in order to measure the benefits of the new treatments. The patients will also be followed-up for toxicity and safety issues, so that any benefits can be weighed against any negative aspects.

Patients participating in the trial will have some additional hospital visits and some extra blood samples taken compared to patients who are not participating in the trial, with the amount varying according to the allocated treatment. Sometimes the blood samples can be taken when the patient is attending hospital for treatment, anyway. On some of the trial arms, the patient may have to make additional visits to the hospital for the blood sample to be taken, although in some cases it may be possible for the blood sample to be taken in the GP's surgery. The additional visits and blood samples are to ensure that follow-up of patients is comparable in all the treatment groups. The blood samples will also be used for genetic and serum marker studies, where this information will be considered with clinical data. Blood samples will be link-anonymised. There will be no feedback to individual patients.

If new information emerges during the course of the trial which may affect the treatment or follow-up of patients who have joined the trial, information will be provided through by the trial team to all Principal Investigators. PIs have therefore the duty to inform patients in their care of any new information emerged using any appropriate channel (e.g. letter, communication at follow up clinic, etc).

## 12.2 ETHICAL APPROVAL

The protocol has a Favourable Opinion from an appropriate Research Ethics Committee, according to national guidelines. Additionally, each site must also obtain management permission for research (R&D approval) from the relevant host organisations before patients can be entered into the trial. The patient's informed consent to participate in the trial should be obtained after a full explanation has been given of the treatment options, including the conventional and generally accepted methods of treatment. Patient information sheets and patient consent forms are given in **Appendix B**.

The right of the patient to refuse to participate in the trial without giving reasons must be respected. After the patient has entered the trial, the clinician must remain free to give alternative treatment to that specified in the protocol, at any stage, if he feels it to be in the best interest of the patient. However, the reason for doing so should be recorded and the patient will remain within the trial for the purpose of follow-up and data analysis according to the treatment option to which he has been allocated. Similarly, the patient must remain free to withdraw at any time from the protocol treatment without giving reasons and without prejudicing his further treatment.

A statement of MRC policy on ethical considerations in clinical trials of cancer therapy, including the question of informed consent, is available from the MRC Head Office web site (http://www.mrc.ac.uk).

# 13 REGULATORY APPROVAL

This trial has been approved in the UK by the MHRA and will be conducted under a CTA (Ref: 00316/0026/001-0001) in the UK.

The trial has been approved in Switzerland by Swissmedic (Ref: 2009 DR 3235).

# 14 INDEMNITY

University College London holds insurance against claims from participants for injury caused by their participation in this clinical trial. Participants may be able to claim compensation if they can prove that UCL has been negligent. However, as this clinical trial is being carried out in a hospital, the hospital continues to have a duty of care to the participant of the clinical trial. University College London does not accept liability for any breach in the hospital's duty of care, or any negligence on the part of hospital employees. This applies whether the hospital is an NHS Trust or otherwise.

Participants may also be able to claim compensation for injury caused by participation in this clinical trial without the need to prove negligence on the part of University College London or another party. Participants who sustain injury and wish to make a claim for compensation should do so in writing in the first instance to the Chief Investigator, who will pass the claim to the managing organisation's Insurers, via the managing organisation's office.
Hospitals selected to participate in this clinical trial must provide clinical negligence insurance cover for harm caused by their employees and a copy of the relevant insurance policy or summary can be provided on request.

# 15 FINANCE

STAMPEDE is funded by the Clinical Trials Advisory Awards Committee (CTAAC) on behalf of Cancer Research UK; it is also funded by the MRC through the MRC Clinical Trials Unit. The trial has National Cancer Research Network (NCRN) approval and, therefore, local NCRN funds may be available at each centre to support entry of patients into this trial.

**Zoledronic acid** is manufactured by Novartis. Novartis have agreed to provide an educational grant to support the conduct of this study. Novartis have also agreed to supply the study drug, zoledronic acid free of charge for patients participating in the study.

**Docetaxel** is manufactured by Sanofi-Aventis Pharma. They have agreed to supply the study drug, docetaxel at a discounted rate for patients that are participating in the trial and to provide an educational grant to support the conduct of the study. The Department of Health has agreed to provide a central subvention as follow: £1,787 per patient randomised to Arms C and E of the trial and prescribed docetaxel. This amount is payable in respect of a hospital trust randomising more than 3 patients. For more details contact the STAMPEDE Trial Manager.

**Celecoxib** is manufactured by Pfizer. They agreed to supply free drug and to provide funds to distribute drug to participating sites.

**Abiraterone** is manufactured by Janssen Pharma PV (pharmaceutical companies of Johnson & Johnson). They have agreed to provide free drug and funds to distribute drug to participating sites and to help support the conduct and management of the trial.

**Enzalutamide** is manufactured by Astellas Pharma. They have agreed to provide free drug and funds to distribute drug to participating sites and to help support the conduct and management of the trial.

# 16 TRIAL COMMITTEES

## 16.1 TRIAL MANAGEMENT GROUP (TMG)

A Trial Management Group (TMG) has been formed comprising the Chief Investigator, other co-investigators and members of the MRC CTU. The membership of the TMG may be expanded if other groups of trialists wish to participate. It will also be amended during the trial if other circumstances require e.g. retirement.

The TMG will be responsible for the day-to-day running and management of the trial and will meet by teleconference at least 3 monthly and in person as needed. The TMG members are detailed in **Appendix K**.

Further details of TMG functioning are provided in the TMG charter (available on request).

## 16.2 TRIAL STEERING COMMITTEE (TSC)

A Trial Steering Committee (TSC) has been formed to provide overall supervision for the trial and provide advice through its independent chair. The ultimate decision for the continuation of the trial lies with the TSC. The TSC will meet regularly.

Further details of TSC functioning are provided in the TSC charter (available on request).

## 16.3 INDEPENDENT DATA MONITORING COMMITTEE (IDMC)

An Independent Data Monitoring Committee (IDMC) has been formed. The IDMC will be the only group who sees the confidential, accumulating data to the trial. Reports to the IDMC will be produced by the MRC CTU. The IDMC will meet within 6 months of the trial opening with the frequency of meetings dictated by the IDMC. The IDMC will consider data in accordance with the analysis plan (see **Section 9.5**) and will be advisory to the TSC. The IDMC can recommend premature closure or reporting of the trial, or that recruitment to any research arm be discontinued.

From version 8.0 onwards, any recommendation from the IDMC to stop recruitment to one or more trial arms will be acted upon immediately, pending ratification from the TSC. As this period between meetings should be very short, sites would not be notified until after the TSC have made a decision. IDMC recommendations based on emerging safety issues would be discussed with sites promptly.

Further details of IDMC functioning and the procedures for interim analysis and monitoring are provided in the IDMC charter (available on request).

### Figure 12: Diagram of relationships between trial committees

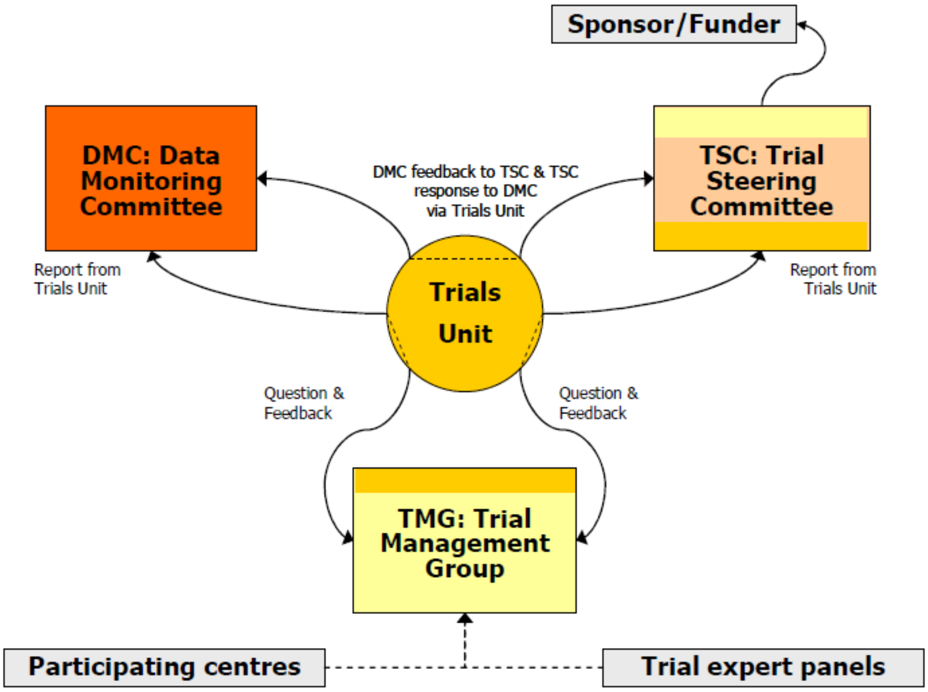

# 17 ANCILLARY STUDIES

## 17.1 QUALITY OF LIFE

A quality of life (QL) study is being performed to assess the impact of each treatment arm on the quality of patient's lives and participation in this study was limited to the first 700 patients recruited (this was reached in September 2008) patients. The QL study re-opened from the implementation of version 8.0 of the protocol. The EORTC QLQ-C30 with the prostate-specific module QLQ PR25 will be used. Key items for assessment are pain reduction for patients with metastatic disease and urinary symptoms for patients with locally advanced disease. In addition specific hypotheses will be generated for each of the research arms. The EuroQol (EQ-5D) (63) will be used in the study as a generic measure of health-related quality of life which can be linked to public preferences. These data will be used to calculate quality-adjusted life-years as part of the economic evaluation (see **Section 17.2**). Patients who were recruited into the QL study, should continue on the study throughout the trial. Questionnaires should be self-administered, although it is recommended that a key person (e.g. research nurse) at each centre be responsible for the data collection to optimise compliance and completeness of the data.

The QL and the HE questionnaires should be completed without conferring with friends or relatives and all questions should be answered even if the patient feels them to be irrelevant.

The responsible person should check each questionnaire for its completeness, ensuring that the correct date of completion and patient identifiers are present. The research nurse should approach patients at appropriate clinical visits to complete a questionnaire. If no clinical visit is scheduled for the patient (with a window of 4 weeks around the expected date) the nurse should organise the completion of the questionnaire, by post or by a visit to the patient at home (or in a hospice).

## 17.2 HEALTH ECONOMICS

A health economics (HE) sub-study will be performed. Core resource use information will be collected, using CRFs on days in hospital (by speciality) and outpatient visits. Data being collected on concomitant medication will also be used in the economic analysis. Information on patients' use of primary care and community-based services will be collected as additional questions in the QL questionnaire. Costs will be calculated on the basis of representative UK unit costs at the point of analysis. Health outcomes will be assessed in terms of quality-adjusted life years (QALYs). Quality adjustments will be based on patients' responses to the EQ-5D health status measure which will be administered at baseline and each point of follow-up as part of the QL questionnaire. A cost-effectiveness analysis will compare all regimens that continue to recruit into their Activity Stage IV.

## 17.3 TRANSLATIONAL SUB-STUDIES

### 17.3.1 DNA Analysis

Blood samples from as many patients as possible will be collected for future research. With patient consent, an additional droplet of blood sample will be collected and stored for DNA and protein analysis in order to try to identify molecular features of clinical significance.

Blood samples should be sent directly to the central laboratory on the FTA elute cards provided. Patient information sheets and consent forms which highlight this research are given in **Appendix B**, while details of specimen collection, posting and contact details are given in **Appendix D**.

#### 17.3.2 Tissue Microarray

Patient consent will be sought to utilise paraffin embedded tissue for the construction of tissue microarrays from needle cores. One needle biopsy will be selected for microarray and the remaining tissue will be returned to the originating histopathology lab. Given the entry criteria for the trial, the majority of patients will have extensive disease in the diagnostic needle core biopsies, in contrast to men with localised, low grade disease. Consequently, removal of one core is unlikely to compromise any subsequent histopathological assessment. Details regarding transfer of samples will be issued at the time of construction of the micro array. Additional analyses e.g. DNA extraction may also be performed on the tissue arrays.

# 18 PUBLICATION

The results from different centres will be analysed together and published as soon as possible. Individual clinicians must not publish data concerning their patients that are directly relevant to questions posed by the study until the TMG has published its report. The TMG together with the STAMPEDE collaborators will form the basis of the writing committee and decide on the nature of publications. Any release, of efficacy or safety data, presentation or publication will be agreed with the TSC according to the terms of their charter.

All publications will acknowledge the participating centres and clinicians, and these will be detailed in an appendix to the main report. Papers will have named authors determined by the TMG according to the following principles:

- To be as inclusive as possible where this is practicable
- To ensure that there is justification for anyone to be named as an author
- Reasons for nomination for authorship may include: trial design; grant holding; day-to-day trial oversight (TMG membership); analysis; discussion and interpretation of data; representation for key groups; active participation at large recruiting sites. It should be accepted that the people qualifying for authorship will vary over time. In addition, key positions will vary depending on the nature of the publication: clinical lead for clinical papers, statistician lead for methodology papers, translational papers may be led by authors not on the main TMG if appropriate (e.g., the bone sub-study). In the event of any dispute related to authorship or data release, the TSC will be responsible for making the executive decision.

In the manuscript, a full list of sites and the number of patients recruited will be provided. In the presentations, this list of sites will also be shown. The term “the STAMPEDE investigators” will clearly be stated and relevant names included in the presentation credits.

A detailed Publication Plan is documented elsewhere.

# 19 PROTOCOL AMENDMENTS

## 19.1 PROTOCOL

### 19.1.1 AMENDMENTS MADE TO SECTIONS IN PROTOCOL VERSION 1.0 (MAY 2004)

Administrative changes such as typos, word change etc.

Name additions/changes to:

TMG members

TSC members

IDMC members

'General Information' Section – additional information re. Abridged version of protocol

Section 1.2 – Figure 1, Celecoxib duration amended

Section 1.3 – Figure 2, addition of cardiovascular assessment form, name and timings amended

Section 2.3 – Docetaxel information updated

Section 2.4 – Additional text re dose and duration justification for Celecoxib use.

Section 3 – Title change and content updated

Section 4.2 – New exclusion criteria added

Section 4.3.1 – New investigations added and additional text re testosterone measurements and additional text re. prior celecoxib treatment

Section 6.1.4 – Celecoxib duration amended

Section 6.1.5 – Additional text re. Co-administration of docetaxel and bisphosphonates

Section 6.1.6 – Celecoxib duration amended

Section 6.2.2 – additional docetaxel information

Section 6.2.3 – addition of CV event history

Section 11 – Safety reporting updated

Section 12.1 – Additional text re. the collection of blood for genetic and serum marker studies

Section 15 – Additional information re. Central Subvention for docetaxel arms

### 19.1.2 AMENDMENTS MADE TO SECTIONS IN PROTOCOL VERSION 1.1 (MAY 2005)

Section 6.2 Administration and Dose Modifications, subsection 6.2.1 Zoledronic Acid

### 19.1.3 AMENDMENTS MADE TO SECTIONS IN PROTOCOL VERSION 2.0 (JUN 2005)

General Information section – SAE reporting fax number and timeframe added.

Section 1.2 – Addition of anti-androgen use for M0 patients as a method of HT

Section 1.2 – Increase in amount of blood needed & addition tissue sample request.

Section 1.3 Trial Documentation updated to include new table detailing trial documentation ahead of accreditation, the inclusion of the radiotherapy forms and correct case report form timings

Section 2.1 – Addition of anti-androgen use for M0 patients as a method of HT

Section 4.1.3 – Inclusion criteria Vii "Normal testosterone prior to hormone treatment" removed.

Section 4.1.3 - φnote has been omitted and moved to section 4.2 (see number 8)

Section 4.2 – Exclusion criteria added to exclude patients with active peptic ulceration, gastrointestinal bleeding and inflammatory bowel disease.

Section 4.2 – Exclusion Criteria added to exclude patients with planned major dental work

Section 4.3.1 - All blood test timelines changed from 14 days to 28 days.

Section 4.3.1 – Hormone Therapy pre-randomisation deadline extended from 4 weeks to 12 weeks.

Section 4.3.1 – Additional information regarding the use of NSAIDs and cox-2-inhibitors before coming on to the STAMPEDE study and once commenced on study treatment

Section 4.3.2 – Updated to ask for all vitamins and minerals the patient is taking to be recorded.

Section 4.3.3 – Updated to include the extra blood required and the request for consent of patients' tissue samples.

Section 6.1.1 – Addition of anti-androgen use for M0 patients as a method of HT

Section 6.1.6 – Addition of the calcium & vitamin name "calcichew".

Section 6.6.2 – asking also to collect vitamins and minerals under concomitant medication.

Section 6.6.3 – New section to inform investigators that patient's, who they wish to give radiotherapy to, are also eligible for STAMPEDE

Section 6.6.4 – New section to detail what data is being collected on the radiotherapy given to patients.

Section 7.1; figure 4 – Addition of radiotherapy form and in note, addition of AA alone

Section 7.1.2 – omission of repeated scans and x-rays at 24 weeks, also omitted in note under figure 4.

Chapter 11 – Safety reporting section updated

Section 17.3 – Increase in amount of blood needed & additional tissue sample request.

### 19.1.4 Amendments Made To Section In Protocol Version 3.0 (Jul 2006)

Front Cover - NCRN logo added for accuracy

Front Cover - Clarification that protocol developed with NCRI rather than on behalf of

Front Cover - Clarification that it is a 6 arm trial

General Information section - MRC CTU staff section updated

Section 1.2 – Statistics section updated.

Section 1.2 - Additional research paragraph updated to reflect additional studies and for clarification of terms

Section 1.2 - Blood collection volume changed to reflect new technique used

Section 1.3 (figure 3) - Table showing case report form schedule updated to reflect clarification of follow-up schedule and addition of new CRF (End of Treatment)

Section 2.2 - AS changed to HT (clarification of terms)

Section 2.3 - Updated in information in regard to use of docetaxel added to reflect up to date practice

Section 2.5 - Sub-headings numbered for consistency

Section 3.0 - Information in regard to the Pilot Phase now written in past tense as Pilot Phase has now been completed

Section 4.1.1 - Inclusion criteria extended so that patients who fulfil 2 out of the three of the first inclusion criteria can be eligible.

Section 4.3.1 - Change in time scales by which baseline investigations need to be completed.

Section 4.3.1 - Clarification that chest X-ray is only required if chest is not included in the CT

Section 4.3.1 - Removal of 12 week timeline for baseline PSA test to be performed. (Stipulation that it must be performed before start of HT)

Section 4.3.2 – Information added in regard to time allowed from randomisation to start of treatment

Section 4.3.3 - Additional research paragraph updated to reflect additional studies and for clarification of terms

Section 4.3.3 - Blood collection volume changed to reflect new technique used

Sections 6.1.2-6.1.6 - Androgen Suppression replaced with hormone therapy for consistency of terms

Section 6.2.2 - '(Taxotere)' Removed for consistency

Section 6.2.2 _ information added in regard to the need to closely monitor liver function prior to docetaxel administration

Section 7.1 - Page number reference updated

Section 7.1.1 - PSA measurement timings updated to accurately reflect follow-up schedule

Section 7.3 (Table 4) - Table and key updated to accurately reflect follow-up schedule and to include information about new CRFs and removal of withdrawal CRF

Section 8 - Rewording for clarification of definition of trial withdrawal

Section 8.1 - Instruction that withdrawal from trial treatment should be recorded on End of Treatment Form rather than withdrawal form

Section 8.1 - Information updated to emphasise that trial treatment must be discontinued following a progression

Section 8.2- Information added in regard to patient transfers

Section 8.3 - Instruction that withdrawal from trial completely must be notified in writing to the MRC CTU rather than included on withdrawal form

Section 9 and Summary – Target event numbers updated to reflect the slightly revised numbers obtained by using –nstage- which is the new, recommended program for MAMS trials

Sections 11.1 and 11.2 - Form numbers removed to allow for future changes in numbering

Section 11.2 – Reference to toxicity grading website added

Section 11.2.1 - Reference to table in appendix G added

Section 12.2 - 'Suggested' removed from 'Suggested patient information sheets'

Section 13 - CTA reference added

Section 17.3 - Information added to reflect new blood collection method for DNA analysis and in regard to additional translational studies for which funding has recently been approved

### 19.1.5 Amendments Made To Protocol Version 4.0 (Dec 2007)

General Information Section - Randomisation and SAE reporting details sections clarified

Section 1.2 and throughout protocol - Efficacy Stages 1-111 renamed to Activity Stages 1-111 for accuracy and clarity

Section 1.2 - Follow schedule corrected

Section 4.1.2 - Inclusion criteria widened to include high risk relapsing patients, that would not have met the previous PSA based criteria

Section 4.1.3 - Note added to reference location of WHO performance status definitions

Section 4.2 - Notes added to reference locations of toxicity gradings and NYHA classifications

Section 4.3.1 - Timings of baseline scan information changed to accurately reflect most common current practice

Section 6.1.1 - Information about use of LHRH antagonists to ensure that the protocol accurately reflects current and future practice

Section 6.1.1 - Information about suggested duration of hormone therapy added to ensure that the protocol accurately reflects current practice

Section 6.2.2 - Additional information added about the timing of liver function tests prior to docetaxel administration added for clarity

Section 6.6.4 - Information on radiotherapy data collection added

Section 7.1.1 - Erroneous information about the timing of PSA measurements removed

Figure 3 - Moved to new section in protocol for clarity and extended to include current information on data collection

Figure 3b - Added to describe how extent of data collection during follow-up should change, post treatment and post progression

Figure 4 - Notes added to explain the changes in data collected at follow-up and to information that the quality of life study will be applicable to the first 700 patients randomised only

Figure 4 - Note added to include palliative radiotherapy CRF

Section 11.3 - SAE reporting information updated

Section 19 - Protocol amendments list updated

### 19.1.6 Amendments Made To Protocol Version 5.0 (Aug 2008)

1. General Information Section – Randomisation phone line number updated – non UK extension added

2. Section 3 – Information about QL study removed to reflect closure of QL study after first 700 patients

3. Section 4.2 – Exclusion criteria clarified to explain that only patients with severe poor cardiovascular history should be excluded

4. Section 4.3.1 – Information on co-administration of NSAIDS with celecoxib changed based on clinical advice.

5. Section 5 - Randomisation phone line number updated – non UK extension added

6. Section 6.2.1. – Information added to clarify that patients who develop an osteonecrosis of the jaw should stop zoledronic acid treatment

7. Section 6.2.3 – 'severe' text added to accurately reflect which patients should be excluded based on their cardiovascular history

8. Section 7.1.2 – Definition of disease progression extended for clarity

9. Figure 3 – Updated to include reference to newly created skeletal related event form

10. Figure 4 – Previous error in table amended to show that the 4th Zoledronic Acid form that is submitted contains information about 3 cycles rather than 2 as previously indicated

11. Table 4 – 'Other important medical condition' added to definition of serious in the SAE section, to accurately reflect SAE form and current practice

12. Section 11.1 – Information added on reporting or pregnancies

13. Section 17 - Information about QL study removed to reflect closure of QL study after first 700 patients

### 19.1.7 Amendments Made To Protocol Version 6.0 (Jul 2009)

1. General Information Section – Trial Pharmacist removed and changes of:

Co-Investigator

Patient Representatives

Trial Manager

Data Manager

General Information Section - Coordinating Centre – address change

General Information Section – change of Sponsor address

Section 1.1 – ratio of patients randomised to the investigational arms updated

Section 1.2 – figure 1b added to clarify trial design from Apr-2011 onwards

Section 1.2 – paragraph added to explain trial changes after the second activity analysis

Section 1.2 – wording added to clarify that QL data only collected for first 700 patients randomised

Section 1.3 – SSA Favourable Opinion removed from list of trial documentation required ahead of site accreditation

Section 2.1 – Amount of men diagnosed with prostate cancer annually updated

Section 2.4 –note added to explain completion of recruitment to celecoxib- containing arms

Section 2.5.2 - note added to explain completion of recruitment to celecoxib- containing arms

Section 3 – SSA Favourable Opinion removed

Section 4.2 – Exclusion criterion xiii greyed out

Section 4.3.1 – paragraph removed regarding potential randomisation to celecoxib-containing arms

Section 5 – Randomisation instructions expanded to exclude public holidays or dates when notice has been given by the CTU

Section 6.1.4 – formatting changed to grey font to reflect recruitment completion for arm D

Section 6.1.6 - formatting changed to grey font to reflect recruitment completion for arm F

Section 6.2.3 – recruitment note added

Section 6.6.3 – radiotherapy statement changed to reflect data from recent trials

Section 7.1.2 – removal of reference to SRE- specific CRF

Section 7.3 – Figure 3 - Addition of Bone Density Risk Factor Form and BMD sub-study assessment forms to summary of timing table

Section 7.3 – Figure 4 – Weeks added to timings of assessments post 2 years

Section 7.3- Figure 4 – note added to explain recruitment completion for arms D and F

Section 12.1 – Wording changed to reflect change to randomisation allocation ratio

Section 12.1 – Addition of statement regarding new information emerging during the trial

Section 12.2 – Reference to SSA removed

Section 16.3 – Statement added regarding actioning IDMC recommendation ahead of TSC ratification

### 19.1.8 Amendments Made To Protocol Version 7.0 (Jul 2011)

1. General Information Section- SAE reporting fax number corrected

2. Section 11- SAE reporting fax number corrected

### 19.1.9 Amendments Made To Protocol Version 7.1 (Jul 2011)

Throughout protocol – numbering has been updated in some sections new accommodate new information that has been added.

General Information Section – contact details updated

General Information Section – Funding information updated to include involvement from additional company

General Information Section – Wording on compliance and regulations updated to reflect current MRC CTU standard wording

General Information Section – Abbreviations list updated

Section 1.1 – The number of investigational agents being studied updated from three to four

Section 1.1 – Information regarding celecoxib updated to reflect that recruitment to these arms was discontinued in Apr-2011

Section 1.1 – Information about new IMP, Abiraterone inserted

Section 1.1 – Sample size and trial duration information updated to reflect changes brought about by additional trial arm

Section 1.2 – Summary information updated to reflect the discontinuation of recruitment to celecoxib arms and the addition of abiraterone

Figures 1a, b and c - Updated to reflect the discontinuation of recruitment to celecoxib arms and the addition of abiraterone

Section 1.2 – Information on trial stages updated to reflect changes brought about by additional trial arm

Section 1.2 – Information updated regarding the re-opening of the quality of life sub-study from implementation of protocol version 8.0

Section 2.1 – Wording related to hormone therapy updated for clarity

Section 2.1 - Updated to reflect the discontinuation of recruitment to celecoxib arms and the addition of abiraterone

Section 2.2 – Updated references added

Section 2.3 – Updated references added

Section 2.5 – Section added to give background information on new IMP, abiraterone

Section 2.6.1 – Updated references added

Section 2.7 – Section added to give information regarding radiotherapy which is to be given as part of standard care following recently published trial data.

Section 3 – Wording updated regarding selection of investigators to reflect current MRC CTU practice

Section 4.1 – Inclusion criteria updated with new criterion regarding radiotherapy use

Section 4.1 - Inclusion criteria updated with new criterion regarding contraceptive use

Section 4.1 – Wording of inclusion and exclusion criteria updated for clarity

Section 4.1 – Exclusion criteria updated with new criterion regarding acceptable liver function for trial entry

Section 4.1 – Exclusion criteria updated with specifics related to blood pressure levels

Section 4.1 - Exclusion criteria updated with new criterion regarding concomitant medications

Section 4.1 - Exclusion criteria updated with new criterion regarding prior treatment with abiraterone

Section 4.1 - Exclusion criteria updated with new criterion regarding prior treatment with chemotherapy

Section 4.1 - Exclusion criteria updated with new criterion regarding prior treatment with zoledronic acid

Section 4.3 – Wording updated to reflect that patients who initially fail screening can be re-screened at a later date

Section 4.3.2 – Wording updated regarding prior anti-androgen and LHRH use updated for clarity

Section 5.1 – Co-enrolment guidelines information updated to describe newly created co-enrolment CRF

Section 6.1 – Trial treatment information updated to reflect the fact that anti-androgens alone will be no longer permitted as hormone therapy

Section 6.1.1 – Updated to describe patients for whom radiotherapy should be given as standard practice

Section 6.1.1 a and b - Sections added to give information regarding radiotherapy treatment

Section 6.1.1-6.1.6 – References to further sections updated

Section 6.1.7 – Section added to describe abiraterone treatment

Section 6.2.4 - Section added to describe abiraterone treatment

Section 6.6 - Section added to give information regarding radiotherapy treatment

Section 7.1.1 – Reference to blood being taken at patient's home removed as this does not occur in practice

Section 7.1.2 – Wording updated regarding the reporting of biochemical failures for clarity

Section 7.1.2 – Wording updated regarding skeletal-related events for clarity

Section 7.1.3 – Section added to describe additional assessments required related to abiraterone treatment

Section 7.1.4 – Section added to provide information on when treatment should commence

Figure 4 – Updated for clarity regarding return of BMD sub-study forms, the addition the co-enrolment CRF and the description of the re-opening of the QoL Sub-study.

Figure 5 – Updated with reference to abiraterone and co-enrolment form

Section 7.3 - Wording on trial closure updated to reflect current MRC CTU standard wording

Section 8.1 – Additional criteria for definition of progression added for clarity

Section 8.1 – Definition of progression for abiraterone patients added.

Section 9 – Statistical information updated to describe the addition of the new trial arm

Section 11 – Safety reporting wording updated for clarity

Section 11 – SAE reporting fax number updated

Section 12 – Ethical information updated to describe the unequal randomisation allocation ratio

Section 12 – Ethical information updated to describe that the visit schedule will vary according to trial arm

Section 12.2 – Wording updated to reflect international participation in the trial

Section 13 – Wording updated to reflect international participation in the trial

Section 14 – Wording updated to reflect international participation in the trial

Section 15 - Updated to reflect the discontinuation of recruitment to celecoxib arms and the addition of abiraterone

Section 16 – Reference to trial committee charters added for information

Section 17.1 – Information added to reflect re-opening of quality of life sub-study

Section 17.2 – Timing of health economics analysis updated to previous error

Section 18 – Information on publication policy expanded for clarity

Section 19 – Information regarding amendments to protocol appendices moved to the separate appendices document

Section 20 – References extensively updated

### 19.1.10 Amendments Made To Protocol Version 8.0 (Sep 2011)

Throughout protocol – numbering, sections headings, tables, figures and bibliographical references have been updated in some sections to accommodate new information that has been added

Throughout protocol – Androgen Deprivation Therapy has replaced Hormone Therapy as deemed more representative of the type of hormone therapy used in the study

General Information Section – New staff members of the MRC CTU and Co-Investigators added and contact details updated

General Information Section – Abbreviations list updated

Section 1.1 – Information regarding the new research radiotherapy treatment inserted

Section 1.1 – Information regarding docetaxel updated

Section 1.2 – Wording updated to reflect the addition of the new research comparison arm

Section 1.3 – Additional criteria for the re-accreditation of participating centres (for protocol version 9.0 only)

Section 2.1.1 – Wording updated to clarify the use of anti-androgen in trial patients

Section 2.1.2 – Information added to describe the rationale for the RT comparison arm

Section 2.8 – Information added to describe research RT treatment to prostate for patients with newly diagnosed metastatic disease

Section 3.1 – Information added to describe RT Quality Assurance procedures and centre accreditation

Section 4.1.1 to 4.1.3 – Wording updated to clarify inclusion criteria for all patients groups (newly diagnosed non-metastatic, metastatic and relapsing patients)

Section 4.2 – Clarification added on cardiovascular exclusion criteria

Section 4.2 – New exclusion criterion added concerning patients with prior exposure to hormone therapy

Section 4.2 – New exclusion criterion added to reflect the addition of the new RT comparison arm

Section 4.4.1 – Clarification added regarding pre-randomisation checks

Section 4.4.2 – Clarification added regarding permissible hormone therapy duration prior to randomisation

Section 4.4.5 – Information added regarding starting research radiotherapy treatment

Section 4.4.6 – Information updated on concomitant medications

Section 5 – Clarification regarding randomisation allocation added to reflect the addition of the new RT research arm

Section 6.1.8 – Information added to describe the administration of research radiotherapy

Section 6.2.1 – Clarification added regarding the measurement of serum creatinine levels prior to the administration of zoledronic acid

Section 6.2.3 – Clarification regarding the completion of recruitment to the celecoxib containing arms

Section 6.25 – Information added regarding the administration of research radiotherapy treatment

Section 6.6 – Clarification incorporated to describe the administration of standard-of-care radiotherapy

Section 7.1.4 – Information added regarding data collection and non-administration of standard radiotherapy

Section 7.2 – Section updated to include new treatment specific CRFs and timing of CRFs

Section 8.1 – Clarification added for the criteria to stop treatment for patients randomised to arm G

Section 8.2 – Section expanded to include additional details on study patient transfer to different centres

Section 8.3 – Additional sentence inserted to reinforce the importance of compliance with follow up assessments

Section 9.1 – Additional paragraph inserted to clarify the method of randomisation and allocation distribution in the light of the introduction of the new RT arm

Section 9.4 – Wording updated to clarify the assessment of safety data

Section 9.5.4 – Wording updated concerning the end of randomisations to arm G

Section 9.6 to 9.6.4 – Section added describing sample size issues and trial stages for arm H

Section 9.8 – Clarification on intermediate stopping guidelines

Section 9.9 – Clarification on the outline analysis plan

Section 11 – Information on safety reporting updated to reflect the addition of the research RT comparison arm

Section 11 – Clarification added regarding arm A safety reporting timelines

Section 12.1 – Clarification added regarding the Principal Investigator's responsibilities

Section 14 – Indemnity section updated to reflect current MRC policy

Section 16 – Clarification regarding TMG membership

Section 17.3 – Section on Bone Mineral Density sub-study removed

Section 19 – Information regarding amendments to protocol appendices moved to the separate appendices document

Section 20 – References updated

### 19.1.11 Amendments Made To Protocol Version 9.0 (Oct-2012)

Throughout protocol – numbering, sections headings, tables, figures and bibliographical references have been updated in some sections to accommodate the completion of recruitment to original research arms B, C and E.

Throughout protocol – Tenses have been changed to reflect activities that were in the future and which have now been passed.

Section 1 – Figure added and clarifications added to each figure

Section 2 – Previous reference 8 removed

Section 4 – Clarification of acceptable alternatives to bone scans

Section 6.2.5 – Correction of an error defining the PTV: the wording has been reordered

Table 4 – Dose-volume objectives corrected: order swapped

Table 5- Correction CRFs names

Section 17.3.2 – Clarification that DNA may be extracted

### 19.1.12 Amendments Made To Protocol Version 10.0 (Apr-2013)

Throughout protocol – numbering, sections headings, tables, figures and bibliographical references have been updated in some sections

Throughout protocol – typos have been corrected

Section 4 –Clarification of exclusion criteria V (now V and VI)

Section 6 – Timing of orchidectomy prior to randomisation extended to 12 weeks

Section 6 – Clarification of hypokalaemia, blood pressure and fluid retention management

Section 9 – Statistical considerations amended in light of the recruitment extension for the abiraterone comparison

Section 14 - Section updated to reflect the changes in the structure of the MRC CTU (now MRC CTU at UCL) and indemnity arrangements

### 19.1.13 Amendments Made To Protocol Version 11.0 (Sep-2013)

Throughout protocol – numbering, sections headings, tables, figures and bibliographical references have been updated in some sections

Throughout protocol – typos have been corrected

Co-investigators list updated to reflect the addition of the “enzalutamide + abiraterone comparison” lead

Section 1.2 – Enzalutamide added as trial treatment

Section 1.2 – Protocol version 12.0 added to the list of amendments

Section 2.10 – Rationale for the combination of enzalutamide and abiraterone

Section 4.2 – Eligibility criteria amended to reflect the addition of enzalutamide + abiraterone arm

Section 4.4.2 – Wording clarified

Section 6.8 – Clarification regarding end of trial treatment after starting trial therapy

Section 6.10 – Section added to describe enzalutamide and abiraterone treatment for the new research arm (Arm J)

Section 6.11.4.A – Section added to describe the management of toxicities from trial abiraterone

Section 6.11.4.B - Section added to describe the management of toxicities from trial enzalutamide

Section 9.1.4 – Section added to describe the statistical considerations concerning the introduction of Arm J

Section 9.3 – Principles and assumption for the introduction of Arm J added

Section 9.7 and sub-sections – Sample size issues and trial stages for Arm J

Section 9.9 – Details on interim monitoring and analyses for Arm J added

Section 11.2.1.D – Wording clarified regarding safety reporting requirements for control arm

Section 12.1 – Wording clarified

Section 15 – Details on funding for the “enzalutamide + abiraterone comparison” added

Section 19 - Amendments made to protocol updated

Reference list updated

## 20 REFERENCES

1. James ND, Sydes MR, Mason MD, Clarke NW, Anderson J, Dearnaley DP, et al. Celecoxib plus hormone therapy versus hormone therapy alone for hormone-sensitive prostate cancer: first results from the STAMPEDE multiarm, multistage, randomised controlled trial. The lancet oncology. 2012;13(5):549-58. Epub 26-Mar-2012.
2. Cancer Research UK. CancerStats Key Facts: Prostate Cancer. Cancer Research UK. 2011.
3. Sharifi N, Gulley JL, Dahut WL, Sharifi N, Gulley JL, Dahut WL. An update on androgen deprivation therapy for prostate cancer. Endocrine-Related Cancer. 2010;17(4):R305-15.
4. Widmark A, Klepp O, Solberg A, Damber JE, Angelsen A, Fransson P, et al. Endocrine treatment, with or without radiotherapy, in locally advanced prostate cancer (SPCG-7/SFUO-3): an open randomised phase III trial. Lancet. 2009;373(9660):301-8. Epub 2008/12/19.
5. Warde PR, Mason MD, Sydes MR, Gospodarowicz MK, Swanson GP, Kirkbride P, et al. Intergroup randomized phase III study of androgen deprivation therapy (ADT) plus radiation therapy (RT) in locally advanced prostate cancer (CaP) (NCIC-CTG, SWOG, MRC-UK, INT: T94-0110; NCT00002633). J Clin Oncol. 2010;28(18s Supplement: Proceedings of ASCO 2010):Abstr CRA4504.
6. Warde P, Mason M, Ding K, Kirkbride P, Brundage M, Cowan R, et al. Survival Benefit with Combined Androgen Deprivation and Radiation Therapy in Locally Advanced Prostate Cancer – Results of a Phase III Trial. The Lancet. 2011 - in press.
7. Mason M, Sydes M, Parulekar W, Parmar M, Anderson J, Barber J, et al. Final analysis of intergroup randomized phase III study of androgen deprivation therapy (ADT) + radiation therapy (RT) in locally advanced prostate cancer (CaP) (NCIC-CTG, SWOG, MRC-UK, INT: T94-0110). National Cancer Research Institute (NCRI) Cancer Conference 2012. 2012;2012.
8. Saad F, Colombel M. Management of castration-resistant prostate cancer: bisphosphonates and emerging therapies. Expert Review of Anticancer Therapy. 2010;10(12):1991-2002.
9. Sartor O, Halstead M, Katz L, Sartor O, Halstead M, Katz L. Improving outcomes with recent advances in chemotherapy for castrate-resistant prostate cancer. Clinical Genitourinary Cancer. 2011;8(1):23-8.
10. Attard G, Richards J, de Bono JS, Attard G, Richards J, de Bono JS. New strategies in metastatic prostate cancer: targeting the androgen receptor signaling pathway. Clinical Cancer Research. 2011;17(7):1649-57.
11. Lipton A. Improving progression-free and overall survival in patients with cancer: a potential role for bisphosphonates. Expert Opinion on Pharmacotherapy. 2011;12(5):749-62.
12. Lipton A, Small E, Saad F, Gleason D, Gordon D, Smith M, et al. The new bisphosphonate, Zometa (zoledronic acid), decreases skeletal complications in both osteolytic and osteoblastic lesions: a comparison to pamidronate. [Review] [55 refs]. Cancer Investigation. 2002;20 Suppl 2:45-54.
13. Green JR, Muller K, Jaeggi KA. Preclinical pharmacology of CGP 42'446, a new, potent, heterocyclic bisphosphonate compound. Journal of Bone & Mineral Research. 1994;9(5):745-51.
14. Major P, Lortholary A, Hon J, Abdi E, Mills G, Menssen HD, et al. Zoledronic acid is superior to pamidronate in the treatment of hypercalcemia of malignancy: a pooled analysis of two randomized, controlled clinical trials. J Clin Oncol. 2001;19(2):558-67. Epub 2001/02/24.
15. Costa L, Harper P, Coleman RE, Lipton A, Costa L, Harper P, et al. Anticancer evidence for zoledronic acid across the cancer continuum. Critical Reviews in Oncology-Hematology. 2011;77 Suppl 1:S31-7.
16. Rosen LS, Gordon D, Kaminski M, Howell A, Belch A, Mackey J, et al. Long-term efficacy and safety of zoledronic acid compared with pamidronate disodium in the treatment of skeletal complications in patients with advanced multiple myeloma or breast carcinoma: a randomized, double-blind, multicenter, comparative trial. Cancer. 2003;98(8):1735-44. Epub 2003/10/10.

17. Rosen LS, Gordon DH, Dugan W, Jr., Major P, Eisenberg PD, Provencher L, et al. Zoledronic acid is superior to pamidronate for the treatment of bone metastases in breast carcinoma patients with at least one osteolytic lesion. Cancer. 2004;100(1):36-43. Epub 2003/12/24.
18. Saad F, Gleason D, Murray R, Tchekmedyian S, Venner P, Lacombe L, et al. A Randomized, Placebo-Controlled Trial of Zoledronic Acid in Patients With Hormone-Refractory Metastatic Prostate Carcinoma Journal of the National Cancer Institute. 2002;94(19).
19. Dearnaley DP, Sydes MR, Mason MD, Stott M, Powell CS, Robinson AC, et al. A double-blind, placebo-controlled, randomized trial of oral sodium clodronate for metastatic prostate cancer (MRC PR05 Trial). J NatlCancer Inst. 2003;95(17):1300-11.
20. Petrylak DP, Tangen CM, Hussain MH, Lara PN, Jr., Jones JA, Taplin ME, et al. Docetaxel and estramustine compared with mitoxantrone and prednisone for advanced refractory prostate cancer. N Engl J Med. 2004;351(15):1513-20. Epub 2004/10/08.
21. Tannock IF, de Wit R, Berry WR, Horti J, Pluzanska A, Chi KN, et al. Docetaxel plus prednisone or mitoxantrone plus prednisone for advanced prostate cancer. N Engl J Med. 2004;351(15):1502-12. Epub 2004/10/08.
22. Hawkey CJ. COX-2 inhibitors.[comment]. Lancet. 1999;353(9149):307-14.
23. Taketo MM. Cyclooxygenase-2 inhibitors in tumorigenesis (Part II). Journal of the National Cancer Institute. 1998;90(21):1609-20.
24. Taketo MM. Cyclooxygenase-2 inhibitors in tumorigenesis (Part I). Journal of the National Cancer Institute. 1998;90(20):1529-36.
25. Nelson JE, Harris RE. Inverse association of prostate cancer and non-steroidal anti-inflammatory drugs (NSAIDs): results of a case-control study. Oncology Reports. 2000;7(1):169-70.
26. Eberhart CE, Coffey RJ, Radhika A, Giardiello FM, Ferrenbach S, DuBois RN. Up-regulation of cyclooxygenase 2 gene expression in human colorectal adenomas and adenocarcinomas. Gastroenterology. 1994;107(4):1183-8.
27. Roberts RO, Jacobson DJ, Girman CJ, Rhodes T, Lieber MM, Jacobsen SJ. A population-based study of daily nonsteroidal anti-inflammatory drug use and prostate cancer. [see comments.]. Mayo Clinic Proceedings. 2002;77(3):219-25.
28. Basler JW, Piazza GA, Basler JW, Piazza GA. Nonsteroidal anti-inflammatory drugs and cyclooxygenase-2 selective inhibitors for prostate cancer chemoprevention. Journal of Urology. 2004;171(2 Pt 2):S59-62; discussion S-3.
29. Masferrer JL, Leahy KM, Koki AT, Zweifel BS, Settle SL, Woerner BM, et al. Antiangiogenic and antitumor activities of cyclooxygenase-2 inhibitors. Cancer Research. 2000;60(5):1306-11.
30. Steinbach G, Lynch PM, Phillips RK, Wallace MH, Hawk E, Gordon GB, et al. The effect of celecoxib, a cyclooxygenase-2 inhibitor, in familial adenomatous polyposis. New England Journal of Medicine. 2000;342(26):1946-52.
31. Solomon SD, McMurray JJ, Pfeffer MA, Wittes J, Fowler R, Finn P, et al. Cardiovascular risk associated with celecoxib in a clinical trial for colorectal adenoma prevention. New England Journal of Medicine. 2005;352(11):1071-80.
32. Danila DC, Morris MJ, de Bono JS, Ryan CJ, Denmeade SR, Smith MR, et al. Phase II multicenter study of abiraterone acetate plus prednisone therapy in patients with docetaxel-treated castration-resistant prostate cancer. J Clin Oncol. 2010;28(9):1496-501. Epub 2010/02/18.
33. Reid AH, Attard G, Danila DC, Oommen NB, Olmos D, Fong PC, et al. Significant and sustained antitumor activity in post-docetaxel, castration-resistant prostate cancer with the CYP17 inhibitor abiraterone acetate. J Clin Oncol. 2010;28(9):1489-95. Epub 2010/02/18.
34. Ryan CJ, Smith MR, Fong L, Rosenberg JE, Kantoff P, Raynaud F, et al. Phase I clinical trial of the CYP17 inhibitor abiraterone acetate demonstrating clinical activity in patients with castration-resistant prostate cancer who received prior ketoconazole therapy. Journal of Clinical Oncology. 2010;28(9):1481-8.

35. de Bono JS, Logothetis CJ, Molina A, Fizazi K, North S, Chu L, et al. Abiraterone and increased survival in metastatic prostate cancer. New England Journal of Medicine. 2011;364(21):1995-2005.
36. Ryan CJ, Smith MR, de Bono JS, Molina A, Logothetis CJ, de Souza P, et al. Abiraterone in metastatic prostate cancer without previous chemotherapy. N Engl J Med. 2013;368(2):138-48. Epub 2012/12/12.
37. Attard G, Reid AHM, de Bono JS. Abiraterone acetate is well tolerated without concomitant use of corticosteroids. Journal of Clinical Oncology. 2010;28(29):e560-1; author reply e2.
38. Flanigan RC, Salmon SE, Blumenstein BA, Bearman SI, Roy V, McGrath PC, et al. Nephrectomy followed by interferon alfa-2b compared with interferon alfa-2b alone for metastatic renal-cell cancer. N Engl J Med. 2001;345(23):1655-9. Epub 2002/01/05.
39. Mickisch GH, Garin A, van Poppel H, de Prijck L, Sylvester R. Radical nephrectomy plus interferon-alfa-based immunotherapy compared with interferon alfa alone in metastatic renal-cell carcinoma: a randomised trial. Lancet. 2001;358(9286):966-70. Epub 2001/10/05.
40. Warde P, Mason M, Ding K, Kirkbride P, Brundage M, Cowan R, et al. Combined androgen deprivation therapy and radiation therapy for locally advanced prostate cancer: a randomised, phase 3 trial. The Lancet. 2011;378:2104-11. Epub 03-Nov-2011.
41. Neville-Webbe HL, Holen I, Coleman RE. The anti-tumour activity of bisphosphonates. Cancer Treatment Reviews. 2002;28(6):305-19.
42. Witters LM, Crispino J, Fraterrigo T, Green J, Lipton A. Effect of the combination of docetaxel, zoledronic acid, and a COX-2 inhibitor on the growth of human breast cancer cell lines. American journal of clinical oncology. 2003;26(4):S92-7. Epub 2003/08/07.
43. Attard G, Cooper CS, de Bono JS. Steroid hormone receptors in prostate cancer: a hard habit to break? Cancer cell. 2009;16(6):458-62. Epub 2009/12/08.
44. Chen G, Wang X, Zhang S, Lu Y, Sun Y, Zhang J, et al. Androgen receptor mutants detected in recurrent prostate cancer exhibit diverse functional characteristics. The Prostate. 2005;63(4):395-406. Epub 2004/12/24.
45. Taplin ME, Bubley GJ, Ko YJ, Small EJ, Upton M, Rajeshkumar B, et al. Selection for androgen receptor mutations in prostate cancers treated with androgen antagonist. Cancer Res. 1999;59(11):2511-5. Epub 1999/06/11.
46. Veldscholte J, Ris-Stalpers C, Kuiper GG, Jenster G, Berrevoets C, Claassen E, et al. A mutation in the ligand binding domain of the androgen receptor of human LNCaP cells affects steroid binding characteristics and response to anti-androgens. Biochemical and biophysical research communications. 1990;173(2):534-40. Epub 1990/12/14.
47. Zhao XY, Malloy PJ, Krishnan AV, Swami S, Navone NM, Peehl DM, et al. Glucocorticoids can promote androgen-independent growth of prostate cancer cells through a mutated androgen receptor. Nature medicine. 2000;6(6):703-6. Epub 2000/06/03.
48. Attard G, Reid AH, Auchus RJ, Hughes BA, Cassidy AM, Thompson E, et al. Clinical and biochemical consequences of CYP17A1 inhibition with abiraterone given with and without exogenous glucocorticoids in castrate men with advanced prostate cancer. The Journal of clinical endocrinology and metabolism. 2012;97(2):507-16. Epub 2011/12/16.
49. Richards J, Lim AC, Hay CW, Taylor AE, Wingate A, Nowakowska K, et al. Interactions of abiraterone, eplerenone, and prednisolone with wild-type and mutant androgen receptor: a rationale for increasing abiraterone exposure or combining with MDV3100. Cancer Res. 2012;72(9):2176-82. Epub 2012/03/14.
50. Scher HI, Fizazi K, Saad F, Taplin ME, Sternberg CN, Miller K, et al. Increased survival with enzalutamide in prostate cancer after chemotherapy. N Engl J Med. 2012;367(13):1187-97. Epub 2012/08/17.
51. Tombal B, Borre M, Rathenborg P, Werbrouck P, Heidenreich A, Iversen P, et al. Enzalutamide monotherapy: Phase II study results in patients with hormone-naive prostate cancer. Journal of Clinical Oncology. 2013;31(Supplement 6):Abstract 18.

52. Efstathiou E, Titus MA, Tsavachidou A, Hoang A, Karlou M, Wen S, et al. MDV3100 effects on androgen receptor (AR) signaling and bone marrow testosterone concentration modulation: A preliminary report. . 2011 ASCO Annual Meeting, 2011 J Clin Oncol 2011.
53. Locke JA, Guns ES, Lubik AA, Adomat HH, Hendy SC, Wood CA, et al. Androgen levels increase by intratumoral de novo steroidogenesis during progression of castration-resistant prostate cancer. Cancer Res. 2008;68(15):6407-15. Epub 2008/08/05.
54. Tran C, Ouk S, Clegg NJ, Chen Y, Watson PA, Arora V, et al. Development of a second-generation antiandrogen for treatment of advanced prostate cancer. Science. 2009;324(5928):787-90. Epub 2009/04/11.
55. Scher HI, Beer TM, Higano CS, Anand A, Taplin ME, Efstathiou E, et al. Antitumour activity of MDV3100 in castration-resistant prostate cancer: a phase 1-2 study. Lancet. 2010;375(9724):1437-46. Epub 2010/04/20.
56. Efstathiou E, Titus M, Wen A, Miguel AS, Hoang A, De A, et al. The effects of enzalutamide (ENZA) in combination with abiraterone acetate (AA) in patients with bone metastatic castration resistant prostate cancer (mCRPC). Eur J Cancer. 2013;49(ECCO 2013 Supplement):Abstract 2854.
57. Kouloulias V, Giraud J-Y, Davis BJ, Dusserre A, Zurlo A, Bolla M. Quality assurance of the 22961 EORTC trial. A phase III study of the optimal combination of hormonal adjuvant treatment by LHRH analogue and radiation therapy for the management of locally advanced prostate cancer: the dummy run. 2004(0167-8140 (Print)).
58. Royston P, Parmar MKB, Qian W. Novel designs for multi-arm clinical trials with survival outcomes with an application in ovarian cancer. Statistics in Medicine. 2003;22(14):2239-56.
59. Royston P, Barthel FMS, Parmar MKB, Choodari-Oskooei B, Isham V. Designs for clinical trials with time-to-event outcomes based on stopping guidelines for lack of benefit. Trials. 2011;12(1):81.
60. Royston P. nstage: MAMS trial sample size calculator. MRC Clinical Trials Unit, London2009.
61. Haybittle JL. Repeated assessment of results in clinical trials of cancer treatment. The British journal of radiology. 1971;44(526):793-7. Epub 1971/10/01.
62. Peto R, Pike MC, Armitage P, Breslow NE, Cox DR, Howard SV, et al. Design and analysis of randomized clinical trials requiring prolonged observation of each patient. I. Introduction and design. British journal of cancer. 1976;34(6):585-612. Epub 1976/12/01.
63. Dolan P, Gudex C, Kind P, Williams A. Discussion Paper 138: A social tarif for EuroQol: results from a UK General Population Survey. . Centre for Health Economics. University of York, 1995.
