# Supplementary material for: Abiraterone acetate plus prednisolone for metastatic patients starting hormone therapy: 5‐year follow‐up results from the STAMPEDE randomised trial (NCT00268476)
Source: Int J Cancer. 2022 May 16;151(3):422–34. doi: 10.1002/ijc.34018 (PMC9321995; doi:10.1002/ijc.34018)
Supplement: Supplementary file 2 — Appendix S2Supporting Information. [file IJC-151-422-s001.zip › IJC_34018_STAMPEDE_Protocol_v19.0_Clean .pdf]

STAMPEDE

Systemic Therapy in Advancing or Metastatic Prostate Cancer: Evaluation of Drug Efficacy

A multi-arm multi-stage randomised controlled trial

Version: 19.0
Date: 01-June-2018

MRC CTU AT UCL ID: PR08
ISRCTN #: ISRCTN78818544
NCT #: NCT00268476
EUDRACT #: 2004-000193-31
CTA #: 00316/0026/001-0001
MREC #: 04/MRE07/35

Authorised by:

Name: Professor Nicholas D James
Role: Chief Investigator & Comparison CI for "Abiraterone comparison"
Signature:

Name: Matthew Sydes
Role: Trial Statistician
Signature:

Authorised by:

**Name:**	**Dr Chris Parker**
Role:	**CCI "M1|RT comparison"**
Signature:	
**Name:**	**Professor Gerthardt Attard**
Role:	**CCI "Enzalutamide & abiraterone comparison"**
Signature:	
**Name:**	**Professor Noel Clarke**
Role:	**Co-CCI "Metformin comparison"**
Signature:	
**Name:**	**Professor Silke Gillessen**
Role:	**Co-CCI "Metformin comparison"**
Signature:	
**Name:**	**Professor Ruth Langley**
Role:	**CCI "Transdermal oestradiol comparison"**
Signature:	

GENERAL INFORMATION

This document was constructed using the MRC CTU at UCL Protocol Template Version 4.0. It describes the STAMPEDE trial, coordinated by the Medical Research Council (MRC) Clinical Trials Unit (CTU) at University College London (UCL), and provides information about procedures for entering patients into it. The protocol should not be used as an aide-memoire or guide for the treatment of other patients. Every care has been taken in drafting this protocol, but corrections or amendments may be necessary. These will be circulated to the registered investigators in the trial, but sites entering patients for the first time are advised to contact the Cancer And Other Non-Infectious Diseases Group, MRC CTU at UCL, London, UK, to confirm they have the most up-to-date version.

COMPLIANCE

The trial will be conducted in compliance with the approved protocol, the Declaration of Helsinki 1996, the principles of Good Clinical Practice (GCP), Commission Directive 2005/28/EC with the implementation in national legislation in the UK by Statutory Instrument 2004/1031 and subsequent amendments, the UK Data Protection Act (DPA number: Z6364106), and the National Health Service (NHS) Research Governance Framework for Health and Social Care (RGF). International sites will comply with the principles of GCP as laid down by the ICH topic E6 (Note for Guidance on GCP), Commission Directive 2005/28/EC (the European Directive 2001/20/EC [where applicable]) and applicable national regulations.

SPONSOR

The MRC is the sponsor of STAMPEDE and MRC CTU has been delegated responsibility for the overall management of STAMPEDE. Queries relating to MRC sponsorship should be addressed to the Director, Professor Max Parmar, Institute of Clinical Trials & Methodology, MRC CTU at UCL, 2nd Floor, 90 High Holborn, London, WC1V 6LJ UK, or via the STAMPEDE Trial Team.

On 01-Aug-2013, the MRC CTU became part of University College London (UCL). The MRC maintains sponsorship for the trial however UCL is the legal entity responsible for the running of the trial. This responsibility is delegated to the coordinating trial unit, the MRC CTU at UCL.

FUNDING

Cancer Research UK's Clinical Research Committee (formerly the Clinical Trials Advisory Awards Committee), Medical Research Council, and educational grants from Novartis, Sanofi-Aventis, Pfizer, Janssen Pharma NV, Astellas, Clovis Oncology.

AUTHORISATIONS AND APPROVALS

The following persons are authorised to sign the final protocol and protocol amendments for the sponsor: Professor Nicholas James (Chief Investigator) and Matthew Sydes (Trial Statistician) and the Co-Chief-investigators for each comparison subsequently added, Dr Chris Parker, Professor Gerhardt Attard, Professor Noel Clarke, Professor Silke Gillessen and Professor Ruth Langley.

TRIAL REGISTRATION

This trial has been registered with the ClinicalTrials.gov Clinical Trials Register, where it is identified as NCT00268476.

RANDOMISATION
Call MRC CTU at UCL, Monday to Friday 0900-1700
Excluding public holidays or dates when notice has been given by the Unit.
Tel: +44 (0) 20 7670 4777

SAE REPORTING
Fax to 020 7670 4818 within 24 hours of becoming aware of the event
Or send via **encrypted** email to mrcctu.stampede@ucl.ac.uk

COORDINATING SITE

MRC Clinical Trials Unit at UCL<br>90 High Holborn<br>2nd Floor<br>London WC1V 6LJ<br>UK	Switchboard:	020 7670 4700
	Fax:	020 7670 4818
	Email:	mrcctu.stampede@ucl.ac.uk
	Website:	www.stampedetrial.org

MRC CTU AT UCL STAFF

Trial Manager:	Shabinah Ali	Tel:	0207 670 4758
Trial Manager:	Mazna Anjum	Tel:	0207 670 4772
Trial Manager:	Michelle Buckner	Tel:	0207 670 4840
Trial Manager:	Joanna Calvert	Tel:	0207 670 4916
Trial Manager	Dymphna Lee	Tel:	0207 670 4711
Trial Manager	Charlotte Tyson	Tel:	0207 670 4846
Trial Manager:	Christopher Wanstall	Tel:	0207 670 4882
Trial Manager:	Arlen Wilcox	Tel:	0207 670 4822

FOR ALL OTHER CONTACTS: http://www.stampedetrial.org/contact-us/trial-team/

SAAK COORDINATING CENTRE

Corinne Schär Bern, Switzerland

Chief Investigator

Prof Nicholas James

Clinical Oncology, The Medical School
University of Birmingham
Birmingham, B15 2TJ
UK

Tel: 0121 371 3615 (Secretary)

Email: n.d.james@bham.ac.uk

Co-investigators and TMG members

Prof Gerhardt Attard<br>Oncologist	London, UK	CCI "Enzalutamide+Abiraterone comparison"
Dr Simon Chowdhury<br>Oncologist	London, UK	
Prof Noel Clarke<br>Urologist	Manchester, UK	TMG Vice-Chair<br>Co-CCI "Metformin comparison"
Mr William Cross<br>Urologist	Leeds, UK	
Prof David Dearnaley<br>Oncologist	London, UK	
Dr Duncan Gilbert<br>Oncologist	London & Brighton, UK	
Prof Silke Gillessen<br>Oncologist	St Gallen, Switzerland	C-CCI "Metformin comparison"
Prof Rob Jones<br>Oncologist	Glasgow, UK	
Prof Ruth Langley<br>Oncologist	London & Brighton, UK	CCI "Transdermal oestradiol comparison"
Dr Zafar Malik<br>Oncologist	Liverpool, UK	
Dr Chris Parker<br>Oncologist	London, UK	CCI "M1|RT comparison"
Mr Alastair Ritchie<br>Surgeon	Edinburgh, UK	
Dr Martin Russell<br>Oncologist	Glasgow, UK	
Prof George Thalmann<br>Urologist	Bern, Switzerland	
Robin Millman<br>Patient representative	UK	
David Matheson,<br>Patient representative	UK	
Prof Mark Sculpher<br>Health Economics Advisor	York, UK	
Amanda Adler<br>Metformin adviser	Cambridge UK	

Trial Advisory Groups	Chairs
Biological Research Group	TBC<br>Prof David Waugh (Co-Chair)
Bone & Imaging Group	Prof Nicholas James
Clinical Safety Committee	Prof Noel Clarke
Comparison Management Groups	Each led by Comparison Chief Investigator(s)
Metabolic Translational Group	Prof Silke Gillessen
Outcome Review Group	Prof Robert Jones
Site Advisory Team	Mr William Cross
STRATOSPHere consortium	Prof Gerhardt Attard

CONTENTS

GENERAL INFORMATION 3

CONTENTS 7

SUMMARY OF TRIAL 12

ABBREVIATIONS & GLOSSARY 20

1 LAY SUMMARY 25

2 BACKGROUND 29
2.1 INTRODUCTION AND SETTING 29
2.1.1 Long-term Androgen Deprivation Therapy 29
2.1.2 Role Of Radiotherapy For People With M0 Disease 29
2.1.3 Role Of SOC docetaxel 30
2.1.4 Role of SOC abiraterone in addition to ADT 30
2.2 DESIGN 30
2.3 PREVIOUSLY-REPORTED RESEARCH TREATMENTS 31
2.4 COMPARISONS IN FOLLOW-UP 31
2.5 RATIONALE FOR RECRUITING COMPARISONS 31
2.5.1 Metformin 31
2.5.2 Transdermal Oestradiol 32

3 SELECTION OF INSTITUTIONS AND INVESTIGATORS 35
SITE/INVESTIGATOR CRITERIA 35
3.1.1 Principle Investigator's Qualifications & Agreements 35
3.1.2 Adequate Resources 36
3.2 COMPARISON-SPECIFIC SITE ACCREDITATION 36
3.2.1 Transdermal Oestradiol Comparison: arms A & L 36
3.3 REQUIRED TRIAL DOCUMENTATION 37

4 SELECTION OF PARTICIPANTS 38
4.1 IDENTIFYING POTENTIAL TRIAL PARTICIPANTS 38
4.2 APPROACH TO INFORMED CONSENT 38
4.2.1 Screening Investigations Prior To Randomisation 38
4.2.2 Baseline Investigations required for participants allocated to arms A, K, L 39
4.3 PRIOR PERMITTED SOC TREATMENTS 39
4.3.1 Hormone Treatment Prior To Randomisation 39
4.3.2 Standard-Of-Care (SOC) Radiotherapy 40
4.3.3 Standard-Of-Care (SOC) Docetaxel 40
4.3.4 Standard-Of-Care (SOC) Abiraterone 40
4.4 GENERAL INCLUSION CRITERIA 41
4.4.1 High-Risk Newly-Diagnosed Non-Metastatic Node-Negative Disease 41
4.4.2 Newly-Diagnosed Metastatic Or Node-Positive Disease 41
4.4.3 Previously Radically Treated, Now Relapsing (Prior Radical Surgery And/or Radiotherapy) ... 41
4.4.4 General inclusion criteria required for all participants 41
4.5 GENERAL EXCLUSION CRITERIA 42
4.6 COMPARISON-SPECIFIC ELIGIBILITY CRITERIA 43

4.6.1 Metformin Comparison (randomisation between arm A and arm K) 43
4.6.2 Transdermal Oestradiol Comparison (randomisation between arm A and arm L) 43
4.7 SUB-STUDY ELIGIBILITY CRITERIA 44
4.7.1 Eligibility for germline DNA sub-study (Saliva samples) 44
4.7.2 Eligibility for the circulating tumour DNA sub-study (sequential blood samples) 44
4.7.3 Eligibility for tumour sample analysis (FFPE blocks) 44
4.7.4 Eligibility for the Biomarker-Screening Pilot study 44

5 RANDOMISATION AND ENROLMENT 45
5.1 RANDOMISATION 45
5.2 CO-ENROLMENT GUIDELINES 45

6 TREATMENT OF PARTICIPANTS 46
6.1 STANDARD-OF-CARE (SOC) 46
6.1.1 Hormone Therapy 46
6.1.2 Standard-Of-Care (M0) Prostate RT 47
6.1.3 Standard-Of-Care Docetaxel 47
6.1.4 Standard-Of-Care Abiraterone 48
6.2 RESEARCH TREATMENTS 48
6.2.1 Required Timelines When Starting Research Treatment 48
6.2.2 Research Abiraterone + Prednisolone (relevant to arms G & J) 48
6.2.3 Research Abiraterone + Prednisolone: Administration And Management Of Toxicities 49
6.2.4 Research Enzalutamide + Abiraterone + Prednisolone (Arm J) 54
6.2.5 Enzalutamide: Dose Modification & Toxicity Management 55
6.2.6 Metformin: Administration, Dose Modifications And Management Of Toxicities 57
6.2.7 Research Transdermal Oestradiol (Arm L) 60
6.2.8 Transdermal Oestradiol: Administration, Dose Modifications And Management Of Toxicities62
6.3 CONCOMITANT TREATMENTS AND DRUG INTERACTIONS 62
6.3.2 Abiraterone: Interaction With Other Medicinal Products 63
6.3.3 Enzalutamide: Interaction With Other Medicinal Product 63
6.3.4 Metformin: Interaction With Medicinal Products And Other Forms Of Interaction 66
6.3.5 Transdermal Oestradiol: Drug Interactions 66
6.4 TRIAL PRODUCTS 67
6.5 TREATMENT DATA COLLECTION 67
6.6 MEASURES OF COMPLIANCE/ADHERENCE 67
6.7 ADMINISTRATION OF STANDARD RADIOTHERAPY TO M0 PARTICIPANTS 67
6.7.1 Treatment Details 67

7 ASSESSMENTS AND PROCEDURES 69
7.1 SCHEDULE FOR ASSESSMENTS 69
7.1.1 Treatment & Follow-up Schedules 69
7.1.2 PSA, Testosterone And Oestradiol Measurements 69
7.1.3 Assessment Of Treatment Failure (Definition Of Progression) 69
7.1.4 Additional Metabolic And Cardiovascular Outcomes 71
7.1.5 Additional Safety Assessments 72
7.2 DATA COLLECTION PROCEDURES 73
7.2.1 Data Collection For SOC Hormone Therapy 73
7.2.2 Data Collection For SOC Docetaxel 73
7.2.3 Data collection for SOC Abiraterone 73
7.2.4 Data Collection And Non-Administration Of Standard Radiotherapy 74
7.2.5 Data Collection For Palliative Radiotherapy 74
7.2.6 Data Collection for Research (M1) Radiotherapy 74

7.2.7 Data Collection for Additional Treatments Given for Disease Progression 74
7.3 FOLLOW-UP PROCEDURE 75
7.3.1 Follow-up Telephone Consultations 75
7.3.2 Follow-up Using Electronic Healthcare Records 75
7.4 COMPARISON CLOSURE 76
7.4.1 Comparisons for which Follow-up has Ceased 76

8 STOPPING OF TREATMENT OR FOLLOW-UP 80
8.1 STOPPING RESEARCH INTERVENTIONS 80
8.1.1 Stopping Research Treatment: Abiraterone, Enzalutamide + Abiraterone 80
8.1.2 Stopping Research Treatment: Metformin 80
8.1.3 Stopping Research Treatment: Transdermal Oestradiol 81
8.2 PARTICIPANT TRANSFERS 81
8.3 EARLY CESSATION OF TRIAL PARTICIPATION 81

9 STATISTICAL CONSIDERATIONS 83
9.1 METHOD OF RANDOMISATION 83
9.2 OUTCOME MEASURES 83
9.3 SAMPLE SIZE: PRINCIPLES 84
9.4 SAMPLE SIZE ISSUES & TRIAL STAGES: ADDITIONAL RESEARCH ARM H 86
9.4.1 Pilot Phase: Additional Research Arm H 86
9.4.2 Activity Stages I-III: Additional Research Arm H 86
9.4.3 Efficacy Stage IV: Additional Research Arm H 86
9.4.4 Sample Size For Additional Research Arm H 86
9.5 SAMPLE SIZE ISSUES & TRIAL STAGES: ADDITIONAL RESEARCH ARM J 88
9.5.1 Pilot Phase: Additional Research Arm J 88
9.5.2 Activity Stages I-II: Additional Research Arm J 88
9.5.3 Efficacy Stage III: Additional Research Arm J 88
9.5.4 Sample Size For Additional Research Arm J 88
9.5.5 Further Sample Size Issues For Additional Research Arm J 89
9.6 SAMPLE SIZE ISSUES & TRIAL STAGES: ADDITIONAL RESEARCH ARM K 89
9.6.1 Implementation: Additional Research Arm K 89
9.6.2 Outcome Measures: Additional Research Arm K 90
9.6.3 Pilot Phase: Additional Research Arm K 91
9.6.4 Activity Stage I: Additional Research Arm K 91
9.6.5 Efficacy Stage II: Additional Research Arm K 91
9.6.6 Sample Size For Additional Research Arm K 92
9.6.7 Further Sample Size Issues For Additional Research Arm K 92
9.7 SAMPLE SIZE ISSUES & TRIAL STAGES: ADDITIONAL RESEARCH ARM L 93
9.7.1 Implementation And Outcome Measures: Additional Research Arm L 93
9.7.2 Additional Use of Outcome Data from the "transdermal oestradiol comparison" 94
9.7.3 Definition of PFS and Use As Co-primary Outcome Measure: Additional Research Arm L 94
9.8 FURTHER NOTES ON TRIAL DESIGN 95
9.8.1 Overall Sample Size 95
9.8.2 Factorial Design 95
9.9 INTERIM MONITORING AND ANALYSES 95
9.10 OUTLINE ANALYSIS PLAN 96
9.10.1 Pilot / Safety Phases 96
9.10.2 Activity And Efficacy Stages 96

10 MONITORING AND QUALITY ASSURANCE 98
10.1 MONITORING AT CTU 98

10.1.1 Central monitoring of consent 98
10.2 DIRECT ACCESS TO DATA 98
10.3 VISITS TO INVESTIGATOR SITES 98
10.4 CONFIDENTIALITY 98

11 SAFETY REPORTING 100
11.1 SAFETY REPORTING DEFINITIONS 100
11.1.1 Adverse event definitions 101
11.1.2 Defining treatment for the purposes of safety reporting 101
11.2 SAFETY PROCESSES: TRIAL-SPECIFIC SAE REPORTING EXEMPTIONS 102
11.3 SITE INVESTIGATOR RESPONSIBILITIES 102
11.3.1 Notification period 102
11.3.2 Investigator Assessment 103
11.3.3 Event Follow-up 106
11.4 CTU RESPONSIBILITIES 106

12 ETHICAL CONSIDERATIONS AND APPROVAL 108
12.1 ETHICAL CONSIDERATIONS 108
12.2 ETHICAL APPROVAL 109

13 REGULATORY APPROVAL 111
13.1 DATA COLLECTION & RETENTION 111

14 INDEMNITY 112

15 FINANCE 113

16 TRIAL COMMITTEES 114
16.1 TRIAL MANAGEMENT GROUP (TMG) 114
16.2 TRIAL STEERING COMMITTEE (TSC) 114
16.3 INDEPENDENT DATA MONITORING COMMITTEE (IDMC) 114
16.4 TMG SUB-GROUPS AND EXPERT PANELS 115
16.5 MRC CTU AT UCL INTERNAL GROUPS 115

17 ANCILLARY STUDIES 117
17.1 PATIENT REPORTED OUTCOMES 117
17.1.1 Quality of life (QL) 117
17.1.2 Health Economics 117
17.2 TRANSLATIONAL SUB-STUDIES 119
17.2.1 Germline DNA Analysis 119
17.2.2 Circulating Tumour-DNA Analysis (Sequential Blood Samples) 119
17.2.3 Tissue Sample Analysis (FFPE Blocks) 119
17.2.4 Biomarker-Screening Pilot 120
17.2.5 Informed consent to receive results arising from genetic sub-studies 120
17.3 DISEASE VOLUMETRIC ANALYSIS SUB-STUDY 121
17.4 USING ROUTINE DATA TO IDENTIFY CLINICAL TRIAL OUTCOMES 121

18 PATIENT AND PUBLIC INVOLVEMENT 122
18.1 POTENTIAL IMPACT OF PPI 122
18.2 PATIENT REPRESENTATIVES 122

19 PUBLICATIONS 123

20 DATA AND/OR SAMPLE SHARING 124

21 PROTOCOL AMENDMENTS 125

21.1 PROTOCOL 125

21.1.1 Amendments Made To Protocol Version 1.0 (May-2004) 125
21.1.2 Amendments Made To Protocol Version 1.1 (May-2005) 125
21.1.3 Amendments Made To Protocol Version 2.0 (Jun-2005) 125
21.1.4 Amendments Made To Protocol Version 3.0 (Jul-2006) 126
21.1.5 Amendments Made To Protocol Version 4.0 (Dec-2007) 127
21.1.6 Amendments Made To Protocol Version 5.0 (Aug-2008) 127
21.1.7 Amendments Made To Protocol Version 6.0 (Jul-2009) 128
21.1.8 Amendments Made To Protocol Version 7.0 (Jul--2011) 128
21.1.9 Amendments Made To Protocol Version 7.1 (Jul-2011) 129
21.1.10 Amendments Made To Protocol Version 8.0 (Sep-2011) 130
21.1.11 Amendments Made To Protocol Version 9.0 (Oct-2012) 131
21.1.12 Amendments Made To Protocol Version 10.0 (Apr-2013) 131
21.1.13 Amendments Made To Protocol Version 11.0 (Sep-2013) 132
21.1.14 Amendments Made To Protocol Version 12.0 (Jan-2014) 132
21.1.15 Amendments Made To Protocol Version 13.0 (Feb-2015) 132
21.1.16 Amendments Made To Protocol Version 14.0 (Oct-2015) 133
21.1.17 Amendments Made To Protocol Version 15.0 (Mar-2017) 134
21.1.18 Amendments Made To Protocol Version 16.0 (Oct-2017) 134
21.1.19 Amendments Made To Protocol Version 17.0 (Feb-2017) 135
21.1.20 Amendments made to Protocol Version 18.0 (Jun-2018) 136

22 REFERENCES 138

SUMMARY OF TRIAL

SUMMARY INFORMATION TYPE	SUMMARY DETAILS
Acronym	STAMPEDE
Long Title of Trial	Systemic Therapy in Advancing or Metastatic Prostate Cancer: Evaluation of Drug Efficacy: A multi-arm multi-stage randomised controlled trial
Version	19.0 (in progress)
Date	01-Jun-2018
MRC CTU at UCL ID	PR08
NCT #	NCT00268476
EudraCT #	2004-000193-31
Study Design	Multi-arm multi-stage platform randomised controlled trial
Type of Participants to be Studied	People starting long-term hormone therapy for metastatic or high-risk non-metastatic prostate cancer
Setting	Tertiary care
Interventions to be Compared	Various - see comparison-specific tables
Study Hypothesis	Various - see comparison-specific tables
Definitive Primary Outcome Measure	Overall survival (unless stated)
Intermediate Primary Outcome Measure	Failure-free survival (unless stated)
Secondary Outcome Measure(s)	Toxicity<br>Symptomatic skeletal events<br>Quality-of-life<br>Cost-effectiveness
Randomisation	Minimisation using a random element across a number of stratification factors
Number of Participants	See comparison-specific tables
Duration	See comparison-specific tables
Sponsor	Medical Research Council
Funders	Cancer Research UK<br>Medical Research Council<br>Astellas<br>Clovis Oncology<br>Janssen<br>Novartis<br>Pfizer<br>Sanofi-Aventis

SUMMARY INFORMATION TYPE	SUMMARY DETAILS
**"Original comparisons"**	
Type of Participants to be Studied	People starting long-term hormone therapy for metastatic or high-risk non-metastatic prostate cancer
Control Arm	• Arm A: Standard-of-care (SOC)<br>• Androgen-deprivation therapy (ADT)
Interventions to be Compared	• Arm B: SOC + zoledronic acid<br>• Arm C: SOC + docetaxel<br>• Arm D: SOC + celecoxib<br>• Arm E: SOC + zoledronic acid + docetaxel<br>• Arm F: SOC + zoledronic acid + celecoxib
Allocation ratio	• 2 control arm : 1 research arm [2A:1B:1C:1D:1E:1F]
Study Hypothesis	Research interventions will improve survival over SOC
Definitive Primary Outcome Measure	Overall survival
Intermediate Primary Outcome Measure	Failure-free survival
Status	Primary results published and **active follow-up to be discontinued** Summer 2018 (1, 2)
**"Abiraterone comparison"**	
Type of Participants to be Studied	People starting long-term hormone therapy for metastatic or high-risk non-metastatic prostate cancer
Control arm	Arm A: Standard-of-care (SOC) +/- RT
Intervention to be Compared	• Arm G: SOC + abiraterone
Allocation ratio	• 1 control arm : 1 research arm [1A:1G]
Study Hypothesis	Addition of abiraterone to SOC will improve survival over SOC alone
Definitive Primary Outcome Measure	Overall survival
Intermediate Primary Outcome Measure	Failure-free survival
Number of Participants	Around 1,800 patients for 267 control arm definitive primary outcome measure events
Duration	6 to 8 years
Status	Primary results published (3), remains on active follow-up to permit a further longer-term analysis
**"M1|RT comparison"**	
Type of Participants to be Studied	People starting long-term hormone therapy for newly-diagnosed metastatic prostate cancer with no contraindication to prostate radiotherapy
Control arm	• Arm A: Standard-of-care (SOC)
Intervention to be Compared	• Arm H: SOC + radiotherapy to the prostate (RT)
Allocation ratio	• 1 control arm : 1 research arm [1A:1H]
Study Hypothesis	Addition of RT to SOC will improve survival over SOC alone

**SUMMARY INFORMATION TYPE**	**SUMMARY DETAILS**
Definitive Primary Outcome Measure	Overall survival
Intermediate Primary Outcome Measure	Failure-free survival
Number of Participants	Around 1,800 patients for 267 control arm definitive primary outcome measure events
Duration	6 to 8 years
Status	In follow-up; results of primary analysis expected in 2018
**"Enzalutamide + abiraterone comparison"**	
Type of Participants	People starting long-term hormone therapy for metastatic or high-risk non-metastatic prostate cancer
Control Arm	Arm A: Standard-of-care (SOC) +/- docetaxel
Interventions to be Compared	• Arm J: SOC + enzalutamide + abiraterone
Allocation ratio	• 1 control arm : 1 research arm [1A:1J]
Study Hypothesis	Addition of enzalutamide, in combination with abiraterone, to SOC will improve survival over SOC alone
Definitive Primary Outcome Measure	Overall survival
Intermediate Primary Outcome Measure	Failure-free survival
Number of Participants	Around 1,800 patients for 267 control arm definitive primary outcome measure events
Duration	6 to 8 years
Status	In follow-up
**"Metformin comparison"**	
Type of Participants to be Studied	Non-diabetic people, with no contraindication to metformin, starting long-term hormone therapy for metastatic or high-risk non-metastatic prostate cancer
Control arm	• Arm A: Standard-of-care (SOC)
Intervention to be Compared	• Arm K: SOC + metformin
Allocation ratio	• 1 control arm : 1 research arm [1A:1K]
Study Hypothesis	Addition of metformin to SOC will improve survival over SOC alone
Definitive Primary Outcome Measure	Overall survival
Intermediate Primary Outcome Measure	Overall survival
Number of Participants	Around 2800 patients, including around 1,700 M1 (metastatic) patients, for 473 control arm definitive primary outcome measure events among M1 patients
Duration	7 years
Status	Recruiting

SUMMARY INFORMATION TYPE	SUMMARY DETAILS
**"Transdermal oestradiol comparison"**	
Type of Participants to be Studied	People starting long-term hormone therapy for metastatic or high-risk non-metastatic prostate cancer, having had no more than one 4-week (or one-month) LHRH (Luteinizing hormone releasing hormone) injection & 8 weeks of anti-androgens
Control arm	Arm A: Standard-of-care (SOC)
Intervention to be Compared	• Arm L: Transdermal oestradiol ± RT ± docetaxel
Allocation ratio	• 1 control arm : 1 research arm [1A:1L]
Study Hypothesis	Transdermal oestradiol will be non-inferior to standard hormone therapy, while having fewer side-effects and improved quality-of-life
Definitive Primary Outcome Measures	Co-primary endpoints of progression-free survival and overall survival
Intermediate Primary Outcome Measure	Progression-free survival
Number of Participants	Around 500 to include within a meta-analysis with the PATCH trial, which will include around 2,000 patients overall
Duration	4 to 6 years
Status	Recruiting

Figure 1: Randomisation schema from protocol v19.0 onwards

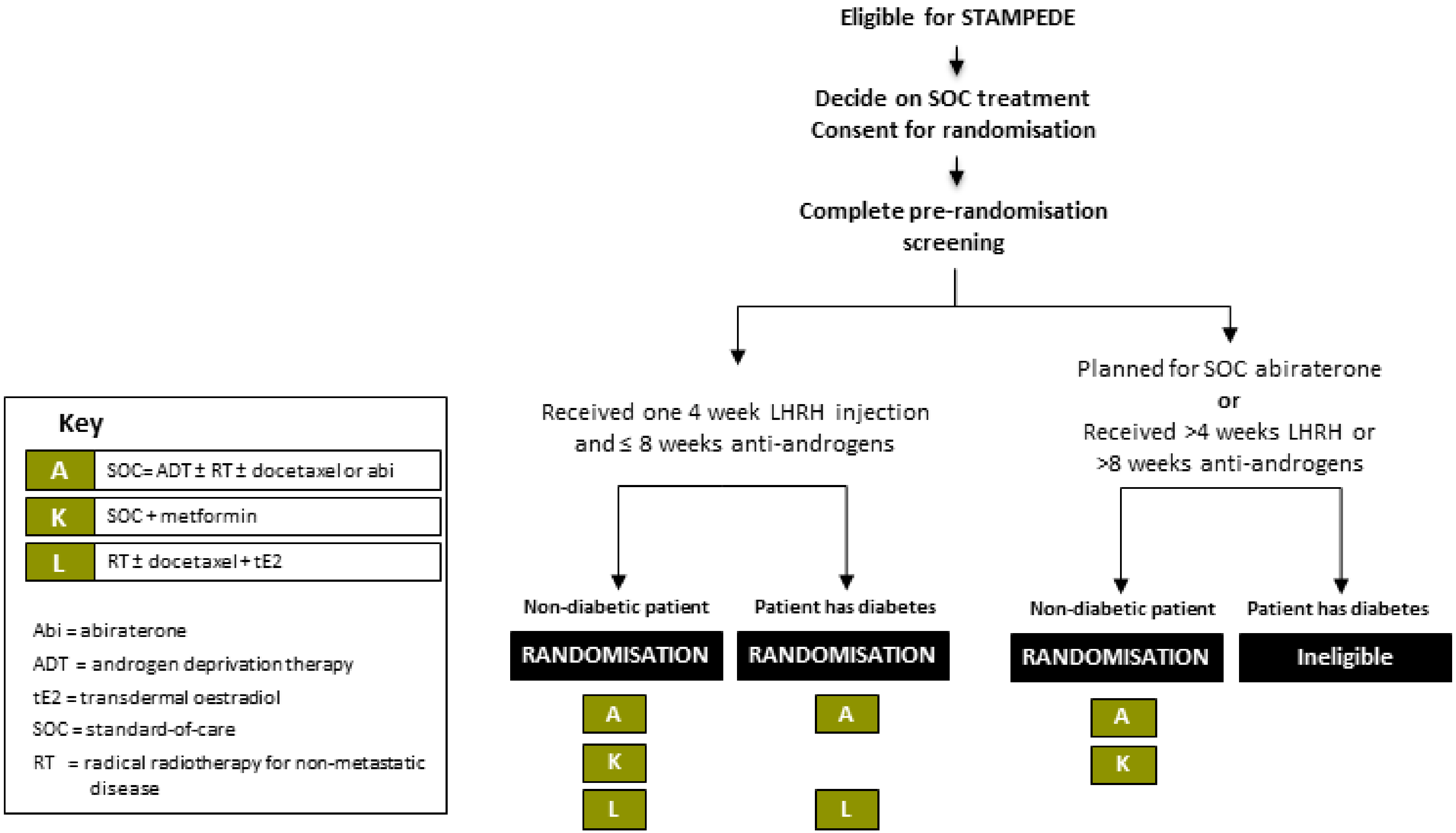

Figure 2: Arms of the STAMPEDE trial open to recruitment over time

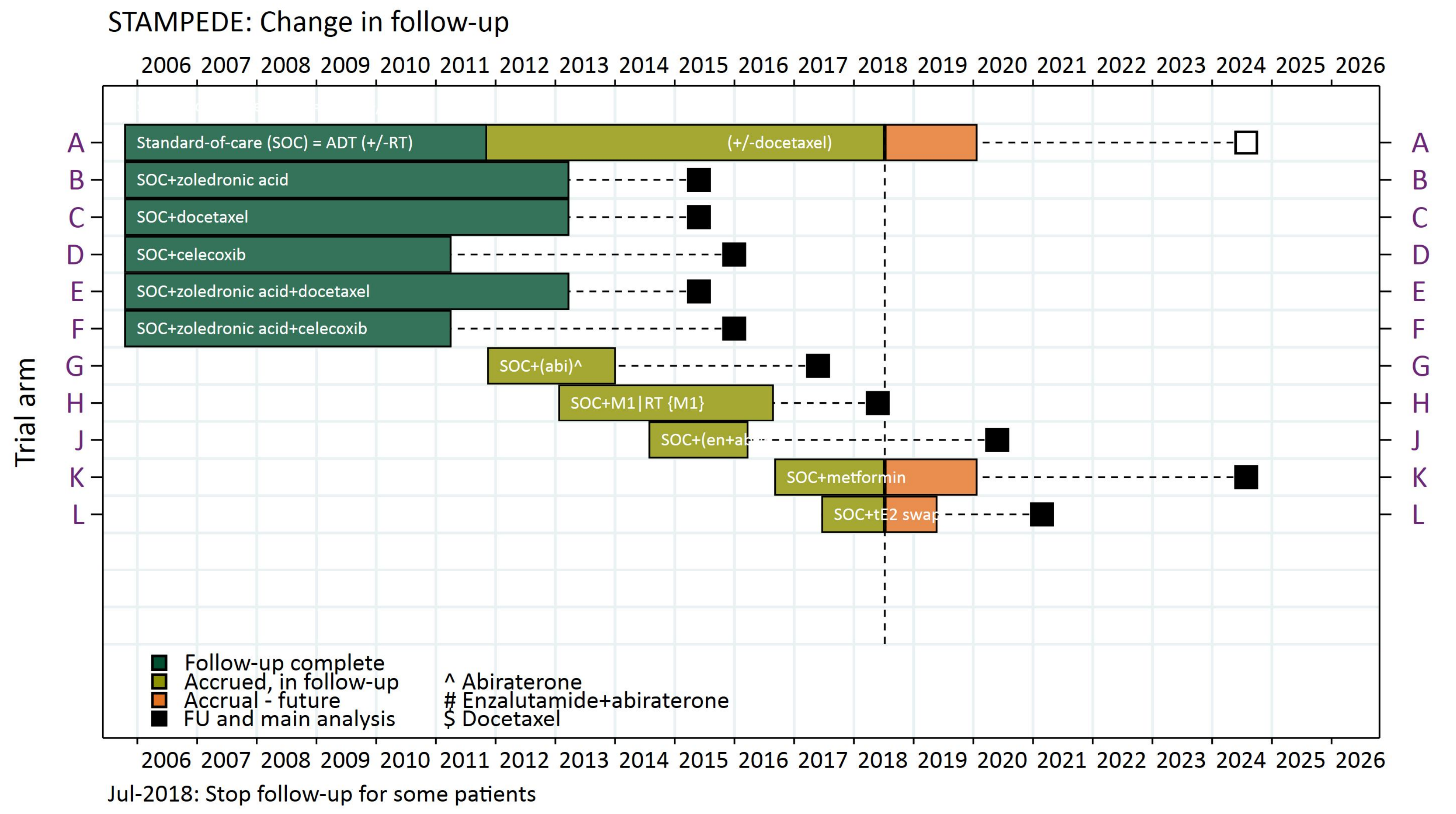

Table 1: Schedule of Assessments for Participants Randomised before 05-Sep-2016

	ASSESSMENT WEEK											ALL FURTHER VISITS [1]	AT EACH DISEASE EVENT [2]	END OF TRT	PRIOR TO 2ND LINE TRT
	4-6	12	18	24	36	48	60	72	84	96	104				
**Arm A/G/J**															
Blood collection cell-free DNA Streck™ tubes [3]						X [4]		X [4]	X [4]				X	X	X
Saliva sample [3]	Any time point														
FFPE block [3]	Once, at the point of request														
PSA	X	X	X	X	X	X	X	X	X	X	X	X			
Waist circumference + Weight	X	X	X	X	X	X	X	X	X	X	X	X			
Concomitant medication	X	X	X	X	X	X	X	X	X	X	X	X			
Height															
QL + HE [5,3]	X	X	X	X	X	X	X	X	X	X	X	X			
**Arm G&J only**															
Blood pressure [6]	X	X	X	X	X	X	X	X	X	X	X	X			
Safety bloods (LFTs and potassium) [6]	X	X	X	X	X	X	X	X	X	X	X	X			

[1] Follow-up visits after year 2 need to be carried out every 6 months for the first 5 years. At year 6 and onwards visits should be every 12 months whilst active follow-up continues.
[2] Disease events are defined as each type of disease progression: PSA (biochemical), clinical (symptomatic) and radiological (objective).
[3] Only if participating in relevant sub-study, for information regarding samples see the **Sample Collection & Handling Manual** available via the STAMPEDE website for details
[4] Sample only required for participants with metastatic disease at trial entry (M1)
[5] Review **Table 33** for a breakdown of participants that are still required to complete Quality-of-life (QL) + health economic (HE) questionnaires
[6] For participants receiving research abiraterone, BP, liver function tests (LFTs) and serum potassium monitoring is required 2-weekly in the first 12 weeks, then monthly until 12 months on treatment. For participants who have not experienced toxicity following 12 months of treatment, this may be reduced to every 2 months whilst research abiraterone continues. Increased monitoring is required in participants experiencing toxicity; see **Table 9, Table 10** and **Table 11** for details.

Table 2: Schedule of Assessments for Participants Randomised on or after 05-Sep-2016

	Pre-Rand[n]	ASSESSMENT WEEK											ALL FURTHER VISITS [1]	AT EACH DISEASE EVENT [2]	END OF TRT	PRIOR TO 2ND LINE TRT
		4-6	12	18	24	36	48	60	72	84	96	104				
**Arms A/K/L**																
Cardiac (ECG BP)	X															
Screening bloods[3]	X															
Full radiological screening[4]	X															
WHO PS	X															
Blood collection cell-free DNA Streck™ tubes[5]	X						X[3]		X[3]	X[3]				X	X	X
Saliva sample[5]	Any time point															
FFPE block[5]	At the point of request															
Waist circumference	X	X	X	X	X	X	X	X	X	X	X	X	X			
Weight	X	X	X	X	X	X	X	X	X	X	X	X	X			
Height	X															
QL & HE[5, 6]	X	X	X	X	X	X	X	X	X	X	X	X	X			
HbA1c & Lipid profile	X				X[7]		X[7]					X[7]	X[7]			
Fasting Glucose & Triglycerides	X				X[7]		X[7]					X[7]	X[7]			
PSA	X[8]	X	X	X	X	X	X	X	X	X	X	X	X			
Concomitant medication	X	X	X	X	X	X	X	X	X	X	X	X	X			
**Arm K only**																
Safety bloods (eGFR) [9]					X		X		X		X		X			
**Arm L only**																
Testosterone & Oestradiol [10]		X[11]	X		X		X		X		X		X			

[1] Follow-up visits after year 2 need to be carried out every 6 months for the first 5 years. At year 6 and onwards visits should be every 12 months whilst active follow-up continues.
[2] Disease events are defined as each type of disease progression: PSA (biochemical), clinical (symptomatic) and radiological (objective).
[3] U&Es, LFTs, Serum creatinine and FBCs to be completed before randomisation. Cholesterol and albumin within 4 weeks before or after randomisation.
[4] Pre-randomisation imaging must be representative of current disease status, see **section 4.2.1**
[5] Only if participating in relevant sub-study, for information regarding samples see the **Sample Collection & Handling Manual** available via the STAMPEDE website for details
[6] Review **Table 33** for a breakdown of participants that are still required to complete Quality-of-life (QL) + health economic (HE) questionnaires
[7] If missed, samples can be obtained +/-12 weeks of the scheduled FU visit, maintaining 10-12 weeks in between the tests due at week 24 and 48 weeks.
[8] Pre-ADT PSA must have been obtained within 6 months prior to randomisation and ideally another PSA analysis should be completed within 2 weeks of randomisation.
[9] Increased monitoring of renal function required if renal function declines see **Section 6.2.6**. To continue until metformin permanently stopped.
[10] Hormone tests are required whilst the participant is receiving research transdermal oestradiol. Note that additional tests may be necessary as detailed in **Section 6.2.8**.
[11] First hormone tests for patients receiving research transdermal oestradiol should be at 4 weeks

ABBREVIATIONS & GLOSSARY

ABBREVIATION	EXPANSION
AA	Anti-androgen
AAH	Amalgamated Anthracite Holdings
ACE	Angiotensin-Converting Enzyme
ACTH	Adrenocorticotropic hormone
ADT	Androgen deprivation therapy
AE	Adverse Event
AR	Androgen receptor
AS	Activity Stage
AUC	Area under the plasma concentration–time curve
BID	Twice a day (bis in die)
BP	Blood pressure
BRCA2	BReast CAncer gene 2
BRG	Biological Research Group
BSA	Body surface area
CCI	Comparison Chief Investigator
CF	Consent Form
CI	Confidence interval
Co-CCI	Comparison Co-Chief Investigator
Cox-2	Cyclooxygenase 2
CRF	Case Report Form
CRN	Clinical Research Network
CRUK	Cancer Research UK
CRPC	Castrate-Resistant Prostate Cancer
CT	Computerised tomography
CTA	Clinical Trials Authorisation
CTAAC	Clinical Trials Advisory and Awards Committee
ctDNA	Circulating tumour DNA
CTC	Common Toxicity Criteria
CTU	Clinical Trials Unit
CTV	Clinical Tumour Volume
CVS	Cardiovascular
CXR	Chest X-ray

ABBREVIATION	EXPANSION
DAB	Dual Androgen Blockade
DHT	Dihydrotestosterone
DNA	Deoxyribonucleic Acid
DPA	Data Protection Act
ES	Efficacy Stage
IB	Investigator Brochure
ICH	International Conference on Harmonization
ECG	Electro cardiogram
FBC	Full Blood Count
FFS	Failure-Free Survival
FFPE	Formalin Fixed Paraffin Embedded
GCP	Good Clinical Practice
GFR	Glomerular Filtration Rate
GP	General Practitioner
HbA1c	Glycated haemaglobin
Hb	Haemoglobin
HE	Health Economics
HES	Hospital Episode Statistics
Hr	Hour
HR	Hazard Ratio
HSCIC	Health & Social Care Information Centre
HT	Hormone Therapy
IDMC	Independent Data Monitoring Committee
IM	Intramuscular
IMRT	Intensity Modulated Radiation Therapy
INR	International Normalized Ratio
IR	Immediate-Release
ISRCTN	International Standard Randomised Controlled Trial Number
IU	International Units
IV	Intravenous
LFTs	Liver Function Tests
LHRH	Luteinising Hormone Releasing Hormone
LREC	Local Research Ethics Committee
m	Month
mcg	Microgram

ABBREVIATION	EXPANSION
MHRA	Medicine and Healthcare Products Regulatory Agency
min	Minutes
MRC	Medical Research Council
MREC	Multi-Centre Research Ethics Committee
MRI	Magnetic resonance imaging
mTOR	Mammalian Target of Rapamycin
M0	Non-metastatic
M1	Metastatic
NCI	National Cancer Institute (USA)
NCRAS	National Cancer Registration and Analysis Service
NHS	National Health Service
N0	Node-negative
N+	Node-positive
NSAID	Non-Steroidal Anti-inflammatory Drugs
NYHA	New York Heart Association
OD	Once per day (omne in die)
ONS	Office for National Statistics
OS	Overall Survival
PATCH	Prostate Adenocarcinoma: TransCutaneous Hormones
PFS	Progression-free survival
PHE	Public Health England
PI	Principal Investigator
PIS	Patient Information Sheet
po	Orally (per orum)
PSA	Prostate Specific Antigen
pts	Patients
PTV	Planned Tumour Volume
QALY	Quality-adjusted Life Years
qds	Four times each day (quater die sumendus)
QL	Quality-of-life
RSI	Reference Safety Information
RTDS	National Radiotherapy Dataset
R&D	Research and Development
SACT	Systemic Anti-Cancer Therapy Dataset
SAE	Serious Adverse Event

ABBREVIATION	EXPANSION
SAR	Serious Adverse Reaction
sc	Under skin (sub-cutaneous)
SmPC	Summary of Product Characteristics
SOC	Standard-of-Care
SR	Sustained-Release
SSA	Site Specific Assessment
STAMPEDE	Systemic Therapy in Advancing and Metastatic Prostate Cancer: Evaluation of Drug Efficacy
SUSAR	Suspected Unexpected Serious Adverse Reactions
SWOG	South West Oncology Group
tE2	Transdermal Oestradiol
TMG	Trial Management Group
TMT	Trial Management Team
TEAE	Treatment-emergent adverse event
TURP	Trans-Urethral Resection of Prostate
TSC	Trial Steering Committee
T2DM	Type 2 Diabetes Mellitus
UCL	University College London
ULN	Upper Limit of Normal
U+E	Urea and Electrolytes
WHO	World Health Organisation

TERM	DEFINITION
ADT	Androgen deprivation therapy given in the form of LHRH agonists/antagonists (abbreviated to LHRH) or alternatively, transdermal oestradiol.
Anti-androgens	Refers to 1st generation oral androgen receptor blockers including bicalutamide, flutamide and cyproterone. Please note that the use of cyproterone will impact on comparison-specific eligibility.
Comparison	In STAMPEDE many research treatments are evaluated and compared with participants receiving the current protocol standard-of-care. The term comparison describes the participants who have been randomised to receive research treatment and their comparable controls, each comparison is named by the research treatment e.g. the “metformin comparison” refers to all participants in arm K and the comparable non-diabetic patients contemporaneously randomised to arm A.
Hormone Therapy	Refers to all forms of hormone therapy given in the first line setting and includes LHRH, anti-androgens, transdermal oestradiol, GnRH agonists and antagonists. This term does not include novel AR-targeted agents such as abiraterone or enzalutamide.
PSA nadir	For trial purposes, this refers to the lowest PSA value detected between randomisation and week 24 on trial. This is used to derive the PSA progression value.
Protocol research treatment	Additional treatments participants allocated to research arms receive as part of the STAMPEDE protocol e.g. metformin for participants allocated to arm K, or alternative in the case of transdermal oestradiol.
Protocol standard-of-care (SOC) treatment	Standard forms of background treatment permitted as part of the STAMPEDE protocol which include licenced ADT (e.g. LHRH analogues) given in the setting of hormone-naïve prostate cancer and first-line use of docetaxel or abiraterone.
Non-protocol treatments	All prostate cancer treatments given following disease progression in the management of CRPC.
Prednisolone	In Swiss sites this may be referred to as prednisone.

1 LAY SUMMARY

STAMPEDE is a large clinical trial that aims to assess new treatment approaches for people affected by high-risk prostate cancer. The trial has been open since 2005 and has tested many different ways of treating prostate cancer and some results are now already known. Each new or alternative treatment is compared with the current standard approach, referred to as a “comparison”. More than 10,000 people have joined STAMPEDE so far with answers becoming available throughout the trial as information on life expectancy and disease control rates are gathered and compared.

New participants joining the trial from protocol v19.0 onwards may be eligible to join one of two treatment comparisons:

- The “metformin comparison” made between the control arm (arm A) who receive standard treatment only and the metformin treatment group (arm K) who receive standard treatment and metformin.

- The “transdermal oestradiol comparison” made between the control arm (arm A) and the transdermal oestradiol treatment group (arm L) who receive transdermal oestradiol as an alternative form of standard hormone treatment.

Eligibility for each treatment group is dependent on several factors including the stage of prostate cancer, whether it has spread to involve other parts of the body (metastatic), and how long a patient has received hormone therapy prior to joining STAMPEDE. A computer program will be used to randomly allocate participants between all treatment groups for which they are eligible. **Table 3** summarises which treatment arms are currently open to recruitment.

All trial participants are asked whether they would like to join some of the sub-studies being run alongside the trial. These aim to address several additional research questions such as what effect each treatment has on quality-of-life(QL), and which provides the greater value for money for the health service. Some sub-studies are focused on improving our understanding of the biology of prostate cancer. For example, can genetic changes be identified in prostate cancer cells that could predict which treatments might work best and may explain why some treatments stop working?

Table 3: Summary of treatment groups currently open to recruitment (Protocol version 19.0)

TREATMENT BEING TESTED	TREATMENT GROUP	SUMMARY	FROM PROTOCOL VERSION
Metformin	Arm K	This anti-diabetic medication is proposed to have anti-cancer effects and may help prevent the side-effects of long-term ADT. STAMPEDE will investigate whether adding metformin to the current standard-of-care for non-diabetic people can improve life expectancy.	15.0
Transdermal oestradiol	Arm L	This is a form of hormone treatment which can suppress testosterone as effectively as standard forms of androgen-deprivation therapy (ADT) and has been shown to avoid some of the side-effects. For example, treatment with transdermal oestradiol does not appear to cause the bone to thin, a common problem with standard forms of ADT which might lead to the bones becoming fragile (osteoporosis) and more likely to break. It may also help to avoid some of the side-effects and therefore improve overall quality of life compared with standard forms of ADT. STAMPEDE will investigate whether transdermal oestradiol can treat prostate cancer as well as current standard forms of ADT. Transdermal oestradiol is currently being tested in another large clinical trial called PATCH which already has over 1,400 men participating.	16.0

Further results are expected in the next few years from other treatments tested in STAMPEDE, which have completed recruitment, summarised in **Table 4.** These include treatments currently used in different settings, including abiraterone and enzalutamide, both currently used when hormone treatment is no longer effective and the cancer has started to grow again, termed castrate-resistant prostate cancer (CRPC). As well as prostate radiotherapy, which is a treatment used in localised prostate cancer, tested in STAMPEDE as an additional treatment for people with cancer that has spread to other parts of the body (metastatic prostate cancer).

Table 4: Summary of treatment groups closed to recruitment; results awaited but follow-up ongoing

Treatment being tested	Treatment group	Summary	From Protocol version
Prostate radiotherapy	Arm H	This is treatment with high-energy x-rays targeted to the prostate gland. This treatment is now mandatory within STAMPEDE for participants with cancer that is confined to the prostate gland as large trials have shown it improves life expectancy. We are not certain whether we should give radiotherapy to the prostate if the cancer has already spread and so we are investigating this in STAMPEDE.	9.0
Enzalutamide (given with abiraterone)	Arm J	Enzalutamide is another novel hormone treatment, similar to abiraterone, which is also used in advanced prostate cancer, when standard hormone therapy has stopped working. Enzalutamide works by blocking androgen receptors and this may complement abiraterone. STAMPEDE is testing whether this treatment combination is a more effective way of controlling prostate cancer growth for longer and improving life expectancy.	12.0

Abiraterone was tested alone in arm G and the primary results of this comparison have been presented. Follow-up is ongoing as a further longer term analysis is planned.

Table 5: Summary of treatment group for which primary results reported but follow-up ongoing

Treatment tested	Treatment group	Summary of rationale and results	Protocol version Added
Abiraterone	Arm G	This is a novel hormone treatment which works by inhibiting steroid hormone synthesis so blocks prostate cancer cells from generating their own male hormones. This is thought to be a major way in which prostate cancer cells resume growth following anti-hormonal therapies. The results of STAMPEDE have shown that the addition of abiraterone with prednisone improves life expectancy and disease control or relapse rates when used earlier, for people with locally-advanced or metastatic disease.	8.0

In the past STAMPEDE also tested whether adding docetaxel chemotherapy, zoledronic acid, or celecoxib, alone or in combination, was beneficial in controlling prostate cancer growth and improving life expectancy. Recruitment has been completed to all of these original treatment groups, the results have been presented and it is no longer necessary to provide follow-up information relating to participants allocated to these comparisons, see **Table 6**.

For further information relevant to these treatment groups, refer to the STAMPEDE website where you can see earlier versions of the protocol and find summaries of the results and links to the scientific publications, www.stampedetrial.org.

Table 6: Summary of treatment groups reported and no longer on active follow-up

TREATMENT TESTED	TREATMENT GROUP	SUMMARY OF RATIONALE AND RESULTS	PROTOCOL VERSION ADDED
Zoledronic acid	Arm B	Prostate cancer cells can spread to bones and weaken them. Zoledronic acid is a drug that reduces bone destruction and hardens bones.<br>The results of STAMPEDE show that the addition of zoledronic acid alone does not prolong life expectancy. These results were comparable with data from other similar trials.	1.0
Docetaxel	Arm C	Docetaxel is a type of chemotherapy which can stop cells replicating. It has been used to treat advanced prostate cancer for some time, and is also used in e.g. the treatment of lung, breast and ovarian cancer.<br>The results of STAMPEDE show that the addition of docetaxel to hormone treatment does improve life expectancy, most markedly in people with metastatic disease, and delays time to progression or relapse for people with locally-advanced and metastatic disease.<br>The results of STAMPEDE were combined with other similar trials testing docetaxel and together, the results support this effect.<br>Docetaxel may now be given as part of standard treatment to all suitable people entering STAMPEDE (from protocol v14.0).	1.0
Celecoxib	Arm D	Celecoxib is an aspirin-like drug that is used to treat arthritis. It slows down the growth of cancer cells in the laboratory. STAMPEDE tested whether the addition of celecoxib could delay the growth of prostate cancer cells. Recruitment stopped early as an earlier analysis failed to demonstrate sufficient benefit. The final results were presented at GU ASCO 2016, a major international congress, and show that alone, celecoxib does not improve life expectancy.	1.0

Note that the combination of docetaxel and zoledronic acid was assessed in Arm E and, whilst beneficial overall, did not provide additional benefit over docetaxel. The combination of celecoxib and zoledronic acid was assessed in Arm F. No benefit was seen overall, however an improvement in life-expectancy was observed in the group of participants who had metastatic disease at trial entry who received both celecoxib and zoledronic acid (4).

2 BACKGROUND

2.1 INTRODUCTION AND SETTING

Prostate cancer is a major health problem world-wide and accounts for nearly one fifth of all newly-diagnosed male cancers. In the UK, approximately 47,150 people were diagnosed with prostate cancer in 2015 and over 11,000 people died from the disease (5).

2.1.1 Long-term Androgen Deprivation Therapy

The initial (first-line) treatment for locally-advanced or metastatic prostate cancer is based on androgen deprivation therapy (ADT) achieved either surgically with bilateral orchidectomy, or medically with LHRH agonists or antagonists (7). Long-term use of oral anti-androgens is permitted only when given with LHRH agonists, to achieve dual androgen blockade (previously termed maximum androgen blockade - MAB).

When used alone ADT produces initial responses in up to 95% of patients but is rarely curative. STAMPEDE aims to improve outcomes for people affected by high-risk prostate cancer by testing if additional treatments added to ADT can improve disease control and life-expectancy. Data from the control arm in STAMPEDE has shown that for people with newly-diagnosed metastatic disease, treated with ADT alone, the time to progression is just 11 months (7). Such progressive disease is referred to as castrate-resistant prostate cancer (CRPC).

Another important issue with ADT is the numerous associated side-effects, particularly with prolonged use. Since patients continue on LHRH after disease progression (with additional agents added), many people remain on treatment for a decade or longer. STAMPEDE is evaluating alternative forms of ADT and additional treatment with metformin aiming to mitigate some of the adverse effects of ADT which include osteoporosis (leading to an increased risk of fracture), adverse metabolic disturbance, cognitive decline, sexual dysfunction, hot flushes, physical deterioration and fatigue.

2.1.2 Role Of Radiotherapy For People With M0 Disease

Two randomised trials, SPCG7 (8) and NCIC PR.3 / MRC PR07 (9-11) have tested the question of whether ADT alone combined with radiotherapy is the best treatment for patients with high-risk localised prostate cancer (N0M0). Both trials demonstrated an improvement in overall and disease specific survival from the addition of radiotherapy to ADT. The size of this overall survival benefit is substantial (hazard ratio 0.68 in SPCG7 and 0.77 in PR07). As these two mature, large, well-conducted randomised trials have demonstrated benefit, we now mandate that radiotherapy be standard for patients with N0M0 disease (i.e. no nodal or metastatic spread). Patients with node-negative M0 prostate cancer will only be allowed to enter the trial if standard radiotherapy is planned. Any patients with N0M0 disease for whom radiotherapy is contra-indicated should be discussed with the STAMPEDE team prior to inclusion. For patients with node-positive, M0 disease there are no randomised data on whether radiotherapy is indicated or not. However the NCIC PR.3 / MRC PR07 trial included patients with unknown nodal status who received whole pelvic radiotherapy (12) and demonstrated a large overall benefit. Additionally, non-randomised data from the STAMPEDE control arm suggests that the benefit observed in patients with N0M0 disease can be extended to those with pelvic nodal involvement. Therefore the STAMPEDE TMG recommends that pelvic nodal radiotherapy be considered for patients with node-positive, M0 disease at the discretion of the treating clinician (13).

2.1.3 Role Of SOC docetaxel

The primary analysis of the "original comparisons" has shown docetaxel to significantly prolong survival (HR 0.78; 95% CI 0.66-0.93)(1). This is in support of the results of the CHAARTED trial which showed docetaxel improved survival in people with metastatic disease (14, 15). There was no evidence of heterogeneity in STAMPEDE in the treatment effect across any patient subgroups and median survival was improved by 10 months, from 71 to 81 months. In a well powered and pre-planned sub-group analysis of people with metastatic disease at randomisation the treatment effect was most apparent with a median survival benefit of 15 months. As a result the STAMPEDE TMG recommends that docetaxel should be strongly considered in all people with metastatic disease at presentation who are commencing ADT for the first time and are fit enough to receive chemotherapy.

Survival data for people without metastases at diagnosis is less mature but a statistically significant improvement in failure-free survival is seen, therefore, docetaxel may also be considered for people with high-risk non-metastatic disease who are commencing ADT for the first time and are fit enough to receive chemotherapy. Therefore, docetaxel is now permitted as part of the standard-of-care for all people entering STAMPEDE at the discretion of the treating clinician and patient.

2.1.4 Role of SOC abiraterone in addition to ADT

The primary analysis of the “abiraterone comparison” has shown abiraterone to improve survival in this setting (HR 0.63; 0.52-0.76; p<0.001)(3). The results are consistent with the co-published LATITUDE trial, which recruited an overlapping subset of the population eligible for STAMPEDE i.e. newly-diagnosed high-risk metastatic patients (16). The STAMPEDE TMG recommends that, where available, the addition of abiraterone to ADT may be considered on the basis of this evidence of benefit. In the absence of data supporting concurrent or sequential use in the absence of disease progression, investigators are required to specify which of docetaxel or abiraterone will be used and may **not** use both. Therefore from protocol v19.0 onwards, SOC use of docetaxel **or** abiraterone is permitted at the discretion of the treating clinician and patient.

The choice of SOC treatment must be selected **prior** to randomisation as not all SOC choices can serve as comparators for all research arms. Transdermal oestradiol cannot be given in addition to abiraterone within the STAMPEDE protocol due to lack of safety data available so far. Therefore the use of SOC abiraterone is **only** permitted in participants randomised to the metformin comparison (arm K and comparable arm A patients), see **Section 4.6** for details on comparison specific eligibility criteria.

2.2 DESIGN

STAMPEDE (also known as MRC PR08) is an innovative, multi-arm multi-stage (MAMS) randomised controlled trial open in multiple centres in the UK and Switzerland. The multi-arm design allows many treatment approaches to be tested simultaneously, and multi-stage refers to the pre-specified interim analyses that can be used to stop recruitment early to arms showing insufficient evidence of activity. The trial recruits people with high-risk locally advanced or metastatic prostate cancer, commencing long-term ADT for the first time.

The trial opened to recruitment in 2005 and initially assessed the effects of a bisphosphonate (zoledronic acid), a cytotoxic chemotherapeutic agent (docetaxel) and a cyclooxygenase (Cox-2) inhibitor (celecoxib), as single agents or combinations (arms B-F), referred to as the “original comparisons”.

Since the start of the trial, a number of new research arms have been added to STAMPEDE to evaluate:

- Abiraterone, a steroid synthesis inhibitor (arm G)
- Prostate radiotherapy for patients with newly-diagnosed metastatic disease (arm H)
- Enzalutamide, an inhibitor of androgen receptor signalling, given with abiraterone (arm J)
- Metformin, a repurposed anti-diabetic medication (arm K)
- Transdermal oestradiol, a repurposed alternative form of ADT (arm L)

2.3 PREVIOUSLY-REPORTED RESEARCH TREATMENTS

Data have been reported on the "original comparisons" evaluating zoledronic acid, docetaxel, celecoxib and the combination of zoledronic acid with docetaxel or with celecoxib (1, 2, 26). As such, the rationale for these treatments, along with their design and details of treatment administration, are no longer covered within this version of the protocol.

The primary survival analysis of the "abiraterone comparison" has also now been reported although these participants remain on active follow-up as subsequent analyses of long-term follow-up are planned(3). The rationale can be found in previous protocol versions, however treatment information remains as it is relevant to participants who remain on abiraterone given alone (arm G) and in combination with enzalutamide (arm J).

All previous versions of the protocol are available via www.stampedetrial.org, please refer to:

- Protocol version 11.0 and before for information relevant to "original comparisons" (Zoledronic acid, docetaxel, celecoxib)
- Protocol version 13.0 for information relevant to the "abiraterone comparison"

2.4 COMPARISONS IN FOLLOW-UP

The rationale for comparisons that have completed recruitment and remain in follow-up can be found in previous versions of the protocol. Recruitment completed to the radiotherapy arm in Sep-2016 as the revised recruitment target sample was reached. Treatment has been completed in all patients and the results will be reported when the data has matured. Recruitment was completed to the "enzalutamide and abiraterone comparison" in March 2016, as the recruitment target was reached. Participants remain on treatment therefore this information remains in this protocol version.

All previous versions of the protocol are available via www.stampedetrial.org, please refer to:

- Protocol version 15.0 or older for details relevant to "M1|RT comparison"
- Protocol version 16.0 or older for details relevant to "enzalutamide & abiraterone comparison"

2.5 RATIONALE FOR RECRUITING COMPARISONS

2.5.1 Metformin

All people joining STAMPEDE are planned for long-term ADT, a treatment associated with an increased risk of insulin resistance, hyperglycaemia, dyslipidaemia and obesity. Over 50% of people receiving long-term ADT will develop Metabolic Syndrome resulting in increased cardiovascular morbidity and mortality (45). Obesity and high bind insulin C-peptide levels, indicating insulin resistance are independent predictors of increased prostate cancer-specific mortality and the

presence of metabolic syndrome and diabetes in people treated with ADT is associated with shorter survival.

Metformin, which in non-diabetic individuals has been shown to lower the incidence of diabetes, counteracts some of these side-effects of ADT, including insulin insensitivity, hyperinsulinaemia and diabetes. It also reduces the levels of cholesterol, LDLs and triglycerides by inhibiting the fatty acid synthesis via activation of Adenosine Monophosphate Activated Kinase (AMPK) and decreases the platelet aggregation factor 1, platelet aggregation, vascular adhesion molecules, CRP and leptin (46-49). Through mitigation of the cardiovascular and metabolic consequences of ADT, metformin is proposed to reduce treatment-associated morbidity and improve all-cause mortality.

In addition, recent data has emerged consolidating the knowledge that cancer progression is linked integrally with metabolic modulators and that modification of this process by metformin has an important effect on cancer progression and survival. Pre-clinical data has shown that metformin is an important stimulator of AMPK which acts as the cellular "master switch" for energy regulation. AMPK acts to inhibit the effects of elevated insulin levels which promote metastasis, tumour growth and treatment resistance. Insulin increases mRNA and protein expression of steroidogenic enzymes leading to the up-regulation of intracellular testosterone levels, secreted androgens, thereby activating the AR (50). Metformin also influences the PI3K-AKT pathway and has an anti-proliferative effect via inhibitor of mTOR as well as targeting cancer stem cells. In vitro, metformin has been shown to inhibit androgen-induced IGF-IR up-regulation through disruption of androgen signalling (51).

Evidence in support of this includes a systematic review and meta-analysis of 13,008 people with type 2 diabetes mellitus (T2DM) and concurrent cancer which has shown improved survival in people treated with metformin compared with other anti-diabetic agents. In a systematic review of observational data from over 1 million people, there was a significant association seen between metformin and decreased risk of death from any cancer. Another systematic review found that the use of metformin in diabetic patients was associated with a significantly lower risk of cancer incidence and cancer mortality (52).(52). In a large retrospective cohort study of 3837 diabetic people with prostate cancer, metformin was associated with a decreased risk of prostate cancer specific mortality (HR=0.76 [0.64-0.89]) and death (HR=0.76 [0.70-0.82]). In a prospective non-randomised phase II study in non-diabetic CRPC patients, 36% of patients receiving metformin were progression-free at 3 months and >50% had a prolongation of their PSA doubling time (53).

In summary, meformin is proposed to mitigate many of the adverse side-effects of long-term ADT as well as having multiple potential anti-cancer effects and therefore STAMPEDE will evaluate re-purposing this treatment as a novel therapeutic approach in the management of high risk locally-advanced or metastatic prostate cancer.

2.5.2 Transdermal Oestradiol

2.5.2.A Background & Rationale

ADT with LHRH analogue injections suppresses testosterone to castrate levels, but also depletes oestradiol, since around 80% of oestradiol in men is derived by aromatisation from testosterone. Thus men who are treated with LHRH will have toxicities caused by low levels of both testosterone and oestrogen. The LHRH-associated toxicities which are due to low testosterone include loss of libido, erectile dysfunction and decrease in muscle mass. Other toxicities associated with LHRH such as osteoporosis, increased fracture risk, hot flushes, memory loss, dyslipidemia and increased body fat deposition are thought to be due to oestradiol deficiency. In particular, the adverse effect of LHRH on bone health has been well documented. Oestradiol deficiency prolongs the life-span of bone-resorptive osteoclasts, with the resulting imbalance between osteoclasts and bone-forming

osteoblasts increasing the rate of bone thinning. This may lead to osteoporosis and increased risk of fracture, with the rate of fracture increasing with duration of LHRH.

Transdermal oestradiol is a potential alternative to LHRH that may avoid some treatment-related side-effects, therefore improving quality-of-life, which would be advantageous if shown to be equally effective at prolonging survival. Exogenous administration of oestradiol suppresses androgen production through a negative feedback loop involving the hypothalamic-pituitary axis, whilst avoiding the fall in oestradiol associated with castrate levels of testosterone (54). This, in turn, mitigates the toxicities of LHRH associated with oestradiol deficiency. Oral oestrogen was previously used for ADT before the development of LHRH, but discontinued as first-line treatment due to increased thromboembolic toxicity, attributable to first-pass hepatic metabolism (55).

Parenteral administration (e.g. intravenous, intramuscular or transdermal oestradiol) avoids first-pass hepatic metabolism, mitigating the cardiovascular risk, as supported by results so far from the ongoing PATCH (Prostate Adenocarcinoma TransCutaneous Hormones [MRC PR09; ISRCTN70406718]) trial and previous studies evaluating parenteral oestradiol in the form of intramuscular polyestradiol phosphate (54, 56).

To date, there are a number of encouraging results from the PATCH trial demonstrating the safety and early activity of transdermal oestradiol compared to LHRH agonists in people with advanced hormone-naïve prostate cancer (see **Appendix I** for further details). In particular, similar rates of cardiovascular events have been observed in the transdermal oestradiol and LHRH arms, as well as equivalent rates of testosterone suppression (based on around 900 patients enrolled up to Oct-2015) (54). Transdermal oestradiol has been shown to avoid the loss in bone mineral density associated with LHRH, and results in improved metabolic profiles and quality-of-life compared to LHRH (57). Furthermore, a pre-planned, confidential, interim analysis of the PATCH trial in Jun-2013 based on progression-free survival (PFS) led to the trial being extended to phase III; that analysis included n=638 participants with 206 PFS events, reviewed against a pre-specified non-inferiority margin hazard ratio of 1.25 and 1-sided alpha 0.25. The phase III evaluation of clinical efficacy for transdermal oestradiol will be based on progression-free and overall survival as co-primary outcome measures.

Demonstrating that transdermal oestradiol is an equally effective approach to ADT would provide a globally important alternative (to LHRH), with the potential to reduce treatment-associated morbidity and improve quality-of-life. In addition, there is a possibility that transdermal oestradiol may improve overall survival compared to standard hormone therapy. First, transdermal oestradiol may reduce treatment-associated morbidity and could potentially benefit overall survival. Second, up to 30% of people with castrate-resistant prostate cancer respond to oral oestrogen as post-relapse therapy, suggesting oestradiol may potentially have additional direct anti-tumour effects (58).

2.5.2.B Meta-Analysis With PATCH Trial

To further assess the clinical efficacy of transdermal oestradiol, the relevant data from the "transdermal oestradiol comparison" within STAMPEDE will be combined with data from patients recruited into PATCH i.e. the "transdermal oestradiol comparison" within STAMPEDE is not sufficiently powered to form a stand-alone analysis. The evaluation of transdermal oestradiol will be based on a non-inferiority approach (in contrast to the other comparisons within STAMPEDE which are superiority questions), to test the hypothesis that transdermal oestradiol is at least as effective as standard hormone therapy, but with fewer side-effects.

Recruitment of patients to the "transdermal oestradiol comparison" through STAMPEDE enables the transdermal oestradiol research question to be answered more quickly than via PATCH alone. It also

reduces the number of participants allocated standard treatment alone in both trials, thereby increasing the proportion of participants receiving a novel treatment approach and improving trial efficiency.

As of Feb-2017, nearly 1,200 participants had been recruited directly to the PATCH trial (also coordinated by MRC CTU at UCL) for the phase III evaluation of clinical efficacy of transdermal oestradiol. The overall recruitment target for the transdermal oestradiol evaluation is approximately 2,000 participants (including initially around 500 to be recruited through STAMPEDE).

3 SELECTION OF INSTITUTIONS AND INVESTIGATORS

Centres who wish to participate in STAMPEDE should be registered with the MRC CTU at UCL for this purpose. Before any participants are registered or randomised, the CTU must receive a completed and signed Investigator Statement. The STAMPEDE Investigator Statement is signed by the Principal Investigator for that institution (download from http://www.stampedetrial.org/). The return of the Investigator Statement will be taken as confirmation of agreement to adhere to the trial protocol. In addition, a fully-signed model agreement is also required before recruitment can begin.

In compliance with the principles of GCP, all institutions participating in the trial will complete a delegation log and forward this to the CTU. Each person working on the STAMPEDE trial must sign off a section of this log indicating their responsibilities. CTU must be notified of any changes to trial personnel and/or their responsibilities. An up-to-date copy of this log must be stored in the Investigator Site file at the institution and also at CTU.

The Clinical Trial Authorisation (CTA) for the STAMPEDE trial requires that the Medicines and Healthcare Products Regulatory Agency (MHRA) be supplied with the names and addresses of all participating investigators/institutions. Trial staff at the CTU will perform this task; hence, it is vital to receive full contact details for all investigators prior to their entering participants.

Following substantial amendments and new comparisons opening, sites will be notified of relevant documents and training required and if and when they are able to participate. Further accreditation packs may be circulated as a result to update trial documentation.

SITE/INVESTIGATOR CRITERIA

3.1.1 Principle Investigator's Qualifications & Agreements

1. The investigator(s) should be qualified by education, training, and experience to assume responsibility for the proper conduct of the trial at their site and should provide evidence of such qualifications through an up-to-date curriculum vitae and/or other relevant documentation requested by the Sponsor, the REC, the IRB, and/or the regulatory authorities.

2. The investigator must hold a long term contract with their site. Locum members of staff cannot fill the role of Principle Investigator (PI).

3. The investigator should be thoroughly familiar with the appropriate use of the investigational products, as described in the protocol, the Reference Safety Information (RSI) (i.e. current Investigator Brochure or Summary of Product Characteristics) and other information sources provided by the Sponsor.

4. The investigator should be aware of, and should comply with, the principles of GCP and the applicable regulatory requirements. A record of GCP training should be accessible for all investigators.

5. The investigator/site should permit monitoring and auditing by the Sponsor, and inspection by the appropriate regulatory authorities.

6. The investigator should maintain a delegation log of appropriately-qualified persons to whom the investigator has delegated significant trial-related duties.

7. The investigator should sign an investigator statement, which verifies that the site is willing and able to comply with the requirements of the trial.

3.1.2 Adequate Resources

1. The investigator should be able to demonstrate a potential for recruiting the required number of suitable subjects within the agreed recruitment period (that is, the investigator regularly treats the target population).

2. The investigator should have sufficient time to properly conduct and complete the trial within the agreed trial period.

3. The investigator should have available an adequate number of qualified staff and adequate facilities for the foreseen duration of the trial to conduct the trial properly and safely.

4. The investigator should ensure that all persons assisting with the trial are adequately informed about the protocol, the investigational products, and their trial-related duties and functions.

5. The site should have sufficient data management resources to allow prompt data return to the CTU.

3.2 COMPARISON-SPECIFIC SITE ACCREDITATION

3.2.1 Transdermal Oestradiol Comparison: arms A & L

Only UK centres participating in STAMPEDE will be accredited for the “transdermal oestradiol comparison”, since treatment with transdermal oestradiol is administered using Progynova TS 100mcg/24 hours transcutaneous oestradiol patches (see **Section 6.2.7**) which are currently unavailable in Switzerland.

3.3 REQUIRED TRIAL DOCUMENTATION

Table 7 presents a summary of the required trial documentation for participating centres. Templates are provided on the STAMPEDE website, www.stampedetrial.org.

Table 7: Trial documentation required for participating centres

TRIAL DOCUMENTATION	TIMING
Confirmation of capacity and capability (including IRMER approval)	Before centre participation
Signed Investigator Statement	Before centre participation
Signature list & delegation of responsibilities	Before centre participation
Trial personnel contact details	Before centre participation
Participant information sheets (PIS), GP Letter & Informed consent form (ICF) on local paper	Before centre participation
Signed Clinical Trial Agreement between Trust and Sponsor (or Variation if applicable)	Before centre participation
Site initiation training	Before centre participation
Signed Pharmacy Pack acknowledgment	Before centre participation

4 SELECTION OF PARTICIPANTS

4.1 IDENTIFYING POTENTIAL TRIAL PARTICIPANTS

STAMPEDE recruits participants with high-risk prostate cancer who are commencing long-term androgen-deprivation therapy (defined as at least 2 years) for the first time. All participants must fulfil one of the following **broad disease categories:**

- High-risk newly-diagnosed non-metastatic, node-negative disease
- Newly-diagnosed metastatic or node-positive disease
- Previously radically treated, now relapsing with high risk features

See **Section 4.4** for detailed category inclusion criteria

4.2 APPROACH TO INFORMED CONSENT

Providing potential participants with information about STAMPEDE at the earliest opportunity will allow them to consider their participation and provide sufficient time to complete the required screening procedures to determine comparison-specific eligibility.

Throughout the consent process it must be made clear that the participant is able to refuse to take part in all or any aspect of the trial at any time, for any reason without incurring any consequence or impact on their standard treatment. All aspects of the trial e.g. sub-studies should be presented and optional participation discussed, but investigators are encouraged to adopt a staged approach where possible to avoid information overload.

Original signed consent forms must be kept in the site investigator file, a copy provided to the participant and an anonymised copy sent to the CTU following randomisation, refer to **Section 10.1.1**.

4.2.1 Screening Investigations Prior To Randomisation

All participants should have the following examinations performed to confirm eligibility prior to randomisation. Please note, all screening investigations should be recent such that they reflect the participant's current disease status. The PI or a delegated clinician should confirm and sign off eligibility prior to randomisation and should be documented in the patients notes.

The following imaging is always required:

- Cross-sectional imaging (CT, MRI, PSMA-CT-PET or Choline-PET-CT) of pelvis and abdomen
- Bone Scan (or equivalent e.g. whole body MRI, PSMA-CT-PET or Choline-PET-CT)
- Chest X-ray (only if chest was not included in cross sectional imaging i.e. CT, Choline-PET-CT or PSMA-CT-PET which would be preferable; MRI imaging of chest is not sufficient on its own)

Please note, for trial purposes M1 disease will be defined using internationally agreed criteria, therefore M1 staging cannot be based solely on PET avid lesions. To be considered M1, the metastatic lesion must also be visible on standard imaging i.e. bone scan, CT or MRI.

The following bloods and additional measurements are required prior to randomisation:

- Pre-hormone treatment PSA (this must be obtained within 6 months of randomisation)

- Haematology: Full blood count
- Biochemistry: Liver function tests, Serum creatinine
- HbA1c
- Systolic and diastolic blood pressure

Participants who initially fail to meet the trial eligibility criteria can be re-screened at a later date if timelines permit. Of note, it is acceptable to use a full blood count measured prior to chemotherapy to confirm eligibility for participants randomised prior to, or during, SOC docetaxel treatment.

Prior to randomisation:

- Check details of any prior treatments for prostate cancer
- Check any contraindications to radiotherapy or research treatment
- Check concomitant medications (see **Section 6.3.4** and **Section 6.3.5** for details on relevant drug interactions relevant to metformin and transdermal oestradiol)

It is expected that patients are fit for trial purposes, by assessment of the treating clinician, including, but not limited to, having a controlled blood pressure prior to randomisation.

4.2.2 Baseline Investigations required for participants allocated to arms A, K, L

The following blood tests and additional measurements are required at baseline (within 4 weeks before or after randomisation):

- Biochemistry: Cholesterol, albumin
- Fasting glucose and fasting triglycerides
- Lipid profile (fasting or non-fasting; total cholesterol, LDL and HDL)
- Baseline PSA (ideally within 2 weeks prior to randomisation)
- ECG
- Waist circumference measurement
- Weight and height

Where available, the following blood test is required:

- Testosterone (pre-ADT)

See **Table 2** for a detailed schedule of assessments for all participants randomised to arms A, K or L.

4.3 PRIOR PERMITTED SOC TREATMENTS

4.3.1 Hormone Treatment Prior To Randomisation

From protocol v16.0, participants can potentially be randomised to the "transdermal oestradiol comparison" and it would be preferable for these participants to have had as little exposure to ADT as possible.

Within the separate PATCH trial, participants are randomised within 8 weeks after starting anti-androgens and cannot have received an LHRH injection. This approach is also favoured in STAMPEDE, but participants who have received a single 4-week (or 1-month) LHRH injection remain eligible, as shown in **Table 8.** Note that anti-androgen monotherapy is not permitted as a form of long-term hormone therapy but the initial use is encouraged to meet the eligibility criteria for the "transdermal oestradiol comparison". Anti-androgens may include flutamide or bicalutamide, please note that the use of cyproterone will mean the participant is ineligible for arm L.

Table 8: Maximum prior hormone therapy

TIME CONSIDERATIONS	PRIOR ANTI-ANDROGENS	PRIOR LHRH	ELIGIBLE FOR INCLUSION TO
Ideal duration	≤8 weeks	≤4 weeks	A:K:L
Maximum duration	14 weeks	12 weeks	A:K

4.3.2 Standard-Of-Care (SOC) Radiotherapy

In participants with non-metastatic disease, the treating clinician and participant must have decided, prior to randomisation, whether prostate radiotherapy is to be given as part of SOC.

4.3.3 Standard-Of-Care (SOC) Docetaxel

The treating clinician and participant must have decided, **prior** to randomisation, whether docetaxel is to be given as part of SOC. Investigators should aim to start SOC docetaxel treatment within *around* 14 weeks after starting hormone therapy, consistent with the timelines achieved for research arm C. Participants may start docetaxel treatment prior to randomisation. See Section 6.1.3 for treatment details.

If SOC docetaxel treatment was not commenced prior to randomisation and participants are subsequently allocated to receive transdermal oestradiol (Arm L), it is recommended that docetaxel treatment commences *after* participants have been established on transdermal oestradiol for around 4 weeks, when most participants are likely to have completed the induction period (see Section 6.2.7.B). SOC docetaxel **cannot** be selected if SOC abiraterone is selected.

4.3.4 Standard-Of-Care (SOC) Abiraterone

The treating clinician and participant must have decided, **prior** to randomisation, whether abiraterone is to be given as part of standard-of-care (SOC). SOC abiraterone **cannot** be selected if SOC docetaxel is selected. See Section 6.1.4 for treatment details.

Abiraterone may have commenced prior to randomisation. The use of abiraterone impacts on comparison-specific eligibility. At present, there are no safety data available on the use of abiraterone in combination with transdermal oestradiol and until such data are available, participants planned for SOC abiraterone are **only** eligible for randomisation to the “metformin comparison”; see Section 4.6 for comparison-specific eligibility criteria. However, a sub-study to evaluate the safety of abiraterone used in combination with transdermal oestradiol is planned within the PATCH trial. When completed, the findings will be reviewed by the relevant committees and sites to be advised if, going forward, participants planned for SOC abiraterone will be eligible for the “transdermal oestradiol comparison”.

4.4 GENERAL INCLUSION CRITERIA

The PI or a delegated clinician must confirm that participants fulfil all the criteria in one of the following three categories:

4.4.1 High-Risk Newly-Diagnosed Non-Metastatic Node-Negative Disease

Both:

- At least two of: T category T3/4, PSA≥40ng/ml or Gleason sum score 8-10
- Intention to treat with radical radiotherapy (unless there is a contra-indication; exemption can be sought in advance of consent, after discussion with CTU)

OR

4.4.2 Newly-Diagnosed Metastatic Or Node-Positive Disease

At least one of:

- Stage T_{any} N+ M0
- Stage T_{any} N_{any} M+

OR

4.4.3 Previously Radically Treated, Now Relapsing (Prior Radical Surgery And/or Radiotherapy)

At least one of:

- PSA ≥4ng/ml and rising with doubling time less than 6 months
- PSA ≥20ng/ml
- N+
- M+

AND

4.4.4 General inclusion criteria required for all participants

I. Histologically confirmed prostate adenocarcinoma
II. Intention to treat with long-term androgen deprivation therapy
III. Treating clinician and participant should have decided if additional systemic therapy with docetaxel or abiraterone is to be used as part of the standard-of-care prior to randomisation
IV. Fit for all protocol treatment[1] and follow-up, WHO performance status 0-2[2]
V. Have completed the appropriate investigations prior to randomisation
VI. Adequate haematological function: neutrophil count >$1.5x10^9$/l and platelets >$100x10^9$/l
VII. Adequate renal function, defined as GFR >30ml/min/$1.73m^2$
VIII. Written informed consent
IX. Willing and expected to comply with follow-up schedule
X. Using effective contraceptive method if applicable

[1] Medical contraindications to the trial medications are given in **Section 6**
[2] For WHO performance status definitions see **Appendix A**

4.5 GENERAL EXCLUSION CRITERIA

I. Prior systemic therapy for locally-advanced or metastatic prostate cancer (except as listed in **Section 4.3)**
II. Metastatic brain disease or leptomeningeal disease
III. Abnormal liver functions consisting of any of the following:
- Serum bilirubin ≥1.5 x ULN (except for participants with Gilbert's disease, for whom the upper limit of serum bilirubin is 51.3µmol/l or 3mg/dl)
- Aspartate aminotransferase (AST) or alanine aminotransferase (ALT) ≥2.5 x ULN

IV. Any other previous or current malignant disease which, in the judgement of the responsible clinician, is likely to interfere with STAMPEDE treatment or assessment
V. Any surgery (e.g. TURP) performed within the past 4 weeks
VI. Participants with significant cardiovascular disease, including:
- Severe/unstable angina
- Myocardial infarction less than 6 months prior to randomisation
- Arterial thrombotic events less than 6 months prior to randomisation
- Clinically significant cardiac failure requiring treatment, defined as New York Heart Association (NYHA) class II or above[1]
- Cerebrovascular disease (e.g. stroke or transient ischaemic episode) less than 6 months prior to randomisation
- Or any other significant cardiovascular disease that in the investigator's opinion means the participant is unfit for any of the study treatments.

VII. Prior chemotherapy for prostate cancer[2]
VIII. Prior exposure to long-term hormone therapy before randomisation (unless as described in **Section 4.3.1**)
IX. Prior exposure to systemic treatment for prostate cancer (excluding ADT or participants receiving abiraterone as part of SOC)

[1] NYHA classifications can be found in Appendix A
[2] Excluding participants receiving docetaxel as part of SOC

4.6 COMPARISON-SPECIFIC ELIGIBILITY CRITERIA

In addition to the general inclusion and exclusion criteria, the following comparison-specific eligibility criteria apply.

4.6.1 Metformin Comparison (randomisation between arm A and arm K)

In addition to the general inclusion and general exclusion criteria the following comparison-specific inclusion criteria must be met to be eligible for randomisation to the "metformin comparison":

- Hb A1c <48mmol/mol (equivalent to <6.5%)[1]
- Adequate renal function, defined as GFR ≥45ml/min/1.73m[2] (except for Switzerland [2])
- No history of lactic acidosis or predisposing conditions
- No current or previous treatment with metformin
- No contraindications to metformin

The method used to determine glomerular filtration rate may vary according to local practice. Equations that either estimate glomerular filtration rate (eGFR) or creatinine clearance (CrCl) may be used and the same threshold value applies. Where possible, HbA1c should be performed prior to commencing SOC docetaxel to reduce the likelihood of corticosteroid-related hyperglycaemia impacting on eligibility. All participants with abnormal baseline HbA1c (i.e. 6.5% or higher) should be informed and referred to their GP for further management.

4.6.2 Transdermal Oestradiol Comparison (randomisation between arm A and arm L)

In addition to the general inclusion and exclusion criteria, participants fulfilling all of the following are eligible for the "transdermal oestradiol comparison":

- ≤8 weeks of anti-androgen (AR-antagonists) use
- ≤1 dose of monthly or 4-weekly LHRH agonist/antagonist
- No prior LHRH agonist injection with a stated duration of effect greater than 1 month
- ≤12 weeks since first dose of any hormone therapy
- Not had a bilateral orchidectomy
- No use of cyproterone acetate (77) prior to randomisation
- No known porphyria
- No known history of deep vein thrombosis or pulmonary embolism confirmed radiologically
- No known thrombophilic disorder (e.g. Protein C, Protein S, antithrombin deficiency)
- Not planned to receive SOC abiraterone (see **Section 4.3.4** for information)

****
****
****
****

4.7 SUB-STUDY ELIGIBILITY CRITERIA

There are currently four sub-studies that aim to further the understanding of the biology of prostate cancer through additional genetic analyses and correlation with clinical data. For details on each sub-study, see **Section 17.2.**

4.7.1 Eligibility for germline DNA sub-study (Saliva samples)

All newly randomised trial participants who join arms A, K or L are asked to provide a saliva sample from which germline (inherited) DNA can be extracted.

Participants randomised from **15-Nov-2011** onwards who consented to provide a blood spot (Consent Form version 4.0 part K) can also be retrospectively approached to provide a saliva sample providing they have received the REC-approved letter explaining the need for additional saliva sample collection as the DNA extraction using the blood spot method did not work as well as anticipated.

For further information please refer to the **Sample collection and handling manual**.

4.7.2 Eligibility for the circulating tumour DNA sub-study (sequential blood samples)

All newly registered or randomised trial participants are eligible to join the circulating tumour DNA (ctDNA) sub-study.

The criteria for enrolment in the ctDNA sub-study can be summarised as:

- Newly registered or randomised STAMPEDE participants
- Informed consent to provide sequential blood samples according to the sampling schedule

For details on the sample collection and the different sampling schedule for M0 and M1 participants, please refer to the **Sample collection and handling manual**.

4.7.3 Eligibility for tumour sample analysis (FFPE blocks)

All newly randomised trial participants should be provided with the STAMPEDE Additional Research Participant Information Sheet in order to consider optional donation of remaining diagnostic prostate cancer tissue stored as formalin fixed paraffin embedded (FFPE) blocks.

The criteria for enrolment into the FFPE block collection:

- Newly randomised STAMPEDE participants who have not already donated FFPE blocks for the biomarker screening sub-study.
- Informed consent to gift remaining tissue to be used for additional research analyses

4.7.4 Eligibility for the Biomarker-Screening Pilot study

A selected number of sites are invited to participate in a pilot aiming to evaluate different approaches to prospective biomarker-screening. Sub-study specific training will be provided to research teams at participating sites.

If relevant, refer to **Section 17.2.4** and the **Biomarker screening manual** for eligility criteria and further details.

5 RANDOMISATION AND ENROLMENT

5.1 RANDOMISATION

Participant eligibility will be confirmed during the randomisation process and participants will be allocated to any of the open research comparisons for which they are eligible (see **Section 4.6**). To randomise a participant please carefully complete the Randomisation CRF and then contact the CTU.

RANDOMISATION
Call MRC CTU at UCL, Monday to Friday 0900-1700
Excluding public holidays or dates when notice has been given by the CTU.
Tel: +44 (0) 20 7670 4777

A trial ID and treatment will be allocated and given over the phone or by email. In addition, a letter confirming these details will be sent. The trial ID will be the primary way in which the participant will be identified and should be used in all correspondence. Centres should send a letter to the participant's GP to inform them of their trial participation and treatment allocation. The GP letter is supplied as a template and can be downloaded from the trial website, www.stampedetrial.org.

5.2 CO-ENROLMENT GUIDELINES

STAMPEDE participants should not join any other interventional clinical trials of prostate cancer treatment until a failure-free survival (FFS) event has been experienced and reported. After this point, the participant may be entered into further treatment studies e.g. evaluating treatments for CRPC. The primary outcome measure of STAMPEDE is overall survival and follow-up reports must continue after co-enrolment. Participation in post-progression studies must be reported to CTU on the Co-enrolment CRF; details of any interventional treatments received post-progression in such studies should be reported on the Additional Treatment Log.

Data sharing agreements with "downstream" trials are encouraged to improve data quality in both trials and to reduce costs to both organisations.

Co-enrolment in non-interventional studies for any indication is permitted at any time providing that it does not interfere with treatment or assessment in STAMPEDE; this does **not** require reporting using the Co-enrolment CRF which is designed to capture interventional prostate cancer clinical trials only.

6 TREATMENT OF PARTICIPANTS

6.1 STANDARD-OF-CARE (SOC)

The SOC for this patient group is **androgen deprivation therapy** (ADT) as per local practice (see **Section 6.1.1**). For some participant groups, this should now be supplemented with SOC radiotherapy (see **Section 6.1.2**). From protocol v14.0 onwards the SOC permits docetaxel for all suitable participants (see **Section 6.1.3**). From protocol v19.0 onwards, where accessible SOC abiraterone may also be permited as an alternative to docetaxel.

In summary, SOC treatment is defined as being **one** of the following combinations:

- ADT alone
- ADT + Prostate Radiotherapy (RT) +/- nodal
- ADT + Docetaxel
- ADT + Docetaxel + RT
- ADT + Abiraterone*
- ADT + Abiraterone + RT*

*__Note__ not all forms of SOC treatment are permitted in all comparisons. SOC abiraterone is **only** permitted within the metformin comparison.

6.1.1 Hormone Therapy

The planned duration of ADT should be **at least 2 years** and lifelong in those with metastatic disease. With the exception of those allocated to transdermal oestradiol (Arm L), all participants will receive ADT as per local practice to achieve castrate levels of testosterone. The method of planned or current long-term standard-of-care ADT must be specified prior to randomisation. See below for the permitted methods of ADT and see **Section 4.3.1** for more information on ADT timing before randomisation. Participants allocated to Arm L will go on to receive transdermal oestradiol in place of standard ADT methods.

6.1.1.A Bilateral Orchidectomy

Operations should be performed by appropriately trained surgeons. A total or sub-capsular orchidectomy may be performed. Participants having a bilateral orchidectomy are required to adhere to the same timelines as specified in **Section 4.3.1** unless there is a strong clinical reason not to do so.

6.1.1.B LHRH Agonists e.g. goserelin, leuprorelin

LHRH agonists used according to local practice. The prophylactic use of anti-androgens to prevent tumour “flare” is recommended.

6.1.1.C LHRH Antagonists e.g. degarelix

LHRH antagonists used according to local practice. The use of prophylactic use of anti-androgens to prevent tumour “flare” is not necessary.

6.1.1.D Dual Androgen Blockade

Long-term use of anti-androgens alongside LHRH agonists, according to local practice. Note this was previously referred to as maximum androgen blockade.

6.1.1.E Others

Discuss with the CTU.

6.1.2 Standard-Of-Care (M0) Prostate RT

6.1.2.A N0M0 Participants

Investigators should give standard radiotherapy (RT) to participants with node negative, non-metastatic disease (N0M0), in accordance with data from the PR07 and SPCG trials. If there is an intention to omit radiotherapy (e.g. RT is contraindicated for the participant) in participants with N0M0 disease this must be discussed with the STAMPEDE trial team before randomisation to confirm eligibility. See **Section 6.7** for further details of radiotherapy administration.

6.1.2.B N+M0 Participants

For participants with node-positive, M0 disease there are no randomised data on whether radiotherapy is indicated or not. However the NCIC PR.3 / MRC PR07 trial included participants with unknown nodal status who received whole pelvic radiotherapy (12) and demonstrated a large overall benefit. Additionally, non-randomised data from the STAMPEDE control arm suggests that the benefit observed in participants with N0M0 disease can be extended to those with pelvic nodal involvement. Therefore the STAMPEDE TMG recommends that pelvic nodal radiotherapy be considered for participants with node-positive, M0 disease at the discretion of the treating clinician (13).

6.1.2.C Planned Use Of SOC RT

Suitability for radiotherapy is assessed by the treating clinicians. Investigators will be asked to state their intention with regards to planned radiotherapy in this group at randomisation. Intention to give radiotherapy (or not) for **all** participants must be stated at randomisation to ensure that there is no bias towards particular combinations of systemic therapy with radiotherapy.

SOC radiotherapy is not a core part of the trial, therefore we intend to collect minimal data about the radiotherapy administered. It is accepted that some participants will develop progressive disease before radiotherapy can be administered and if this occurs the reasons for non-delivery of treatment must be recorded on the Radiotherapy Detail CRF.

6.1.3 Standard-Of-Care Docetaxel

Investigators are strongly encouraged to consider giving docetaxel as part of the standard-of-care for participants with newly-diagnosed metastatic disease, based on the survival benefit demonstrated by both STAMPEDE in the primary analysis of the "original comparisons" and CHAARTED (14) (15, 26). Investigators may also consider giving docetaxel to participants with high-risk locally-advanced disease, given both the significant improvement in failure-free-survival and consistency of effect for prostate cancer-specific survival shown by STAMPEDE.

The treating clinician and participant must have decided **prior** to randomisation if docetaxel is to be given. Treatment may start prior to randomisation. For participants allocated to receive transdermal oestradiol (Arm L) who have not already started prior to randomisation, it is recommended that docetaxel commences around 4 weeks after starting research treatment (see **Section 6.2.7**). A SOC Docetaxel Treatment CRF should be completed for all participants randomised to STAMPEDE confirming whether docetaxel was given or not, regardless of being planned.

Docetaxel is given according to local protocols as a standard non-trial treatment. The regime used previously within STAMPEDE was 75mg/m^2 Day 1 as 1hr IV infusion, plus prednisolone 5mg BID for 21 days repeated every 3 weeks for a maximum of 6 cycles. Treatment was started after a median of 9 weeks after commencing ADT. GCSF use is at the investigator's discretion but should be considered; prednisolone may be omitted.

6.1.4 Standard-Of-Care Abiraterone

From the date that protocol v19.0 is activated at site, the treating clinician and participant may consider the use of abiraterone in the castrate-sensitive setting, termed SOC abiraterone, where this is available. SOC abiraterone is **only** permitted for participants within the metformin comparison (i.e. participants allocated to arm K or the comparable control group in arm A). This is because there is currently insufficient safety data to support the use of abiraterone with transdermal oestradiol.

The use of SOC abiraterone must be decided prior to randomisation to ensure use is balanced between control (arm A) and treatment arm (arm K). Therefore SOC abiraterone is not permitted in participants previously randomised prior to activation of protocol v19.0. In the absence of data supporting concurrent or sequential use in the absence of disease progression, investigators are required to specify which of docetaxel or abiraterone will be used and may **not** use both.

Abiraterone is given according to local protocols as a standard non-trial treatment. The dosing, safety monitoring and toxicity management contained within the STAMPEDE protocol refers to research abiraterone given to participants previously allocated to arms G or J, but may be used as a guide. As with other SOC therapies, minimum data collection will be required. A SOC abiraterone treatment CRF will be required for all participants who have started or who plan to start SOC abiraterone at randomisation. See **Section 7.2.3** for details of data collection for SOC abiraterone.

6.2 RESEARCH TREATMENTS

6.2.1 Required Timelines When Starting Research Treatment

Allocated treatment should start promptly after randomisation. Please refer to the relevant sub-sections in **Section 6.2** for more information on starting of research treatment.

6.2.2 Research Abiraterone + Prednisolone (relevant to arms G & J)

Note: recruitment has closed to all research comparisons containing abiraterone; that is Arm G (SOC + abiraterone) and Arm J (SOC + enzalutamide + abiraterone).

Abiraterone will be administered as a single 1000mg daily oral dose (4 tablets to be taken together once a day) together with prednisolone or prednisone 5mg daily to prevent secondary mineralocorticoid excess. Abiraterone absorption is increased by food. The tablets should be taken at least 2 hours after food, swallowed whole with some water. No food should be eaten for 1 hour afterwards.

Prednisolone (prednisone in Switzerland) should be taken as a single dose with food in the morning. If a participant allocated to receive abiraterone develops only biochemical failure, the responsible clinician might switch from abiraterone + prednisolone 5mg od to abiraterone and dexamethasone 0.5mg od. The steroid dose should also be reviewed in the event of side-effects due to mineralocorticoid excess (e.g., hypokalaemia, hypertension, peripheral oedema) see **Table 9**, **Table 10** and **Table 12**.

Participants receiving or planned for dual androgen blockade (DAB) at randomisation should not continue anti-androgens if allocated to receive abiraterone

6.2.2.A Treatment duration

Please note that for some participants treatment with abiraterone may continue until all categories of disease progression or up to a maximum duration of 2 years.

Arm G (SOC + abiraterone) participants who have now reached their maximum duration of 2 years on trial treatment include:

- All N0M0 participants who received radical radiotherapy
- All N+M0 participants who received radical radiotherapy

Arm J (SOC + enzalutamide + abiraterone) participants who have now reached their maximum duration of 2 years on trial treatment include:

- N0M0 participants starting treatment over 2 years ago who received radical radiotherapy
- N+M0 participants who received radical radiotherapy and starting treatment over 2 years ago

All such participants should have reported permanent stopping of research abiraterone on an End of Research Treatment CRF. Please see sections below for more information.

In participants with **M1 disease or relapsed previously radically treated disease**, treatment with abiraterone will continue from randomisation until all categories of disease progression have occurred, consistent with the COU-AA-301 and COU-AA-302 trials (78, 79) i.e. abiraterone will be given for these participants until a composite of:

- PSA progression (as defined in **Section 7.1.3.A)**
- Radiological progression (appearance of new lesions or progression of existing lesions) **and**
- Clinical progression (defined as new cancer-related symptoms)

It is accepted that these flexible criteria for stopping treatment with abiraterone are open to the investigator's interpretation and discretion. Participants might continue treatment beyond the first failure-free survival (FFS) event; however the first FFS event must be reported as per the other arms; all categories of disease progression (PSA, radiological and clinical) need to be reported once.

See **Section 7.1.3** for further information on the trial definition of progression.

In participants **with N0M0 disease or N+M0 disease undergoing radical radiotherapy**, treatment will continue for a maximum of 2 years or until all categories of disease progression if this occurs before 2 years. ADT can also be discontinued in this group after a minimum of 2 years at the discretion of the local investigator (see **Section 6.1.1**).

For participants with **N+M0 disease not planned for radical radiotherapy**, or who do not receive planned prostate RT, treatment will continue as for participants with M1 disease until all categories of disease progression.

For trial purposes the duration of treatment is determined by the intention at the time of randomisation e.g. if a participant with M0N+ disease is initially not planned for radical radiotherapy, the planned duration of treatment to progression should not change if radical radiotherapy is subsequently given. Discuss with the CTU team if you require further advice.

6.2.3 Research Abiraterone + Prednisolone: Administration And Management Of Toxicities

Abiraterone absorption is increased by food therefore should be taken on an empty stomach without food. The tablets should be taken at least 2 hours after food, swallowed whole with some water. No food should be eaten for 1 hour afterwards.

Prednisolone (prednisone in Switzerland) should be taken as a single dose with food in the morning.

6.2.3.A Abiraterone Contraindications

- Unusual or allergic reaction to past abiraterone acetate treatment
- Uncontrolled hypertension
- Uncontrolled heart failure
- Active or chronic liver disease

See **Section 6.3** for details on contraindicated concomitant medications and **Table 19** for details on drugs that may interact with abiraterone.

6.2.3.B Research Abiraterone Special Warnings And Required Monitoring Whilst on Treatment

:: Hypokalaemia

Abiraterone may cause hypokalaemia due to secondary mineralocorticoid excess, this can be counteracted by co-prescription of prednisolone. Regular monitoring of serum potassium levels are required whilst receiving treatment with abiraterone. The Investigator Brochure states that monitoring should be performed 2-weekly for the first 12 weeks and then every month or as per protocol whilst receiving abiraterone (80).

When abiraterone is used routinely in the licenced setting (CRPC), it is common practice to prescribe the next course of treatment for 8 weeks to participants who have been on abiraterone for over 12 months with no abnormalities, having checked that the potassium is normal (or >3mmol/L and in line with previous results) prior to writing the prescription.

The STAMPEDE protocol requires continued monthly monitoring for participants who experience hypokalaemia related to research abiraterone. For participants who have been monitored appropriately with no evidence of hypokalaemia, the frequency of monitoring may be reviewed after 12 months on treatment and, at the discretion of the investigator, may be reduced to every 2 months if judged appropriate. This is consistent with the approach adopted in the LATITUDE trial in which abiraterone was evaluated in high-risk metastatic hormone-naïve prostate cancer (16). Treatment should always be interrupted in the presence of symptoms (constipation, palpitations, fatigue, muscle weakness or spasm, tingling or numbness), see **Table 11.**

:: Hepatic Impairment

Abiraterone treatment can be associated with increased liver enzymes and hepatotoxicity therefore regular monitoring of liver function tests (LFTs) is required whilst on treatment. LFTs (ALT or AST and bilirubin). The Investigator Brochure states that monitoring should be performed 2-weekly for the first 12 weeks and then every month or as per protocol (80) whilst receiving abiraterone.

When abiraterone is used routinely, it is common practice to prescribe the next course of treatment for 8 weeks to participants who have been on abiraterone for over 12 months with no abnormalities, having checked that the liver function tests are normal, or no worse than grade 1 prior to writing the prescription (16). This is acceptable provided those with grade 1 abnormalities are monitored more frequently and treatment is interrupted if they increase to grade 2, see **Table 11.** The STAMPEDE protocol requires monthly monitoring in the first 12-months on treatment with research abiraterone. For participants who have been monitored appropriately with no evidence of liver function abnormality, the frequency of monitoring may be reviewed after 12 months on treatment and, at the discretion of the investigator, reduced to every 2 months if judged appropriate, consistent with the approach adopted in the LATITUDE trial(16).

If clinical symptoms or signs suggestive of hepatotoxicity develop, serum transaminases, in particular serum alanine aminotransferase (ALT), should be measured immediately. See **Table 11** for the management of abiraterone induced hepatotoxicity.

:: Blood pressure management
Abiraterone may cause hypertension. Regular monitoring of blood pressure is required whilst receiving treatment. The Investigator Brochure states that monitoring should be performed 2-weekly for the first 12 weeks and then every month whilst receiving abiraterone. STAMPEDE Investigators will be required to ensure monthly blood pressure monitoring is performed and reviewed in the first 12-months on treatment, it is acceptable for this to be documented self-monitoring or via the GP providing this is reviewed at each follow-up. After 12 months on treatment, it is acceptable for blood pressure monitoring to be performed every 2-months and reviewed at each follow-up visit, providing blood pressure has been well controlled. For the management of abiraterone induced hypertension see **Table 9.**

:: Cardiovascular history
Abiraterone acetate should be used with caution in participants with a history of cardiovascular disease. The safety of abiraterone acetate in participants with left ventricular ejection fraction <50% or New York Heart Association (NYHA) Class III or IV heart failure has not been established. Before treatment with abiraterone acetate, hypertension must be controlled and hypokalaemia must be corrected.

Caution is required in treating participants whose underlying medical conditions might be compromised by increases in blood pressure, hypokalaemia, or fluid retention, e.g. those with heart failure, recent myocardial infarction, or ventricular arrhythmia.

:: Renal Impairment
No dose adjustments are required in renal impairment; however caution is advised if participants develop severe renal impairment as there is limited clinical data in this population. Systemic exposure to abiraterone after a single oral 1000mg dose did not increase in participants with end-stage renal disease on dialysis.

6.2.3.C Abiraterone Undesirable Effects
The most common adverse drug reactions observed in the integrated safety data for those participants who received 1000mg abiraterone acetate plus prednisone or prednisolone in clinical studies (n=1,070) were fatigue, arthralgia, peripheral oedema, back pain, bone pain, nausea, constipation, hypokalemia and anaemia.

The adverse events graded as 3 or 4 and which occurred in more than 5% of participants were fatigue, peripheral oedema, anaemia and back pain see **Appendix C**.

6.2.3.D Abiraterone Overdose
Human experience of overdose with abiraterone is limited. There is no specific antidote to abiraterone acetate. In the event of an overdose, administration of abiraterone acetate should be stopped and general supportive measures undertaken, including monitoring for cardiac arrhythmias, liver function and electrolytes.

6.2.3.E Management Of Specific Toxicities From Prednisolone
The co-administration of prednisolone/prednisone 5mg once daily is required whilst receiving abiraterone to prevent secondary mineralocorticoid excess and 5 mg once daily is used in this trial.

Prednisolone/prednisone dose increase of up to 5mg BID is recommended to manage mineralocorticoid-related toxicities (e.g., hypokalaemia, hypertension, peripheral oedema) see Table 9, Table 10 and Table 12.

If a participant experiences serious symptoms of Cushing's syndrome (e.g., weight gain, muscle loss) investigators may reduce the steroid dose but participants should be closely monitored for symptoms of secondary mineralocorticoid excess. It should be noted that weight gain and muscle loss are also associated with ADT

Table 9: Management of abiraterone associated hypertension (given alone or with enzalutamide)

TOXICITY EVENT	ACTION
Grade 1	Management as per investigator
Grade 2	Management as per investigator with anti-hypertensive treatment. Follow local guidance for selection of anti-hypertensives but avoid thiazide diuretics to minimise risk of serum potassium derangement. Calcium channel antagonists or beta blockers are often preferred.<br>As with other symptoms of mineralocorticoid excess, consider increasing prednisolone dose to 5mg BID.
Grade 3-4	**Withhold abiraterone and enzalutamide**. Adjust or add anti-hypertensive medications to mitigate the toxicity. When hypertension resolves to Grade ≤1 or baseline, resume both enzalutamide and abiraterone at full dose with prednisolone 5mg bid.

A cardiologist's opinion should be considered if blood pressure control is not achieved within 4 weeks.

Table 10: Management of abiraterone associated hypokalaemia (given alone or with enzalutamide)

TOXICITY EVENT	ACTION
Grade 1<br>(<LLN- 3.0mmol/L)	Supplement with oral potassium and monitor closely and increase prednisolone dose to 5mg BID.<br>Exclude and manage other causes of hypokalemia.
Grade 2<br>(<LLN – 3.0mmol/L and symptomatic)	**Pause abiraterone.**<br>Supplement with oral potassium and monitor closely and increase prednisolone dose to 5mg BID.<br>Exclude and manage other causes of hypokalemia.<br>Re-start abiraterone with close monitoring, discontinue if recurs.
Grade 3<br>(<3.0–2.5mmol/L)<br>or Grade 4<br>(<2.5mmol/L and life-threatening)	Abiraterone will be **permanently discontinued** and the participants will be hospitalized for intravenous potassium replacement and cardiac monitoring. After the return of serum potassium to normal, prednisolone will be discontinued. The participant can continue on enzalutamide alone. If hypokalaemia persists, consider a dose reduction of enzalutamide to 120mg once a day.

Table 11: Management of abnormal Liver Function Tests (LFTs) associated with abiraterone (given alone or with enzalutamide)

TOXICITY EVENT	ACTION
**Grade 1** increases in AST, ALT or bilirubin (e.g. increase in AST or ALT from ULN to 2.5X ULN; increase in total bilirubin from ULN to 1.5X ULN)	The frequency of LFT monitoring should be increased to at least weekly, if the investigator judges that the laboratory abnormalities are potentially related to study medication.<br>No dose reduction is required.<br>Providing LFTs are stable for 4 weeks, resume monthly checks.
**Grade 2** increases in AST, ALT or bilirubin (e.g. increase in AST or ALT to >2.5-5X ULN; increase in total bilirubin from >1.5-3X ULN)	Withhold abiraterone, enzalutamide and all other concomitant medications that are potentially hepatotoxic.<br>The frequency of LFT monitoring should be increased to at least weekly until the liver function tests return to baseline value or grade 1 when all trial medication can be re-started.<br>No dose reduction is required after one episode providing this resolved within 4 weeks but should be considered if Grade 2 derangements recurs.
**Grade 3** increases in AST, ALT or bilirubin (e.g. increase in AST or ALT to >5X ULN; increase in total bilirubin to >3X ULN),	Withhold abiraterone and enzalutamide and all other concomitant medications that are potentially hepatotoxic.<br>At least weekly monitoring is required until the LFTs return to baseline value or Grade 1.<br>enzalutamide can be re-started with no dose reduction. See below for abiraterone re-challange.
**Grade 4** increases in AST, ALT or bilirubin (e.g. increase in AST or ALT to >20x ULN; increase in total bilirubin to >10x ULN)	Participants must discontinue abiraterone and enzalutamide immediately.<br>At least weekly monitoring is required until the LFTs return to baseline value or grade 1 and then prednisone can be discontinued and the investigator can consider restarting enzalutamide.<br>Abiraterone should not be re-introduced.
RE-CHALLENGE	ACTION
Recurrent grade 2 derangement	Reduce to 750mg once LFTs return to grade 1
If study treatment resumption is considered for participants who have experienced Grade 3 increases in AST, ALT, or bilirubin	Resume study treatment with abiraterone dose reduction to 750mg when grade 3 toxicities resolve to grade 1 or baseline.
If Grade 3 or higher increases in AST, ALT or bilirubin recur after the first dose reduction	Hold study medication and all other concomitant medications that are potentially hepatotoxic. At least weekly LFT monitoring is required, starting immediately regardless of study schedule and continued until a return to baseline values or Grade 1.
If study treatment resumption is considered for participants who have experienced Grade 3 increases in AST, ALT, or bilirubin with the first dose reduction	Resume study treatment with abiraterone dose reduction to 500mg when AST, ALT or bilirubin returns to baseline value or grade 1.

An opinion from a hepatologist should be considered if there are any concerns or liver function derangement shows no improvement within 2 weeks of discontinuation of abiraterone.

Table 12: Management of fluid retention/oedema associated with abiraterone (given alone or with enzalutamide)

TOXICITY EVENT	ACTION
Grade 1-2	Increase prednisolone dose to 5mg bid.
Grade 3-4	Withhold abiraterone.<br>Consider addition of mineralocorticoid receptor antagonist eplerenone until resolution of symptoms. Enzalutamide can be continued. When fluid retention/oedema returns to baseline or resolves to ≤Grade 1, resume abiraterone at full dose with prednisone 5mg bid, if symptoms do not resolve abiraterone should not be re-started and enzalutamide should be dose reduced to 120 mg per day.

Table 13: Management of diarrhoea (associated with abiraterone or enzalutamide)

TOXICITY EVENT	ACTION
Grade 1-2	Symptomatic management.
Grade 3-4	Withhold abiraterone.<br>If no improvement reduce dose of enzalutamide to 120 mg per day. Once resolved to Grade 1, recommence abiraterone at 750 mg per day.

6.2.4 Research Enzalutamide + Abiraterone + Prednisolone (Arm J)

Note: recruitment has closed to Arm J (SOC + enzalutamide + abiraterone).

Please note that for some participants treatment with enzalutamide + abiraterone may continue until all categories of disease progression or up to a maximum duration of 2 years.

Arm J (SOC + enzalutamide + abiraterone) participants who have now reached their maximum duration of 2 years on trial treatment include:

- N0M0 participants starting treatment over 2 years ago
- N+M0 participants receiving radical radiotherapy and starting treatment over 2 years ago

6.2.4.A Treatment administration

Abiraterone as described in **Section 6.2.2**.

Prednisolone/Prednisone as described in **Section 6.2.2**.

Enzalutamide will be administered as a 160mg oral dose (four capsules), taken together at the same time every day, with or without food.

Trial treatment must stop if other systemic treatments are initiated at any time for disease progression control (including chemotherapy, radium-223 etc).

Anti-androgens (i.e. bicalutamide) should not be given in combination with enzalutamide (as with abiraterone) due to the risk of toxicity; as such participants on, or planned for MAB, at randomisation should not continue with their anti-androgen (AR-antagonists) use if allocated to receive enzalutamide + abiraterone, additionally anti-androgens started whilst on enzalutamide (+abiraterone) treatment should trigger enzalutamide (+abiraterone) to be stopped. See **Table 19**

and Table 20 for further details on drugs that may interact with abiraterone and enzalutamide respectively.

6.2.4.B Treatment duration

In participants with **M1 disease or relapsed previously radically treated disease**, treatment with both abiraterone and enzalutamide will continue until all categories of progression have occurred, consistent with the approach taken for abiraterone (see Section 6.2.2) i.e. abiraterone and enzalutamide will be given until a composite of:

- PSA progression (as defined in Section 7.1.3.A)
- Radiological progression (appearance of new lesions or progression of existing lesions) **and**
- Clinical progression (defined as new cancer-related symptoms).

It is accepted that these flexible criteria for stopping treatment with abiraterone and enzalutamide are open to the investigator's interpretation and discretion. Participants may continue treatment beyond the first failure-free survival (FFS) event; the first FFS event must be reported as per the other arms.

If a participant develops PSA progression only whilst on abiraterone and enzalutamide, the local investigator might consider switching from abiraterone + prednisolone 5mg od to abiraterone and dexamethasone 0.5mg OD.

See Section 7.1.3 for further information on the definition of progression.

In participants with **N0M0 disease or N+M0 disease undergoing radical radiotherapy**, treatment would continue for 2 years or all categories of disease progression as defined for M1 participants, whichever is the sooner. ADT can be discontinued in this group at 2 years at the discretion of the local investigator (see Section 6.1.1).

For participants with **N+M0 disease not planned for radical radiotherapy**, or who do not receive planned prostate RT, treatment will continue as for participants with M1 disease until all categories of disease progression.

For trial purposes the duration of treatment is determined by the intention at the time of randomisation e.g. if a participant with M0N+ disease is initially not planned for radical radiotherapy, the planned duration of treatment to progression should not change if radical radiotherapy is subsequently given. Discuss with the CTU team if you require further advice.

6.2.5 Enzalutamide: Dose Modification & Toxicity Management

Enzalutamide can be taken with or without food.

6.2.5.A Enzalutamide Contraindications

The full induction potential of enzalutamide may not occur until approximately 1 month after the start of treatment, when steady-state plasma concentrations of enzalutamide are reached, although some induction effects may be apparent earlier. Monitoring should continue for at least the first month of treatment and dose adjustments considered. Given the long half-life of enzalutamide (5.8 days), effects on enzymes may persist for one month or longer after stopping enzalutamide. A gradual dose reduction of the concomitant medicinal product may be necessary when stopping enzalutamide treatment. See Table 20 for further details on specific drug interactions with enzalutamide.

6.2.5.B Enzalutamide Special Warnings And Precautions For Use

:: History of seizures

Caution should be used in administering enzalutamide to participants with a history of seizures or other predisposing factors including, but not limited to, underlying brain injury, stroke, primary brain tumours or brain metastases or alcoholism. In addition, the risk of seizure may be increased in participants receiving concomitant medications that may lower the seizure threshold. Enzalutamide should be **permanently discontinued** in participants who have a seizure while on treatment.

:: Hepatic impairment

A hepatic impairment study showed that the composite AUC of enzalutamide plus N-desmethyl enzalutamide after administration of a single dose of enzalutamide was similar in patients with baseline mild, moderate or severe hepatic impairment (Child-Pugh Class A, B or C respectively) relative to patients with normal hepatic function, and no starting dose adjustment is needed.

:: Renal impairment

No dose adjustments are required in renal impairment; however caution is advised if participants develop severe renal impairment as there is limited clinical data in this population.

6.2.5.C Enzalutamide Overdose

There is no antidote for enzalutamide. In the setting of an overdose, stop treatment with enzalutamide and initiate general supportive measures taking into consideration the half life of 5.8 days. Participants may be at increased risk of seizures following an overdose.

6.2.5.D Management Of Specific Toxicities Due To Abiraterone And Enzalutamide

The safety monitoring and toxicity management plan described below takes into account AEs based on the reported clinical safety data of abiraterone and enzalutamide given separately. There are limited reported data on the safety and toxicity of the combination of enzalutamide and abiraterone however the recommendations summarised here have been updated in light of the experience gained in STAMPEDE as recommended by the STAMPEDE TMG.

:: Seizures

If any participant suffers a seizure whilst on treatment, enzalutamide should be permanently discontinued immediately. Abiraterone and prednisolone can be continued providing there are no abiraterone-specific toxicities.

Table 14: Management of arthralgia & muscle pain (associated with enzalutamide)

TOXICITY EVENT	ACTION
Grade 1-2	Symptomatic management
Grade 3-4	Reduce dose of enzalutamide to 120 mg /day

Table 15: Management of fatigue (associated with enzalutamide)

TOXICITY EVENT	ACTION
Grade 1-2	Consider a dose reduction to 120 mg/day
Grade 3	**Pause enzalutamide** for 1 week or until the toxicity grade improves to grade 2 or lower severity. Re-started at a reduced dose (120mg/day or 80mg/day) in consultation with the study team.

6.2.6 Metformin: Administration, Dose Modifications And Management Of Toxicities

6.2.6.A Required timelines to start metformin

For all participants allocated to arm K, metformin should start as soon as possible after randomisation and within a maximum of 4 weeks. The CTU should be contacted if it is not possible to adhere to this required timeline. Metformin can be given in combination with SOC docetaxel or SOC abiraterone.

6.2.6.B Metformin administration

Metformin will be given as a daily dose in addition to standard-of-care treatment. The target dose is **850mg Std BID.**

The starting dose for metformin is 850mg once daily. If tolerated this should be increased to the target dose after 4-6 weeks i.e. at the first follow-up visit.

Metformin should be taken around the same time each day and treatment tolerance is best if taken with or after food. For twice daily dosing, the minimum time between doses should be 8 hours, doses should not be taken closer together if forgotten or missed. If metformin is well tolerated and it is desirable to make a dose modification outside of the trial follow-up schedule, it is acceptable to conduct a telephone consultation.

6.2.6.C Metformin treatment duration

In the case of **M0 participants**, if ADT is stopped after a minimum of 2 years, metformin should continue for a **minimum of 3 years** following randomisation and for a further **12 months after the administration of the last LHRH** (whichever is longer). This is to allow for the delay in testosterone levels returning to normal following stopping ADT.

If ADT is not stopped, then metformin should continue as for M1 participants. In the event that ADT is stopped and then re-started for relapsed disease, if ADT is restarted whilst participants remain on metformin (i.e. within 12 months of the last administration of LHRH) then metformin should continue whilst on ADT. If metformin is stopped 12 months after the last administration of LHRH it should not be re-started in the event of relapse.

For **M1 participants** metformin should continue whilst on ADT. **Treatment should continue post-progression** providing it is judged to be in the participant's best interest. Metformin can be given together with any additional treatments started for progression, excluding other IMPs i.e. investigators may choose to stop metformin treatment post-progression in order to enable participants to participate in another clinical trial evaluating treatments for CRPC.

In the event research treatment is stopped, unless a participant states otherwise, consent is assumed for continued recording trial data.

6.2.6.D Metformin dose modifications

If metformin 850mg Std BID is not well tolerated, the following dose reductions should be implemented:

- 500mg SR BID
- 750mg SR BID
- 500mg Std BID
- 500mg SR OD
- 750mg SR OD
- 500mg Std OD
- 850mg Std OD

6.2.6.E Metformin Special Warnings And Required Monitoring Whilst on Treatment

:: Renal impairment

Metformin is not nephrotoxic, but is exclusively excreted by the kidneys. Therefore treatment should only be started in participants with stable renal function. From protocol v16.0 the renal threshold has been revised in light of updated FDA guidance and published prescribing recommendations. Metformin should be only started when the GFR ≥45ml/min/1.73m^2. Renal function should be monitored **at least every 6 months** in participants with stable renal function, whilst on metformin.

Additional monitoring is required in any participant at risk of deteriorating renal function. In line with published prescribing recommendations, if the GFR falls to between 30-45 ml/min/1.73m^2 a **dose reduction** is required to ensure the maximum 24hr dose is 1000mg or less and monitoring of renal function is required at least 3 monthly (81).

Metformin should be **permanently stopped** if the GFR falls to ≤30ml/min/1.73m^2.

See **Table 16** for situations when metformin treatment should be paused due to the risk of deteriorating renal function.

Table 16: Situations when metformin treatment should be paused

SITUATIONS	RISK FACTOR
Iodinated contrast agents	If the GFR<60 ml/min/1.73m^2 metformin should be paused for 24 hours prior to receiving contrast and re-started 48 hours post-administration. (82)
Anaesthesia (peridural; spinal or general)	Pause metformin 48 hours prior to procedure and re-start no earlier than 48 hours following procedure, providing oral intake re-established and renal function is stable and at baseline.
Surgery	Pause metformin 48 hours prior to procedure and re-start no earlier than 48 hours following procedure, providing oral intake re-established and renal function is stable and at baseline.
Dehydration<br>e.g. nausea, vomiting or diarrhoea	Pause metformin and re-start only when oral intake is re-established and renal function is stable and at baseline.
Obstructive uropathy<br>e.g. urinary retention or ureteric obstruction	Pause metformin and re-start only when renal function confirmed to be stable and at baseline.

:: Treatment breaks

It is anticipated that metformin treatment will be paused for approximately 72 hours around the time of contrast enhanced CT scans (see **Table 16**) and may need to be paused during episodes of

inter-current illness. If metformin is paused for 6 days or less this information does not need to be recorded and no additional action is needed. Treatment pauses of 7 days or more should be recorded by updating the Metformin Treatment Log.
If metformin treatment is paused for more than 2 weeks, investigators may consider re-starting at 850mg once daily for the first 4 weeks before escalating to full dose providing tolerance is acceptable. It is suggested that, providing participants have a sufficient supply of labelled IMP metformin tablets, a telephone consultation may be sufficient to assess tolerance and advice regarding dose modification in order to limit hospital visits.

If treatment is paused for more than 3 months or >50% of doses are missed for any reason the trial team should be informed as metformin may need to be discontinued.

6.2.6.F Management Of Specific Toxicities From Metformin

:: Gastrointestinal disturbance

Gastrointestinal disturbances are very common with metformin and include nausea, vomiting, diarrhoea, abdominal pain and loss of appetite. These are most common when first starting treatment (occur in >1/10 individuals).

If toxicities occur we recommend a dose reduction and/or a switch to a sustained release (SR) preparation if available (see **Table 17**).

Table 17: Management of metformin related gastrointestinal toxicity

Toxicity event	Action
Grade 1	• Ensure metformin is taken with or after food.<br>• Consider switching to 750 mg BID SR preparation if available.<br>Or, if unavailable consider:<br>• 1 week treatment pause, re-start at reduced dose 850mg once daily. Attempt an escalation after 1 month but if necessary, remain at 850mg OD.<br>And, if unable to tolerate 850mg OD or sustained release preparations are not available<br>• Consider dose reduction to 500mg OD (SR or IR if not available)
Grade 2<br>or higher	Reduce to 500mg OD SR (or IR if not available); re-attempt dose escalation after minimum of 1 week if symptoms improve, aiming to continue at the maximum tolerated dose<br>If grade 2 toxicity persists consider the following:<br>• Pause treatment for 2 weeks, and re-start at 850mg sustained release or if not available 500mg OD.<br>• And re-attempt a dose escalation 2 months later.<br>• And continue at the maximum tolerated dose providing symptoms ≤ grade 1.

If toxicities persist, consider a 1 week treatment pause, before re-starting at one of the dose-modified regimes and attempt an dose escalation after a minimum of 1 month. See **Section 6.2.6.D** for all recommended dose reductions.

Other possible metformin related toxicites included taste disturbance, skin reactions and B12 deficiency resulting in megaloblastic anaemia (see **Appendix C**).If a participant becomes anaemic whilst taking metformin, investigators should consider measuring haematinics including vitamin B12 measured and replace if deficient.

:: Lactic acidosis

This is a very rare (3/100,000 patient years), but serious metabolic consequence. Reported cases have occurred primarily in diabetic patients with significant renal impairment who are also dehydrated. It is unclear whether this is due to the underlying diabetes or metformin. This is supported by a meta-analysis demonstrating similar rates of lactic acidosis in people with diabetes taking metformin compared with diabetic participants not taking metformin (83). This evidence suggests this side effect may be a complication of diabetes and may not be associated with metformin treatment. The risk factors for lactic acidosis are: renal impairment, prolonged fasting or malnutrition, excessive alcohol intake, hepatic insufficiency or any condition associated with hypoxia e.g. cardiac or respiratory failure or circulatory shock due to any cause.

The risk of lactic acidosis should be considered in the event of non-specific symptoms such as muscle cramps, abdominal pain and/or severe weakness or lethargy. Any participant with a suspected metabolic acidosis requires immediate discontinuation of metformin and evaluation. Lactic acidosis is characterised by metabolic acidosis (decreased blood pH, high lactate above 5mmol/L and an increased anion gap and lactate/pyruvate ratio). The most effective way to remove lactate and metformin is haemodialysis.

:: Metformin overdose

Hypoglycaemia has not been reported with even significant metformin overdoses although lactic acidosis has occurred in such circumstances. Participants should be urgently assessed in the event of an overdose and hospital admission considered. The management of metformin overdoses should be as per standard clinical care by the local team. The most effective way to remove lactate and metformin is haemodialysis.

6.2.7 Research Transdermal Oestradiol (Arm L)

For all participants allocated to transdermal oestradiol, treatment should start as soon as possible after randomisation (and ideally within 1 week after randomisation). It is not necessary to wait for completion of the 4-week (or 1-month) duration of the LHRH injection if this was previously given prior to randomisation. For those prescribed bicalutamide or flutamide prior to randomisation, this treatment should be discontinued before treatment with transdermal oestradiol can commence (no washout period is needed). If SOC docetaxel has not been started before randomisation, it is recommended that it is started *after* participants have been established on transdermal oestradiol for around 4 weeks (when most will have completed the induction period).

Participants randomised to receive transdermal oestradiol may also receive SOC radiotherapy (**Section 6.1.2**) as clinically appropriate, as has been done in the PATCH trial. Participants randomised to the transdermal oestradiol comparison are not eligible to receive SOC abiraterone.

Transdermal oestradiol is delivered as Progynova TS 100mcg/24 hours transcutaneous oestradiol patches according to the following dose regimen which has been shown within the PATCH trial to be sufficient for achieving castrate levels of testosterone.

6.2.7.A Induction Regimen

Four transdermal transdermal oestradiol patches to be changed twice weekly (e.g. Monday and Thursday) for four weeks. A confirmatory testosterone and oestradiol sample should be taken at 4 weeks with the sample drawn the **day before** the patches are changed.

6.2.7.B Maintenance Regimen

If the participant has achieved a testosterone value of ≤1.7nmol/L at 4 weeks then treatment is changed to a **maintenance regimen** of **three** patches changed twice weekly. The oestradiol level

should also be monitored at the 4 week time point, with castrate levels of testosterone typically achieved with a plasma oestradiol level ≥500pmol/L.

If a participant's testosterone is >1.7nmol/L at four weeks then they should remain on the induction regimen for another 4 week period, with monitoring of testosterone and oestradiol samples taken at around the week 8 time point, the day before patches are changed. Once the participant achieves a castrate level of testosterone ≤1.7nmol/L, they can be reduced to the maintenance regimen.

6.2.7.C Monitoring Hormone Levels

Oestradiol and testosterone levels should continue to be monitored throughout follow-up, while the participant remains on transdermal oestradiol treatment, to assess for evidence of compliance and to also ensure the participant is on the appropriate dose. See **Section 7.1.2**. and **Table 2** for when these values are required, noting also that the samples can be taken at the same time as scheduled PSA measurements.

A repeat blood test should be carried out within 4 weeks if, at any time, the participant's oestradiol level is found to be <300pmol/L or >2000pmol/L or the testosterone level is >1.7nmol/L while on the maintenance regime, with particular attention paid to the day that the patches are changed compared to when the blood sample is drawn (should be the day before changing patches). If the participant continues to have out of range oestradiol levels, and/or persistent testosterone >1.7nmol/L, then a member of the CTU team should be contacted for advice.

If the maintenance patch dose is changed at any time (for example, reducing from 3 to 2 patches changed twice weekly), then additional oestradiol and testosterone tests are required around 4 weeks after dose modification.

The changing of patch brand should be avoided unless absolutely necessary (see **Appendix I.3**) but if advised by the CTU trial team, then additional oestradiol, testosterone and PSA tests are required following the change (see **Table 18**). It is important that participants are then monitored in real-time during this initial period, with the CTU team to be contacted if the hormone results are out of range as it may be necessary to modify the dose regimen.

Table 18: Additional assessments required following change of maintenance patch dose or brand

ASSESSMENTS REQUIRED	WEEKS FROM DOSE MODIFICATION OR CHANGE IN PATCH BRAND*			
	WEEK 0 (PRIOR TO CHANGE)	WEEK 4 (POST CHANGE)	WEEK 8 (POST CHANGE)	WEEK 12 (POST CHANGE)
	**Change of maintenance dose**			
OESTRADIOL<br>TESTOSTERONE<br>PSA	X	X		
	**Change of patch brand**			
OESTRADIOL,<br>TESTOSTERONE<br>PSA	X	X	X	X**

* These additional tests are timed from the day of dose modification or day of starting new patch brand. However, if the post-change tests coincide within 1 week of scheduled tests (see **Table 2**), it is not necessary to repeat the tests.
** Dependent on prior results, 12 week test may be requested by CTU trial team

6.2.8 Transdermal Oestradiol: Administration, Dose Modifications And Management Of Toxicities

Consecutive patches should be applied to different sites. It is recommended that patches are placed on dry, intact and hairless skin and on areas where little wrinkling occurs, at the following sites only:

- Shoulder girdle
- Back
- Upper arms
- Buttocks

Patches should not be placed on or near the breast area, or on areas of the body where there are large amounts of subcutaneous fat, particularly around the abdomen, as this could affect absorption. Please note that these recommendations are mainly based on studies in women using the patches.

To apply the patch, remove the protective liner and press on to the skin immediately, holding for at least 30 seconds to ensure proper adhesion. If necessary, tape can be used to fix the patch in place. If applied correctly, the participant can bath or shower as normal, however the patches might come off in very hot water or in a sauna.

Dermatitis can be a common side-effect of using the patches, especially in the induction period, which can usually be controlled by alternating the site of patch application. Participants should be advised that if patches become dislodged they should not put on extra patches, but apply their next set of patches when they are next due to be applied.

Prophylactic irradiation of the breast area, shown to reduce risk of gynaecomastia is permitted: a single fraction of 8Gy is recommended preferably before treatment with transdermal oestradiol (84).

We expect participants to remain on the prescribed dose, and any potential dose modifications other than those indicated **in Section 6.2.7** should be discussed with CTU team. If a participant has a cardiovascular event (see **Section 7.1.4.A**), discontinuation of treatment with transdermal oestradiol may be considered at the discretion of the treating clinician.

6.3 CONCOMITANT TREATMENTS AND DRUG INTERACTIONS

All concomitant medications should be continued throughout the trial unless the responsible clinician decides otherwise. If participants continue to require medication for the management of docetaxel-related toxicities, please discuss this with the CTU team.

6.3.1.A Data collection on concomitant treatments for participants in arms A, G, H, J, K, L

Long-term (>6 months) use of the following concomitant medications of classes of interest is collected:

- Statins
- Metformin
- Aspirin
- Bisphosphonates or denosumab
- Opiate pain killers
- NSAIDs
- ACE inhibitors or angiotensin II antagonists

This information is of interest both in terms of baseline use and ongoing use through the trial; as such it should be recorded on the Randomisation CRF and will be collected at each follow-up assessment (see **Table 1** and **Table 2)**.

6.3.2 Abiraterone: Interaction With Other Medicinal Products

Details on drug interactions are described in **Appendix C** and **Table 19** provides a summary on the main interactions.

:: Anti-androgens
Abiraterone is steroid synthesis inhibitor and should **not** be given together with any other anti-androgens given the risk of toxicity. Cyproterone acetate should be discontinued 10 days and finasteride stopped 48 hours before commencing abiraterone. Concomitant use of dutasteride, bicalutamide, flutamide and tamoxifen are all **contraindicated.**

:: Spironalactone
Spironolactone binds to the androgen receptor, may increase prostate specific antigen (PSA) levels and is associated with abiraterone resistance therefore concomitant use is **contraindicated.**

Myopathy has occurred in patients treated with abiraterone, typically this occurs when first initiating treatment and resolves when abiraterone is stopped. Caution is recommended in participants receiving concomitant treatments with medicinal products known to be associated with myopathy/rhabdomyolysis e.g. statins.

6.3.3 Enzalutamide: Interaction With Other Medicinal Product

Details on drug interactions are described in **Appendix C** and **Table 20** provides a summary on the main interactions.

:: Anti-androgens
Enzalutamide is potent androgen receptor antagonist and should **not** be given together with any other anti-androgens given the risk of toxicity. Cyproterone acetate should be discontinued 10 days and finasteride stopped 48 hours before commencing enzalutamide. Concomitant use of dutasteride, bicalutamide, flutamide and tamoxifen are all **contraindicated.**

Table 19: Drugs that may interact with abiraterone

DRUGS WHICH MAY REDUCE ABIRATERONE LEVELS			
**Substrate**	**Clinical Use**	**Drug**	**Recommendation**
CYP3A4	Anti-epileptics*	Phenytoin<br>Carbamazepine<br>Phenobarbital<br>Primadone	Contraindicated
	Anti-depressants	St Johns Wart	Contraindicated
	Anti-TB	Rifampicin<br>Rifabutin	Contraindicated
	Anti-retroviral	Atazanavir<br>Saquinavir<br>Ritonavir<br>Indinavir<br>Nelfanavir	Contraindicated. Seek specialist advice and discuss with trial team
**DRUGS WHICH MAY ACCUMULATE WHEN GIVEN WITH ABIRATERONE**			
**Substrate**	**Clinical Use**	**Drug**	**Recommendation**
CYP2D6	Cardiac	Metoprolol<br>Propranolol<br>Propafenone<br>Flecanide	Monitoring required as drug levels may increase with abiraterone use
	Anti-depressants	Desipramine<br>Venlafaxine<br>Citalopram	Monitoring required as drug levels may increase with abiraterone use
	Anti-psychotics	Haloperidol<br>Risperidone	Monitoring required as drug levels may increase with abiraterone use
	Analgesia	Tramadol<br>Codeine<br>Oxycodone	Monitoring required as drug levels may increase with abiraterone use
	Alpha blockers	Tamsulosin	Monitoring required as drug levels may increase with abiraterone use
	Anti-diabetic	Repaglinide	Monitoring required as drug levels may increase with abiraterone use

Table 20: Drugs which may interact with enzalutamide

DRUGS WHICH MAY INCREASE ENZALUTAMIDE LEVELS			
**Substrate**	**Clinical Use**	**Drug**	**Recommendation**
CYP2C8 inhibitors	Lipid-lowering	Gemfibrozil	Avoid, if no alternatives, reduce enzalutamide dose to 80mg
**DRUGS WHICH MAY DECREASE ENZALUTAMIDE LEVELS**			
**Substrate**	**Clinical Use**	**Drug**	**Recommendation**
CYP2C8 inducers	Anti-TB	Rifampicin<br>Rifabutin	Avoid and switch to an alternative if possible
CYP3A4 inducers	Anti-epileptics	Phenytoin<br>Carbamazepine<br>Phenobarbital	Contraindicated
	Anti-depressant	St Johns Wart	Contraindicated
	Anti-retrovirals	Atazanavir<br>Saquinavir<br>Ritonavir<br>Indinavir<br>Nelfanavir	Contraindicated. Seek specialist advice and discuss with trial team
**ENZALUTAMIDE MAY REDUCE DRUG LEVELS**			
**Substrate**	**Clinical Use**	**Drug**	**Recommendation**
CYP2C19	Gastric protection	Omeprazole	Omeprazole AUC reduced by 70%<br>Consider increasing dose of omeprazole for same therapeutic effect
CYP3A4	Analgesia	Fentanyl*<br>Alfentanil*<br>Tramadol	Monitor closely and consider alteratives
	Immunosuppressants	Sirolimus*<br>Tacrolimus*<br>Cyclosporine*	Monitor closely
	Anti-migraine	Ergotamine	Monitor closely
	Cardiac	Nifedipine<br>Ivabradine	Monitor closely, consider alternatives as clinical effect may be reduced
CYP2C9	Anti-epileptics	Phenytoin*	Contraindicated
	Anti-coagulants	Warfarin*	Warfarin AUC reduced by 56%<br>Consider switching to low molecular heparin, increase INR monitoring if this is not possible
**DRUGS WHICH MAY ACCUMULATE WHEN GIVEN WITH ENZALUTAMIDE**			
**Substrate**	**Clinical Use**	**Drug**	**Recommendation**
p-gp		Colchicine*<br>Dabigatran*<br>Digoxin*	Monitor closely

*narrow therapeutic index

6.3.4 Metformin: Interaction With Medicinal Products And Other Forms Of Interaction

Caution is needed when initiating potential nephrotoxic drugs as metformin is renally excreted therefore may accumulate if renal function deteriorates, please refer to Table 21 for more information on drugs which may require additional monitoring of renal function.

Metformin does **not** interact with any of the other treatments for prostate cancer and **should be continued** during all further treatments given for disease progression.

As metformin is being given as an IMP in the context of a clinical trial, continued use will not be permitted if participants participate in other interventional clinical trials for prostate cancer (i.e. CRPC setting). Investigators should use their discretion and discuss discontinuing metformin with the trial team if it is felt to be in the participant's best interest.

Table 21: Drugs which require additional monitoring of renal function

Clinical use	Drug	Recommendation
Anti-hypertensives and other cardiac disease	ACE inhibitors/angiotension II receptor blockers<br>e.g. ramipril, lisinopril, Irbesartan	Increased frequency of renal function monitoring until confirmed to be stable
	Diuretics<br>e.g. Furosemide, bumetanide	
Antibiotics	Aminoglycoside antibiotics<br>e.g. Gentamicin or amikacin	Hold metformin during treatment<br>Re-start once treatment complete
Analgesia	NSAIDS<br>e.g. Ibuprofen, diclofenac, naproxen	Avoid if possible<br>If used increased frequency of renal function monitoring is required until confirmed to be stable

If the renal function declines to eGFR<45ml/min/m^2 a dose reduction is required and the frequency of monitoring of renal function must increase to a minimum of 3-monthly, see Section 6.2.6.D.

6.3.5 Transdermal Oestradiol: Drug Interactions

Tamoxifen should not be prescribed for participants receiving transdermal oestradiol. The metabolism of oestrogens may be increased by concomitant use of substances known to induce drug metabolising enzymes, specifically cytochrome P450 enzymes, such as anticonvulsants (e.g. phenobarbital, phenytoin, carbamezapine) and anti-infectives (e.g. rifampicin, rifabutin, nevirapine, efavirenz). Ritonavir and nelfinavir, although known as strong inhibitors, by contrast exhibit inducing properties when used concomitantly with steroid hormones. Herbal preparations containing St. John's wort (Hypericum Perforatum) may induce the metabolism of oestrogens.

With transdermal administration, the first-pass effect in the liver is avoided and, thus, transdermally applied oestrogens might be less affected than oral hormones by enzyme inducers. Oestradiol levels are already monitored as part of trial follow-up while participants are on transdermal oestradiol. As oestradiol levels are already monitored as part of trial follow-up while participants are on transdermal oestradiol, no additional monitoring is required.

6.4 TRIAL PRODUCTS

Details of the procedures for obtaining the drugs within the trial, dispensing and disposal of unused drug are given in **Appendix B**. Arrangements for free or discounted drugs are given in the Finance section (**Section 15**).

6.5 TREATMENT DATA COLLECTION

Data will be recorded on case report forms (CRFs); the top copy/original should be sent to CTU for data entry and a copy kept at the local centre. Up-to-date versions of all CRFs can be found on the trial website (http://www.stampedetrial.org/) and centres will be notified of any changes throughout the course of the trial. The type of data to be recorded is detailed in the Assessments and Procedures section (**Section 7**).

6.6 MEASURES OF COMPLIANCE/ADHERENCE

Date of treatment, dose, delays and reasons for delays or dose modifications of all trial treatments will be recorded. The estimated number of abiraterone tablets, enzalutamide capsules or metformin tablets taken in a given time period will also be recorded as well as any dose reductions. See **Table 30** for a description of the treatment logs.

Oestradiol levels will be collected for participants in the transdermal oestradiol arm and used to assess compliance to treatment (see **Section 6.2.8**).

Evidence of compliance with safety monitoring is required for participants on research abiraterone e.g. potassium and LFTs, or metformin treatment e.g. renal function, as described in **Section 6.2.3.B** and **Section 6.2.6.D**. Site investigators should document in the participant's medical records the date of the blood test or review of blood pressure measurements and confirmation that the results were known to be within acceptable limits and if not, the toxicity should be graded according to CTCAE and the action described. This should be available at on-site monitoring visits and used to verify the information provided on the follow-up CRF and treatment logs.

Note, safety monitoring for SOC abiraterone is as per local practice and compliance data is not required by the trial.

6.7 ADMINISTRATION OF STANDARD RADIOTHERAPY TO M0 PARTICIPANTS

6.7.1 Treatment Details

Standard radiotherapy will be given to appropriate participants in each of the trial arms, following a period of neo-adjuvant ADT therapy, as is generally standard in UK practice. For participants with negative nodes on axial imaging, clinicians may choose between irradiating prostate and seminal vesicles alone or including the pelvic nodes in addition. Additional staging tests such as pelvic node sampling may be considered in making this decision. Conformal or intensity modulated radiotherapy should be used in all participants. Where participants have good clinical evidence that nodes are free of tumour or participants for whom nodal radiotherapy is contra-indicated (e.g. significant bowel disease), treatment may be given to the prostate gland and seminal vesicles only. The recommended dose is 74Gy in 37 fractions to the prostate and seminal vesicles or the equivalent using hypo-fractionated schedule, 60Gy in 20 fractions. Alternative dosing schedules are permitted but must be agreed with the STAMPEDE Trial Management Group.

6.7.1.A Standard-Of-Care RT Timing In M0 participants

If receiving docetaxel as part of the standard-of-care (permitted from protocol v14.0), the participant must have sufficiently recovered from any docetaxel toxicity before RT can begin. In all other participants not receiving SOC docetaxel, SOC RT may be started sooner (2-6 months post-randomisation) consistent with the data from the MRC PR07 trial (12).

7 ASSESSMENTS AND PROCEDURES

7.1 SCHEDULE FOR ASSESSMENTS

7.1.1 Treatment & Follow-up Schedules

An individualised form with a follow-up schedule will be provided for each randomised participant following receipt at the CTU of the patient consent form and Randomisation Eligibility Checklist. Which follow-up schedule applies depends on which comparison the participant was randomised to as summarised in Table 22.

Table 22: Summary of follow-up schedules by participant group

COMPARISON	PARTICIPANT DETAILS	FOLLOW-UP SCHEDULE
"Original"	Arms B, C, D, E, F and Arm A recruited between trial start (2005) and 15-Nov-2011	Active follow-up to be discontinued in Q3 2018
"Abiraterone"	Arms A and G randomised between 15-Nov-2011 and 17-Jan-2014	See **Table 1**
"Abiraterone and enzalutamide"	Arms A and J randomised between 27-Jun-2014 and 31-March 2016	See **Table 1**
"M1|RT"	Arms A and H randomised between 30-Jan-2013 and 02-Sep-2016	See **Table 2**
"Metformin"	Arms A and K randomised since 05-Sep-2016	See **Table 2**
"Transdermal oestradiol"	Arms A and L randomised since 20-Jun-2017	See **Table 2**

7.1.2 PSA, Testosterone And Oestradiol Measurements

All participants should have PSA measured prior to starting ADT and at every subsequent trial follow-up visit, regardless of allocated treatment arm. For participants who do not have a scheduled hospital visit, it is acceptable for arrangements to be made for blood samples to be drawn at their GP surgery.

For arm L participants, oestradiol and testosterone levels should continue to be monitored while the participant remains on transdermal oestradiol treatment, for when these measurements should be obtained. Note, the first follow-up visit post-randomisation can be scheduled at 4 instead of 6 weeks to coincide with the 4-week hormone tests (see **Section 6.2.7**). These samples could be taken at the same time as the PSA tests, unless additional tests are required as detailed in **Sections 6.2.7**. It is also preferable for samples to be taken the day before the oestradiol patches are changed, to allow consistent measurements of testosterone and oestradiol with respect to the pharmacokinetic profile of the patches.

7.1.3 Assessment Of Treatment Failure (Definition Of Progression)

All participants should have baseline radiological examinations as detailed in **Section 4.2.1.** Participants are not routinely assessed for response. However, in order that objective progression

can be assessed, it is necessary to have imaging taken at time of best response as judged by the treating clinician. The frequency of imaging is at the discretion of the treating clinician.
The following outcomes should be reported on the Progression log:

- Biochemical failure
- Local progression
- Lymph node progression
- Progression or development of new distant metastases, defined as lymph nodes outside the pelvis, bone or organ involvement
- Skeletal-related events confirmed as progression (see below)

7.1.3.A Biochemical Failure

For the purposes of the STAMPEDE trial, a unique threshold PSA value for biochemical failure is calculated, referred to as the **PSA progression value**.

This value is derived for each participant based on their **PSA nadir,** defined as the lowest PSA value reported between *randomisation* and 24 weeks on trial. Please refer to the PSA progression value calculator on the STAMPEDE website.

The exact method for deriving the progression value for a participant depends on the value of their PSA nadir, and how this compares to their pre-treatment PSA value (i.e. the extent of the fall in PSA from the starting point).

The PSA progression value is calculated in one of three ways:

A. If the lowest recorded PSA value in the 24 weeks following randomisation is more than 4ng/ml and more than 50% of the pre-treatment PSA level then the participant fulfils the criteria for immediate treatment failure.

B. For participants whose PSA nadir in the 24 weeks following randomisation is less than or equal to 50% of the pre-treatment PSA level but remains above 4ng/ml, biochemical failure will be defined as a rise of 50% above the nadir level.

C. For participants whose PSA nadir is less than or equal to 4ng/ml, biochemical failure is defined as at least a 50% rise above the nadir value that is also above 4ng/ml.

Confirming biochemical failure: the timing of assessments needs to be considered because spurious rises in PSA can occur e.g. following procedures involving the urinary tract. For this reason, any isolated rise in PSA should be confirmed before reporting biochemical failure.

In the case that the raised PSA value reaches the progression value, a confirmatory PSA test should be performed between one week and 3 months later. Biochemical failure is confirmed if the second value is around the same level or higher i.e. the trend is confirmed. The date of PSA progression should be provided as the date of the **first** raised PSA that fulfilled the trial definition of progression. Only the first instance of biochemical failure needs to be reported.

A confirmatory PSA is not required if there are other signs of progression e.g. progression of cancer related symptoms (clinical progression) or new radiological progression.

Second-line treatment commenced specifically for biochemical failure should not start until the trial definition for biochemical failure has been met. However, if second-line treatment does start before the trial definition is met then report the closest PSA value prior to the treatment start date as the

progression value. This is not required if second-line treatment is being started for other signs of progression e.g. clinical or radiological.

Testosterone levels: are only required when reporting biochemical progression whilst receiving hormone treatment to confirm the diagnosis of castrate-resistant prostate cancer. Testosterone levels are not required when reporting biochemical progression in participants not receiving hormone therapy e.g. participants who presented with non-metastatic disease have relapsed following completion of treatment.

See **Appendix E** for further details on the trial definition of biochemical failure.

7.1.3.B Local, Lymph Node And Metastatic Failure

For each of local, lymph node and distant metastases progression, **both of** the following should be reported:

- Date of first clinical/symptomatic progression
- Date of first objective/radiological progression

7.1.3.C Skeletal-related Events

- Pathological Fracture
- Spinal cord compression
- Requirement for RT to bone (e.g. for pain or impending fracture)
- Requirement for surgery (e.g. for prevention or management of fracture)

SREs are a secondary outcome measure and a disease event of interest. SREs may represent disease progression but can also occur due to treatment-related effects e.g. osteoporotic fracture due to treatment-related bone-mineral density loss. All SREs should be recorded on the Follow-up form.

All SREs should be investigated further to establish whether or not the participant has progressed and, if confirmed as progression, a Progression Log should be completed to record this along with an Additional Treatment Log to give details of any treatment received (e.g. palliative RT or surgery). The summary of timing of Case Report Forms can be viewed in **Table 23** and **Table 24**.

7.1.3.D Objective/Radiological progression

Investigator determined radiological progression should be reported. For specific comparisons it may be necessary to centrally review baseline and progression scans e.g. CT scans and bone scans. Requests for scans will be made if and when these are required for a proportion of relevant participants and processes put in place for electronic transfer and site reimbursement.

7.1.4 Additional Metabolic And Cardiovascular Outcomes

A number of metabolic and cardiovascular (CVS) outcomes are being assessed in the “metformin comparison” and “transdermal oestradiol comparison” as outlined below. From protocol v17.0 onwards, a metabolic profile (lipids, glucose and HbA1c) will be measured for all participants randomised from 05-Sep-2016 onwards to capture data on metabolic and cardiovascular outcomes for both comparisons. See **Table 2** for a schedule of assessments, please note it is permitted to obtain these measurements within 12 weeks of the scheduled follow-up visit. The summary of timing of Case Report Forms can be viewed in **Table 23** and **Table 24**.

7.1.4.A Cardiovascular Outcomes: Transdermal Oestradiol Comparison

Cardiovascular morbidity and mortality was the primary outcome measure for the first stage in the PATCH trial (completed in 2010), which showed similar rates of CVS events in participants receiving transdermal oestradiol compared to those receiving LHRH injections (54). These results have been

confirmed by longer-term data within the trial (see **Appendix I**). Continued monitoring of CVS outcomes will be undertaken by the PATCH IDMC for both the PATCH trial, as well as for the participants in STAMPEDE allocated to transdermal oestradiol together with their contemporaneous controls.
While Arm L participants are undergoing treatment with transdermal oestradiol, the majority of these CVS events will fall under the definitions of Serious Adverse Events (see **Section 11**). Once a participant has a cardiovascular event, the discontinuation of treatment with transdermal oestradiol may be considered at the discretion of the treating clinician and the participant switched to standard of care hormone therapy.

An increased risk of venous thromboembolism has been observed when docetaxel is used in combination with certain agents for the treatment of prostate cancer. Therefore, the rate of CVS events will be closely monitored among participants within Arm L who are receiving docetaxel as part of their first-line treatment. For more details see **Appendix I**. However, within the PATCH trial (based on data up to 17-Sep-2017), no cardiovascular endpoint events had been reported among participants on transdermal oestradiol receiving upfront docetaxel.

7.1.5 Additional Safety Assessments

The comparison specific follow-up schedules are summarised in **Table 1** and **Table 2.**
These summarise all the required additional safety assessments that are required whilst participants are receiving research treatments: abiraterone, metformin and transdermal oestradiol..
All safety assessments are required until research treatments have been permanently stopped for more than 30 days.

The summary of the timing of Case Report Forms also can be viewed in **Table 23** and **Table 24**.

7.1.5.A Additional Safety Assessment: Research Abiraterone

Due to the risk of liver toxicity and secondary hyperaldosteronism with abiraterone, all participants require regular monitoring of **potassium, liver function tests and blood pressure** whilst receiving research abiraterone. Monitoring should be performed 2-weekly in the first 12 weeks of treatment, then **monthly until 12-months on treatment** and then, providing treatment is well tolerated, **2-monthly thereafter** whilst treatment with research abiraterone continues, see **Section 6.2.3.B.**

Investigators are responsible for ensuring blood tests are performed at the required frequency and need to review and document the results. It is acceptable for blood pressure to be self-monitored at the required frequency by trial participants or via the GP, providing this is reviewed at each follow-up by investigators.

Confirmation that potassium and liver functions test have been performed regularly and blood pressure control reviewed will be required at each follow-up visit. Any abnormalities should be graded according to CTCAE version 4.0 and recorded on the toxicity section of the follow-up CRF; any abnormalities fulfilling the criteria for a SAE (e.g. requiring hospital admission) should also be reported on a SAE CRF (see **Section 11**).

Please note, the protocol guidance relates to research abiraterone i.e. treatment received by participants allocated to arms G and J. This may be used as a guide when using SOC abiraterone, but investigators should adhere to local practice. STAMPEDE data collection relating to suspected abiraterone related toxicities is limited to research abiraterone only.

7.1.5.B Additional Safety Assessment: Metformin

Participants with normal and stable renal function receiving metformin require monitoring of **renal function (U&Es) every 6 months** whilst on treatment. More frequent monitoring is required in

participants with declining renal function, or when initiating new potentially nephrotoxic medications or at times of intercurrent illness (see Section 6.2.6.D). Changes in renal function (eGFR, graded according to CTCAEv.4) are recorded on the Follow-up CRF. It is acceptable for blood sampling to be arranged via the GP at the participant's home or local hospital.

7.1.5.C Additional Safety Assessment: Transdermal Oestradiol

Hormone levels are monitored while participants are on transdermal oestradiol, and if oestradiol levels are found to be >2000pmol/L with confirmed repeat test, please contact CTU for advice (see Section 6.2.7.C).

7.2 DATA COLLECTION PROCEDURES

Treatment-related data are collected on Treatment Specific Logs. It is important that **all** treatments given for progressive disease are recorded on the Additional Treatment Log. The summary of timing of Case Report Forms can be viewed in Table 23**.**

7.2.1 Data Collection For SOC Hormone Therapy

Information relating to SOC hormone therapy is recorded on the SOC Hormone Therapy Log, unless it is a treatment change for disease progression. The SOC Hormone Therapy Log should be updated with any changes in long-term hormone therapy e.g. if anti-androgens are being added to LHRH for dual androgen blockade in the **absence of progression**. If however, anti-androgens are being added as an additional treatment for progressive disease, then this should be recorded on the Additional Treatment Log. Please note SOC hormone therapy only refers to LHRH or anti-androgens.

If a participant allocated to receive transdermal oestradiol switches to receiving SOC Hormone Therapy i.e. LHRH, in the **absence of progression**, then this information should be recorded on the SOC Hormone Therapy Log. However, any changes in hormone therapy initiated to treat disease progression should be recorded on the Additional Treatment Log e.g. switching from transdermal oestradiol to LHRH due to progressive disease.

7.2.2 Data Collection For SOC Docetaxel

7.2.2.A For participants allocated to arms A, K, L

The decision to use docetaxel as part of the standard-of-care (SOC) must be made before randomisation and should be recorded on the Randomisation CRF. The date of the first cycle should be recorded at the time of randomisation; this can be a planned date when randomisation occurs prior to docetaxel commencing but should be within around 12 weeks of starting hormone therapy (see Section 6.1.3). For participants allocated to arms A, K or L all further details should be recorded on the SOC Docetaxel Treatment CRF upon completion of the final cycle. If a participant does not receive the planned docetaxel, this must also be recorded on the SOC Docetaxel Treatment CRF, together with the reason why.

7.2.3 Data collection for SOC Abiraterone

The decision to use SOC abiraterone must be made before randomisation to determine comparison-specific eligibility and to ensure the use of abiraterone is balanced between the control and research group within the “metformin comparison”. The use or planned use of SOC abiraterone must be recorded on the randomisation CRF. A SOC Abiraterone Treatment Log is required for all participants who have started or planned to start abiraterone. The required data collection includes the start date, dose and details of any treatment interruptions including reason (e.g. toxicity, type and grade) and action taken (e.g. dose change or delay). In addition, treatment details for concomitant steroids will also be recorded. If a participant does not receive the planned abiraterone, this must also be recorded on the SOC Abiraterone Treatment CRF, together with the reason why.

7.2.4 Data Collection And Non-Administration Of Standard Radiotherapy

There are CRFs to be completed for **all STAMPEDE participants** regardless of being planned for, or subsequently receiving, primary radiotherapy. Where radiotherapy is not received a reason should be provided on the Radiotherapy Detail CRF whether this is standard-of-care radiotherapy for participants (on any research arm) or research RT to the prostate for Arm H participants.

All radiotherapy and acute side-effects details should be recorded on the Radiotherapy Detail and Radiotherapy Acute Toxicity CRFs upon completion of the RT schedule; any RT late side-effects should be recorded on the Follow-up CRF under the section for RTOG Toxicities.
If RT is not given, this should be stated on the Radiotherapy Detail CRF together with the reason for non-administration of the treatment in those instances where RT was planned and not given (for example, due to early metastatic progression or participant refusal).

7.2.5 Data Collection For Palliative Radiotherapy

Details of any radiotherapy given for progressive disease should be recorded on the Additional Treatment Log and if necessary (e.g. RT for bone pain) as a Skeletal related event (SRE) on the follow-up form.

7.2.6 Data Collection for Research (M1) Radiotherapy

Arm H only: all radiotherapy and acute side-effects details will be recorded on the Radiotherapy Detail and Radiotherapy Acute Toxicity CRFs upon completion of the RT schedule; any RT late side-effects should be recorded on the Follow-up CRF under the section for RTOG Toxicities.

In those cases where RT is not given (for example, due to early metastatic progression or participant refusal), this should be stated on the Radiotherapy Detail CRF together with the reason for non-administration of the treatment.

7.2.7 Data Collection for Additional Treatments Given for Disease Progression

All treatments given for disease progression are recorded on the Additional Treatment Log. Additional treatment should not be given in the absence of disease progression. This log should be updated with all subsequent changes to treatment. Only treatments for progressive disease need to be recorded; details of supportive treatments such as pain killers or bone-strengthening agents e.g. zoledronic acid, given to relieve symptoms, do not need to be provided.

In some scenarios, SOC hormone therapies such as LHRH or anti-androgens may be given as a treatment for progressive disease. For example, LHRH may be re-started on relapse for participants with M0 disease who discontinued hormone therapy and commenced surveillance. In addition, participants allocated to trandermal oestradiol may switch to LHRH on progression. Historically, some participants progressing on LHRH will have commenced anti-androgens (dual androgen blockade) as a treatment for progression. In all cases, if treatment is being started for disease progression, treatment data are collected on the Additional Treatment Log and the details of the progression event recorded on the Progression Log.

Please note that any change in ADT which are solely a change in the participant's long-term hormone therapy, and not for disease progression, should be reported on the SOC Hormone Therapy Log only and not on the Additional Treatment Log.

7.3 FOLLOW-UP PROCEDURE

Every effort should be made to follow-up all participants who have been randomised up until formal closure of a comparison. Participants should, if possible, remain under the care of an oncologist or urologist for the duration of the trial. If care of a participant is returned to the GP, it is the responsibility of the responsible clinician who obtained the participant's consent to participate in the trial to ensure that all relevant data collection forms are completed. Nurse-led follow-up is permitted and should be conducted in line with local practice and procedures.

If the participant moves away from the local area, arrangements should be made for trial follow-up to be undertaken by their new local centre. Details of other participating centres can be obtained from the STAMPEDE Trial Team. Information on participant transfer procedures is detailed in **Section 8.2.** If the responsible clinician moves, appropriate arrangements should be made to arrange for trial follow-up to continue at the centre.

All efforts should be made to preserve the initial participant's consent for long-term survival information to be flagged through national registries, for example NHS Digital (previously the Health and Social Care Information Centre); Office of National Statistics (ONS) in England/Wales; General Register Office in Scotland; Hospital Episode Statistics (HES) or Public Health England.

Please see **Section 8** for more information on early stopping of follow-up.

7.3.1 Follow-up Telephone Consultations

In certain circumstances it may be appropriate to replace hospital visits with telephone consultations providing that it is still possible to collect all the necessary follow-up information. Situations where this may be considered include at the point where participants would normally be discharged from oncology or urology services. In these instances, it is acceptable to alternate appointments with telephone consultations providing the required blood results are available to the research team. All necessary information required to complete the Follow-up CRF is still required. All details on the telephone consultation must be recorded in the participants' notes as per in person assessments.

Other circumstances where it may be appropriate to use telephone consultations is when assessing treatment tolerance and advising regarding dose modifications, providing that all the required safety monitoring has been adhered to. For example, when re-commencing metformin after a treatment break it may be appropriate to confirm tolerance and advise regarding dose escalation over the phone.

7.3.2 Follow-up Using Electronic Healthcare Records

All participants are asked to provide consent to enable the CTU to improve the reliability of long-term follow-up data through linking to other sources of electronic healthcare data. This may include hospital based record systems, NHS digital and national registers, such as the office of national statistics or data held by public health England or other sources which hold relevant information about treatment or outcomes. To ensure study data is updated with accurate data held by others the CTU will collect direct identifiers (patient name and NHS number) and securely store this data for this purpose only, and separately from the trial database. This information will be securely transferred and used to verify the data received by the CTU.

7.4 COMPARISON CLOSURE

For the purpose of complying with UK the clinical regulations (UK Medicines for Human Use Act [Clinical Trials]), each comparison will only be considered 'closed' when active follow-up has ceased. Active follow-up is defined as hospital-based or telephone assessments required to be able to complete the scheduled follow-up assessments. This will be reviewed separately for each comparison after the point of the primary analysis and, if appropriate, later, updated analyses. Longer-term outcome data beyond this time point may be sought through linkage with national registers where possible (and where adequate consent has been obtained) and/or via site research teams.

7.4.1 Comparisons for which Follow-up has Ceased

In Q3-2018, active follow-up will be stopped for all trial participants allocated to the research arms within the "original comparisons"; this is defined as all participants allocated to arms B, C, D, E, and F together with all participants allocated to arm A recruited before 15-Nov-2011.

Active follow-up is defined as hospital-based or telephone assessments required to be able to complete the scheduled follow-up assessments. It should be noted that there may still be some data collection requested from sites to support ongoing sub-studies on closed comparisons. These requests may be for confirmation of health status or data already collected at sites e.g. baseline imaging data and FFPE tumour blocks.

For M1 participants on arm C and contemporaneous arm A, the PSA at baseline will be collected retrospectively where available. Further details regarding this data collection will be disseminated to the respective sites.

Given the above, any longer term analyses of data beyond comparison closure will be performed using observational data collected through national registers and NHS Digital or other datasets, providing such data are accessible.

CRFs, clinical notes and administrative documentation should be kept in a secure location (for example, locked filing cabinets in a room with restricted access). It is permissible to archive this information providing that it can be made accessible and available to the competent or equivalent authorities, the Sponsor, and other delegated authorities with suitable notice as the data may be subject to audit or inspection from any of the above. Information must be held for 25 years after the end of the trial as per **Section 13.1.**

Table 23: Summary of timing of case report forms (CRFs)

CASE REPORT FORMS	TIMING OF ASSESSMENT AND CRF
**Baseline**	
Randomisation Eligibility Checklist	Prior to Randomisation
Randomisation	At Randomisation
Baseline	At Randomisation
Cardiovascular Assessment	At Randomisation
Bone Density Risk Factor	At Randomisation
Blood Form	Prior to starting trial treatment, at progression and end of first line treatment and pre-progression if participant has metastatic disease. Refer to the Sample Collection and Handling Manual for time points.
Saliva Pathology	At randomisation or any point on trial. When saliva sample has been taken and sent to Sponsor's designated laboratory.
**Treatment**	
SOC Docetaxel Treatment	To be completed for all participants randomised to STAMPEDE 20 weeks after randomisation.
SOC Hormone Therapy Log	To be completed every time there is a change in SOC hormone therapy to report (including when Arm L participants switch to SOC HT pre-progression). To be sent in with the corresponding Follow-up CRF.
Abiraterone and Enzalutamide Treatment Log (SOC and research treatment)	To be completed for patients on arms G or J, when treatment is first started and subsequently every time there is a dose change, treatment pause and re-start.<br>Also to be completed for participants who have started or are planned to start SOC Abiraterone within the "metformin comparison".<br>To be sent in with the corresponding Follow-up CRF.
Metformin Treatment	To be completed for patients on arm K, when treatment is first started and subsequently every time there is a dose change, treatment pause and re-start.<br>To be sent in with the corresponding Follow-up CRF.
Transdermal Oestradiol Treatment Log*	To be completed for patients on arm L, when treatment is first started and subsequently when reporting change in dose or type of patch.
RT Detail	To be completed for all participants randomised to STAMPEDE:<br>• Upon completion of SOC RT<br>• If planned RT is no longer planned (at 10 months after randomisation)<br>• Arm H participants when research RT completed<br>• Arm A participants with newly-diagnosed M1 disease at 3 months to confirm RT was not given
RT Acute Toxicity	For all participants who receive primary RT
**Assessments**	
Follow-up	To be completed at every comparison specific follow-up until comparison closure (See **Table 1** and **Table 2** **Error! Reference source not found.**for comparison specific assessment schedules)

CASE REPORT FORMS	TIMING OF ASSESSMENT AND CRF
Toxicity	Required at each follow-up and in the event that treatment is changed due to toxicity.
Transdermal Oestradiol Treatment Hormone Results Log	To be completed whenever there are testosterone and oestradiol test results while arm L participants are on transdermal oestradiol.
End of Research Treatment	To be completed when (each) allocated research treatment is permanently stopped or in the event that allocated research treatment is never started (in each case a reason for stopping/never starting should be provided).
Progression Log	To be completed at the occurrence of each progression event (PSA, local, nodal, distant metastases) and for each method of detection (clinical/symptomatic and objective/radiological).<br>Skeletal-related events confirmed as progression should also be reported here.
Additional Treatment Log	To be completed each time a participant who has progressed starts or completes any additional treatment for progression.
Serious Adverse Event	To be completed following any Serious Adverse Event having confirmed none of the trial specific exemptions are met
Death	At Death
**Administration**	
Participant Transfer Confirmation Form	To be completed when a participant is transferred to a different hospital for the administration of trial treatment and follow-up
Tissue Sample Form	To be completed when sending tumour blocks to Sponsor's designated laboratory.
Co-enrolment	To be completed when a participant is co-enrolled in a post-progression interventional prostate cancer trial. Please see **Section 5.2** for more information.

* For the transdermal oestradiol arm, the 6-week follow-up form can be completed at the same time as the 4-week visit for the hormone tests (see Section 6.2.7.A)

Table 24: Schedule For Completion Of Treatment Forms For All Comparisons By Arm.

Timing From Randomisation			Treatment Log[$]
Years	Months	Weeks	
**6-Weekly**			
0	-	6*	G, J, K, L
-	-	12	G, J, K, L
-	-	18	G, J, K, L
-	6	24	G, J, K, L
**12-Weekly**			
-	9	36	G, J, K, L
1	12	48	G, J, K, L
-	15	60	G, J, K, L
-	18	72	G, J, K, L
-	21	84	G, J, K, L
-	-	96	G, J, K, L
**6-Monthly**			
2	24	104	G, J, K, L
	30	130	G, J, K, L
3	36	156	G, J, K, L
	42	182	G, J, K, L
4	48	208	G, J, K, L
	54	234	G, J, K, L
5	60	260	G, J, K, L
**Annual**			
6	72	-	G, J, K, L
7	84	-	G, J, K, L
Etc.	-	-	G, J, K, L

Key:

G = SOC + abiraterone
J = SOC + enzalutamide + abiraterone
K = SOC + metformin
L = Transdermal oestradiol ± RT ± docetaxel

Notes:

* For the transdermal oestradiol arm, the 6-week follow-up form can be completed at the same time as the 4-week visit for the hormone tests (see Section 6.2.7.A)

$ For participants in Arm L on transdermal oestradiol, the hormone tests results are to be reported on the Transdermal Oestradiol Treatment Hormone Results Log

8 STOPPING OF TREATMENT OR FOLLOW-UP

Participants should be given every encouragement to adhere to their allocated protocol treatment and follow-up schedule, in order to reduce bias. However, a participant has the right to withdraw consent for participation in any aspect of this trial at any time.

8.1 STOPPING RESEARCH INTERVENTIONS

A participant may stop **any STAMPEDE research treatment** for the following reasons:

- Unacceptable toxicity
- Intercurrent illness which prevents further treatment
- Participant refusal
- Any alteration in the participant's condition which justifies the discontinuation of treatment in the clinician's opinion

8.1.1 Stopping Research Treatment: Abiraterone, Enzalutamide + Abiraterone

For **participants randomised to Arm G or J**, research treatment should also be discontinued for the following reasons:

- Disease progression whilst on therapy. As detailed in **Section 7.1.3**, the disease event for stopping treatment may be after the first reportable Failure-Free Survival event. Treatment must be stopped once all three types (biochemical, radiological and clinical) of progression have occurred.
- Intention to commence a new systemic anti-cancer treatment due to evidence of relapse

Trial abiraterone must stop if other systemic treatments are initiated at any time for disease progression control (including chemotherapy, radium-223 etc). Anti-androgens (e.g. bicalutamide) should not be given in combination with abiraterone or enzalutamide due to the risk of toxicity. However, participants may continue on abiraterone or abiraterone and enzalutamide if they receive radiotherapy on a single occasion for a skeletal-related event. Sites must contact the STAMPEDE trial team for further guidance as appropriate.

In all cases reason for permanent stopping of research treatment should be recorded on the End of Research Treatment CRF.

8.1.2 Stopping Research Treatment: Metformin

For **participants randomised to Arm K,** treatment duration is detailed in **Section 6.2.6**.
Please note that in contrast to other treatments tested in STAMPEDE metformin does **not** need to be stopped following progression. Metformin treatment should aim to **continue post-progression** whilst participants continue to receive ADT.

Reasons for early stopping of metformin can be:

- Decline in renal function (metformin must be stopped if GFR <30ml/min/1.73m^2, see **Section 6.2.6.D**)
- Decline in performance status (WHO PS >2)
- Unacceptable toxicity
- Participant refusal
- Intercurrent illness preventing continued metformin treatment
- Investigator decision e.g. administration of IMP within a CTIMP in CRPC setting

If metformin is paused for more than 3 months or >50% of doses are missed please discuss with the trial team as treatment is likely to need to be stopped.

8.1.3 Stopping Research Treatment: Transdermal Oestradiol

For **participants randomised to Arm L**, treatment with transdermal oestradiol may be discontinued for the following main reasons:

- Unacceptable toxicity
- Participant refusal
- Intercurrent illness
- Investigator decision
- Cardiovascular event (see **Section 7.1.4.A**)

In addition, if there is evidence of disease progression, subsequent therapy is at the discretion of the treating clinician with references to any relevant guidelines. For participants who are on the 3 patch maintenance dose and have castrate levels of testosterone, there is currently no evidence that increasing the number of patches further once the participant has progressed would be beneficial and is therefore not recommended.

In the event of stopping research treatment, unless a participant states otherwise, consent is assumed for continued recording of trial data.

8.2 PARTICIPANT TRANSFERS

For participants moving away from the area and planning to transfer care, every effort should be made for the participant to be followed-up at another trial centre. The participant will need to sign a new consent form at the new trial centre. Once this has been done, the new trial centre will take over responsibility for their ongoing participation in the trial.

To document the transfer process the main contact person at both the current and receiving hospitals should complete and sign the Patient Transfer Confirmation form. A fully completed form must be returned to CTU prior to the participant transfer and any outstanding data queries for the participant should be completed prior to transfer.

On receipt of the completed transfer form a member of the STAMPEDE team will confirm the database has been updated and request confirmation of the name of the participant's new clinician. Photocopies of the following documents may then be sent to the new hospital to complete the transfer and originals must be also retained at the original site for monitoring purposes:

- Consent form
- Completed CRFs
- Any documentation relating to the participant's participation in STAMPEDE (participant names must be removed from any documentation).

8.3 EARLY CESSATION OF TRIAL PARTICIPATION

If a participant explicitly withdraws consent to have any further trial data recorded their decision must be respected and CTU must be informed in writing. All communication surrounding the early cessation of trial participation should be noted in the participant's records. Please note data for the participant prior to this decision will still be required.

In the majority of cases, participants continue to give permission for their data and information on their health to continue to be collected via clinical notes and national registries. Any information on the follow-up status, however minimal, would be helpful. Investigators are encouraged to facilitate ongoing collection of follow-up data for example, through considering telephone consultations (see **Section 7.3.1**).

Early cessation of trial participation should not be undertaken lightly and the site must consider the implications for the trial and the participant in reaching such a decision. Without long-term data, the efficacy of trial treatments would be less reliable and could lead to inconclusive results. The early stopping of trial treatment should not lead to the early cessation of trial participation and in such cases follow-up assessments should be continued as per trial protocol.

Participants can change their minds about withdrawal at any time and reaffirm their consent to participate in the trial. Follow-up data should be collected only from the point of when consent was re-instated.

9 STATISTICAL CONSIDERATIONS

9.1 METHOD OF RANDOMISATION

Participants will be randomised centrally using a computerised algorithm developed and maintained by CTU. Randomisation will be performed using the method of minimisation over a number of clinically important stratification factors with an additional random element. To decrease determinability, the factors are not listed here but can be found in the Statistical Analysis Plan.

Participants will be randomised between arms as follows:

- All participants who fulfil both comparison-specific eligibility criteria for metformin and transdermal oestradiol will be allocated between A:K:L.
- All participants ineligible for metformin, but eligible for transdermal oestradiol, will be allocated between A:L
- All participants ineligible for transdermal oestradiol, but eligible for metformin, will be allocated between A:K.

See **Appendix H** for the allocation weighting of each arm by previous protocol version; this also shows allocation weighting for research arms previously closed to recruitment.

9.2 OUTCOME MEASURES

The definitive primary outcome measure for each comparison in the trial is overall survival (all-cause mortality), unless otherwise stated. The design of the trial is such that it is important to have additional intermediate primary outcome measures to assess activity in each research arm as the trial progresses.

For comparisons involving research arms B to J the intermediate primary outcome measure is failure-free survival (FFS); this and other outcome measures are listed in **Table 25.**

Table 25: Trial Outcome Measures by Comparison Stage (Arms B-J)

COMPARISON STAGE	PRIMARY OUTCOME MEASURE	SECONDARY OUTCOME MEASURES
Pilot phase	Safety*	Feasibility
Activity Stages (AS)	Failure-free survival (FFS)†	Overall survival (OS)<br>Toxicity<br>Symptomatic skeletal events (SSE)
Efficacy Stage (ES)	Overall survival	Quality-of-life<br>Cost effectiveness<br>Failure-free survival†<br>Toxicity<br>Symptomatic skeletal events (SSE)

*Based on toxicity
†Including biochemical failure (see Section 7.1.3 and Appendix E)

For the "metformin comparison" the intermediate and definitive primary outcome measure are the same, being overall survival; see **Table 28** for full details of all outcome measures for that comparison.

For the "transdermal oestradiol comparison", overall survival and progression-free survival are the definitive co-primary outcome measures, and the intermediate primary outcome measure is progression-free survival (PFS); see **Table 30**. The rationale for choosing progression-free survival rather than failure-free survival as the outcome measure for this comparison is outlined in **Section 9.7.3**.

The reasons for different emphases in each recruitment stage are explained in **Section 9.3**.

9.3 SAMPLE SIZE: PRINCIPLES

The design is a multi-arm multi-stage, multi-centre, platform, randomised controlled trial. There are a number of stages for each research arm: a Pilot/Feasibility/Safety Phase, Activity Stages and a final Efficacy Stage. Full details of the methodology underlying the trial design are given by Royston et al. (85, 86) The original sample size calculations were performed using the stage2 (version 1.2.0, Mar-2002) and stagen (version 1.1.1, May-2004) programs, both implemented in Stata (Stata Corp, TX) and updated using the later nstage program (version 1.0.3, Jun-2007; version 2.1.0, Jun-2009; version 3.0.1, Sep-2014). (87)

Other than transdermal oestradiol, we have adequately powered each comparison to detect an appropriate improvement in overall survival at the final Efficacy Stage, with high power at each of the planned interim Activity Stages to detect a pre-defined target difference in the intermediate primary outcome. For example, in a cohort with 2 years median FFS and 4 years median overall survival (OS) a target HR of 0.75 for research arm relative to control would translate into an absolute improvement in FFS of 10%, from approximately 50% to 60% at two years, and in OS of 10%, from approximately 50% to 60%, at four years.

The "transdermal oestradiol comparison" is powered only for contributing to a meta-analysis of participants from the STAMPEDE "transdermal oestradiol comparison" and the PATCH trial. It will assess non-inferiority of transdermal oestradiol in terms of overall and progression-free survival which are co-primary outcome measures. For details of the sample size calculations, planned analyses and corresponding operating characteristics, see version 10 of the PATCH protocol.

As each comparison is powered to detect a relative difference in survival, the analyses will be performed when the pre-planned number of events has been reported in the control arm, rather than after a certain number of participants have been recruited to the comparison or a certain amount of time has elapsed. Further details of the sample size calculations and varying assumptions for each research comparison are summarised in the relevant **Sections 9.4-9.8** and detailed in a separate Statistical Design Document which is available on request.

As with all trials, changes in both the standard-of-care and second-line therapies over time are possible which improve outcomes and thus will affect the observed control arm event rates and associated reporting timelines. In particular, from protocol v8.0, standard-of-care RT was mandated for all participants with N0 M0 disease and no RT contraindication (this is likely to improve outcomes for this subgroup) and standard-of-care docetaxel permitted from protocol v14.0. Further agents are starting to be licensed for participants with castrate-refractory disease which may also improve survival rates. Improved FFS rates would delay the intermediate analyses, for comparisons where FFS is the intermediate primary outcome measure; while improved OS would delay the definitive

analyses. Similarly, improved PFS rates could delay both the time of intermediate and definitive analysis for the "transdermal oestradiol comparison". For each comparison event rates are estimated based on data which are publicly available at the time of design. The Statistical Design Document for arms A-K includes models where median survival is varied around such estimated rates.

Figure 3: Schema of progress of STAMPEDE through the trial*

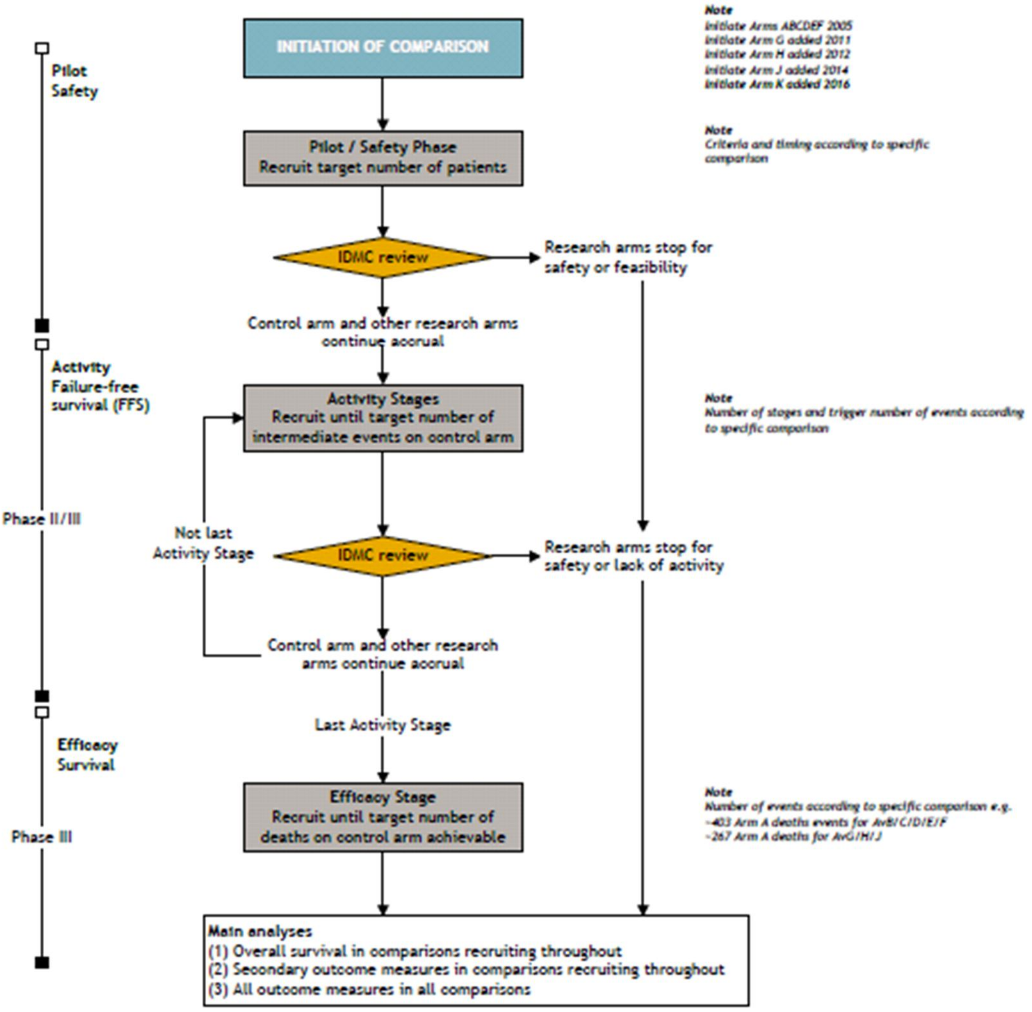

Key
FFS: Failure-free survival
HR: Hazard ratio
IDMC: Independent Data Monitoring Committee
Pts: Patients

Notes
Exact accrual depends on many factors including accrual rate, event rate and arms recruiting in each stage

* Except for the “transdermal oestradiol comparison”

9.4 SAMPLE SIZE ISSUES & TRIAL STAGES: ADDITIONAL RESEARCH ARM H

This is the "M1|RT comparison" and includes participants allocated to research Arm H (SOC+RT) and newly-diagnosed M1 participants with no contraindication to RT allocated to the control Arm A whilst Arm H was open to recruitment. Suitability for allocation to the comparison was assessed before randomisation to ensure comparability with contemporaneous control arm participants.

Table 26: Guidelines for stopping accrual to additional research Arm H

ACTIVITY STAGE	SIG LEVEL	POWER	TARGETED HR	NUMBER OF CONTROL ARM EVENTS	CONSIDER DISCONTINUATION IF (HROBSERVED) IS...
I	0.50	95%	0.75	~75	>1.00
II	0.25	95%	0.75	~142	>0.92
III	0.10	95%	0.75	~221	>0.89

9.4.1 Pilot Phase: Additional Research Arm H

The IDMC reviewed safety data, in the context of data from the control arm, when the first 30 participants allocated to Arm H had been on trial for around six months.

9.4.2 Activity Stages I-III: Additional Research Arm H

The same principles were applied to this new comparison as to previous comparisons and an equal allocation ratio of control arm participants to participants allocated to Arm H was employed, as for Arm G. The number of control arm events required to trigger the intermediate analyses are the same as for the "abiraterone comparison" (see Table 26).

9.4.3 Efficacy Stage IV: Additional Research Arm H

The analysis of Efficacy Stage IV for this comparison will be performed when around 267 deaths have been observed in the relevant control arm participants. This will give 90% power to detect the targeted hazard ratio of 0.75 at one-sided significance level of 0.025.

9.4.4 Sample Size For Additional Research Arm H

Consideration was given to ceasing further randomisations to Arm H if it was not showing sufficient evidence of activity on the intermediate primary outcome measure (FFS), as for the other research arms. This research comparison is relevant to around 60% of participants joining STAMPEDE. At the point of the scientific approval, accrual was averaging around 80 participants per month to the trial; therefore, up to approximately 48 participants a month would be eligible for the comparison. If accrual to the trial was slower at 70 participants per month, then accrual to this comparison could be between 18 and 42 participants per month, depending on which other trial arms are open to recruitment at the time.

We are targeting a 25% relative improvement in overall survival following local radiotherapy to the prostate in this participant group. This is the same size of effect targeted with the other research arms in STAMPEDE. This relative improvement can be further justified in the light of MRC PR07 which demonstrated an improvement of this magnitude for adding radiotherapy to ADT in locally-advanced disease, with a hazard ratio for overall survival of 0.77 (95% CI 0.61 to 0.98). In that trial, fewer than half of the deaths were from prostate cancer, whereas in newly-diagnosed metastatic participants nearly all people will die of their disease. Therefore, it is relevant to note the

relative benefit of radiotherapy in PR07 in terms of prostate cancer-specific survival, where the hazard ratio was 0.46 (95% CI 0.34 to 0.61) after a median follow-up time of 8 years (88).

We anticipated that around 1250 participants were required over 4 years to observe 267 control arm deaths after 5.25 years. This assumed that (i) recruitment was constantly 70 pts/m to the trial overall; (ii) the original research arms stopped accrual within 6 months after activation of the RT arm; (iii) the abiraterone arm stopped accrual around 24 months after activation of the RT arm; and (iv) a further new research arm with an equal allocation ratio was introduced 18 months after activation of the RT arm. In Protocol version 13.0, we reflected on these four points: (i) recruitment to the trial has been faster; (ii) the original research arms completed accrual 2 months after activation of the RT arm; (iii) the abiraterone arm stopped accrual 12 months after activation of the RT arm; and (iv) Arm J was activated 18 months after activation of the RT arm, Arm H.

Of participants joining STAMPEDE during this time, 60% have been eligible for the “M1|RT comparison”. Prior to randomisation, a RT schedule must be nominated: Weekly or Daily. We have observed that around half of participants in the comparison are nominated for RT with the Daily schedule and half for the Weekly schedule, primarily chosen by trial site with participant groups nominated for each schedule observed to be comparable at baseline. There will likely be interest to know the effect of each RT schedule when the main results are reported. This will be explored by “within schedule” comparisons of participants randomised to research vs control (arms H vs A) within each nominated RT schedule.

To ensure adequate power for these “within schedule” analyses, in Protocol version 13.0, the target sample size was increased from 1,250 participants up to around 1,800 participants, resulting in an approximate increase in the split by planned RT schedule from 625 to 900 in each “within schedule” analysis. A FFS analysis “within schedule” will be carried out at the time of the “main analysis”; this is predicted to have ~300 control arm FFS events by schedule (FFS “within schedule” analysis parameters: target HR=0.75, power 90%, 1-sided α=0.015). For either of the RT schedules showing evidence of an effect on FFS, a comparative “within schedule” analysis will be carried out on survival when ~199 control arm deaths are observed in that schedule comparison. This is a closed test with OS only formally compared within schedule if there is an advantage in FFS for that RT schedule at the main analysis. Thus, extending recruitment enables a secondary analysis of the impact of RT on survival by planned “RT schedule” to happen within around 18 months from the first main analysis.

All sample scenarios are documented in the Trial Master File.

All participants joining the trial will be starting long-term ADT for the first time. The focus of this comparison will be on the newly-diagnosed, metastatic participants (with no contraindications to RT), which is the largest subgroup of participants in the trial and the group of participants at highest risk of death from prostate cancer. Participants with non-metastatic disease will be excluded from this particular comparison as there are already randomised data demonstrating the survival benefit from radiotherapy in participants with locally-advanced disease. Radiotherapy is now mandatory in node negative participants; it is also recommended in the node-positive, non-metastatic (N+ M0) group. Relapsing participants are also excluded from this comparison.

For the control arm of the whole trial, we constructed sample size scenarios based on median failure-free survival being 18, 24 or 30 months; the event rate would depend on the participant mix. We now know that around 60% of participants have M1 disease at trial entry and we have reported that FFS at 24 months is 51% across the whole of the control arm participant sample.(89)

For the updated sample size calculation for the “M1|RT comparison”, we based our estimates on the subgroup of participants with newly-diagnosed M1 disease in the control arm. Therefore, we

estimate median FFS for control arm participants in this comparison to be 1 year and estimate that median overall survival will be around 3.5 years.

9.5 SAMPLE SIZE ISSUES & TRIAL STAGES: ADDITIONAL RESEARCH ARM J

This is the "enzalutamide + abiraterone comparison" and includes participants allocated to research Arm J (SOC + enzalutamide + abiraterone) and participants contemporaneously allocated to the control Arm A.

9.5.1 Pilot Phase: Additional Research Arm J

The IDMC first reviewed safety data for this combination when the first 50 participants allocated to Arm J had been on trial around 6 weeks (i.e. to the first follow-up visit).

Furthermore, an additional review of safety was performed when these 50 Arm J participants had been on trial for around 6 months. Safety is routinely reviewed at regular intervals and additional safety reviews will be performed if the IDMC raises any concerns.

Direct comparison will be available with contemporaneously randomised participants on Arm A (SOC alone). Contextual data will be provided from Arm G (SOC plus abiraterone). Indicative safety data may also be available on the combination from other studies in CRPC.

9.5.2 Activity Stages I-II: Additional Research Arm J

The principles of intermediate analyses were applied to this new comparison as to previous comparisons, but some of the details were different, and an equal allocation ratio of control arm participants to participants allocated to Arm J was employed; as for Arms G and H. Owing to the expected accrual rate to the trial (>100 pts/m), the expected slower event rate in all participants given improvements to SOC and specifically in participants randomised to this comparison. Given the simultaneous recruitment of M1 (but not M0) participants to the "M1|RT comparison", only two activity stages were planned before accrual completed. These are set out in **Table 27**.

Table 27: Guidelines for stopping accrual to the additional research Arm J

ACTIVITY STAGE	SIG LEVEL	POWER	TARGETED HR	NUMBER OF CONTROL ARM EVENTS	CONSIDER DISCONTINUATION IF (HRJOBSERVED) IS...
I	0.40	95%	0.70	~66	>0.957
II	0.12	95%	0.70	~139	>0.869

9.5.3 Efficacy Stage III: Additional Research Arm J

The analysis of the final Efficacy Stage for this comparison will be performed when around 267 deaths have been observed in the control arm. This would give 90% power to detect the targeted hazard ratio of 0.75 at a one-sided significance level of 0.025.

9.5.4 Sample Size For Additional Research Arm J

Consideration was given to ceasing further randomisations to Arm J if it was not showing sufficient evidence of activity on the intermediate primary outcome measure (FFS), just as for the other research arms.

The participant mix for this comparison is likely to represent a more favourable prognosis on average than in the original comparisons, due to concurrent recruitment of M1 but not M0 participants, to Arm H.

We anticipate that around 1,800 participants are required within 3.5 years to observe ~267 control arm deaths within 6 years. This time will be dependent on the observed overall survival. The default scenario assumes that (i) recruitment is constantly 70pts/m to the trial overall, (ii) Arm H (M1|RT) accrues throughout and (iii) a further new research arm with an equal allocation ratio is introduced 18 months after activation of Arm J. The stopping date for Arm G is no longer an assumption.

Variations on these factors are documented in a Statistical Design Document. If accrual rates to the trial are at 150pts/m (as observed during summer 2013), accrual of around 1,800 participants to the comparison could be achieved within 2 years. These sample scenarios will also be documented in the Trial Master File.

Updating the standard-of-care to include docetaxel has minimal impact on the projected time to maturity of the “enzalutamide + abiraterone comparison”.

9.5.5 Further Sample Size Issues For Additional Research Arm J

Careful consideration will be given to the implications of any emerging data from the "abiraterone comparison". This had no effect on recruitment to the “enzalutamide + abiraterone comparison” because the recruitment target was reached before any data were available from the "abiraterone comparison".

Indirect comparisons to understand the contribution from each agent may be possible if this research arm is demonstrably superior to the standard-of-care. These plans will be developed and documented elsewhere, but a higher number of participants will help with the power to the indirect comparison.

9.6 SAMPLE SIZE ISSUES & TRIAL STAGES: ADDITIONAL RESEARCH ARM K

This is the “metformin comparison” and includes participants allocated to research Arm K (SOC + metformin) and the equivalent non-diabetic participants with no contraindication to metformin contemporaneously allocated to the control Arm A whilst Arm K is open to recruitment. Suitability for allocation to the comparison is assessed before randomisation to ensure comparability with contemporaneous control arm participants

9.6.1 Implementation: Additional Research Arm K

The implementation of the MAMS principles are different in this comparison for the following reasons:

- Although all non-diabetic participants will be eligible for allocation to the “metformin comparison”, the timing of the analyses will be driven only by the M1 participants. (See Section 9.6.4 for discussion of the implications for power overall and in M0/M1 subgroup analyses.)

- Failure-free survival will not be used as the intermediate primary outcome measure; overall survival will be used as both the intermediate and definitive primary outcome measure. This is because we are not convinced that any comment on metformin’s usefulness should be determined from an ability to act on a PSA-driven outcome measure. Furthermore, treatment with metformin is intended to continue throughout long-term hormone therapy which may include going well beyond an FFS event, particularly in M1 participants.

- The target HR is 0.80 for overall survival (a 20% relative improvement). This is a smaller relative improvement in survival than targeted for previous comparisons because of metformin's known low toxicity profile, the low cost of the drug and the potential positive effects on metabolic parameters and morbidity; a smaller impact on overall survival may still have clinical benefit.

9.6.2 Outcome Measures: Additional Research Arm K

Table 28 lists the outcome measures for this comparison and can be compared with the outcome measures for the other comparisons in Table 25.

Table 28: Trial outcome measures by stage for the "metformin comparison"

COMPARISON STAGE	PRIMARY OUTCOME MEASURES	SECONDARY OUTCOME MEASURES
Pilot phase	Safety*	Feasibility<br>Metabolic effects§<br>Cardiovascular event: major adverse cardiac events‡
Activity Stage (AS) I	Overall survival	Failure-free survival† (FFS)<br>Symptomatic skeletal events (SSE)<br>Toxicity<br>Metabolic effects §<br>Cardiovascular event: major adverse cardiac events‡
Efficacy Stage (ES) II	Overall survival	Metastases-free survival (M0 participants)<br>Progression free survival (M1 participants)<br>Toxicity<br>Symptomatic skeletal events (SSE)<br>Failure-free survival† (FFS)<br>Metabolic effects §<br>Quality-of-life<br>Cost effectiveness<br>Correlative outcomes₳<br>Cardiovascular event: major adverse cardiac events‡

*Based on toxicity
§Including changes in: BMI; Haemoglobin A1c (HbA1c); waist circumference and a new diagnosis of diabetes mellitus
‡MACE; nonfatal MI, nonfatal stroke, & death from CVS causes
†Including biochemical failure (see Section 6.1.2 and Appendix J)
₳Plasma lipid and fasting triglyceride levels, fasting plasma glucose
Sarcopenia and/or radiological progression free survival (rPFS)
Plasma insulin
AMP Kinase

Note: All arms are unblinded so primary outcome measures for this comparison are objectively measured with caution to be taken around interpretation of more subjective secondary outcome measures such as symptomatic skeletal events

9.6.3 Pilot Phase: Additional Research Arm K

The IDMC will review safety data for this comparison when the first 50 participants allocated to Arm K have been on trial around 12 months. Furthermore, analyses will be conducted on metabolic parameters (see Table 28). If there is harm observed in metabolic effects, or any serious concerns regarding the toxicity profile, recruitment would be stopped; there are no formal criteria to guide this.

Safety is routinely reviewed at regular intervals and additional safety reviews will be performed if the IDMC raises any concerns.

9.6.4 Activity Stage I: Additional Research Arm K

The principles of intermediate analyses will be applied to this new comparison as to previous comparisons, but some of the details will be different, and an equal allocation ratio of control arm participants to participants allocated to Arm K is employed; as for Arms G, H and J. Owing to the expected accrual rate to the trial overall (>100 pts/m) and the interim primary outcome being overall survival, only one intermediate activity stage is planned before accrual is completed; this is set out in Table 29..

Although analyses are triggered by events in M1 participants, they will include all participants in the "metformin comparison"; this will have high power. A separate subgroup analysis in M1 participants (conventionally-powered) and M0 participants (limited power) will then look at consistency of effect; few deaths in M0 participants are expected at this time. The IDMC recommendation will be based on the totality of the available data, including safety, metabolic and compliance data.

Table 29: Guidelines for stopping accrual to the additional research Arm K

ACTIVITY STAGE	SIG LEVEL	POWER	TARGETED HR	NUMBER OF CONTROL ARM EVENTS	CONSIDER DISCONTINUATION IF HR_K (OBSERVED) IS...
I	0.40	92%	0.80	~121 M1 deaths	>0.965

9.6.5 Efficacy Stage II: Additional Research Arm K

The analysis of the final Efficacy Stage for this comparison will be performed when around 473 deaths have been observed for M1 participants randomised contemporaneously to the control arm. This would give 92% power to detect the targeted hazard ratio of 0.80 at a one-sided significance level of 0.025 at the final Efficacy Stage, and 86% pairwise power overall.

As with the intermediate activity, this analysis will include all participants in the comparison, with a separate subgroup analysis in M1 and M0 participants looking at consistency of effect. At this time point we predict approximately 100 control arm M0 deaths will be observed. Further subgroup analyses, defined by the stratification factors, are planned to check for consistency of effect at intermediate and final analyses. Due to this comparison being powered for overall survival in M1 patients, the relatively high OS for M0 patients means that anaysis of OS in this subgroup will not have high power. As such, an additional outcome measure of metastases-free survival will be analysed as part of the subgroup analysis.

9.6.6 Sample Size For Additional Research Arm K

Consideration will be given to ceasing further randomisations to Arm K if it is not showing sufficient evidence of improvement on overall survival at the intermediate analysis.

We anticipate that around 2,800 participants, including around 1,700 M1 participants, are required over 3 years to observe ~473 control arm M1 deaths over around 7 years. (This is a revision from the initial target – see **Section 9.6.7**). This number and time will be dependent on the observed overall survival. The default scenario assumes (i) recruitment is constantly 100pts/m to the trial overall, (ii) co-recruitment throughout of the equivalent of one other research arm, and (iii) the majority of metastatic participants will also have docetaxel but non-metastatic participants will not. Variations on these factors are documented in a Statistical Design Document. Sample scenarios will also be documented in the Trial Master File.

Updating the standard-of-care to permit first-line use of docetaxel was assumed within the sample size scenarios and is reflected in the projected time to maturity of the "metformin comparison".

9.6.7 Further Sample Size Issues For Additional Research Arm K

Careful consideration will be given to the emerging data from the "abiraterone comparison" when this reports in 2017.

Analyses for the "metformin comparison" will be timed from randomisation. The point of randomisation compared to the start of hormone therapy may differ, depending on the planned use of docetaxel. This practical information will be reviewed by the TMG and IDMC.

For the development of Protocol v19, the sample size calculations for the "metformin comparison" were discussed by the TMG and revised to the estimates as presented in Section 9.6.4 – Section 9.6.6.

The original sample size estimates for this comparison were based on a lower target for power than the previously-added comparisons, with 90% power for the interim analysis, 85% power for the final analysis and 80% pairwise power overall. The observed accrual to the "metformin comparison" is higher than forecast. Therefore the TMG took the opportunity to revisit the sample size target for the "metformin comparison".

The revised sample size estimates aim for a higher target power of 92% at both interim and final analysis with 86% pairwise power overall, increasing the analysis power for this comparison in line with that of previous STAMPEDE comparisons. These revisions have resulted in the overall sample size for the comparison increasing from 1800 patients in Protocol v18 to 2800 patients in Protocol v19. This will be achievable within the forecast timelines for recruitment i.e. by the end of 2019, and has the benefit of bringing forward the reporting timelines by approximately one year.

9.7 SAMPLE SIZE ISSUES & TRIAL STAGES: ADDITIONAL RESEARCH ARM L

This is the "transdermal oestradiol comparison" and includes participants allocated to research Arm L (transdermal oestradiol ± RT ± docetaxel) and the equivalent, eligible participants contemporaneously allocated to the control Arm A (SOC).

The phase III evaluation of the clinical efficacy of transdermal oestradiol will ultimately be based on the relevant data from this comparison within STAMPEDE and the PATCH trial, combined using an individual participant data meta-analysis. The overall evaluation is based on a non-inferiority design.

9.7.1 Implementation And Outcome Measures: Additional Research Arm L

The transdermal oestradiol evaluation is based on the following approach.

9.7.1.A Earlier Stages In The PATCH Trial

- The early stages of the PATCH trial already demonstrated the safety and early activity of transdermal oestradiol in comparison to LHRH therapy (see **Appendix I**)(54). The pilot phase (completed in 2010, n=254) showed the rates of cardiovascular events in the transdermal oestradiol and LHRH arms were similar, and the castration rates were equivalent. These results were confirmed by longer-term data including nearly 900 patients enrolled up to Oct-2015.

- A pre-planned, confidential interim analysis undertaken in Jun-2013, based on progression-free survival, at the end of the Phase II component of the PATCH trial, led the PATCH IDMC to recommend further recruitment for an extension to Phase III. That analysis included 638 patients with 206 PFS events, and reviewed data against a pre-specified non-inferiority margin hazard ratio of 1.25 with a 1-sided alpha 0.25.

9.7.1.B STAMPEDE And PATCH Meta-analysis

- To assess the clinical efficacy of transdermal oestradiol, the relevant data from the STAMPEDE "transdermal oestradiol comparison" will be combined with that data from all patients recruited into PATCH; the data from STAMPEDE will not be analysed alone.

- As the eligibility criteria with respect to the timing of start of ADT differs between the STAMPEDE "transdermal oestradiol comparison" and the PATCH trial (see **Section 4.3.1**), the "transdermal oestradiol comparison" will undergo an initial Pilot Phase to assess castration rates and safety among those participants on Arm L. This will also include a safety review of participants receiving transdermal oestradiol in combination with docetaxel. The data will be reviewed by the PATCH IDMC when there are 30 participants in Arm L who have been followed up for at least 18 weeks. A feasibility review will also be performed at the same time.

- The pre-planned Activity Stage II, on intermediate primary outcome measure progression-free survival, will take place based on combined data from the STAMPEDE "transdermal oestradiol comparison" participants and PATCH patients.

- The same approach will be used at the final Efficacy Stage, with progression-free and overall survival as definitive co-primary outcome measures (see PATCH Protocol v10.0 for further details). The rationale for choosing progression-free survival as both the intermediate primary outcome measure and as part of the definitive co-primary outcome measure for the "transdermal oestradiol comparison" is outlined in **Section 9.7.3**

Table 30 summarises the outcome measures for each stage of this research comparison. The target sample size for the meta-analysis of the "transdermal oestradiol comparison" is approximately 2,000 participants, with around 500 to be recruited through the STAMPEDE "transdermal oestradiol comparison". By Feb-2017, around 1,200 patients had been recruited directly to the PATCH trial.

Table 30: Trial outcome measures by stage for the "transdermal oestradiol comparison"

COMPARISON STAGE	DATA SOURCE(S)	PRIMARY OUTCOME MEASURES	SECONDARY OUTCOME MEASURES
Pilot phase (completed 2010)	PATCH trial	Cardiovascular morbidity and mortality	Castration rates<br>Other toxicities<br>Metabolic effects
Activity Stage I (completed 2013)	PATCH trial	Progression-Free Survival*	Cardiovascular and other toxicities<br>Castration rates<br>Metabolic effects
Activity Stage II+$	PATCH and STAMPEDE trials	Progression-Free Survival*	Cardiovascular & other toxicities
Efficacy Stage III$	PATCH and STAMPEDE trials	Progression-Free Survival*<br>Overall survival	Cardiovascular & other toxicities<br>Prostate cancer specific survival<br>Quality-of-life

* Defined as the earliest among biochemical failure, clinical progression (local progression, lymph node progression, distant metastases), or death from any cause (see Section 9.7.3).

+ In addition, there is Pilot Phase to assess castration rates and safety among Arm L participants within STAMPEDE, since the eligibility criteria with respect to timing of start of ADT differs between the transdermal oestradiol comparison within STAMPEDE and the PATCH trial (see Section 4.3.1).

$ The timing of these analyses is determined by when a pre-specified number of events for the primary outcome measure have been observed in the control arms for the PATCH and STAMPEDE trials combined. Please see the PATCH Protocol v10.0 for further details.

9.7.2 Additional Use of Outcome Data from the "transdermal oestradiol comparison"

Participants allocated to the "transdermal oestradiol comparison" may provide additional consent to participate in translational sub-studies, see Section 4.7 for details. Subsequent correlative analysis using outcome data from these participants will be undertaken by the STAMPEDE team and collaborators, overseen by the STAMPEDE BRG and other STAMPEDE oversight committees.

9.7.3 Definition of PFS and Use As Co-primary Outcome Measure: Additional Research Arm L

Note that the definition of progression-free survival (PFS) used within the "transdermal oestradiol comparison" analyses differs slightly to that of failure-free survival used for other research comparisons within STAMPEDE. This is because it includes death from any cause as an event- i.e. both PCa deaths and non-PCa deaths (see Appendix D for further details of the definition of progression). Progression-free survival is hence defined as time from randomisation to the first of: biochemical failure, clinical progression or death from any cause.

The use of PFS rather than FFS for the "transdermal oestradiol comparison" has no practical impact on STAMPEDE. The rationale for choosing PFS as part of the co-primary outcome measure for the

"transdermal oestradiol comparison" is to capture any potential effects on survival due to the different toxicity profiles between transdermal oestradiol and LHRH.

Although PFS and survival are co-primary endpoints, their respective primary analyses will be triggered at different timepoints particularly because PFS is likely to contain a relatively low proportion of deaths as the contributing first PFS event.

9.8 FURTHER NOTES ON TRIAL DESIGN

9.8.1 Overall Sample Size

Given the adaptive nature of the study, there is no formal overall sample size target, but the numbers of participants required for each comparison are detailed in **Sections 9.4-9.8**. To date, more than 10,000 participants have been recruited overall and at least 11,000 participants will join the trial.

9.8.2 Factorial Design

We note here that we did not employ a factorial design in the original design of this trial because we anticipated the possibility of synergy between SOC, zoledronic acid and docetaxel and between SOC, zoledronic acid and celecoxib.

It would not be possible to assess any such interactions reliably in a factorial trial (see the Statistical Design Document for further details).

9.9 INTERIM MONITORING AND ANALYSES

The accumulating data will be reviewed at regular intervals (approximately annually) by an Independent Data Monitoring Committee (IDMC), including pre-specified formal intermediate analyses of activity data (see also **Section 16**). These analyses will be performed by the trial team at CTU. Only participants randomised contemporaneously, and eligible for that comparison, will be included in the comparison of each research arm against control e.g. participants allocated to the control arm prior to Protocol version 12.0 will not contribute to the "enzalutamide + abiraterone comparison" (Arm A vs Arm J). For the "transdermal oestradiol comparison", the relevant STAMPEDE data will only be analysed as a meta-analysis in combination with the PATCH trial. Therefore, interim data from this comparison will be reviewed by the PATCH IDMC.

The IDMC will be asked to give advice on whether the accumulating data from the trial justifies continuing recruitment of further participants or further follow-up; guidelines for discontinuation of accrual for the relevant Activity Stages, together with results from any other relevant trials will aid them in this. A decision to discontinue recruitment, either in all participants or in selected subgroups, will be made only if the result is likely to convince a broad range of clinicians including those entering participants into the trial and the general clinical community. The intermediate stopping guidelines apply to the intermediate primary outcome measure.

To stop accrual early for benefit in any comparison would require convincing data in terms of the definitive primary outcome measure, overall survival. For example, this could be one-sided $p<0.0005$ as proposed by Haybittle-Peto.(90, 91) The use of such a guideline for stopping for benefit has a minimal impact on the operating characteristics.

If a decision is made to continue without change, the IDMC will advise on the frequency of future reviews of the data on the basis of accrual and event rates. The IDMC will make recommendations to the Trial Steering Committee (TSC, see **Section 16**) as to whether the trial should continue in its present form. While the trial is ongoing the accumulating data will generally remain confidential, unless the TSC and IDMC agree that the data should be made public.

9.10 OUTLINE ANALYSIS PLAN

Analyses will be performed on an intention-to-treat basis.

For comparisons involving arms A-K, the standard unadjusted log-rank approach will be applied to analyses of intermediate and definitive primary outcome measures. The impact of potential confounders including the stratification factors used at randomisation will be considered in a Cox proportional hazard model.

Flexible parametric models will be used to calculate the absolute differences between the arms to show treatment differences over time and to estimate restricted mean "survival" times (RMST). The estimated difference in RMST will be used preferentially to compare treatment arms if the proportional hazards assumptions required for hazard ratios cannot be supported. The χ^2 test or Mann-Whitney test will be implemented for categorical data comparisons, including toxicity, as appropriate. Where relevant the primary outcome measure(s) (see **Section 9.2**) will be considered for all arms of the trial at each phase, but the main emphasis will be placed on the comparison of the research arms that have continued to recruit throughout the trial.

In the "transdermal oestradiol comparison," a meta-analysis approach will be used to combine data from the STAMPEDE and PATCH trials. The analysis will also take into account the change in randomisation ratio partway through the PATCH trial (from 2:1 for transdermal oestradiol versus LHRH before Feb-2011, to 1:1 thereafter). In addition, as the comparison uses a non-inferiority design, sensitivity analyses will be conducted based on a number of pre-defined descriptions for the per-protocol population.

9.10.1 Pilot / Safety Phases

Feasibility of the trial originally, and now of individual research comparisons, was and still is considered in terms of acceptability of the trial randomisation, reported toxicities and adherence to trial medication. Centres participating in the Pilot Phase for the original research arms were required to keep an anonymised log of all participants assessed for trial eligibility (see protocol v2.0) so that the number of participants who did not participate in the study and the number of eligible participants who chose to not participate in the study could be summarised (reasons for non-participation were collected where the participant was willing). The anonymised logs are no longer needed for new research arms (since protocol v8.0).

For each research comparison we shall describe the incidence of expected and unexpected severe toxicities and adverse events/reactions (see **Section 11.1.1**) amongst the participants who are randomised to the comparison to decide whether to continue beyond this Pilot/safety Phase.

9.10.2 Activity And Efficacy Stages

The approach to analysis of these stages is summarised within the sample size calculations (see earlier subsections of **Section 83**). Each research arm will be compared in a pairwise fashion against the contemporaneously recruited control arm.

Full details are available in the Statistical Analysis Plan. See **Figure 3** for an overview of the schema of progress.

10 MONITORING AND QUALITY ASSURANCE

10.1 MONITORING AT CTU

Data provided to the CTU will be checked for missing or unusual values (range checks) and consistency over time. If missing or questionable data are identified, staff at the CTU will request that the data be clarified. The exact procedures for data clarification and the amendment of CRFs will be described in the trial Data Management Plan and instructions will be sent to all STAMPEDE institutions as soon as they have been approved to participate in the trial. The CTU will also send reminders for any overdue data

10.1.1 Central monitoring of consent

Anonymised copies of the participant's initial consent form (including the additional research consent) should be sent to the STAMPEDE team at the CTU, as soon as randomisation has been completed. Once the consent has been received by the CTU and reviewed, the participants "treatment and follow up schedule" can be released to sites along with the Randomisation Eligibility Checklist.

Any subsequent re-consent forms should be sent as soon as possible to enable central monitoring and recording of consent. The dates and signatures should be visible on the copies sent to the CTU; however the name of the participant must be omitted. Any queries resulting after central monitoring will be redirected to sites for clarification. The original non-anonymised consent forms should be kept at site in the Investigator Site File.

10.2 DIRECT ACCESS TO DATA

Collaborating institutions should be aware that direct access to participant data by CTU staff may be required for trial-related monitoring or audit. Participant consent for this will be obtained as part of the general trial consent process.

10.3 VISITS TO INVESTIGATOR SITES

A selection of institutions will be visited at least once during the course of the STAMPEDE trial. The CTU will give the responsible investigator adequate notice of the monitoring visit to allow adequate time, space and staff for these visits. The standard operating procedures (SOPs) for monitoring are available from the CTU.

After the monitoring visit the monitor will complete a site visit report. This report may be circulated to the CTU team for comment. Once the TMT have reviewed the report and agreed on any recommendations the monitor will finalise the report and send a copy to the Principal Investigator (PI) at the site. A copy will be kept in the CTU STAMPEDE Trial Master File.

10.4 CONFIDENTIALITY

All information collected during the course of the research will be kept strictly confidential. In addition, all procedures for handling, processing, storage and destruction of data are compliant with the Data Protection Act 1998. No individual participants will be identified when results from the trial are published.

Participants are asked to give their permission for information about their health status to be obtained from the Office of National Statistics (ONS), via NHS Digital (formerly HSCIC), Public Health England, National Cancer Research Advisory Service, or any similar or national equivalent. This will facilitate data collection and verification and reduce the burden on sites. In addition, participants will be asked for permission to inform their GP of their involvement in the STAMPEDE trial.

11 SAFETY REPORTING

The principles of GCP require that both investigators and sponsors follow specific procedures when reporting adverse events/reactions in clinical trials. These procedures are described in this section of the protocol and in **Section 7.1.5**. Further information on the expected toxicities for the investigational medicinal products (IMPs) being tested in arms on active follow-up (LHRH analogues, abiraterone, enzalutamide, metformin and transdermal oestradiol) can be found in the reference safety information accessible via the STAMPEDE website, all links are found in **Appendix C**.

11.1 SAFETY REPORTING DEFINITIONS

The definitions of the EU Directive 2001/20/EC Article 2 based on the principles of GCP apply to this trial protocol. These definitions are given in **Table 31**.

Table 31: Event Terms and Definitions

TERM	DEFINITION
Adverse Event (**AE**)	Any untoward medical occurrence in a clinical trial participant to whom a medicinal product has been administered. These include occurrences which are not necessarily caused by the product.
Adverse Reaction (**AR**)	Any untoward and unintended reaction to an investigational medicinal product related to any dose administered.
Unexpected Adverse Reaction (**UAR**)	An adverse reaction, the nature or severity of which is not consistent with the information about the medicinal product in question set out in reference safety information (summary of product characteristics or Investigator brochure) for that product.
Serious Adverse Event (**SAE**) or Serious Adverse Reaction (**SAR**) or Suspected Unexpected Serious Adverse Reaction (**SUSAR**)	Respectively any adverse event, adverse reaction or unexpected adverse reaction that fulfils the definition of **serious:**<br>• results in death<br>• is life-threatening*<br>• requires hospitalisation or prolongation of existing hospitalisation**<br>• results in persistent or significant disability or incapacity<br>• consists of a congenital anomaly or birth defect<br>• Other important medical condition***

Clarifications and Exceptions

*The term 'life-threatening' in the definition of 'serious' refers to an event in which the participant was at risk of death at the time of the event; it does not refer to an event which hypothetically might have caused death if it were more severe.

**Hospitalisation is defined as an inpatient admission, regardless of length of stay, even if the hospitalisation is a precautionary measure for continued observation. A&E attendances are not defined as a hospitalisation unless participants are admitted. Hospitalisations for a pre-existing condition, not thought to have been exacerbated by STAMPEDE protocol treatment (including elective procedures that have not worsened) do not constitute an SAE.

*** Medical judgement should be exercised in deciding whether an AE/AR is serious in other situations. Important AE/ARs that are not immediately life-threatening or do not result in death or hospitalisation but may jeopardise the participant or may require intervention to prevent one of the other outcomes listed in the definition above, should also be considered serious.

11.1.1 Adverse event definitions

Adverse events (AE) include:

- An exacerbation of a pre-existing illness
- An increase in frequency or intensity of a pre-existing episodic event or condition
- A condition (event through it may have been present prior to the start of the trial) detected after trial drug administration
- Continuous persistent disease or a symptom present at baseline that worsens following administration of the study treatment

Serious Adverse Events (SAE) are AEs that fulfil the definition of serious as detailed in **Table 31.** SAEs are reported using the SAE CRF. If the event is assessed as possibly, probably or definitely related to treatment, it is categorised as a Serious Adverse Reaction (SARs). If the reaction is unexpected based on the product reference safety information, it is categorised as a Suspected Unexpected Serious Adverse Reaction (SUSAR), see **Table 32.**

Notable Adverse Events (NAE) these include any new primary cancers which should be reported as a NAE on a SAE CRF for expedited clinical review. Except non-melanoma skin cancer (e.g. basal cell carcinomas and squamous cell carcinomas) which should be recorded as an AE on the Toxicity (AE) CRF. Other NAEs include pregnancy occurring in a partner of a STAMPEDE participant. This must also be reported on an SAE CRF. Pregnancies must be followed up until outcome, whether this is a live birth, stillbirth, or planned or spontaneous abortion.

11.1.2 Defining treatment for the purposes of safety reporting

STAMPEDE is an adaptive platform protocol in which research treatments are given in addition to standard-of-care (SOC) therapies, or as alternatives in the case of transdermal oestradiol.

Protocol treatments are grouped as:

- **Protocol SOC treatments** are standard forms of treatment permitted as part of the STAMPEDE protocol:
  - Licenced ADT (e.g. LHRH analogues) given in the setting of castrate-sensitive prostate cancer
  - Docetaxel given in castrate-sensitive prostate cancer
  - Abiraterone given in castrate-sensitive prostate cancer

  Please note, if a participant allocated to transdermal oestradiol switches to standard ADT in the absence of progression, this would still be considered as being on protocol treatment.

- **Protocol research treatments** are the IMPs under investigation in STAMPEDE i.e. the additional or alternative treatments participants allocated to research arms on active follow-up (G-L) receive as part of the STAMPEDE protocol:
  - Arm G: abiraterone
  - Arm J: abiraterone & enzalutamide
  - Arm K: metformin
  - Arm L: transdermal oestradiol

  Note, the research treatment in arm H (prostate RT) is not an IMP, but safety reporting requirements to the CTU are the same.

Non-protocol treatments are all prostate cancer treatments commenced post disease progression in the management of CRPC. This includes ADT given in the setting of CRPC.

11.2 SAFETY PROCESSES: TRIAL-SPECIFIC SAE REPORTING EXEMPTIONS

The following events which may fulfil the definition of "serious" are exempt from regulatory reporting and therefore expedited reporting is not required. They may still require reporting as an AE on the Toxicity (AE) CRF, or on an alternative CRF e.g. progression log.

- **Serious adverse events** unrelated to protocol treatment i.e. unrelated SAEs (refer to Section **11.1.2**) occurring more than 30 days after stopping protocol treatment.

- **Serious adverse events** occurring after disease progression that are **unrelated** (i.e. not SARs or SUSARs) to protocol treatment are exempt, providing protocol treatment stopped at least 30 days ago.
  N.B non-protocol treatment includes ADT in CRPC setting, therefore the 30 day rule does not apply for patients continuing on ADT alone. (Refer to Section **11.1.2**)

- **Non-fatal progression events:** events that fulfil the definition of serious e.g. result in hospital admission, but are due to disease progression are exempt from reporting as an SAE, instead details should be provided on the Progression Log.

- **Death as a result of disease progression or disease-related deaths:** Do not complete an SAE CRF, instead details should be reported on the Death Form.

- **Elective hospitalisation** and surgery for treatment of locally-advanced or metastatic prostate cancer or its complications. These should be recorded as a non-trial inpatient admission on the follow-up form under Non-Trial visits.

- **Elective hospitalisation** to simplify treatment or procedures. If related to prostate cancer, record as non-trial inpatient admission on the follow-up form. If unrelated e.g. pre-existing conditions that have not been exacerbated by protocol treatment, do not report.

11.3 SITE INVESTIGATOR RESPONSIBILITIES

11.3.1 Notification period

All **Adverse Events** (AEs) are reportable from the time of randomisation until 30 days after discontinuation of protocol treatment (refer to Section **11.1.2**). All AEs should be recorded in the participant's medical notes and on the Toxicity(AE) CRF linked to the Follow-up CRF. The Toxicity (AE) CRF should be sent to the CTU within one month of the corresponding Follow-up CRF being due.

All **Serious Adverse Events** (SAEs) are reportable from the time of randomisation until 30 days after discontinuation of protocol treatment (refer to Section **11.1.2**), with the exception of reactions (SARs and SUSARs) which continue to be reportable until comparison closure. In the case of LHRH analogues this is assumed to be 30 days after the depot expiration date e.g. up to 8 weeks after administration of a 4-week depot or 16 weeks after administration of a 12-week depot. SAEs must be reported within 24 hours of the investigator being made aware of the event using the SAE CRF.

All **Notable Adverse Events** are reportable from randomisation until comparison closure, using the SAE CRF.

11.3.2 Investigator Assessment

11.3.2.A Seriousness

When an AE occurs the investigator or delegate must assess whether the event is serious. Refer to **Table 31** and **Section 11.2** a list of exemptions.

11.3.2.B Grading severity of adverse event

The severity (i.e. intensity) of all AEs must be graded using Common Terminology Criteria for Adverse Events (CTCAE) v4.03. The complete CTCAE v4.03 can be found at:
http://www.stampedetrial.org/centres/tools-training/training-materials-resouces/
Any questions concerning this process should be directed to the CTU team in the first instance.

11.3.2.C Causality

The Investigator must assess the causality of all serious events or reactions in relations to protocol treatment using the definitions in **Table 32**.

11.3.2.D Expectedness

If there is at least a possible involvement of the protocol treatment, the investigator should make an initial assessment of the expectedness of the event, however the Sponsor has the final responsibility for determination of expectedness. This determines whether a reaction is a SAR or SUSAR, see **Table 32**. Expectedness is determined using the current reference safety information (RSI) (i.e. summary of product characteristics or current investigator brochure) approved for the trial. Links to all RSI can be found in **Appendix C.** An event is considered unexpected if it is:

- Not listed in the RSI
- If severity exceeds what is listed in the RSI
- If frequency exceeds what is listed in the RSI
- If event outcome exceeds what is listed in the RSI

Table 32: Assessment of causality and expectedness

CAUSAL RELATIONSHIP	DESCRIPTION	EXPECTEDNESS		
		NOT RELATED	EXPECTED REACTION	UNEXPECTED REACTION
Unrelated	There is no evidence of any causal relationship	**Unrelated SAE**	***No assessment required as unrelated to treatment***	
Unlikely	There is little evidence to suggest there is a causal relationship (e.g. the event did not occur within a reasonable time after administration of the trial medication). There is another reasonable explanation for the event (e.g. the participant's clinical condition, other concomitant treatment).	**Unrelated SAE**		
Possibly	There is some evidence to suggest a causal relationship (e.g. because the event occurs within a reasonable time after administration of the trial medication). However, the influence of other factors may have contributed to the event (e.g. the participant's clinical condition, other concomitant treatments)		**SAR**	**SUSAR**
Probably	There is evidence to suggest a causal relationship and the influence of other factors is unlikely.		**SAR**	**SUSAR**
Definitely	There is clear evidence to suggest a causal relationship and other possible contributing factors can be ruled out.		**SAR**	**SUSAR**

11.3.2.E Notification responsibilities for non-protocol treatments

It should be noted that docetaxel, abiraterone and enzalutamide may be given as non-trial treatments in the management of CRPC. It is not necessary to report AEs or SAEs relating to this non-trial use where the treatment commenced post progression. Instead the yellow card system should be used to notify the regulatory authorities of adverse drug reactions in this setting: (https://yellowcard.mhra.gov.uk/)

Figure 4: SAE reporting flowchart

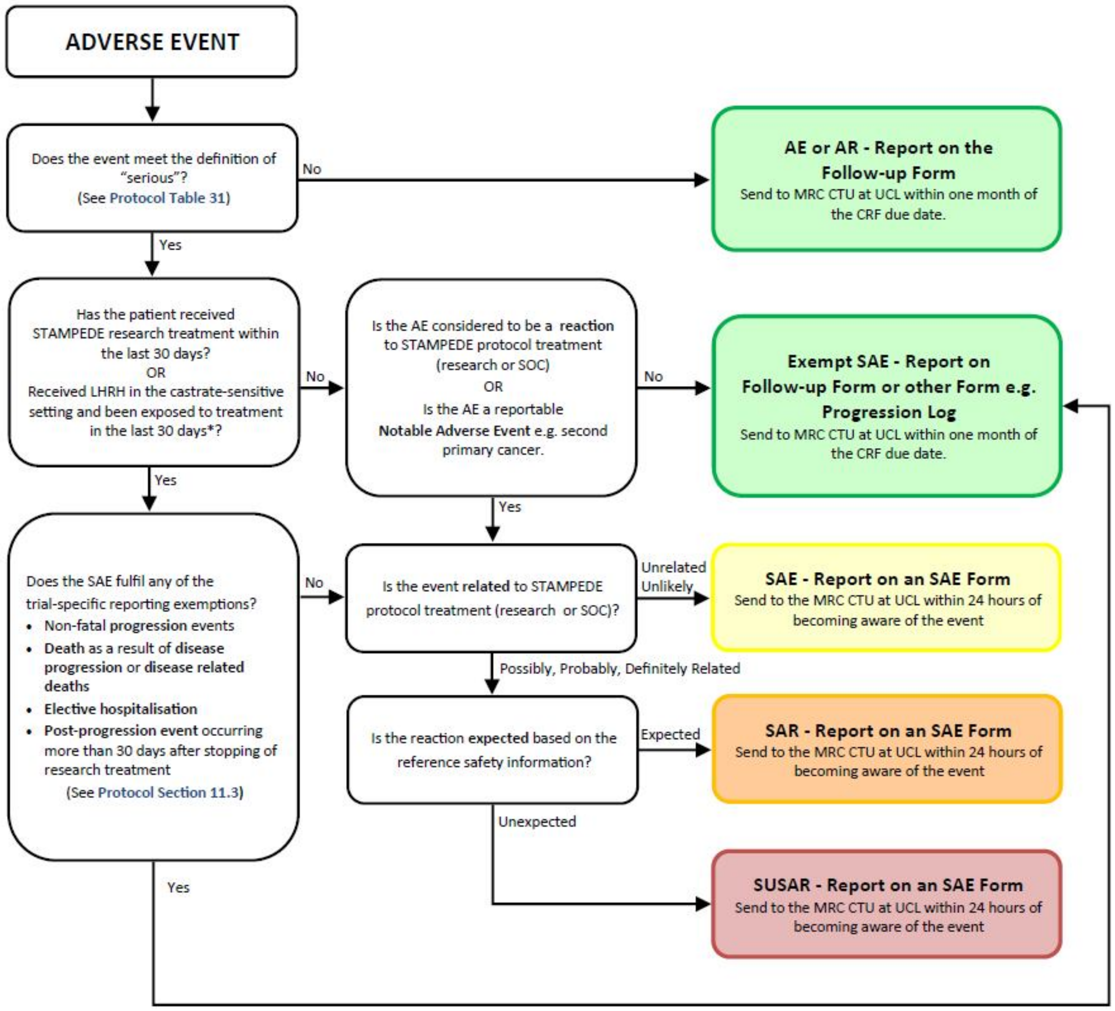

**Exposure to LHRHa is assumed to be until the depot expiration date, therefore unrelated SAEs are reportable up until 8 weeks after the administration of a 4-week depot or 16 weeks after the administration of a 12-week depot.*

Box 1: SAE report notification checklist

Before sending the SAE CRF please check that the event does not meet any of the exemption criteria, see Section 11.2. Once confirmed, please ensure that the information provided meets **all** of the following minimum criteria required for initial processing and review:

1. At least **two** patient identifiers
2. The main event or symptom being reported
3. Indication of why the event was **serious**
4. **Grade** severity of event/reaction according to CTCAE version 4.0
5. Assessment of **causality** in relation to protocol treatment (including SOC treatments)
6. Assessment of **expectedness if considered related to treatment**. Please refer to Reference Safety Information in Appendix C
7. Provide the **date of last administration** for all trial treatments (minimum month/year) – *if reporting an unrelated SAE confirm that this is still reportable*
8. **Signature** (if not by a clinician, by a site trial team member in the first instance)

SAE REPORTING
Fax to 020 7670 4818 within 24 hours of becoming aware of the event
Or send via **encrypted** email to mrcctu.stampede@ucl.ac.uk

11.3.3 Event Follow-up

Participants must be followed up until clinical recovery is complete or stabilised. Follow-up should continue after completion of protocol treatment if necessary. Follow-up information should be updated on the original SAE CRF by ticking the box marked "follow-up" and faxing to the CTU as information becomes available. Extra information and/or copies of test results may be provided separately but must be anonymised. The participant must be identified by trial ID and initials only. The participant's name should not be used on any correspondence.

11.4 CTU RESPONSIBILITIES

The STAMPEDE trial team will acknowledge receipt of all SAEs via email. Please contact the STAMPEDE trial team if an acknowledgement email is not received within 3 working days.

At least one medically qualified person at the CTU, or comparison chief-investigator or another appropriate TMG member will review all SAE reports received. The causality assessment given by the local Investigator at the hospital cannot be overruled and in the case of disagreement, both opinions will be recorded.

The CTU is undertaking the duties of trial sponsor and is responsible for the reporting of SUSARs and other SARs to the regulatory authorities (through the MHRA to competent authorities in other European member states) and the UK research ethics committees. Additionally, the CTU has sponsor oversight for reporting in other countries in which the trial is taking place. The CTU is responsible for reporting fatal and life-threatening SUSARs to the competent authorities within 7 days of the CTU becoming aware of the event; other SUSARs must be reported within 15 days.

The CTU will also keep all investigators informed of any safety issues that arise during the course of the trial.

The CTU will submit Annual Safety Reports in the form of a Developmental Safety Update Report (DSUR) to Competent Authorities (Regulatory Authority and Ethics Committee).

Any drug companies involved will also be notified of reportable (serious and unexpected and drug-related/unknown relationship) events as per their agreement with the sponsor. CTU will also provide companies with a copy of the Annual Safety Report in the required format.

12 ETHICAL CONSIDERATIONS AND APPROVAL

12.1 ETHICAL CONSIDERATIONS

12.1.1.A Randomisation

This is a randomised trial therefore neither the participants nor their physicians will be able to choose the participants' treatment. Treatment will be allocated randomly using a computer-based algorithm. This is to ensure that the groups of participants receiving each of the different treatments are as similar as possible.

All participants, with the exception of those allocated to transdermal oestradiol (Arm L), will receive standard hormone treatment. All participants, including those allocated to Arm L, may also receive other standard-of-care treatments which may include prostate radiotherapy and/or docetaxel. Use of radiotherapy and/or docetaxel will be unaffected by trial participation and is left to the discretion of the treating clinician and participant. Participants may be randomised to receive additional treatment (metformin) given with standard-of-care treatments, or an alternative form of hormone treatment (transdermal oestradiol). An even allocation ratio is being currently being used which means all eligible participants have an equal chance of being randomised to the control or research arms.

Through the introduction of a "transdermal oestradiol comparison" into the STAMPEDE trial platform, sufficient data will be collected to evaluate this treatment approach more rapidly. By undertaking a meta-analysis using data collected in both PATCH and STAMPEDE trials, fewer participants overall are allocated the control arm i.e. more participants gain access to novel treatments and results will be available sooner.

12.1.1.B Evaluation of Novel Therapeutic Strategies

There is some evidence to suggest that the newer treatment options may have advantages over standard treatment alone with regards to clinical outcome, but this is not confirmed and toxicity may be increased. This trial will follow a large group of people who have been randomly allocated to either the standard treatment(s) or the novel treatment strategies in order to measure the benefits of these approaches. The participants will also be followed-up for toxicity and safety issues, so that any benefits can be weighed against any negative aspects including the impact treatments have on other aspects of medical health e.g. cardiovascular disease, as well as quality-of-life and value for money (health economic analysis).

12.1.1.C Additional Tests and Hospital Visits

Trial participants will have some additional hospital visits and some extra blood samples compared with standard practice, the exact requirements depend on the allocated treatment and stage of disease. Efforts are made to reduce the burden of extra visits and tests, for example extra blood tests can be performed at a time when a blood draw would be performed as part of standard care, or participants can have the blood samples taken at their GP's surgery instead.

12.1.1.D Facilitating Participant Feedback From Investigations and Additional Analyses

For participants who choose to take part in additional sub-studies, biological samples including blood, saliva and remaining stored FFPE tumour samples will be used in research projects. These projects will enable the study of genetic factors and other biomarkers that can help identify individuals who serve to benefit most from the treatments tested in STAMPEDE and to further understand why and how treatment resistance develops. All samples will remain anonymised and only made accessible to approved collaborators granted access by the STAMPEDE oversight

committees. We will make every effort to protect the confidentiality of this information and make sure personal identities are protected.

From protocol v16.0 onwards, participants may opt to receive feedback regarding genetic results that may arise from the research analyses of genetic material extracted from any of the biological samples collected as part of the trial e.g. saliva, FFPE tumour blocks or circulating tumour DNA extracted from blood. Only results which are of established clinical relevance and for which testing would be available under standard NHS genetic testing guidelines will be fed back. Any genetic analysis undertaken as part of additional research associated with STAMPEDE does not replace clinically indicated investigations as only a proportion of STAMPEDE participants will undergo prospective testing and therefore it cannot be guaranteed that results will be fed back in a timely fashion.

This change has been made in response to emerging data that demonstrates a small proportion of people may have genetic faults in genes such as Breast Cancer Gene 2 (BRCA2). This has implications for both participants and potentially their biological relatives. For participants and their treating clinician, knowledge of this information may facilitate access into further clinical trials and may potentially impact on the choice of treatment following progression.

Any participant who consents to receive feedback and in whom a known pathogenic mutation of clinical significance is detected on testing of research samples collected as part of STAMPEDE will be told of this. Participants will be recommended to undergo genetic counselling accessed via clinical genetics services and consider confirmatory testing. This is necessary to determine if the defect is germline (inherited) and ensures access to appropriate ongoing support. If confirmed as a germline (inherited) abnormality, this will enable biological relatives to also access appropriate genetic counselling and testing if they wish.

The introduction of the “metformin comparison” means that all participants, not known to be diabetic, will be screened for diabetes prior to trial entry. This is to enable the effect of metformin to be studied in non-diabetic participants. All participants in whom screening bloods are abnormal will be referred for confirmatory tests and further management according to local guidelines e.g. via their GP. Screening is expected to lead to a small proportion of potential trial participants receiving a new diagnosis of diabetes but will ensure appropriate management of both conditions.

12.1.1.E Considering the Impact of Emerging Data

If new information emerges during the course of the trial which may affect the treatment or follow-up of participants all Principal Investigators (PIs) will be informed of this and required to inform trial participants.

12.1.1.F Electronic health records

Participants are requested to provide consent to permit linkage of trial data to other sources of electronic health data to improve the reliability of long-term follow-up data. Explicit consent is requested for the CTU to securely and seperately store direct identifiers (name and NHS number). This is to permit verification of the information held by others and received by the CTU, ensuring that the trial database is only updated with accurate information.

12.2 ETHICAL APPROVAL

The protocol has a Favourable Opinion from an appropriate Research Ethics Committee, according to national guidelines. Additionally, each site must also obtain management permission for research (Local R&D approval or equivalent) from the relevant host organisations before participants can be entered into the trial. The participant’s informed consent to participate in the trial should be

obtained after a full explanation has been given of the treatment options, including the conventional and generally accepted methods of treatment. Participant information sheets and participant consent forms are available on the STAMPEDE website (www.stampedetrial.org).

The right of the participant to refuse to take part in the trial without giving reasons must be respected. After the participant has entered the trial, the clinician must remain free to give alternative treatment to that specified in the protocol, at any stage, if he feels it to be in the best interest of the participant. However, the reason for doing so should be recorded and the participant will remain within the trial for the purpose of follow-up and data analysis according to the treatment option to which he has been allocated. Similarly, the participant must remain free to withdraw at any time from the protocol treatment without giving reasons and without prejudicing his further treatment.

A statement of MRC policy on ethical considerations in clinical trials of cancer therapy, including the question of informed consent, is available from the MRC Head Office web site (http://www.mrc.ac.uk). In addition, the MRC and the Wellcome Trust framework on the feedback of health-related findings in research is readily available (https://www.mrc.ac.uk/documents/pdf/mrc-wellcome-trust-framework-on-the-feedback-of-health-related-findings-in-researchpdf/) and has been used when developing the trial specific processes.

13 REGULATORY APPROVAL

This trial has been approved in the UK by the MHRA and will be conducted under a Clinical Trials Authorisation (CTA Ref: 00316/0026/001-0001) in the UK.

The trial has been approved in Switzerland by Swissmedic (Ref: 2009 DR 3235).

13.1 DATA COLLECTION & RETENTION

CRFs, clinical notes and administrative documentation should be kept in a secure location (for example, locked filing cabinets in a room with restricted access) and held for 25 years after the end of the trial. During this period, all data should be accessible to the competent or equivalent authorities, the Sponsor, and other delegated authorities with suitable notice as it may be subject to audit or inspection from any of the above.

14 INDEMNITY

University College London holds insurance against claims from participants for injury caused by their participation in this clinical trial. Participants may be able to claim compensation if they can prove that UCL has been negligent. However, as this clinical trial is being carried out in a hospital, the hospital continues to have a duty of care to the participant of the clinical trial. University College London does not accept liability for any breach in the hospital's duty of care, or any negligence on the part of hospital employees. This applies whether the hospital is an NHS Trust or otherwise.

Participants may also be able to claim compensation for injury caused by participation in this clinical trial without the need to prove negligence on the part of University College London or another party. Participants who sustain injury and wish to make a claim for compensation should do so in writing in the first instance to the Chief Investigator, who will pass the claim to the managing organisation's Insurers, via the managing organisation's office.

Hospitals selected to participate in this clinical trial must provide clinical negligence insurance cover for harm caused by their employees and a copy of the relevant insurance policy or summary can be provided on request.

15 FINANCE

STAMPEDE is funded by Cancer Research UK's Clinical Research Committee (formerly the Clinical Trials Advisory Awards Committee; CTAAC). It is also funded by the MRC through the MRC Clinical Trials Unit at UCL. The trial has National Institute for Health Research Clinical Research Network (NIHR CRN) approval and, therefore, local NCRN funds may be available at each centre to support entry of participants into this trial.

Funding arrangements for research arms now closed to recruitment can be found in **Protocol version 13.0**

Standard therapies including **ADT**, **prostate radiotherapy** and **docetaxel** will be administered as per routine clinical care using local NHS supplies.

Abiraterone is manufactured by Janssen Pharma PV (pharmaceutical companies of Johnson & Johnson). They have agreed to provide free drug, funds to distribute drug to participating sites and to help support the conduct and management of the trial.
If abiraterone is required to be given to patients as standard of care, funding will not be provided. Drug should be administered as per routine clinical care using local NHS supplies.

Enzalutamide is manufactured by Astellas Pharma. They have agreed to provide free drug and funds to distribute drug to participating sites and to help support the conduct and management of the trial.

Metformin will be administered using local NHS supplies.

Transdermal oestradiol will be administered as Progynova TS 100 patches, manufactured by Bayer who have agreed to supply these patches at a trial-specific discounted price. All accredited STAMPEDE centres will be able to order Progynova patches through AAH Pharmaceuticals Ltd wholesalers at the discounted rate.

Biomarker Screening Pilot will be funded by Clovis Oncology who will fund all sample analysis and help support the coordination of sample retrieval including site reimbursement through an educational grant to the MRC CTU at UCL.

16 TRIAL COMMITTEES

16.1 TRIAL MANAGEMENT GROUP (TMG)

A Trial Management Group (TMG) has been formed comprising the Chief Investigator; each comparison lead investigator, other co-investigators and members of MRC CTU at UCL Internal Trial Management Team. The membership of the TMG may be expanded if other groups of trialists wish to participate. It will also be amended during the trial if other circumstances require e.g. retirement.

The TMG will be responsible for the day-to-day running and management of the trial. The TMG will meet by teleconference at least on a monthly basis where possible and in person as needed. The TMG members are detailed in **Appendix F**.

Further details of TMG functioning are provided in the TMG charter (available on request).

16.2 TRIAL STEERING COMMITTEE (TSC)

A Trial Steering Committee (TSC) has been formed to provide overall supervision for the trial and provide advice through its independent chair. The ultimate decision for the continuation of the trial lies with the TSC. The TSC will meet regularly.

The relationship of the TSC with the other STAMPEDE working groups is detailed in **Figure 5**. Further details of TSC functioning are provided in the TSC charter (available on request).

16.3 INDEPENDENT DATA MONITORING COMMITTEE (IDMC)

An Independent Data Monitoring Committee (IDMC) has been formed. The IDMC will be the only group who sees the confidential, accumulating data to the trial. Reports to the IDMC will be produced by the CTU. The IDMC will meet within 6 months of the trial opening with the frequency of meetings dictated by the IDMC. The IDMC will consider data in accordance with the analysis plan (see **Section 9**) and will be advisory to the TSC. The IDMC can recommend premature closure or reporting of the trial, or that recruitment to any research arm is discontinued.

From protocol v8.0 onwards, any recommendation from the IDMC to stop recruitment to one or more trial arms will be acted upon immediately, pending ratification from the TSC. As this period between meetings should be very short, sites would not be notified until after the TSC have made a decision. IDMC recommendations based on emerging safety issues will be discussed with sites promptly.

The relationship of the IDMC with the other STAMPEDE working groups is detailed in **Figure 5**. Further details of IDMC functioning and the procedures for interim analysis and monitoring are provided in the IDMC charter (available on request).

Data from the “transdermal oestradiol comparison” are viewed by the PATCH IDMC, in meta-analysis with PATCH, rather than by the STAMPEDE IDMC. Recommendations of any actions relating to STAMPEDE would be made to the STAMPEDE TSC.

16.4 TMG SUB-GROUPS AND EXPERT PANELS

The trial has a number of TMG sub-groups and expert panels, each comprising of specific members of the TMG, MRC CTU at UCL, field experts and other STAMPEDE clinicians and site staff. The groups are all chaired by TMG members and report directly into the TMG.

- The **Biological Research Group** (BRG), the **Bone and Imaging Group** (BIG) and the **Metabolic Translational Group** (MTG) all input and provide expert oversight of relevant translational aspects of the trial and associated sub-studies.
- The **STRATOSPHere Consortium Management Group** (STRATOPHere: STratification for RAtional Treatment-Oncomarker pairings of STAMPEDE Participants starting long-term Hormone treatment) coordinates the parallel translational programme funded by Prostate Cancer UK.
- The **Comparison Management Group** (CMG) interact to propose, plan and develop new comparisons as required.
- The **Site Advisory Team** (SAT) includes STAMPEDE site research staff to provide advice to the TMG on how proposed amendments to the protocol and CRFs directly affect staff practices.
- The **Outcome Review Group** (ORG) conducts cause of death reviews as required for secondary end point analysis.
- The **Clinical Safety Committee** (CSC) review all SAEs of STAMPEDE participants and provide guidance to site clinicians and research staff in regards to clinical safety aspects of the trial.
- The **Genetic Sub-Group** (GSG) provides oversight of all results arising from genetic testing

The relationship of each of these groups with the other STAMPEDE working groups is detailed in **Figure 5.**

16.5 MRC CTU AT UCL INTERNAL GROUPS

CTU requires a number of internal working groups to run a platform protocol. These internal groups assist the TMG in the operation of STAMPEDE, providing guidance on scientific strategies of research and publication, research governance in regulatory information and protocol review and the management of research quality within the STAMPEDE trial.

The relationship of each of these groups with the other STAMPEDE working groups is detailed in **Figure 5**.

Figure 5: Organigram of the relationships between STAMPEDE working groups

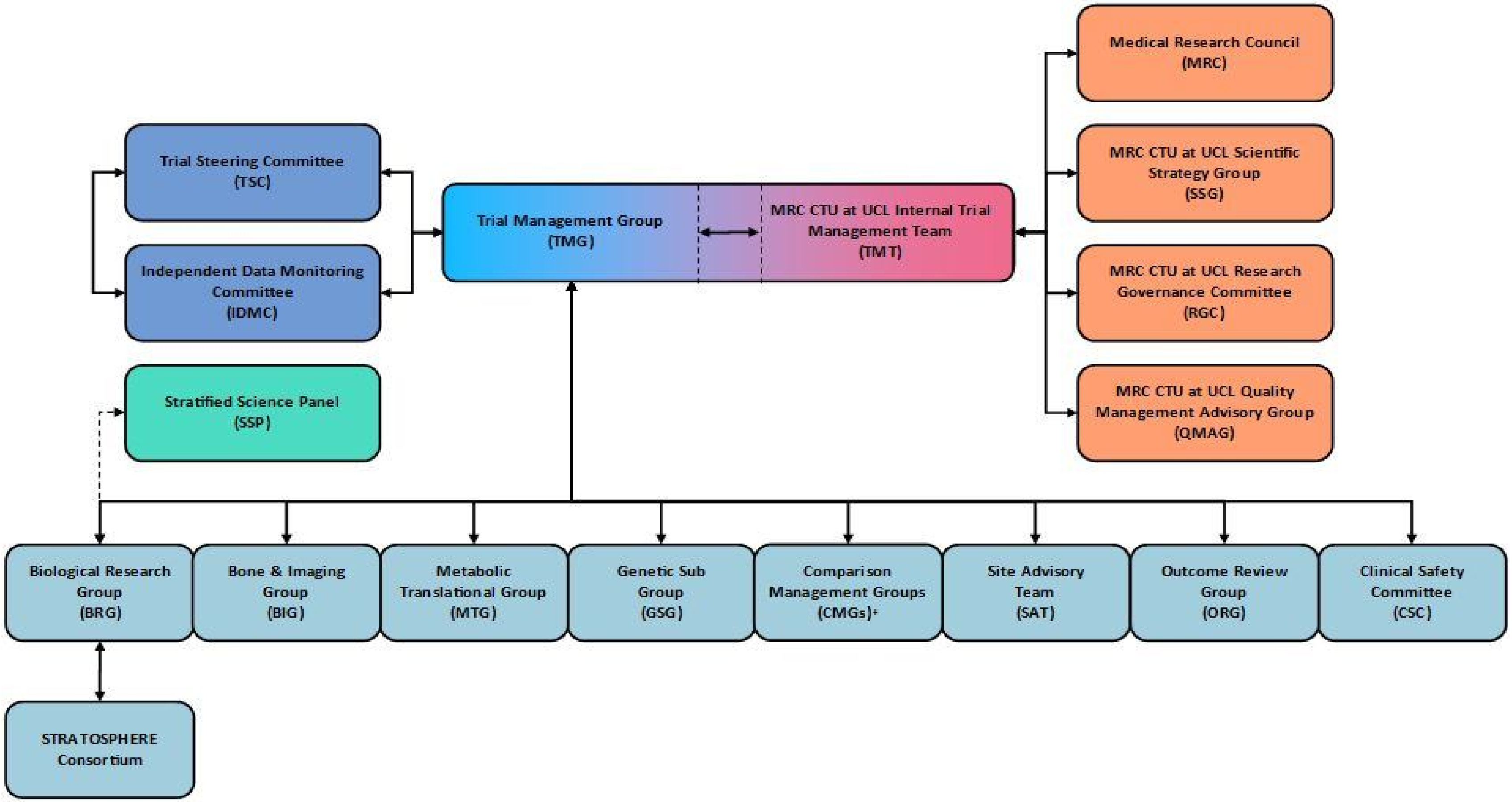

+The number and timeline of current and planned comparisons will dictate the need for the number of CMGs in operation. At any point there may be one or more.

17 ANCILLARY STUDIES

17.1 PATIENT REPORTED OUTCOMES

STAMPEDE collects patient reported outcomes in form of the EORTC QLQ-30 Quality of Life form and the EQ-5D Health Economics Form.

The research nurse should approach participants at appropriate clinical visits to complete a questionnaire. If no clinical visit is scheduled for the participant (with a window of 4 weeks around the expected date) the nurse should organise the completion of the questionnaire e.g. by post. Questionnaires should be self-administered; participants should be encouraged to complete the questionnaires without conferring with friends or relatives and all questions should be answered even if the participant feels them to be irrelevant. The research teams should encourage the participants to answer all questions but should not review the responses as these should remain confidential.

17.1.1 Quality of life (QL)

The EORTC QLQ-C30 with the prostate-specific module QLQ PR25 will be used. Key items for assessment are pain reduction for participants with metastatic disease and urinary symptoms for participants with locally-advanced disease. In addition specific hypotheses will be generated for each of the research arms.

17.1.1.A Changes in QL data collection from protocol v19.0 onwards

Initial participation in the QL sub-study was limited to the first 700 participants recruited (this was reached in Sep-2008). After a pause, the QL sub-study re-opened from the implementation of protocol version 8.0 (Nov 2011 onwards).

From protocol v19.0, QL and HE data collection will change, as laid out in **Table 33.** QL and HE collection stops for most participants but continues as planned in participants in the “abiraterone comparison” and “abiraterone and enzalutamide comparison” and becomes lifelong in participants in the “metformin comparison”. HE collection (without QL) will also continue in participants in the “M1|RT comparison” randomised after Apr 2016.

Table 33 summarises the participant reported outcome data collection (QL and HE) by comparison. Going forward, for each new comparison within STAMPEDE a pre-defined sample size for the participant reported outcomes will be described and a sampling approach considered where appropriate.

17.1.2 Health Economics

The EuroQol (EQ-5D) will be used in the study as a generic measure of health-related quality-of-life which can be linked to public preferences. These data will be used to calculate quality-adjusted life-years as part of the economic evaluation. Healthcare resource use will be collected at each follow-up. This includes non-trial inpatient days, non-trial outpatient, GP visits and data on concomitant medications. Information on participants’ use of primary care and community-based services will also be collected as additional questions in the questionnaire. Costs will be calculated on the basis of representative UK unit costs at the point of analysis. A cost-effectiveness analysis will compare all regimens that continue to recruit into their final Efficacy Stage. For further details please refer to **Appendix G**.

Table 33: Patient reported outcome data collection by comparison

COMPARISON	PARTICIPANT DETAILS	COLLECTION OF PATIENT REPORTED OUTCOMES<br>E.G. EORTC QLQ-C30, EQ-3D
“Original”	Arms B, C, D, E, F and Arm A recruited between trial start (2005) and 15-Nov-2011	No further collection of participant reported outcomes as comparisons have closed to follow-up
“Abiraterone”	Arms A and G randomised between 15-Nov-2011 and 17-Jan-2014	Data collection to continue until disease progression or 5 years post randomisation (i.e. all data collection stops 17-Jan-2019).
“Abiraterone and enzalutamide”	Arms A and J randomised between 27-Jun-2014 and 31-March 2016	Data collection to continue until disease progression or 5 years post randomisation whichever occurs first.
“M1|RT”	Arms A and H randomised between 30-Jan-2013 and 02-Sep-2016	From protocol v19.0 QL and HE data collection will stop for all participants recruited to Arm H prior to April-2016.<br>HE (EQ-5D) data collection continues lifelong for all A and H participants randomised between Apr-2016 to Sep-2016.
“Metformin”	Arms A and K randomised since 05-Sep-2016	From protocol v19.0 the QL and HE sub-studies are closed to newly randomised participants<br>For all existing arm A and K participants (i.e. randomised prior to activation of protocol v19.0) data collection continues at each follow-up lifelong.
“Transdermal oestradiol”	Arms A and L randomised since 20-Jun-2017	From protocol v19.0 the QL and HE sub-studies are closed to newly randomised participants within this comparison. QL data will be collected through the PATCH trial.

17.2 TRANSLATIONAL SUB-STUDIES

Samples obtained from consenting STAMPEDE participants are analysed as part of separate translational sub-studies. These are conducted through collaborations with other academic and industry partners. All applications for collaboration and sample accesss are reviewed by the STAMPEDE oversight committees and overseen by the STAMPEDE BRG. For details on eligibility criteria for each translational sub-study refer to **Section 4.7.** For details regarding sample collection refer to the **Sample collection and handling manual** available via the website.

17.2.1 Germline DNA Analysis

DNA is being extracted from saliva samples provided by consenting participants enrolled in STAMPEDE. The purpose of this sub-study is to examine the germline (inherited) genetic changes present in people with high-risk localised or metastatic prostate cancer. The aim is to determine the prevalance of germline genetic aberrations present pre-diagnosis and to correlate prostate cancer risk single-nucleotide-polymorphisms (SNP) genetic profiles, identified in Genome-wide Association Studies (GWAS) and other sequence variants from next generation sequencing (NGS), with duration of response to ADT and the experimental treatments tested in STAMPEDE.

All newly randomised trial participants who join arms A, K or L are eligible to join this sub-study. For details relating to Saliva sample collection and shipping refer to the **Sample collection and handling manual.**

17.2.2 Circulating Tumour-DNA Analysis (Sequential Blood Samples)

The aims of this analysis include to identify molecular subgroups with differential treatment effects and, through sequential sampling, identify molecular changes associated with disease progression to explore resistance mechanisms and early detection of treatment failure.

Sequential samples are required in order to detect genetic changes within tumours over time. The most important sampling timepoint is at progression, as it is hoped this can inform the potential mechanisms of treatment resistance. The sampling schedule is different for M0 and M1 participants and is detailed in the **Sample collection and handling manual**.

From protocol v14.0 (activated from Jan-2014 onwards), sequential blood samples were collected from participants within the “enzalutamide and abiraterone” comparison, i.e. allocated to arm A or J between 29-Jul-2014 and 31-Mar-2016. From protocol v16 onwards, **all participants** may be invited to join this sub-study.

For eligibility criteria for the circulating tumour DNA sub-study refer to **Section 4.7.2.**
For details relating to blood sample collection and shipping refer to the **Sample collection and handling manual.**

17.2.3 Tissue Sample Analysis (FFPE Blocks)

As the clinical outcome data matures for several of the treatments comparisons evaluated within STAMPEDE, correlative analysis of the archival formalin-fixed paraffin-embedded (FFPE) tumour tissue will be undertaken, aiming to identify if genetic mutations present in prostate cancer cells pre-treatment predict how well each treatment works. In addition, projects providing preliminary prevalence and feasibility data to inform future biomarker-directed randomisations will be conducted.

From 2016 onwards, the CTU has been coordinating the retrieval of archival tumour blocks from selected consenting STAMPEDE participants. These samples are usually stored as FFPE tissue blocks

at the hospital where the procedure was performed. Randomising sites will be asked to assist in the retrieval of FFPE samples when these are requested. Research teams will be required to confirm sufficient consent has been provided and to provide an anonymised copy of the relevant consent form. If not done so already, an anonmysised copy of the consent form should also be sent to the CTU, as per **Section 10.1.1.**

For further details on where to check sufficient informed consent, sample processing and shipping and reimbursement, see the **Sample collection and handling manual.**

17.2.4 Biomarker-Screening Pilot

In preparation for the introduction of biomarker-selected comparisons, piloting of biomarker-screening is being undertaken in a limited number of sites selected by the MRC CTU at UCL. Sites will be invited to participate and sub-study specific training provided.

All patients who fulfil the criteria should be **registered without delay** in order to proceed to biomarker-screening. Please refer to the **Biomarker-screening manual** for details regarding the eligibility criteria.

Once eligibility is confirmed complete the registration CRF and contact the CTU using the randomisation line (see **Section 5.1**). At the point of registration the required samples (FFPE Tumour block, blood and saliva sample) should be sent for centralised testing. Please refer to the **Biomarker-screening manual** for further details.

All participants in the Biomarker-Screening Pilot will be allocated a registration number which relates specifically to the biomarker-screening process. The registration number will be used to identify the patient until the point of randomisation when this will be linked and replaced by the trial number.

Registration will occur **before** the patient is randomised. In the pilot phase, results of biomarker-screening are **not** required prior to randomisation as recruitment has not yet been activated to any biomarker-selected comparison. Therefore participants can be randomised straight after being registered and may be allocated to any of the current open arms for which they are eligible (arm A, L and K). For further details on results and feedback to participants, please see **Section 17.2.5**.

17.2.5 Informed consent to receive results arising from genetic sub-studies

For participants joining the trial from protocol v16.0 onwards (activated June-2017), the consent process has been updated. Trial participants are asked to provide explicit informed consent if they wish to receive feedback of any results that arise from research analyses of genetic material extracted from any of the biological samples collected as part of the trial e.g. saliva, FFPE tumour blocks or circulating tumour DNA extracted from blood.

Only results which are of established clinical relevance and for which testing would be available under standard NHS genetic testing guidelines will be fed back e.g. pathogenic BRCA2 mutations. Any genetic analysis undertaken as part of additional research associated with STAMPEDE does not replace clinically indicated investigations as only a proportion of STAMPEDE participants will undergo prospective testing. Analyses are conducted on a purely research basis and it cannot be guaranteed that results will be fed back immediately.

STAMPEDE investigators are strongly recommended to refer all participants in whom a clinically relevant genetic result is detected during research analyses to a clinical geneticist. This is to facilitate access to genetic couselling and the required confirmatory testing. This is also necessary in order to

offer appropriate advice to biological relatives in the event of confirmatory testing detecting a germline (inherited) abnormality. The list of clinically relevant gene mutations to be fed back will be based on current clinical guidelines. The STAMPEDE Biological Research Group will review this periodically to ensure it remains current and to oversee this process.

In the information provided to STAMPEDE participants who joined the trial prior to Protocol version 16.0, it was stated that any subsequent genetic results would not be linked to them or their families and therefore results will not be provided in this instance. It is possible for trial participants to update their consent by re-consenting to the current Additional Research Consent Form. This should be anonmysised and sent to the CTU as per standard procedures, see **Section 10.1.1.**

17.3 DISEASE VOLUMETRIC ANALYSIS SUB-STUDY

Baseline imaging obtained from STAMPEDE participants are accessed and analysed as part of the trial data collection. Collection and analysis will be undertaken in collaboration with partners on the TMG, initially, in ordered to determine disease volume. For details partaking to retrospective imaging centralisation and image handling, please refer to the individual sub study Working practices available from CTU. All subsequent applications for collaboration and imaging access are reviewed by the STAMPEDE oversight committees following the usual processes.

17.4 USING ROUTINE DATA TO IDENTIFY CLINICAL TRIAL OUTCOMES

This sub-study is developing methods to explore whether routine data can be used to quickly and accurately capture trial-related events in centrally-held datasets. These methods need to be developed and validated using different sources of routine data and to identify different types of events. These data sources include, but are not limited to, data from the Public Health England (PHE) National Cancer Registration and Analysis Service (NCRAS), the National Radiotherapy dataset (RTDS) and the Systemic Anti-Cancer Therapy dataset (SACT) and NHS Digital, Hospital Episode Statistics (HES), and Office of National Statistics (ONS) data.

The overall aim of this sub-study is to develop a clinically useable tool, to accurately identify disease driven events and trial outcomes, to help reduce the burden of collecting trial data from traditional participant-investigator contact. By using data that has already been accurately collected in patients that have given appropriate consent, or for whom the appropriate permissions are in place (e.g. via the Confidentiality Advisory Group), it may be possible to improve timeliness, reduce costs and save resources. Development of enhanced ways to obtain trial data is being undertaken, to recalculate analyses already carried out but also to perform secondary analyses not possible with conventionally collected trial data. The projected aim is to utilise validated methods for routine follow-up and/or analysis in the future, as outlined in the protocol, longer term outcome data may be sought via routine data sources.

18 PATIENT AND PUBLIC INVOLVEMENT

Patient and Public Involvement (PPI) in research is defined by INVOLVE (an advisory group established by the NIHR) as research being carried out 'with' or 'by' members of the public rather than 'to', 'about' or 'for' them. INVOLVE intends 'public' to include patients, potential patients, carers and other users of health and social care services, as well as people from organisations that represent people who use services. In some cases, this may include involvement of a trial's participants in guidance or oversight of a trial.

18.1 POTENTIAL IMPACT OF PPI

PPI is in place to have constant patient overview and investment to guide research. Ultimately STAMPEDE has been created to test whether alterations in treatment help to improve outcomes and quality of life of patients. It is essential to have patients' input as they understand what other patients are going through

The nature of STAMPEDE is such that, even after a main analysis of a comparison has been performed, other participants are still being recruited to other arms. We have a duty to participants and the public to disseminate findings and results, both negative and positive. With this in mind, participants are periodically provided with study findings and updates. Study findings are also presented at conferences.

18.2 PATIENT REPRESENTATIVES

Patient representatives are actively involved in the management of STAMPEDE including updates and alterations. Part of their role is to review all material that will enter the hands of a patient or family member. This is to ensure all documentation used is clear, concise and has wording that is appropriate for everyone, as well as conveying the intended information. Patient representatives sit on the Trial Management Group (TMG).

19 PUBLICATIONS

The results from different centres will be analysed together and published as soon as possible. Individual clinicians must not publish data concerning their participants that are directly relevant to questions posed by the study until the TMG has published its report. The TMG together with the STAMPEDE collaborators will form the basis of the writing committee and decide on the nature of publications. For the "transdermal oestradiol comparison", as the efficacy analyses will based on relevant data from the STAMPEDE and PATCH trials, TMGs for the two studies will form the writing committee. Any release, of efficacy or safety data, presentation or publication will be agreed with the TSC according to the terms of their charter.

All publications will acknowledge the participating centres and clinicians, and these will be detailed in an appendix to the main report. Papers will have named authors determined by the TMG according to the following principles:

- To be as inclusive as possible where this is practicable
- To ensure that there is justification for anyone to be named as an author

Reasons for nomination for authorship may include: trial design; grant holding; day-to-day trial oversight (TMG membership); analysis; discussion and interpretation of data; representation for key groups; active participation at large recruiting sites.

It should be accepted that the people qualifying for authorship will vary over time. In addition, key positions will vary depending on the nature of the publication: clinical lead for clinical papers, statistician lead for methodology papers, translational papers may be led by authors not on the main TMG if appropriate (e.g., the bone sub-study). In the event of any dispute related to authorship or data release, the TSC will be responsible for making the executive decision.

In the manuscript, a full list of sites and the number of participants recruited will be provided. In the presentations, this list of sites will also be shown. The term "the STAMPEDE investigators" will clearly be stated and relevant names included in the presentation credits.

A detailed **Publication Plan** is documented separately.

20 DATA AND/OR SAMPLE SHARING

Data will be shared according to the CTU's controlled access approach, based on the following principles:

- No data should be released in response to a data release request that would compromise an ongoing trial, unless specifically for safety reasons.
- There must be a strong scientific or other legitimate rationale for the data to be used for the requested purpose.
- Investigators who have invested time and effort into developing a trial or study should have a period of exclusivity in which to pursue their aims with the data, before key trial data are made available to other researchers.
- The resources required to process requests should not be under-estimated, particularly successful requests which lead to preparing data for release. Therefore adequate resources must be available in order to comply in a timely manner or at all, and the scientific aims of the study must justify the use of such resources.
- Data exchange complies with Information Governance and Data Security Policies in all of the relevant countries.

Data will be available for sharing on successful request and after the main publication for each comparison. Researchers wishing to access STAMPEDE data should contact the TMG via the CTU team in the first instance.

21 PROTOCOL AMENDMENTS

21.1 PROTOCOL

21.1.1 Amendments Made To Protocol Version 1.0 (May-2004)

Administrative changes such as typos, word change etc.

Name additions/changes to:

TMG members

TSC members

IDMC members

'General Information' Section – additional information re. Abridged version of protocol

Section 1.2 – Figure 1, Celecoxib duration amended

Section 1.3 – Figure 2, addition of cardiovascular assessment form, name and timings amended

Section 2.3 – Docetaxel information updated

Section 2.4 – Additional text re dose and duration justification for Celecoxib use.

Section 3 – Title change and content updated

Section 4.2 – New exclusion criteria added

Section 4.3.1 – New investigations added and additional text re testosterone measurements and additional text re. prior celecoxib treatment

Section 6.1.4 – Celecoxib duration amended

Section 6.1.5 – Additional text re. Co-administration of docetaxel and bisphosphonates

Section 6.1.6 – Celecoxib duration amended

Section 6.2.2 – additional docetaxel information

Section 6.2.3 – addition of CVS event history

Section 11 – Safety reporting updated

Section 12.1 – Additional text re. the collection of blood for genetic and serum marker studies

Section 15 – Additional information re. Central Subvention for docetaxel arms

21.1.2 Amendments Made To Protocol Version 1.1 (May-2005)

Section 6.2 Administration and Dose Modifications, subsection 6.2.1 Zoledronic Acid

21.1.3 Amendments Made To Protocol Version 2.0 (Jun-2005)

General Information section – SAE reporting fax number and timeframe added.

Section 1.2 – Addition of anti-androgen use for M0 patients as a method of HT

Section 1.2 – Increase in amount of blood needed & addition tissue sample request.

Section 1.3 Trial Documentation updated to include new table detailing trial documentation ahead of accreditation, the inclusion of the radiotherapy forms and correct case report form timings

Section 2.1 – Addition of anti-androgen use for M0 patients as a method of HT

Section 4.1.3 – Inclusion criteria Vii "Normal testosterone prior to hormone treatment" removed.

Section 4.1.3 - φnote has been omitted and moved to section 4.2 (see number 8)

Section 4.2 – Exclusion criteria added to exclude patients with active peptic ulceration, gastrointestinal bleeding and inflammatory bowel disease.

Section 4.2 – Exclusion Criteria added to exclude patients with planned major dental work

Section 4.3.1 - All blood test timelines changed from 14 days to 28 days.

Section 4.3.1 – Hormone Therapy pre-randomisation deadline extended from 4 weeks to 12 weeks.

Section 4.3.1 – Additional information regarding the use of NSAIDs and cox-2-inhibitors before coming on to the STAMPEDE study and once commenced on study treatment

Section 4.3.2 – Updated to ask for all vitamins and minerals the patient is taking to be recorded.

Section 4.3.3 – Updated to include the extra blood required and the request for consent of patients' tissue samples.

Section 6.1.1 – Addition of anti-androgen use for M0 patients as a method of HT

Section 6.1.6 – Addition of the calcium & vitamin name "calcichew".

Section 6.6.2 – asking also to collect vitamins and minerals under concomitant medication.

Section 6.6.3 – New section to inform investigators that patient's, who they wish to give radiotherapy to, are also eligible for STAMPEDE

Section 6.6.4 – New section to detail what data is being collected on the radiotherapy given to patients.

Section 7.1; figure 4 – Addition of radiotherapy form and in note, addition of AA alone

Section 7.1.2 – omission of repeated scans and x-rays at 24 weeks, also omitted in note under figure 4.

Chapter 11 – Safety reporting section updated

Section 17.3 – Increase in amount of blood needed & additional tissue sample request.

21.1.4 Amendments Made To Protocol Version 3.0 (Jul-2006)

Front Cover - NCRN logo added for accuracy

Front Cover - Clarification that protocol developed with NCRI rather than on behalf of

Front Cover - Clarification that it is a 6 arm trial

General Information section - MRC CTU staff section updatedyyyy

Section 1.2 – Statistics section updated.

Section 1.2 - Additional research paragraph updated to reflect additional studies and for clarification of terms

Section 1.2 - Blood collection volume changed to reflect new technique used

Section 1.3 (figure 3) - Table showing case report form schedule updated to reflect clarification of follow-up schedule and addition of new CRF (End of Treatment)

Section 2.2 - AS changed to HT (clarification of terms)

Section 2.3 - Updated in information in regard to use of docetaxel added to reflect up to date practice

Section 2.5 - Sub-headings numbered for consistency

Section 3.0 - Information in regard to the Pilot Phase now written in past tense as Pilot Phase has now been completed

Section 4.1.1 - Inclusion criteria extended so that patients who fulfil 2 out of the three of the first inclusion criteria can be eligible.

Section 4.3.1 - Change in time scales by which baseline investigations need to be completed.

Section 4.3.1 - Clarification that chest X-ray is only required if chest is not included in the CT

Section 4.3.1 - Removal of 12 week timeline for baseline PSA test to be performed. (Stipulation that it must be performed before start of HT)

Section 4.3.2 – Information added in regard to time allowed from randomisation to start of treatment

Section 4.3.3 - Additional research paragraph updated to reflect additional studies and for clarification of terms

Section 4.3.3 - Blood collection volume changed to reflect new technique used

Sections 6.1.2-6.1.6 - Androgen Suppression replaced with hormone therapy for consistency of terms

Section 6.2.2 - '(Taxotere)' Removed for consistency

Section 6.2.2 _ information added in regard to the need to closely monitor liver function prior to docetaxel administration

Section 7.1 - Page number reference updated

Section 7.1.1 - PSA measurement timings updated to accurately reflect follow-up schedule

Section 7.3 (Table 4) - Table and key updated to accurately reflect follow-up schedule and to include information about new CRFs and removal of withdrawal CRF

Section 8 - Rewording for clarification of definition of trial withdrawal

Section 8.1 - Instruction that withdrawal from trial treatment should be recorded on End of Treatment Form rather than withdrawal form

Section 8.1 - Information updated to emphasise that trial treatment must be discontinued following a progression

Section 8.2- Information added in regard to patient transfers

Section 8.3 - Instruction that withdrawal from trial completely must be notified in writing to the MRC CTU rather than included on withdrawal form

Section 9 and Summary – Target event numbers updated to reflect the slightly revised numbers obtained by using –nstage- which is the new, recommended program for MAMS trials

Sections 11.1 and 11.2 - Form numbers removed to allow for future changes in numbering

Section 11.2 – Reference to toxicity grading website added

Section 11.2.1 - Reference to table in appendix G added

Section 12.2 - 'Suggested' removed from 'Suggested patient information sheets'

Section 13 - CTA reference added

Section 17.3 - Information added to reflect new blood collection method for DNA analysis and in regard to additional translational studies for which funding has recently been approved

21.1.5 Amendments Made To Protocol Version 4.0 (Dec-2007)

General Information Section - Randomisation and SAE reporting details sections clarified

Section 1.2 and throughout protocol - Efficacy Stages 1-111 renamed to Activity Stages 1-111 for accuracy and clarity

Section 1.2 - Follow schedule corrected

Section 4.1.2 - Inclusion criteria widened to include high risk relapsing patients, that would not have met the previous PSA based criteria

Section 4.1.3 - Note added to reference location of WHO performance status definitions

Section 4.2 - Notes added to reference locations of toxicity gradings and NYHA classifications

Section 4.3.1 - Timings of baseline scan information changed to accurately reflect most common current practice

Section 6.1.1 - Information about use of LHRH antagonists to ensure that the protocol accurately reflects current and future practice

Section 6.1.1 - Information about suggested duration of hormone therapy added to ensure that the protocol accurately reflects current practice

Section 6.2.2 - Additional information added about the timing of liver function tests prior to docetaxel administration added for clarity

Section 6.6.4 - Information on radiotherapy data collection added

Section 7.1.1 - Erroneous information about the timing of PSA measurements removed

Figure 3 - Moved to new section in protocol for clarity and extended to include current information on data collection

Figure 3b - Added to describe how extent of data collection during follow-up should change, post treatment and post progression

Figure 4 - Notes added to explain the changes in data collected at follow-up and to information that the quality-of-life study will be applicable to the first 700 patients randomised only

Figure 4 - Note added to include palliative radiotherapy CRF

Section 11.3 - SAE reporting information updated

Section 19 - Protocol amendments list updated

21.1.6 Amendments Made To Protocol Version 5.0 (Aug-2008)

1. General Information Section – Randomisation phone line number updated – non UK extension added
2. Section 3 – Information about QL study removed to reflect closure of QL study after first 700 patients
3. Section 4.2 – Exclusion criteria clarified to explain that only patients with severe poor cardiovascular history should be excluded
4. Section 4.3.1 – Information on co-administration of NSAIDS with celecoxib changed based on clinical advice.
5. Section 5 - Randomisation phone line number updated – non UK extension added
6. Section 6.2.1. – Information added to clarify that patients who develop an osteonecrosis of the jaw should stop zoledronic acid treatment
7. Section 6.2.3 – ‘severe’ text added to accurately reflect which patients should be excluded based on their cardiovascular history
8. Section 7.1.2 – Definition of disease progression extended for clarity
9. Figure 3 – Updated to include reference to newly created skeletal related event form

10. Figure 4 – Previous error in table amended to show that the 4th Zoledronic Acid form that is submitted contains information about 3 cycles rather than 2 as previously indicated

11. Table 4 – 'Other important medical condition' added to definition of serious in the SAE section, to accurately reflect SAE form and current practice

12. Section 11.1 – Information added on reporting or pregnancies

13. Section 17 - Information about QL study removed to reflect closure of QL study after first 700 patients

21.1.7 Amendments Made To Protocol Version 6.0 (Jul-2009)

1. General Information Section – Trial Pharmacist removed and changes of:

Co-Investigator

Patient Representatives

Trial Manager

Data Manager

General Information Section - Coordinating Centre – address change

General Information Section – change of Sponsor address

Section 1.1 – ratio of patients randomised to the investigational arms updated

Section 1.2 – figure 1b added to clarify trial design from Apr-2011 onwards

Section 1.2 – paragraph added to explain trial changes after the second activity analysis

Section 1.2 – wording added to clarify that QL data only collected for first 700 patients randomised

Section 1.3 – SSA Favourable Opinion removed from list of trial documentation required ahead of site accreditation

Section 2.1 – Amount of people diagnosed with prostate cancer annually updated

Section 2.4 –note added to explain completion of recruitment to celecoxib- containing arms

Section 2.5.2 - note added to explain completion of recruitment to celecoxib- containing arms

Section 3 – SSA Favourable Opinion removed

Section 4.2 – Exclusion criterion xiii greyed out

Section 4.3.1 – paragraph removed regarding potential randomisation to celecoxib-containing arms

Section 5 – Randomisation instructions expanded to exclude public holidays or dates when notice has been given by the CTU

Section 6.1.4 – formatting changed to grey font to reflect recruitment completion for arm D

Section 6.1.6 - formatting changed to grey font to reflect recruitment completion for arm F

Section 6.2.3 – recruitment note added

Section 6.6.3 – radiotherapy statement changed to reflect data from recent trials

Section 7.1.2 – removal of reference to SRE- specific CRF

Section 7.3 – Figure 3 - Addition of Bone Density Risk Factor Form and BMD sub-study assessment forms to summary of timing table

Section 7.3 – Figure 4 – Weeks added to timings of assessments post 2 years

Section 7.3- Figure 4 – note added to explain recruitment completion for arms D and F

Section 12.1 – Wording changed to reflect change to randomisation allocation ratio

Section 12.1 – Addition of statement regarding new information emerging during the trial

Section 12.2 – Reference to SSA removed

Section 16.3 – Statement added regarding actioning IDMC recommendation ahead of TSC ratification

21.1.8 Amendments Made To Protocol Version 7.0 (Jul--2011)

1. General Information Section- SAE reporting fax number corrected

2. Section 11- SAE reporting fax number corrected

21.1.9 Amendments Made To Protocol Version 7.1 (Jul-2011)

Throughout protocol – numbering has been updated in some sections new accommodate new information that has been added.

General Information Section – contact details updated

General Information Section – Funding information updated to include involvement from additional company

General Information Section – Wording on compliance and regulations updated to reflect current MRC CTU standard wording

General Information Section – Abbreviations list updated

Section 1.1 – The number of investigational agents being studied updated from three to four

Section 1.1 – Information regarding celecoxib updated to reflect that recruitment to these arms was discontinued in Apr-2011

Section 1.1 – Information about new IMP, Abiraterone inserted

Section 1.1 – Sample size and trial duration information updated to reflect changes brought about by additional trial arm

Section 1.2 – Summary information updated to reflect the discontinuation of recruitment to celecoxib arms and the addition of abiraterone

Figures 1a, b and c - Updated to reflect the discontinuation of recruitment to celecoxib arms and the addition of abiraterone

Section 1.2 – Information on trial stages updated to reflect changes brought about by additional trial arm

Section 1.2 – Information updated regarding the re-opening of the quality-of-life sub-study from implementation of protocol version 8.0

Section 2.1 – Wording related to hormone therapy updated for clarity

Section 2.1 - Updated to reflect the discontinuation of recruitment to celecoxib arms and the addition of abiraterone

Section 2.2 – Updated references added

Section 2.3 – Updated references added

Section 2.5 – Section added to give background information on new IMP, abiraterone

Section 2.6.1 – Updated references added

Section 2.7 – Section added to give information regarding radiotherapy which is to be given as part of standard care following recently published trial data.

Section 3 – Wording updated regarding selection of investigators to reflect current MRC CTU practice

Section 4.1 – Inclusion criteria updated with new criterion regarding radiotherapy use

Section 4.1 - Inclusion criteria updated with new criterion regarding contraceptive use

Section 4.1 – Wording of inclusion and exclusion criteria updated for clarity

Section 4.1 – Exclusion criteria updated with new criterion regarding acceptable liver function for trial entry

Section 4.1 – Exclusion criteria updated with specifics related to blood pressure levels

Section 4.1 - Exclusion criteria updated with new criterion regarding concomitant medications

Section 4.1 - Exclusion criteria updated with new criterion regarding prior treatment with abiraterone

Section 4.1 - Exclusion criteria updated with new criterion regarding prior treatment with chemotherapy

Section 4.1 - Exclusion criteria updated with new criterion regarding prior treatment with zoledronic acid

Section 4.3 – Wording updated to reflect that patients who initially fail screening can be re-screened at a later date

Section 4.3.2 – Wording updated regarding prior anti-androgen and LHRH use updated for clarity

Section 5.1 – Co-enrolment guidelines information updated to describe newly created co-enrolment CRF

Section 6.1 – Trial treatment information updated to reflect the fact that anti-androgens alone will be no longer permitted as hormone therapy

Section 6.1.1 – Updated to describe patients for whom radiotherapy should be given as standard practice

Section 6.1.1 a and b - Sections added to give information regarding radiotherapy treatment

Section 6.1.1-6.1.6 – References to further sections updated

Section 6.1.7 – Section added to describe abiraterone treatment

Section 6.2.4 - Section added to describe abiraterone treatment

Section 6.6 - Section added to give information regarding radiotherapy treatment

Section 7.1.1 – Reference to blood being taken at patient's home removed as this does not occur in practice

Section 7.1.2 – Wording updated regarding the reporting of biochemical failures for clarity

Section 7.1.2 – Wording updated regarding skeletal-related events for clarity

Section 7.1.3 – Section added to describe additional assessments required related to abiraterone treatment

Section 7.1.4 – Section added to provide information on when treatment should commence

Figure 4 – Updated for clarity regarding return of BMD sub-study forms, the addition the co-enrolment CRF and the description of the re-opening of the QL Sub-study.

Figure 5 – Updated with reference to abiraterone and co-enrolment form

Section 7.3 - Wording on trial closure updated to reflect current MRC CTU standard wording

Section 8.1 – Additional criteria for definition of progression added for clarity

Section 8.1 – Definition of progression for abiraterone patients added.

Section 9 – Statistical information updated to describe the addition of the new trial arm

Section 11 – Safety reporting wording updated for clarity

Section 11 – SAE reporting fax number updated

Section 12 – Ethical information updated to describe the unequal randomisation allocation ratio

Section 12 – Ethical information updated to describe that the visit schedule will vary according to trial arm

Section 12.2 – Wording updated to reflect international participation in the trial

Section 13 – Wording updated to reflect international participation in the trial

Section 14 – Wording updated to reflect international participation in the trial

Section 15 - Updated to reflect the discontinuation of recruitment to celecoxib arms and the addition of abiraterone

Section 16 – Reference to trial committee charters added for information

Section 17.1 – Information added to reflect re-opening of quality-of-life sub-study

Section 17.2 – Timing of health economics analysis updated to previous error

Section 18 – Information on publication policy expanded for clarity

Section 19 – Information regarding amendments to protocol appendices moved to the separate appendices document

Section 20 – References extensively updated

21.1.10 Amendments Made To Protocol Version 8.0 (Sep-2011)

Throughout protocol – numbering, sections headings, tables, figures and bibliographical references have been updated in some sections to accommodate new information that has been added

Throughout protocol – Androgen Deprivation Therapy has replaced Hormone Therapy as deemed more representative of the type of hormone therapy used in the study

General Information Section – New staff members of the MRC CTU and Co-Investigators added and contact details updated

General Information Section – Abbreviations list updated

Section 1.1 – Information regarding the new research radiotherapy treatment inserted

Section 1.1 – Information regarding docetaxel updated

Section 1.2 – Wording updated to reflect the addition of the new research comparison arm

Section 1.3 – Additional criteria for the re-accreditation of participating centres (for protocol version 9.0 only)

Section 2.1.1 – Wording updated to clarify the use of anti-androgen in trial patients

Section 2.1.2 – Information added to describe the rationale for the RT comparison arm

Section 2.8 – Information added to describe research RT treatment to prostate for patients with newly diagnosed metastatic disease

Section 3.1 – Information added to describe RT Quality Assurance procedures and centre accreditation

Section 4.1.1 to 4.1.3 – Wording updated to clarify inclusion criteria for all patients groups (newly diagnosed non-metastatic, metastatic and relapsing patients)

Section 4.2 – Clarification added on cardiovascular exclusion criteria

Section 4.2 – New exclusion criterion added concerning patients with prior exposure to hormone therapy

Section 4.2 – New exclusion criterion added to reflect the addition of the new RT comparison arm

Section 4.4.1 – Clarification added regarding pre-randomisation checks

Section 4.4.2 – Clarification added regarding permissible hormone therapy duration prior to randomisation

Section 4.4.5 – Information added regarding starting research radiotherapy treatment

Section 4.4.6 – Information updated on concomitant medications

Section 5 – Clarification regarding randomisation allocation added to reflect the addition of the new RT research arm

Section 6.1.8 – Information added to describe the administration of research radiotherapy

Section 6.2.1 – Clarification added regarding the measurement of serum creatinine levels prior to the administration of zoledronic acid

Section 6.2.3 – Clarification regarding the completion of recruitment to the celecoxib containing arms

Section 6.25 – Information added regarding the administration of research radiotherapy treatment

Section 6.6 – Clarification incorporated to describe the administration of standard-of-care radiotherapy

Section 7.1.4 – Information added regarding data collection and non-administration of standard radiotherapy

Section 7.2 – Section updated to include new treatment specific CRFs and timing of CRFs

Section 8.1 – Clarification added for the criteria to stop treatment for patients randomised to arm G

Section 8.2 – Section expanded to include additional details on study patient transfer to different centres

Section 8.3 – Additional sentence inserted to reinforce the importance of compliance with follow-up assessments

Section 9.1 – Additional paragraph inserted to clarify the method of randomisation and allocation distribution in the light of the introduction of the new RT arm

Section 9.4 – Wording updated to clarify the assessment of safety data

Section 9.5.4 – Wording updated concerning the end of randomisations to arm G

Section 9.6 to 9.6.4 – Section added describing sample size issues and trial stages for arm H

Section 9.8 – Clarification on intermediate stopping guidelines

Section 9.9 – Clarification on the outline analysis plan

Section 11 – Information on safety reporting updated to reflect the addition of the research RT comparison arm

Section 11 – Clarification added regarding arm A safety reporting timelines

Section 12.1 – Clarification added regarding the Principal Investigator's responsibilities

Section 14 – Indemnity section updated to reflect current MRC policy

Section 16 – Clarification regarding TMG membership

Section 17.3 – Section on Bone Mineral Density sub-study removed

Section 19 – Information regarding amendments to protocol appendices moved to the separate appendices document

Section 20 – References updated

21.1.11 Amendments Made To Protocol Version 9.0 (Oct-2012)

Throughout protocol – numbering, sections headings, tables, figures and bibliographical references have been updated in some sections to accommodate the completion of recruitment to original research arms B, C and E.

Throughout protocol – Tenses have been changed to reflect activities that were in the future and which have now been passed.

Section 1 – Figure added and clarifications added to each figure

Section 2 – Previous reference 8 removed

Section 4 – Clarification of acceptable alternatives to bone scans

Section 6.2.5 – Correction of an error defining the PTV: the wording has been reordered

Table 4 – Dose-volume objectives corrected: order swapped

Table 5- Correction CRFs names

Section 17.3.2 – Clarification that DNA may be extracted

21.1.12 Amendments Made To Protocol Version 10.0 (Apr-2013)

Throughout protocol – numbering, sections headings, tables, figures and bibliographical references have been updated in some sections

Throughout protocol – typos have been corrected

Section 4 –Clarification of exclusion criteria V (now V and VI)

Section 6 – Timing of orchidectomy prior to randomisation extended to 12 weeks

Section 6 – Clarification of hypokalaemia, blood pressure and fluid retention management

Section 9 – Statistical considerations amended in light of the recruitment extension for the abiraterone comparison

Section 14 - Section updated to reflect the changes in the structure of the MRC CTU (now MRC CTU at UCL) and indemnity arrangements

21.1.13 Amendments Made To Protocol Version 11.0 (Sep-2013)

Throughout protocol – numbering, sections headings, tables, figures and bibliographical references have been updated in some sections

Throughout protocol – typos have been corrected

Co-investigators list updated to reflect the addition of the "enzalutamide + abiraterone comparison" lead

Section 1.2 – Enzalutamide added as trial treatment

Section 1.2 – Protocol version 12.0 added to the list of amendments

Section 2.10 – Rationale for the combination of enzalutamide and abiraterone

Section 4.2 – Eligibility criteria amended to reflect the addition of enzalutamide + abiraterone arm

Section 4.4.2 – Wording clarified

Section 6.8 – Clarification regarding end of trial treatment after starting trial therapy

Section 6.10 – Section added to describe enzalutamide and abiraterone treatment for the new research arm (Arm J)

Section 6.11.4.A – Section added to describe the management of toxicities from trial abiraterone

Section 6.11.4.B - Section added to describe the management of toxicities from trial enzalutamide

Section 9.1.4 – Section added to describe the statistical considerations concerning the introduction of Arm J

Section 9.3 – Principles and assumption for the introduction of Arm J added

Section 9.7 and sub-sections – Sample size issues and trial stages for Arm J

Section 9.9 – Details on interim monitoring and analyses for Arm J added

Section 11.2.1.D – Wording clarified regarding safety reporting requirements for control arm

Section 12.1 – Wording clarified

Section 15 – Details on funding for the "enzalutamide + abiraterone comparison" added

Section 19 - Amendments made to protocol updated

Reference list updated

21.1.14 Amendments Made To Protocol Version 12.0 (Jan-2014)

Throughout protocol – typos have been corrected

Section 4.4.2. Wording clarified

Section 4.3. Wording clarified for eligibility to M1|RT comparison

Section 6.10. Addition of use of dexamethasone post-biochemical progression for Arm J patients

Section 6.11.4.A. Correction of CTCAE version

Section 6.11.4.C. Clarification on enzalutamide dose modification to be in line with current SmPC

Section 9.6. Sample size increase for M1|RT comparison

Section 11. Correction of safety reporting timelines for Arm A patients

Section 17. Addition of saliva samples collection for DNA analysis

Table 4, 5 and 6. Clarification on Case Report Forms and Follow-up schedule

21.1.15 Amendments Made To Protocol Version 13.0 (Feb-2015)

Throughout protocol – typos have been corrected

Throughout protocol – clarification on the new definition of standard-of-care

Table of contents updated to reflect any changes to the protocol

Section 1.1. Wording added throughout section to include reference to survival results from "original comparisons"

Section 2.1.1. Section improved to include reference to survival results from "original research comparisons"

Section 2.1.2. Section improved to include reference to survival results from "original research comparisons".

Section 2.1.3. Additional section added to describe the role of docetaxel for people with M0 or M1 disease

Section 2.9. Clarification on treatment completion and primary results for "original research comparisons"

Section 4.2. Clarification of Exclusion criteria XIII and XVI

Section 4.4.2. Clarification on HT prior to randomisation

Section4.4.3. New section to clarify standard-of-care docetaxel treatment prior to randomisation

Section 4.4.7. Clarification on concomitant medication and contra-indicated concomitant medications

Section 4.5. Clarification provided on tissue block collection

Section 6. Inclusion of docetaxel into the standard-of-care

Section 6.2.3 New section to describe standard-of-care docetaxel administration

Section 6.11. Improvement throughout sections and sub-sections for abiraterone and enzalutamide-related toxicity management

Section 6.12. Section improved throughout to incorporate clearer details on concomitant medications and drug-to-drug interactions

Section 7.1.4. New section to describe data collection for standard-of-care docetaxel

Section 9.7.4. Clarification provided about implications for "enzalutamide+ abiraterone comparison" following change of standard-of-care treatment

Section 11.2.1.D Clarification on SAE notification timelines to reflect change in standard-of-care treatments (addition of docetaxel)

Figure 1. Figure updated to reflect change in standard-of-care

Figure 2. Figure updated to reflect trial history and recruitment over time

Figure 3. Figure updated to reflect changes in standard-of-care and recruiting arms

Table 1. Table updated to remove repetition

Table 13. Table updated to include new CRF to report standard-of-care docetaxel treatment

Table 15. Table updated to include only active trial treatments

21.1.16 Amendments Made To Protocol Version 14.0 (Oct-2015)

Throughout protocol – typos have been corrected

Throughout protocol – clarification on the new definition of standard-of-care

Table of contents updated to reflect any changes to the protocol

Section 1. Wording added throughout section to include reference "metformin comparison"

Section 2. Section updated to include reference "metformin comparison"

Section 4.2. Exclusion criteria review to reflect Arm J closure and instruction of "metformin comparison"

Section 4.3. Clarification of comparison specific eligibility (M1|RT and metformin)

Section 4.5.7. Clarification on concomitant medication and contra-indicated concomitant medications

Section 6. Treatment sections improved throughout

Section 6.11. Section updated to include details on metformin treatment

Section 6.12. Amendment throughout sections and sub-sections for metformin treatment

Section 6.13. Amendment throughout sections and sub-sections for metformin treatment

Section 6.13. Improvement throughout sections and sub-sections for abiraterone and enzalutamide treatment

Section 7.0. Amendment throughout sections and sub-sections to include assessment and procedures specific to "metformin comparison"

Section 9.0. Section updated and streamlined to capture statistical considerations on each comparison

Section 9.0. Details on "metformin comparison" added

Section 11. Safety processes updated and clarified

Section 16.0 Membership to oversight groups updated

Section 11.2.1.D Clarification on SAE notification timelines to reflect change in standard-of-care treatments (addition of docetaxel)

21.1.17 Amendments Made To Protocol Version 15.0 (Mar-2017)

Throughout protocol – re-structure of the treatment-related information for ease of use

Throughout protocol – clarification on the definition of standard-of-care

Throughout protocol – typos have been corrected

Addition of TMG members

Table of contents updated to reflect any changes to the protocol

New section for summary of trial added in table format

Section 1. Revised format for the summary of treatment groups, with the new transdermal oestradiol arm also added

Section 2. Clarification regarding research treatments that have previously reported or completed recruitment, section updated to include the "transdermal oestradiol comparison"

Section 3. New sections added for the "transdermal oestradiol comparison" and future planned biomarker-selected comparisons

Section 4.1.4. Change in definition of adequate renal function

Section 4.3. New section added for the biomarker screening pilot, selection criteria removed for "research RT comparison"

Section 4.4.1. Change in definition of adequate renal function

Section 4.4.2. New section added for the patient selection criteria specific to the "transdermal oestradiol comparison"

Section 4.5. Screening procedure tables and figure added for clarification.

Section 4.5.1. New section added for biomarker screening pilot investigations prior to randomisation.

Section 5.1.1. New section added for the biomarker screening pilot registration.

Section 6. New sections added for the "transdermal oestradiol comparison"

Section 7. Amendment throughout sections and sub-sections to include assessment and procedures specific to "transdermal oestradiol comparison"

Section 7.1.4.B. Section added on cardiovascular outcomes for the "transdermal oestradiol comparison"

Table 18. Table added to clarify follow-up assessments

Section 8. Section updated for "transdermal oestradiol comparison"

Section 9. Section updated for "transdermal oestradiol comparison"

Section 12.1.1.D. Section added on participant feedback from investigations and additional analyses

Section 15. Section updated for "transdermal oestradiol comparison" and biomarker screening pilot

21.1.18 Amendments Made To Protocol Version 16.0 (Oct-2017)

Summary of trial- Table 1: Schedule of Assessments has been added

Abbreviations & Glossary- new terms have been added

Section 1- Table 4: Abiraterone information updated as results of primary analysis published

Section 4.3 - Biomarker timelines redefined, the length of prior hormone therapy has increased to reflect change in turnaround time for testing

Section 4.6 - Biomarker screening information updated

Section 6.2 – Clarification on safety monitoring required for patients receiving trial abiraterone added . Abiraterone overdose information altered for clarity.

Section 6.3.4 - Drug interactions updated to specify that tamoxifen is contraindicated in combination with abiraterone, enzalutamide and transdermal oestradiol.

Section 6.5 – Detail on requirements at site to demonstrate compliance with per-protocol required safety monitoring added

Section 7 – Schedule for assessments updated, removal of table 19

Section 7.1 - Clarification on additional safety monitoring required for patients receiving trial abiraterone added

Section 7.4 - Table 20 QL information removed and added to Table 1: Schedule for Assessments

Section 10.1.1- Central monitoring of consent information added

Section 11 – Re-structured and re-worded for clarity on reporting requirements for safety data captured on the SAE CRF. Explanation provided for exempted events and definitions added. Table 28 and Box 1 updated and Figure 1 added.

Section 11.2- Updated SAE exceptions, SAE flow chart added for clarity

Section 11.3 - Update of investigator assessments and notification checklist for expedited safety reporting

Section 11.4 - Update of wording of CTU responsibilities

Section 17.4 - Sub-study information added to include Disease Volumetric sub-study

21.1.19 Amendments Made To Protocol Version 17.0 (Feb-2017)

Throughout protocol - Typos have been corrected, abbreviations & glossary & table of contents updated

Throughout protocol - Addition of abiraterone as SOC & original comparisons closed to active follow-up

Throughout protocol – Update of and removal of biomarker pilot information now randomisation to the rucaparib comparison is to be activated

Trial administration – Information updated, full contact list linked to website, all comparison chief-investigators added as co-signatories

Summary of trial – Updated, “rucaparib comparison” added and “original comparisons” closed to active follow-up; figure 1 updated with new randomisation schema and S-STAMPEDE Cohort study

Schedule of Assessments updated– Table 1a removed, Tables 1, 2 and 3 added

Lay Summary – Re-drafted, “rucaparib comparison” added

Section 2 – Role of SOC abiraterone added, reported comparisons updated and rationale for comparisons that have completed recruitment removed. Rational for the “rucaparib comparison” added

Section 3.1 – Addition of site and investigator criteria

Section 4 – Complete restructuring of section, addition of biomarker screening and registration information.

Section 4.2 – Additional information about proposed approach to staged informed consent

Section 4.4 – Clarification as to required pre-randomisation screening by comparison

Section 4.5.4 – Detail regarding SOC abiraterone (permitted in metformin comparison only)

Section 4.9.3 – Eligibility to be randomised to the “rucaparib comparison” added

Section 4.10 - Sub-study eligibility criteria clarified; new germline blood sub-study added (PAXgene for S1A and S1M) and stratified – STAMPEDE cohort study added

Section 5 – additional information relating to registration and randomisation to the “rucaparib comparison” added

Section 6.1.4- SOC abiraterone detail added

Section 6.2.7.C – Table 19: Additional assessments required following change of transdermal oestradiol patch or dose added

Section 6.2.9 - Rucaparib treatment specific information added

6.3 – Concomitant medications updated: clarification that spironalactone is contraindicated with abiraterone and rucaparib drug interactions added

Section 7.1 – Table 27: summary of follow-up schedules by participant group added

Section 7.1.5.D – Additional Safety assessment required for participants receiving rucaparib added

Section 7.2.3 – Data collection for SOC abiraterone clarified

Section 7.2.8 & 7.3.2 - Data collection & Follow-up procedures for S-STAMPEDE Cohort participants described

Section 7.3.3 – Clarification added regarding procedures to use linked follow-up information obtained from sources of electronic health data

Table 29 and 28 updated with new CRFs

Section 8.1.4 – Reasons to stop rucaparib

Section 9.8 – addition of statisitcal considerations relating to the “rucaparib comparison”

Section 11 – Stopping of SARs and SUSARs reporting for “original comparisons” closed to active follow up and addition of rucaparib-specific notable events

Section 12- ethical considerations updated with detail relating to data to permit linkage with sources of electronic health data

Section 13 – Data archiving and retention guidance added

Section 16 – Updates of STAMPEDE oversight committees including expanded TMG sub-groups

Section 17.1.1 A- Closure of HE & QL sub-studies to new participants and stopping of data collection for several comparisons; summarised in Table 40.

Section 17.2 – S-STAMPEDE cohort study & additional germline data collection added

Section 17.4 – New sub-study: Using routine data to identify clinical trial outcomes added

Section 18 – New section regarding patient and public involvement in STAMPEDE added

Section 20 - New section regarding data and sample sharing added

21.1.20 Amendments made to Protocol Version 18.0 (Jun-2018)

Throughout protocol: Removal of “rucaparib comparison” information

Throughout protocol: Redraft of biomarker screening pilot study into ancillary studies section

Summary of Trial – Figure 1 updated; removal of registration information for S-STAMPEDE Cohort study

Schedule of Assessments – Removal of registration from figure 1; Table 2 updated to include PSA within 8 weeks of randomisation; Table 3 S-STAMPEDE Schedule of Assessments removed

Abbreviations & Glossary - Terms relating to the “rucaparib comparison” have been deleted

Section 1 – Lay summary “rucaparib comparison” information removed

Section 2 – Rationale for incorporating molecular stratificaion and “rucaparib comparison” removed

Section 3.2 – “Rucaparib comparison” comparison-specific site accreditation removed

Section 4 – Removal of biomarker screening and registration information

Section 4.2 – Removal of staged informed consent process

Section 4.3 – Removal of biomarker screening eligibility information

Section 4.4.3 – Removal of “rucaparib comparison” screening investigations prior to randomisation

Section 4.5 – Removal of prior permitted SOC treatments for “rucaparib comparison”

Section 4.7 – Removal of and clarification to the general inclusion & exclusion criteria of Serum Pottasium & Cardiovascular disease respectively.

Section 4.9.3 – Removal of “rucaparib comparison” specific eligibility criteria

Section 4.10 – Addition of information regarding biomarker pilot screening

Section 4.10.1 – Removal of S-STAMPEDE Cohort sub-study

Section 4.10.2 – Removal of PAXgene sample collection

Section 5 – Removal of information relating to registration and randomisation to the “rucaparib comparison”

Section 6.2.6.B – Addition of additional metformin dose reduction stages

Section 6.2.9 – Removal of “rucaparib comparison” research treatment information

Section 6.3.1 – Removal of “rucaparib comparison” therapeutic interactions information

Section 7 - Table 30 removal of “rucaparib comparison” specific CRFs; table 31 – removal of arm S1M schedule for completion of treatment forms

Section 7.1.5.D – Removal of rucaparib additional safety assessments

Section 7.2.8 – Removal of data collection for S-STAMPEDE Cohort participants

Section 7.3.2 – Removal of follow-up for S-STAMPEDE cohort participants

Section 8.1.1 – Clarification of metformin, abiraterone and enzalutamide use post progression

Section 8.1.4 – Removal of “rucaparib comparison” stopping trial treatment information

Section 9.6.7 – Revised sample size for “metformin comparison”

Section 9.8 – Removal of statistical consideration relating to the “rucaparib comparisons”

Section 10.1.1 – Updated central monitoring of consent process

Section 11 – Removal of rucaparib-sepcific notable events

Section 11.1.1 – Clarification of trial-specific exemptions and notable adverse events

Section 16 – Addition of Genetic Sub-Group

Section 17.1.1.A – Clarification of Qulaity of Life and Health Economics data collection

Section 17.2 – Details of biomarker screening pilot study information moved here

Section 17.2.1 – removal of S-STAMPEDE Cohort study information

22 REFERENCES

1. James ND, Sydes MR, Clarke NW, Mason MD, Dearnaley DP, Spears MR, et al. Addition of docetaxel, zoledronic acid, or both to first-line long-term hormone therapy in prostate cancer (STAMPEDE): survival results from an adaptive, multiarm, multistage, platform randomised controlled trial. Lancet. 2016;387(10024):1163-77.

2. Mason MD, Clarke NW, James ND, Dearnaley DP, Spears MR, W.S.R. A, et al. Adding celecoxib with or without zoledronic acid for hormone-naive prostate cancer: long-term survival results from an adaptive, multi-arm, multi-stage, platform, randomised controlled trial. 2017. In press. DOI: 10.1200/JCO.2016.69.0677.

3. James ND, de Bono JS, Spears MR, Clarke NW, Mason MD, Dearnaley DP, et al. Abiraterone for Prostate Cancer Not Previously Treated with Hormone Therapy. N Engl J Med. 2017;377(4):338-51.

4. Mason MD, Clarke NW, James ND, Dearnaley DP, Spears MR, Ritchie AWS, et al. Adding Celecoxib With or Without Zoledronic Acid for Hormone-Naive Prostate Cancer: Long-Term Survival Results From an Adaptive, Multiarm, Multistage, Platform, Randomized Controlled Trial. J Clin Oncol. 2017;35(14):1530-41.

5. cancerresearchuk. Prostate cancer statistics 2015 [Available from: http://www.cancerresearchuk.org/health-professional/cancer-statistics/statistics-by-cancer-type/prostate-cancer.

6. Cancer Research UK. CancerStats Key Facts: Prostate Cancer. Cancer Research UK. 2011.

7. Sharifi N, Gulley JL, Dahut WL, Sharifi N, Gulley JL, Dahut WL. An update on androgen deprivation therapy for prostate cancer. Endocrine-Related Cancer. 2010;17(4):R305-15.

8. Widmark A, Klepp O, Solberg A, Damber JE, Angelsen A, Fransson P, et al. Endocrine treatment, with or without radiotherapy, in locally advanced prostate cancer (SPCG-7/SFUO-3): an open randomised phase III trial. Lancet. 2009;373(9660):301-8.

9. Warde PR, Mason MD, Sydes MR, Gospodarowicz MK, Swanson GP, Kirkbride P, et al. Intergroup randomized phase III study of androgen deprivation therapy (ADT) plus radiation therapy (RT) in locally advanced prostate cancer (CaP) (NCIC-CTG, SWOG, MRC-UK, INT: T94-0110; NCT00002633). J Clin Oncol. 2010;28(18s Supplement: Proceedings of ASCO 2010):Abstr CRA4504.

10. Warde P, Mason M, Ding K, Kirkbride P, Brundage M, Cowan R, et al. Survival Benefit with Combined Androgen Deprivation and Radiation Therapy in Locally Advanced Prostate Cancer – Results of a Phase III Trial. The Lancet. 2011 - in press.

11. Mason M, Sydes M, Parulekar W, Parmar M, Anderson J, Barber J, et al. Final analysis of intergroup randomized phase III study of androgen deprivation therapy (ADT) + radiation therapy (RT) in locally advanced prostate cancer (CaP) (NCIC-CTG, SWOG, MRC-UK, INT: T94-0110). National Cancer Research Institute (NCRI) Cancer Conference 2012. 2012;2012.

12. Warde P, Mason M, Ding K, Kirkbride P, Brundage M, Cowan R, et al. Combined androgen deprivation therapy and radiation therapy for locally advanced prostate cancer: a randomised, phase 3 trial. The Lancet. 2011;378:2104-11.

13. Nicholas D James MRS, Noel W Clarke, David P Dearnaley, Malcolm D Mason, Christopher C Parker, Alastair W S Ritchie, J. Martin Russell, Francesca Schiavone, Gerhardt Attard, Johann S de Bono, Alison Birtle,Daniel S Engeler,Tony Elliott, David Matheson, Joe O'Sullivan, Delia Pudney, Narayanan

Srihari, Jan Wallace, Jim Barber, Isabel Syndikus, Mahesh K B Parmar, Matthew R Sydes. Failure-free survival and the impact of radiotherapy in patients with newly diagnosed non metastatic prostate cancer: Data from patients in the control arm of the STAMPEDE trial (MRC PR08, CRUK/06/019). JAMA Oncology. 2015.

14. Sweeney CJ, Chen YH, Carducci M, Liu G, Jarrard DF, Eisenberger M, et al. Chemohormonal Therapy in Metastatic Hormone-Sensitive Prostate Cancer. N Engl J Med. 2015;373(8):737-46.

15. Vale CL, Burdett S, Rydzewska LH, Albiges L, Clarke NW, Fisher D, et al. Addition of docetaxel or bisphosphonates to standard of care in men with localised or metastatic, hormone-sensitive prostate cancer: a systematic review and meta-analyses of aggregate data. The lancet oncology. 2016;17(2):243-56.

16. Fizazi K, Tran N, Fein L, Matsubara N, Rodriguez-Antolin A, Alekseev BY, et al. Abiraterone plus Prednisone in Metastatic, Castration-Sensitive Prostate Cancer. N Engl J Med. 2017;377(4):352-60.

17. Robinson D, Van Allen EM, Wu YM, Schultz N, Lonigro RJ, Mosquera JM, et al. Integrative clinical genomics of advanced prostate cancer. Cell. 2015;161(5):1215-28.

18. Grasso CS, Wu YM, Robinson DR, Cao X, Dhanasekaran SM, Khan AP, et al. The mutational landscape of lethal castration-resistant prostate cancer. Nature. 2012;487(7406):239-43.

19. Beltran H, Yelensky R, Frampton GM, Park K, Downing SR, MacDonald TY, et al. Targeted next-generation sequencing of advanced prostate cancer identifies potential therapeutic targets and disease heterogeneity. European urology. 2013;63(5):920-6.

20. Mateo J, Carreira S, Sandhu S, Miranda S, Mossop H, Perez-Lopez R, et al. DNA-Repair Defects and Olaparib in Metastatic Prostate Cancer. New England Journal of Medicine. 2015;373(18):1697-708.

21. Pritchard CC, Mateo J, Walsh MF, De Sarkar N, Abida W, Beltran H, et al. Inherited DNA-Repair Gene Mutations in Men with Metastatic Prostate Cancer. New England Journal of Medicine. 2016;375(5):443-53.

22. Tutt A, Robson M, Garber JE, Domchek SM, Audeh MW, Weitzel JN, et al. Oral poly(ADP-ribose) polymerase inhibitor olaparib in patients with BRCA1 or BRCA2 mutations and advanced breast cancer: a proof-of-concept trial. The Lancet. 2010;376(9737):235-44.

23. Audeh MW, Carmichael J, Penson RT, Friedlander M, Powell B, Bell-McGuinn KM, et al. Oral poly(ADP-ribose) polymerase inhibitor olaparib in patients with BRCA1 or BRCA2 mutations and recurrent ovarian cancer: a proof-of-concept trial. The Lancet.376(9737):245-51.

24. Mirza MR, Monk BJ, Herrstedt J, Oza AM, Mahner S, Redondo A, et al. Niraparib Maintenance Therapy in Platinum-Sensitive, Recurrent Ovarian Cancer. New England Journal of Medicine. 2016;375(22):2154-64.

25. Ledermann J, Oza AM, Lorusso D, Aghajanian C, Oaknin A, Dean A, et al. LBA40_PRARIEL3: A phase 3, randomised, double-blind study of rucaparib vs placebo following response to platinum-based chemotherapy for recurrent ovarian carcinoma (OC). Annals of Oncology. 2017;28(suppl_5):mdx440.034-mdx440.034.

26. James ND, Sydes MR, Clarke NW, Mason MD, Dearnaley DP, Spears MR, et al. Addition of docetaxel, zoledronic acid, or both to first-line long-term hormone therapy in prostate cancer (STAMPEDE): survival results from an adaptive, multiarm, multistage, platform randomised controlled trial. Lancet. 2015.

27. Attard G, Richards J, de Bono JS, Attard G, Richards J, de Bono JS. New strategies in metastatic prostate cancer: targeting the androgen receptor signaling pathway. Clinical Cancer Research. 2011;17(7):1649-57.

28. Fizazi K, Scher HI, Molina A, Logothetis CJ, Chi KN, Jones RJ, et al. Abiraterone acetate for treatment of metastatic castration-resistant prostate cancer: final overall survival analysis of the COU-AA-301 randomised, double-blind, placebo-controlled phase 3 study. The lancet oncology. 2012;13(10):983-92.

29. Rathkopf DE, Smith MR, de Bono JS, Logothetis CJ, Shore ND, de Souza P, et al. Updated interim efficacy analysis and long-term safety of abiraterone acetate in metastatic castration-resistant prostate cancer patients without prior chemotherapy (COU-AA-302). European urology. 2014;66(5):815-25.

30. Flanigan RC, Salmon SE, Blumenstein BA, Bearman SI, Roy V, McGrath PC, et al. Nephrectomy followed by interferon alfa-2b compared with interferon alfa-2b alone for metastatic renal-cell cancer. N Engl J Med. 2001;345(23):1655-9.

31. Mickisch GH, Garin A, van Poppel H, de Prijck L, Sylvester R. Radical nephrectomy plus interferon-alfa-based immunotherapy compared with interferon alfa alone in metastatic renal-cell carcinoma: a randomised trial. Lancet. 2001;358(9286):966-70.

32. Attard G, Cooper CS, de Bono JS. Steroid hormone receptors in prostate cancer: a hard habit to break? Cancer cell. 2009;16(6):458-62.

33. Chen G, Wang X, Zhang S, Lu Y, Sun Y, Zhang J, et al. Androgen receptor mutants detected in recurrent prostate cancer exhibit diverse functional characteristics. The Prostate. 2005;63(4):395-406.

34. Taplin ME, Bubley GJ, Ko YJ, Small EJ, Upton M, Rajeshkumar B, et al. Selection for androgen receptor mutations in prostate cancers treated with androgen antagonist. Cancer Res. 1999;59(11):2511-5.

35. Veldscholte J, Ris-Stalpers C, Kuiper GG, Jenster G, Berrevoets C, Claassen E, et al. A mutation in the ligand binding domain of the androgen receptor of human LNCaP cells affects steroid binding characteristics and response to anti-androgens. Biochemical and biophysical research communications. 1990;173(2):534-40.

36. Zhao XY, Malloy PJ, Krishnan AV, Swami S, Navone NM, Peehl DM, et al. Glucocorticoids can promote androgen-independent growth of prostate cancer cells through a mutated androgen receptor. Nature medicine. 2000;6(6):703-6.

37. Attard G, Reid AH, Auchus RJ, Hughes BA, Cassidy AM, Thompson E, et al. Clinical and biochemical consequences of CYP17A1 inhibition with abiraterone given with and without exogenous glucocorticoids in castrate men with advanced prostate cancer. The Journal of clinical endocrinology and metabolism. 2012;97(2):507-16.

38. Richards J, Lim AC, Hay CW, Taylor AE, Wingate A, Nowakowska K, et al. Interactions of abiraterone, eplerenone, and prednisolone with wild-type and mutant androgen receptor: a rationale for increasing abiraterone exposure or combining with MDV3100. Cancer Res. 2012;72(9):2176-82.

39. Scher HI, Fizazi K, Saad F, Taplin ME, Sternberg CN, Miller K, et al. Increased survival with enzalutamide in prostate cancer after chemotherapy. N Engl J Med. 2012;367(13):1187-97.

40. Tombal B, Borre M, Rathenborg P, Werbrouck P, Heidenreich A, Iversen P, et al. Enzalutamide monotherapy: Phase II study results in patients with hormone-naive prostate cancer. Journal of Clinical Oncology. 2013;31(Supplement 6):Abstract 18.

41. Efstathiou E, Titus MA, Tsavachidou A, Hoang A, Karlou M, Wen S, et al. MDV3100 effects on androgen receptor (AR) signaling and bone marrow testosterone concentration modulation: A preliminary report. . 2011 ASCO Annual Meeting, 2011 J Clin Oncol 2011.

42. Locke JA, Guns ES, Lubik AA, Adomat HH, Hendy SC, Wood CA, et al. Androgen levels increase by intratumoral de novo steroidogenesis during progression of castration-resistant prostate cancer. Cancer Res. 2008;68(15):6407-15.

43. Tran C, Ouk S, Clegg NJ, Chen Y, Watson PA, Arora V, et al. Development of a second-generation antiandrogen for treatment of advanced prostate cancer. Science. 2009;324(5928):787-90.

44. Scher HI, Beer TM, Higano CS, Anand A, Taplin ME, Efstathiou E, et al. Antitumour activity of MDV3100 in castration-resistant prostate cancer: a phase 1-2 study. Lancet. 2010;375(9724):1437-46.

45. Smith MR, Finkelstein JS, McGovern FJ, Zietman AL, Fallon MA, Schoenfeld DA, et al. Changes in body composition during androgen deprivation therapy for prostate cancer. The Journal of clinical endocrinology and metabolism. 2002;87(2):599-603.

46. Eriksson A, Attvall S, Bonnier M, Eriksson JW, Rosander B, Karlsson FA. Short-term effects of metformin in type 2 diabetes. Diabetes, obesity & metabolism. 2007;9(4):483-9.

47. Bailey CJ. Treating insulin resistance in type 2 diabetes with metformin and thiazolidinediones. Diabetes, obesity & metabolism. 2005;7(6):675-91.

48. Ohira M, Miyashita Y, Ebisuno M, Saiki A, Endo K, Koide N, et al. Effect of metformin on serum lipoprotein lipase mass levels and LDL particle size in type 2 diabetes mellitus patients. Diabetes research and clinical practice. 2007;78(1):34-41.

49. Wulffele MG, Kooy A, de Zeeuw D, Stehouwer CD, Gansevoort RT. The effect of metformin on blood pressure, plasma cholesterol and triglycerides in type 2 diabetes mellitus: a systematic review. Journal of internal medicine. 2004;256(1):1-14.

50. Lubik AA, Gunter JH, Hendy SC, Locke JA, Adomat HH, Thompson V, et al. Insulin increases de novo steroidogenesis in prostate cancer cells. Cancer Res. 2011;71(17):5754-64.

51. Venkateswaran V, Haddad AQ, Fleshner NE, Fan R, Sugar LM, Nam R, et al. Association of diet-induced hyperinsulinemia with accelerated growth of prostate cancer (LNCaP) xenografts. J Natl Cancer Inst. 2007;99(23):1793-800.

52. Noto H, Goto A, Tsujimoto T, Noda M. Cancer risk in diabetic patients treated with metformin: a systematic review and meta-analysis. PloS one. 2012;7(3):e33411.

53. Rothermundt C, Hayoz S, Templeton AJ, Winterhalder R, Strebel RT, Bartschi D, et al. Metformin in chemotherapy-naive castration-resistant prostate cancer: a multicenter phase 2 trial (SAKK 08/09). European urology. 2014;66(3):468-74.

54. Langley RE, Cafferty FH, Alhasso AA, Rosen SD, Sundaram SK, Freeman SC, et al. Cardiovascular outcomes in patients with locally advanced and metastatic prostate cancer treated with luteinising-hormone-releasing-hormone agonists or transdermal oestrogen: the randomised, phase 2 MRC PATCH trial (PR09). The lancet oncology. 2013;14(4):306-16.

55. Byar DP. Proceedings: The Veterans Administration Cooperative Urological Research Group's studies of cancer of the prostate. Cancer. 1973;32(5):1126-30.

56. Hedlund PO, Henriksson P. Parenteral estrogen versus total androgen ablation in the treatment of advanced prostate carcinoma: effects on overall survival and cardiovascular mortality. The Scandinavian Prostatic Cancer Group (SPCG)-5 Trial Study. Urology. 2000;55(3):328-33.

57. Gilbert DC, Duong T, Kynaston HG, Alhasso AA, Cafferty FH, Rosen SD, et al. Quality-of-life outcomes from the Prostate Adenocarcinoma: TransCutaneous Hormones (PATCH) trial evaluating luteinising hormone-releasing hormone agonists versus transdermal oestradiol for androgen suppression in advanced prostate cancer. BJU international. 2016.

58. Smith DC, Redman BG, Flaherty LE, Li L, Strawderman M, Pienta KJ. A phase II trial of oral diethylstilbesterol as a second-line hormonal agent in advanced prostate cancer. Urology. 1998;52(2):257-60.

59. James ND, Spears MR, Clarke NW, Dearnaley DP, De Bono JS, Gale J, et al. Survival with Newly Diagnosed Metastatic Prostate Cancer in the "Docetaxel Era": Data from 917 Patients in the Control Arm of the STAMPEDE Trial (MRC PR08, CRUK/06/019). European urology. 2015;67(6):1028-38.

60. Patrikidou A, Loriot Y, Eymard JC, Albiges L, Massard C, Ileana E, et al. Who dies from prostate cancer? Prostate Cancer Prostatic Dis. 2014;17(4):348-52.

61. Kumar A, Coleman I, Morrissey C, Zhang X, True LD, Gulati R, et al. Substantial interindividual and limited intraindividual genomic diversity among tumors from men with metastatic prostate cancer. Nature medicine. 2016;22(4):369-78.

62. Abida W, Walsh M, Armenia J, Vijai J, Gopalan A, Brennan R, et al. Abstract LB-070: Next generation sequencing of prostate cancer reveals germline and somatic alterations detected at diagnosis and at metastasis that may impact clinical decision making2016. LB-070 p.

63. Lupo B, Trusolino L. Inhibition of poly(ADP-ribosyl)ation in cancer: old and new paradigms revisited. Biochimica et biophysica acta. 2014;1846(1):201-15.

64. Polo SE, Jackson SP. Dynamics of DNA damage response proteins at DNA breaks: a focus on protein modifications. Genes & development. 2011;25(5):409-33.

65. Barbieri CE, Baca SC, Lawrence MS, Demichelis F, Blattner M, Theurillat JP, et al. Exome sequencing identifies recurrent SPOP, FOXA1 and MED12 mutations in prostate cancer. Nature genetics. 2012;44(6):685-9.

66. Pritchard CC, Morrissey C, Kumar A, Zhang X, Smith C, Coleman I, et al. Complex MSH2 and MSH6 mutations in hypermutated microsatellite unstable advanced prostate cancer. Nature communications. 2014;5:4988.

67. Marcos EC, Romero Laorden N, Rodriguez JMP, del Pozo A, Sáez MI, Colmenero AM, et al. LBA32PROREPAIR-B: A prospective cohort study of DNA repair defects in metastatic castration resistant prostate cancer (mCRPC). Annals of Oncology. 2017;28(suppl_5):mdx440.025-mdx440.025.

68. Shrivastav M, De Haro LP, Nickoloff JA. Regulation of DNA double-strand break repair pathway choice. Cell research. 2008;18(1):134-47.

69. Farmer H, McCabe N, Lord CJ, Tutt AN, Johnson DA, Richardson TB, et al. Targeting the DNA repair defect in BRCA mutant cells as a therapeutic strategy. Nature. 2005;434(7035):917-21.

70. Bryant HE, Schultz N, Thomas HD, Parker KM, Flower D, Lopez E, et al. Specific killing of BRCA2-deficient tumours with inhibitors of poly(ADP-ribose) polymerase. Nature. 2005;434(7035):913-7.

71. Annunziata CM, O'Shaughnessy J. Poly (ADP-ribose) polymerase as a novel therapeutic target in cancer. Clinical cancer research : an official journal of the American Association for Cancer Research. 2010;16(18):4517-26.

72. Sandhu SK, Schelman WR, Wilding G, Moreno V, Baird RD, Miranda S, et al. The poly(ADP-ribose) polymerase inhibitor niraparib (MK4827) in BRCA mutation carriers and patients with sporadic cancer: a phase 1 dose-escalation trial. The lancet oncology. 2013;14(9):882-92.

73. Robson M, Im S-A, Senkus E, Xu B, Domchek SM, Masuda N, et al. Olaparib for Metastatic Breast Cancer in Patients with a Germline BRCA Mutation. New England Journal of Medicine. 2017;377(6):523-33.

74. Coleman RL, Oza AM, Lorusso D, Aghajanian C, Oaknin A, Dean A, et al. Rucaparib maintenance treatment for recurrent ovarian carcinoma after response to platinum therapy (ARIEL3): a randomised, double-blind, placebo-controlled, phase 3 trial. The Lancet.390(10106):1949-61.

75. Foedermayr M, Sebesta M, Rudas M, Berghoff AS, Promberger R, Preusser M, et al. BRCA-1 methylation and TP53 mutation in triple-negative breast cancer patients without pathological complete response to taxane-based neoadjuvant chemotherapy. Cancer chemotherapy and pharmacology. 2014;73(4):771-8.

76. Castro E, Goh C, Leongamornlert D, Saunders E, Tymrakiewicz M, Dadaev T, et al. Effect of BRCA Mutations on Metastatic Relapse and Cause-specific Survival After Radical Treatment for Localised Prostate Cancer. European urology. 2015;68(2):186-93.

77. Hsueh AJW, Peck Jr EJ, Clark JH. Progesterone antagonism of the oestrogen receptor and oestrogen-induced uterine growth. Nature. 1975;254:337.

78. de Bono JS, Logothetis CJ, Molina A, Fizazi K, North S, Chu L, et al. Abiraterone and increased survival in metastatic prostate cancer. New England Journal of Medicine. 2011;364(21):1995-2005.

79. Ryan CJ, Smith MR, de Bono JS, Molina A, Logothetis CJ, de Souza P, et al. Abiraterone in metastatic prostate cancer without previous chemotherapy. N Engl J Med. 2013;368(2):138-48.

80. Janssen Research & Development L. ZYTIGA® (abiraterone acetate) Investigator Brochure. 13 ed2017.

81. Lipska KJ, Bailey CJ, Inzucchi SE. Use of metformin in the setting of mild-to-moderate renal insufficiency. Diabetes care. 2011;34(6):1431-7.

82. Radiologists TRCo. Standards for intravascular contrast administration to adult patients [3rd:[Available from: https://www.rcr.ac.uk/sites/default/files/Intravasc_contrast_web.pdf.

83. Salpeter SR, Greyber E, Pasternak GA, Salpeter EE. Risk of fatal and nonfatal lactic acidosis with metformin use in type 2 diabetes mellitus: systematic review and meta-analysis. Archives of internal medicine. 2003;163(21):2594-602.

84. Viani GA, Bernardes da Silva LG, Stefano EJ. Prevention of gynecomastia and breast pain caused by androgen deprivation therapy in prostate cancer: tamoxifen or radiotherapy? International journal of radiation oncology, biology, physics. 2012;83(4):e519-24.

85. Royston P, Parmar MKB, Qian W. Novel designs for multi-arm clinical trials with survival outcomes with an application in ovarian cancer. Statistics in Medicine. 2003;22(14):2239-56.

86. Royston P, Barthel FMS, Parmar MKB, Choodari-Oskooei B, Isham V. Designs for clinical trials with time-to-event outcomes based on stopping guidelines for lack of benefit. Trials. 2011;12(1):81.

87. Royston P. nstage: MAMS trial sample size calculator. MRC Clinical Trials Unit, London2009.

88. Mason MD, Parulekar WR, Sydes MR, Brundage M, Kirkbride P, Gospodarowicz M, et al. Final Report of the Intergroup Randomized Study of Combined Androgen-Deprivation Therapy Plus Radiotherapy Versus Androgen-Deprivation Therapy Alone in Locally Advanced Prostate Cancer. J Clin Oncol. 2015;33(19):2143-50.

89. James ND, Sydes MR, Mason MD, Clarke NW, Anderson J, Dearnaley DP, et al. Celecoxib plus hormone therapy versus hormone therapy alone for hormone-sensitive prostate cancer: first results from the STAMPEDE multiarm, multistage, randomised controlled trial. The lancet oncology. 2012;13(5):549-58.

90. Haybittle JL. Repeated assessment of results in clinical trials of cancer treatment. The British journal of radiology. 1971;44(526):793-7.

91. Peto R, Pike MC, Armitage P, Breslow NE, Cox DR, Howard SV, et al. Design and analysis of randomized clinical trials requiring prolonged observation of each patient. I. Introduction and design. British journal of cancer. 1976;34(6):585-612.
