# Supplementary material for: Abiraterone acetate plus prednisolone for metastatic patients starting hormone therapy: 5‐year follow‐up results from the STAMPEDE randomised trial (NCT00268476)
Source: Int J Cancer. 2022 May 16;151(3):422–34. doi: 10.1002/ijc.34018 (PMC9321995; doi:10.1002/ijc.34018)
Supplement: Supplementary file 2 — Appendix S2Supporting Information. [file IJC-151-422-s001.zip › IJC_34018_STAMPEDE_Protocol_v7.0.pdf]

CANCER RESEARCH UK

MRC Clinical Trials Unit

NCRN National Cancer Research Network

NCRI National Cancer Research Institute

STAMPEDE

Systemic Therapy in Advancing or Metastatic Prostate Cancer: Evaluation of Drug Efficacy

A multi-stage multi-arm randomised controlled trial

Developed with the NCRI Prostate Clinical Studies Group

MRC PR08

ISRCTN number: ISRCTN78818544
EUDRACT number: 2004-000193-31
CTA number: 00316/0026/001-0001
NCT number: NCT00268476

Part of the National Cancer Research Network Portfolio

PROTOCOL VERSION 7.0

14 Jun 2011

Authorised by:

Name	**Matthew Sydes**	Signature	M Sydes
Role	**Trial Statistician**	Date	**14 Jun 2011**
Name	**Professor Nicholas D James**	Signature	Nicholas James
Role	**Chief Investigator**	Date	**14 Jun 2011**

GENERAL INFORMATION

This document describes a trial coordinated by the Medical Research Council (MRC) Clinical Trials Unit (CTU) and provides information about procedures for entering patients into it. The protocol should not be used as an aide-memoire or guide for the treatment of other patients; every care was taken in its drafting, but corrections or amendments may be necessary. These will be circulated to the known investigators in the trial, but centres entering patients for the first time are advised to contact the Cancer Division, MRC CTU, London to confirm they have the most up to date version. Clinical problems relating to this study should be referred to the Chief Investigator.

Sponsor

Medical Research Council, 2nd Floor, David Phillips Building, Polaris House, North Star Avenue, Swindon, SN2 1FL, UK

Funding

Clinical Trials Advisory Awards Committee (on behalf of Cancer Research UK, Medical Research Council, and other charities) together with educational grants from Novartis and Aventis.

Compliance

This trial will adhere to the principles outlined in the International Conference on Harmonization (ICH) Good Clinical Practice (GCP) guidelines. It will be conducted in compliance with the protocol, MRC GCP, Data Protection Act (DPA number: 5886415) and other regulatory requirements, as appropriate.

Authorisation

The following persons are authorised to sign the final protocol and protocol amendments for the sponsor: Professor N James (Chief Investigator) and Matthew Sydes (Trial Statistician).

RANDOMISATIONS

To randomise call MRC CTU, Monday to Friday 0900-1700 excluding public holidays or dates when notice has been given by the CTU.

Telephone: + 44(0) 20 7670 4777

SAE REPORTING

Fax to 020 7670 4744 within 24 hours of becoming aware of the event

TRIAL ADMINISTRATION

Chief Investigator

Prof Nicholas James
CRUK Institute for Cancer Studies
University of Birmingham
Edgbaston, Birmingham
B15 2TT
Tel: 0121 414 4097/7584
Fax: 0121 414 3263
Email: n.d.James@bham.ac.uk
d.meredith@bham.ac.uk
(secretary)

Trial Surgeon

Mr Alastair Ritchie
Urological Surgeon
Contact via MRC CTU

Co-Investigators

Mr John Anderson, Urologist, Sheffield
Mr Noel Clarke, Urologist, Manchester
Prof David Dearnaley, Oncologist, Royal Marsden
Prof Malcolm Mason, Oncologist, Cardiff
Prof Johann de Bono, Oncologist, Royal Marsden
Dr Martin Russell, Oncologist, Glasgow

Patient Representatives

John Dwyer, Stockport, Patient

Health Economics

Prof Mark Sculpher, York

Molecular Genetics

Prof John Masters, Pathologist, London

For full details of all trial contacts please see Appendix L

Coordinating Centre

MRC Clinical Trials Unit
Cancer Division
Aviation House
Kingsway
London
WC2B 6NH

Tel: 020 7670 4700
Fax: 020 7670 4818
Email: stampede@ctu.mrc.ac.uk

MRC Clinical Trials Unit Staff

Trial Manager:	Tom Fairfield	020 7670 4831	stampede@ctu.mrc.ac.uk
Data Manager:	Charlene Green	020 7670 4882	stampede@ctu.mrc.ac.uk
Data Manager:	Sara Peres	020 7670 4794	stampede@ctu.mrc.ac.uk
Senior Trial Statistician:	Matthew Sydes	020 7670 4798	ms@ctu.mrc.ac.uk
Statistician	Gordana Jovic	020 7670 4647	gj@ctu.mrc.ac.uk
Director of MRC CTU:	Max Parmar	020 7670 4729	mp@ctu.mrc.ac.uk

For general queries, supply of trial materials and collection of data please contact the STAMPEDE Trial Manager

Clinical queries during office hours should be directed to the Chief Investigator, Professor Nick James or the Trial Physician (see above). Out of hours, please call Queen Elizabeth Hospital switchboard on (0121) 472 1311 and ask to bleep Professor James.

CONTENTS

1 Summary 8
1.1 LAY SUMMARY 8
1.2 ABSTRACT AND SUMMARY OF TRIAL DESIGN 9
1.3 TRIAL DOCUMENTATION 11

2 Background 12
2.1 INTRODUCTION AND RATIONALE 12
2.2 BISPHOSPHONATES 12
2.3 CHEMOTHERAPY 13
2.4 CYCLOOXYGENASE-2 INHIBITORS 14
2.5 TREATMENT COMBINATIONS 15

3 Selection of Institutions and Investigators 17

4 Selection of Patients 18
4.1 PATIENT INCLUSION CRITERIA 18
4.2 PATIENT EXCLUSION CRITERIA 19
4.3 SCREENING PROCEDURES 19

5 Randomisation and Enrolment 22
5.1 CO-ENROLMENT GUIDELINES 22

6 Treatment of Patients 23
6.1 TRIAL TREATMENT 23
6.2 ADMINISTRATION AND DOSE MODIFICATIONS 25
6.3 TRIAL PRODUCTS 26
6.4 MEASURES OF COMPLIANCE/ADHERENCE 26
6.5 TREATMENT DATA COLLECTION 27
6.6 NON-TRIAL TREATMENT 27

7 Assessments and Procedures 28
7.1 FLOW CHART/SCHEDULE FOR FOLLOW-UP 28
7.2 FOLLOW-UP
7.3 TRIAL CLOSURE 29

8 Stopping of Treatment or Follow up 32
8.1 STOPPING TRIAL INTERVENTIONS 32
8.2 PATIENT TRANSFERS 32
8.3 WITHDRAWAL FROM THE TRIAL COMPLETELY 32

9 Statistical considerations 33
9.1 METHOD OF RANDOMISATION 33
9.2 OUTCOME MEASURES 33
9.3 SAMPLE SIZE 33
9.4 INTERIM MONITORING AND ANALYSES 37
9.5 OUTLINE ANALYSIS PLAN 37

10 Monitoring & Quality Assurance 39
10.1 MONITORING AT MRC CTU 39
10.2 DIRECT ACCESS TO DATA 39
10.3 VISITS TO INVESTIGATOR SITES 39
10.4 CONFIDENTIALITY 39

11 Safety Reporting 40
11.1 DEFINITIONS
11.2 INSTITUTION/INVESTIGATOR RESPONSIBILITIES 41
11.3 MRC CTU RESPONSIBILITIES 43

12 Ethical considerations and approval 44
12.1 ETHICAL CONSIDERATIONS 44
12.2 ETHICAL APPROVAL 45

13 Regulatory Approval 46

14 Indemnity 47

15 Finance 48

16 Trial Committees 49
16.1 TRIAL MANAGEMENT GROUP (TMG) 49
16.2 TRIAL STEERING COMMITTEE (TSC) 49
16.3 INDEPENDENT DATA MONITORING COMMITTEE (IDMC) 49

17 Ancillary studies 51
17.1 QUALITY OF LIFE 51
17.2 HEALTH ECONOMICS 51
17.3 TRANSLATIONAL SUB-STUDIES 52

18 Publication 53

19 Protocol Amendments 54
19.1 PROTOCOL
19.2 APPENDICES

20 References 63

LIST OF FIGURES

Figure 1a: Arms of the STAMPEDE Trial from the start of the trial to April 2011
Figure 1b: Arms of the STAMPEDE Trial from April 2011 onwards
Figure 2: Summary of timing of trial documentation ahead of accreditation
Figure 3: Summary of timing of case report forms upon randomisation
Figure 3b: Data required on forms
Figure 4: Detailed schedule for completion of forms
Figure 5: Progress of STAMPEDE through the trial stages
Figure 6: Diagram of relationships between trial committees

LIST OF TABLES

Table 1: Outcome Measures
Table 2: Hazard Ratio assumptions under null and alternative hypothesis
Table 3: Guidelines for stopping accrual to the ith research arm
Table 4: Terms and definitions for adverse events
Table 5: Adverse events; some inclusions and exclusions

ABBREVIATIONS AND GLOSSARY

**ACE**	Angiotensin-Converting Enzyme
**AS**	Androgen suppression
bid	Twice a day *(bis in die)*
**BP**	Blood pressure
**BSA**	Body surface area
**CERES**	Consumers for Ethics in Research
**CF**	Consent Form
**CI**	Chief Investigator
**CI**	Confidence interval
**COSTART**	Coding Symbols for a Thesaurus of Adverse Reaction Terms
**Cox-2**	Cyclooxygenase-2
**CRF**	Case Report Form
**CRUK**	Cancer Research UK
**CT**	Computerised tomography
**CTA**	Clinical Trials Authorisation
**CTAAC**	Clinical Trials Advisory and Awards Committee
**CTC**	Common Toxicity Criteria
**CTU**	Clinical Trials Unit
**CTV**	Clinical Tumour Volume
**CXR**	Chest X-ray
**DDX**	Doctors and Dentists Exemption
**DNA**	Deoxyribonucleic Acid
**DPA**	Data Protection Act
**ERC**	Endpoint Review Committee
**ICH**	International Conference on Harmonization
**ECG**	Electro cardiogram
**FBC**	Full Blood Count
**FFS**	Failure-Free Survival
**GCP**	Good Clinical Practice
**GP**	General Practitioner
**GRO**	General Register Office
**HE**	Health Economics
**hr**	Hour
**HR**	Hazard Ratio
**HRPC**	Hormone Refractory Prostate Cancer
**HT**	Hormone Therapy
**IDMC**	Independent Data Monitoring Committee
**IM**	Intramuscular
**ISRCTN**	International Standard Randomised Controlled Trial Number
**IU**	International Units
**IV**	Intravenous
**LD**	Longest diameter
**LFTs**	Liver Function Tests
**LHRH**	Luteinising Hormone Releasing Hormone
**LREC**	Local Research Ethics Committee
**MHRA**	Medicine and Healthcare Products Regulatory Agency
**min**	Minutes
**MRC**	Medical Research Council
**MREC**	Multi-Centre Research Ethics Committee
**MRI**	Magnetic resonance imaging
**NCI**	National Cancer Institute (USA)
**NCRN**	National Cancer Research Network
**NHS**	National Health Service
**NSAID**	Non-Steroidal Anti-inflammatory Drugs
**ONS**	Office for National Statistics

**OS**	Overall Survival
**PI**	Principal Investigator
**PIS**	Patient Information Sheet
po	*per orum* (orally)
**PSA**	Prostate Specific Antigen
**PTV**	Planned Tumour Volume
**QALY**	Quality-adjusted Life Years
qds	*quater die sumendus* (4 times each day)
**QL**	Quality of Life
**R&D**	Research and Development
**RECIST**	Response Evaluation Criteria In Solid Tumours
**SAE**	Serious Adverse Event
sc	*Sub-cutaneous* (under skin)
**SNP**	Single Nucleotide Polymorphism
**SSA**	Site Specific Assessment
**STAMPEDE**	Systemic Therapy in Advancing and Metastatic Prostate Cancer: Evaluation of Drug Efficacy
**SUSAR**	Suspected Unexpected Serious Adverse Reactions
**TMG**	Trial Management Group
**TURP**	Trans-Urethral Resection of Prostate
**TSC**	Trial Steering Committee
**ULN**	Upper Limit of Normal
**U+E**	Urea and Electrolytes
**WHO**	World Health Organisation

1 SUMMARY

1.1 LAY SUMMARY

Prostate cancers depend upon the male hormone testosterone for their growth. Lowering testosterone levels (either by removing all or part of both testes, or by giving anti-hormone injections) slows the growth of prostate cancers. This type of treatment is called hormone treatment and is often used when prostate cancers have spread outside the prostate gland. Although hormone treatment is usually successful at stopping the cancer growing for a period of time, the cancer will begin to grow again in most men.

A number of newer treatments have recently become available and have shown initial promise against prostate cancer. Newer treatments are usually used in prostate cancer when hormone treatment is no longer effective and the cancer has started to grow again. The aim of this trial, which is called STAMPEDE, is to assess three of these newer treatments, given earlier in the course of the disease in combination with hormone treatment.

The three new treatments assessed from the commencement of the trial are:

1. Zoledronic acid: Prostate cancer cells can spread to bones and weaken them. Zoledronic acid is a drug that reduces bone destruction and hardens bones. This may make them more resistant to attack by cancer cells.

2. Docetaxel: A drug that stops cells replicating that is currently being used to treat lung, breast and ovarian cancer.

3. Celecoxib: An aspirin-like drug that is used to treat arthritis. It slows down the growth of cancer cells in the laboratory. We wish to see if it has the same effect on cancer cells in patients.

STAMPEDE will look at the effect of combining one or two of the new treatments described above with hormone treatment. A computer program will be used to allocate which treatment the patient receives, using a chance process. Twice as many patients will be allocated to the control arm of hormone treatment only, compared with each of the other research arms. The trial will look at the effects of the combined treatments on quality of life and find out whether the new treatment combinations increase the time when the cancer is not growing and result in patients living longer. The study will also look at which treatment provides the greater value for money for the health service. Approximately 3,300 patients will participate in the trial and it will take between 6-10 years to complete.

1.2 ABSTRACT AND SUMMARY OF TRIAL DESIGN

STAMPEDE is a multi-centre, randomised controlled trial for patients with locally advanced or metastatic prostate cancer who are about to commence hormone therapy (HT). Patients can have either newly diagnosed disease, or have been previously treated with radical radiotherapy or surgery but now have a rising prostate specific antigen (PSA) (further details on eligibility see section 4). The trial will assess the effects of adding different agents, both as single agents and in combinations, to hormone therapy. The investigational agents are (i) a bisphosphonate, zoledronic acid, (ii) a cytotoxic chemotherapeutic agent, docetaxel and (iii) a cyclooxygenase (Cox-2) inhibitor, celecoxib. The trial has multiple arms; the control arm of the trial is HT only, achieved through the use of luteinising hormone releasing hormone (LHRH) analogues or LHRH antagonists, bicalutamide alone (for M0 patients only) or bilateral orchidectomy according to local practice. The other trial arms are summarised in Figure 1.

Figure 1a - Arms of the STAMPEDE Trial from the start of the trial to April 2011

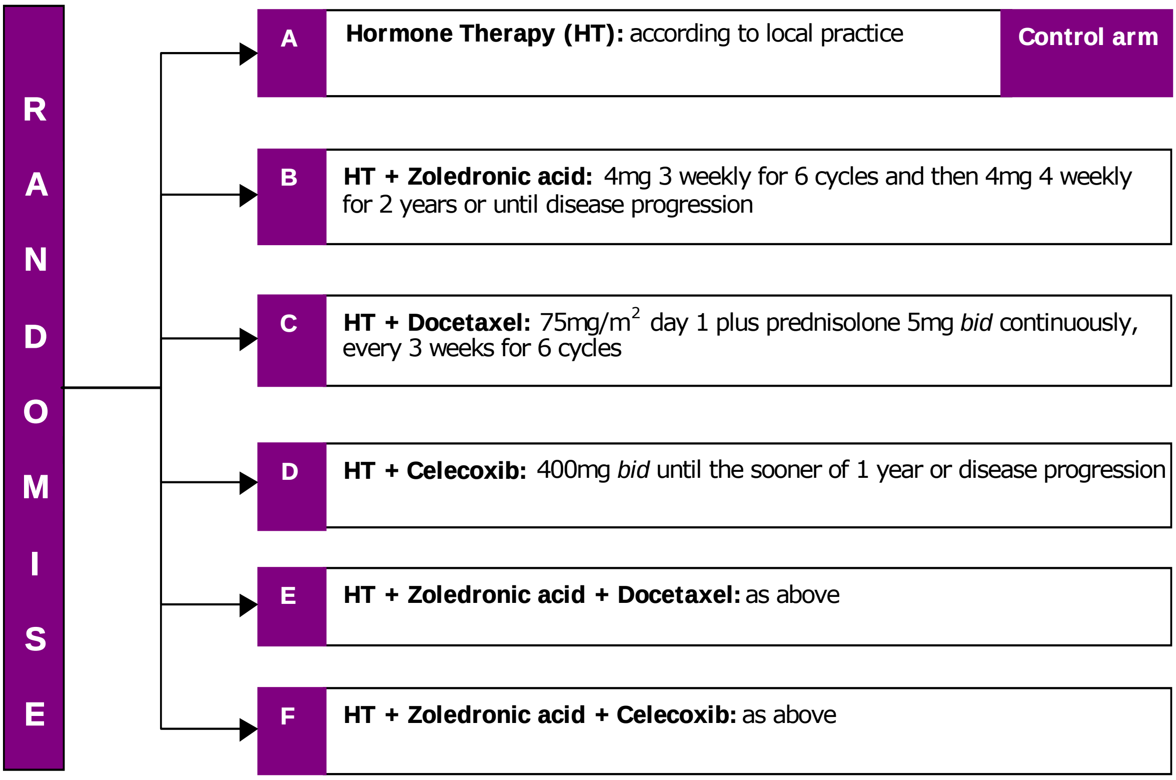

Figure 1b- Arms of the STAMPEDE Trial from April 2011 onwards

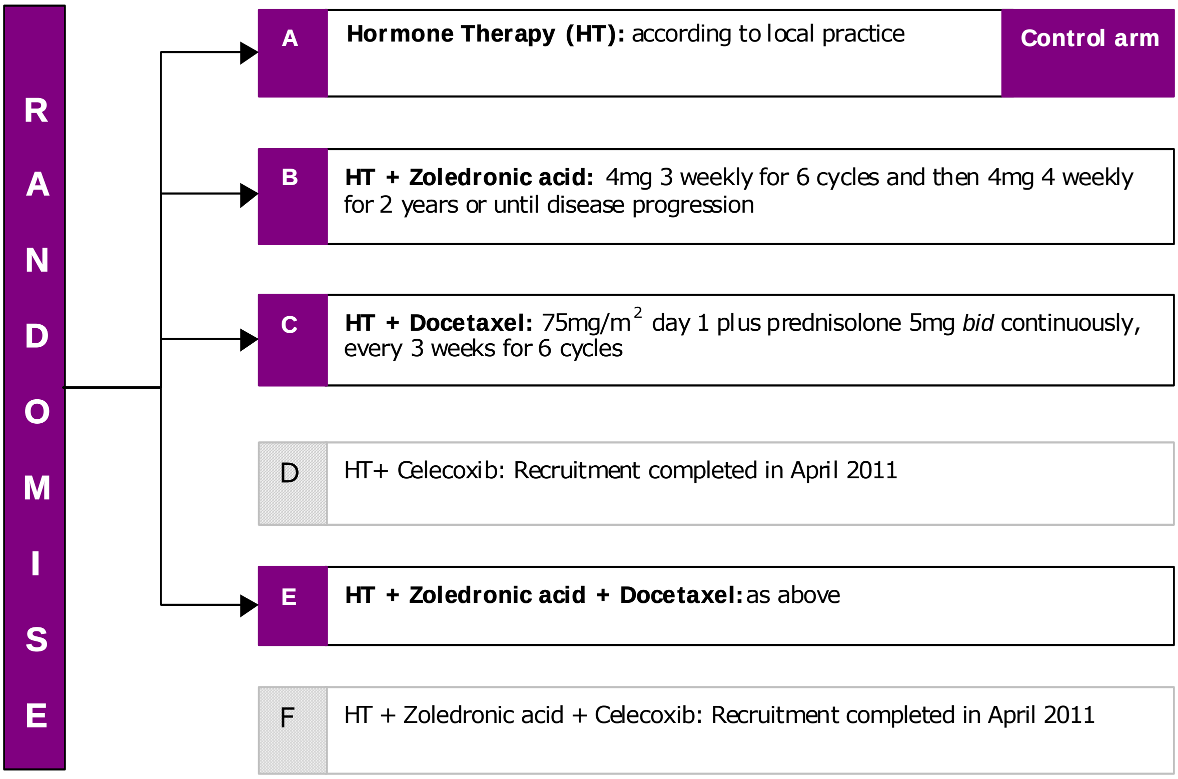

The trial will be conducted in five stages: a Pilot Phase, Activity Stages I to III and Efficacy Stage IV. The primary outcome measure of the Pilot Phase is the safety of the investigational arms and 210 patients will be recruited. Investigational arms will only proceed to recruitment in the next stage if they have been shown to be both safe and feasible, although patient data from all patients and all stages will be included in the final analyses. In Activity Stages I-III the primary outcome measure is failure-free survival (FFS). Further patients will be recruited until around 113, 216 and 335 FFS events have been observed in the control arm. Some evidence of activity will be required for a research arm to proceed to further recruitment in each stage and guidelines are in place. In Efficacy Stage IV, patients will be recruited until around 405 deaths have been reported in the control arm. Overall, approximately 3,300 patients will be recruited over 5-7 years although the exact number of patients and duration will depend on the observed accrual rate, observed event rate and the number of patients accruing at each stage.

Recruitment to arms D (HT + celecoxib) and F (HT + zoledronic acid + celecoxib) was stopped in April 2011 after the second planned activity analysis revealed a lack of sufficient activity. Refer to section 9.3.3 for further information regarding the guidelines for stopping accrual to research arms during the activity stages of the trial.

Patients will be assessed 6 weekly for the first 24 weeks after randomisation and then every 12 weeks up to 2 years, then 6-monthly until 5 years and annually, thereafter. The first 700 patients on trial will also be asked to complete questionnaires aimed at assessing the effects of the investigational treatments on their quality of life (QL) and on their use of health care resources (Health Economics (HE) study).

In addition, there are translational sub-studies. Patients willing to participate will be asked to donate a droplet of blood at randomisation which will be stored for either DNA and protein analysis in order to try to identify markers that are associated with response to therapy, side-effects or susceptibility to prostate cancer.

Patients will also be asked to give permission to use some of their stored material for further studies on the causes and nature of prostate cancer. In selected centres patients will also be asked to participate in a bone mineral density sub-study. There are separate patient information sheets for the QL and HE study and the translational sub-studies (For further details of ancillary studies see section 17).

1.3 TRIAL DOCUMENTATION

Figure 2 presents a summary of the required trial documentation for participating centres and Figure 3 presents a summary of the timings of the case report forms (CRFs) for your randomised patients.

Figure 2 - Summary of trial documentation required ahead of accreditation

Trial documentation	Timing
R&D approval	Before centre participation
Investigator Statement	Before centre participation
Signature list & delegation of responsibilities	Before centre participation
Trial personnel contact details	Before centre participation
PIS, GP & CF on local paper	Before centre participation
Signed Clinical Trial Agreement between Trust and Sponsor	Before centre participation

2 BACKGROUND

2.1 INTRODUCTION AND RATIONALE

Prostate cancer is a major health problem world-wide and accounts for nearly one fifth of all newly diagnosed male cancers. In the UK, approximately 35,000 men are diagnosed with prostate cancer each year and in 2001 almost 10,000 men died from the disease (1).

The initial (first line) treatment for locally advanced or metastatic prostate cancer is hormone therapy (HT) achieved either surgically with bilateral orchidectomy, or medically with LHRH analogues (2) or bicalutamide alone (for M0 patients only). HT produces responses in up to 85% of patients (3) but it is not curative and disease recurs in virtually all patients, with a median time to progression of 18-24 months. Such disease is referred to as hormone refractory prostate cancer (HRPC).

There are several treatments, which are used 'second-line' in patients with HRPC, but no evidence as to which is associated with the best response or whether any of them might have a role in first-line treatment; these include further hormonal manipulations (4;5), bisphosphonates (6), cytotoxic chemotherapy (3) and novel agents (7). The traditional approach to the testing and introduction of new treatments for prostate cancer is in hormone refractory disease. An alternative approach is to investigate new drugs and new approaches to treatment as first-line therapy in patients starting hormone therapy. At this point patients would be fitter and better able to tolerate treatment than when they have HRPC, and there is also the possibility of having a larger and more durable effect.

STAMPEDE (also known as MRC PR08) is an innovative, multi-arm multi-stage, multi-centre, randomised controlled trial. It assesses the effects of a bisphosphonate (zoledronic acid), a cytotoxic chemotherapeutic agent (docetaxel) and a cyclooxygenase (Cox-2) inhibitor (celecoxib), as single agents or combinations, in patients commencing hormone therapy for advancing or metastatic prostate cancer. The trial is divided into five stages such that, for each investigational arm, safety and activity data are generated in the first four stages; an investigational arm will only proceed to the fifth and final stage of recruitment, where it will be assessed for its effect on overall survival, if it has been shown to be safe and active. It is important to note, however, that patient data from all arms and all stages will be included in the final analyses of the primary outcome measure, even if the investigational arm did not proceed to the final stage.

2.2 BISPHOSPHONATES

The bisphosphonates are a class of drug that act by reducing osteoclast formation, inhibiting osteoclast activity and inducing osteoclast apoptosis. They are effective at controlling

hypercalcaemia and preventing skeletal complications associated with malignant disease (8). Zoledronic acid is a new, highly potent, third generation bisphosphonate; studies comparing the efficacy of zoledronic acid to other bisphosphonates suggest that zoledronic acid has a 40-850 fold higher potency than clodronate in preclinical models of bone resorption (9). It has also been shown to be more effective than pamidronate (90mg) in controlling malignant hypercalcaemia (10). In addition, zoledronic acid has also demonstrated direct anti-cancer activity, including inhibition of proliferation of breast cancer and prostate cancer cells *in vitro* (11).

In randomised controlled trials of 1,648 patients, 4mg zoledronic acid was more effective than pamidronate in reducing the risk of skeletal complications in patients with bone metastases from breast cancer (12). Also, in metastatic prostate cancer, zoledronic acid has been shown to reduce the rate of skeletal related events compared to placebo in a trial involving 429 men (13). In April 2002, zoledronic acid received approval from the Committee for Propriety Medicinal Products for the prevention of skeletal related events (for example, fractures) in patients with any advanced malignancies involving bone.

The MRC PR05 prostate cancer trial showed that a first generation bisphosphonate (clodronate) commenced at the time of hormone therapy initiation, delayed time to progression in patients with bony metastatic disease and there was some evidence that it may also improve survival (14). There is, therefore, a good rationale for investigating a more potent bisphosphonate in patients with prostate cancer who are about to commence HT therapy.

2.3 CHEMOTHERAPY

Over recent years there has been increasing evidence of the clinical efficacy of chemotherapy in prostate cancer. One of the most active agents is docetaxel, a semi-synthetic taxane that binds microtubules and inhibits mitosis (15). In phase I and II trials, docetaxel exhibited significant activity in prostate cancer in dosing regimens in the range of 40 to 75 mg/m^2, administered every three weeks. Evidence of this activity includes PSA decline, objective response in bi-dimensionally measurable lesions and improvements in pain control. The safety profile was assessed as being acceptable throughout these studies with a good risk/benefit ratio in this clinical setting (16-19). In addition, the concurrent administration of prednisolone has been shown to materially decrease the toxicity associated with docetaxel (20).

Recent evidence also indicates that docetaxel compares favourably with mitozantrone which had previously been considered the most active chemotherapy agent against prostate cancer. A phase II trial reported PSA response rates of 38-46% with docetaxel with prednisolone compared to 28% for mitozantrone plus prednisolone (21).

More recently two metastatic phase III studies in patients with hormone refractory prostate cancer (HRPC) using a docetaxel-containing regimen have been completed: the SWOG 9916 study (22) and the TAX 327 study (23). Both studies show that the use of a docetaxel-based regimen improved survival for patients with metastatic HRPC and had significantly greater PSA response rates compared to the mitoxantrone plus prednisolone arm.

In the TAX 327 trial (23), 1,006 patients with metastatic HRPC were randomized to receive either mitoxantrone 12 mg/m² with prednisone 10mg daily (Arm C) or docetaxel 75mg/m² q3 weekly x 10 cycles and prednisone (Arm A) or docetaxel 30 mg/m²/wk x 5 of 6 weeks x 5 cycles with prednisone (Arm B). Median overall survival was 16.5 months for patients treated with mitoxantrone versus 18.9 months for the 3 weekly docetaxel regimen (hazard ratio 0.76 (0.62-0.94)). There was also improvements for 3 weekly docetaxel in pain (22% vs 35%, p = 0.01) and PSA response (32% vs 45%, p = 0.0005)

In June 2006 in the UK docetaxel was given NICE (National Institute for Health and Clinical Excellence) approval for use in hormone refractory prostate cancer patients.

2.4 CYCLOOXYGENASE-2 INHIBITORS

Note: *recruitment completed to both celecoxib-containing arms in April 2011 at the end of Activity Stage II*

Cyclooxygenase-2 (Cox-2) is an isoenzyme induced by a variety of mitogens, cytokines and growth factors that are associated with inflammation, ovulation and carcinogenesis (24-26). There is a growing body of evidence that inhibition of Cox-2 may play an important role in the prevention of cancer and the delay of progression in established cancer. A number of case-control studies have shown a reduction in risk of prostate cancer associated with the use of non-steroidal anti-inflammatory drugs (NSAID), which include inhibition of Cox-2 amongst their mode of action (27). Pathological studies show Cox-2 is upregulated in prostate carcinoma (28) and one study suggested that NSAID use may delay progression from subclinical to clinical prostate cancer (29).

Celecoxib, a Cox-2 inhibitor, is better tolerated than other NSAIDs and there is evidence that it is active as a chemoprevention agent (30). It also has important antineoplastic properties such as the ability to inhibit angiogenic factors and induce apoptosis in human cancer cells including prostate cancer (31;32).

Evidence has suggested that an anti-cancer effect is only seen at higher doses of celecoxib than required for an anti-inflammatory effect (33). Therefore, the dose of 800mg/day for STAMPEDE patients has been chosen. Although there is some high profile evidence of a small absolute

increase in CVS toxicity risk associated with higher doses of celecoxib (34), most current cancer trials are using a dose of 800mg/day as it is believed that a higher dose will result in a greater increase in cancer effect.

There is also some evidence of a schedule effect on CVS toxicity. It has been observed that CVS toxicity becomes evident after 1 year of taking celecoxib (34). Therefore, a maximum duration of 1 year has been set for celecoxib use in this trial. Any potential risks of course have to be weighed against any potential benefits of celecoxib in the delay of progression in established prostate cancer.

Given case-control data suggesting effects on prostate cancer, pathological expression of Cox-2 in prostate cancer and *in vitro* data suggesting that inhibition of Cox-2 inhibits growth and invasiveness, further investigation in prostate cancer is warranted.

2.5 TREATMENT COMBINATIONS

2.5.1 Bisphosphonate and Chemotherapy

Zoledronic acid and docetaxel have different mechanisms of action. In addition to its skeletal protection activity, zoledronic acid has shown direct activity against prostate cancer cells, both *in vitro* and *in vivo* (11;35;36). There is also *in vitro* and *in vivo* evidence to suggest synergy between zoledronic acid and chemotherapy in breast cancer cells and anti-angiogenic effects in patients (37;38).

Toxicities of the two agents are complementary and administration in combination is expected to be feasible and safe. These aspects will be evaluated in the initial Pilot Phase of the trial. Since both agents show considerable promise as single agents and there is *in vitro* evidence of synergy, we believe there is a strong rationale for evaluating these two agents in combination.

2.5.2 Bisphosphonate and Cyclooxygenase-2 Inhibitors

Note: *recruitment completed to both celecoxib-containing arms in April 2011 at the end of Activity Stage II*

An alternative approach to combination therapy is to target the principal site of relapse and a key mode of progression and this is the rationale for combining zoledronic acid with a Cox-2 inhibitor. Bisphosphonates have already been shown to delay bone disease progression in hormone refractory disease (14). Cox-2 appears to play a crucial role in the molecular phenotype of advanced prostate cancer as outlined above, and this effect is likely to be apparent in both soft tissue and in bone. Toxicities of the two agents are likely to be complementary and there is no strong *a priori* reason to anticipate unacceptable toxicity. The Pilot Phase of the trial will evaluate tolerability and safety of the combination. Targeting both bone progression and the

underlying molecular changes leading to progression can be expected to have synergistic benefits in terms of delaying development of hormone refractory disease.

3 SELECTION OF INSTITUTIONS AND INVESTIGATORS

Centres who wish to participate in the STAMPEDE trial should be registered with the Medical Research Council Clinical Trials Unit (MRC CTU) for this purpose. Before any patients are randomised the MRC CTU must receive a completed and signed Investigator Statement. The STAMPEDE investigator statement is signed by the Principal Investigator for that institution on behalf of all staff at that site who will be working on the STAMPEDE trial (**Appendix M**). R&D approval for the site along with a fully-signed model agreement are also required before recruitment can begin.

In addition and in compliance with GCP all institutions participating in the trial will complete a delegation log and forward this to the MRC CTU. Each person working on the STAMPEDE trial must sign off a section of this log indicating their responsibilities. The MRC CTU must be notified of any changes to trial personnel and/or their responsibilities. An up-to-date copy of this log must be stored in the Investigator Site file at the institution and also at the MRC CTU.

The Clinical Trial Authorisation (CTA) for the STAMPEDE trial requires that the Medicines and Healthcare Products Regulatory Agency (MHRA) be supplied with the names and addresses of all participating investigators/institutions. Trial staff at the MRC CTU will perform this task; hence, it is vital to receive full contact details for all investigators prior to their entering patients.

Finally, before a patient is entered into the trial written informed consent must be obtained. Approved patient information sheets and informed consent forms are supplied in **Appendix B**. .

Only a limited number of centres participated in the Pilot Phase of the trial; this was to ensure that safety and feasibility data were collected expediently. The other stages of the trial will be open to any centre that wishes to participate and has fulfilled the requirements described above.

4 SELECTION OF PATIENTS

4.1 PATIENT INCLUSION CRITERIA

Patients must fulfil **one** of the criteria in section 4.1.1 or **one** of the criteria in section 4.1.2. Additionally, **all** patients must fulfil the criteria in section 4.1.3.

4.1.1 High Risk Newly Diagnosed Patients

…with one of:-

(i) Fulfil at least two out of the three following criteria, Stage T3/4 N0 M0 histologically confirmed prostate adenocarcinoma, PSA≥40ng/ml or Gleason sum score 8-10

(ii) Stage T_{any} N+ M0 or T_{any} N_{any} M+ histologically confirmed prostate adenocarcinoma

(iii) Multiple sclerotic bone metastases with a PSA≥100ng/ml without histological confirmation

OR

4.1.2 Patients with histologically confirmed prostate adenocarcinoma previously treated[Ψ] with radical surgery or radiotherapy who are now relapsing

…with at least one of:-

(i) PSA ≥4ng/ml and rising with doubling time less than 6 months

(ii) PSA ≥20ng/ml

(iii) N+

(iv) M+

[Ψ]**Note:** Prior hormone therapy for localised disease must have been completed at least 12 months previously and have been no longer than 12 months in duration. It can have been given as adjuvant or neoadjuvant therapy.

AND

4.1.3 For all patients

(i) Intention to treat with long-term hormone therapy

(ii) Fit for all protocol treatment[φ] and follow-up, WHO performance status 0-2 [ὢ]

(iii) Have completed the appropriate investigations prior to randomisation

(iv) Adequate haematological function: neutrophil count ≥1.5x10^9/l and platelets ≥100x10^9/l

(v) Adequate renal function: Serum creatinine ≤1.5 ULN

(vi) Adequate liver function: ALT or AST ≤1.5 ULN, bilirubin ≤ULN

(vii) Written informed consent

Willing and expected to comply with follow-up schedule

[ὢ] For WHO performance status definitions see **Appendix A**

[φ]: Medical contraindications to the trial medications are given in **Appendix G**

4.2 PATIENT EXCLUSION CRITERIA

(i) Prior systemic therapy for locally advanced or metastatic prostate cancer except as listed in 4.1.2.
(ii) Metastatic brain disease or leptomeningeal disease
(iii) Any other previous or current malignant disease which, in the judgement of the responsible physician, is likely to interfere with STAMPEDE treatment or assessment
(iv) Patients with active peptic ulceration, gastrointestinal bleeding, inflammatory bowel disease
(v) Symptomatic peripheral neuropathy ≥ grade 2 (NCI CTC)∞
(vi) Any surgery (e.g. TURP) performed within the past 4 weeks
(vii) Renal insufficiency with estimated creatinine clearance <30ml/min
(viii) Patients who have been on a Cox-2-inhibitor for at least 6 months prior to trial entry
(ix) Patients with confirmed severe cardiovascular history e.g.:

   a. Severe/unstable angina
   b. Myocardial infarction
   c. Severe cardiac failure (NYHA II-IV*)
   d. Cerebrovascular disease (eg stroke or transient ischaemic episode)

(X) Patients who have scheduled to have major dental extractions within the next 2 years

∞ See **Appendix G** for common toxicity gradings

*NYHA classifications can be found in **Appendix A**

4.3 SCREENING PROCEDURES

4.3.1 Investigations Prior to Randomisation

All patients should have the following examinations performed the latest available scans should be used:

- CT or MRI of pelvis and abdomen
- Bone Scan
- Chest X-ray (only if chest was not included in CT)
- ECG
- PSA Test

The following blood tests within 8 weeks (56 days) prior to randomisation:-

- Testosterone (if available)
- Urea and Electrolytes
- Liver function tests
- Serum creatinine
- Serum corrected calcium
- Phosphates

- Magnesium
- Albumin
- Total Cholesterol
- HDL Cholesterol
- Systolic blood pressure
- Diastolic blood pressure

It is preferable that patients are **not** started on hormones prior to randomisation. However, if this has occurred, then LHRH analogues / agonists should have not have started more than 12 weeks before randomisation and the baseline PSA measurement must be taken before the treatment was initiated (please report the latest PSA measurement taken before the start of hormone therapy). Testosterone measurement will not be required in patients who have already commenced hormone manipulation. In addition, it is preferable that patients are not started on anti-androgens prior to randomisation. However, if this has occurred then the patients should not have received more than 4 weeks of anti-androgen treatment before randomisation.

For patients who are hypercalcaemic prior to randomisation and require treatment, it is recommended that they are treated with a bisphosphonate and that the treatment should be discontinued when they are stabilised.

For patients who are currently on a Cox-2-inhibitor and who meet the inclusion criteria, please ensure that treatment is discontinued before randomisation. If the patient is allocated to an arm, which does not include celecoxib (arms A, B, C or E), it is advised that the Cox-2 be replaced with a suitable NSAID.

For patients who are taking an NSAID prior to randomisation and are allocated a celecoxib arm (Arm D or F), a clinical decision should be taken as to whether the patient should continue taking the NSAID alongside the celecoxib. This decision should take into account the risk of gastrointestinal problems, and consideration should be given to the co-administration of a proton pump inhibitor

Treatment should be commenced as soon as possible after randomisation, investigators should aim that this is at least within 4 weeks post randomisation and within 12 weeks of starting hormone therapy.

4.3.2 Concomitant Medications

All concomitant medications should be recorded including any vitamin and mineral supplements the patient is taking, regular consumption of NSAID and/or aspirin and use of other

bisphosphonates (see **Section 4.3.1**). All concomitant medications should be continued throughout the trial unless the responsible physician decides otherwise.

4.3.3 Additional Details for Patients Participating in the Sub-studies

An additional droplet of blood must be taken if the patient has given their consent to participate in the DNA analysis sub-study.

The local pathologist will also be asked to give the remaining tumour sample for tissue micro array analysis to be carried out, if the patient has given consent for his remaining samples to be used for further analyses. In selected centres, patients will also be asked to participate in a bone mineral density sub-study. Full details of all sub-studies and instructions relating to the handling of the blood sample are given in Section 17 and **Appendix D**.

5 RANDOMISATION AND ENROLMENT

To enter a patient the randomisation form should be completed and the MRC CTU contacted by phone:

RANDOMISATIONS

To randomise call MRC CTU, Monday to Friday 0900-1700 excluding public holidays or dates when notice has been given by the CTU.

Tel: + 44 (0)20 7670 4777

A trial number and treatment will be allocated and given over the phone or by return fax. In addition, a letter confirming these details will be sent. The trial number will be the primary way in which the patient will be identified and should be used in all correspondence.

5.1 CO-ENROLMENT GUIDELINES

Ideally, patients should not be participating in any other clinical trial of prostate cancer treatment when they enter STAMPEDE and should not enter any other trials until the patient has had a failure-free survival (FFS) event reported. After this point, the patient may be entered into further, second-line treatment studies. The primary outcome measure of STAMPEDE is overall survival. Therefore, follow-up to STAMPEDE must continue and must not be affected by co-enrolment to other studies. It is preferable that the MRC CTU should be notified in writing, with details of the trial: trial name, ISRCTN or NCT, sponsor, randomisation arms, study endpoints and a declaration that STAMPEDE follow-up will not be impeded, *before a patient is co-enrolled.*

6 TREATMENT OF PATIENTS

6.1 TRIAL TREATMENT

Patients will be randomised to the control arm (Arm A) or one of the investigational arms. All patients will receive HT to achieve castration levels of testosterone. The method of HT is a local choice but must be specified for each patient prior to randomisation. The recommended methods of HT are given in section 6.1.1. **All trial treatments should commence as soon as practically possible after randomisation.** Patients having a bilateral orchidectomy should commence any additional treatment with 4 weeks of the operation unless there is a strong clinical reason not to do so.

6.1.1 Arm A (control arm) – Hormone Therapy (HT) Only

The recommended methods of HT are bilateral orchidectomy, LHRH analogues, LHRH antagonists or anti-androgen, bicalutamide alone (M0 patients only) (see below). Please note that any other anti-androgens alone are not permissible as hormone therapy for patients participating in STAMPEDE, but their use is recommended in the short-term to prevent tumour "flare" which may occur after commencing LHRH analogues. Anti-androgen prophylaxis of tumour flare is not required when using LHRH antagonists. At the time of randomisation, centres will be asked to specify the method of HT for each patient. Other methods of HT should be discussed with the Chief Investigator or the Trial Physician. The planned duration of HT should be at least 2 years.

- **Bilateral orchidectomy** Operations should be performed by appropriately trained surgeons. A total or subcapsular orchidectomy may be performed.
- **LHRH agonists** LHRH analogues used according to local practice. The prophylactic use of anti-androgens to prevent tumour "flare" is recommended.
- **LHRH antagonists** used according to local practice. The use of prophylactic use of anti-androgens to prevent tumour "flare" is not necessary.
- **Anti-androgen, bicalutamide alone** can be given to patients who are M0 and should be used according to local practice

6.1.2 Arm B - Hormone Therapy + Zoledronic Acid

- **Hormone Therapy** as described in section 6.1.1.
- **Zoledronic Acid** 4mg 15min IV infusion every 3 weeks, for 6 treatments followed by zoledronic acid 4mg 15min IV infusion every 4 weeks up to a maximum of 2 years from the start of the treatment or until disease (including PSA) progression (see Section 7.2). Patients should also receive an oral supplement of 500mg calcium and 400IU vitamin D daily. These doses are available as a combination tablet.

6.1.3 Arm C - Hormone Therapy + Docetaxel

- **Hormone Therapy** as described in section 6.1.1.
- **Docetaxel** 75mg/m^2 Day 1 as 1hr IV infusion, plus prednisolone 5mg *bid* daily for 21 days. The cycle should be repeated every 3 weeks for a maximum of 6 cycles. The recommended administration schedule, anti-emetic regimen and dose modifications for docetaxel are given in **Appendix F**.

6.1.4 Arm D - Hormone Therapy + Celecoxib

Note: *recruitment completed to both celecoxib-containing arms in April 2011 at the end of Activity Stage II*

- **Hormone Therapy** as described in section 6.1.1.
- **Celecoxib** 400mg *bid* until the sooner of 1 year or disease (including PSA) progression (see Section 7.2).

6.1.5 Arm E - Hormone Therapy + Docetaxel + Zoledronic Acid

- **Hormone Therapy** as described in section 6.1.1.
- **Docetaxel** 75mg/m^2 Day 1 as 1hr IV infusion, plus prednisolone 5mg *bid* daily for 21 days. The cycle should be repeated every 3 weeks for a maximum of 6 cycles. The recommended administration schedule, anti-emetic regimen and dose modifications for docetaxel are given in **Appendix F**.
- **Zoledronic Acid** 4mg 15min IV infusion every 3 weeks, for 6 treatments followed by zoledronic acid 4mg 15min IV infusion every 4 weeks up to a maximum of 2 years from the start of the treatment or until disease (including PSA) progression (see Section 7.2). Patients should also receive an oral supplement of 500mg calcium and 400IU vitamin D daily. These doses are available as a combination tablet.

- **Co-administration of docetaxel and zoledronic acid:** Docetaxel 75mg/m^2 Day 1 as 1hr IV infusion, plus prednisolone 5mg *bid* daily followed by zoledronic acid 4mg 15min IV infusion. There is evidence to suggest that the co-administration of docetaxel and zoledronic acid is sequence dependent (39). Consequently, docetaxel should be administered *before zoledronic acid*

6.1.6 Arm F - Hormone Therapy + Zoledronic Acid + Celecoxib

Note: *recruitment completed to both celecoxib-containing arms in April 2011 at the end of Activity Stage II*

- **Hormone Therapy** as described in section 6.1.1.
- **Zoledronic Acid** 4mg 15min IV infusion every 3 weeks, for 6 treatments followed by zoledronic acid 4mg 15min IV infusion every 4 weeks up to a maximum of 2 years from the start of the treatment or until disease (including PSA) progression (see Section 7.2).

Patients should also receive an oral supplement of 500mg calcium and 400IU vitamin D daily (Calcichew). These doses are available as a combination tablet.

- **Celecoxib** 400mg *bid* until the sooner of 1 year or disease (including PSA) progression (see Section 7.2).

6.2 ADMINISTRATION AND DOSE MODIFICATIONS

6.2.1 Zoledronic Acid

Zoledronic acid will be administered by IV infusion in accordance with the instructions in the summary of product characteristics at a dose of 4mg every 3 weeks for the first 6 cycles, and thereafter every 4 weeks. There may be the opportunity for the 4-weekly infusions to be administered in the patient's home (**Appendix H**).

Serum Creatinine Measurements: Serum creatinine should be measured at baseline and within 48 hours prior to every administration of zoledronic acid.

Serum Electrolytes and FBC: Serum electrolytes including calcium, phosphate and magnesium should also be measured prior to each infusion. FBC should be measured at least 3 monthly.

Zoledronic acid should be discontinued if there is any evidence of hypersensitivity to the drug. In patients with mild to moderate renal impairment, lower doses of Zoledronic acid are recommended. In rare cases, zoledronic acid treatment has been associated with the development of osteonecrosis of the jaw, particularly following dental extractions. If a patient develops osteonecrosis of the jaw then the zoledronic acid should be immediately and permanently discontinued. For full details of zoledronic acid administration and does reductions see **Appendix F.** Contraindications, special precautions, interactions and side effects are listed in **Appendix G.**

6.2.2 Docetaxel

The use of docetaxel should be confined to units specialised in the administration of cytotoxic chemotherapy and it should only be administered under the supervision of a physician qualified in the use of anticancer chemotherapy.

Docetaxel will be administered by IV infusion in accordance with the instructions in the summary of product characteristics at a dose of 75mg/m^2 (up to a maximum dose of 160mg) on day 1 of the study treatment period and then every 3 weeks, thereafter, for a maximum of 6 doses. Patients with a body surface area (BSA) greater than 2.13m^2 should be dosed as though they have a BSA of 2.13m^2. No ideal weight should be used for BSA calculations. Prednisolone 5mg *bid* will be given until completion of chemotherapy. Additional dexamethasone should be given pre- and post-docetaxel infusion to suppress allergic reactions.

Please note that liver function test (LFTs) should be carried out within a week before the first cycle of docetaxel if an anti-androgen has been administered. This is due to an increased risk of neutropenia associated with docetaxel use following anti-androgen administration. Treatment should be delayed if LFTs are abnormal.

For full details of premedication schedule, recommended anti-emetic regimen and dose modifications for docetaxel see **Appendix F**. Contraindications, special precautions, interactions and side effects are listed in **Appendix G.**

Docetaxel in combination with prednisone or prednisolone is indicated for the treatment of patients with hormone refractory metastatic prostate cancer (22)

6.2.3 Celecoxib

Note: *recruitment completed to both celecoxib-containing arms in April 2011 at the end of Activity Stage II*

Celecoxib should be administered in accordance with the instructions in the summary of product characteristics at a dose of 400mg *bid* orally. Rarely this drug is poorly tolerated and in this instance should be discontinued, particular care should be taken with patients with a history of gastrointestinal disease and patients with significant risk factors for cardiovascular events. (see **Appendix G**). Patients with confirmed severe cardiovascular history should not be in STAMPEDE (see exclusion criteria, section 4.2). Contraindications, special precautions, interactions and side effects are listed in **Appendix G.** Dose reductions are not anticipated.

6.3 TRIAL PRODUCTS

Details of the procedures for obtaining the drugs within the trial, dispensing and disposal of unused drug are given in **Appendix E**.

Arrangements for free or discounted drugs are given in the Finance section (section 15).

6.4 MEASURES OF COMPLIANCE/ADHERENCE

Date of treatment, dose, delays and reasons for delays or dose modifications of all study infusions (zoledronic acid and docetaxel) will be recorded. The estimated number of celecoxib tablets taken in a given time period will also be recorded as well as any dose reductions.

6.5 TREATMENT DATA COLLECTION

Data will be recorded on case report forms (CRFs); the top copy/original should be sent to the MRC CTU for data entry and a copy kept at the local centre. The data to be recorded on these can be viewed in the CRF appendix (**Appendix Q**). The type of data to be recorded is detailed in the Assessments and Procedures section **(Section 7)**.

6.6 NON-TRIAL TREATMENT

6.6.1 Medications permitted

Any additional treatment that the responsible physician feels is appropriate is permitted.

6.6.2 Data on concomitant medication

All concomitant medication will be recorded on the baseline form prior to randomisation and on any subsequent Serious Adverse Event forms. This should include aspirin that may be taken on a regular basis for cardiovascular disease, the use of any Non-Steroidal Anti-inflammatory Drugs (NSAID) as well as any vitamin or mineral supplements the patient is taking.

6.6.3 Radiotherapy

Investigators should aim to give radiotherapy (RT) to patients with T_{any}N0M0 disease in accordance with the data from the PR07 and SPCG trials; these patients remain eligible for STAMPEDE. For patients with node positive, non-metastatic disease, radiotherapy is recommended in suitable cases. **Appendix P** details the type, timing, dose and duration of radiotherapy recommended for STAMPEDE patients. Data on RT will be collected and the planned use of RT is a stratification factor in the trial analysis.

6.6.4 Data on Radiotherapy

There are two CRFs to be completed for patients receiving primary radiotherapy. All radiotherapy and acute side effects details will be recorded on the Radiotherapy Form and any late side effects will be recorded on the Late Toxicity Form. For patients who receive palliative radiotherapy, a Palliative Radiotherapy CRF should be completed.

7 ASSESSMENTS AND PROCEDURES

7.1 FLOW CHART / SCHEDULE FOR FOLLOW-UP

A detailed follow up schedule is given in **Figure 4**.

7.1.1 PSA Measurements

All patients should have PSA measured pre-hormone therapy and at weeks 6, 12, 18 and 24 and every 12 weeks, thereafter, up to 2 years post randomisation. Following this, PSA should be measured every 6 months until 5 years and annually, thereafter. For patients who do not have a scheduled hospital visit, it would be acceptable for arrangements to be made for blood samples to be drawn either in a GP's surgery or in the patient's home.

7.1.2 Assessment of treatment failure (Definition of progression)

It is not proposed to routinely assess patients for response. However, in order that objective progression can be assessed, it is necessary to have imaging taken at time of best response as judged by the treating clinician. All patients should have baseline radiological examinations as detailed in section 4.3.1. In addition it is recommended that all patients should have scans or X-rays repeated at 24 weeks (and whenever clinically appropriate) if they were abnormal at baseline, particularly if they have a low PSA value on entry in to the trial making biochemical assessment of treatment failure difficult. The following events would constitute a disease progression and should be reported on a progression form:

- Biochemical failure (see appendix K)
- Local progression
- Lymph node progression
- Progression in distant metastases
- Development of new metastases

Please note that skeletal related events (SREs) may be indicative of disease progression. All SREs should be investigated further to establish whether or not the patient has progressed, in which case a progression form should be completed.

7.2 FOLLOW-UP

Every effort should be made to follow-up all patients who have been randomised. Patients should, if possible, remain under the care of an oncologist or urologist for the duration of the trial. If care of a patient is returned to the GP, it is the responsibility of the consultant who obtained the patient's consent to participate in the trial to ensure that the data collection forms are completed. If the patient moves from the local area, arrangements should be made for trial follow-up to be undertaken by their new local centre. Details of other participating centres can be obtained from the MRC CTU. The consent of patients should be obtained for their names to be

flagged for survival information through national registries (e.g. ONS in England/Wales and GRO in Scotland). If the clinician moves, appropriate arrangements should be made to arrange for trial follow-up to continue at the centre.

7.3 TRIAL CLOSURE

For the purpose of complying with UK Clinical Regulations introduced on May 2004, the trial will be considered '*closed*' when the last patient has completed protocol treatment. However, further observational follow-up of all patients enrolled in the trial will continue until all randomised patients have died. This will initially be via the hospital, but in the longer term may employ national registers.

Figure 3 – Summary of timing of case report forms

-Case Report Forms	Timing
Bone Density Risk Factor form	At Randomisation
Randomisation Form	At randomisation
Baseline Form	At randomisation
Cardiovascular Assessment Form	At randomisation
Pathology Form	At randomisation. When pathology sample has been taken and sent to UCL laboratory
Pre-18 Week Bisphosphonate Form	Treatment administered every 3. Form holds data for 2 cycles. Form to be sent after 2nd cycle given
Post-18 Week Bisphosphonate Treatment Form	Treatment administered every 4. Form holds data for 3 cycles. Form to be sent after 3rd cycle given
Docetaxel Treatment Form	Treatment administered every 3 weeks Form holds 2 cycles. Form to be sent after 2nd cycle given
Follow-Up Form<br>(see fig 3b below for information on date required at follow up)	Every 6 weeks for 6 months, then every 12 weeks until 2 years. Every 6 months until 5 years and annually thereafter
Quality of life form	Every 6 weeks for 6 months, then every 12 weeks until 2 years, every 6 months until 5 years and annually thereafter.*
Radiotherapy Form	If applicable, when primary radiotherapy course has finished.
Late Radiotherapy Toxicity Form	If applicable, 6m, 12m 24m and 36m from start of radiotherapy
Palliative Radiotherapy Form	If applicable, when the palliative radiotherapy course is completed
End of Treatment Form	When each treatment is completed (either at end of scheduled treatment or at early cessation of treatment)
Progression & Additional Treatment Form	At the first occurrence of each type of progression and whenever a patient that has progressed receives additional treatment
Serious Adverse Event Form	Following any Serious Adverse Event
Skeletal Related Event Form	Whenever a patient experiences a skeletal related event
BMD substudy assessment forms	
Death Form	At Death

*Quality of Life Study is only for first 700 patients entered into the trial. MRC CTU will inform centres of which of their patients this applies to.

Figure 3b – Data required on follow up forms

Timing of follow-up	PSA	Evidence of Progression	Hormone Therapy	Cox-2 treatment	Unscheduled Visits	Toxicities
0-2 years	✓	✓	✓	✓	✓	✓
After 2 years	✓	✓	✓	-	✓	-
After Progression	-	✓	✓	-	✓	-

Figure 4 - Schedule for completion of forms by arm.

Timing of Assessment	**Baseline**		**Treatment**		**Outcomes**		Freq
	Randomis^{n}	Pre-Treatment	Zoledronic acid	Docetaxel	Follow-up$^{\Psi}$	QL + HE¥	
Yr 0 Wk 0	all	all				all	
Wk 6			BEF †	CE †	all	all	6 weekly
Wk 12			BEF †	CE †	all	all	
Wk 18			BEF †	CE †	all	all	
Wk 24			BEF ‡		all	all	
Wk 36			BEF ‡		all	all	12 weekly
Wk 48			BEF ‡		all	all	
Wk 60			BEF ‡		all	all	
Wk 72			BEF ‡		all	all	
Wk 84			BEF ‡		all	all	
Wk 96			BEF ‡		all	all	
Yr 2 Month 24 (week 104)			BEF ‡		all	all	6 monthly
Month 30 (week 130)					all	all	
Yr 3 Month 36 (week 156)					all	all	
Month 42 (week 182)					all	all	
Yr 4 Month 48 (week 208) Month 54 (week 234)					all	all	
Yr 5 Month 60 (week 260)					all	all	
annually …					all	all	

Key: all=arms A-F, A=HT alone, B=HT + Zoledronic acid, C=HT + Docetaxel, D=HT + Celecoxib, E=HT + Zoledronic acid + Docetaxel, F= HT + Zoledronic acid + Celecoxib
Note: recruitment completed to both celecoxib-containing arms in April 2011 at the end of Activity Stage II

$^{\Psi}$ See figure 3 for information required at follow up
† Form records data for two cycles
‡ Form records data for three cycles
¥ 1st 700 patients only

Radiotherapy, Late RT Toxicity, Palliative Radiotherapy Progression, SAE, End of Treatment and **Death** forms to be completed as required

Note: an individualised form with a follow-up schedule will be provided for each randomised patient. For patients who are receiving LHRH analogues or bicalutamide alone (if M0), it is assumed that any additional treatment will commence within two weeks of randomisation. For patients who are due to have an orchidectomy it is recognised that surgery will have to be scheduled and the scheduling of any additional treatments may be affected by post-operative recovery. It is recommended that all patients who had abnormal radiological investigations at baseline or present with a low PSA on entry into the STAMPEDE trial should have them repeated 24 weeks after randomisation

8 STOPPING OF TREATMENT OR FOLLOW UP

Patients should be given every encouragement to adhere to protocol treatment and follow-up, in order to reduce biases. However, a patient has the right to withdraw consent for participation in any aspect of this trial at any time.

8.1 STOPPING TRIAL INTERVENTIONS

A patient may stop trial treatment for the following reasons:

1. Progression whilst on therapy (trial treatment **must** be discontinued in this instance)
2. Unacceptable toxicity
3. Intercurrent illness which prevents further treatment
4. Withdrawal of consent for treatment
5. Any alteration in the patient's condition which justifies the discontinuation of treatment in the clinician's opinion

The reason should be recorded on the treatment and/or follow-up forms as well as the End of Treatment form.

Unless a patient states otherwise, it should be assumed that consent is given to continue to record trial data.

8.2 PATIENT TRANSFERS

For patients moving from the area, every effort should be made for the patient to be followed-up at another participating trial centre and for this trial centre to take over responsibility for the patient. A copy of the patient STAMPEDE CRFs will need to be provided to the new site. The patient may need to sign a new consent form at the new site, and until this occurs, the patient remains the responsibility of the original centre.

8.3 WITHDRAWAL FROM THE TRIAL COMPLETELY

If a patient explicitly withdraws consent to have **any** data recorded their decision must be respected and the MRC CTU must be informed in writing. All communication surrounding the withdrawal should be noted in the patient's records and no further STAMPEDE CRFs should be completed for that patient.

Patients can change their minds about withdrawal at any time and re-consent to participate in the trial. Follow-up data should be collected **only** from the point of when consent was re-instated.

9 STATISTICAL CONSIDERATIONS

9.1 METHOD OF RANDOMISATION

Patients will be randomised centrally using a computerised algorithm developed and maintained by the MRC CTU. Randomisation will be performed using the method of minimisation over a number of clinically important stratification factors with an additional random element. To decrease determinability, the factors are not listed here. The trial has 1 control arm and 5 research arms. As the control arm is the comparator arm for all the research arms, it is intended to recruit twice as many patients to the control arm as to each research arm. Therefore, the randomisation ratio will be 2:1:1:1:1:1.

9.2 OUTCOME MEASURES

The overall primary outcome measure for the trial is overall survival (all cause mortality). The design of the trial is such that it is important to have additional intermediate outcome measures to assess each research arm as the trial progresses. These are listed in Table 1. The reasons for different emphases in each recruitment stage are explained in section 9.3.

Table 1: Trial Outcome Measures

Trials stage	Primary outcome measures	Secondary outcome measures
**Pilot phase**	Safety*	Feasibility
**Activity Stage I-III**	Failure-free survival (FFS)†	Overall survival (OS)<br>Toxicity<br>Skeletal related events
**Efficacy Stage IV**	Overall survival	Quality of life<br>Cost effectiveness<br>Failure-free survival†<br>Toxicity<br>Skeletal related events

*Based on toxicity

†Including biochemical failure (see **Appendix K**)

9.3 SAMPLE SIZE

9.3.1 Overall sample size

The overall design for this study is a multi-arm, multi-centre randomised controlled trial (see Figure 4, page 26). There are fives stages to the study; Pilot Phase, Activity Stages I-III and Efficacy Stages IV. Full details of the methodology underlying the trial design are given by Royston *et al*. (40). The sample size calculations were performed using the -stage2- program (version 1.2.0, March 2002) (41) and -stagen- program (version 1.1.1, 18 May 2004), both implemented in Stata 8 (Stata Corp, TX) and using –nstage- program (version 1.0.3, 13 June

2007). The trial is designed under the assumptions in Table 2, and additionally, we assume a slightly higher proportion of non-metastatic than metastatic patients such that the median FFS is two years and median OS four years.

Table 2: Hazard ratio assumptions under null and alternative hypotheses

Size of HR	Pilot	Stage I-III	Stage IV
Under null hypothesis (H_0)	n/a	HR(FFS) = 1.0	HR(OS) = 1.0
Under alternative hypothesis (H_1)	n/a	HR(FFS) = 0.75	HR(OS) = 0.75

The HR of 0.75 for any research arm relative to control would translate into an absolute improvement in FFS of 10%, from approximately 50% to 60% at two years and OS of 10%, from approximately 50% to 60% at four years. A beneficial difference of this size would be clinically worthwhile and, indeed, experience tells us it may be unrealistic to expect a larger difference. Therefore, we have adequately powered the trial to detect a HR of 0.75 for overall survival. This design gives 95% power at Activity Stages I-III and 90% power at Efficacy Stage IV. Further details of the sample size calculations are given in a separate Statistical Design document which is available on request.

Assuming an accrual rate of 500 patients/year then between 2800 and 3600 patients are planned to be entered into the trial over a period of 5½ and 7 years. The exact number of patients to be entered depends on the observed accrual and event rates. The primary analysis on overall survival requires 445 deaths to be observed on the control arm.

9.3.2 Pilot Phase

It is anticipated that 210 patients will be recruited to the Pilot Phase from a limited number of centres over a one year period. Approximately 60 patients will be randomised to the control arm and 30 patients to each of the five research arms each of which will be assessed for safety and feasibility. If recruitment proves infeasible or any of the research arms prove unsafe or not feasible to administer (e.g. poorly tolerated or unexpected toxicity) recruitment to these arms will be discontinued. There are already considerable safety data on the use of docetaxel and zoledronic acid in patients with malignancies including prostate cancer, and on the use of Cox-2 inhibitors (including celecoxib), although mainly from patients with musculoskeletal disorders. There are fewer data on the combination arms; but it is thought very unlikely that any of the research arms will be discontinued during the Pilot Phase. Safety data will continue to be assessed throughout the trial. When 210 patients have been on the trial for a minimum of 18 weeks, the independent Data Monitoring Committee (IDMC) will then review the data from the Pilot phase. Recruitment will continue to the trial during this period as equipoise will remain.

9.3.3 Activity Stages I-III

In the sample size calculations we assume that all research arms successfully pass through the Pilot Phase to Efficacy Stage I and that patients will be recruited at a rate of approximately 500 per year. This is faster than in the Pilot Phase because the trial will recruit from additional centres, both in the UK and internationally. The analysis of Activity Stages I, II and III are planned with 115, 225 and 355 failure-free survival events have been observed in the control arm, respectively.

The Activity Stage analyses will comprise pairwise comparisons of FFS between the control arm and each of the 5 research arms (i=1 to 5). Let HR_i(true) represent the hazard ratio (HR) of the ith research arm to the control arm, and HR_i(observed) the observed value. Discontinuation of accrual of further patients will be considered for the ith research regimen at each of Activity Stages I-III according to the guidelines in Table 3.

Table 3: Guidelines for stopping accrual to the ith research arm

Activity Stage	Number of control arm events	Consider discontinuation if HR_i(observed) is…
I	~113	>1.00
II	~216	>0.92
III	~334	>0.89

9.3.4 Efficacy Stage IV

The analysis of Efficacy Stage IV will be performed when 403 deaths have been observed in the control arm. This would give 90% power to detect the targeted hazard ratio of 0.75 at one-sided significance level of 0.025. The actual length of this stage and the number of patients to be recruited depends on the number of arms passing through to further recruitment from Activity Stages I-III and the observed accrual and event rates.

Figure 5: Progress of STAMPEDE through the trial stages

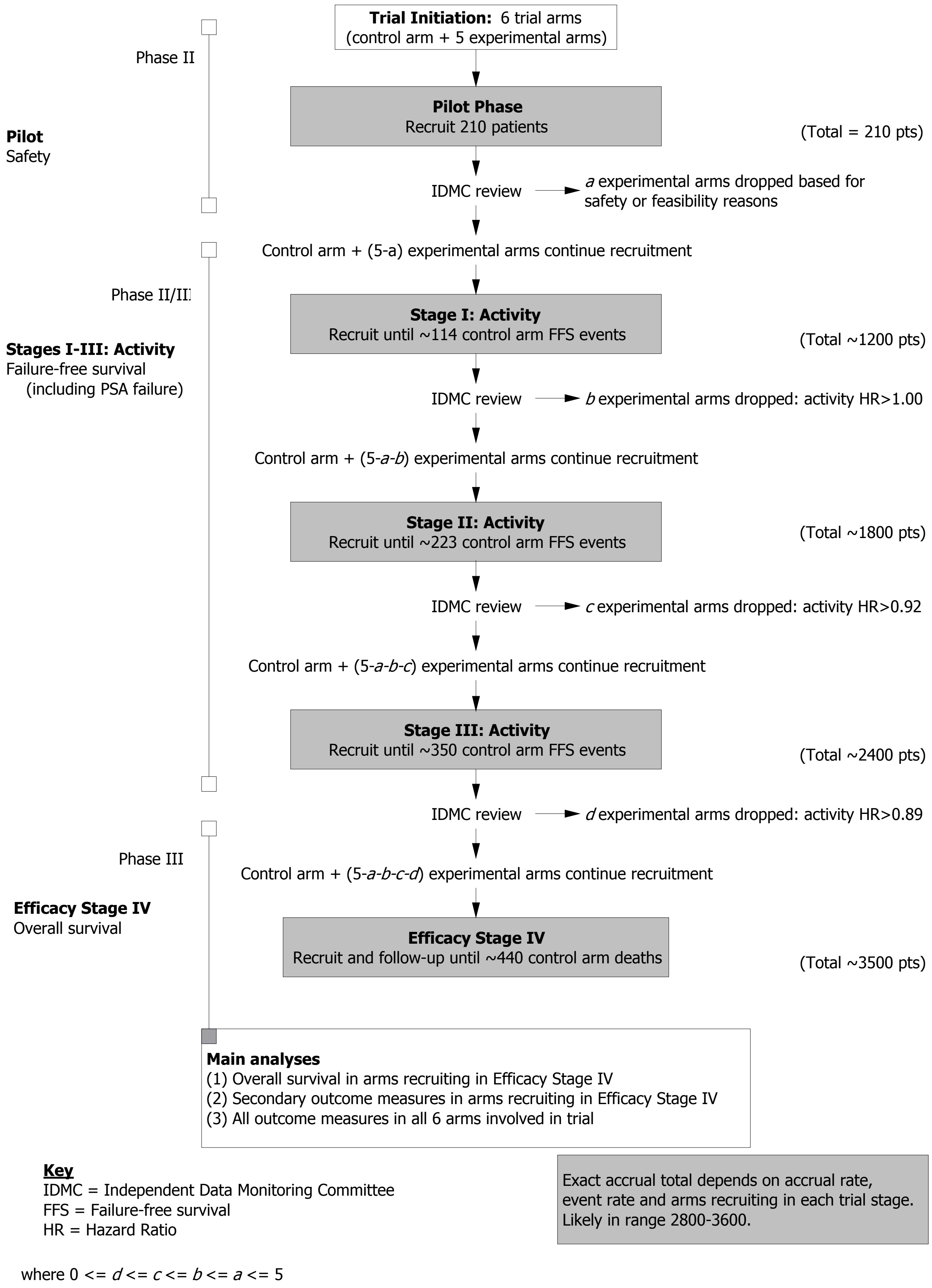

9.3.5 Factorial design

We note here that we have not employed a factorial design in this trial because we anticipate the possibility of synergy between hormone therapy, zoledronic acid and docetaxel and between hormone therapy, zoledronic acid and celecoxib. It would not be possible to assess any such interactions reliably in a factorial trial (see the Statistical Design document for further details).

9.4 INTERIM MONITORING AND ANALYSES

Formal interim analyses of the accumulating data will be performed at regular intervals (approximately annually) for review by an Independent Data Monitoring Committee (IDMC) (see also section 16). These analyses will be performed by the trial team at the MRC CTU. The IDMC will be asked to give advice on whether the accumulating data from the trial with the guidelines for discontinuation of accrual for Activity Stages I-III, together with results from any other relevant trials, justifies continuing recruitment of further patients or further follow-up. A decision to discontinue recruitment, in all patients or in selected subgroups will be made only if the result is likely to convince a broad range of clinicians including those entering patients into the trial and the general clinical community. If a decision is made to continue, the IDMC will advise on the frequency of future reviews of the data on the basis of accrual and event rates. The IDMC will make recommendations to the Trial Steering Committee (TSC, see section 16) as to whether the trial should continue in its present form. While the trial is ongoing the accumulating data will remain confidential.

9.5 OUTLINE ANALYSIS PLAN

Analyses will be performed on an intention-to-treat basis. The standard unadjusted log-rank approach will be applied to analyses of FFS and OS. The impact of potential confounders including the stratification factors used at randomisation will be considered in a Cox proportional hazard model. The χ^2 test or Mann-Whitney test will be implemented for categorical data comparisons, including toxicity, as appropriate. The primary outcome measures in Table 1 (see Section 9.2) will be considered for all arms of the trial at each phase, but the main emphasis will be placed on the comparison of the research arms that have continued to recruit throughout the trial.

9.5.1 Pilot Phase

The Pilot Phase randomises patients between all the study arms so that the results from these patients can be included in the main trial. Feasibility is considered in terms of the acceptability of the trial randomisation and reported toxicities and adherence to trial medication. Centres participating in the Pilot Phase will be required to keep an anonymised log of all patients assessed for trial eligibility. We will then summarise the number of patients who do not

participate in the study, and also the number of eligible patients who choose to not participate in the study (reasons for non-participation will be collected where the patients are willing). On the patients who are randomised, we shall describe the incidence of expected and unexpected severe toxicities and adverse events/reactions (see Section 11) to decide whether to continue with research arms beyond the Pilot Phase. As indicated above, we do not anticipate that recruitment to the research arms will be discontinued after the Pilot Phase, as there is considerable experience with zoledronic acid and docetaxel when combined with HT, while Cox-2 inhibitors generally have a good toxicity profile. Although there are limited data on the combinations, we do not expect severe toxicity.

9.5.2 Activity and Efficacy Stages

The approach to analysis of these stages is summarised within the sample size calculations (see Section 9.3.3). Each research arm will be compared in a pairwise fashion against the control arm.

10 MONITORING & QUALITY ASSURANCE

10.1 MONITORING AT MRC CTU

Data provided to the MRC CTU will be checked for missing or unusual values (range checks) and consistency over time. If missing or questionable data are identified, staff at the MRC CTU will request that the data be clarified. The exact procedures for data clarification and the amendment of CRFs will be described in the trial specific SOPs and instructions will be sent to all STAMPEDE institutions as soon as they have been approved to participate in the trial. The MRC CTU will also send reminders for any overdue data.

10.2 DIRECT ACCESS TO DATA

Collaborating institutions should be aware that direct access to patient data by MRC CTU staff may be required for trial-related monitoring or audit. Patient consent for this will be obtained as part of the general trial consent process.

10.3 VISITS TO INVESTIGATOR SITES

A selection of institutions will be visited at least once during the course of the STAMPEDE trial. The MRC CTU will give the responsible investigator adequate notice of the monitoring visit to allow adequate time, space and staff for these visits. The standard operating procedures (SOP) for monitoring are available from the MRC CTU.

After the monitoring visit the monitor will complete a site visit report. This report will be circulated to the TMG for comment. Once the TMG have reviewed the report and agreed on any recommendations the monitor will finalise the report and send a copy to the Principal Investigator (PI) at the site. A copy will also be sent to the CI for the trial and another copy will be kept in the MRC CTU STAMPEDE trial master file.

10.4 CONFIDENTIALITY

All information collected during the course of the research will be kept strictly confidential. In addition, all procedures for handling, processing, storage and destruction of data are compliant with the Data Protection Act 1998. No individual patients will be identified when the results of the trial are published.

Patients will be asked for permission for information about their health status to be obtained from the Office of National Statistics (ONS) or via the NHS Strategic Tracing Service or similar by the Medical Research Council, if necessary. In addition, patients will be asked for permission to inform their GP of their involvement in the STAMPEDE trial.

11 SAFETY REPORTING

ICH GCP requires that both investigators and sponsors follow specific procedures when reporting adverse events/reactions in clinical trials. These procedures are described in this section of the protocol.

11.1 DEFINITIONS

The definitions from ICH GCP apply in this trial protocol. These definitions are given in table 4.

Table 4: Event Terms and Definitions

Term	Definition
**Adverse Event (AE)**	Any untoward medical occurrence in a patient or clinical trial subject to whom a medicinal product has been administered including occurrences which are not necessarily caused by or related to that product.
**Adverse Reaction (AR)**	Any untoward and unintended response to an investigational medicinal product related to any dose administered.
**Unexpected Adverse Reaction (UAR)**	An adverse reaction, the nature or severity of which is not consistent with the information about the medicinal product in question set out in the summary of product characteristics (or Investigator brochure) for that product.
**Serious Adverse Event (SAE) or Serious Adverse Reaction (SAR) or Suspected Unexpected Serious Adverse Reaction (SUSAR)**	Respectively any adverse event, adverse reaction or unexpected adverse reaction that:<br>• results in death<br>• is life-threatening*<br>• requires hospitalisation or prolongation of existing hospitalisation**<br>• results in persistent or significant disability or incapacity<br>• consists of a congenital anomaly or birth defect<br>• Other important medical condition

Clarifications and Exceptions

*The term 'life-threatening' in the definition of 'serious' refers to an event in which the patient was at risk of death at the time of the event; it does not refer to an event which hypothetically might have caused death if it were more severe.

**Hospitalisation is defined as an inpatient admission, regardless of length of stay, even if the hospitalisation is a precautionary measure for continued observation. Hospitalisations for a pre-existing condition (including elective procedures that have not worsened) do not constitute an SAE.

Medical judgement should be exercised in deciding whether an AE/AR is serious in other situations. Important AE/ARs that are not immediately life-threatening or do not result in death or hospitalisation but may jeopardise the subject or may require intervention to prevent one of the other outcomes listed in the definition above, should also be considered serious.

Pregnancy occurring in a STAMPEDE patient's partner during the patient's participation in the trial, must be reported to the MRC CTU within the same timelines as an SAE and classified as an 'other important medical condition' on the SAE form. The outcome of a pregnancy should be followed up carefully and any abnormal outcome to the mother or child should be reported.

11.1.1 Trial-Specific Exemptions

Disease progression or death as a result of disease progression are not considered to be SAEs and should be reported on the STAMPEDE Progression Form or Death Form.

The following situations that fulfill the definition of an SAE are excluded from expedited notification on an SAE form and should be reported only on the STAMPEDE follow-up form

- Elective hospitalisation and surgery for treatment of locally advanced or metastatic prostate cancer or its complications
- Elective hospitalisation to simplify treatment or procedures
- Elective hospitalisation for pre-existing conditions that have not been exacerbated by trial treatment

11.2 INSTITUTION / INVESTIGATOR RESPONSIBILITIES

All non-serious AEs/ARs, whether expected or not, should be recorded in the toxicity (symptoms) section of the Follow up CRF and sent to the MRC CTU within one month of the form being due. SAEs/SARs should be notified to the MRC CTU as described below.

The severity (i.e. intensity) of all AEs/ARs (serious and non-serious) in this trial should be should be graded using Common Terminology Criteria for Adverse Events (CTCAE) v3.0 (http://ctep.cancer.gov/reporting/index.html). A flowchart is given in **Appendix O** to help explain the notification procedures. Any questions concerning this process should be directed to the MRC CTU in the first instance.

11.2.1 Investigator Assessment

(a) Seriousness

When an AE/AR occurs the investigator responsible for the care of the patient must first assess whether the event is **serious** using the definitions given in Table 4. If the event is serious and not exempt from expedited reporting, then an SAE form must be completed and the MRC CTU notified.

(b) Causality

The Investigator must assess the causality of all serious events/reactions in relation to the trial therapy using the definitions in Table 5. There are 5 categories: unrelated, unlikely, possible, probable and definitely related. If the causality assessment is unrelated or unlikely to be related the event is classified as a SAE. If the causality is assessed as either possible, probable or definitely related then the event is classified as a SAR.

Table 5: Definitions of causality

Relationship	Description	Event Type
**Unrelated**	There is no evidence of any causal relationship	SAE
**Unlikely**	There is little evidence to suggest there is a causal relationship (e.g. the event did not occur within a reasonable time after administration of the trial medication). There is another reasonable explanation for the event (e.g. the patient's clinical condition, other concomitant treatment).	SAE
**Possible**	There is some evidence to suggest a causal relationship (e.g. because the event occurs within a reasonable time after administration of the trial medication). However, the influence of other factors may have contributed to the event (e.g. the patient's clinical condition, other concomitant treatments).	SAR
**Probable**	There is evidence to suggest a causal relationship and the influence of other factors is unlikely.	SAR
**Definitely**	There is clear evidence to suggest a causal relationship and other possible contributing factors can be ruled out.	SAR

(c) Expectedness

If the event is a SAR the Investigator must assess the expectedness of the event. Please see Appendix G (table G.2) for a list of expected toxicities associated with the drugs being used in this trial. If a SAR is assessed as being unexpected it becomes a SUSAR.

(d) Notification

Investigators must notify the MRC CTU of all SAEs occurring from the time of randomisation until 30 days after the last protocol treatment administration. SARs and SUSARs must be notified to the MRC CTU indefinitely (i.e. no matter when they occur after randomisation).

11.2.2 Notification Procedure:

1. The SAE form must be completed by the Investigator (consultant named on the signature list and delegation of responsibilities log who is responsible for the patient's care), with due care being paid to the grading, causality and expectedness of the event as outlined above. In the absence of the responsible investigator the form should be completed and signed by a member of the site trial team. The responsible investigator should subsequently check the SAE form, make changes as appropriate, sign and then re-fax to the MRC CTU as soon as possible. The initial report shall be followed by detailed, written reports as appropriate.

2. Send the SAE form by fax to the MRC CTU. **Fax Number: + 44 (0) 20 7670 4818**

3. Follow-up: Patients must be followed-up until clinical recovery is complete and laboratory results have returned to normal or baseline, or until the event has stabilised. Follow-up should continue after completion of protocol treatment if necessary. Follow-up information should be noted on a further SAE form by ticking the box marked 'follow-up' and faxing to

the MRC CTU as information becomes available. Extra, annotated information and/or copies of test results may be provided separately. The patient **must** be identified by trial number, date of birth and initials only. The patient's name **should not** be used on any correspondence.

11.3 MRC CTU RESPONSIBILITIES

Medically qualified staff at the MRC CTU and/or the Chief Investigator (or a medically qualified delegate) will review all SAE reports received. The causality assessment given by the local Investigator at the hospital cannot be overruled and in the case of disagreement, both opinions will be provided in any subsequent reports.

The MRC CTU is undertaking the duties of trial sponsor and is responsible for the reporting of SUSARs and other SARs to the regulatory authorities (MHRA and competent authorities of other European member states and any other countries in which the trial is taking place) and the research ethics committees as appropriate.

The MRC CTU will also keep all investigators informed of any safety issues that arise during the course of the trial.

SAE REPORTING

Fax to 020 7670 4744 within 24 hours of becoming aware of the event

12 ETHICAL CONSIDERATIONS AND APPROVAL

12.1 ETHICAL CONSIDERATIONS

This is a randomised trial therefore neither the patients nor their physicians will be able to choose the patients' treatment. Treatment will be allocated randomly using a computer-based algorithm. This is to ensure that the groups of patients receiving each of the different treatments are similar.

Patients will be randomised to one or two of the newer treatments in combination with hormone treatment or hormone treatment alone which is the standard treatment for these forms of prostate cancer. Twice as many patients will be randomised to the standard treatment as each of the arms containing the newer combinations of treatments. These newer combined treatment options are being assessed in a detailed and systematic fashion in this trial. There is some evidence to suggest that the newer treatment options *may* have advantages over standard treatment (hormone therapy) alone with regards clinical outcome, but this is not confirmed and toxicity may be increased. This trial will follow a large group of men who have been randomly allocated to either the standard treatment (hormone therapy alone) or the newer combined treatment options in order to measure the benefits of the new treatments. The patients will also be followed-up for toxicity and safety issues, so that any benefits can be weighed against any negative aspects.

Patients participating in the trial will have some additional hospital visits and some extra blood samples taken compared to patients who are not participating in the trial. Sometimes the blood samples can be taken when the patient is attending hospital for treatment, anyway. On some of the trial arms, the patient may have to make additional visits to the hospital for the blood sample to be taken, although in some cases it may be possible for the blood sample to be taken in the GP's surgery or in the patients' home. The additional visits and blood samples are to ensure that follow-up of patients is comparable in all the treatment groups. The blood samples will also be used for genetic and serum marker studies, where this information will be considered with clinical data. Blood samples will be link-anonymised. There will be no feedback to individual patients.

If new information emerges during the course of the trial which may affect the treatment or follow-up of patients who have jointed the trial, information will be provided through the study doctor.

12.2 ETHICAL APPROVAL

The protocol has a Favourable Opinion from a Main Research Ethics Committee (MREC), but each site must also must obtain management permission for research (R&D approval) from the relevant NHS host organisations before patients can be entered into the trial. The patient's informed consent to participate in the trial should be obtained after a full explanation has been given of the treatment options, including the conventional and generally accepted methods of treatment. Patient information sheets and patient consent forms are given in **Appendix B**.

The right of the patient to refuse to participate in the trial without giving reasons must be respected. After the patient has entered the trial, the clinician must remain free to give alternative treatment to that specified in the protocol, at any stage, if he feels it to be in the best interest of the patient. However, the reason for doing so should be recorded and the patient will remain within the trial for the purpose of follow-up and data analysis according to the treatment option to which he has been allocated. Similarly, the patient must remain free to withdraw at any time from the protocol treatment without giving reasons and without prejudicing his further treatment.

A statement of MRC policy on ethical considerations in clinical trials of cancer therapy, including the question of informed consent, is available from the MRC Head Office web site (http://www.mrc.ac.uk).

13 REGULATORY APPROVAL

This trial has been approved by the MHRA and will be conducted under a CTA (Ref: 00316/0026/001-0001) in the UK and equivalent in other countries.

14 INDEMNITY

The MRC and NHS are both publicly funded bodies and are not allowed to purchase advance insurance to cover indemnity because they are backed by the resources of the Treasury.

The MRC will give sympathetic consideration to claims for non-negligent harm suffered by a person as a result of trial or other work supported by MRC. This does not extend to liability for non-negligent harm arising from conventional treatment where this is one arm of a trial. The MRC acts as its own insurer and does not provide cover for non-negligent harm in advance for participants in MRC-funded studies.

Where studies are carried out in a hospital, the hospital continues to have a duty of care to a patient being treated within the hospital, whether or not the patient is participating in an MRC-supported study. MRC does not accept liability for any breach in the hospital's duty of care, or any negligence on the part of employees of hospitals. This applies whether the hospital is a NHS Trust or not.

15 FINANCE

STAMPEDE is funded by the Clinical Trials Advisory Awards Committee (CTAAC) (on behalf of Cancer Research UK, Medical Research Council, and other charities). The trial has National Cancer Research Network (NCRN) approval and, therefore, local NCRN funds may be available at each centre to support entry of patients into this trial.

Zoledronic acid is manufactured by Novartis. Novartis have agreed to provide an educational grant to support the conduct of this study. Novartis have also agreed to supply the study drug, zoledronic acid free of charge for patients participating in the study.

Docetaxel is manufactured by Aventis Pharma. They have agreed to supply the study drug, docetaxel at a discounted rate for patients that are participating in the trial and to provide an educational grant to support the conduct of the study. The Department of Health has agreed to provide a central subvention as follow: £1,787 per patient randomised to arms C and E of the trial and prescribed docetaxel. This amount is payable in respect of a hospital trust randomising more than 3 patients. For more details contact the STAMPEDE Trial Manager.

Celecoxib is manufactured by Pfizer. They agreed to supply free drug and provide funds to distribute drug to participating sites.

16 TRIAL COMMITTEES

16.1 TRIAL MANAGEMENT GROUP (TMG)

A Trial Management Group (TMG) has been formed comprising the Chief Investigator, other co-investigators and members of the MRC CTU. The membership of the TMG may be expanded if other groups of trialists wish to participate. The TMG will be responsible for the day-to-day running and management of the trial and will meet by teleconference at least 3 monthly and in person as needed. The TMG members are detailed in **Appendix L**.

16.2 TRIAL STEERING COMMITTEE (TSC)

A Trial Steering Committee (TSC) will be formed to provide overall supervision for the trial and provide advice through its independent chair. The ultimate decision for the continuation of the trial lies with the TSC. The TSC will meet twice a year.

16.3 INDEPENDENT DATA MONITORING COMMITTEE (IDMC)

An Independent Data Monitoring Committee (IDMC) will be formed. The IDMC will be the only group who sees the confidential, accumulating data to the trial. Reports to the IDMC will be produced by the MRC CTU. The IDMC will meet within 6 months of the trial opening with the frequency of meetings dictated by the IDMC. The IDMC will consider data in accordance with the analysis plan (see section 9.5) and will be advisory to the TSC. The IDMC can recommend premature closure or reporting of the trial, or that recruitment to any research arm be discontinued.

From version 7.0 onwards, any recommendation from the IDMC to stop recruitment to one or more trial arms will be acted upon immediately, pending ratification from the TSC. As this period between meetings should be very short, sites would not be notified until after the TSC have made a decision. IDMC recommendations based on emerging safety issues would be discussed with sites promptly.

Further details of IDMC functioning and the procedures for interim analysis and monitoring are provided in the IDMC charter (available on request).

Figure 6 - Diagram of relationships between trial committees

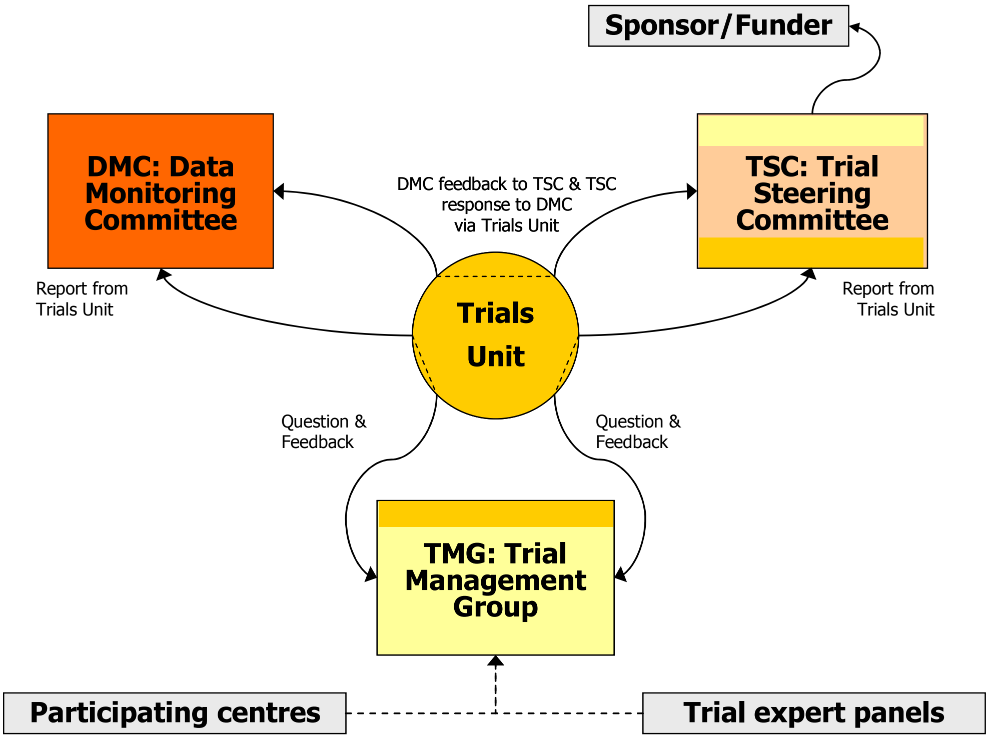

17 ANCILLARY STUDIES

17.1 QUALITY OF LIFE

A quality of life (QL) study is being performed to assess the impact of each treatment arm on the quality of patient's lives and participation in this study has been limited to the first 700 patients recruited (this was reached in September 2008) patients recruited after this point will not be asked to join the QL study. The EORTC QLQ-C30 with the prostate-specific module QLQ PR25 will be used. Key items for assessment are pain reduction for patients with metastatic disease and urinary symptoms for patients with locally advanced disease. In addition specific hypotheses will be generated for each of the research arms. The EuroQol (EQ-5D) (42) will be used in the study as a generic measure of health-related quality of life which can be linked to public preferences. These data will be used to calculate quality-adjusted life-years as part of the economic evaluation (see Section 17.2).Patients who were recruited within the first 700 and who started the QL study, should continue on the study throughout the trial. Questionnaires should be self-administered, although it is recommended that a key person (e.g. research nurse) at each centre be responsible for the data collection to optimise compliance and completeness of the data.

The QL and the HE questionnaires should be completed without conferring with friends or relatives and all questions should be answered even if the patient feels them to be irrelevant.

The responsible person should check each questionnaire for its completeness, ensuring that the correct date of completion and patient identifiers are present. The research nurse should approach patients at appropriate clinical visits to complete a questionnaire. If no clinical visit is scheduled for the patient (with a window of 4 weeks around the expected date) the nurse should organise the completion of the questionnaire, by post or by a visit to the patient at home (or in a hospice).

17.2 HEALTH ECONOMICS

A health economics (HE) sub-study will be performed. Core resource use information will be collected, using CRFs on days in hospital (by speciality) and outpatient visits. Data being collected on concomitant medication will also be used in the economic analysis. Information on patients' use of primary care and community-based services will be collected as additional questions in the QL questionnaire. Costs will be calculated on the basis of representative UK unit costs at the point of analysis. Health outcomes will be assessed in terms of quality-adjusted life years (QALYs). Quality adjustments will be based on patients' responses to the EQ-5D health status measure which will be administered at baseline and each point of follow-up as part of the QL questionnaire. A cost-effectiveness analysis will compare all regimens in Activity Stage II.

17.3 TRANSLATIONAL SUB-STUDIES

17.3.1 DNA Analysis

Blood samples from as many patients as possible will be collected for future research. With patient consent, an additional droplet of blood sample will be collected and stored for DNA and protein analysis in order to try to identify molecular features of clinical significance.

Blood samples should be sent directly to the central laboratory on the FTA elute cards provided. Patient information sheets and consent forms which highlight this research are given in **Appendix B**, while details of specimen collection, posting and contact details are given in **Appendix D**.

17.3.2 Tissue Microarray

Patient consent will be sought to utilise paraffin embedded tissue for the construction of tissue microarrays from needle cores. One needle biopsy will be selected for microarray and the remaining tissue will be returned to the originating histopathologist. Given the entry criteria for the trial, the majority of patients will have extensive disease in the diagnostic needle core biopsies, in contrast to men with localised, low grade disease. Consequently, removal of one core is unlikely to compromise any subsequent histopathological assessment. Details regarding transfer of samples will be issued at the time of construction of the micro array.

17.3.3 Bone Mineral Density

In selected centres, patients will be asked to participate in a bone mineral density sub-study. The aim of this sub-study will be to evaluate the long-term effects of the treatments used in STAMPEDE on bone health. Further details in regard to this study can be found in the Patient information sheets and consent forms (different versions for use when a centre is participating in the Bone Mineral Study) in **Appendix B** and in the Bone Mineral Density sub-protocol in **Appendix Q.**

In addition, a number of sub-studies are being proposed using blood or urine samples from selected centres. We will ask patients to consent to the collection of material on the basis that separate Ethical Approval will be obtained for each sub study prior to any use of the samples.

18 PUBLICATION

The results from different centres will be analysed together and published as soon as possible. Individual clinicians must not publish data concerning their patients that are directly relevant to questions posed by the study until the TMG has published its report. The TMG together with the STAMPEDE collaborators will form the basis of the writing committee and decide on the nature of publications. The main publication will be in the name of the STAMPEDE Trial Collaborators and the writing committee will be listed at the end of the manuscript. All publications will acknowledge the participating centres and clinicians, and these will be detailed in an appendix to the main report.

19 PROTOCOL AMENDMENTS

19.1 PROTOCOL

19.1.1 Amendments made to sections in Protocol version 1.0 May 2004

1. Administrative changes such as typos, word change etc.
2. Name additions/changes to:
   - TMG members
   - TSC members
   - IDMC members
3. 'General Information' Section – additional information re. Abridged version of protocol
4. Section 1.2 – Figure 1, Celecoxib duration amended
5. Section 1.3 – Figure 2, addition of cardiovascular assessment form, name and timings amended
6. Section 2.3 – Docetaxel information updated
7. Section 2.4 – Additional text re dose and duration justification for Celecoxib use.
8. Section 3 – Title change and content updated
9. Section 4.2 – New exclusion criteria added
10. Section 4.3.1 – New investigations added and additional text re testosterone measurements and additional text re. prior celecoxib treatment
11. Section 6.1.4 – Celecoxib duration amended
12. Section 6.1.5 – Additional text re. Co-administration of docetaxel and bisphosphonates
13. Section 6.1.6 – Celecoxib duration amended
14. Section 6.2.2 – additional docetaxel information
15. Section 6.2.3 – addition of CV event history
16. Section 11 – Safety reporting updated
17. Section 12.1 – Additional text re. the collection of blood for genetic and serum marker studies
18. Section 15 – Additional information re. Central Subvention for docetaxel arms

19.1.2 Amendments made to sections in Protocol version 1.1 May 2005

1. Section 6.2 Administration and Dose Modifications, subsection 6.2.1 Zoledronic Acid

19.1.3 Amendments made to sections in Protocol version 2.0, 08 June 2005

1. General Information section – SAE reporting fax number and timeframe added.
2. Section 1.2 – Addition of anti-androgen use for M0 patients as a method of HT

3. Section 1.2 – Increase in amount of blood needed & addition tissue sample request.
4. Section 1.3 Trial Documentation updated to include new table detailing trial documentation ahead of accreditation, the inclusion of the radiotherapy forms and correct case report form timings
5. Section 2.1 – Addition of anti-androgen use for M0 patients as a method of HT
6. Section 4.1.3 – Inclusion criteria Vii "Normal testosterone prior to hormone treatment" removed.
7. Section 4.1.3 - φnote has been omitted and moved to section 4.2 (see number 8)
8. Section 4.2 – Exclusion criteria added to exclude patients with active peptic ulceration, gastrointestinal bleeding and inflammatory bowel disease.
9. Section 4.2 – Exclusion Criteria added to exclude patients with planned major dental work
10. Section 4.3.1 - All blood test timelines changed from 14 days to 28 days.
11. Section 4.3.1 – Hormone Therapy pre-randomisation deadline extended from 4 weeks to 12 weeks.
12. Section 4.3.1 – Additional information regarding the use of NSAIDs and cox-2-inhibitors before coming on to the STAMPEDE study and once commenced on study treatment
13. Section 4.3.2 – Updated to ask for all vitamins and minerals the patient is taking to be recorded.
14. Section 4.3.3 – Updated to include the extra blood required and the request for consent of patients' tissue samples.
15. Section 6.1.1 – Addition of anti-androgen use for M0 patients as a method of HT
16. Section 6.1.6 – Addition of the calcium & vitamin name "calcichew".
17. Section 6.6.2 – asking also to collect vitamins and minerals under concomitant medication.
18. Section 6.6.3 – New section to inform investigators that patient's, who they wish to give radiotherapy to, are also eligible for STAMPEDE
19. Section 6.6.4 – New section to detail what data is being collected on the radiotherapy given to patients.
20. Section 7.1; figure 4 – Addition of radiotherapy form and in note, addition of AA alone
21. Section 7.1.2 – omission of repeated scans and x-rays at 24 weeks, also omitted in note under figure 4.
22. Chapter 11 – Safety reporting section updated
23. Section 17.3 – Increase in amount of blood needed & additional tissue sample request.

19.1.4 Amendments made to section in protocol version 3.0 July 2006.

Front Cover - NCRN logo added for accuracy

Front Cover - Clafication that protocol developed with NCRI rather than on behalf of

Front Cover - Clarification the it is a 6 arm trial

General Information section - MRC CTU staff section updated

Section 1.2 – Statistics section updated.

Section 1.2 - Additional research paragragh updated to refelct additional studies and for clarification of terms

Section 1.2 - Blood collection volume changed to reflect new technique used

Section 1.3 (figure 3) - Table showing case report form scheduyle updated to reflect clarification of follow up schedule and addition of new CRF (End of Treatment)

Section 2.2 - AS changed to HT (clarification of terms)

Section 2.3 - Updated in information in reagrd to use of docetaxel added to reflect up to date practice

Section 2.5 - Sub-headings numbered for consistancy

Section 3.0 - Information in reagrd to the Pilot Phase now written in past tense as Pilot Phase has now been completed

Section 4.1.1 - Inclusion criteria extended so that patinets who fulfil 2 out of the three of the first inclusion criteria can be eligible.

Section 4.3.1 - Change in time scales by which baseline investigations need to be completed.

Section 4.3.1 - Clarification that chest X-ray is only required if chest is not included in the CT

Section 4.3.1 - Removal of 12 week timeline for basline PSA test ot be performed. (Stipulation that it must be performed before start of HT)

Section 4.3.2 – Information added in regard to time allowed from randomisation to start of treatment

Section 4.3.3 - Additional research paragragh updated to refelct additional studies and for clarification of terms

Section 4.3.3 - Blood collection volume changed to reflect new technique used

Sections 6.1.2-6.1.6 - Androgen Supression replaced with Hormone Therapy for consistemcy of terms

Section 6.2.2 - '(Taxotere)' Removed for consistancy

Section 6.2.2 _ information added in regard to the need to closely monitor liver function prior to docetaxel adminstration

Section 7.1 - Page number reference updated

Section 7.1.1 - PSA measurement timings updated to accurately reflect follow up schedule

Section 7.3 (Table 4) - Table and key updated to accurately refelct follow up schedule and to include information about new CRFs and removal of withdrawal CRF

Section 8 - Rewording for clarifcation of definition of trial withdrawal

Section 8.1 - Instruction that withdrawal from trial treatment should be recorded on End of Treatment Form rather than withdrawal form

Section 8.1 - Information updated to emphasise that trial treatment must be discontinued following a progression

Section 8.2- Information added in regard to patient transfers

Section 8.3 - Instruction that withdrawal from trial completely must be notified in writing to the MRC CTU rather than included on withdrawal form

Section 9 and Summary – Target event numbers updated to reflect the slightly revised numbers obtained by using –nstage- which is the new, recommended program for MAMS trials
Sections 11.1 and 11.2 - Form numbers removed to allow for future changes in numbering
Section 11.2 – Reference to toxicity grading website added
Section 11.2.1 - Reference to table in appendix G added
Section 12.2 - 'Suggested' removed from 'Suggested patient information sheets'
Section 13 - CTA reference added
Section 17.3 - Information added to reflect new blood collection method for DNA analysis and in regard to additional translational studies for which funding has recently been approved

19.1.5 Amendments made to protocol version 4.0 Dec 2007

General Information Section - Randomisation and SAE reporting details sections clarified
Section 1.2 and throughout protocol - Efficacy Stages 1-111 renamed to Activity Stages 1-111 for accuracy and clarity
Section 1.2 - Follow schedule corrected
Section 4.1.2 - Inclusion criteria widened to include high risk relapsing patients, that would not have met the previous PSA based criteria
Section 4.1.3 - Note added to reference location of WHO performance status definitions
Section 4.2 - Notes added to reference locations of toxicity gradings and NYHA classifications
Section 4.3.1 - Timings of baseline scan information changed to accurately reflect most common current practice
Section 6.1.1 - Information about use of LHRH antagonists to ensure that the protocol accurately reflects current and future practice
Section 6.1.1 - Information about suggested duration of hormone therapy added to ensure that the protocol accurately reflects current practice
Section 6.2.2 - Additional information added about the timing of liver function tests prior to docetaxel administration added for clarity
Section 6.6.4 - Information on radiotherapy data collection added
Section 7.1.1 - Erroneous information about the timing of PSA measurements removed
Figure 3 - Moved to new section in protocol for clarity and extended to include current information on data collection
Figure 3b - Added to describe how extent of data collection during follow up should change, post treatment and post progression
Figure 4 - Notes added to explain the changes in data collected at follow up and to information that the quality of life study will be applicable to the first 700 patients randomised only
Figure 4 - Note added to include palliative radiotherapy CRF
Section 11.3 - SAE reporting information updated
Section 19 - Protocol amendments list updated

19.1.6 Amendments made to protocol version 5.0 Aug 2008

1. General Information Section – Randomisation phone line number updated – non UK extension added
2. Section 3 – Information about QL study removed to reflect closure of QL study after first 700 patients
3. Section 4.2 – Exclusion criteria clarified to explain that only patients with severe poor cardiovascular history should be excluded
4. Section 4.3.1 – Information on co-administration of NSAIDS with celecoxib changed based on clinical advice.
5. Section 5 - Randomisation phone line number updated – non UK extension added
6. Section 6.2.1. – Information added to clarify that patients who develop an osteonecrosis of the jaw should stop zoledronic acid treatment
7. Section 6.2.3 – 'severe' text added to acurately reflect which patients should be excluded based on their cardiovascular history
8. Section 7.1.2 – Defination of disease progression extended for clarity
9. Figure 3 – Updated to include reference to newly created skeltal related event form
10. Figure 4 – Previous error in table amended to show that the 4th Zoledronic Acid form that is submitted contains information about 3 cycles rather than 2 as previously indicated
11. Table 4 – 'Other important medical condition' added to definition of serious in the SAE section, to accuratly reflect SAE form and current practice
12. Section 11.1 – Information added on reporting or pregnancies
13. Section 17 - Information about QL study removed to reflect closure of QL study after first 700 patients

19.1.7 Amendments made to protocol version 6.0 Jul 2009

1. General Information Section – Trial Pharmacist removed and changes of:
    - Co-Investigator
    - Patient Representatives
    - Trial Manager
    - Data Manager
2. General Information Section - Coordinating Centre – address change
3. General Information Section – change of Sponsor address
4. Section 1.1 – ratio of patients randomised to the investigational arms updated
5. Section 1.2 – figure 1b added to clarify trial design from April 2011 onwards
6. Section 1.2 – paragraph added to explain trial changes after the second activity analysis
7. Section 1.2 – wording added to clarify that QL data only collected for first 700 patients randomised
8. Section 1.3 – SSA Favourable Opinion removed from list of trial documentation required ahead of site accreditation

9. Section 2.1 – Amount of men diagnosed with prostate cancer annually updated
10. Section 2.4 –note added to explain completion of recruitment to celecoxib- containing arms
11. Section 2.5.2 - note added to explain completion of recruitment to celecoxib- containing arms
12. Section 3 – SSA Favourable Opinion removed
13. Section 4.2 – Exclusion criterion xiii greyed out
14. Section 4.3.1 – paragraph removed regarding potential randomisation to celecoxib-containing arms
15. Section 5 – Randomisation instructions expanded to exclude public holidays or dates when notice has been given by the CTU
16. Section 6.1.4 – formatting changed to grey font to reflect recruitment completion for arm D
17. Section 6.1.6 - formatting changed to grey font to reflect recruitment completion for arm F
18. Section 6.2.3 – recruitment note added
19. Section 6.6.3 – radiotherapy statement changed to reflect data from recent trials
20. Section 7.1.2 – removal of reference to SRE- specific CRF
21. Section 7.3 – Figure 3 - Addition of Bone Density Risk Factor Form and BMD sub-study assessment forms to summary of timing table
22. Section 7.3 – Figure 4 – Weeks added to timings of assessments post 2 years
23. Section 7.3- Figure 4 – note added to explain recruitment completion for arms D and F
24. Section 12.1 – Wording changed to reflect change to randomisation allocation ratio
25. Section 12.1 – Addition of statement regarding new information emerging during the trial
26. Section 12.2 – Reference to SSA removed
27. Section 16.3 – Statement added regarding actioning IDMC recommendation ahead of TSC ratification

19.2 APPENDICES

19.2.1 Amendments made to Appendices version 1.0 May 2004

1. Appendix A – Addition of NYHA classifications
2. Appendix B General PIS - Docetaxel information updated, additional information explaining cardiovascular risk related to celecoxib and celecoxib duration amended
3. Appendix B PIS C – Docetaxel information updated
4. Appendix B PIS D – Additional information explaining cardiovascular risk related to celecoxib and celecoxib duration amended
5. Appendix B PIS E – Docetaxel information updated
6. Appendix B PIS F – Additional information explaining cardiovascular risk related to celecoxib and celecoxib duration amended

7. Appendix C GP letter – product name changed from Celebrex to Onsenal
8. Appendix E – Drug supply/ordering procedures amended
9. Appendix F 'Administration of Zoledronic acid' – updated dose modification section
10. Appendix F – Additional section added 'Co-administration of docetaxel + zoledronic acid'
11. Appendix G Celecoxib – updated from revised celecoxib SPC
12. Appendix G: Table G.2 - Comparative table of undesirable effects of docetaxel, Zoledronic Acid and Celecoxib – updated information from revised celecoxib SPC
13. Appendix J: Common Toxicity Criteria – additional clarification and change in table structure
14. Appendix M: Accreditation documents – addition of new documents
15. Appendix P – Assessing and notifying CTU of adverse events flow diagram added

19.2.2 Amendments made to Appendices version 1.1 May 2005

1. Appendix F 'Administration of Zoledronic acid' – updated section on recommend dose reduction in patients with mild to moderate kidney dysfunction (defined as a creatinine clearance of 30-60ml/min) who are receiving zoledronic acid.
2. Appendix G - Drug Safety Information for Drugs used in the Trial; section on zoledronic acid. Additional text informing clinicians that rare cases of Osteocronosis (primarily of the jaws) have been reported in patients treated with bisphosphonates.

19.2.3 Amendments made to Appendices version 2.0, 08 June 2005

24. Appendix B Patient information sheets & consent form – Increase in amount of blood needed & additional tissue sample request.
1. Appendix B General PIS – Addition of the use of bicalutamide alone as a Hormone treatment.
2. Appendix B General PIS – paragraph detailing to patients that they may receive radiotherapy along side their allocated treatment in STAMPEDE.
3. Appendix B General PIS – Side effects of osteoporosis added to "What are the side effects"
4. Appendix B General PIS – Will my taking part be kept confidential – now asking for NHS number and mention of NHS Strategic Tracing Service.
5. Appendix B STAMPEDE Additional Research – Increase in amount of blood needed & addition tissue sample request.
6. Appendix B Consent form – additional consent asked for urine and tumour samples, name to be stored on computer database and an extra copy of consent form to be taken and stored at the MRC CTU.
7. PIS A, B, C, D, E & F – addition of information about side effects of osteoporosis

8. PIS A, B, C, D, E & F – Addition of bicalutamide alone as an option for hormone therapy for M0 patients
9. PIS B, E & F – New side effects added, kidney failure and Osteoncrosis of the jaw
10. Appendix E: drug ordering - number of bottles of celecoxib sent to pharmacies changed from 15 to 24
11. Addition of Appendix P – Detailing radiotherapy guidelines for investigators.

19.2.4 Amendments made to appendices version 3.0 July 2006

Appendix B - Patient Information Sheets (PIS) & Consent Forms - all translational additional now research refered to as 'translational sub-studies'

Appendix B – Information added that there will be different additional research PIS and consent forms for centres taking part in the bone mineral density study (BMD) compared with those that are not

Appendix B All PIS - 'family doctor' replaced by GP to ensure consistancy of terms

Appendix B General PIS section 12 – Information about collection of patinet names re-worded for clarity

Appendix B Additional Research PIS – Type A (for centres NOT participating in the BMD study) and Type B (for centres that are participating in the BMD study) created

Appendix B Additional Research PIS section 2 - Informatin on when to complete questionanaires updated to accuarely reflect follow up schedule

Appendix B Additional Research PIS - Information added to reflect new blood collection method for DNA analysis and in reagrd to additional translational studies for which funding has recently been approved

Appendix B Consent Form - Type A (for centres NOT participating in the BMD study) and Type B (for centres that are participating in the BMD study) created

Appendix B Consent form - Information changed in section L in regard to volume of blood sample required to refeclt new collection practice

Appendix B Consent Form – Information about allowing consent form to be sent to MRC CTU and GP, moved to the optionl section

Appendix B All Arm Specific PIS – Wording chaned in HT section to more clearly explain how different treatments may effect boen in different ways.

Appendix C GP letter - 'study' rplaced by 'trial' and 'androgen supression' replaved by 'hormone therapy' to ensure consistency of terms

Appendix C GP letter – Specification on number of calcichew tablets removed to allow for variations in local practice

Appendix D - DNA analysis sub-study sample collection information changed to reflect new technique (FTA elute cards) being used for blood collection

Appendix E – Specificity on the contents of the drug starter pack removed to allow for changes in the future

Appendix F - Clarification that all docetaxel treatment delays should be reported on the CRF.
Appendix F – Information added in regard to the need to closely monitor liver function prior to administration of docetaxel
Appendix L - MRC CTU staff upadted and IDMC members updated
Appendix M - Investigator Statement updated to reflect current version
Appendix P - 'AS' replaced with 'HT' to ensure consistancy of terms
Appendix P - Description of hormone therapy clarified
Appendix P – Recommended dose of radiotherapy amended to better reflect current practice
Appendix Q – New appendix added to give information in regard to BMD study.

19.2.4 Amendments made to appendices version 4.0 Dec 2007

Appendices spilt for main protocol for ease of use
Appendix B - General PIS - Information about osteonecrosis of the jaw risk added to zoledronic acid section
Appendix B - All arm specific PIS - Information about LHRH antagonists added to the horome therapy section
Appendix K - Instructions in regard to confirming biochemical failure amended
Appendix L - Trial Steering Committee contact details updated

19.2.5 Amendments made to appendices verion 5.0 Aug 2008

Appendix B – Guidance for administrators – QL Information removed
Appendix B – General PIS – QL information removed
Appendix B – Additional Research PIS (type A) – QL Information removed
Appendix B – Additional Research PIS (type B) – QL Information removed
Appendix B – Consent form (type A) – QL Information removed
Appendix B – Consent form (type B) – QL Information removed
Appendix F - Information added to clarify that patients who develop an osteonecrosis of the jaw should stop zoledronic acid treatment
Appendix L – TMG Contact details updated

19.2.6 Amendments made to appendices version 6.0 Jul 2009

Appendix B – patient information sheets for arms D and F greyed out
Appendix B – General PIS updated to v7.0
Appendix C - GP letter updated to v7.0
Appendix E – reference to SSA favourable assessment removed
Appendix L – trial contacts updated
Appendix P – radiotherapy guidelines updated

20 REFERENCES

1. http://info.cancerresearchuk.org/cancerstats/types/prostate/incidence/

2. Goktas S, Crawford ED. Optimal hormonal therapy for advanced prostatic carcinoma. Semin.Oncol 1999;26(2):162-73.

3. Catalona WJ. Management of cancer of the prostate. N Engl J Med 1994;331(15):996-1004.

4. Scher HI, Kelly WK. Flutamide withdrawal syndrome: its impact on clinical trials in hormone-refractory prostate cancer. J Clin Oncol 1993;11(8):1566-72.

5. Vogelzang NJ. One hundred thirteen men with hormone-refractory prostate cancer died today. J Clin Oncol 1996;14(6):1753-5.

6. Posadas EM, Dahut WL, Gulley J. The emerging role of bisphosphonates in prostate cancer. Am.J Ther. 2004;11(1):60-73.

7. Morris MJ, Scher HI. Optimizing targeted therapy and developing novel outcome measures for patients with advanced prostate cancer at Memorial Sloan-Kettering Cancer Center. Crit Rev.Oncol Hematol. 2003;46 Suppl:S21-S31.

8. Riccardi A, Grasso D, Danova M. Bisphosphonates in oncology: physiopathologic bases and clinical activity. Tumori 2003;89(3):223-36.

9. Green JR, Muller K, Jaeggi KA. Preclinical pharmacology of CGP 42'446, a new, potent, heterocyclic bisphosphonate compound. J.Bone Miner.Res. 1994;9(5):745-51.

10. Major P, Lortholary A, Hon J, Abdi E, Mills G, Menssen HD et al. Zoledronic acid is superior to pamidronate in the treatment of hypercalcemia of malignancy: a pooled analysis of two randomized, controlled clinical trials. J Clin Oncol 2001;19(2):558-67.

11. Santini D, Vesasiani Gentilucci A, and Vincenzi A. The antineoplastic role of bisphosphonates: from basic research to clinical evidence. Annals of Oncology 14, 1468-1476. 2003.

12. Rosen LS, Gordon D, Kaminski M, Howell A, Belch A, Mackey J et al. Long-term efficacy and safety of zoledronic acid compared with pamidronate disodium in the treatment of skeletal complications in patients with advanced multiple myeloma or breast carcinoma: a randomized, double-blind, multicenter, comparative trial. Cancer 2003;98(8):1735-44.

13. Saad F, Gleason DM, Murray R, Tchekmedyian S, Venner P, Lacombe L et al. A randomized, placebo-controlled trial of zoledronic acid in patients with hormone-refractory metastatic prostate carcinoma. J Natl.Cancer Inst. 2002;94(19):1458-68.

14. Dearnaley DP, Sydes MR, Mason MD, Stott M, Powell CS, Robinson AC et al. A double-blind, placebo-controlled, randomized trial of oral sodium clodronate for metastatic prostate cancer (MRC PR05 Trial). J Natl.Cancer Inst. 2003;95(17):1300-11.

15. Khan MA, Carducci MA, Partin AW. The evolving role of docetaxel in the management of androgen independent prostate cancer. J Urol. 2003;170(5):1709-16.

16. Petrylak DP, Macarthur RB, O'Connor J, Shelton G, Judge T, Balog J et al. Phase I trial of docetaxel with estramustine in androgen-independent prostate cancer. J Clin Oncol 1999;17(3):958-67.

17. Picus J, Schultz M. Docetaxel (Taxotere) as monotherapy in the treatment of hormone-refractory prostate cancer: preliminary results. Semin.Oncol 1999;26(5 Suppl 17):14-8.

18. Beer TM, El Geneidi M, Eilers KM. Docetaxel (taxotere) in the treatment of prostate cancer. Expert.Rev.Anticancer Ther. 2003;3(3):261-8.

19. Kreis W, Budman DR, Fetten J, Gonzales AL, Barile B, Vinciguerra V. Phase I trial of the combination of daily estramustine phosphate and intermittent docetaxel in patients with metastatic hormone refractory prostate carcinoma. Ann.Oncol 1999;10(1):33-8.

20. Piccart MJ, Klijn J, Paridaens R, Nooij M, Mauriac L, Coleman R et al. Corticosteroids significantly delay the onset of docetaxel-induced fluid retention: final results of a randomized study of the European Organization for Research and Treatment of Cancer Investigational Drug Branch for Breast Cancer. J Clin Oncol 1997;15(9):3149-55.

21. Oudard, S., Beuzeboc, P., Dourthe, L. M., and et al. Preliminary results of a phase II randomized trial of docetaxel (D), estramustine (E) and prednisolone (P) - two schedules - versus mitoxantrone (M) and prednisone in patients with Hormone Refractory Prostate Cancer (HRPC). Proc Am Soc Clin Oncol 21, 117a-(Abstract No. 706). 2002.

22. Petrylak DP, Tangen CM, Hussain MH, Lara PN, Jr., Jones JA, Taplin ME et al. Docetaxel and estramustine compared with mitoxantrone and prednisone for advanced refractory prostate cancer. N Engl J Med 2004;351(15):1513-20.

23. Tannock IF, de Wit R, Berry WR, Horti J, Pluzanska A, Chi KN et al. Docetaxel plus prednisone or mitoxantrone plus prednisone for advanced prostate cancer. N Engl J Med 2004;351(15):1502-12.

24. Hawkey CJ. COX-2 inhibitors. Lancet 1999;353(9149):307-14.

25. Taketo MM. Cyclooxygenase-2 inhibitors in tumorigenesis (Part II). J Natl.Cancer Inst. 1998;90(21):1609-20.

26. Taketo MM. Cyclooxygenase-2 inhibitors in tumorigenesis (part I). J Natl.Cancer Inst. 1998;90(20):1529-36.

27. Nelson JE, Harris RE. Inverse association of prostate cancer and non-steroidal anti-inflammatory drugs (NSAIDs): results of a case-control study. Oncol Rep. 2000;7(1):169-70.

28. Eberhart CE, Coffey RJ, Radhika A, Giardiello FM, Ferrenbach S, DuBois RN. Up-regulation of cyclooxygenase 2 gene expression in human colorectal adenomas and adenocarcinomas. Gastroenterology 1994;107(4):1183-8.

29. Roberts RO, Jacobson DJ, Girman CJ, Rhodes T, Lieber MM, Jacobsen SJ. A population-based study of daily nonsteroidal anti-inflammatory drug use and prostate cancer. Mayo Clin Proc. 2002;77(3):219-25.

30. Basler JW, Piazza GA. Nonsteroidal anti-inflammatory drugs and cyclooxygenase-2 selective inhibitors for prostate cancer chemoprevention. J Urol. 2004;171(2 Pt 2):S59-S62.

31. Masferrer JL, Leahy KM, Koki AT, Zweifel BS, Settle SL, Woerner BM et al. Antiangiogenic and antitumor activities of cyclooxygenase-2 inhibitors. Cancer Res. 2000;60(5):1306-11.

32. Hsu AL, Ching TT, Wang DS, Song X, Rangnekar VM, Chen CS. The cyclooxygenase-2 inhibitor celecoxib induces apoptosis by blocking Akt activation in human prostate cancer cells independently of Bcl-2. J Biol.Chem. 2000;275(15):11397-403.

33. Steinbach G, Lynch PM, Phillips RK, Wallace MH, Hawk E, Gordon GB et al. The effect of celecoxib, a cyclooxygenase-2 inhibitor, in familial adenomatous polyposis. N.Engl.J.Med. 2000;342(26):1946-52.

34. Solomon SD, McMurray JJ, Pfeffer MA, Wittes J, Fowler R, Finn P et al. Cardiovascular risk associated with celecoxib in a clinical trial for colorectal adenoma prevention. N Engl J Med 2005;352(11):1071-80.

35. Boissier S, Ferreras M, Peyruchaud O, Magnetto S, Ebetino FH, Colombel M et al. Bisphosphonates inhibit breast and prostate carcinoma cell invasion, an early event in the formation of bone metastases. Cancer Res. 2000;60(11):2949-54.

36. Corey E, Brown LG, Quinn JE, Poot M, Roudier MP, Higano CS et al. Zoledronic acid exhibits inhibitory effects on osteoblastic and osteolytic metastases of prostate cancer. Clin Cancer Res. 2003;9(1):295-306.

37. Witters LM, Crispino J, Fraterrigo T, Green J, Lipton A. Effect of the combination of docetaxel, zoledronic acid, and a COX-2 inhibitor on the growth of human breast cancer cell lines. Am.J Clin Oncol 2003;26(4):S92-S97.

38. Hiraga T, Ueda A, Tamura D, Hata K, Ikeda F, Williams PJ et al. Effects of oral UFT combined with or without zoledronic acid on bone metastasis in the 4T1/luc mouse breast cancer. Int.J Cancer 2003;106(6):973-9.

39. Neville-Webbe HL, Rostami-Hodjegan A, Evans CA, Coleman RE, Holen I. Sequence- and schedule-dependent enhancement of zoledronic acid induced apoptosis by doxorubicin in breast and prostate cancer cells. Int.J Cancer 2005;113(3):364-71.

40. Royston P, Parmar MK, Qian W. Novel designs for multi-arm clinical trials with survival outcomes with an application in ovarian cancer. Stat.Med 2003;22(14):2239-56.

41. Royston, P. stage2- program. MRC Clinical Trials Unit. 2001.

42. kind, P. The EuroQoL instrument: an index of health-related quality of life. In Spilker B, Quality of Life and Pharmaeconomics in Clinical Trials, Philadelphia: Lippincott-Raven. 1996.

43. Therasse P, Arbuck SG, Eisenhauer EA, Wanders J, Kaplan RS, Rubinstein L et al. New guidelines to evaluate the response to treatment in solid tumors. European Organization for Research and Treatment of Cancer, National Cancer Institute of the United States, National Cancer Institute of Canada. J Natl.Cancer Inst. 2000;92(3):205-16.

44. Dolan, P, Gudex, C, kind, P, and et al. A Social Tariff for EuroQol: Results from a UK General Population Survey. Centre for Health Economics Discussion Paper 138. Centre for Health Economics, University of York: CHE. 1995.
